# Large-Scale Range Collapse of Hawaiian Forest Birds under Climate Change and the Need 21^st^ Century Conservation Options

**DOI:** 10.1371/journal.pone.0140389

**Published:** 2015-10-28

**Authors:** Lucas B. Fortini, Adam E. Vorsino, Fred A. Amidon, Eben H. Paxton, James D. Jacobi

**Affiliations:** 1 U.S. Geological Survey, Pacific Island Ecosystems Research Center, Honolulu, Hawaii, United States of America; 2 Pacific Islands Climate Change Cooperative, Honolulu, Hawaii, United States of America; 3 U.S. Fish & Wildlife Service, Strategic Habitat Conservation Division, Pacific Islands Office, Honolulu, Hawaii, United States of America; Florida State University, UNITED STATES

## Abstract

Hawaiian forest birds serve as an ideal group to explore the extent of climate change impacts on at-risk species. Avian malaria constrains many remaining Hawaiian forest bird species to high elevations where temperatures are too cool for malaria’s life cycle and its principal mosquito vector. The impact of climate change on Hawaiian forest birds has been a recent focus of Hawaiian conservation biology, and has centered on the links between climate and avian malaria. To elucidate the differential impacts of projected climate shifts on species with known varying niches, disease resistance and tolerance, we use a comprehensive database of species sightings, regional climate projections and ensemble distribution models to project distribution shifts for all Hawaiian forest bird species. We illustrate that, under a likely scenario of continued disease-driven distribution limitation, all 10 species with highly reliable models (mostly narrow-ranged, single-island endemics) are expected to lose >50% of their range by 2100. Of those, three are expected to lose all range and three others are expected to lose >90% of their range. Projected range loss was smaller for several of the more widespread species; however improved data and models are necessary to refine future projections. Like other at-risk species, Hawaiian forest birds have specific habitat requirements that limit the possibility of range expansion for most species, as projected expansion is frequently in areas where forest habitat is presently not available (such as recent lava flows). Given the large projected range losses for all species, protecting high elevation forest alone is not an adequate long-term strategy for many species under climate change. We describe the types of additional conservation actions practitioners will likely need to consider, while providing results to help with such considerations.

## Introduction

A key challenge in climate change adaptation planning is projecting how changes in climate will affect efforts to conserve biological communities, and in particular species already under threat. While the projected effects of climate change on individual species may vary from beneficial to deleterious [1], for rare species already at risk of extinction (hereinafter, at-risk species), the projected effects of climate change are generally thought to accelerate declines [2,3]. Hawaiian forest birds are a group of at-risk species that are likely to suffer significant disease-driven impacts from climate change [4–6]. Of the 46 species of forest birds extant at the time of European contact in 1778, only 21 are still extant with 1 species occurring only in captivity. Disease, particularly avian malaria, constrains many native forest birds to high elevations where temperatures are too cool for its life cycle and principal mosquito vector *Culex quinquefasciatus* [5]. Thus, native Hawaiian forest birds are mostly restricted to a narrow band of forests pressed against the top of available habitat [4]. The biological and cultural significance of forest birds have placed them at the core of conservation efforts in Hawai`i. On one hand, the uniqueness and diversity of this group, reached through adaptive radiation greatly surpassing that of the well-known Galapagos Finches [7], make forest birds emblematic of Hawaiian ecosystems. On the other hand, the importance of these birds to Hawaiian culture [8] adds another level of significance to their long-term conservation.

Hawaiian forest birds serve as an ideal group to explore the extent of climate change impacts on at-risk species. Firstly, due to their conservation importance, forest birds have a large set of observation records from decades of comprehensive field surveys [9,10] and are arguably one of the most well-studied species group in Hawai’i. Secondly, temperature, a primary variable defining the distribution of avian malaria, is better understood than precipitation both globally and regionally [11,12]. Because of avian malaria, observed [13,14] and projected [15–17] rapid temperature increases at high elevations are expected to have clear implications to these high elevation bird species. Lastly, Hawai`i’s steep environmental gradients [18] along with naturally small ranges of insular species [19] compress climatic and ecological variability, making Hawai`i ideal to study the link between species response and climate variation.

The strong relationship between temperature, avian malaria, and vector distribution has allowed researchers to bring attention to how climate change will facilitate the movement of malaria into increasingly higher elevations over time [4,5,20]. However, up to now there were no analyses of climate change impacts on the distribution of each individual Hawaiian forest bird species. While an alternative SDM analysis of mosquitoes [21,22] could offer insights into some general distributional restrictions of forest birds, it alone would provide limited insight into potential impacts of climate shifts on individual forest bird species for three reasons. First, the distribution of avian malaria is not only determined by mosquito presence but by climatic limits to the *Plasmodium* life cycle. Second, a mosquito-based analysis would still ignore known differences in bird species’ resistance and tolerance to disease. Third, mosquito distribution records are negligible in number when compared to the decades of data collected for forest bird species. Hence, to elucidate the differential impacts of expected climate shifts on species that vary significantly in niche, disease resistance and tolerance, we directly project distribution shifts for all Hawaiian forest bird species. Under a likely scenario of continued disease-driven distribution limitation, we illustrate the potential impacts of a mid-range end-of-century climate warming scenario on these at-risk species, provide detailed information for conservation planning including the identification of priority areas for protection and restoration, and provide an underlying framework for a necessary climate-focused conservation response to projected impacts.

## Materials and Methods

### Species description and location data

In our analyses, we included all extant native passerine bird species that occur in the wild across the main Hawaiian Islands. This resulted in the exclusion of `alalā (currently only in a captive population) and other species that only occur on the small Northwestern Hawaiian Islands. We further disregarded sub-species differences for Hawai`i `Amakihi, resulting in a total 20 species considered from three families and subfamilies (*Drepanidinae*, *Monarchidae*, *Turdidae*).

Systematic bird surveys have been conducted regularly across the Hawaiian Islands, starting with an archipelago-wide series of surveys across most forest bird habitat in the late 1970s through early 1980s, with subsequent surveys every 1–5 years in many key regions. These records were obtained from the Hawaii Forest Bird Interagency Database [23], which includes point transect data from Scott et al. [9] as well as regional and species-specific surveys conducted after the Hawaiian Forest Bird Survey (e.g., [10,24]). We also obtained playback survey data from O`ahu `Elepaio surveys [25,26], point transect survey data from the Natural Areas Reserve Survey Database for Laupahoehoe (Hawaii Division of Forestry and Wildlife, unpubl. data), and incidental observations obtained from ornithologists on Kauai (O. Johnson, unpubl. data) to help fill in gaps in surveys on some islands. We collected a total of 42,051 presence records for the 20 extant Hawaiian forest birds used as presence data in our models. Additionally, in the cases where survey stations were sampled multiple times over the last 3 decades (31% of 15,415 stations), a species was deemed present at that location if it was sighted at least once during any of the surveys.

Because forest bird surveys were designed to maximize detection of native forest birds, focusing on middle to high elevation forests, they did not adequately cover areas where birds were believed absent or occurring at low densities. As a result, species absence information from point-count survey stations was not adequately spread across the landscape to be included in our models. Consequently, all of our presence-only models utilized randomly generated pseudo-absence points (PAs) along with the compiled presence data. Presence-only SDM methods are increasingly common and, given the challenges of adequately quantifying real absences [27], have been shown to give adequate model predictions compared to presence-absence methods [28,29]. Since we limited the models for each species to the islands they are known to currently occur, to account for differences of analysis extent across species we generated PAs at an average density of 1 per 3.125 km^2^ across the islands considered. This resulted in a minimum of 500 PAs selected per model run for small island (O`ahu, Kaua`i) endemic birds. This is a density that preliminary analysis indicated yielded stable results while minimizing model computations. Our models utilized several PA draws over model iterations, placing them randomly across the entire island(s) upon which each modeled species was known to occur, but at least 500m from known presence locations.

Expert-derived range maps for each species were developed using recent survey data, field knowledge, elevation and habitat information. We used these expert range maps to qualitatively assess whether our models reflected broad expected distribution patterns beyond conventional model evaluation statistics.

### Species distribution modeling approach

We employed an ensemble approach to model individual species’ current and end-of-century distributions [30,31]. By using this methodology we minimized the potential impact of model choice and configuration by combining projections from different SDM methodologies weighted by model performance. We based our ensemble models on two approaches (Generalized boosting model, GBM; and Maximum entropy, MaxEnt) because of their recognized predictive accuracy [32,33] and because preliminary analyses indicated that they performed well across our multiple study species. MaxEnt is a common SDM approach that compares the projected distribution of occurrence localities, as projected over the environmental predictors, to a null distribution (as defined by pseudo-absences) of the predictors [34]. A GBM is a powerful classification tree learning methodology that attempts to improve the predictive accuracy of decision trees through boosting [35,36].

To create the ensemble models for each species, we performed a total of 240 model runs for each model approach, a total of 480 runs per species. For each model run, 20 percent of the data was withheld for model evaluation. As recommended by Franklin [37] and Elith and Leathwick [27] multiple test statistics were used to allow a more robust assessment of model performance and validate model responses. All models were evaluated using Receiver Operating Characteristic (ROC) and True Skill Statistics (TSS) evaluation criteria. ROC measures the ability of the forecast to discriminate between two alternate outcomes, and can thus be considered as a measure of the model’s potential utility [38]. ROC scores range between 0 and 1, with a score of 0.5 indicating a model as good as a random model. TSS evaluates how well models separate positive (presence) from negative (absence) events, independent of event frequency. TSS scores range from -1 to 1, with a score of 0 indicating a model with no skill and thus unable to distinguish between observed events. The final ensemble projection for each species was created by averaging across all individual model runs using a weighted mean based on evaluation statistic scores. Unless otherwise stated all results presented are based on the ROC evaluation criteria, due to the similarity of results across ROC and TSS evaluation criteria. While our models’ primary projection outputs are maps indicating continuous habitat suitability scores between 0 and 1, we focus all discussions on modeled presence instead. Modeled presence was calculated by applying a threshold to habitat suitability scores, leading to equivalent sensitivity/ specificity when compared to the model evaluation data [38,39]. This was done to simplify model output interpretation as modeled presence results are more easily compared among species. Maps of habitat suitability for all species are available in S1 File. To understand the relative importance of individual predictors to each model, we developed species-specific metrics of variable importance [40] that show the significance of each predictor to projected distributions. All models presented were fit using the ‘biomod2’ R package [31]. Biomod2 is a species distribution modeling platform in which multi-model ensemble modeling, calibration, forecasting, and statistical analyses can be conducted iteratively. A complete set of R scripts including all model configurations and a test data set are included in the git repository version below (https://github.com/brasilbrasil/Ensemble_SDM/commit/d8dd885b5bb8a05577116bdcce1577b5e1ed3747).

### Environmental predictors

We attempted to select predictors that described the mean and variation in temperature and rainfall, as these are factors known to impact the distribution of forest bird habitat and avian malaria [4,41]. We derived bioclimatic variables used as predictors in our models from current and future monthly minimum and maximum temperature (Tmin, Tmax) and precipitation data. We obtained current monthly climate data at 250m resolution and spatially aggregated it to 500m to improve model processing time [18,42]. We then summarized the large volume of monthly data into a suite of 19 standard bioclimatic variables that are commonly used in niche modeling due to their biological relevance [43]. To minimize multi-collinearity [44], we selected a minimum number of predictors that described temperature and precipitation means and variability by considering Pearson correlation coefficients between all pairs of bioclimatic variables for the complete extent of the Hawaiian Islands. We then selected four bioclimatic variables with low correlation coefficients (<0.63) as the sole predictors for each of our SDMs: mean annual temperature (MAT; Bio1), mean annual precipitation (MAP; Bio12), temperature annual range (Bio7), and precipitation seasonality (monthly coefficient of variation; Bio15). As with all other analyses and modeling presented, we calculated bioclimatic variables using the R statistical environment [45]. The R package ‘dismo’ provided methods for bioclimatic variable generation based on rainfall and temperature data [44].

We calculated future bioclimatic predictors using the same procedure as for the baseline data. However, we calculated end of century values for monthly Tmin, Tmax and precipitation by integrating climate projections with current climate estimates. To do that, we first calculated the projected change between 1990–2010 and 2080–2100 for each monthly variable developed from the Hawaiian Regional Climate Model (HRCM model with 1 km spatial resolution for Maui and 3 km spatial resolution for all other islands; [17]). We then added these delta values to current monthly climate values before recalculating all bioclimatic variables. The HRCM-based climate projections show an average of 2.5°C warming over the islands, but with a clearly increased warming at higher vs. lower elevations (3.4°C vs. 2.2°C, respectively). General precipitation shifts include increased precipitation in windward, wet areas of Hawai`i and Maui, with generally slight drying trends across the drier areas of the state.

The HRCM is based on the Weather Research and Forecasting model V3.3. It considers future climate forcing based on the SRES A1B emission scenario and the mean of multiple CMIP3 global circulation models. The HRCM simulations replicate the regional and island climate mechanisms that largely dictate local climate such as extreme orographic-based precipitation gradients and trade wind inversions [17]. As such, the computing requirements to run the HRCM simulations limit our analyses to a single future climate scenario. However, preliminary results showed that interpolated GCM projections, such as those commonly used in continental SDM analyses, are of very limited value for a small, hyper-diverse climatic region such as Hawai`i.

### Determining reliability of species models

Because of the clear implications of our results to Hawai`i forest bird conservation efforts, we attempted to comprehensively determine the reliability of our species models beyond standard model evaluation metrics. We determined model reliability by considering three criteria: two criteria related to the robustness of links between species distribution and climate and a third criterion describing the adequacy of location data underlying species models. We applied a highly conservative threshold of 0.9 to our ROC model evaluation scores to indicate which species had distributions very strongly associated with climate variability. We also identified species whose distributions were primarily determined by MAT as those in which we had a greater confidence of their future projected distributions. We did this for two reasons. First, as the purpose of our effort was to project distribution change for species, the current uncertainties in global and regional precipitation projections [11,12] make future distribution projections from precipitation-sensitive models more uncertain. Additionally, while we understand the strong link between rising temperatures and disease prevalence [4,5], the mechanism linking species distributions to other climate variables is less simple and clear [5,6,46], making the assumption that these mechanisms will hold under a future climate more tenuous.

Determining the comprehensiveness of species location data in relation to suitable climate space is important because truncated models representing only a portion a species’ climate range can still have high model evaluation scores. This was a clear possibility for the most common species since bird surveys have often focused on a subset of areas where the rarest species occur. This truncation can also arise for species that occur at the abrupt physical limits of island climate (e.g., `Akeke`e inhabiting the top of Kaua`i) or for species where large portions of their suitable climate space has been lost due to habitat conversion (e.g., lower elevation Palila or O`ahu `Elepaio habitat converted to pasture and other land uses). To determine if location data encompassed the full range of each species modeled, using presence/absence data from all locations for each species, we compared overall species prevalence (proportion of total points a species was sought in which the species was actually observed) with prevalence at the low and high ends of our climate predictors (using <2.5% and >97.5% quantile values). In this simplified analyses we classified species as being comprehensively sampled if they had prevalence at the limits of surveyed climate < 50% of the overall species prevalence, with prevalence weighted by climate predictor importance. This was done as we naturally expected the prevalence at the limits of surveyed climate predictors to be substantially smaller than overall prevalence.

All SDMs that passed the three criteria above were classified as highly reliable. All other SDMs were classified as having reduced model reliability. This categorization provides a conservative and easy to understand evaluation of model reliability that is more comprehensive than model evaluation that typically focuses on model fitting performance.

### Species climate-based range changes in context of current habitat availability

As commonly done elsewhere, our SDMs utilized only climate predictors, hence we refer to all of our resulting projected species distributions as a climate-based ranges, while recognizing actual ranges may be different due to other habitat restrictions. Given the dependence of bird species on forest habitat, we analyzed all species range shifts with respect to the distribution of currently available primary habitat for each species, assuming no future change in primary habitat distribution. We defined the primary habitat for each species as the vegetation cover classes that account for at least 90% of all species sightings. For this analysis we generated a vegetation cover map based on Landfire cover maps [47]. We simplified the original Landfire classes into broad association-level categories (e.g., wet forest, mesic shrubland) and combined all anthropogenic classes (e.g., urban) and other minor classes (e.g., wetland) into a single ‘other’ category. Additionally, because of the spatial scale of the climate data used, and the known fine-scale errors of the original Landfire maps, we aggregated our land cover map from 30m resolution to a 500m resolution using the dominant cover type at each pixel. In determining the classes that make up primary habitat for each species, we discarded the ‘other’ category, as sightings associated with this class were a result of other forest bird habitat being averaged over during the aggregation to 500m. Lastly, the original Landfire classification contained wet forest classes, mesic forest classes, and a much smaller mixed mesic/wet forest class for non-native cover. We included this small class as part of primary habitat for a species any time both wet and mesic forests contributed to the primary habitat for a species.

### Reverse future projections to check model over-fitting

Given the consistent future range decline across all modeled species (*see* results), we performed a simple (and novel) check for potential model over-fitting that compliments the standard approaches used to evaluate and limit model over-fitting. Since over-fitting may result in range declines under scenarios deviating from baseline conditions, we projected all species models onto a hypothetical reverse future climate scenario. This hypothetical climate scenario reflects the opposite of future projected changes for each predictor variable used in our models (e.g., a location with a projected 2°C MAT increase is assigned a 2°C MAT decrease, and so forth). Unless the projected climate shifts are larger than the climatic niche for all species, consistent range declines across all species under this reverse scenario could be taken as a sign of over-fitting of our species models. While this is admittedly a simplistic and limited test, it is nevertheless a very easy and intuitive check on model behavior under alternate climate scenarios.

## Results

### Determining the reliability of species models

Considering our three criteria, we deemed 10 of our 20 models for extant forest bird species to be highly reliable. These species have distributions that are very strongly linked to climate variability (high ROC and TSS scores), especially MAT, with models based on surveys that adequately represent their climatic range (Table 1). The ROC and TSS evaluation scores of the SDMs for all species were very high, indicating a strong dependence on the selected climatic variables (S2 File). However, some common species (`Apapane, Hawai`i `Amakihi and Hawai`i `Elepaio) had consistently lower evaluation scores, below our conservative 0.9 ROC threshold. All of these species are thought to have a greater tolerance/resistance to avian malaria and thus are expected to be less climatically constrained than all other species [6,48,49]. For O`ahu `Amakihi, O`ahu `Elepaio, `Oma`o and Palila, MAT was not consistently the best predictor of distribution of the species across model types (S3 File) making the projected future distribution of these species less certain. With regard to the adequacy of the data used to fit our models, several common species (e.g., all `Amakihi and `Elepaio species) showed high prevalence even at extremes of their climate distribution, indicating their modeled distributions may be truncated (Table 1; S5 File).

**Table 1:** Species model reliability based on three criteria.

Hawaiian name	Scientific name	Climate driven distribution (ROC ≥0.9^1^)	Distribution primarily driven by MAT^2^	Comprehensiveness of survey within suitable climate space ^3^	Overall model reliability^4^
`Akeke`e	*Loxops caeruleirostris*	X	X	X	High
`Akiapōlā`au	*Hemignathus wilsoni*	X	X	X	High
`Akikiki	*Oreomystis bairdi*	X	X	X	High
`Ākohekohe	*Palmeria dolei*	X	X	X	High
Hawai`i `Ākepa	*Loxops coccineus*	X	X	X	High
Hawai`i Creeper	*Oreomystis mana*	X	X	X	High
`I`iwi	*Drepanis coccinea*	X	X	X	High
Maui `Alauahio	*Paroreomyza Montana*	X	X	X	High
Maui Parrotbill	*Pseudonestor xanthophrys*	X	X	X	High
Puaiohi	*Myadestes palmeri*	X	X	X	High
`Anianiau	*Hemignathus parvus*	X	X		Reduced
`Apapane	*Himatione sanguinea*		X		Reduced
Hawai`i `Amakihi	*Chlorodrepanis virens*		X		Reduced
Hawai`i `Elepaio	*Chasiempis sandwichensis*		X		Reduced
Kauai `Amakihi	*Chlorodrepanis stejnegeri*	X	X		Reduced
Kauai `Elepaio	*Chasiempis sandwichensis*	X	X		Reduced
O`ahu `Amakihi	*Chlorodrepanis flava*	X			Reduced
O`ahu `Elepaio	*Chasiempis sandwichensis*	X			Reduced
`Oma`o	*Myadestes obscurus*	X		X	Reduced
Palila	*Loxioides bailleui*	X			Reduced

^1^ Indicative of distribution strongly associated with climate;

^2^ MAT mean annual temperature;

^3^ Distribution of survey locations along climate gradients;

^4^ All species with SDMs that passed the three criteria were classified as highly reliable. All other SDMs were classified as having reduced model reliability.

As a check of our model reliability categorization, all our species with high model reliability had current climate-based distributions that matched the broad geographical patterns of expert-derived current range maps (S4 File). More specifically, agreement between modeled current distributions and expert-derived current range maps was significantly higher for our high model reliability species than our reduced model reliability species (97% vs 88% agreement, p< = 0.0006). The remaining 10 reduced model reliability species still have high TSS and ROC scores relative to similar SDM analyses elsewhere. As such, these models offer useful ecological insights and may be substantially improved with additional species location data and model improvements (e.g., consideration of other meaningful environmental predictors). To discourage the use of these models in conservation planning, except for the second figure in this manuscript, all distribution shift results for the species with reduced model reliability are only included in the manuscript’s S1–S8 Files.

Two additional analyses reinforce our assessment of the overall high performance of our species SDMs. First, considering our climate-based SDMs projections alone, despite range declines across all species by 2100 (67% mean range loss), our reverse projections yield a mean 55.7% climate-based range gain across all species under a hypothetical reverse climate shift. These results demonstrate that our models are not overly conservative due to potential over-fitting. Additionally, further analysis showed that nearly all areas of potential future forest bird distribution across the state do not fall within non-analogue climate areas that are known to pose challenges to SDM projections elsewhere ([50]; S5 File).

### Predicting climate-based species distribution shifts

**Fig 1:**
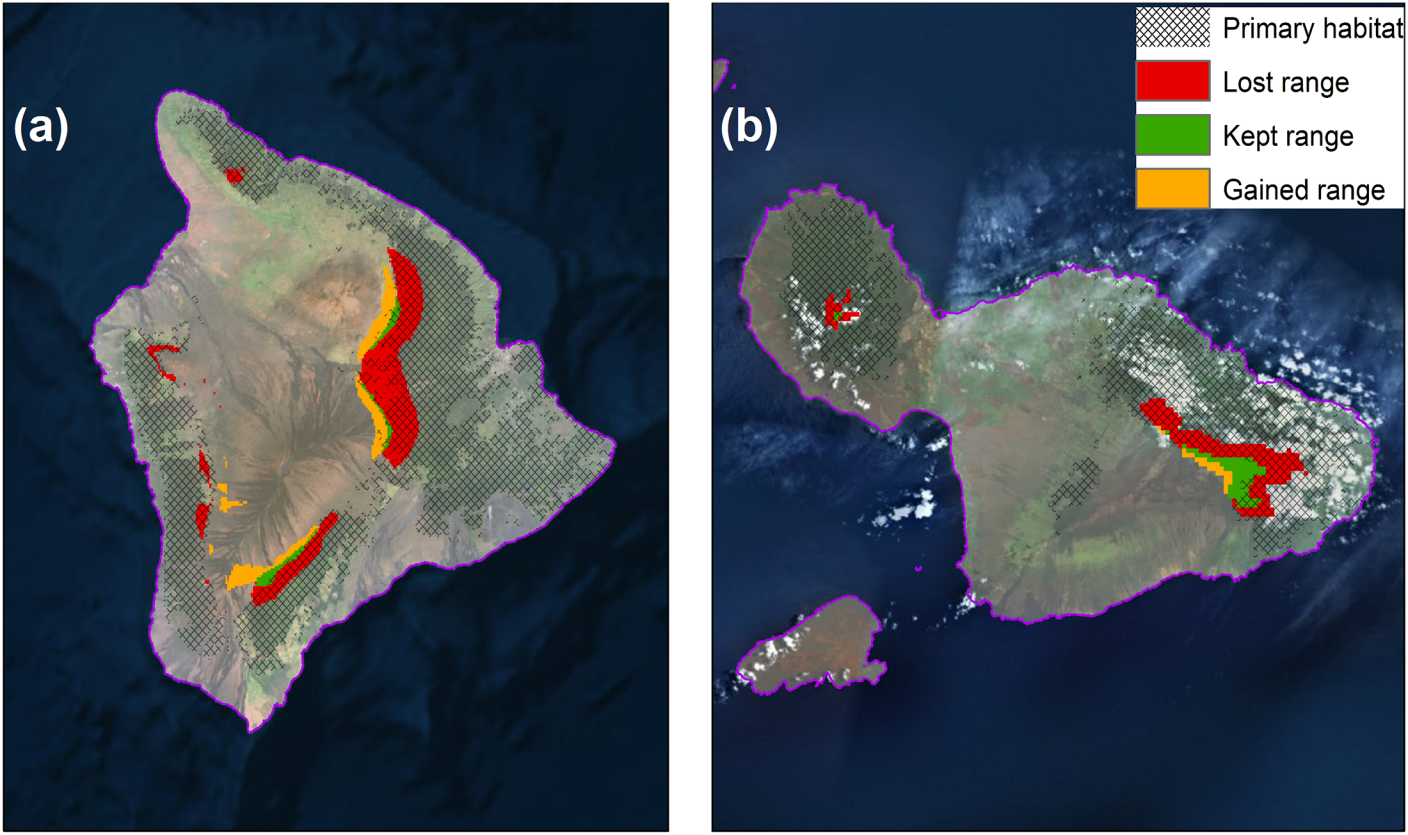
Projected climate-based range change between 1990–2010 and 2080–2100 for Hawai`i `Ākepa (left) and Maui Parrotbill (right). The gridded overlay represents the distribution of primary vegetation types associated with the species (S6 File). The pink overlay shows the spatial configuration of the main Hawaiian Islands.

**Fig 2:**
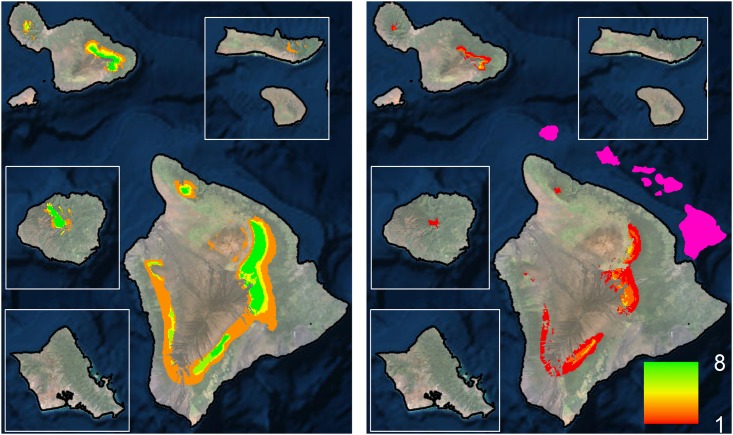
Current (left) and future (right) forest bird number of species based on modeled range and available primary habitat of high model reliability species. The pink overlay shows the spatial configuration of the main Hawaiian Islands.

**Fig 3:**
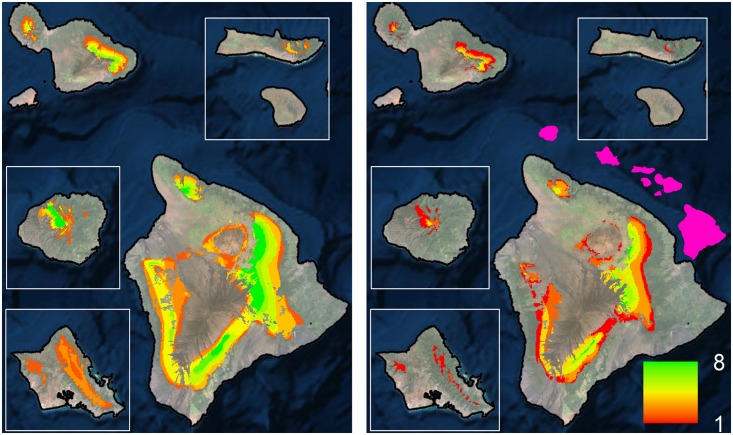
Current (left) and future (right) forest bird number of species based on modeled range and available primary habitat of all extant species. The pink overlay shows the spatial configuration of the main Hawaiian Islands.

To provide more ecologically accurate estimates of projected range shifts, we considered our modeled species distributions in the context of available forest bird habitat (Fig 1). Within this context, 9 out of our 10 high model reliability species are projected to suffer >75% range loss, with all species suffering >50% range loss (Table 2; S6 File). Additionally, three species are projected to lose all of their range (`Akeke`e, `Akikiki and Puaiohi) and three others are projected to lose more than 90% of their range (Hawai`i `Ākepa, `Ākohekohe and Maui Parrotbill). In general, range losses are highest for the single island endemics with small current ranges within the high model reliability species category. Considering only high model reliability species, our results show declines in forest bird richness with clear range contractions across all species (Fig 2). Considering all extant Hawaiian forest bird species together, the patterns of change are also similar (Fig 3), but also include smaller projected range losses for common species in the reduced model reliability group (S8 File). However, the lower model reliability for those species reduces their projection confidence and warrants further research. Overall, there are few areas of forest bird range expansion, primarily in the island of Hawai`i, and many areas of range loss across the entire archipelago (S7 File).

**Table 2:** Projected changes between 1990–2010 and 2080–2100 in ranges of high model reliability species, limited to current available primary habitat. All range estimates are in km^2^. Similar estimates for reduced model reliability species are included in S8 File.

Species	Baseline range	Future range	% range change	Range lost	Range kept	Range gained
`Akeke`e	70	0	-100	70	0	0
`Akiapōlā`au	397	87	-78	381	16	71
`Akikiki	52	0	-100	52	0	0
`Ākohekohe	44	4	-92.1	41	4	0
Hawai`i `Ākepa	422	28	-93.4	404	19	10
Hawai`i Creeper	582	134	-76.9	468	114	21
`I`iwi	1852	743	-59.9	1214	638	105
Maui `Alauahio	102	26	-74.9	76	26	0
Maui Parrotbill	69	7	-89.9	62	7	0
Puaiohi	49	0	-100	49	0	0

If estimates were not restricted to primary habitat, our climate-based SDMs would over-predict range retention between now and end of century for several species (Fig 1, S6 File). Likewise, for most forest bird species, the small amount of predicted climate-based range expansion occurs beyond their primary habitat, including currently non-forested areas such as lava flows and grasslands (S7 File). All high model reliability species were found to be strongly associated with wet and mesic forests, but common species in the reduced model reliability group had a more diversified set of primary habitats (S5 File). While unforested areas can eventually develop into forests suitable for many of the forest bird species, it takes >100 years for adequate forest structure to develop [51–53], and even more time in drier zones.

## Discussion

The impact of climate change on Hawaiian forest birds has been a recent focus of Hawaiian conservation biology, and has centered on the links between climate and avian malaria [6,41,54]. Mechanistic models parameterized for specific Hawaii locations replicate the apparent climate-driven disease dynamics, showing implications of projected warming on Hawaiian forest birds [5,20]. Our results agree with such findings and expand on them by showing that this disease-driven climate sensitivity is clearly consistent with the current distribution of most at-risk Hawaiian forest bird species. Additionally, our models utilize these strong climate and distribution relationships to provide a robust set of projected changes of native forest bird richness across the Hawaiian landscape between now and the end of the century.

Downscaled end-of-century climate projections for Hawai`i based on a moderate A1B emission scenario [17] suggest an average 2.6°C warming in areas that Hawaiian forest birds currently inhabit. These predicted changes in climate are likely to have dramatic effects on the future distribution of Hawaiian forest birds due to an increase in the distribution of avian disease. For most modeled species, the climate-based range of forest birds is projected to contract, particularly at lower elevations, as has been suggested from past research on avian malaria and mosquito vectors [5,6]. For many of the species currently confined to the upper-elevation limits of available habitat, either pressed against the tree line on the islands of Maui and Hawai`i or at the mountain tops of the lower islands, range losses are projected to be severe.

Compared to past analyses of malaria disease dynamics [4], our models yield a much more detailed depiction of climate change impacts on Hawaiian forest birds. This is partly because our analysis spatially discerns substantial differences in distributional responses of species to climate. Evidence of this is seen in the diverse current distribution of forest birds and their projected range shifts. We find a range of predicted outcomes, from common species retaining a comparatively large portion of their current range, to rare species being more vulnerable than previously thought. These conclusions are particularly robust for most of the rare species that had high reliability models which resulted in very consistent projected shifts regardless of the modeling method used. In fact, considering the complete range collapse projected for `Akeke`e, `Akikiki and Puaiohi, our study offers a rare case in which our most extreme projections are among the ones with greatest reliability. For our reduced model reliability species, some of our models for common species may be less reliable in projecting future change as they may under-represent actual distributions due to less comprehensive surveys and weaker sensitivity of species distribution to climate. For some of these species, such as the more common Kaua`i species (Kaua`i `Amakihi, Kaua`i `Elepaio, and `Anianiau), more comprehensive surveys could likely improve model reliability considerably. For others, their weaker dependence on MAT, along with larger numbers of individuals, suggests greater innate or evolved tolerance / resistance to disease and the possibility of adaptation towards persistence in higher temperature areas (e.g., Hawai`i and O`ahu species of `Amakihi, as well as `Apapane, `Elepaio, and `Oma`o; [6,48,49]).

We found that the simple analysis of species-specific habitat preference can yield a much more refined image of current and future distributions than projections using climate predictors alone. While the inclusion of habitat availability has been found useful in past SDM analyses [55], we show that this inclusion is particularly important when projecting future distributions for species that require structurally complex habitat. Most forest birds already occupy nearly all disease-free suitable habitat, and it is unlikely that substantial new habitat of sufficient stature and complexity can emerge from currently non-forested areas, without aggressive and complex reforestation efforts [56,57]. It is still unknown how much vegetation community change may lag behind climate shifts, but estimates suggest it can be several decades [58]. This may be especially true for the bulk of forest bird habitat (i.e., wet and mesic forests) that may require many decades to develop into structurally complex forest bird habitat [51–53]. Even if we were to disregard this vegetation lag, the upward expansion of Hawaiian forests is unlikely given the projected changes in trade wind inversion heights with future warming [59,60].

Several aspects of our analyses are likely to underrepresent the degree of change in forest bird distributions by 2100, and therefore our estimates represent a conservative change projection. First, the A1B emission scenario used, originally devised as a ‘middle of the road’ scenario [61], seems increasingly optimistic given the continued increase in yearly global greenhouse gas emissions [62,63]. Thus, this degree of warming may occur before the end of century and probably does not represent a stable climate end point. Second, the inclusion of species location data from a wide time period and our handling of overlapping records makes our analysis rather optimistic as it ignores recently observed range contractions [10,13]. These data handling choices were based on two considerations. First, not all forest bird range has been comprehensively surveyed recently. Second, these conservative choices reduce the incentive to delay management responses due to uncertainty inherent in model projections. Finally, we do not account for any future habitat conversion or degradation, ongoing processes which have had large impacts on forest bird habitat in the past [64].

### The link between climate and forest bird distributions

Avian malaria is a well-documented mechanism that accounts for the strong relationship between climate and the distribution of Hawaii’s forest bird species. The strong relationship of Hawaiian forest birds to climate, especially the great influence of MAT in determining the distributions of these species, corroborate the importance, but not the exclusivity, of avian malaria in constraining their distribution [65,66]. Yet, no other threat to forest birds can clearly account for this strong climatic relationship of forest bird distributions. While habitat conversion and degradation tend to vary with respect to elevation (and hence temperature), these factors do not explain the distribution limits of our high model reliability species. First, in relation to lower elevation distribution limits, habitat conversion has happened primarily below elevations where high model reliability species currently occur. Second, while habitat degradation (primarily by invasive plants and ungulates) occurs at middle and high elevations, there are approximately 1400 square kilometers of middle elevation (500–2000m elev.) native forests where high model reliability species are absent, as well some high elevation degraded areas where native birds are routinely sighted.

For our high model reliability species, the strong likely disease-driven link between MAT and current distribution of the birds, along with very comprehensive surveys, gives us high confidence that future shifts in climate will result in shifting ranges of forest birds. Yet, for our disease-mediated future bird distribution projections to be borne out, the current relationship between climate, disease and bird distributions must hold. While some abundant species have in fact recently shown evidence of increased disease resistance (e.g., Hawai`i `Amakihi [6,48] and O`ahu `Amakihi [49]), other honeycreepers exposed to malaria have gone extinct (e.g. O’u) that were once more abundant than several of our high model reliability species.

### Conservation of extremely at-risk species under climate change

For Hawaiian forest birds, management has largely centered on conservation of high elevation habitat. Related efforts to control invasive species, eradicate ungulates and mammalian predators, and restore forests have in many cases been critical to successful forest bird conservation [67,68]. However, our research suggests that these strategies may not be sufficient to safeguard the most vulnerable forest birds, which our models reliably project will lose substantial climatic range by the end of the century. Without additional conservation actions, even intact native forests will be unsuitable for many forest bird species as more of their range is lost with increasing temperatures and the consequent spread of avian malaria and its vector. As research closes the knowledge gap of climate change impacts on at-risk species groups elsewhere [69], it is likely other extremely vulnerable species face similar situations, where common conservation tools that focus on habitat protection are unlikely to counter the impacts of projected climate change. In these cases, unless conservation managers are willing to risk future range collapse and extinction of species, a concerted effort to explore the viability of novel long-term solutions that decouple projected climate shifts from species declines must be started before it is too late to successfully implement them. Fortunately, whether novel actions are too late for these at-risk species partly depends on whether actions that “buy time” are simultaneously pursued while long-term management solutions are developed and implemented. Consequently, a long-term conservation strategy for at-risk species that minimizes projected climate change impacts requires three categories of conservation actions: (1) continue dealing with non-climate-related threats, (2) decouple climate from proximate causes of range loss, and (3) develop strategies that buy time. We describe each of these categories below and provide examples of how our model results can be directly relevant to Hawaiian forest bird conservation options.

**Fig 4:**
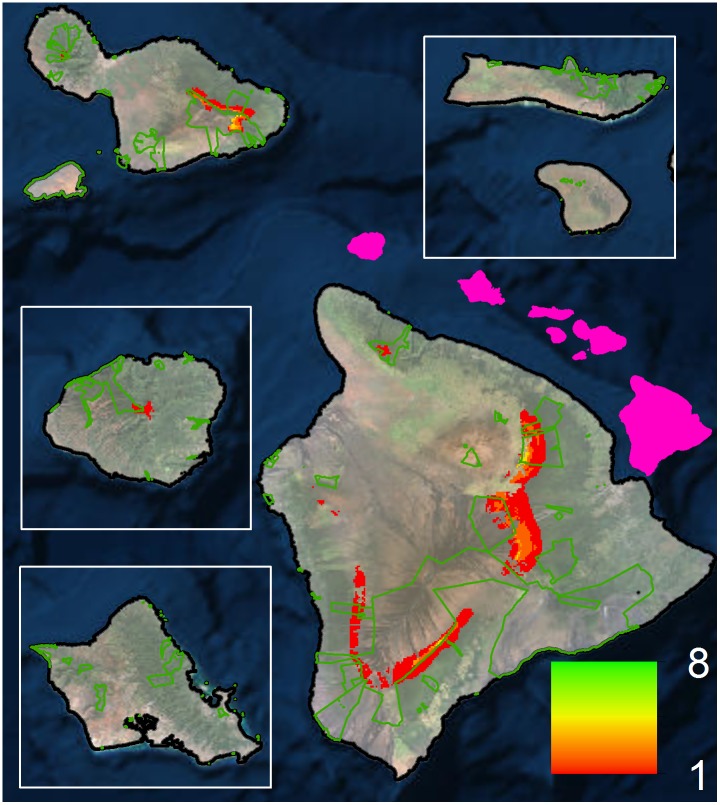
Potential priority areas for forest bird habitat conservation. Map is based on the number of high model reliability species projected to maintain their range between now and end of century. Current protected areas are delineated in green (National parks, State parks, Natural area reserves, wildlife refuges, sea bird sanctuaries, Nature Conservancy lands and other major private conservation areas). The pink overlay shows the spatial configuration of the main Hawaiian Islands. A similar figure including all extant species is included in S7 File.

Species at risk are commonly subject to multiple threats, both climate and non-climate related [70]. Focusing on the reduction of non-climatic threats is a climate adaptation strategy recommended for species where projected impacts are highly uncertain and/or not extreme [71,72]. For our highly vulnerable species, implementing such actions not only helps to ensure the persistence of these species, but also may provide larger numbers of individuals upon which natural selection may lead to adaptation to climate change and other stressors. For forest bird conservation, relevant actions include the full suite of conventional conservation tools aimed at reducing stressors (e.g., predators, habitat degradation), protection of high elevation habitat, and increasing food resources (e.g., through control of invasive wasps, control of competing invasive birds, and, for frugivorous birds, planting native fruit trees). Our distribution models can serve as powerful tools to help identify areas where these types of bird conservation efforts can be focused. For instance, by considering all high model reliability species, we have identified the areas where the greatest number of species is predicted to persist through 2100 (Fig 4). These maps could be used to target protection and conservation actions in areas that provide enduring forest bird habitat under climate change.

For species in which climate change impacts will lead to clear threats to their continued existence, such as several of our high model reliability species, effective conservation efforts must also include strategies that decouple proximate causes of range loss from climate. Given the importance of MAT in defining most forest bird species distributions across Hawai`i due to avian malaria, efforts to interrupt the cycle of malaria transmission and mortality are a long-term necessity. Such actions could include vector control and genetic modification of both birds and mosquitos. Many of these novel options have been discussed in the forest bird conservation community as potential solutions but have not been adequately considered and explored in terms of their costs, viability, and risks. Countering the incursion of disease into high elevation forests will likely require multiple approaches. Control of the mosquito vector through identification and elimination of larval habitat are traditional approaches to disease control [73]. Various forms of mosquito releases, especially developments in genetically modified mosquitoes, provide potential avenues to vector suppression or immunization [74,75]. Introducing certain strains of *Wolbachia sp*. into mosquitoes has previously shown promise in reducing disease transmission [76–78], but recent research hints to potential limitations of the approach to human malaria control [79]. The most long-term solution involves the development of malaria resistance or tolerance in the birds themselves. Monitoring of disease resistance in low elevation areas may allow us to identify populations more resistant or tolerant to disease, and could provide the opportunity to facilitate evolution via translocations of resistant individuals to more naive populations. In that respect, our modeled current distributions can help identify ‘outlier’ populations where increasing disease resistance or tolerance may be taking place. Lastly, while research on human malaria suggest complex genetics of disease resistance [80,81], more research on the genetic basis for bird disease resistance could lead the way to the transfer of genes responsible for disease resistance from high to low disease resistant species, as has been done for plant species [82,83].

Management options that decouple climate from proximate causes of range loss, if successful, could help forest birds repopulate huge swaths of habitat that are currently uninhabitable due to avian disease (dark grey areas in Fig 1). In fact, several species that are projected to lose most of their range by 2100 are known to have had much wider, low-elevation distributions across the archipelago (e.g., Puaiohi, `Akikiki, `I`iwi; [84]). Today, some of their former habitat persists but the birds are largely absent. While options to decouple climate from species declines are not fully developed, their impact would be analogous in principle to documented species recovery following the effective management of direct threats [85].

**Fig 5:**
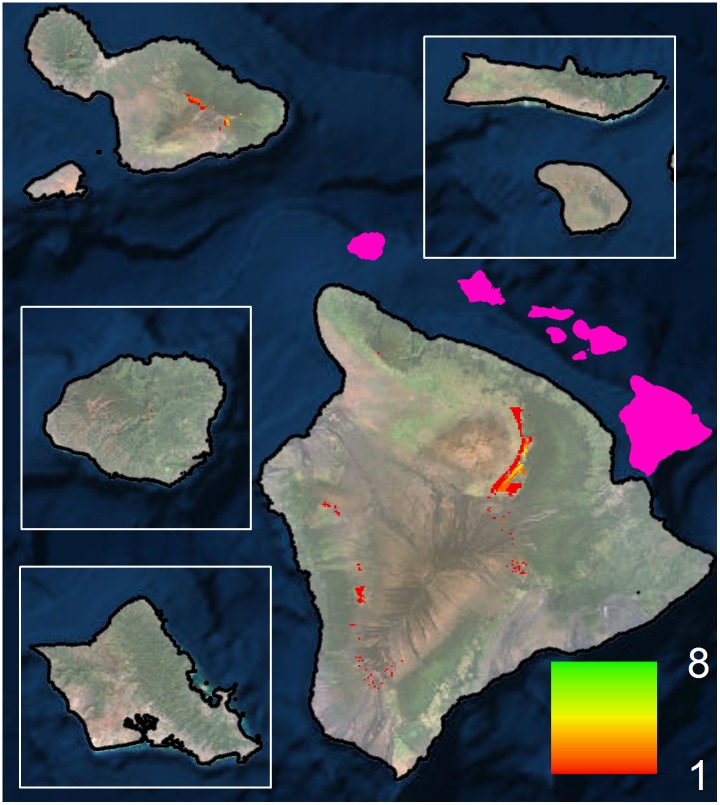
Potential priority areas for forest bird habitat restoration. Map is based on the identification of currently converted forest bird habitat within locations remaining climatically suitable for high model reliability species between now and end of century. The pink overlay shows the spatial configuration of the main Hawaiian Islands. A similar figure including all extant species is included in S7 File.

For species that are already at risk and that our models indicate may lose all available range within the century, actions that reduce climate change impacts on populations while long-term solutions are developed (‘buying time”) are essential and should be implemented as soon as possible. These actions may include aggressive reforestation of former habitats at higher elevations, maintaining captive populations of species that are extremely endangered, and translocating very restricted species to higher elevation habitat outside of their known historic range. We can use our results of future species richness, filtered by areas where primary forest bird habitat has been converted to other land uses (i.e. pasture/grassland), to identify areas where restoration may provide additional habitat for the species (Fig 5). There is a newly initiated research effort that is exploring related options, including the projection of single island species models across the entire archipelago along with niche overlap analyses [86], to identify areas outside known historic range where a species might be successfully translocated. Besides providing additional time for the development of longer-term solutions, these buying-time options would reduce the rate of species exposure to disease and other threats and could provide species additional time to evolve immunity or resistance. However, it should be noted that most buying time options are costly and risky, and thus must be balanced with options from the other two categories of conservation actions described.

**Fig 6:**
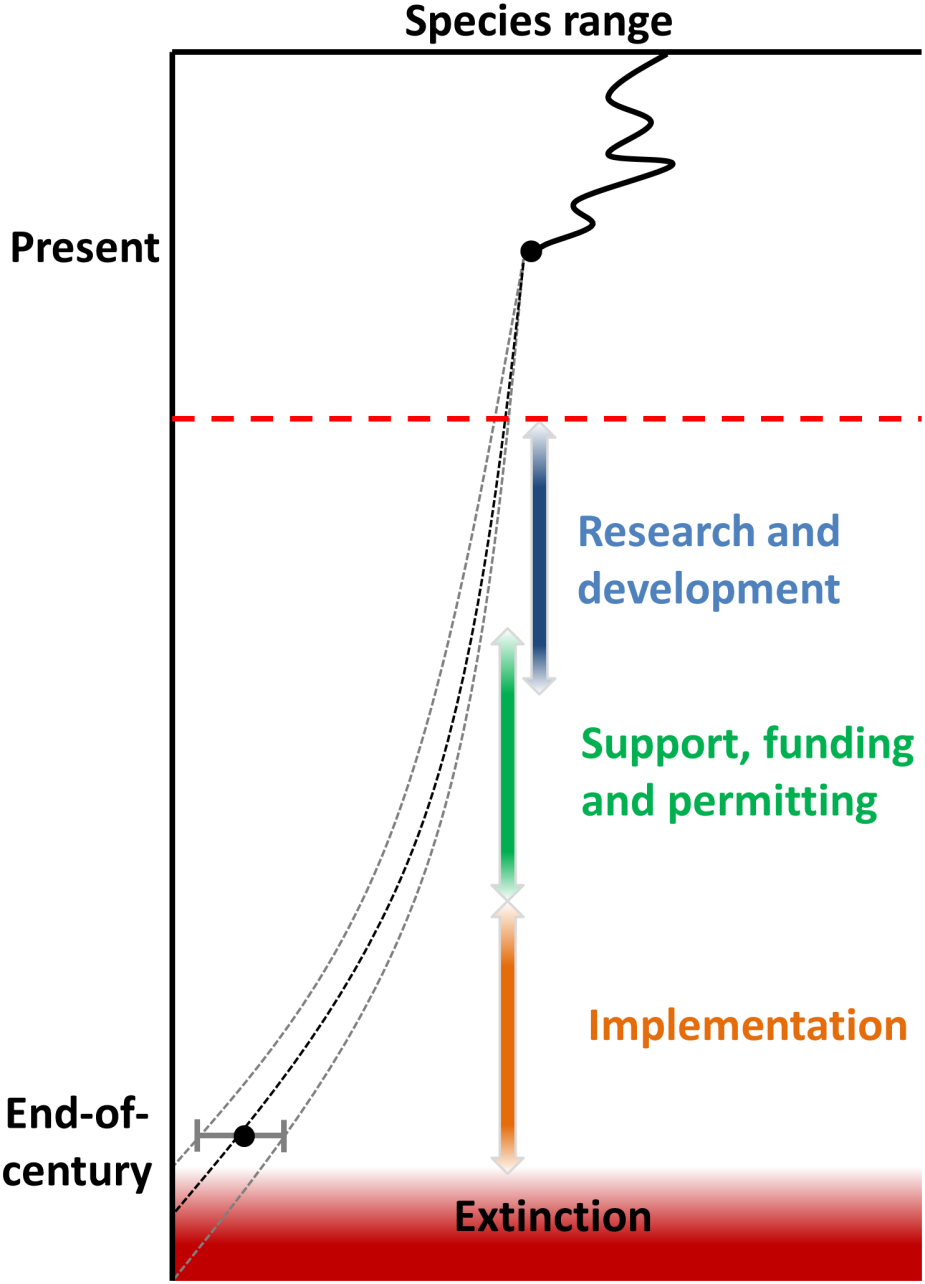
Conceptual timeline for implementing novel forest bird conservation options. Diagram shows the latest possible start (dashed red line) of long-term novel options that decouple climate shifts from species decline. Missing this threshold implies the need of buying time options necessary for species persistence.

Using our end of century SDM projections as reflective of a trajectory of population change, and given the many steps necessary to develop, test and implement any promising novel conservation action (e.g., sterile mosquito release), some species are likely close to a conservation threshold where buying-time actions are required to avoid eventual extinction (Fig 6). Unfortunately, the longer it takes to fully develop novel long-term solutions, the greater the amount of conservation resources that will need to be spent on buying-time actions that ensure the persistence of an increasing number of forest bird species. This conundrum leaves fewer resources available for developing the required long-term solutions. Delay in exploration of long-term solutions therefore brings additional challenges to conservation of at-risk species, as buying time options are unlikely to lead to long-term solutions on their own. This dynamic is especially relevant to Hawaiian conservation where available resources per endangered species is a small fraction of resources available for mainland U.S. species [87]. Without additional funding, a substantial portion of currently available resources may be soon directed towards buying-time actions as current species declines persist. Beyond the necessary research and development of novel options, one way to speed the application of novel conservation actions may be to start tackling the often-overlooked non-technical challenges of conservation of at-risk species, such as ensuring that the proper regulatory frameworks and public support for novel actions are in place well ahead of possible implementation.

## Supporting Information

S1 File Maps of habitat suitability for Hawaiian forest bird species under current and future climate. (PDF)

S2 File ROC and TSS evaluation scores for MaxEnt and GBM individual species models. (PDF)

S3 File Model predictor importance and response curve graphs for all Hawaiian forest bird species models. (PDF)

S4 File Agreement between expert-derived and modeled current climate-based ranges for all Hawaiian forest bird species. (PDF)

S5 File Table of forest bird primary habitat associations, figures of proportional presence of species along climate gradients, and map of 2100 analogous climate areas. (PDF)

S6 File Maps of climate-based species range shifts within primary habitat. (PDF)

S7 File Combined species maps showing changes in forest bird diversity considering all extant species and high model reliability species. (PDF)

S8 File Table with projected changes between 1990–2010 and 2080–2100 in range for all Hawaiian forest bird species limited to current available primary habitat. (PDF)
