# Correction: Large-Scale Range Collapse of Hawaiian Forest Birds under Climate Change and the Need 21^st^ Century Conservation Options

**DOI:** 10.1371/journal.pone.0144311

**Published:** 2015-11-30

**Authors:** The PLOS ONE Staff

The publisher erroneously omitted the word “for” from the article title. The correct title is: Large-Scale Range Collapse of Hawaiian Forest Birds under Climate Change and the Need for 21^st^ Century Conservation Options. The correct citation is: Fortini LB, Vorsino AE, Amidon FA, Paxton EH, Jacobi JD (2015) Large-Scale Range Collapse of Hawaiian Forest Birds under Climate Change and the Need for 21^st^ Century Conservation Options. PLoS ONE 10(10): e0140389. 10.1371/journal.pone.0140389. The publisher apologizes for the error.
