# Supplementary material for: Large-Scale Range Collapse of Hawaiian Forest Birds under Climate Change and the Need 21st Century Conservation Options
Source: PLoS One. 2015 Oct 28;10(10):e0140389. doi: 10.1371/journal.pone.0140389 (PMC4625087; doi:10.1371/journal.pone.0140389)

Appendix S1. Maps of habitat suitability for Hawaiian forest bird species under current and future climate.

Ensemble model suitability scores for each individual species separated between high and reduced model reliability species.

Suitability scores range from 0 to 1 and are based on a mean across models, weighted by ROC scores.

S1
HIGH MODEL RELIABILITY SPECIES

Akekee baseline modeled suitability

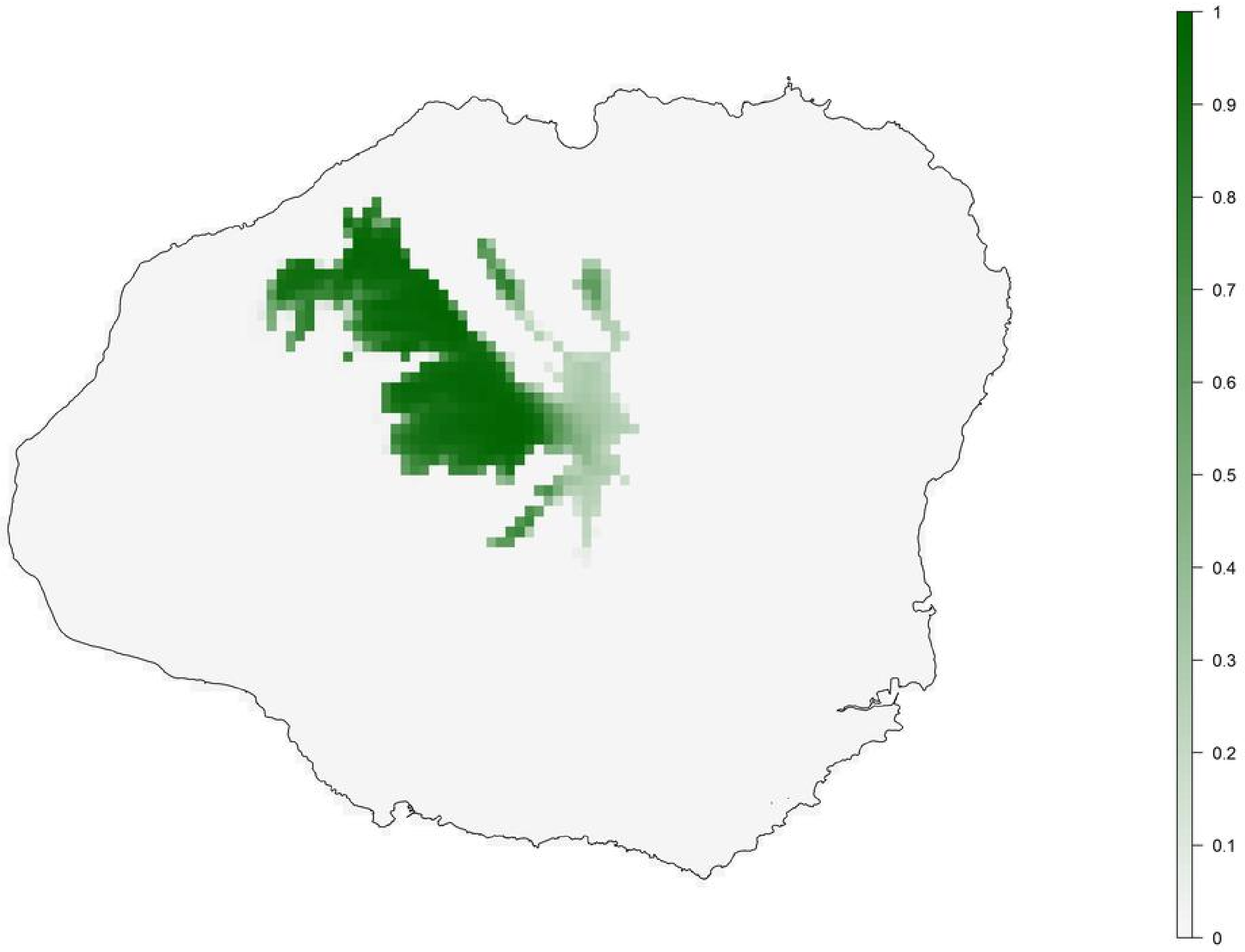

S1

HIGH MODEL RELIABILITY SPECIES

Akekee future modeled suitability

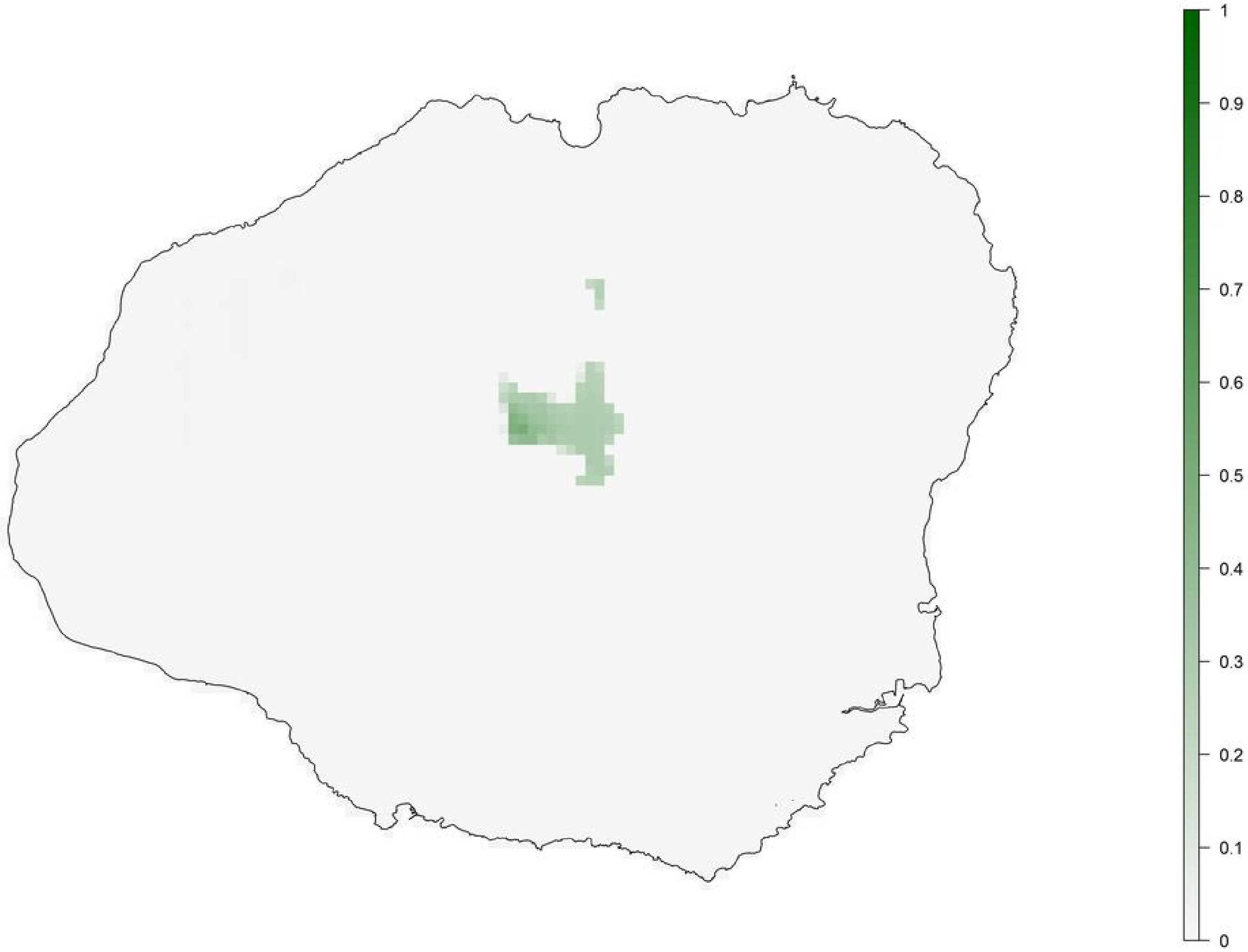

S1

HIGH MODEL RELIABILITY SPECIES

Akiapolauu baseline modeled suitability

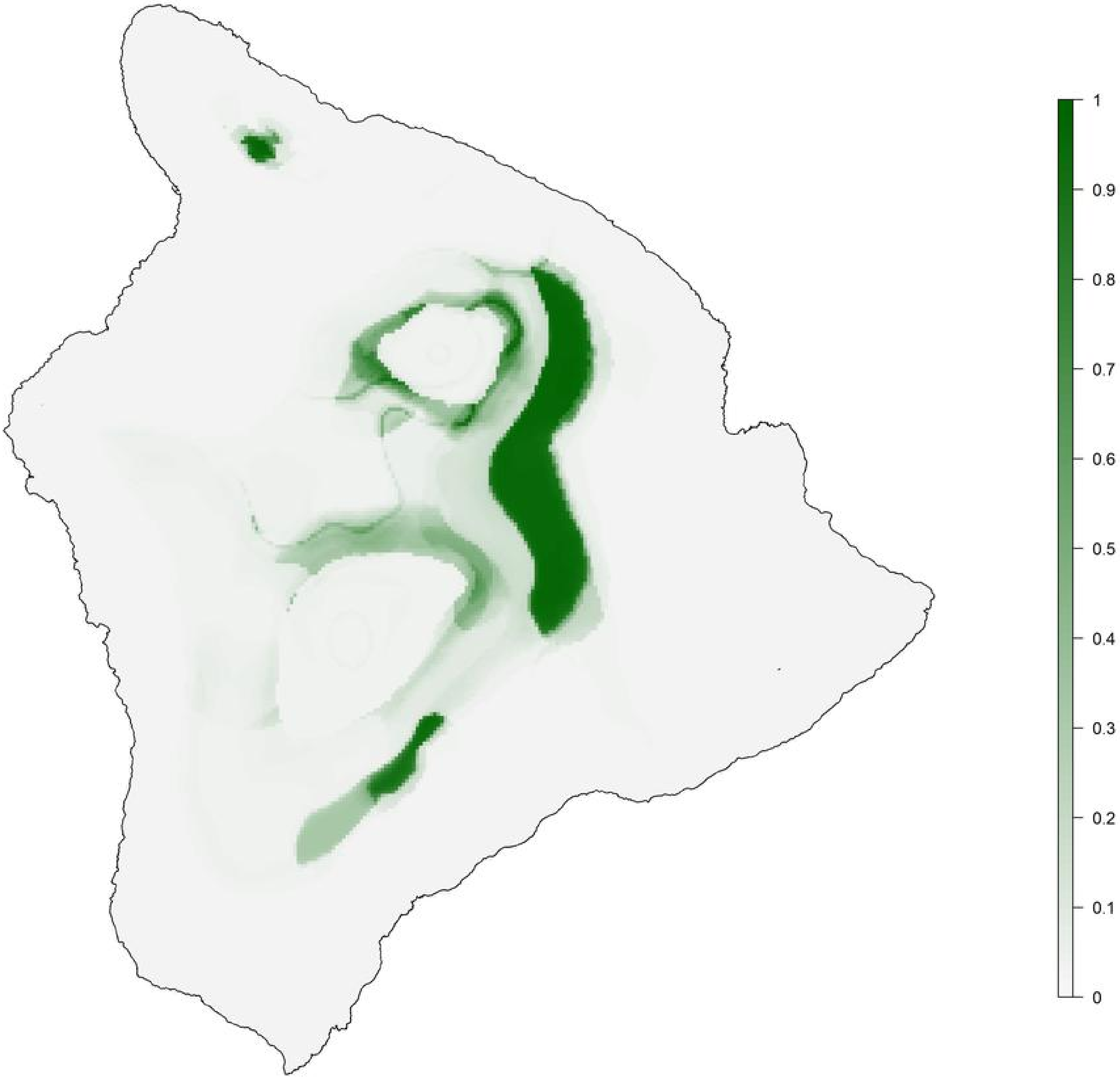

S1
HIGH MODEL RELIABILITY SPECIES

Akiapolauu future modeled suitability

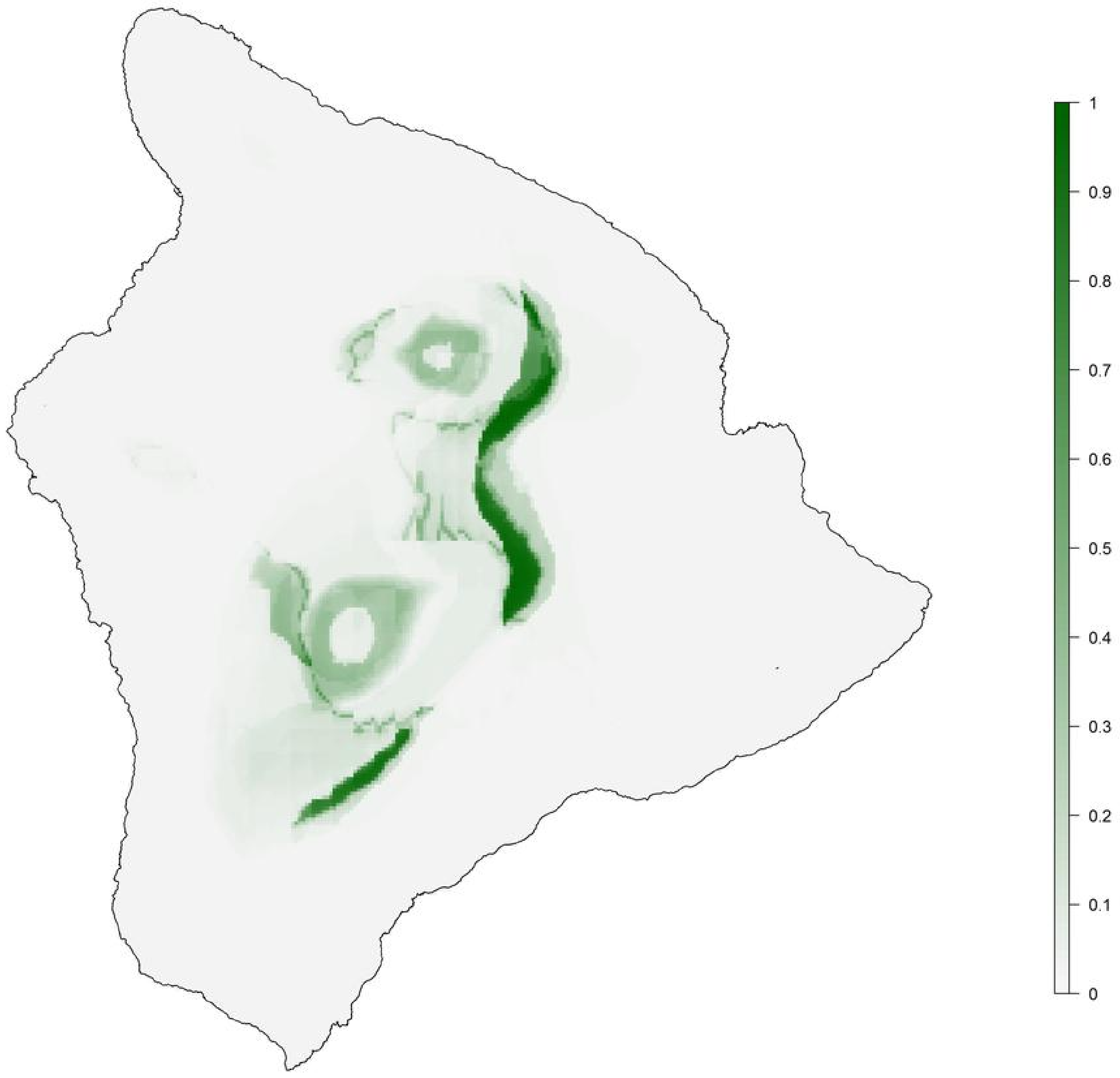

S1

HIGH MODEL RELIABILITY SPECIES

Akikiki baseline modeled suitability

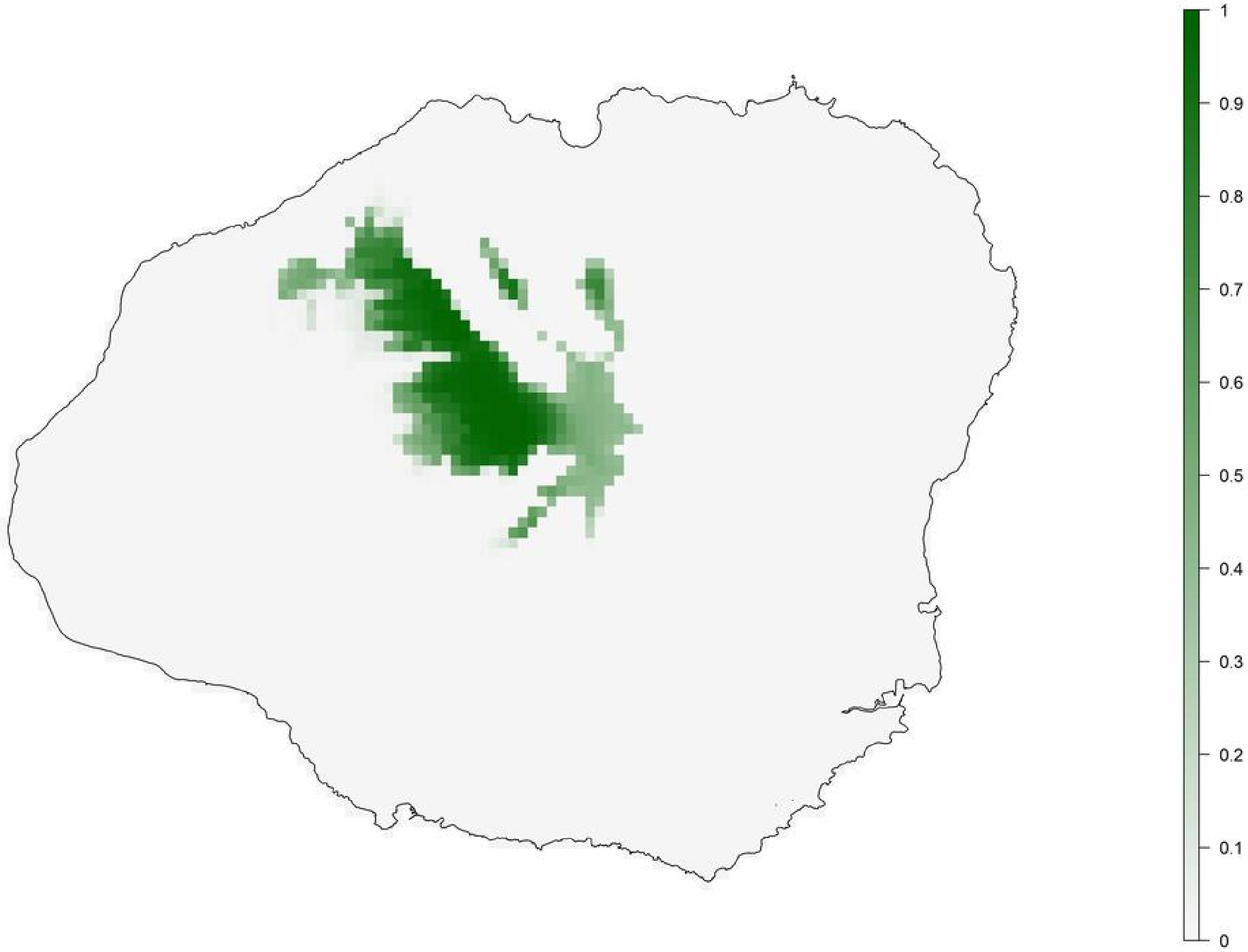

S1
HIGH MODEL RELIABILITY SPECIES

Akikiki future modeled suitability

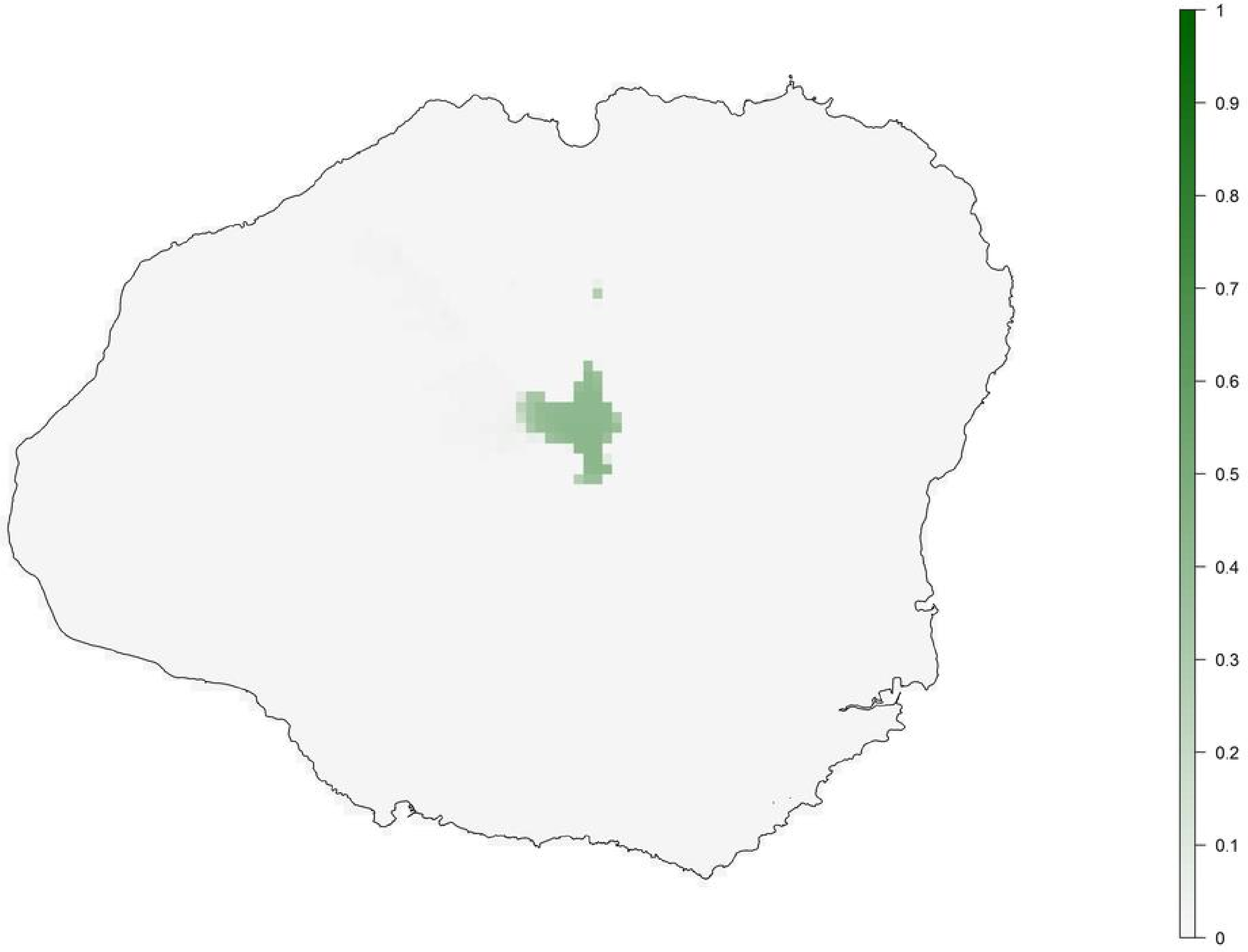

S1

HIGH MODEL RELIABILITY SPECIES

Akohekohe baseline modeled suitability

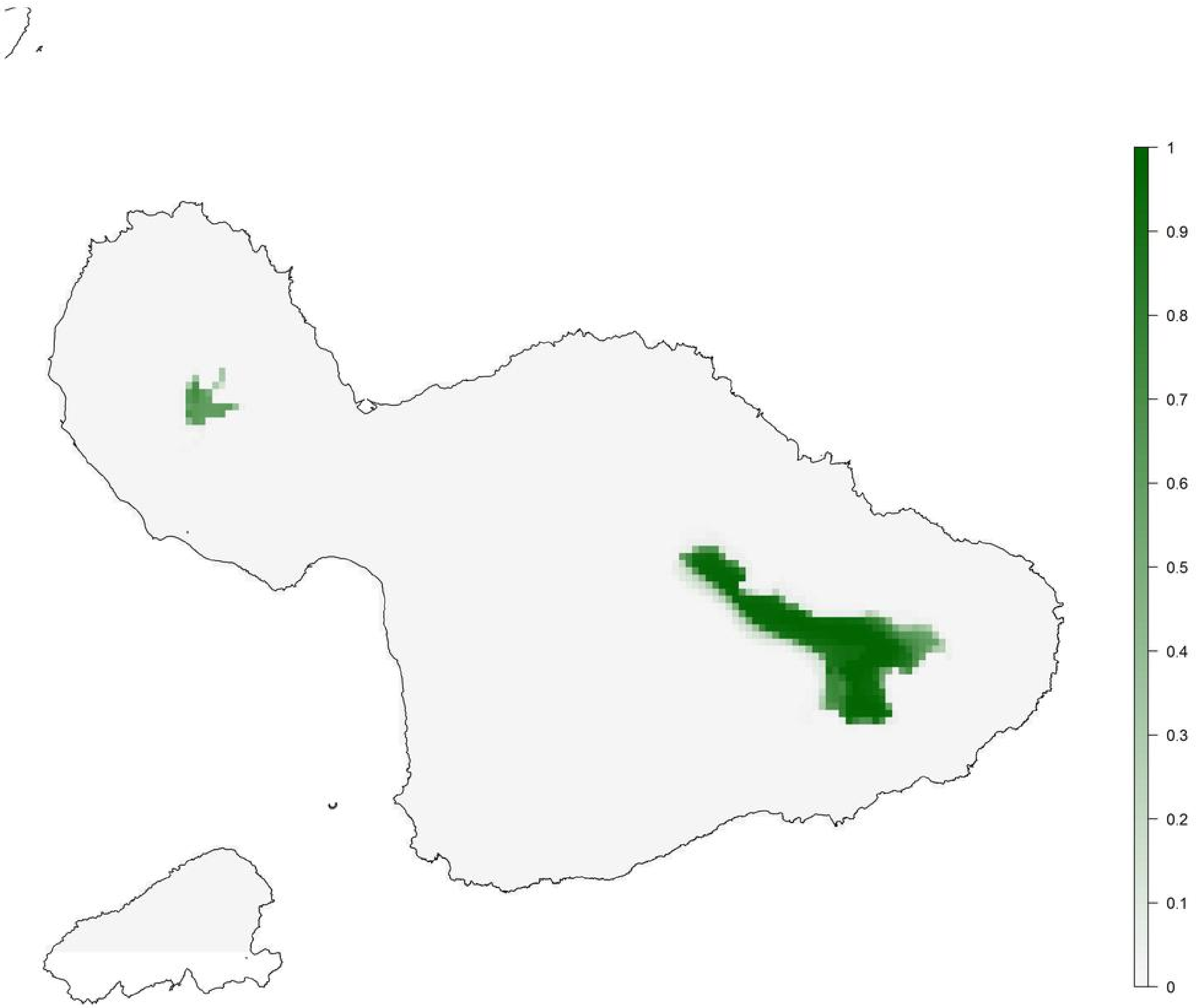

S1
HIGH MODEL RELIABILITY SPECIES

Akohekohe future modeled suitability

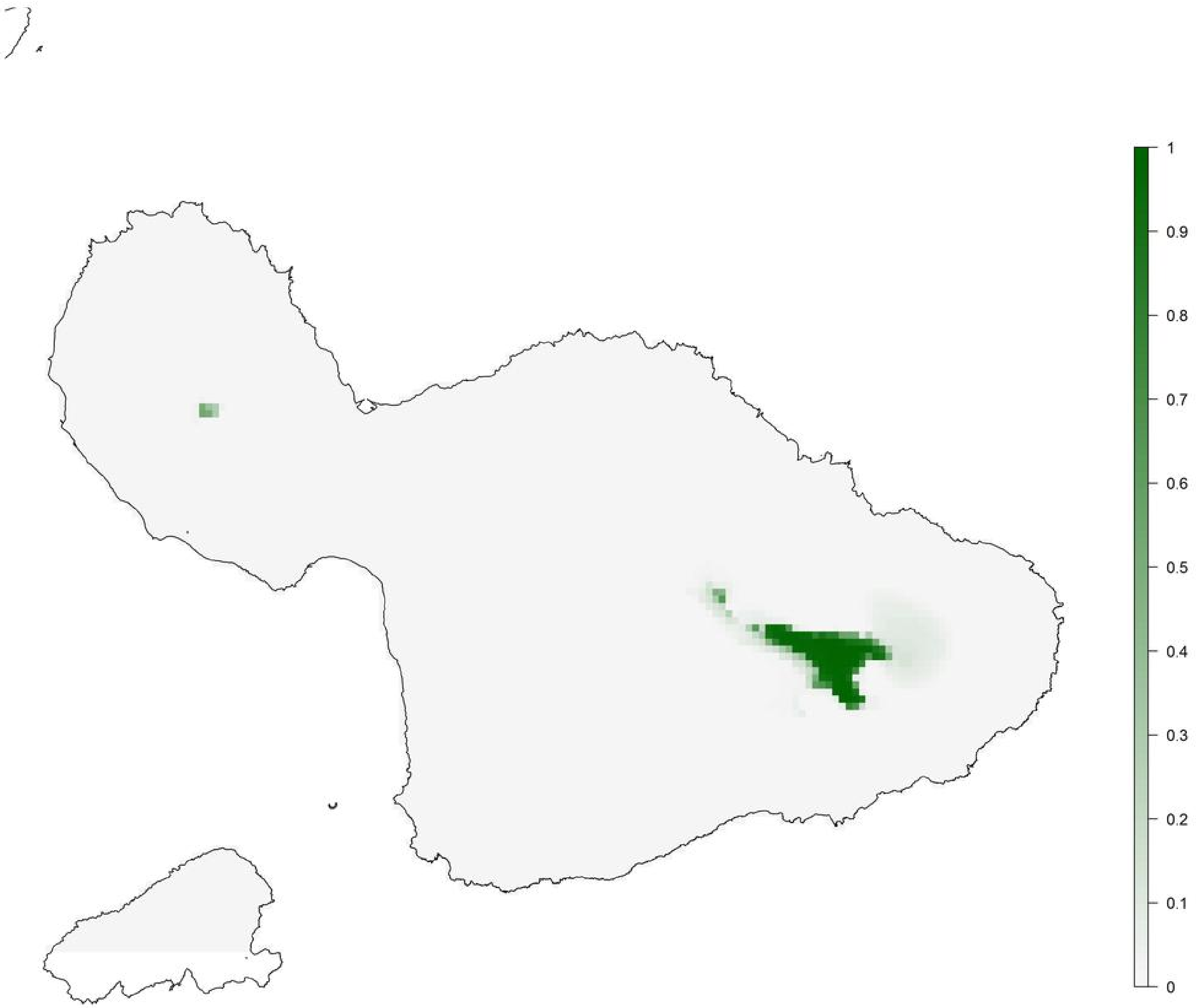

S1
HIGH MODEL RELIABILITY SPECIES

Hawaii Akepa baseline modeled suitability

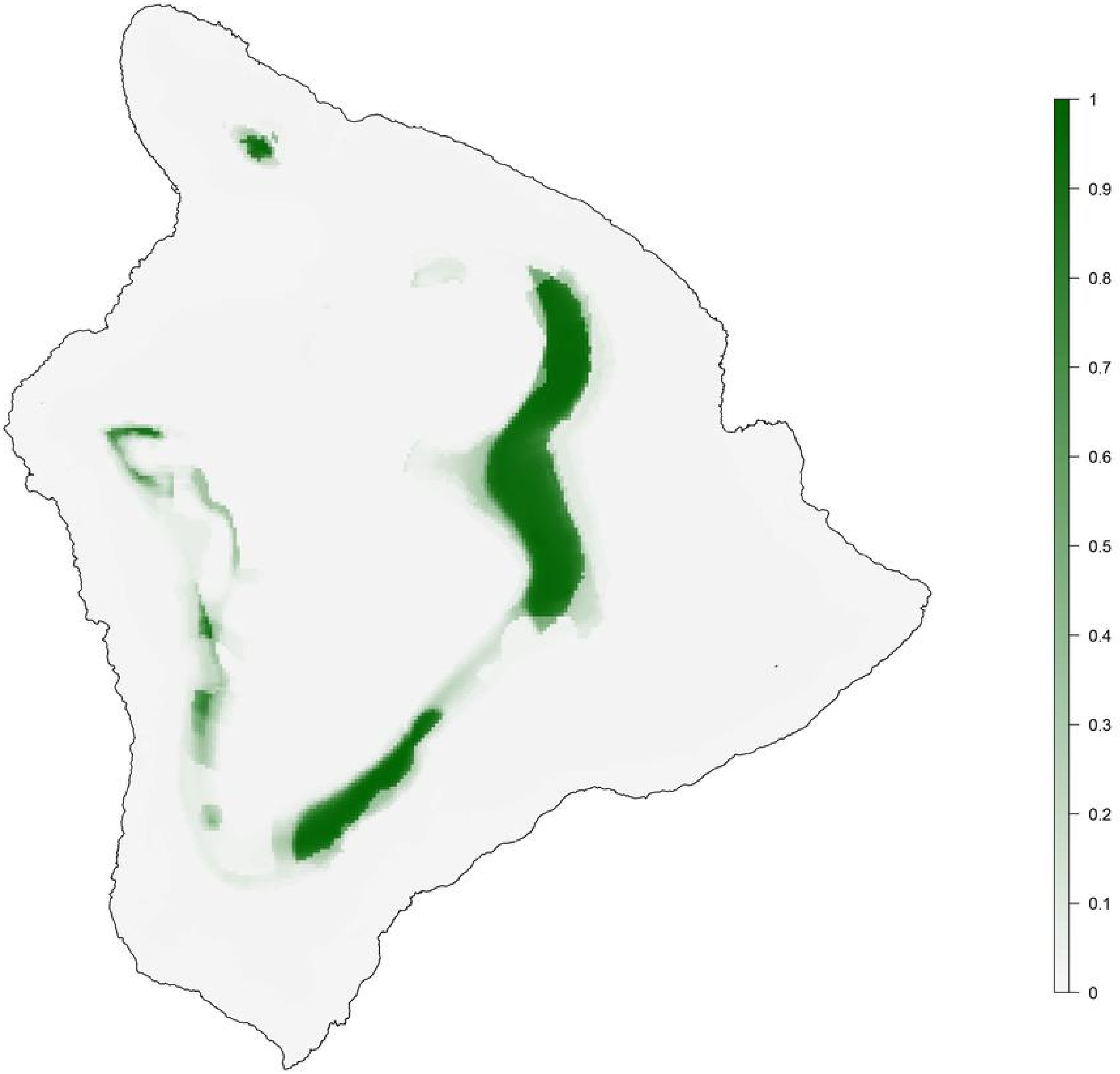

S1
HIGH MODEL RELIABILITY SPECIES

Hawaii Akepa future modeled suitability

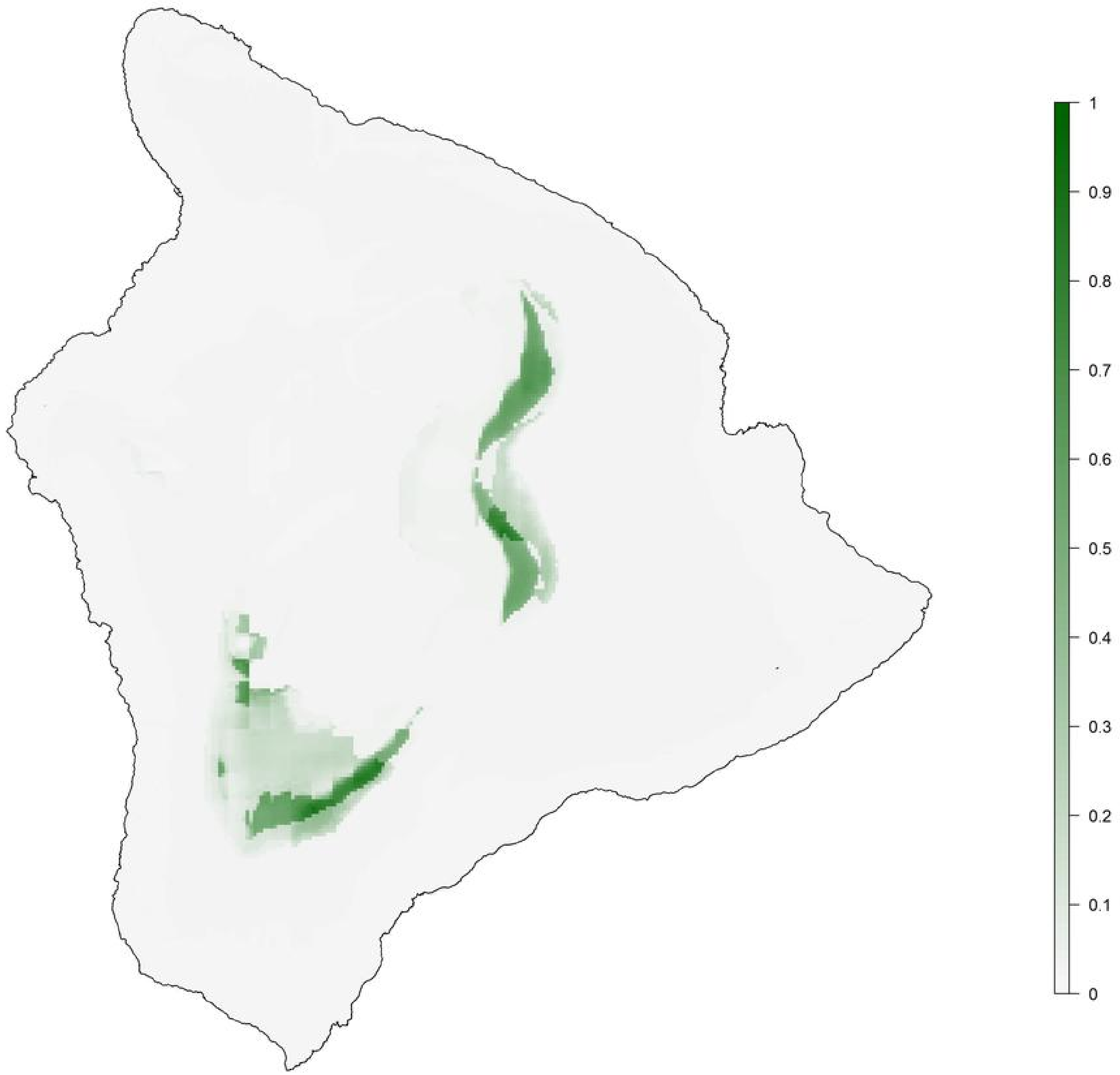

S1

HIGH MODEL RELIABILITY SPECIES

Hawaii Creeper baseline modeled suitability

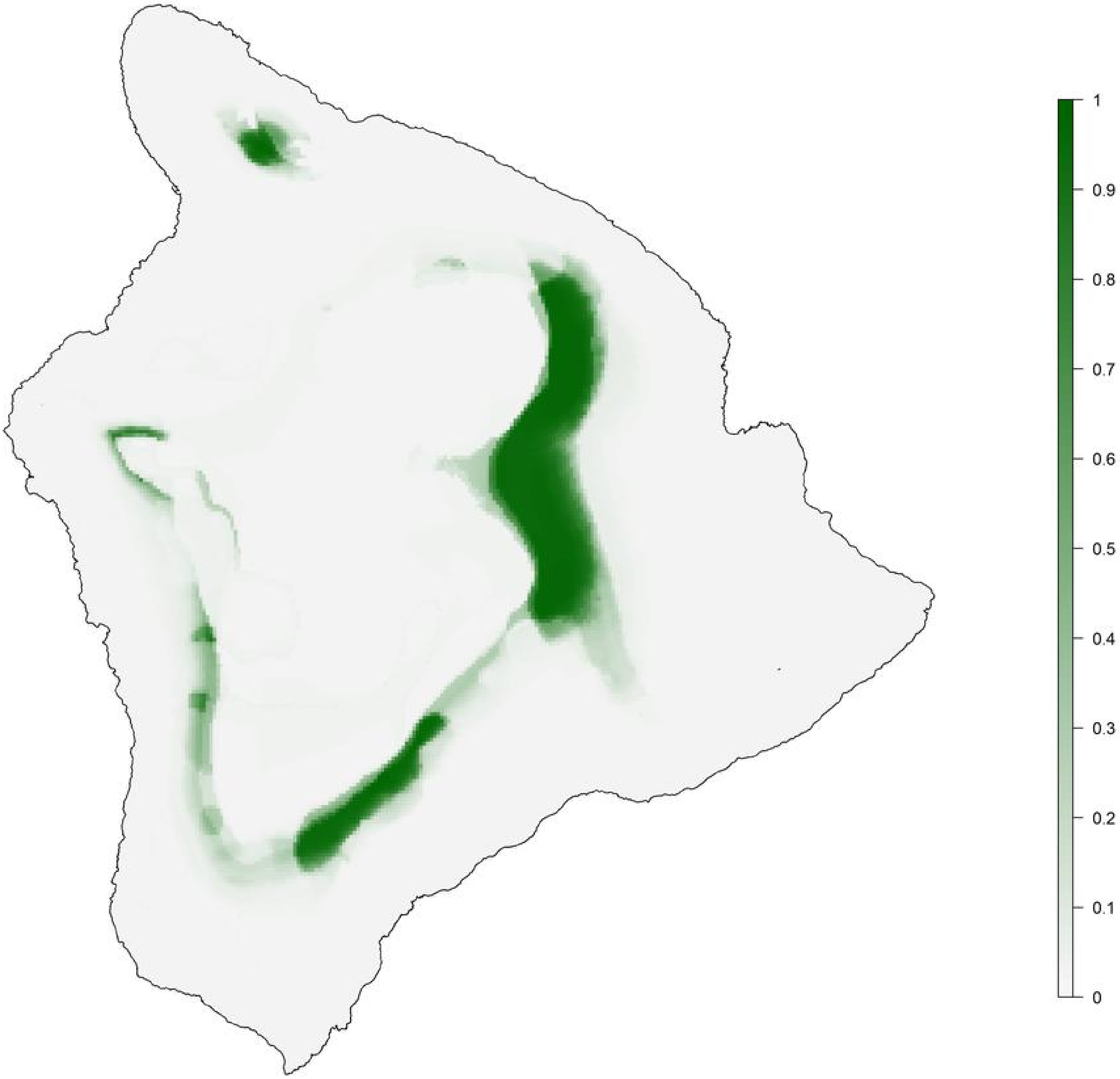

S1
HIGH MODEL RELIABILITY SPECIES

Hawaii Creeper future modeled suitability

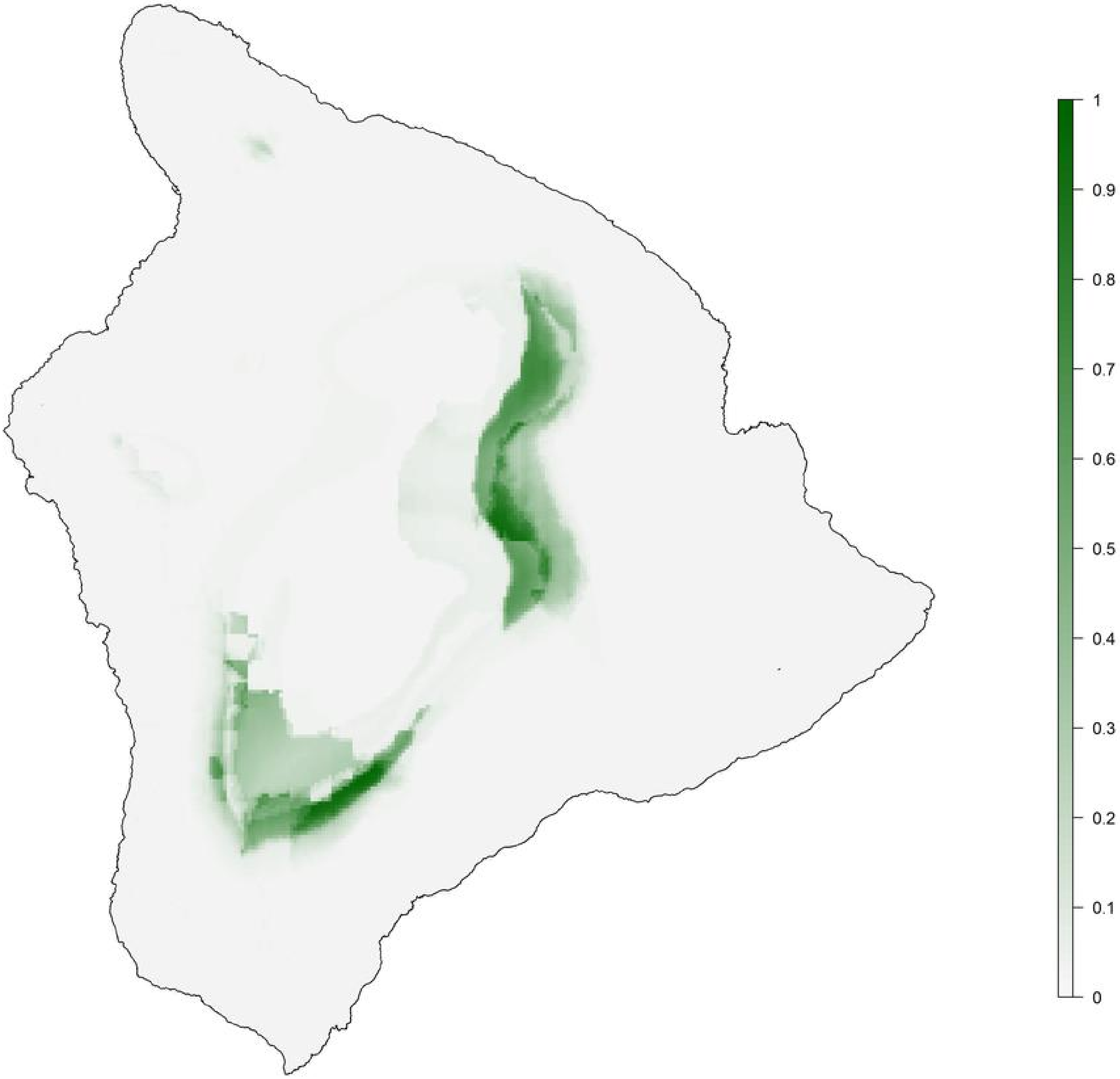

S1

HIGH MODEL RELIABILITY SPECIES

Iiwi baseline modeled suitability

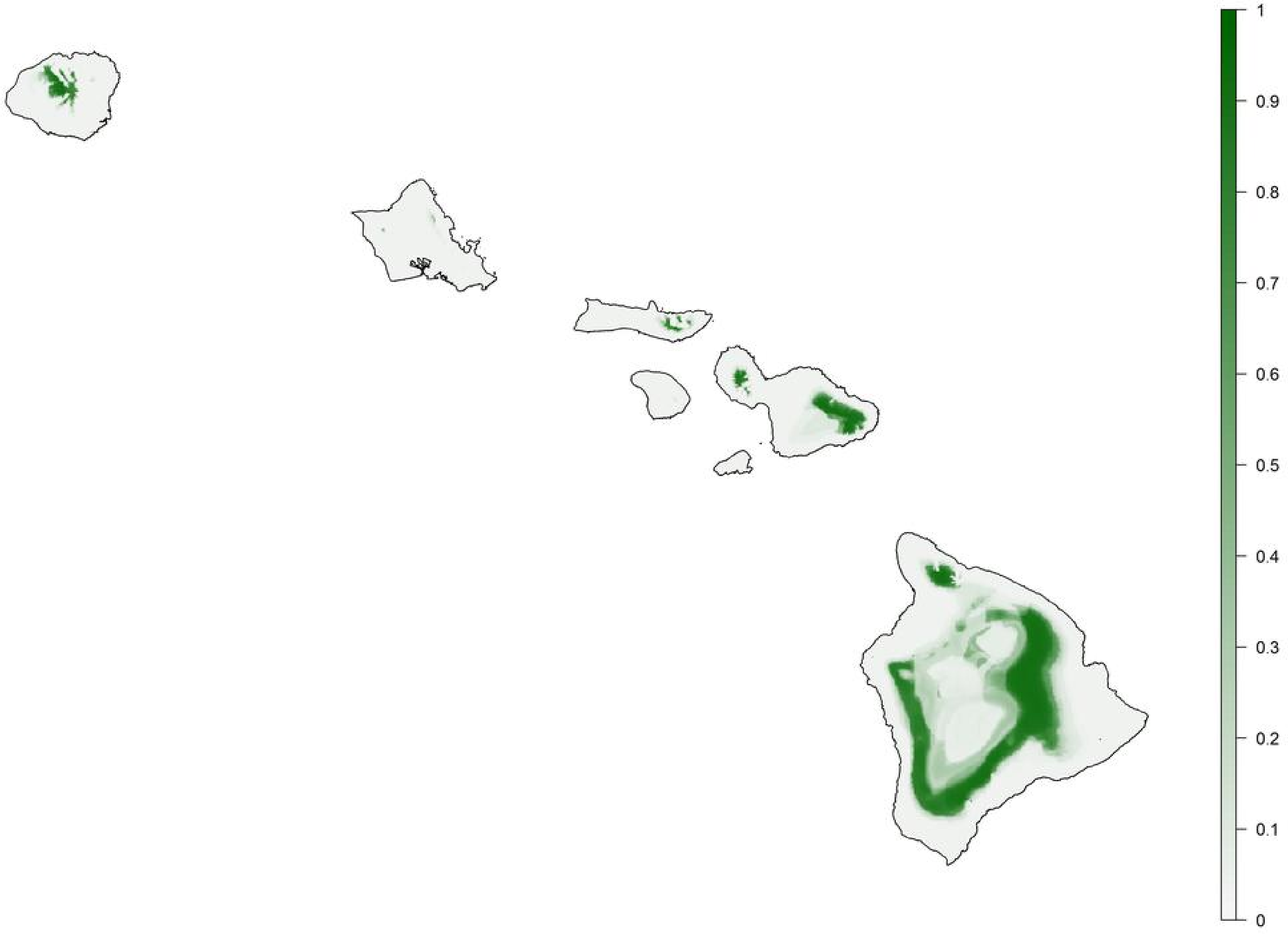

S1
HIGH MODEL RELIABILITY SPECIES

Iiwi future modeled suitability

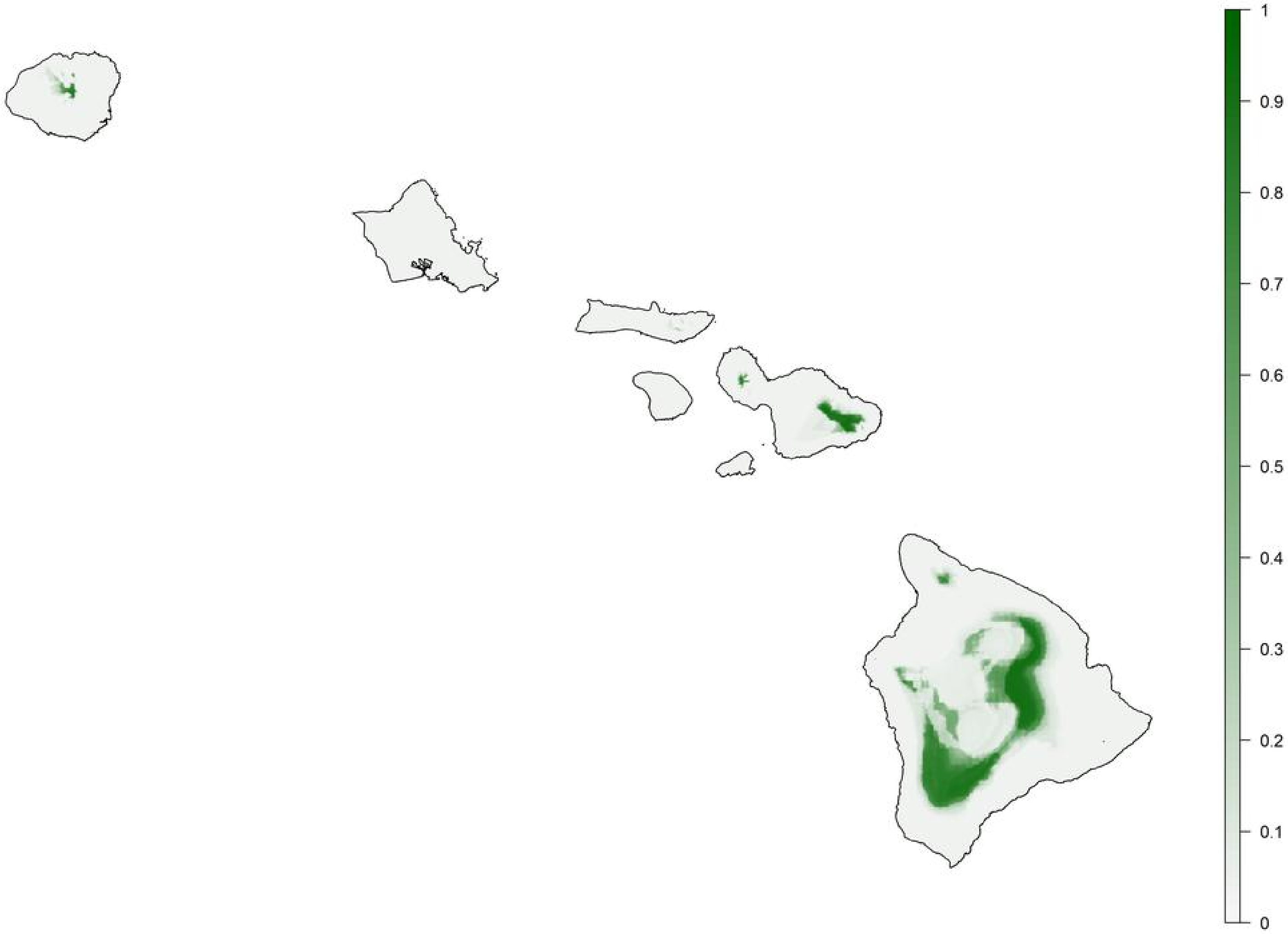

S1
HIGH MODEL RELIABILITY SPECIES

Maui Alauahio baseline modeled suitability

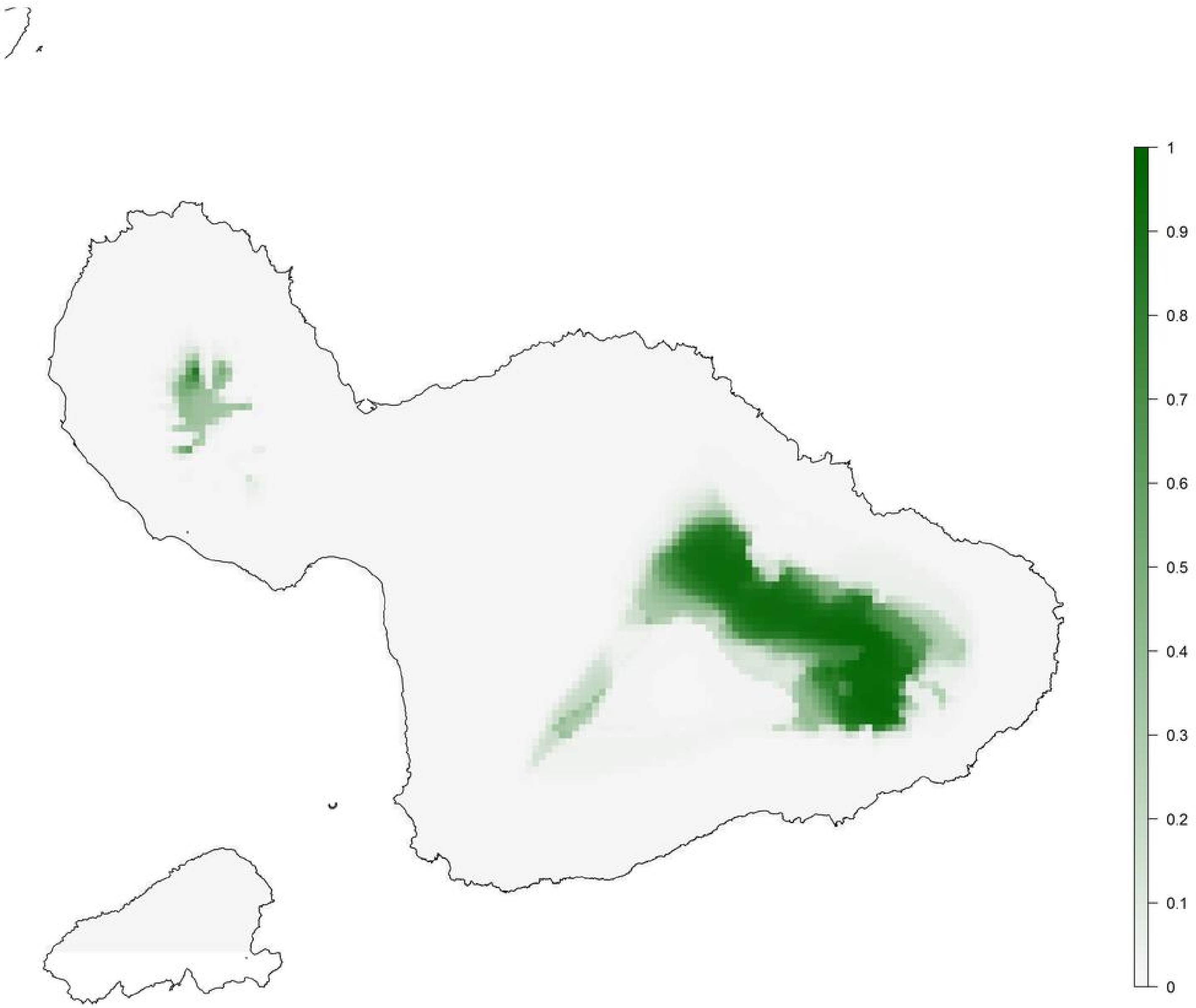

S1

HIGH MODEL RELIABILITY SPECIES

Maui Alauahio future modeled suitability

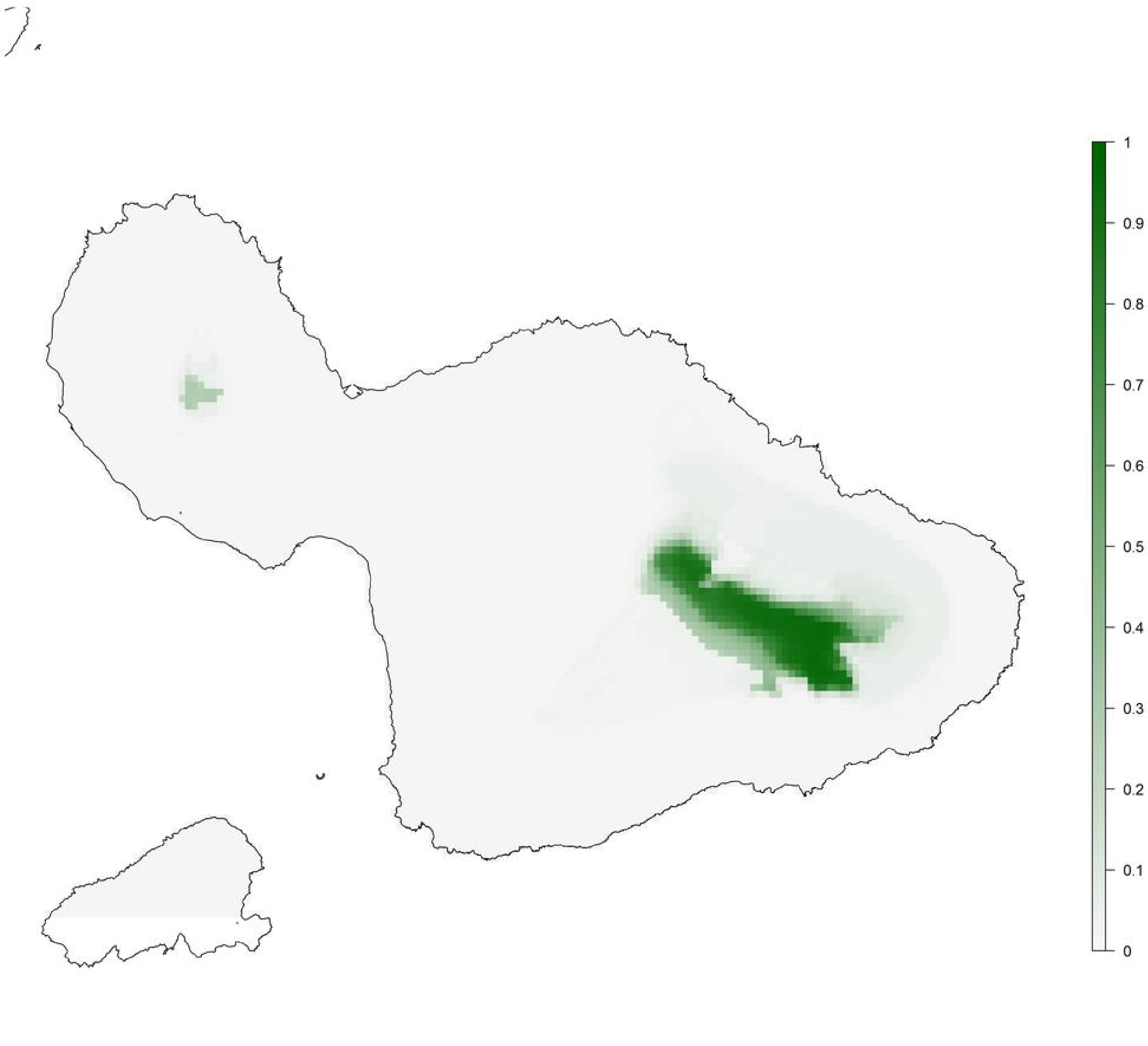

S1

HIGH MODEL RELIABILITY SPECIES

Maui Parrotbill baseline modeled suitability

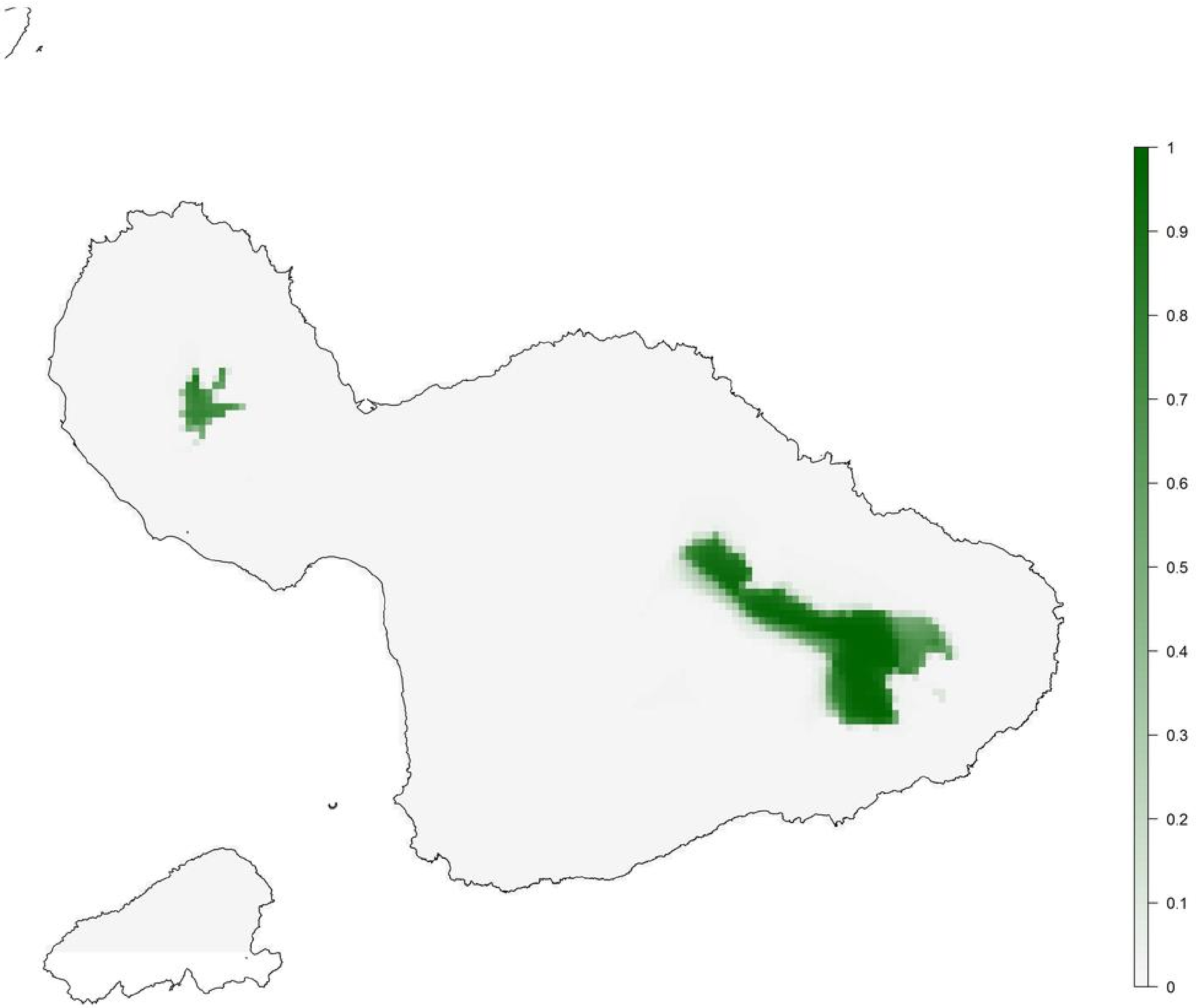

S1
HIGH MODEL RELIABILITY SPECIES

Maui Parrotbill future modeled suitability

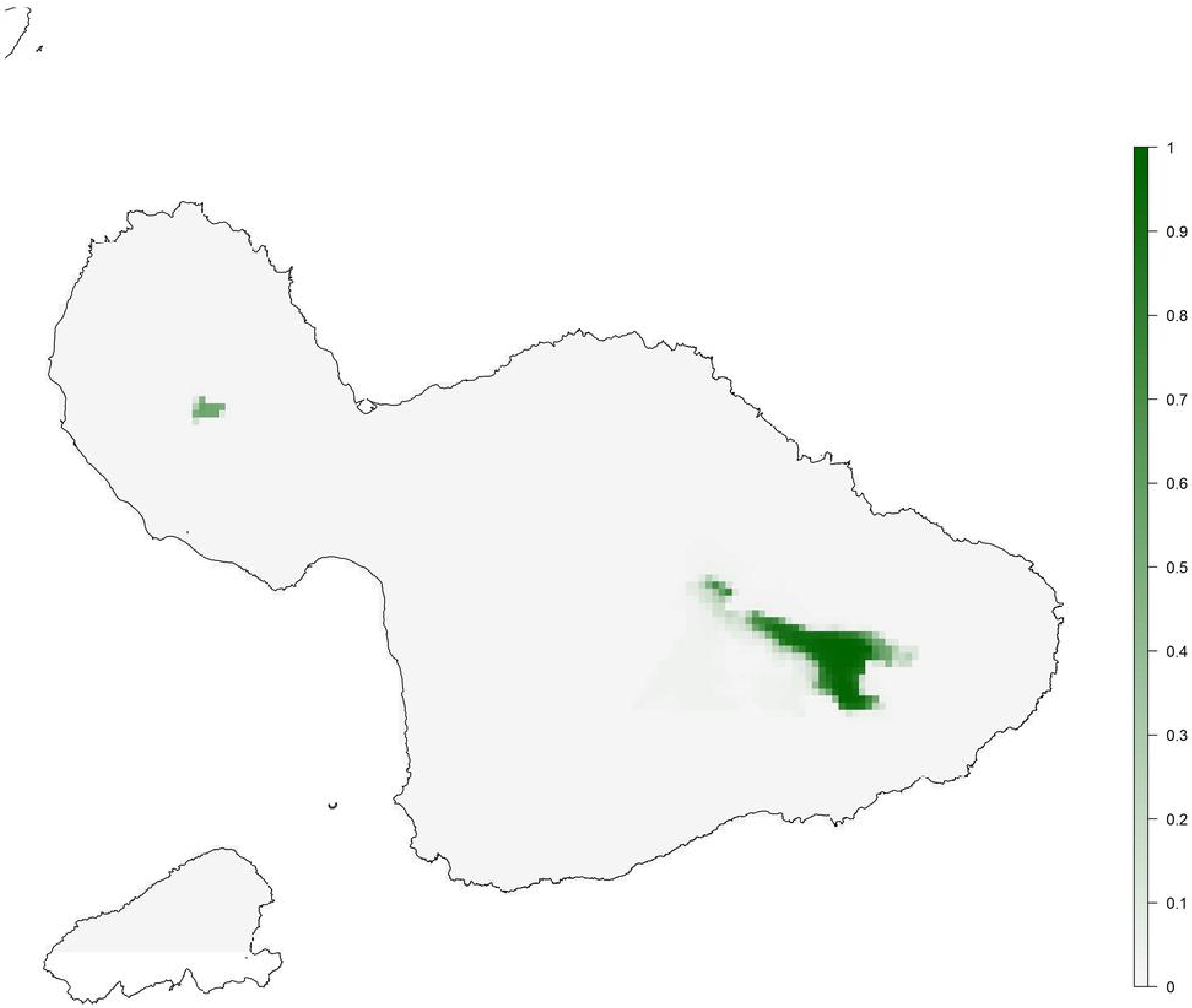

S1

HIGH MODEL RELIABILITY SPECIES

Puaiohi baseline modeled suitability

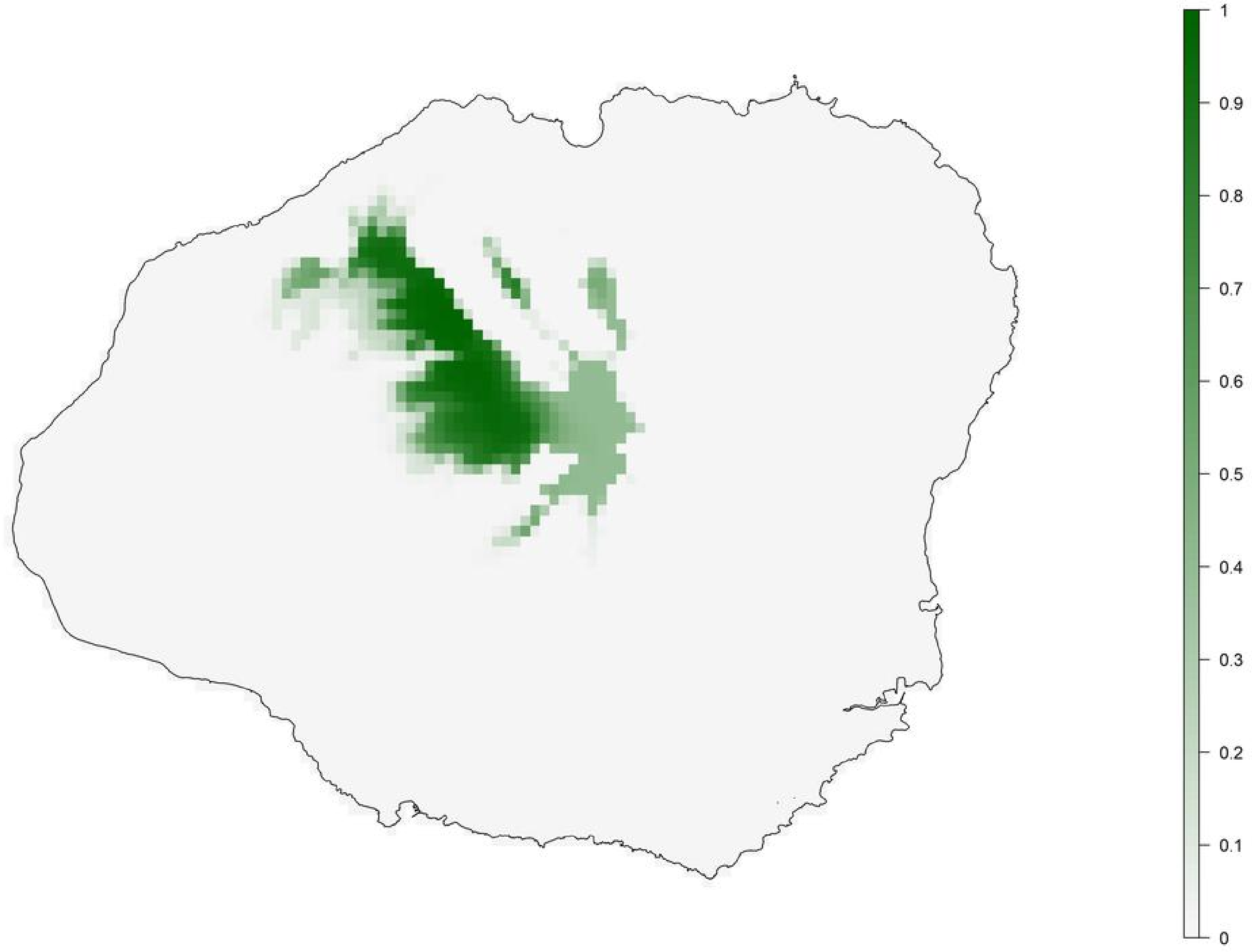

S1
HIGH MODEL RELIABILITY SPECIES

Puaiohi future modeled suitability

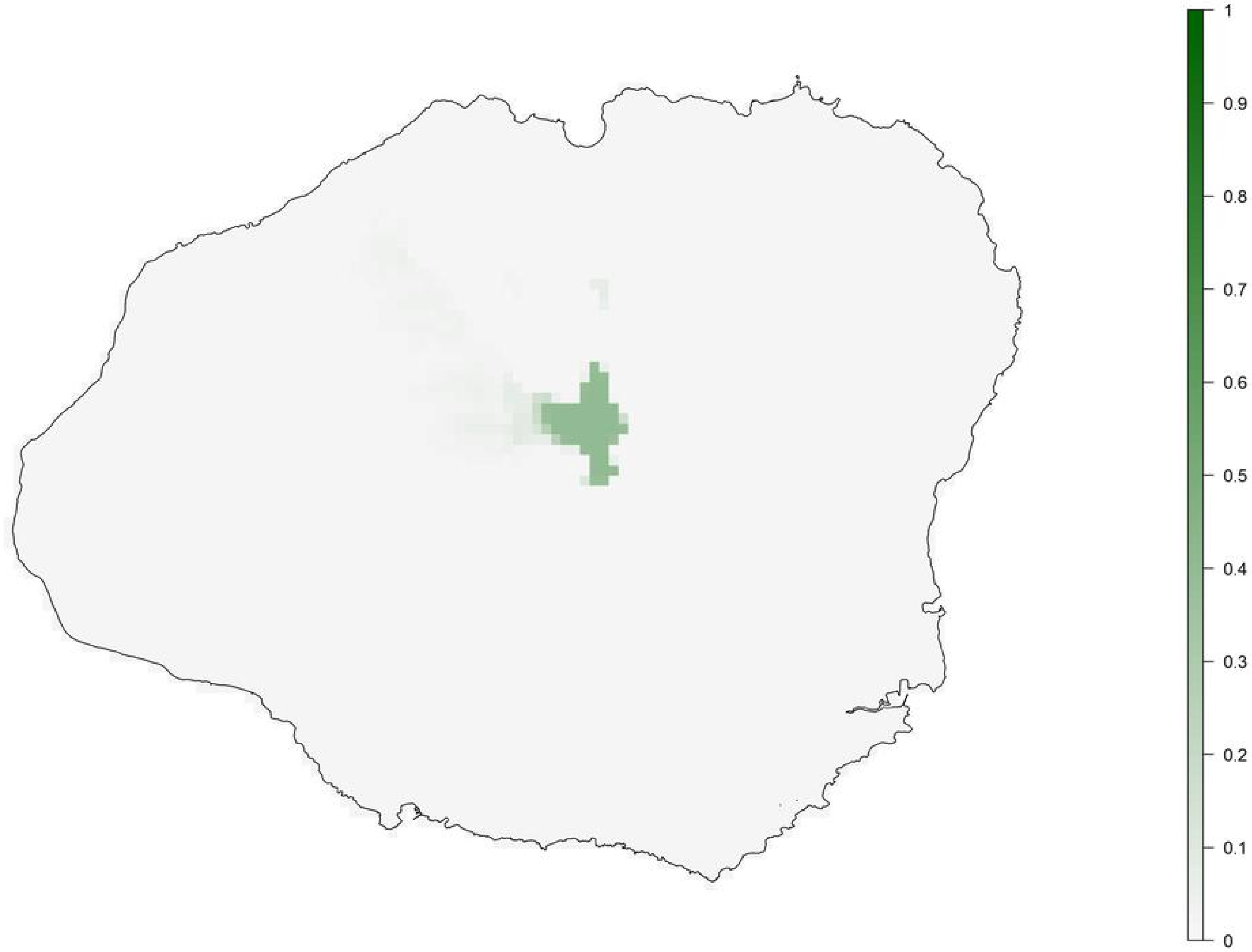

S1
REDUCED MODEL RELIABILITY SPECIES

Anianiau baseline modeled suitability

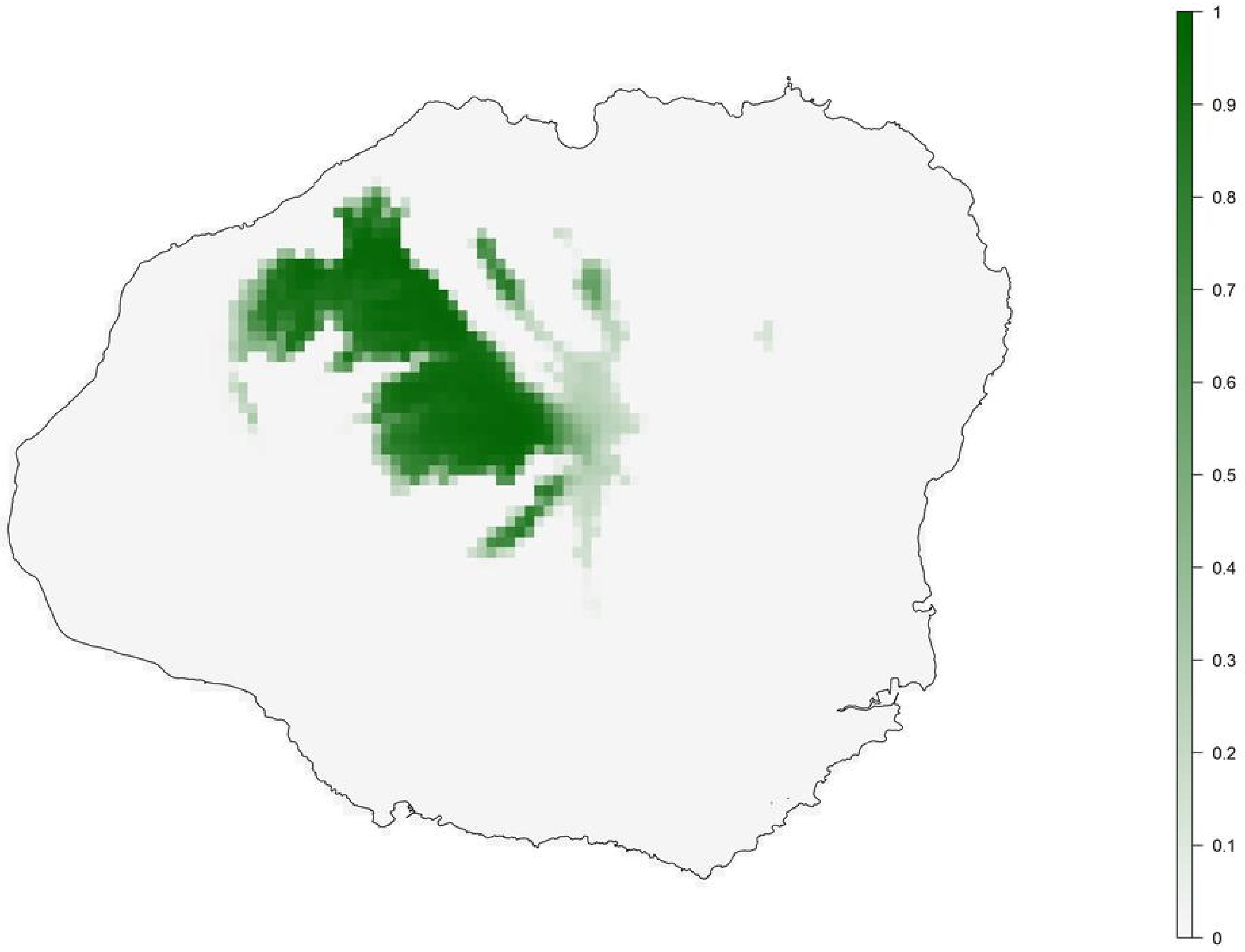

S1

REDUCED MODEL RELIABILITY SPECIES

Anianiau future modeled suitability

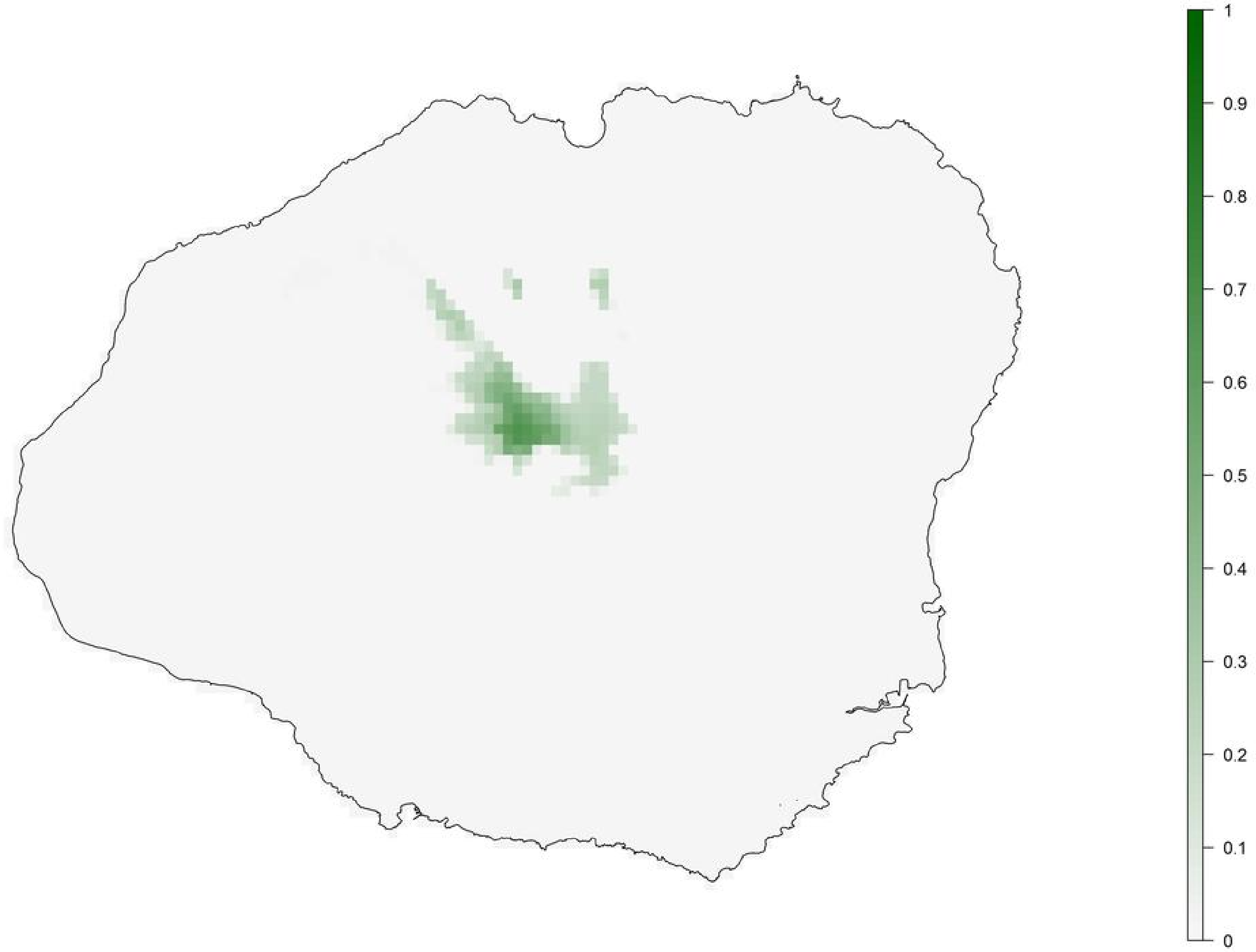

S1
REDUCED MODEL RELIABILITY SPECIES

Apapane baseline modeled suitability

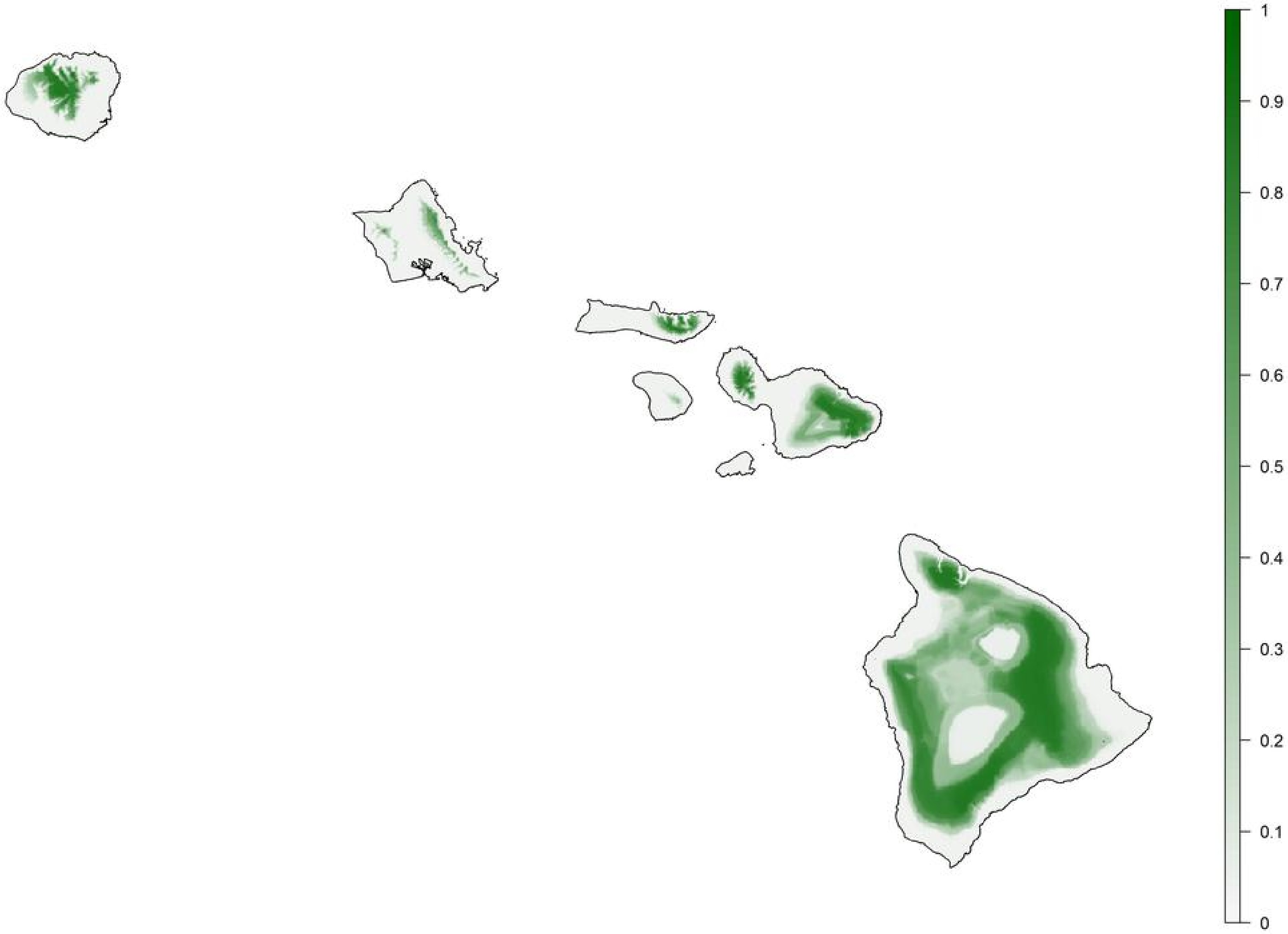

S1

REDUCED MODEL RELIABILITY SPECIES

Apapane future modeled suitability

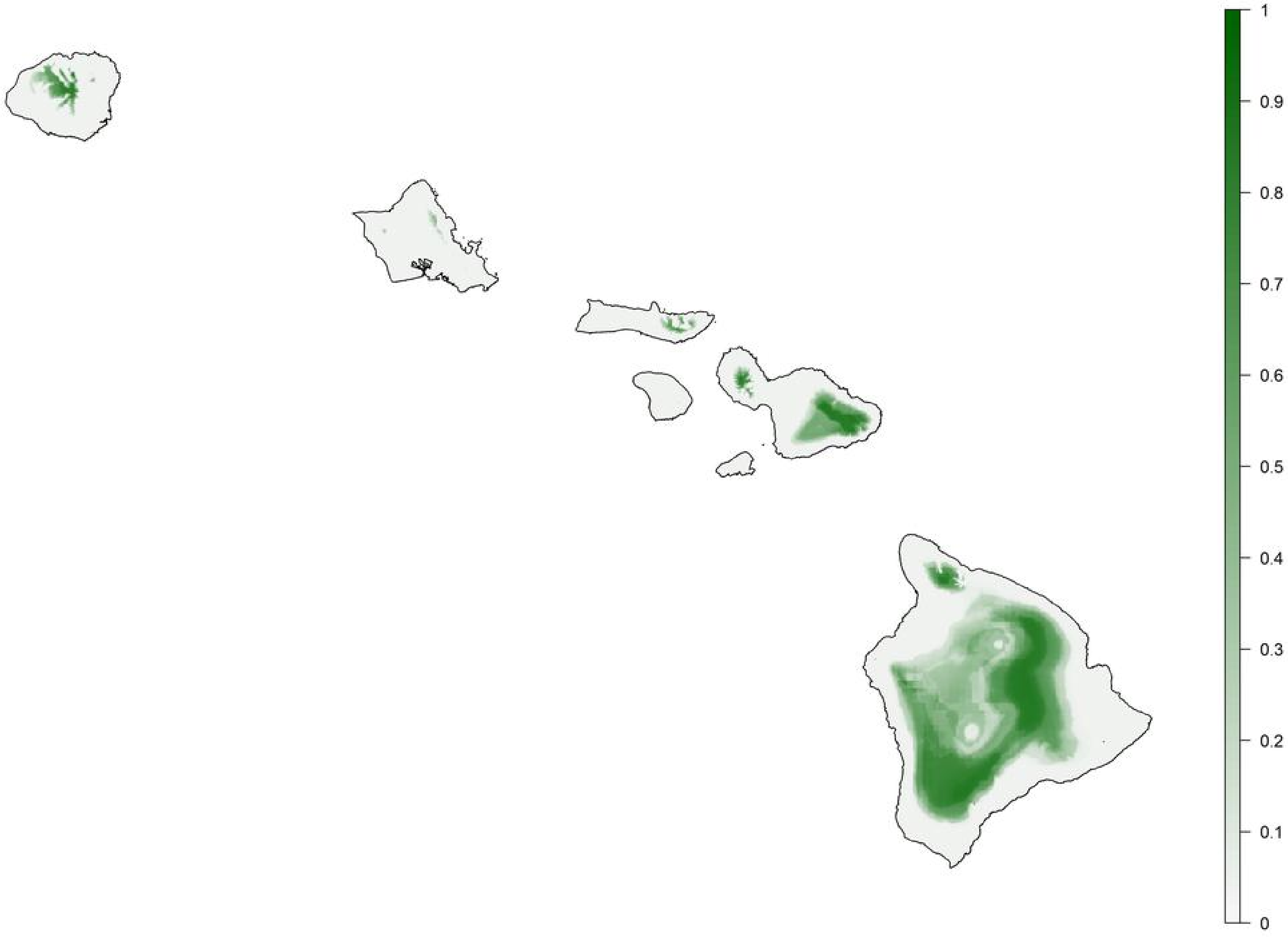

S1
REDUCED MODEL RELIABILITY SPECIES

Hawaii Amakihi baseline modeled suitability

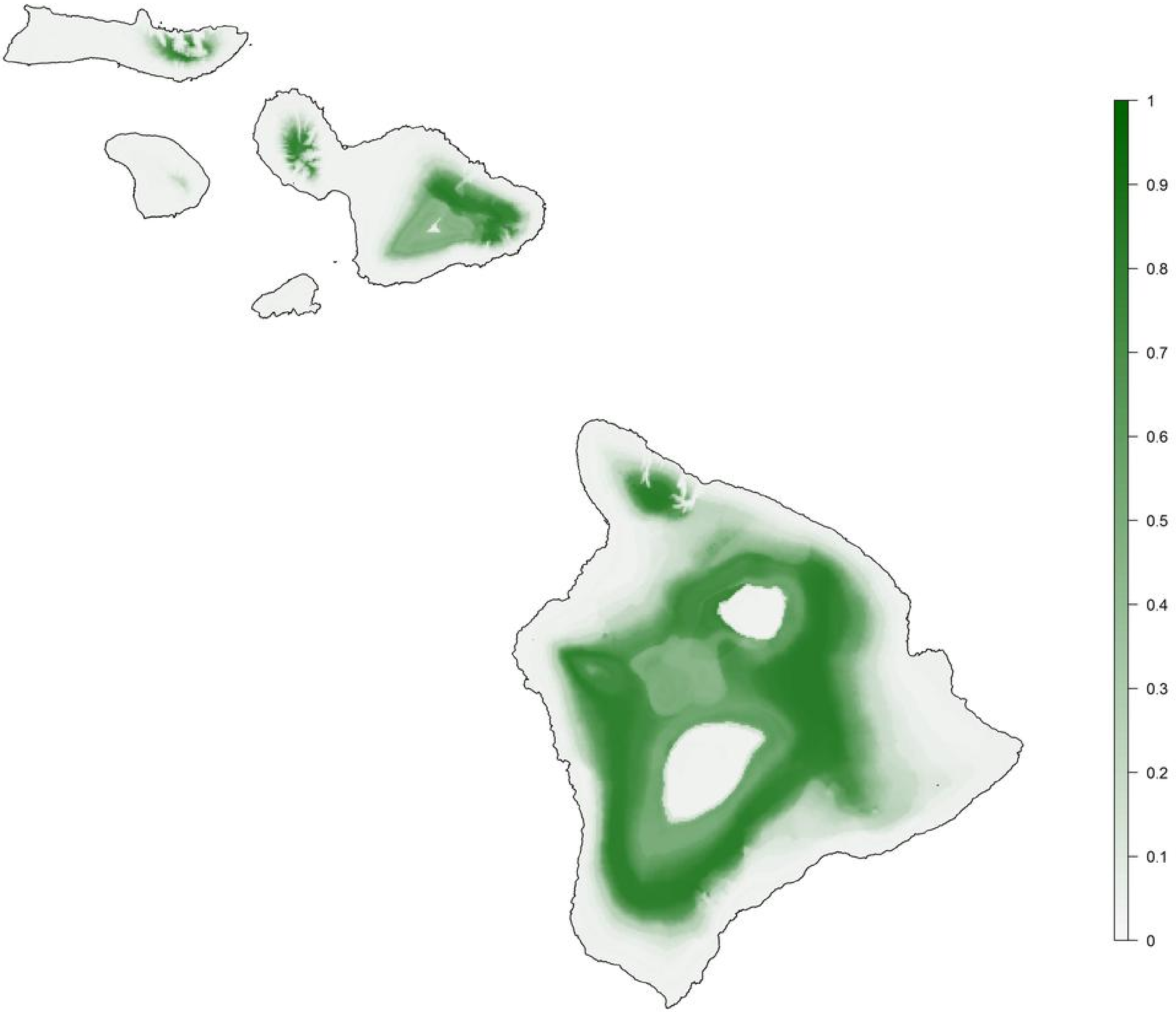

S1
REDUCED MODEL RELIABILITY SPECIES

Hawaii Amakihi future modeled suitability

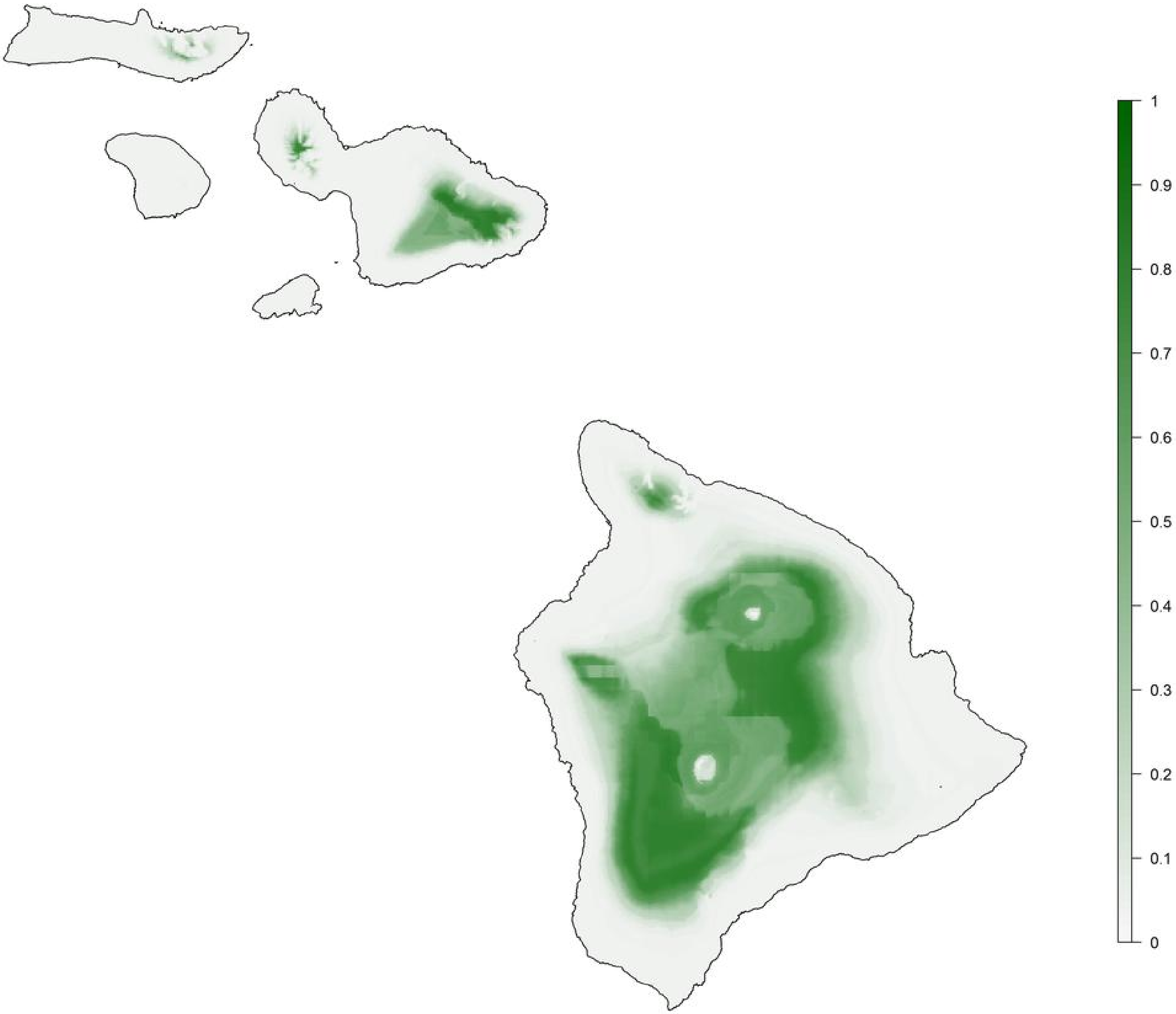

S1
REDUCED MODEL RELIABILITY SPECIES

Hawaii Elepaio baseline modeled suitability

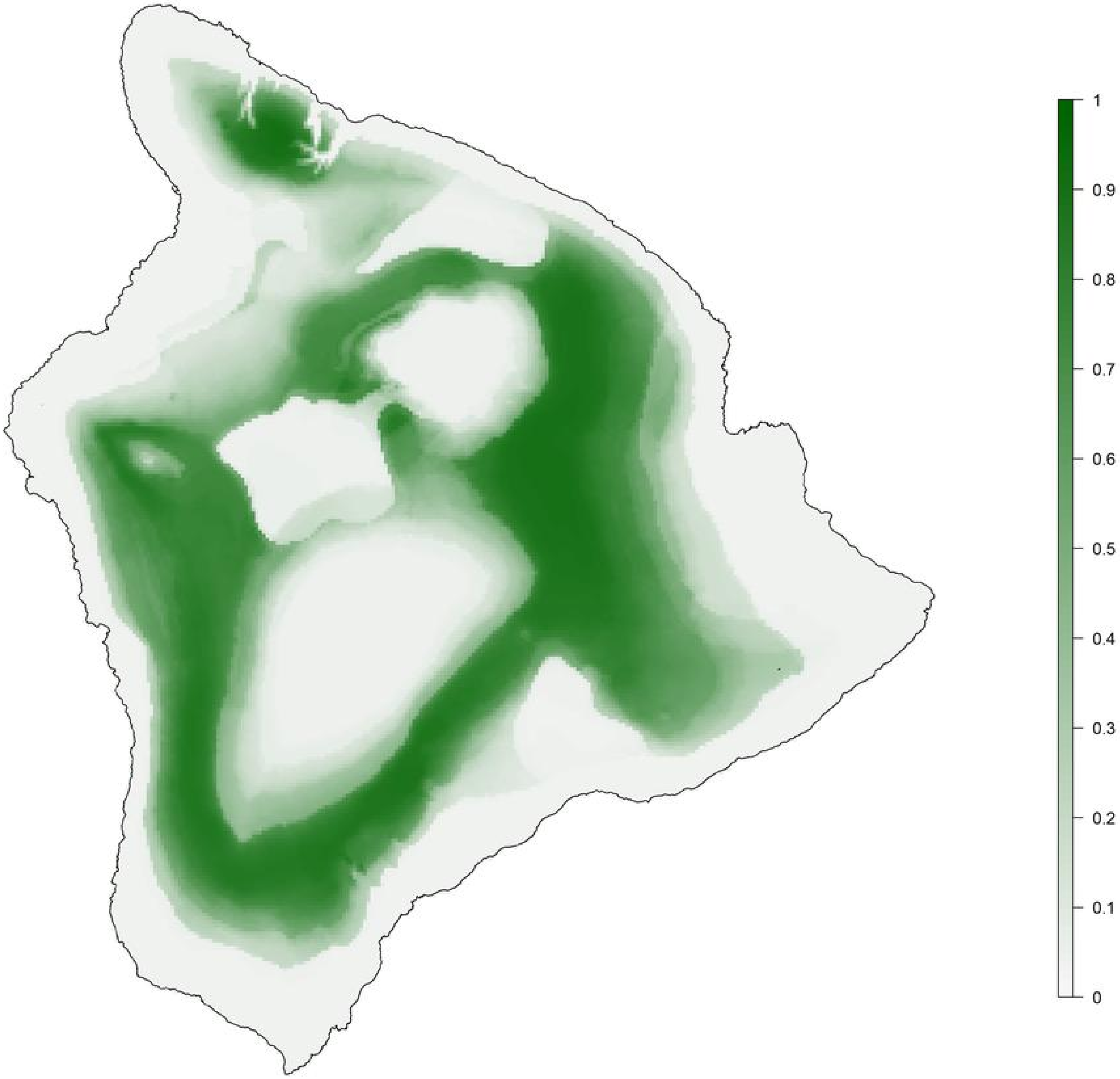

S1
REDUCED MODEL RELIABILITY SPECIES

Hawaii Elepaio future modeled suitability

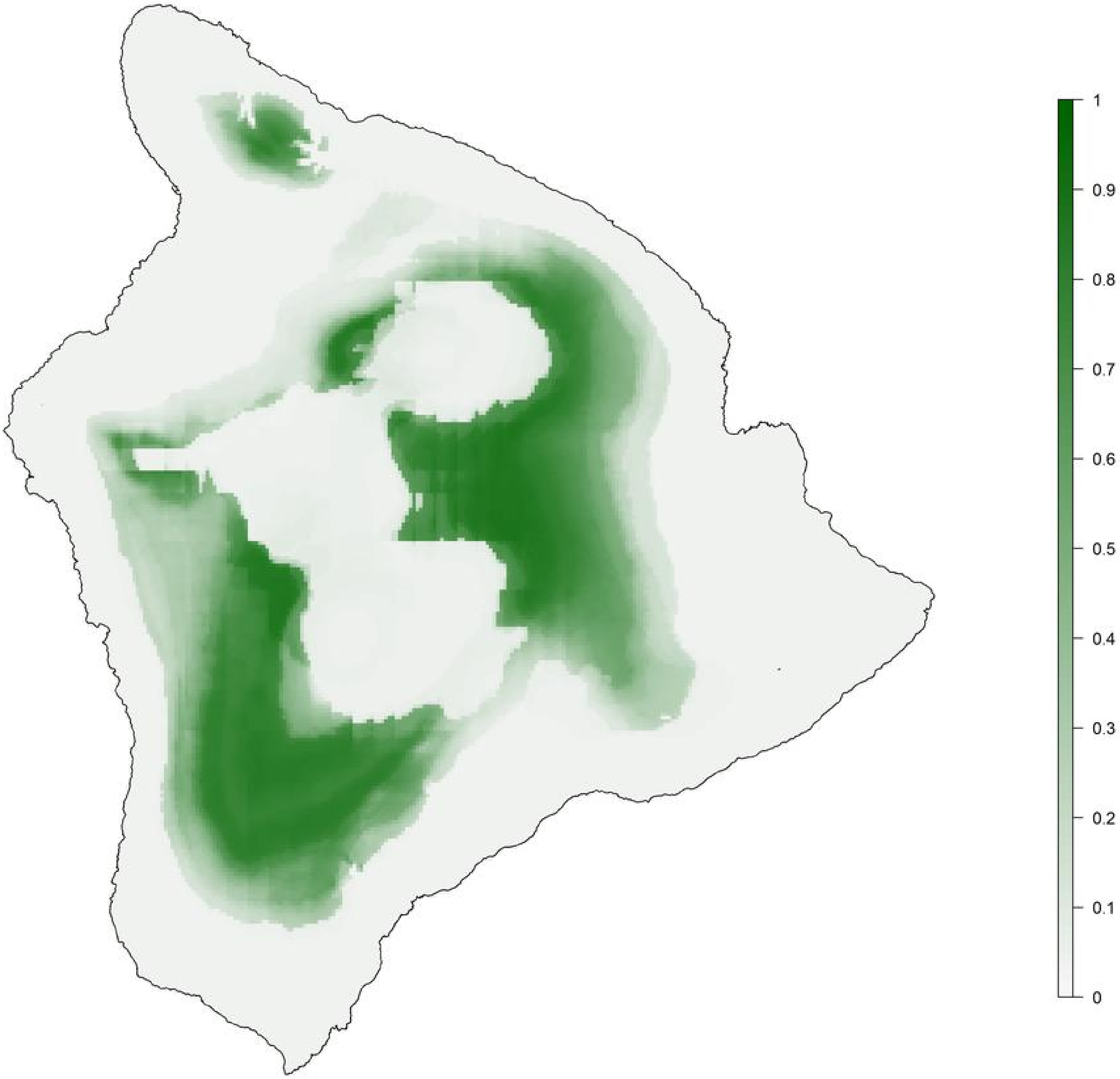

S1

REDUCED MODEL RELIABILITY SPECIES

Kauai Amakihi baseline modeled suitability

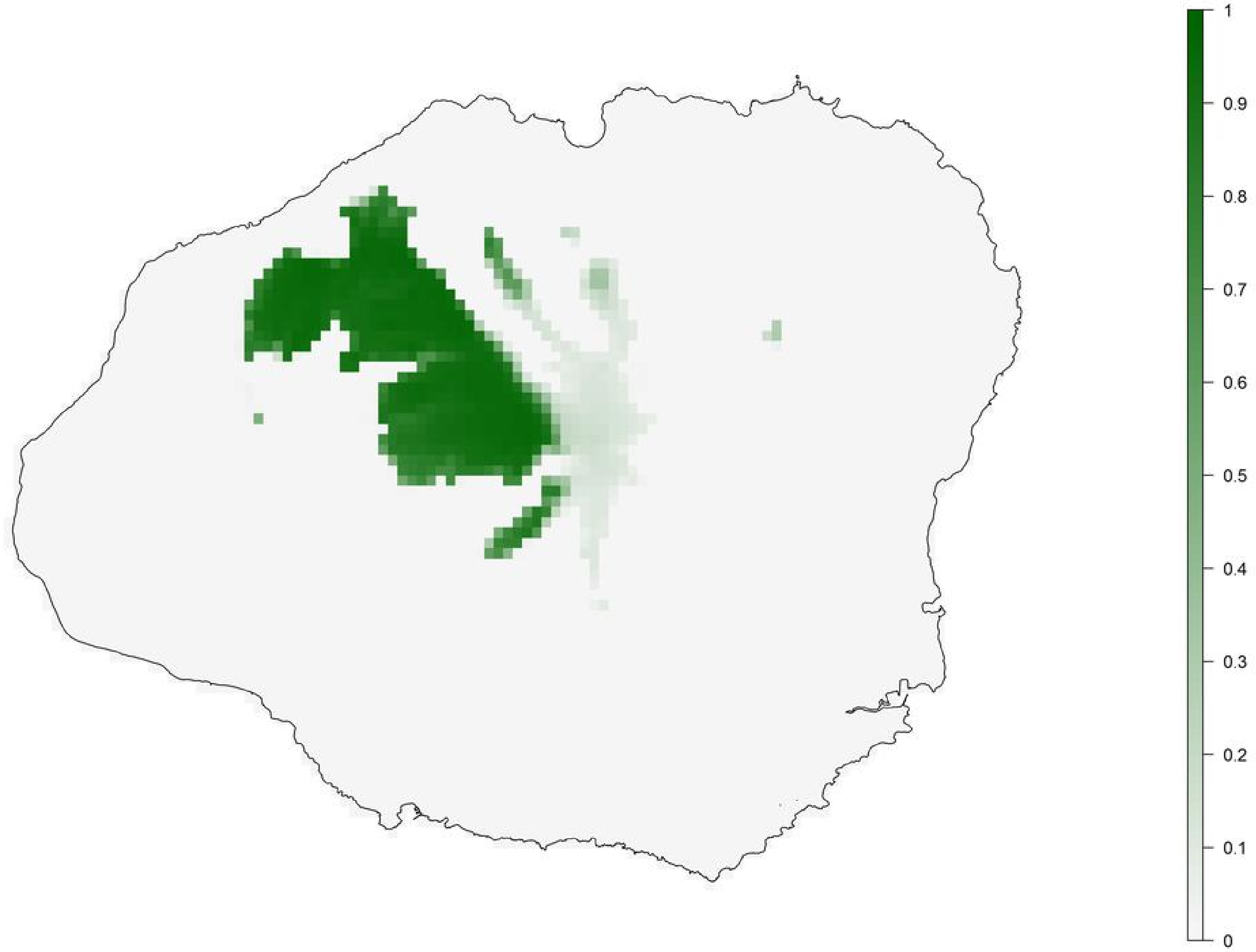

S1
REDUCED MODEL RELIABILITY SPECIES

Kauai Amakihi future modeled suitability

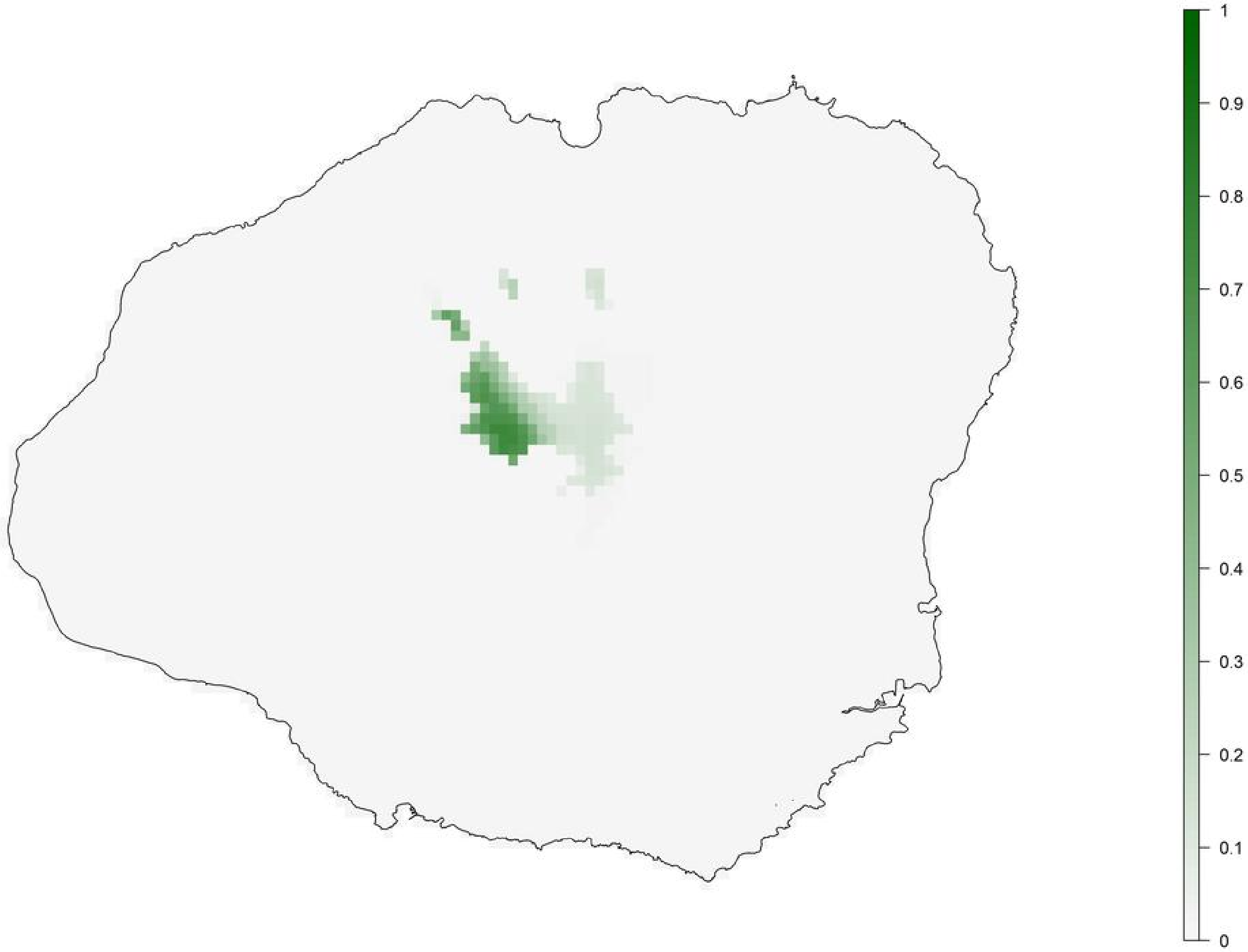

S1

REDUCED MODEL RELIABILITY SPECIES

Kauai Elepaio baseline modeled suitability

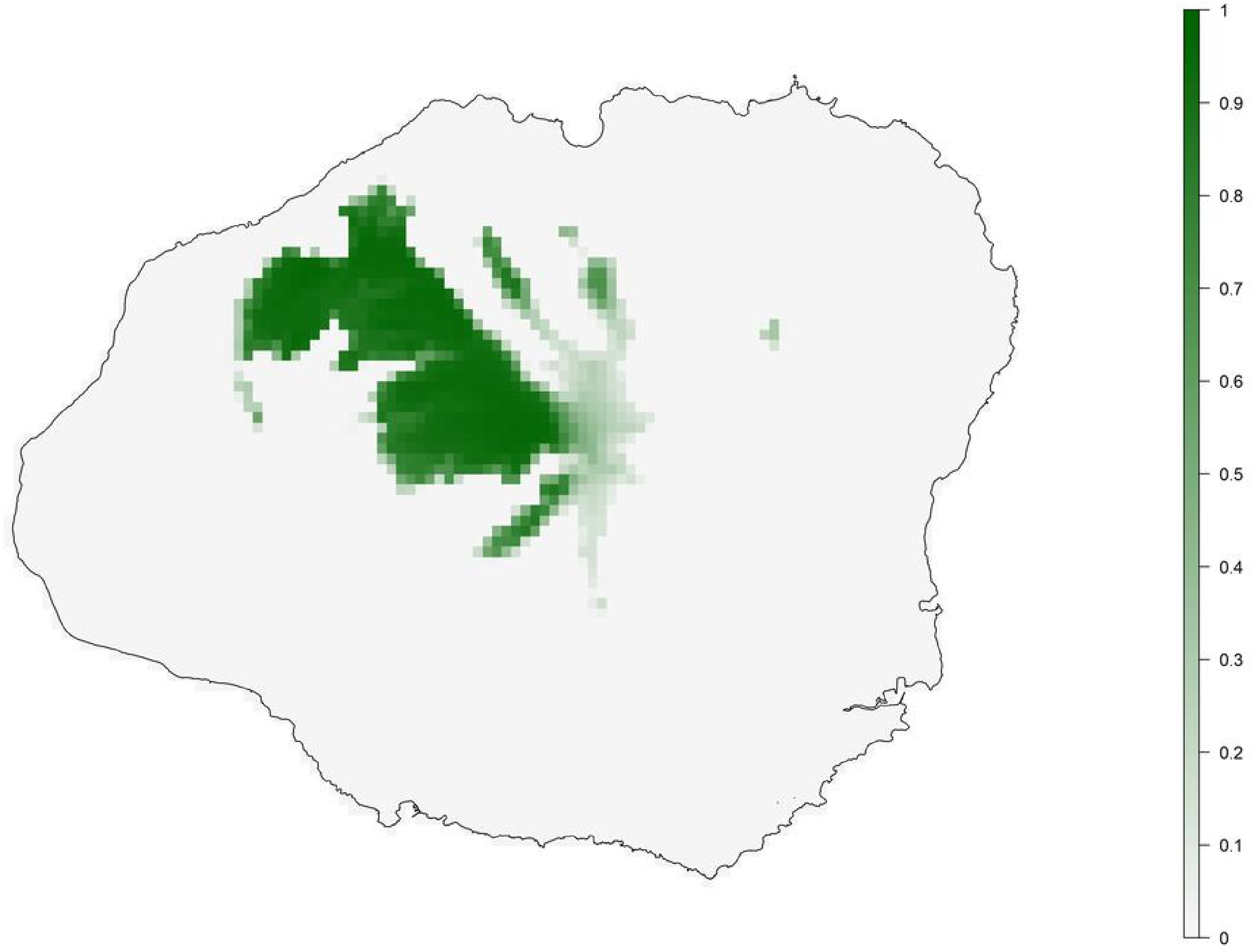

S1

REDUCED MODEL RELIABILITY SPECIES

Kauai Elepaio future modeled suitability

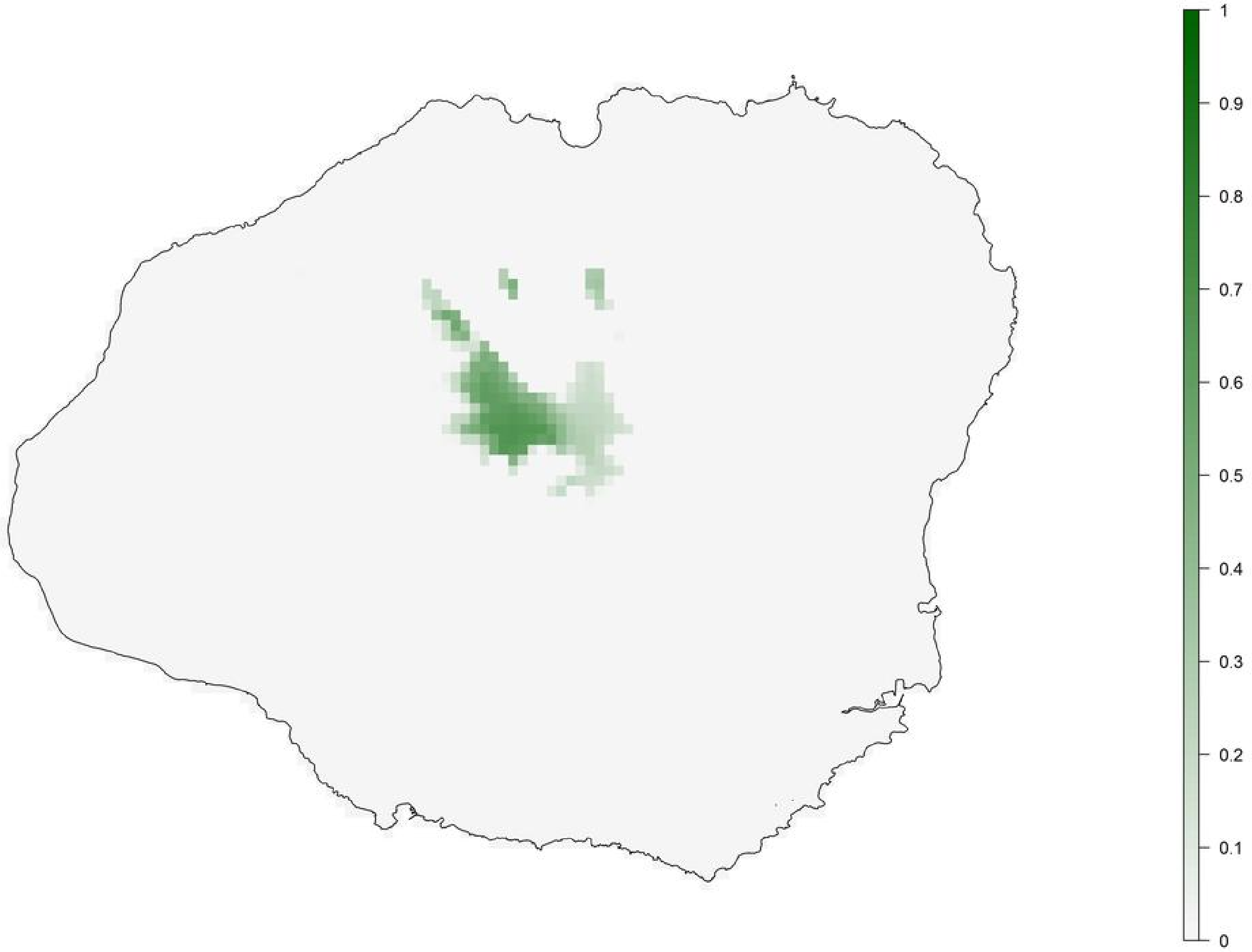

S1

REDUCED MODEL RELIABILITY SPECIES

Oahu Amakihi baseline modeled suitability

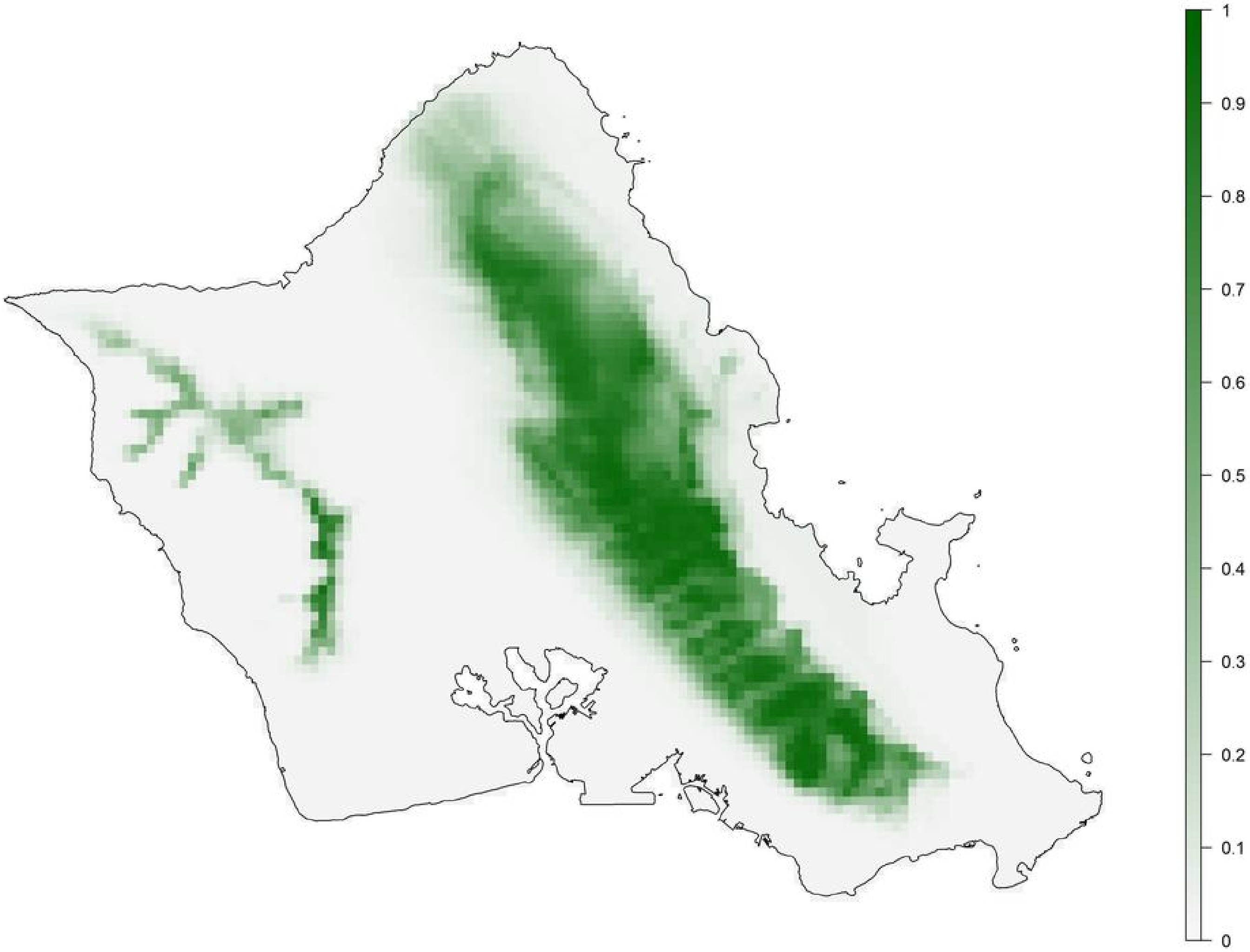

S1
REDUCED MODEL RELIABILITY SPECIES

Oahu Amakihi future modeled suitability

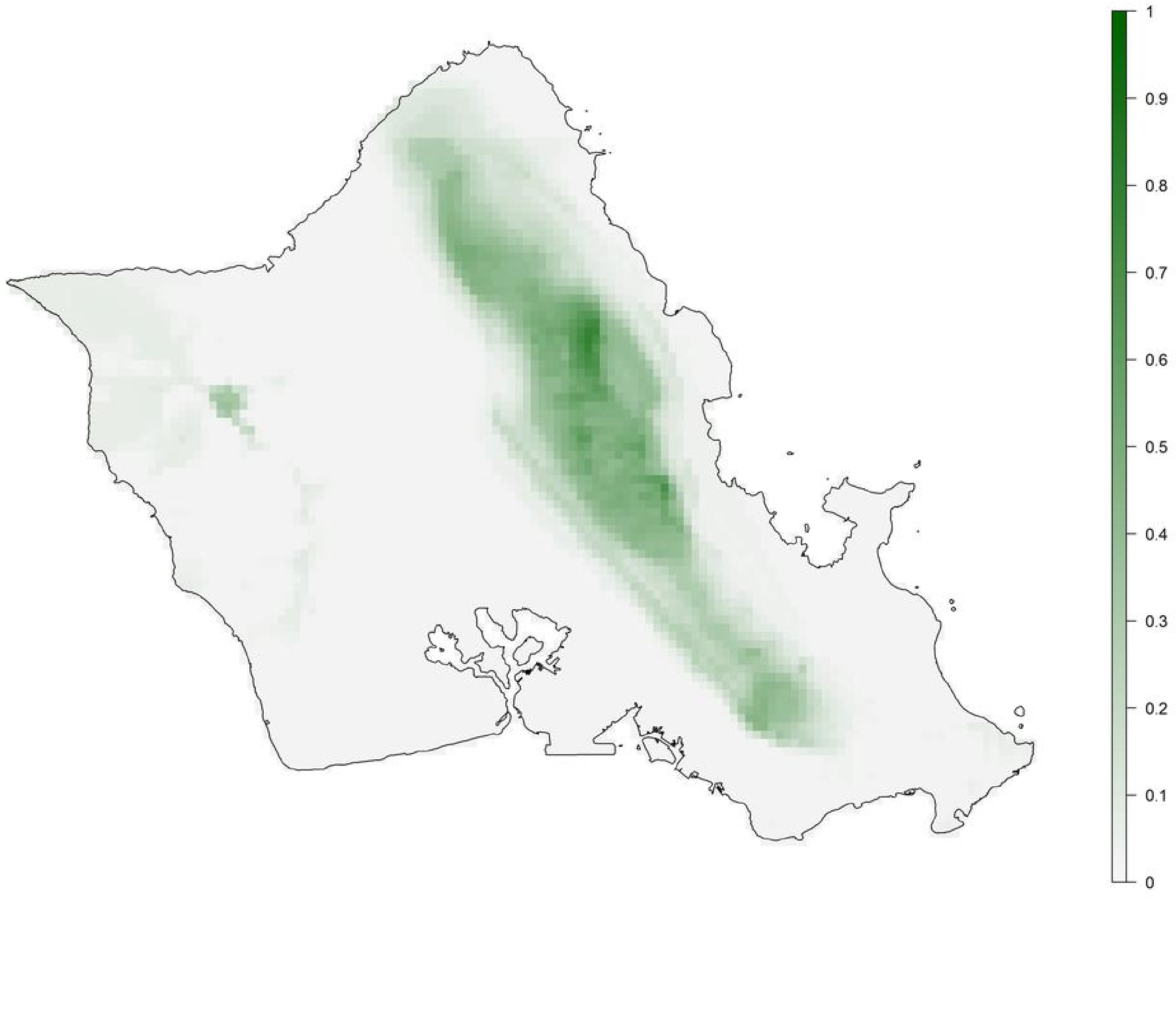

S1

REDUCED MODEL RELIABILITY SPECIES

Oahu Elepaio baseline modeled suitability

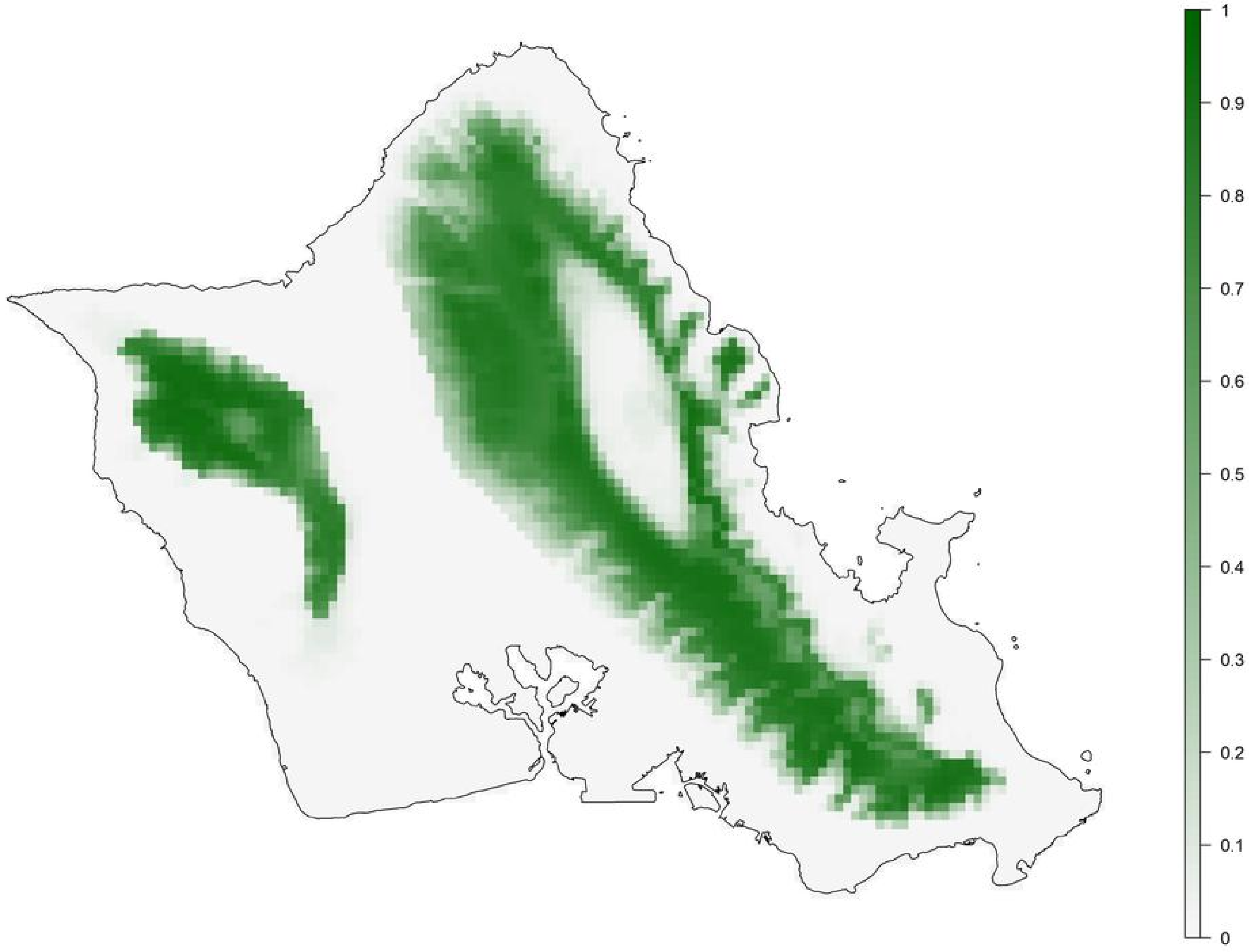

S1

REDUCED MODEL RELIABILITY SPECIES

Oahu Elepaio future modeled suitability

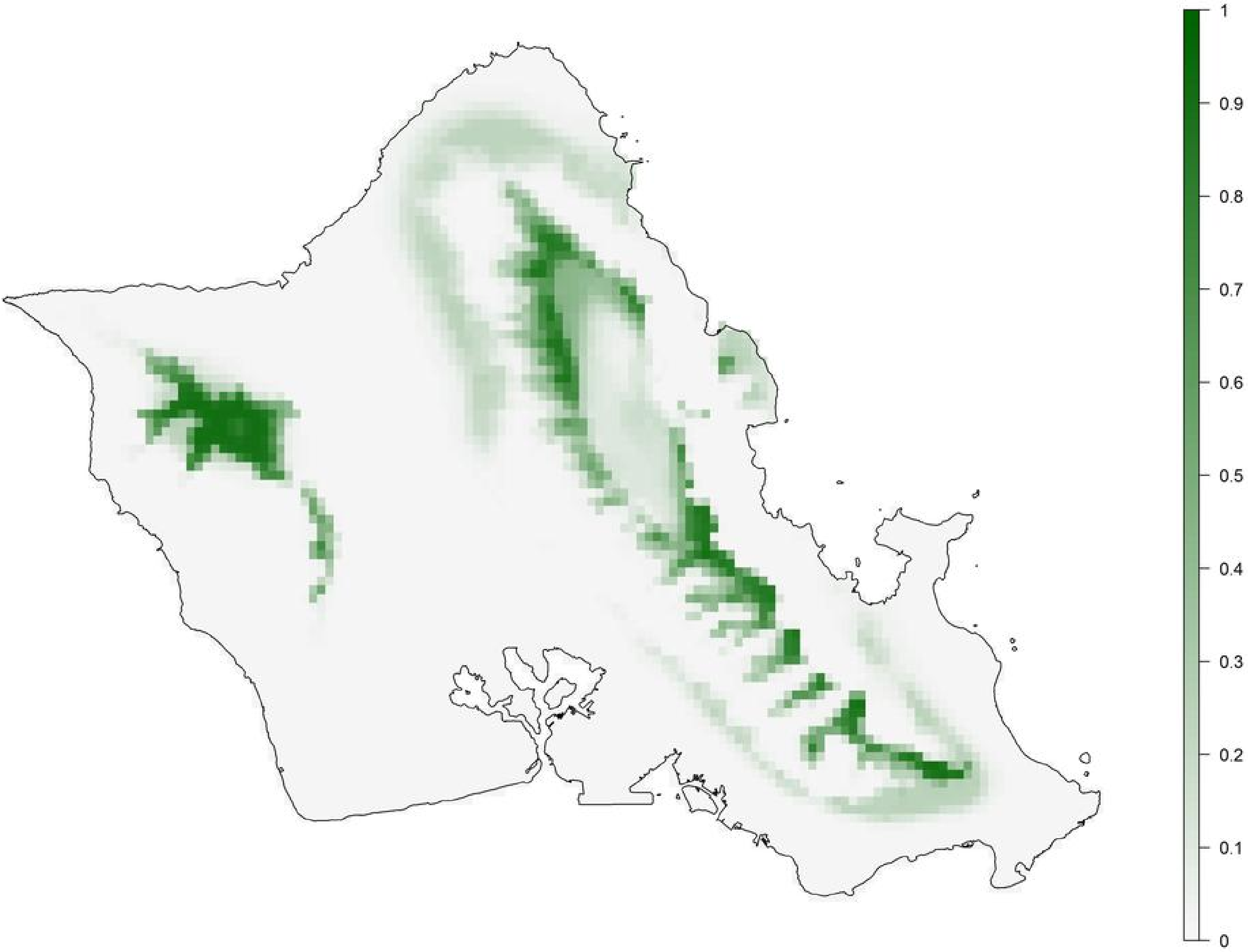

S1
REDUCED MODEL RELIABILITY SPECIES

Omao baseline modeled suitability

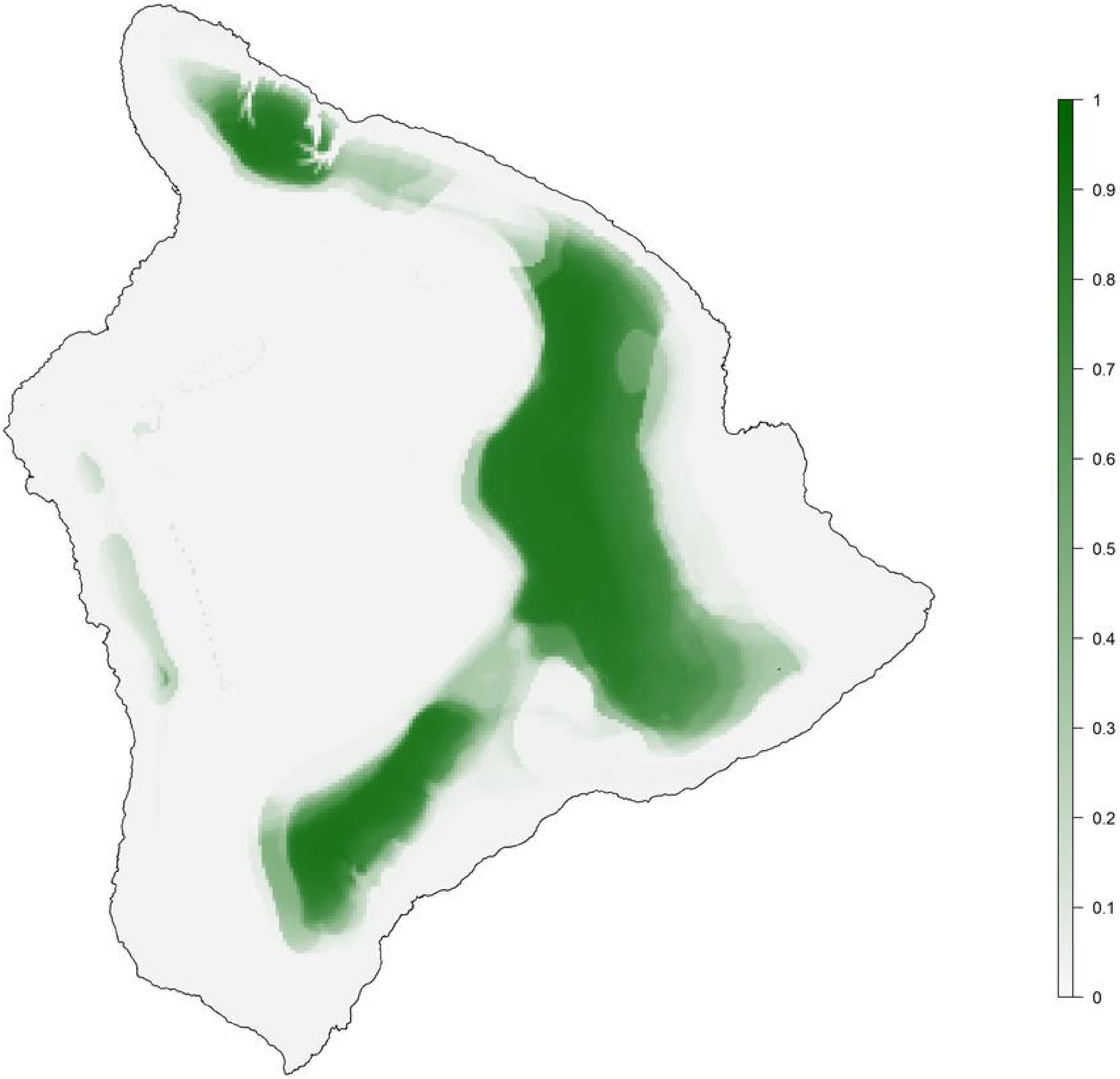

S1
REDUCED MODEL RELIABILITY SPECIES

Omao future modeled suitability

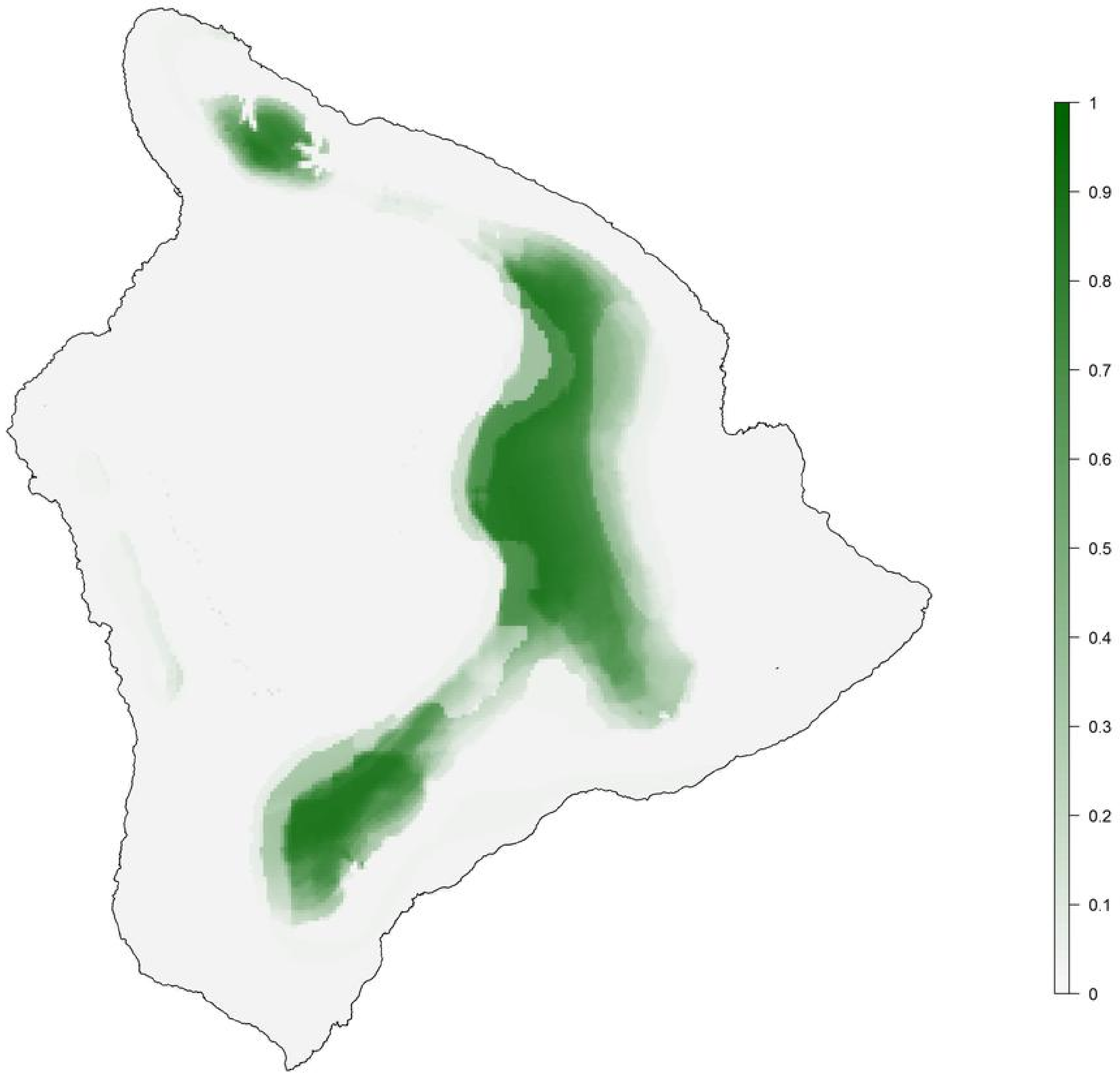

S1
REDUCED MODEL RELIABILITY SPECIES

Palila baseline modeled suitability

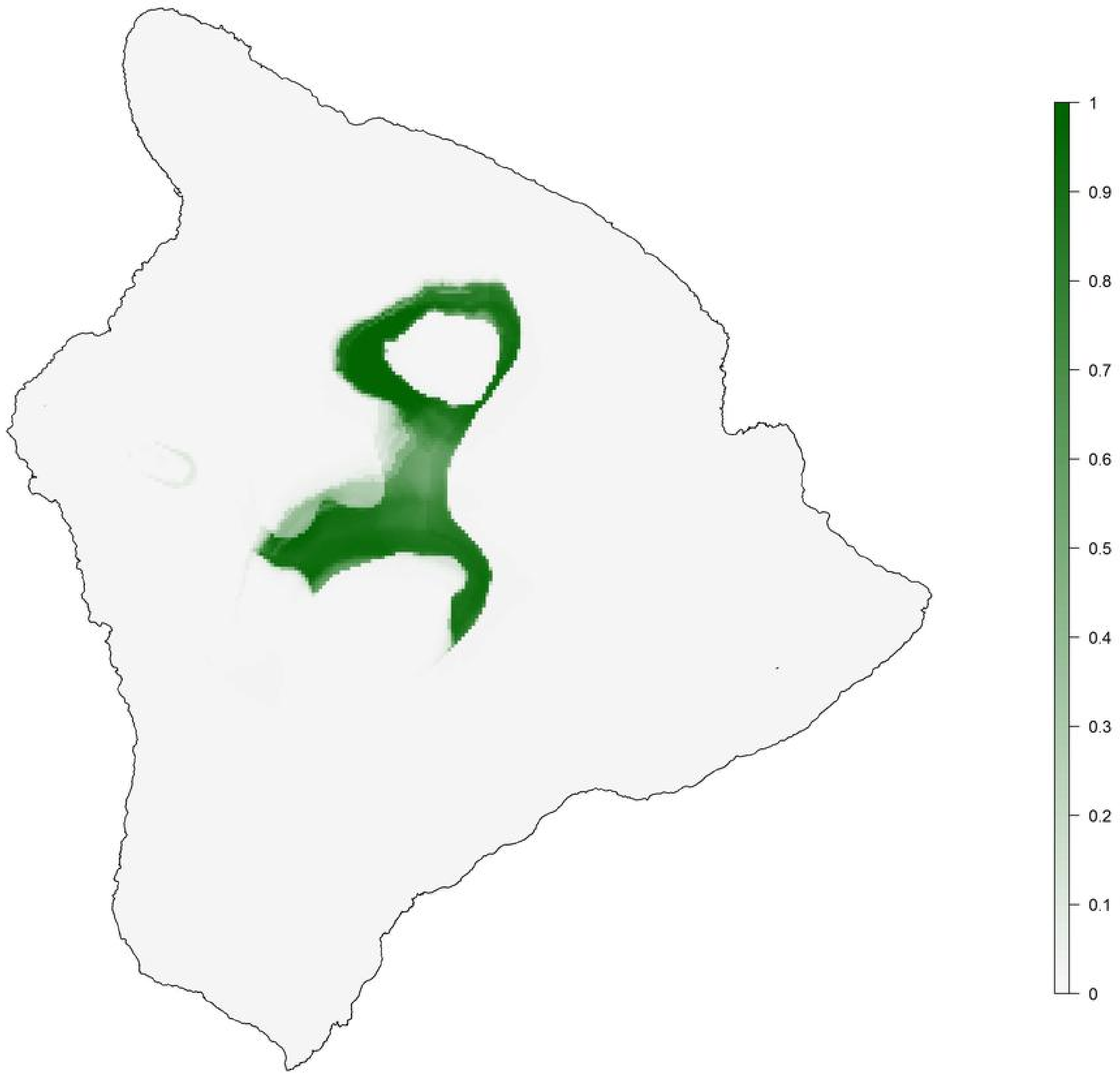

S1
REDUCED MODEL RELIABILITY SPECIES

Palila future modeled suitability

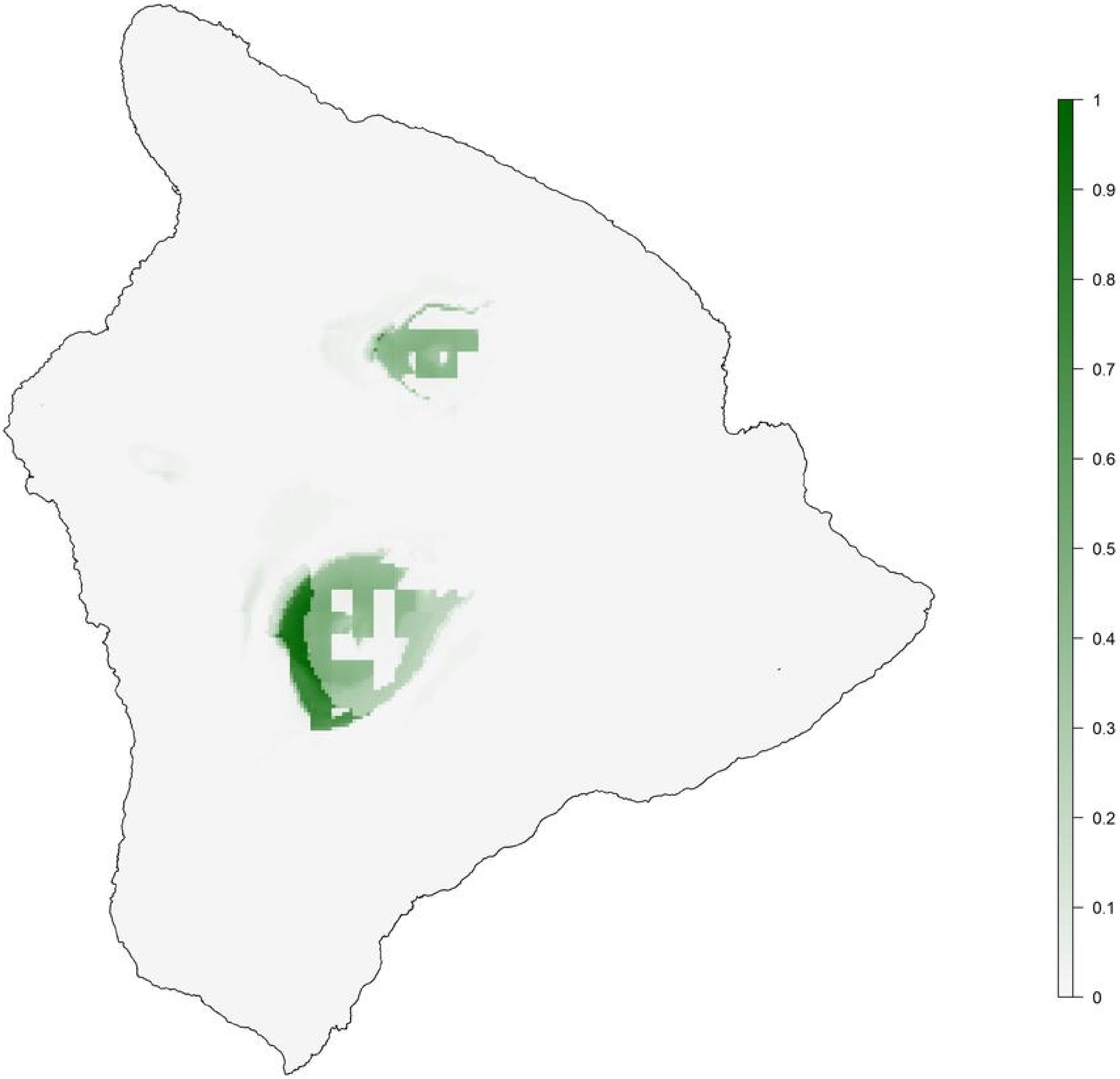

Supplement: S1 File — (PDF) [file pone.0140389.s001.pdf]
