# Supplementary material for: Large-Scale Range Collapse of Hawaiian Forest Birds under Climate Change and the Need 21st Century Conservation Options
Source: PLoS One. 2015 Oct 28;10(10):e0140389. doi: 10.1371/journal.pone.0140389 (PMC4625087; doi:10.1371/journal.pone.0140389)

Appendix S2. ROC and TSS evaluation scores for MaxEnt and GBM individual species models

ROC and TSS model evaluation scores for all species and modeling approaches.

Generated using the 1opt_model_eval_graph.R script in repository.

S2

ROC GBM variable importance box plot

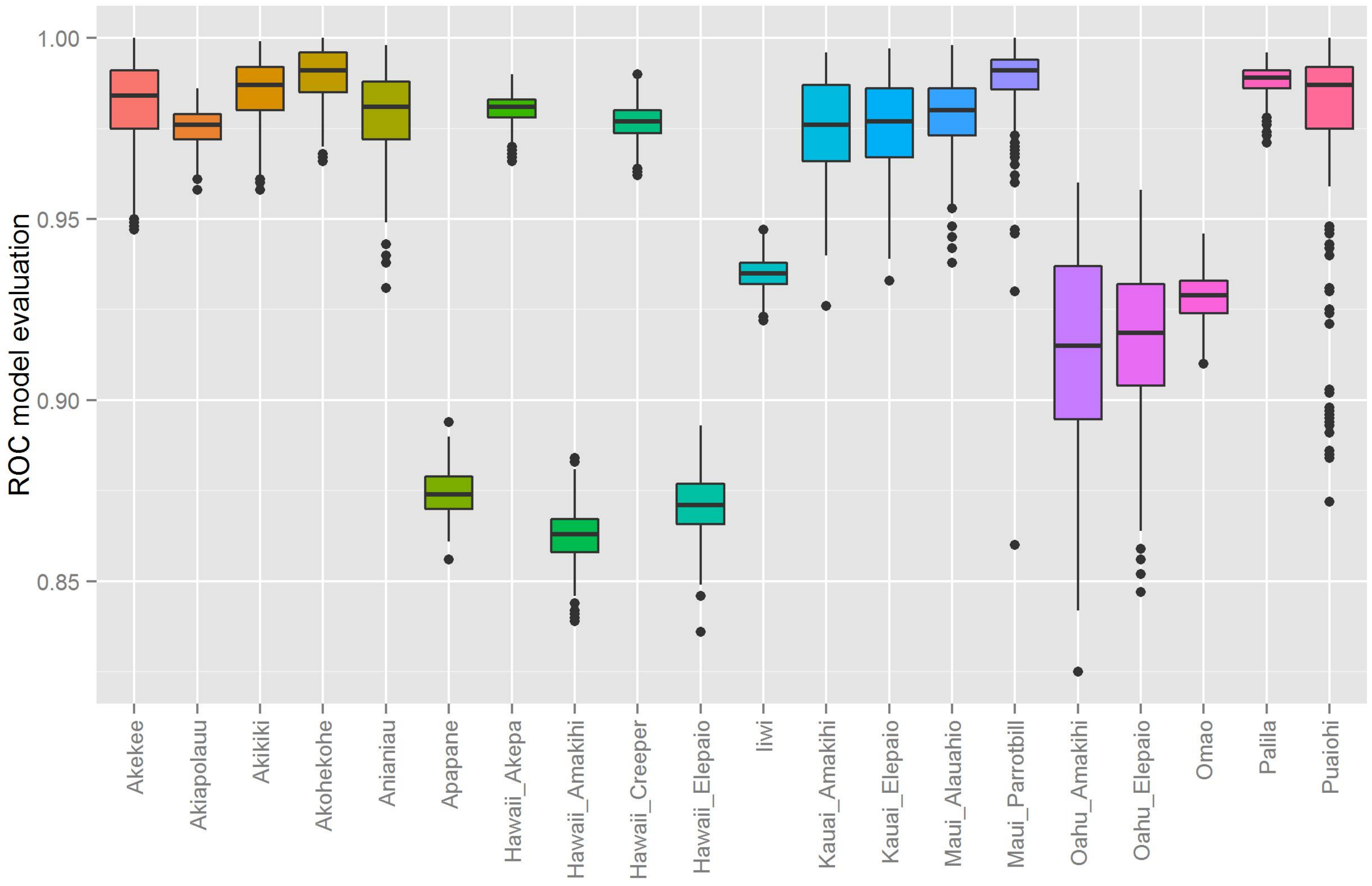

S2

ROC MAXENT variable importance box plot

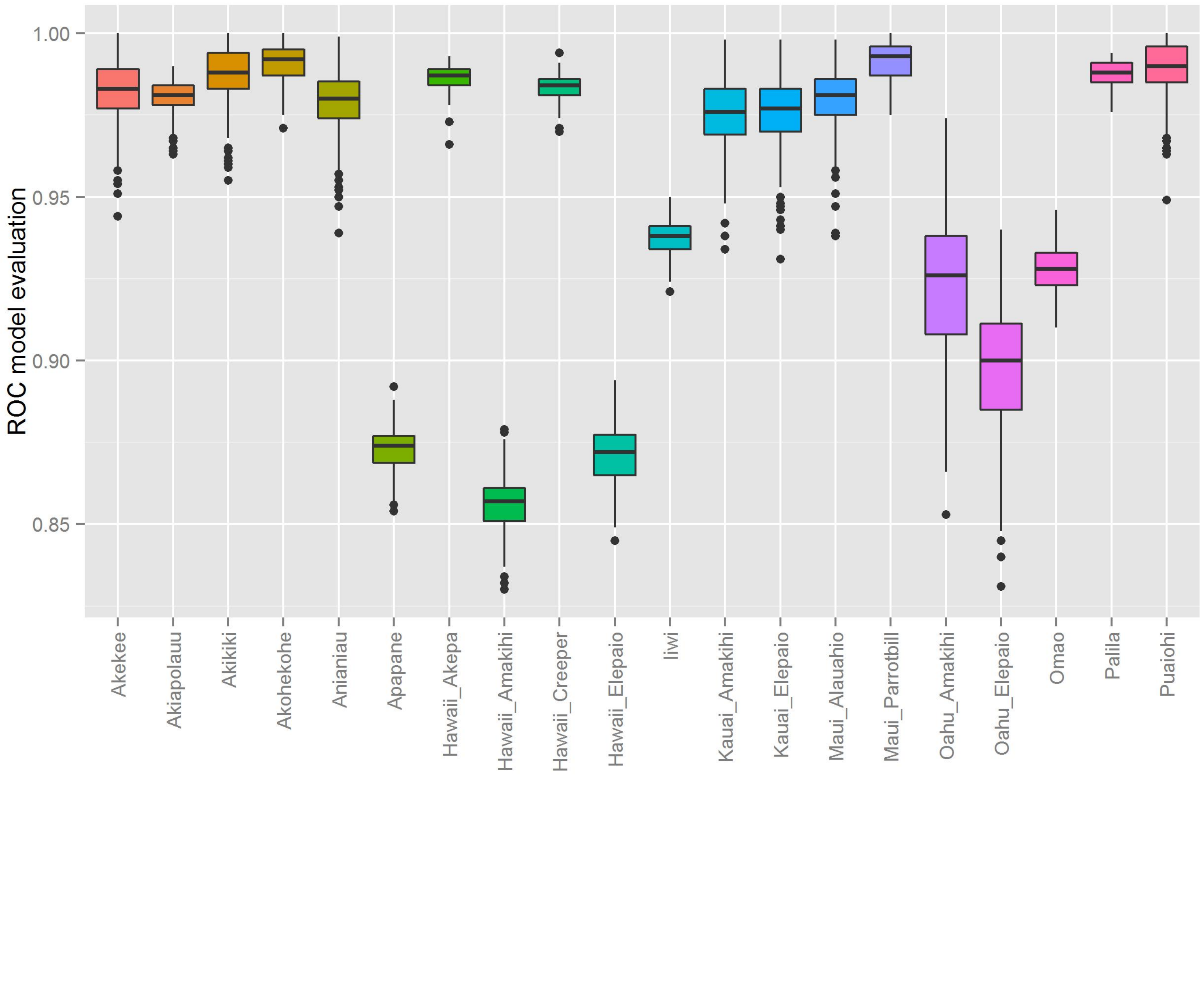

S2

TSS GBM variable importance box plot

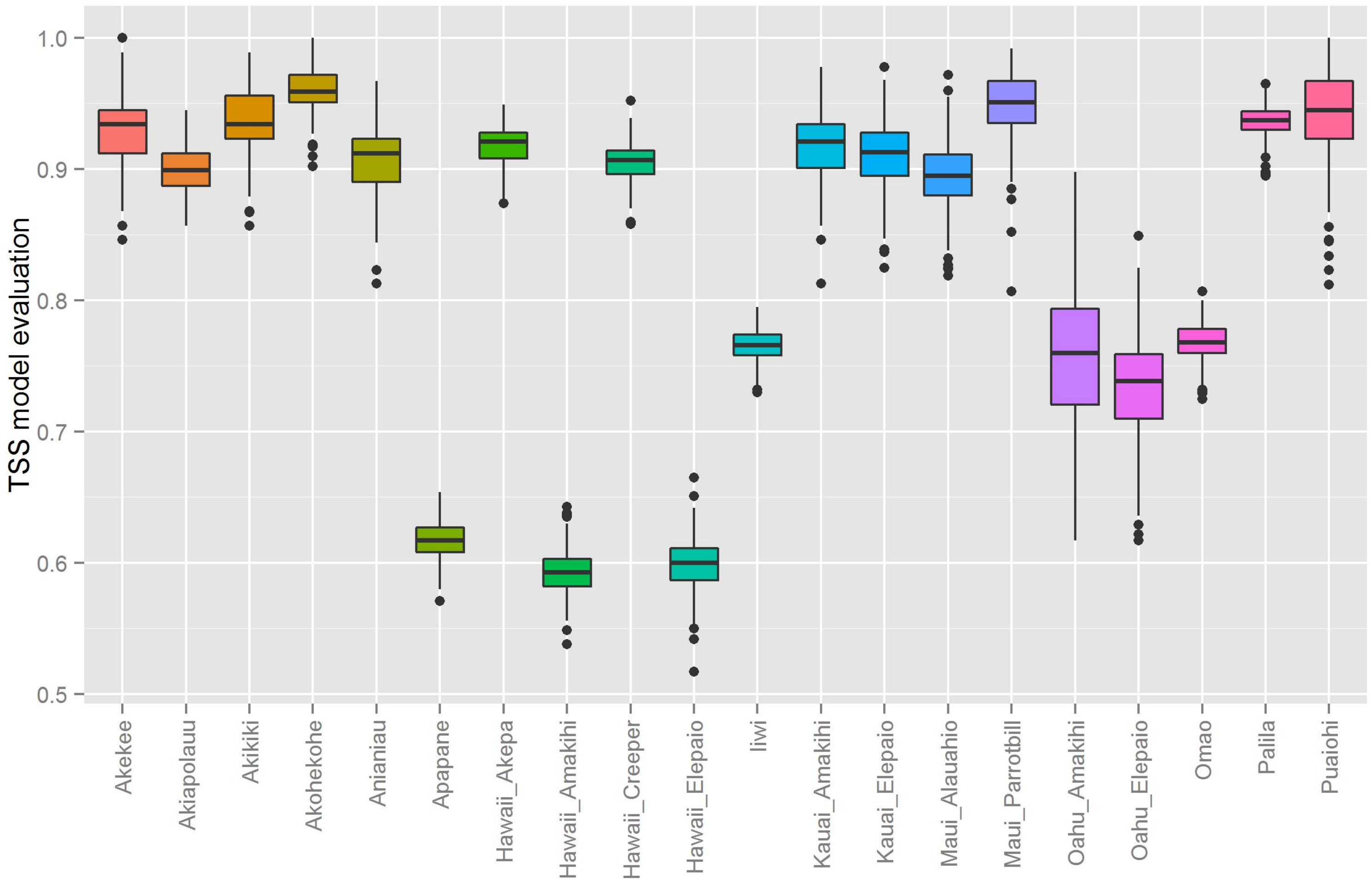

S2

TSS MAXENT variable importance box plot

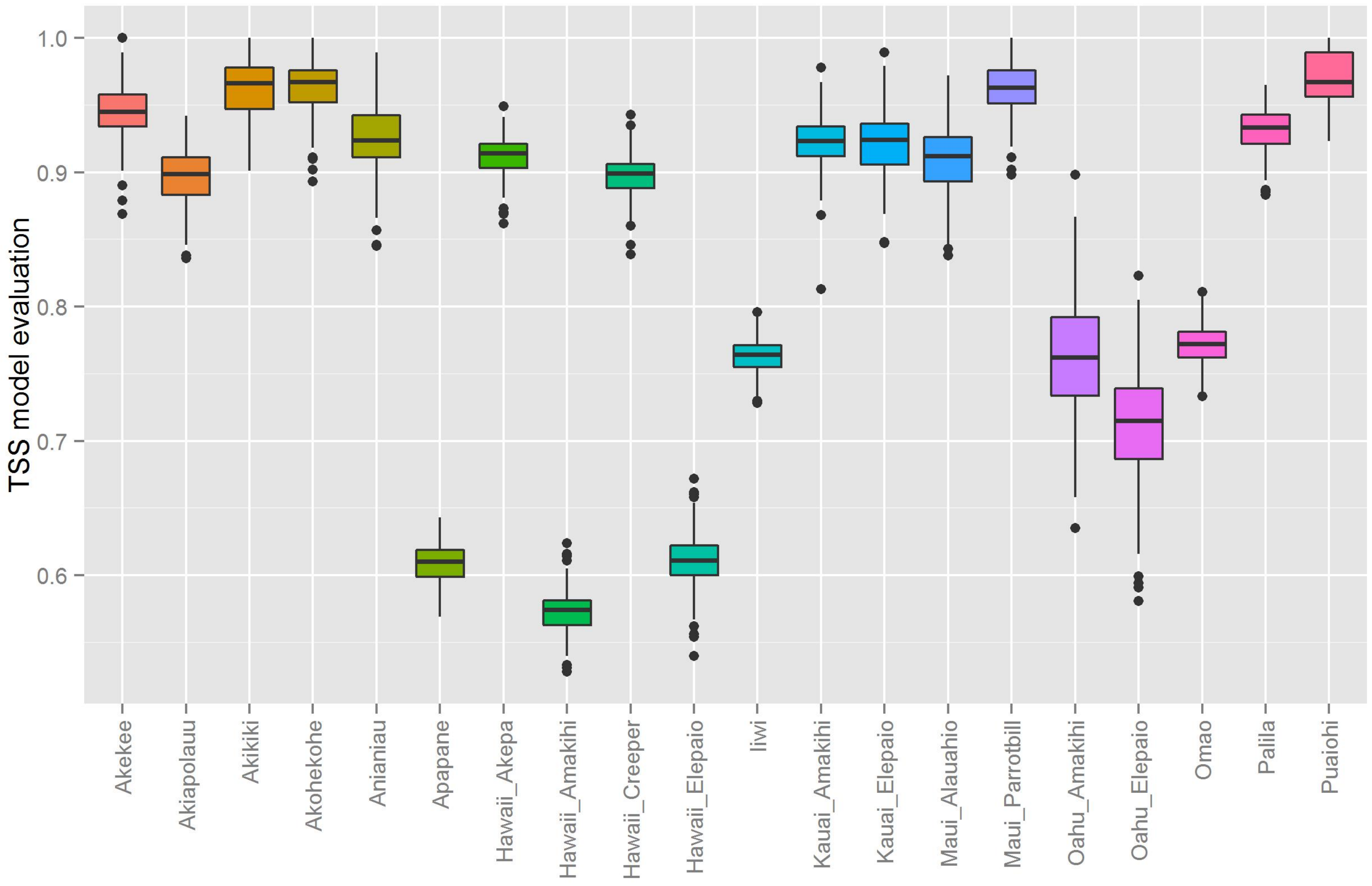

Supplement: S2 File — (PDF) [file pone.0140389.s002.pdf]
