# Supplementary material for: Large-Scale Range Collapse of Hawaiian Forest Birds under Climate Change and the Need 21st Century Conservation Options
Source: PLoS One. 2015 Oct 28;10(10):e0140389. doi: 10.1371/journal.pone.0140389 (PMC4625087; doi:10.1371/journal.pone.0140389)

Appendix S3. Response curve and model predictor importance graphs for all Hawaiian forest bird species models.

First section of appendix includes species response curves for all individual species with a curve for each model run in grey and the mean response curve in black. The second section of the appendix includes predictor importance graphs for all species and modeling approaches. We use bioclim variable names for all predictors in all included graphs: mean annual temperature (**Bio1**), mean annual precipitation (**Bio12**), temperature annual range (**Bio7**), and precipitation seasonality (**Bio15**). Predictor importance graphs were generated using the 1opt_var_importance_graph.R script in repository. The response curves were generated using the 2opt_BM2_FB_SDM_response_curves.r script.

S3: SPECIES RESPONSE CURVES
HIGH MODEL RELIABILITY SPECIES

Akekee response curves GBM all vars

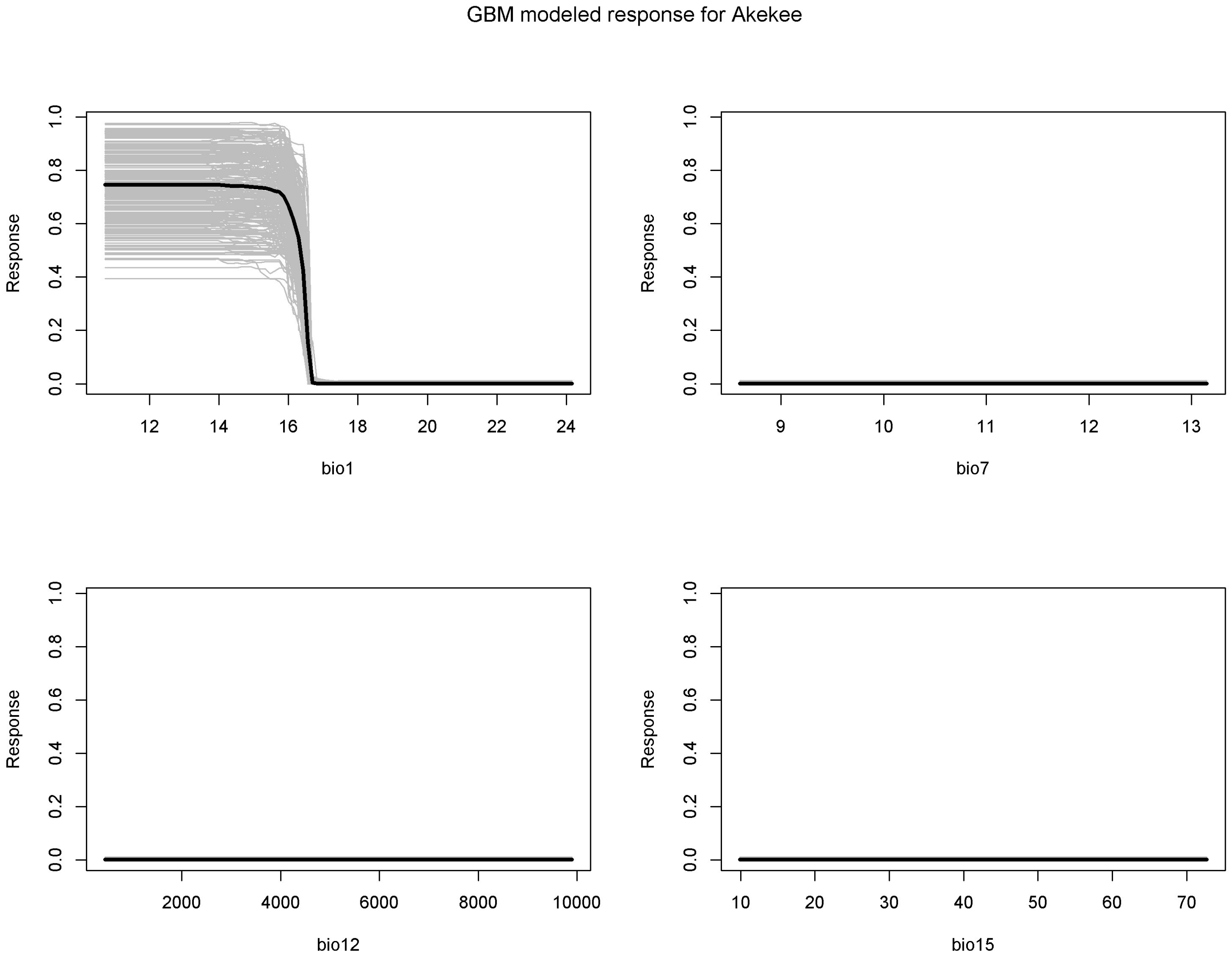

S3: SPECIES RESPONSE CURVES
HIGH MODEL RELIABILITY SPECIES

Akekee response curves MAXENT all vars

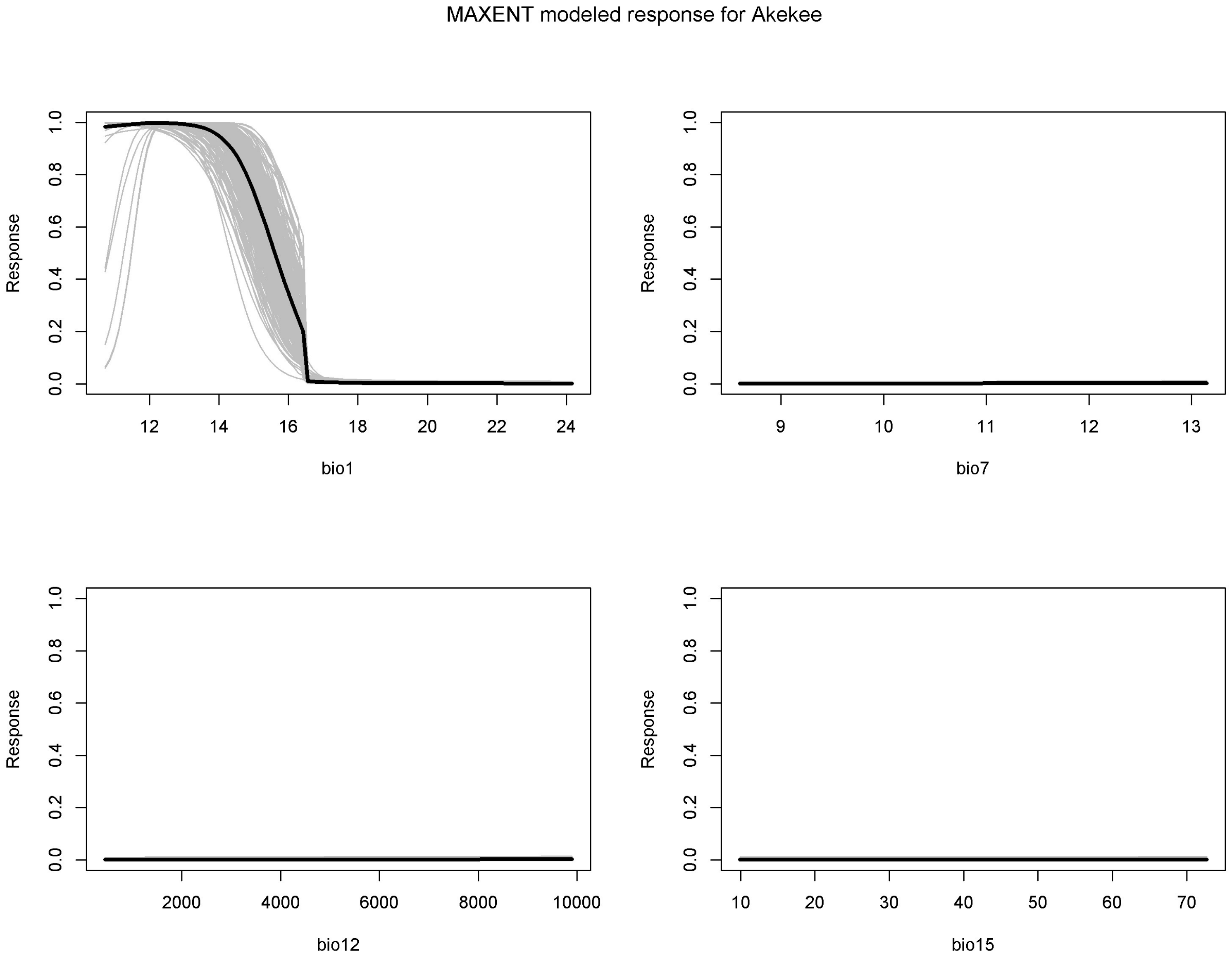

S3: SPECIES RESPONSE CURVES
HIGH MODEL RELIABILITY SPECIES

Akiapolauu response curves GBM all vars

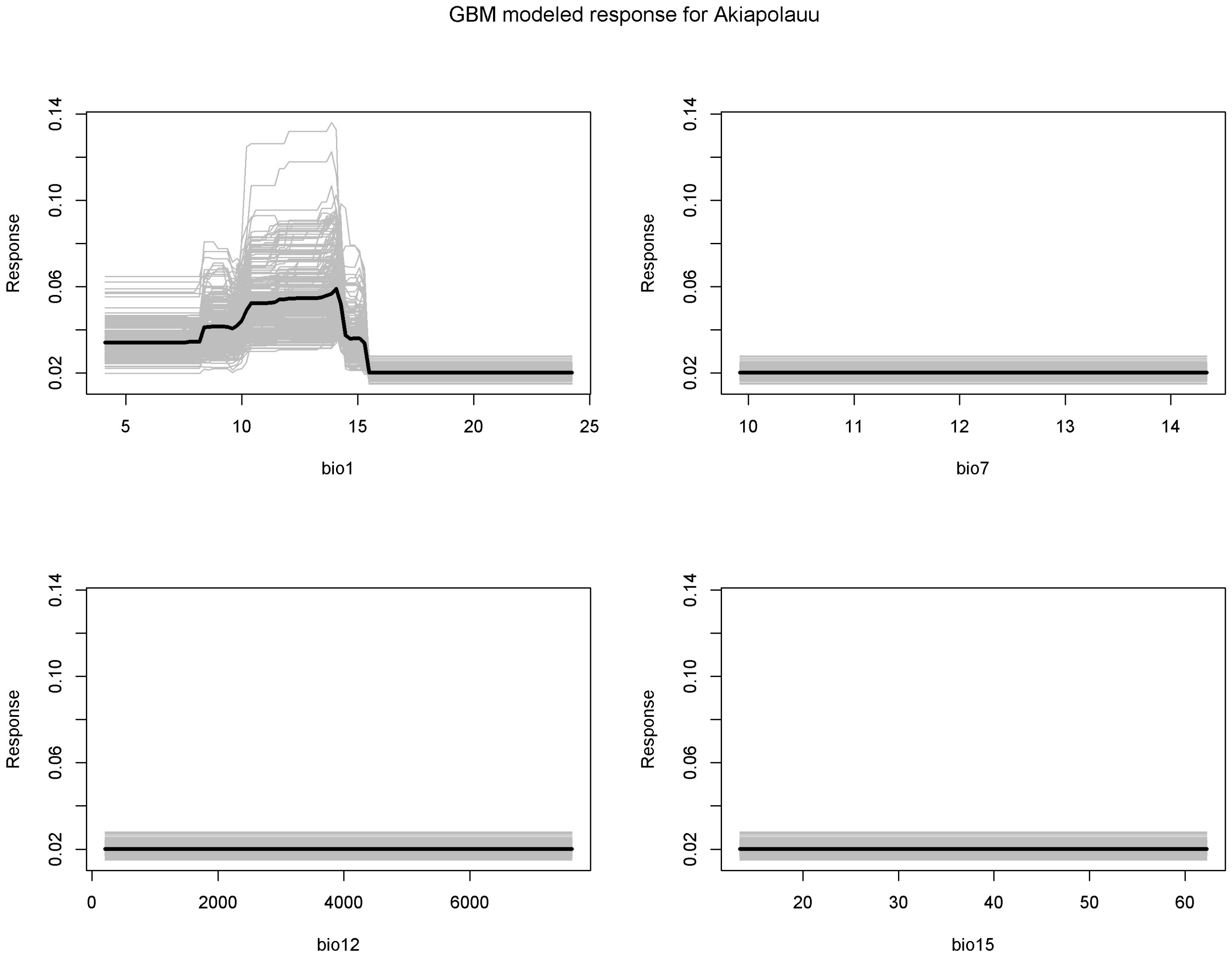

S3: SPECIES RESPONSE CURVES
HIGH MODEL RELIABILITY SPECIES

Akiapolauu response curves MAXENT all vars

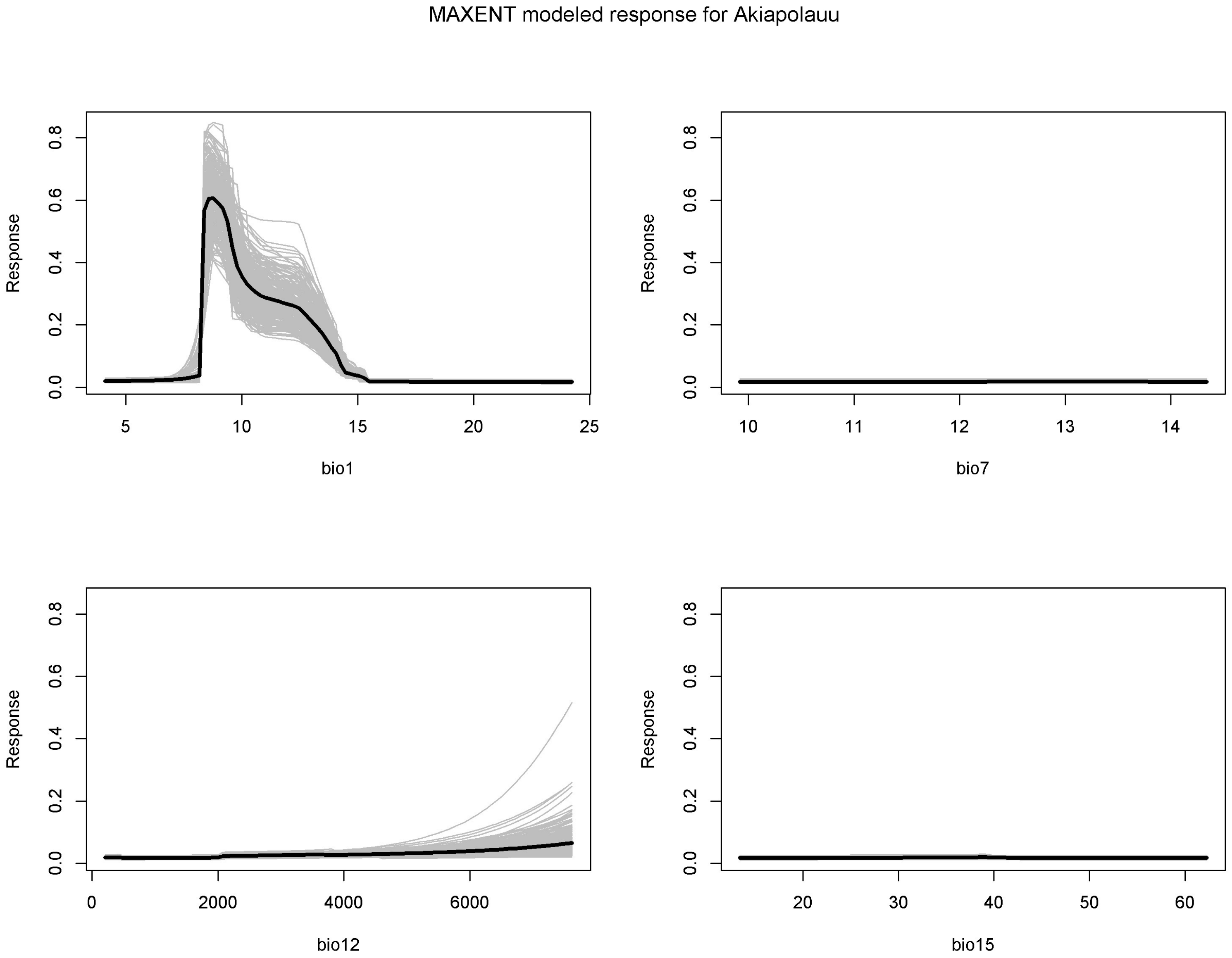

S3: SPECIES RESPONSE CURVES
HIGH MODEL RELIABILITY SPECIES

Akikiki response curves GBM all vars

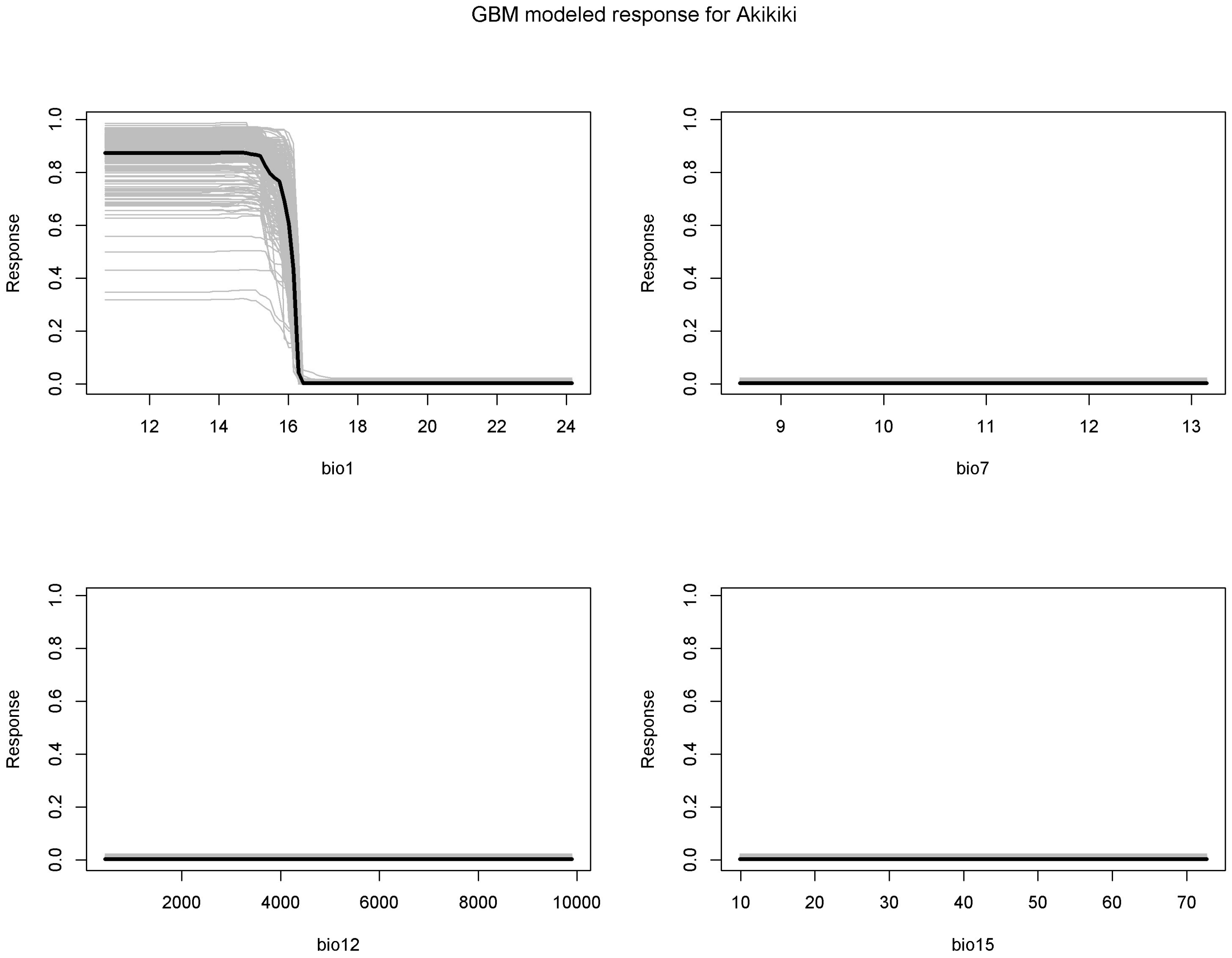

S3: SPECIES RESPONSE CURVES
HIGH MODEL RELIABILITY SPECIES

Akikiki response curves MAXENT all vars

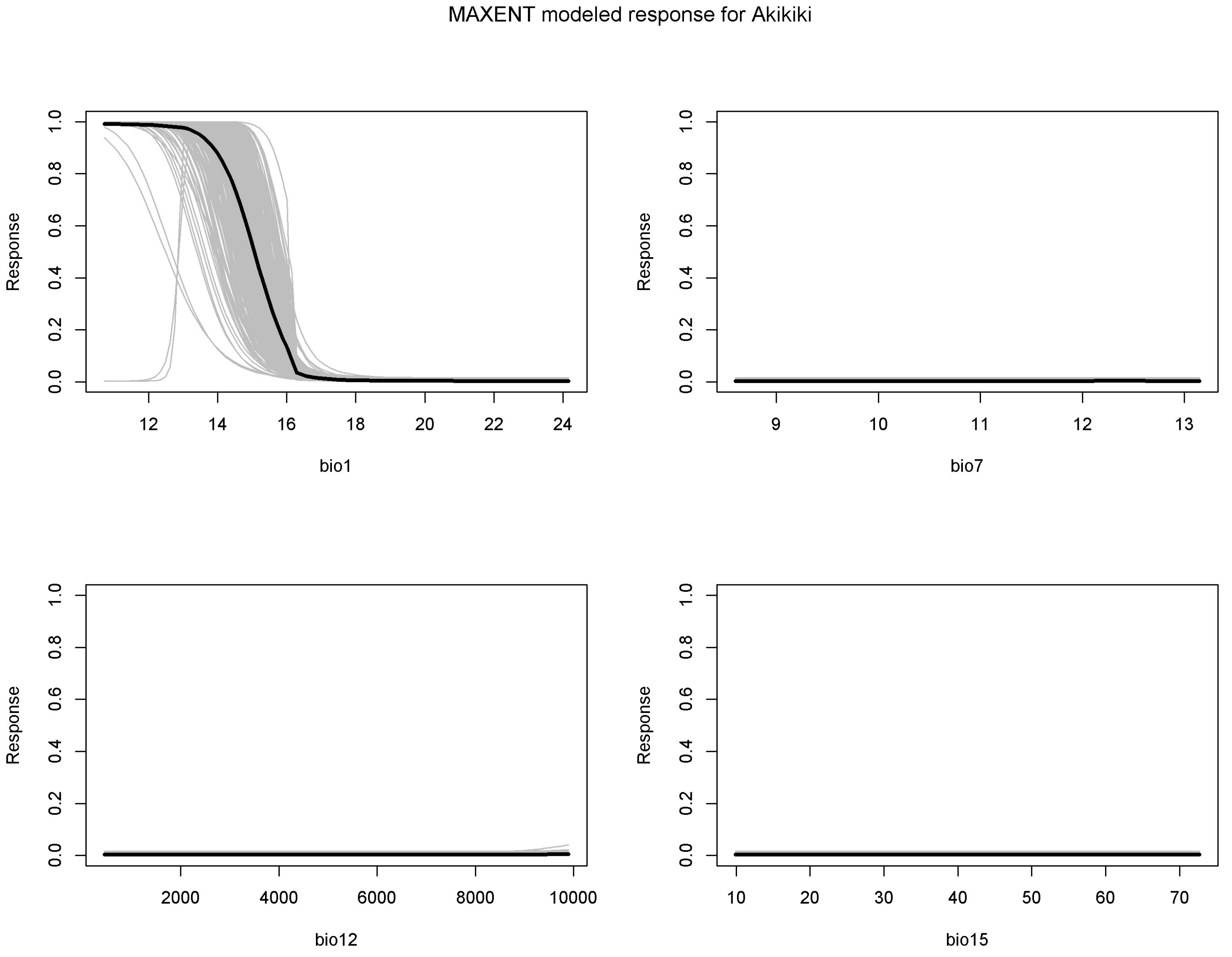

S3: SPECIES RESPONSE CURVES
HIGH MODEL RELIABILITY SPECIES

Akohekohe response curves GBM all vars

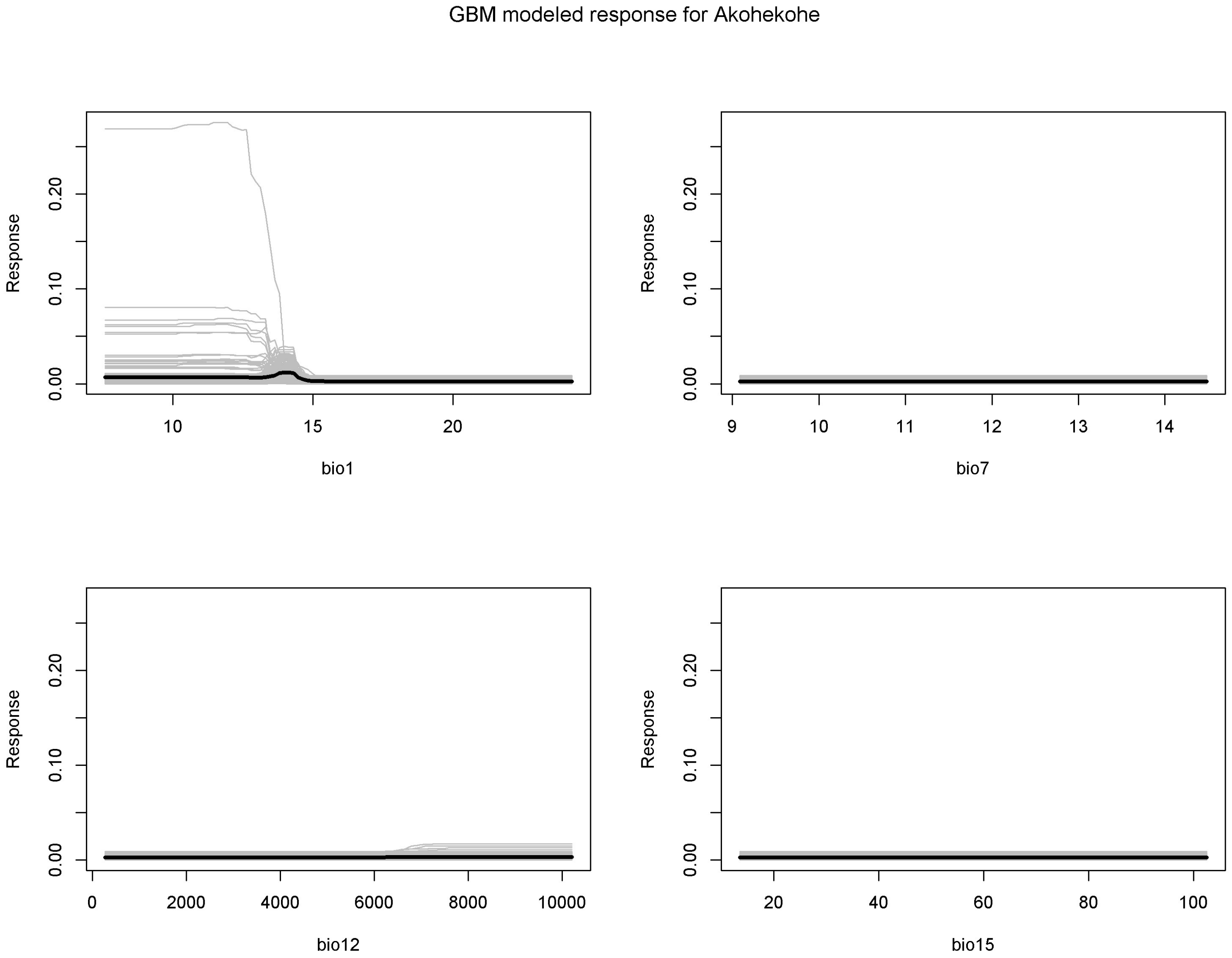

S3: SPECIES RESPONSE CURVES
HIGH MODEL RELIABILITY SPECIES

Akohekohe response curves MAXENT all vars

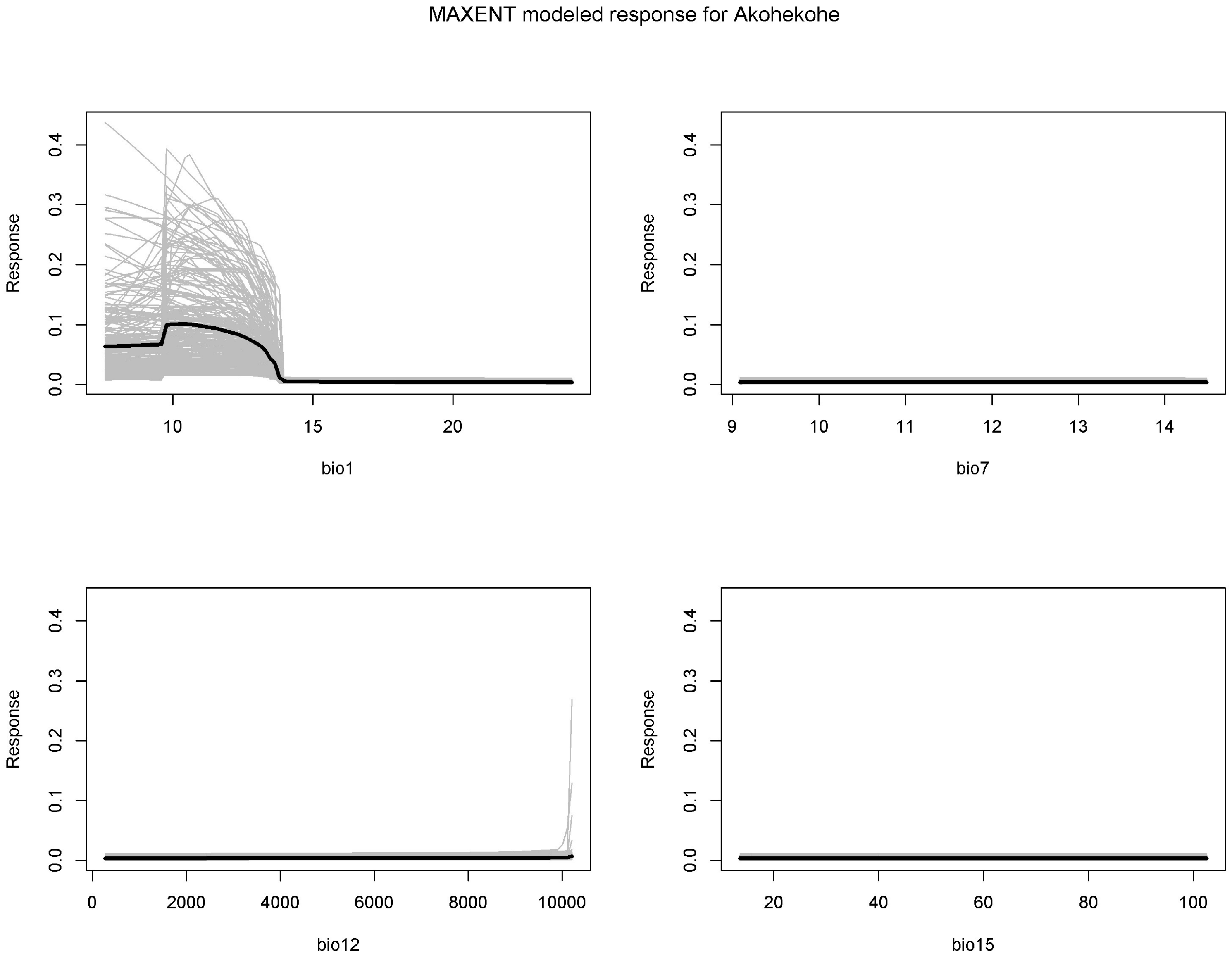

S3: SPECIES RESPONSE CURVES
HIGH MODEL RELIABILITY SPECIES

Hawaii.Akepa response curves GBM all vars

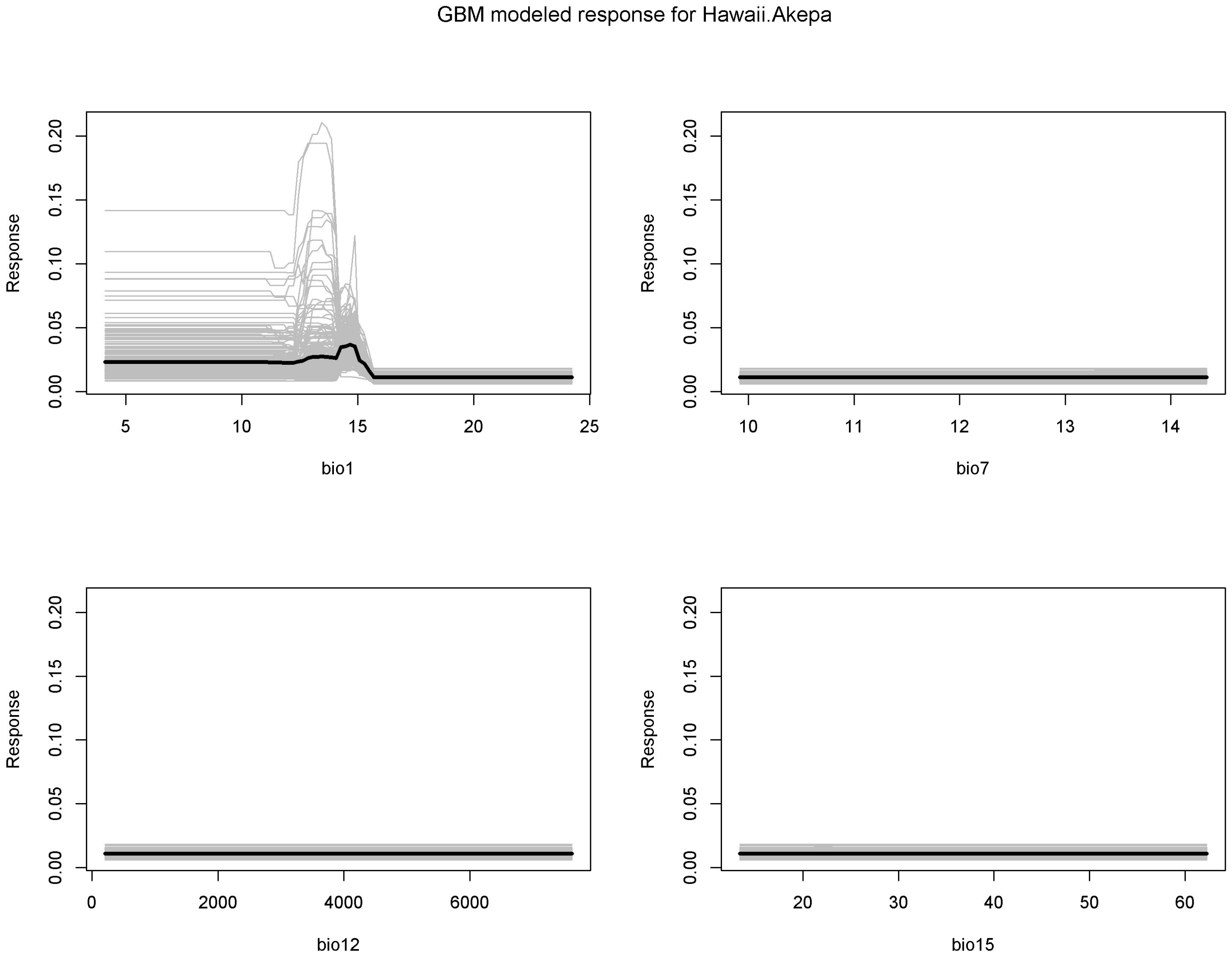

S3: SPECIES RESPONSE CURVES
HIGH MODEL RELIABILITY SPECIES

Hawaii.Akepa response curves MAXENT all vars

MAXENT modeled response for Hawaii.Akepa

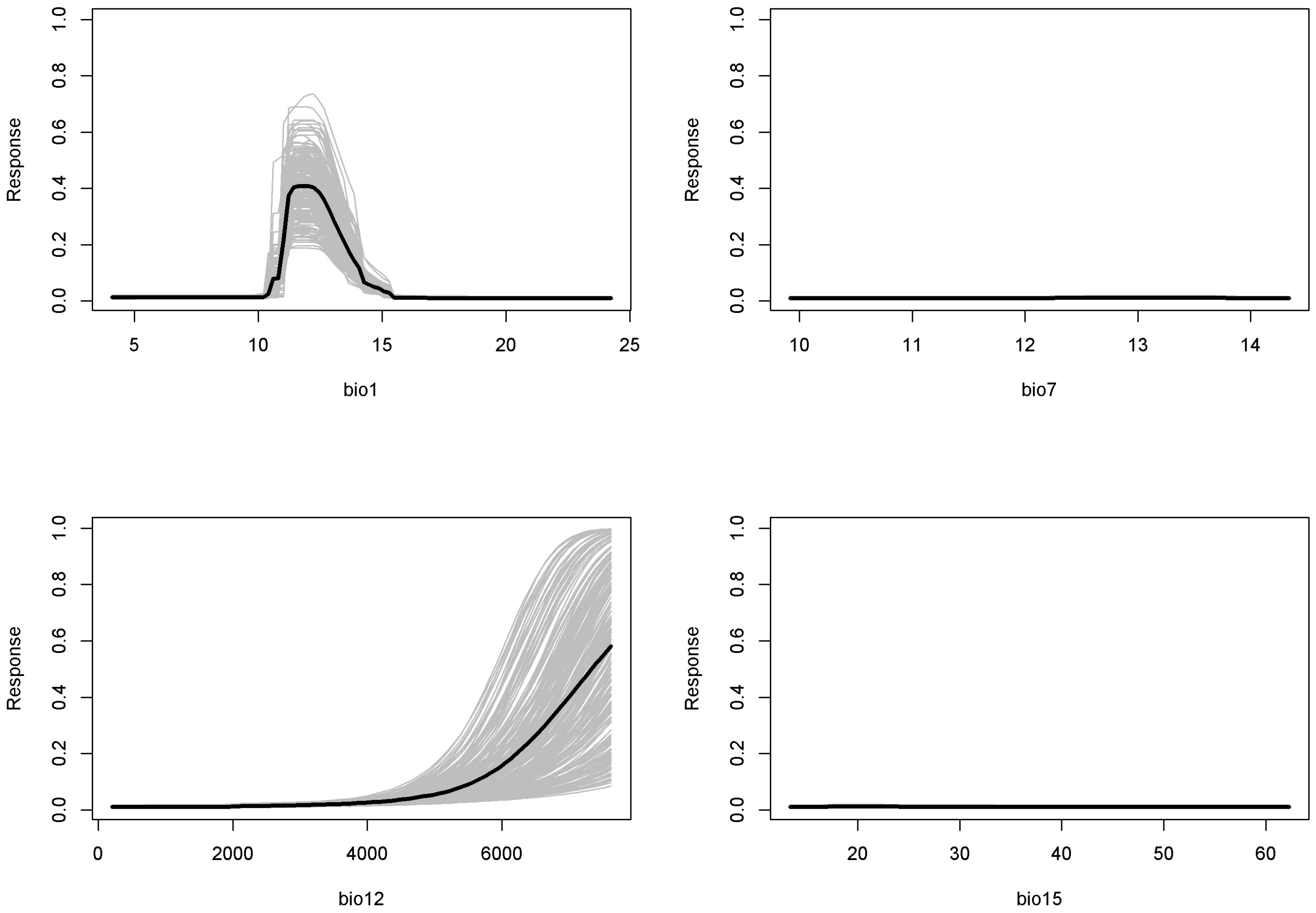

S3: SPECIES RESPONSE CURVES
HIGH MODEL RELIABILITY SPECIES

Hawaii.Creeper response curves GBM all vars

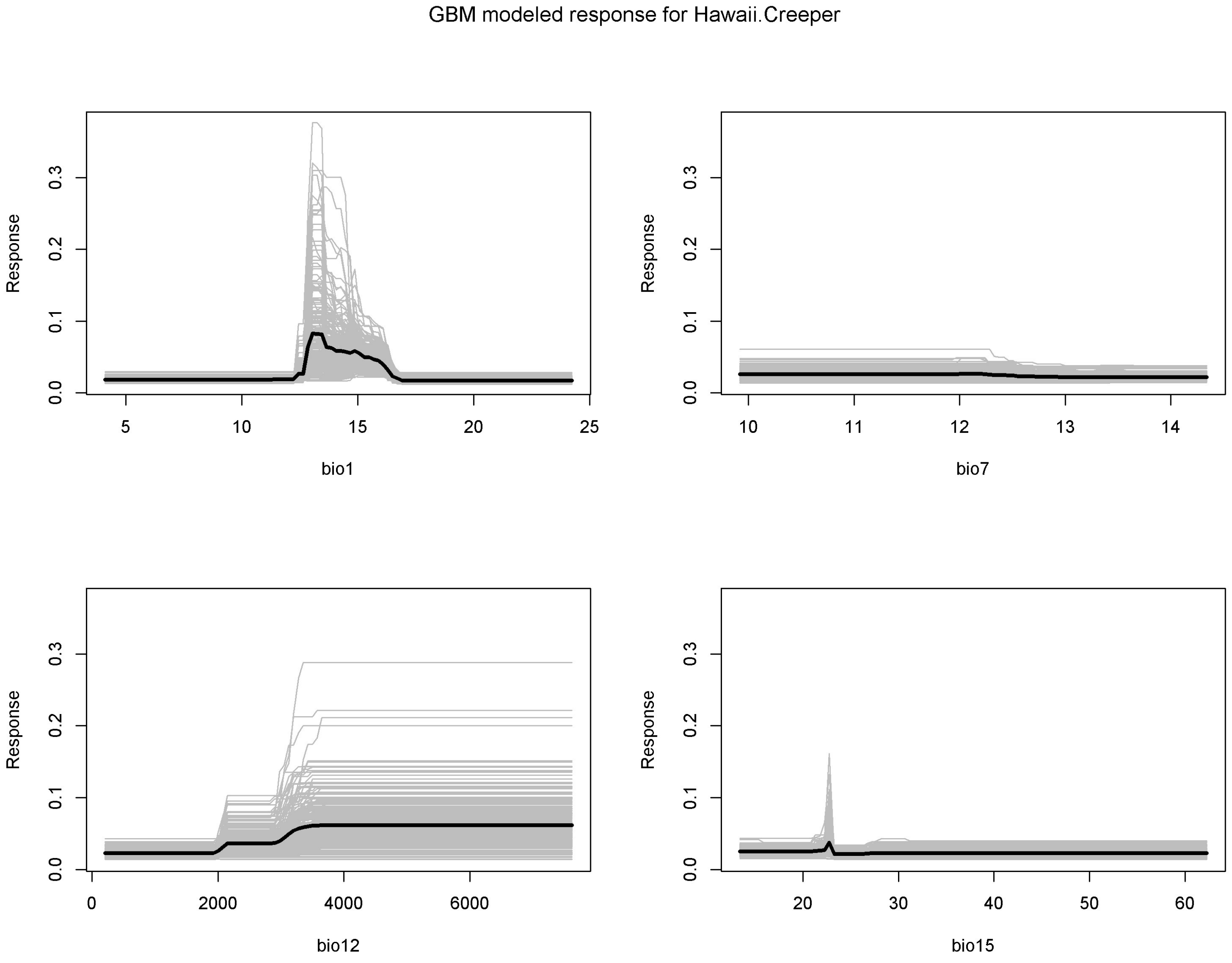

S3: SPECIES RESPONSE CURVES
HIGH MODEL RELIABILITY SPECIES

Hawaii.Creeper response curves MAXENT all vars

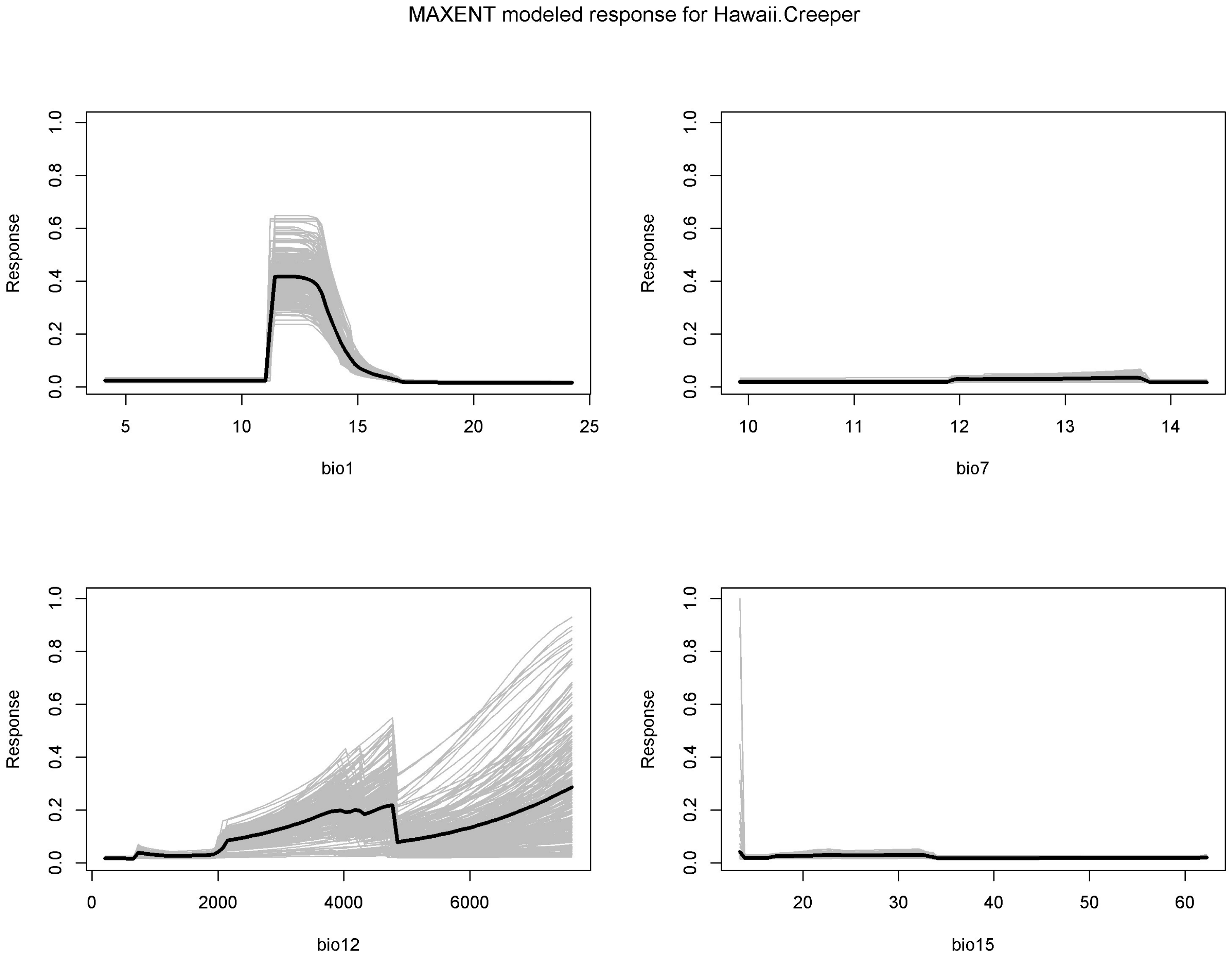

S3: SPECIES RESPONSE CURVES
HIGH MODEL RELIABILITY SPECIES

Iiwi response curves GBM all vars

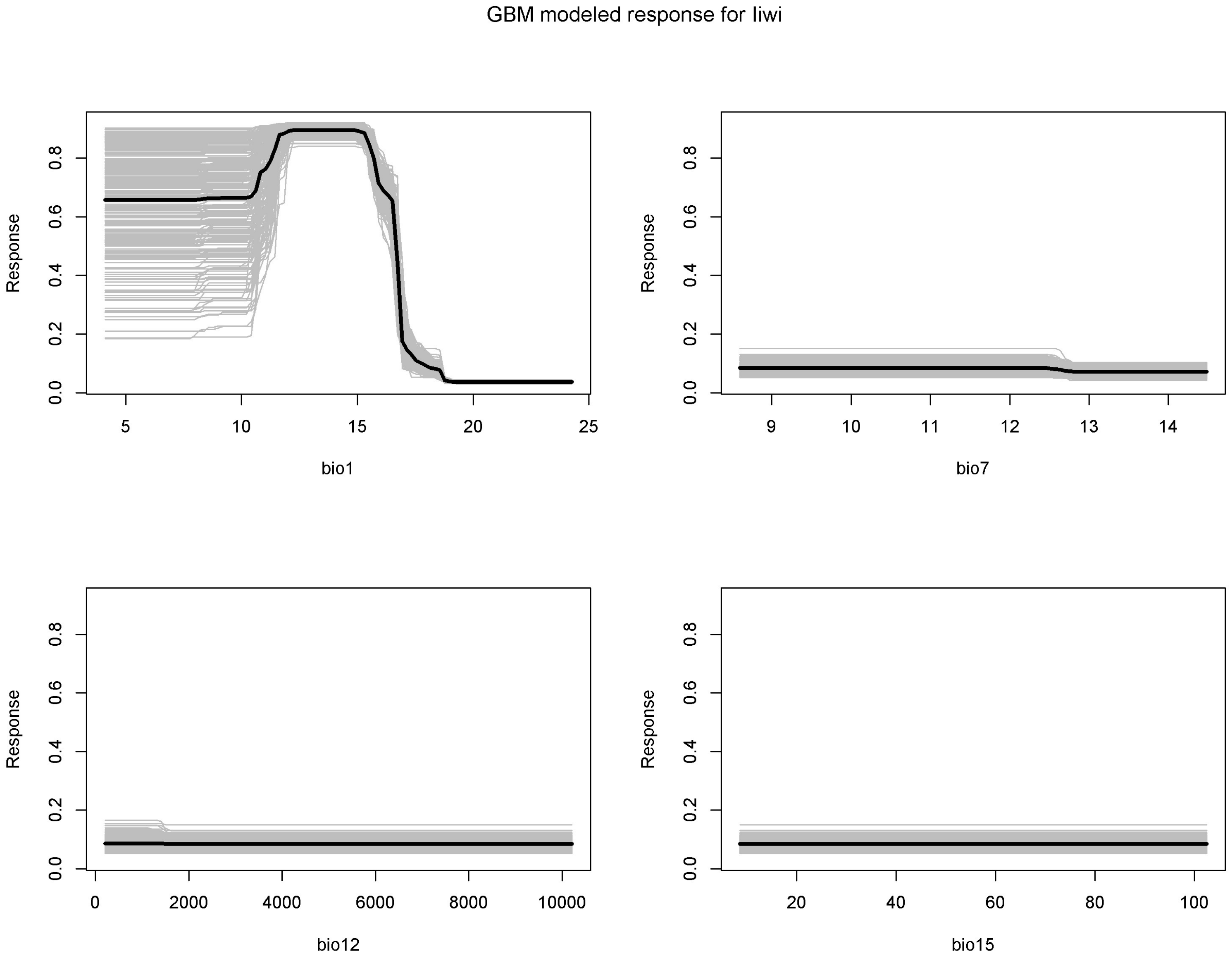

S3: SPECIES RESPONSE CURVES
HIGH MODEL RELIABILITY SPECIES

Iiwi response curves MAXENT all vars

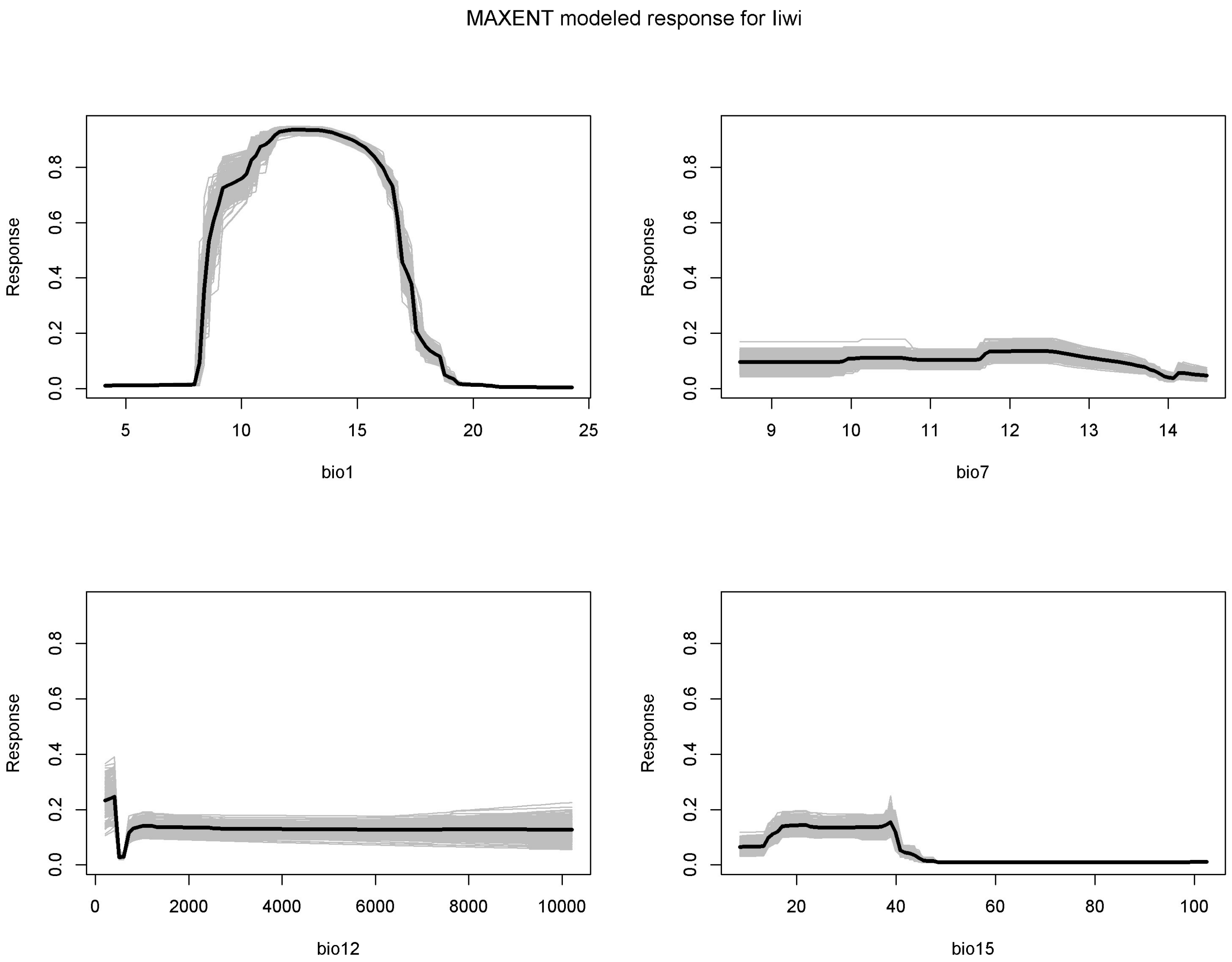

S3: SPECIES RESPONSE CURVES
HIGH MODEL RELIABILITY SPECIES

Maui.Alauahio response curves GBM all vars

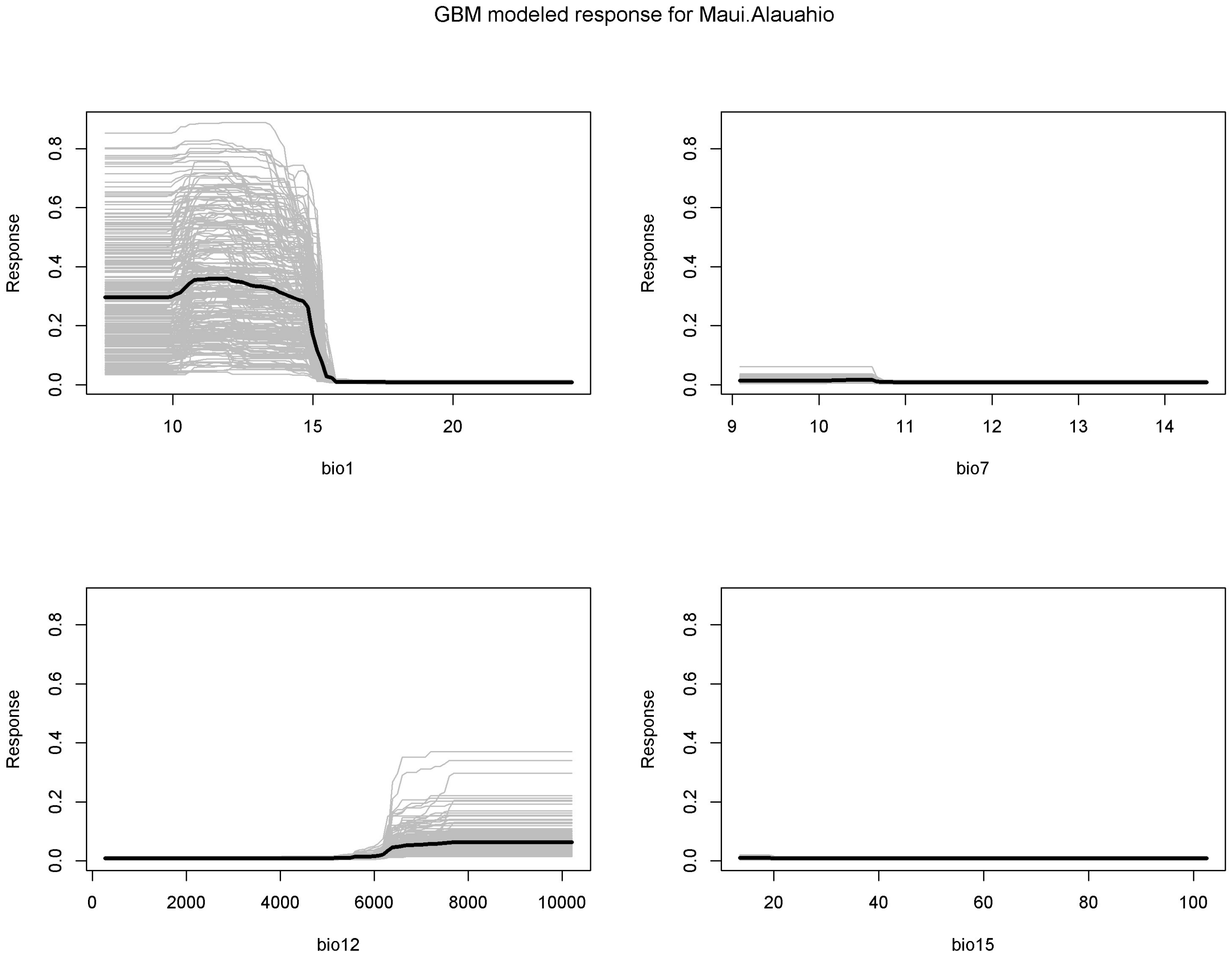

S3: SPECIES RESPONSE CURVES
HIGH MODEL RELIABILITY SPECIES

Maui.Alauahio response curves MAXENT all vars

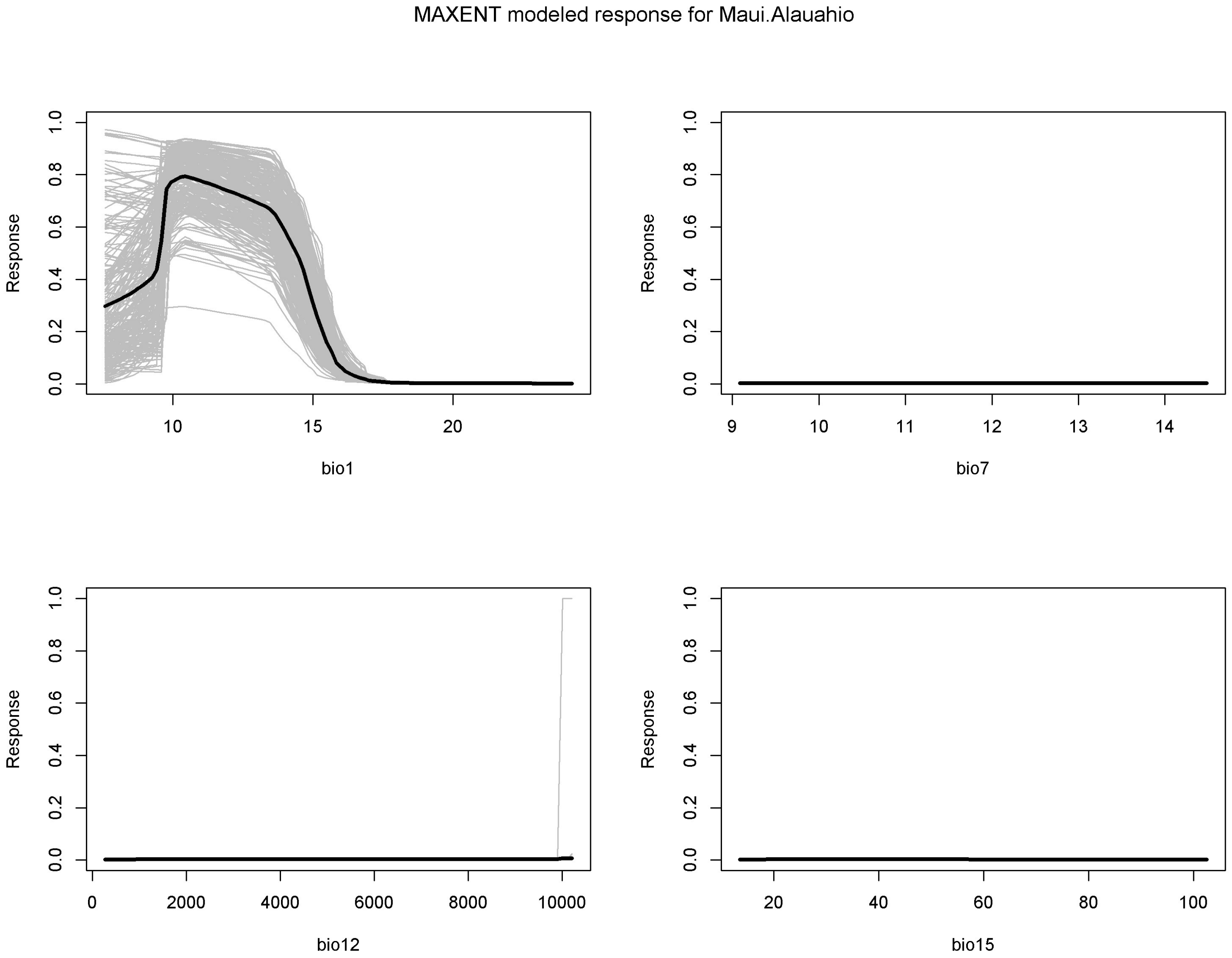

S3: SPECIES RESPONSE CURVES
HIGH MODEL RELIABILITY SPECIES

Maui.Parrotbill response curves GBM all vars

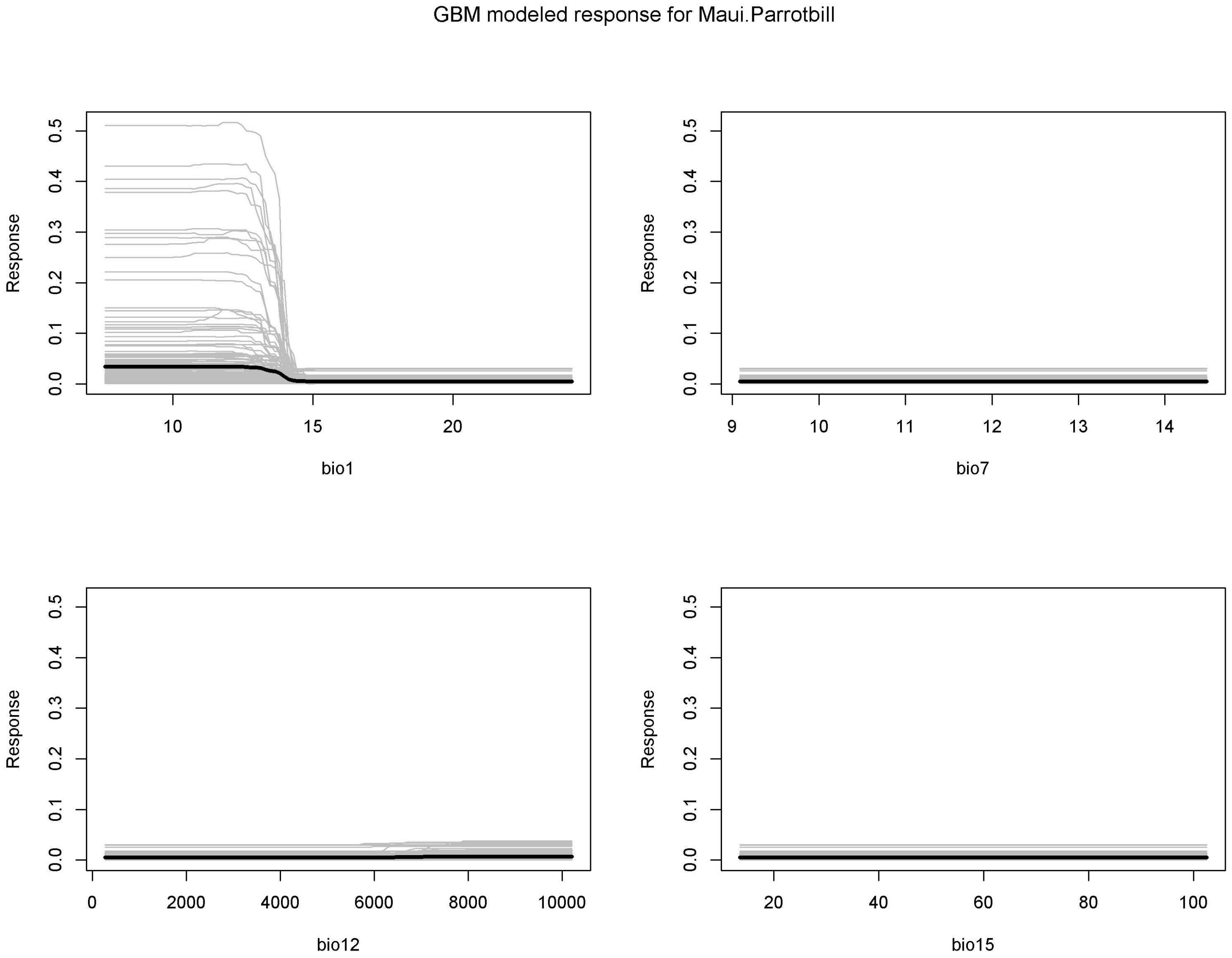

S3: SPECIES RESPONSE CURVES
HIGH MODEL RELIABILITY SPECIES

Maui.Parrotbill response curves MAXENT all vars

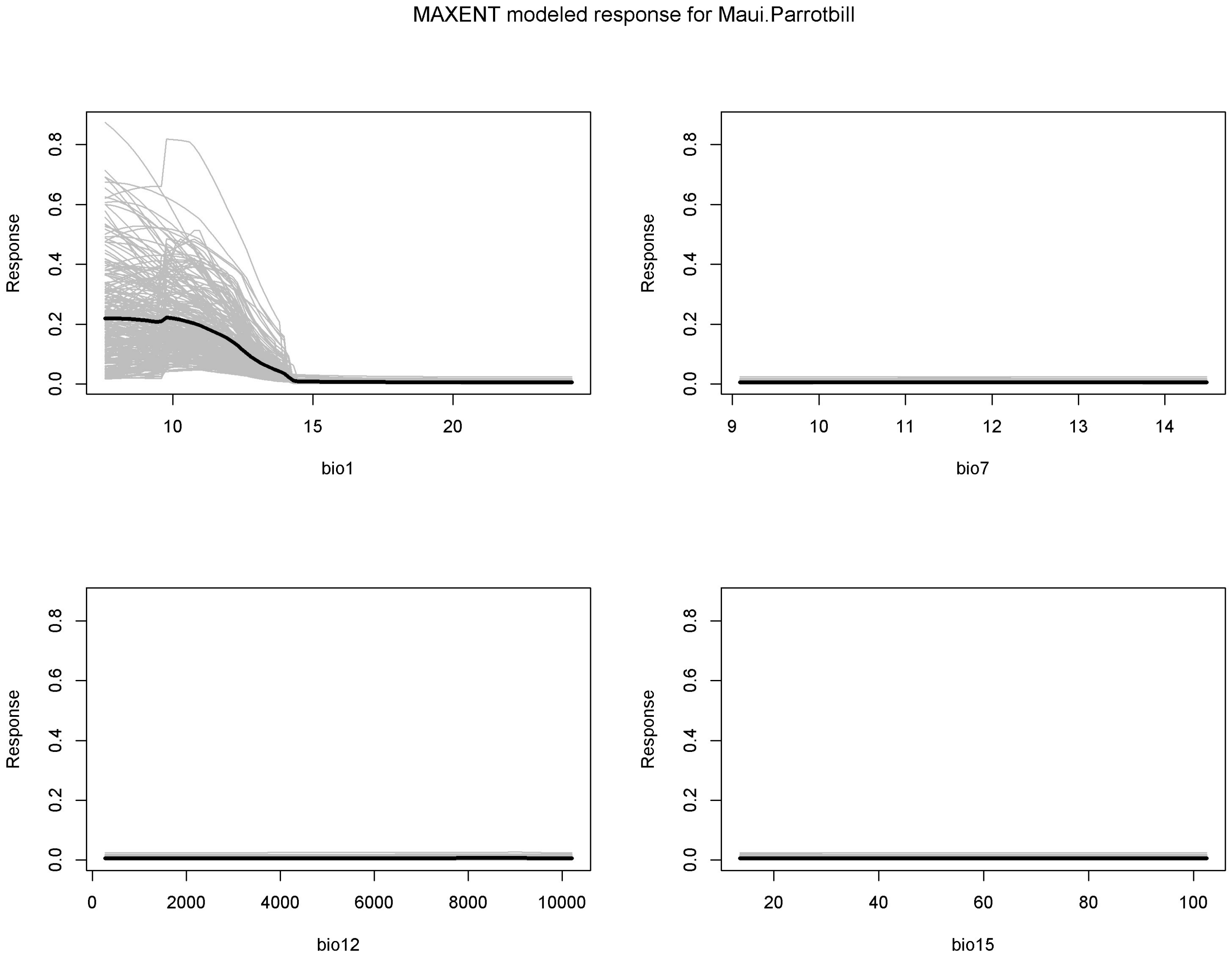

S3: SPECIES RESPONSE CURVES
HIGH MODEL RELIABILITY SPECIES

Puaiohi response curves GBM all vars

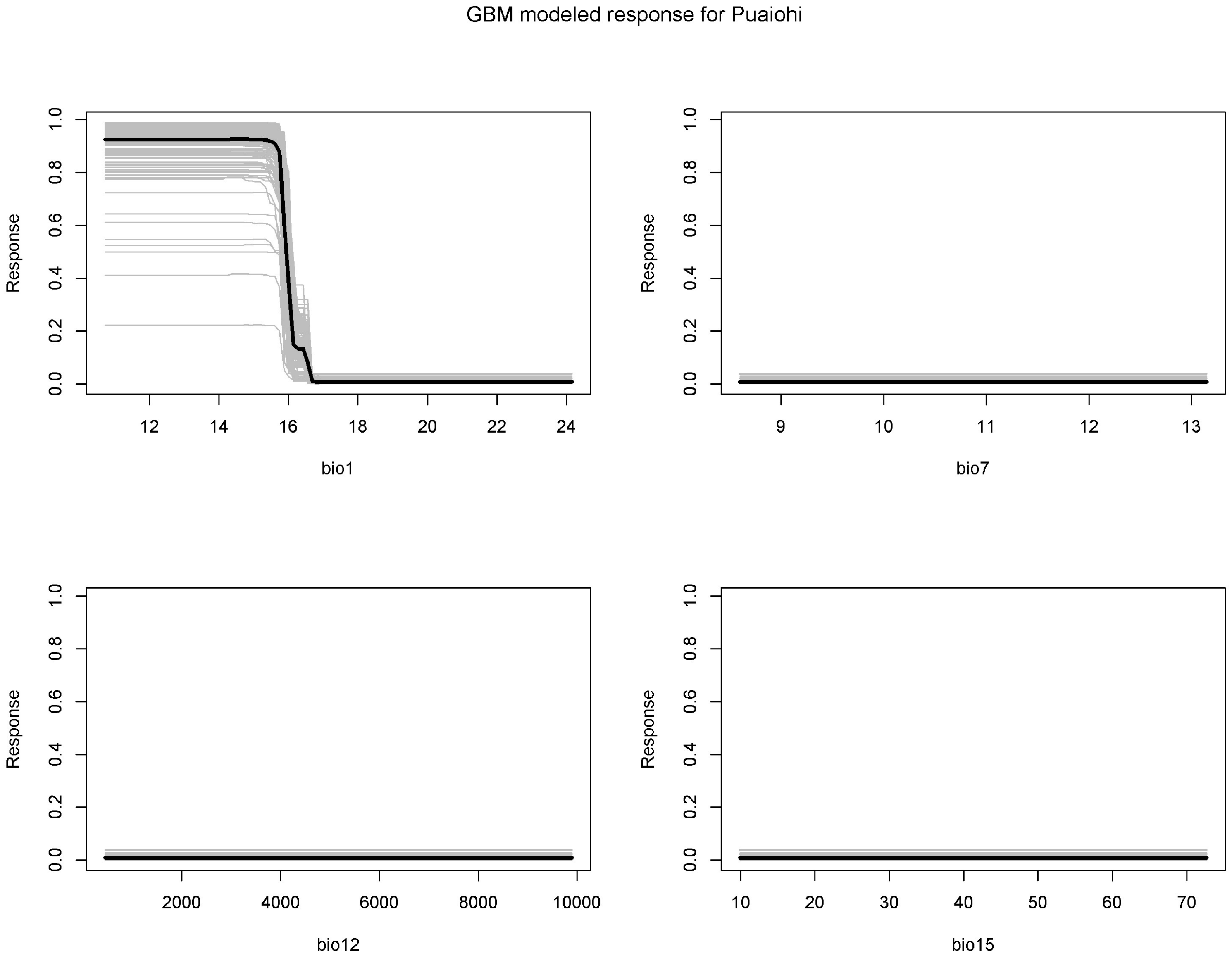

S3: SPECIES RESPONSE CURVES
HIGH MODEL RELIABILITY SPECIES

Puaiohi response curves MAXENT all vars

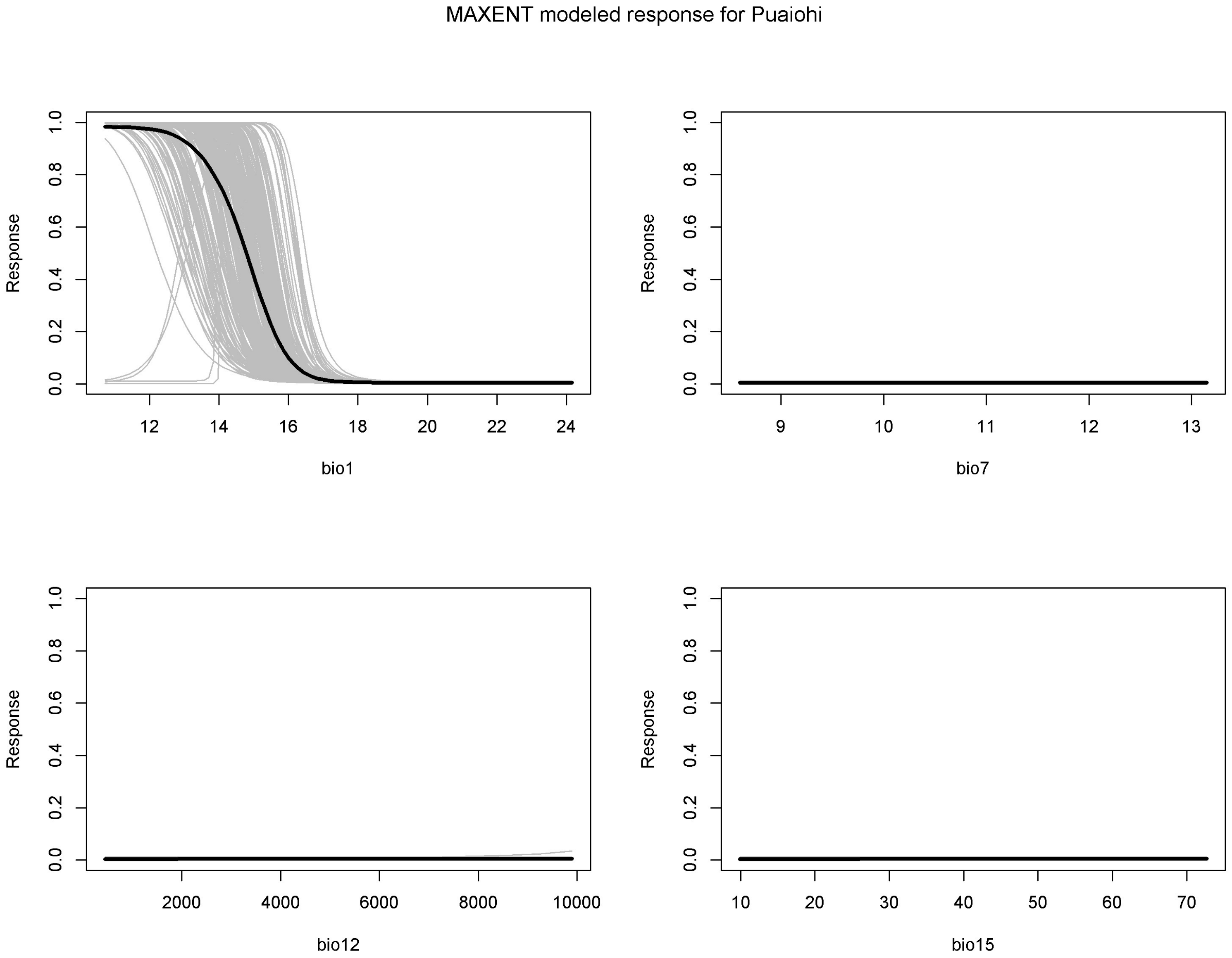

S3: SPECIES RESPONSE CURVES
REDUCED MODEL RELIABILITY SPECIES

Anianiau response curves GBM all vars

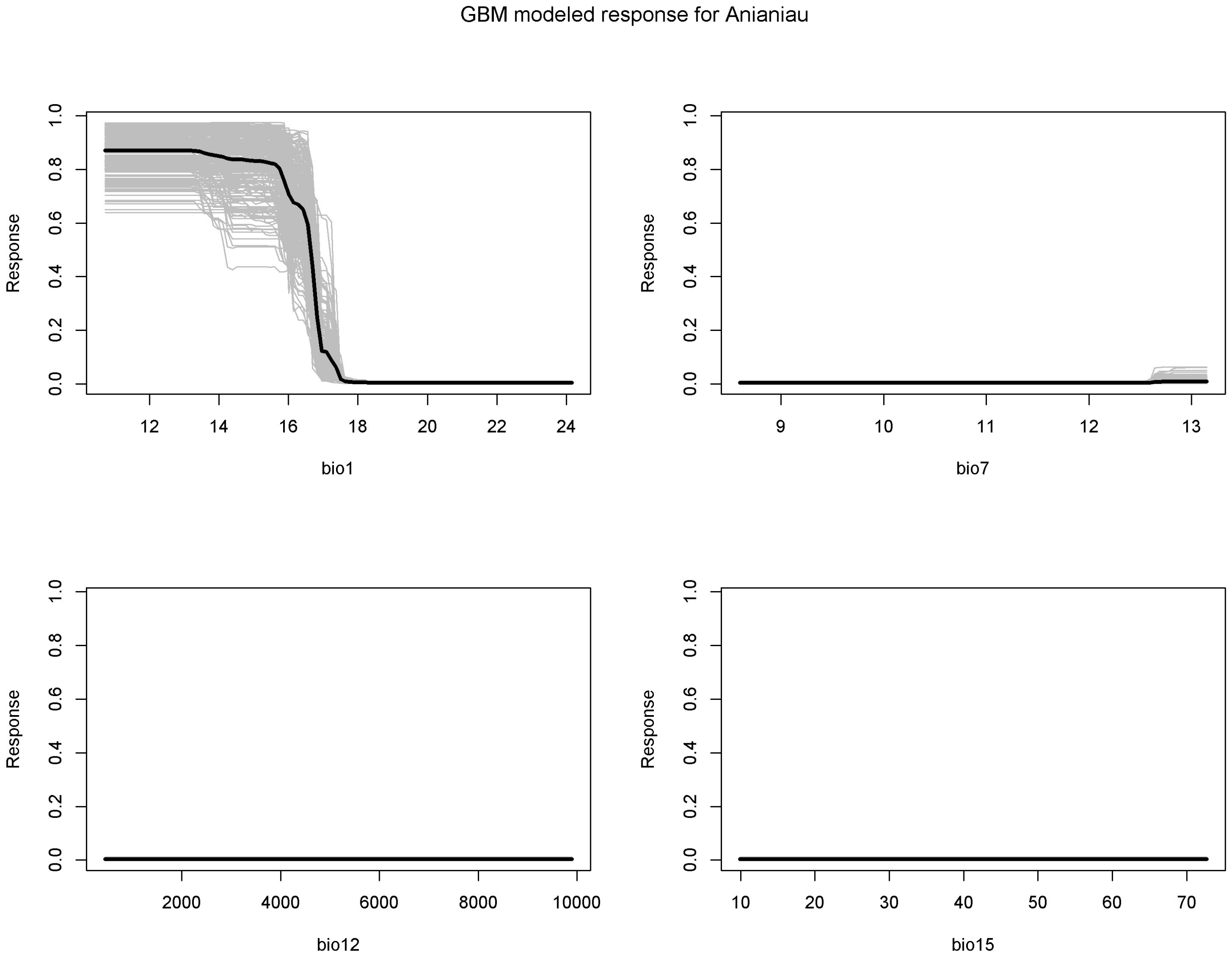

S3: SPECIES RESPONSE CURVES
REDUCED MODEL RELIABILITY SPECIES

Anianiau response curves MAXENT all vars

MAXENT modeled response for Anianiau

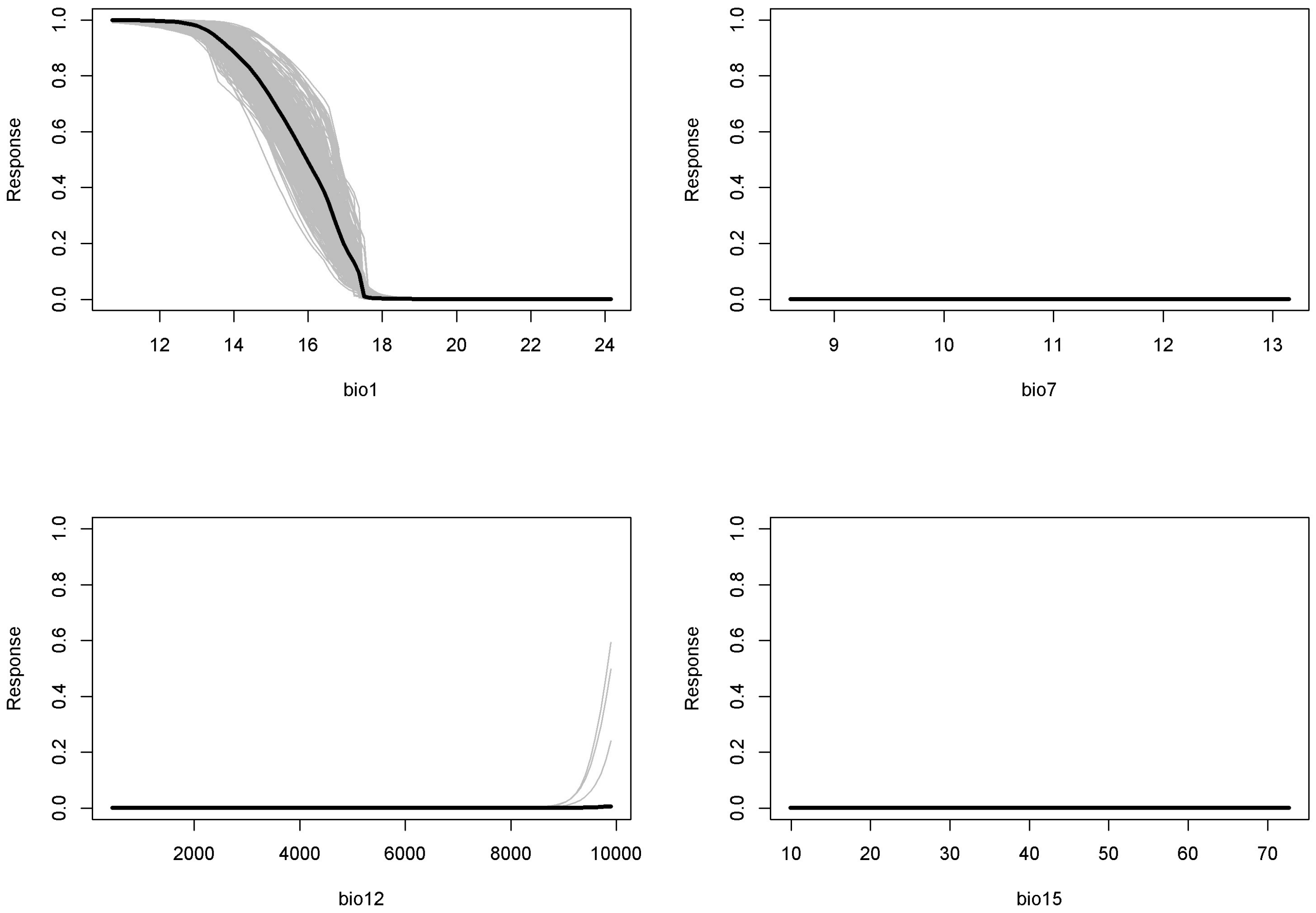

S3: SPECIES RESPONSE CURVES
REDUCED MODEL RELIABILITY SPECIES

Apapane response curves GBM all vars

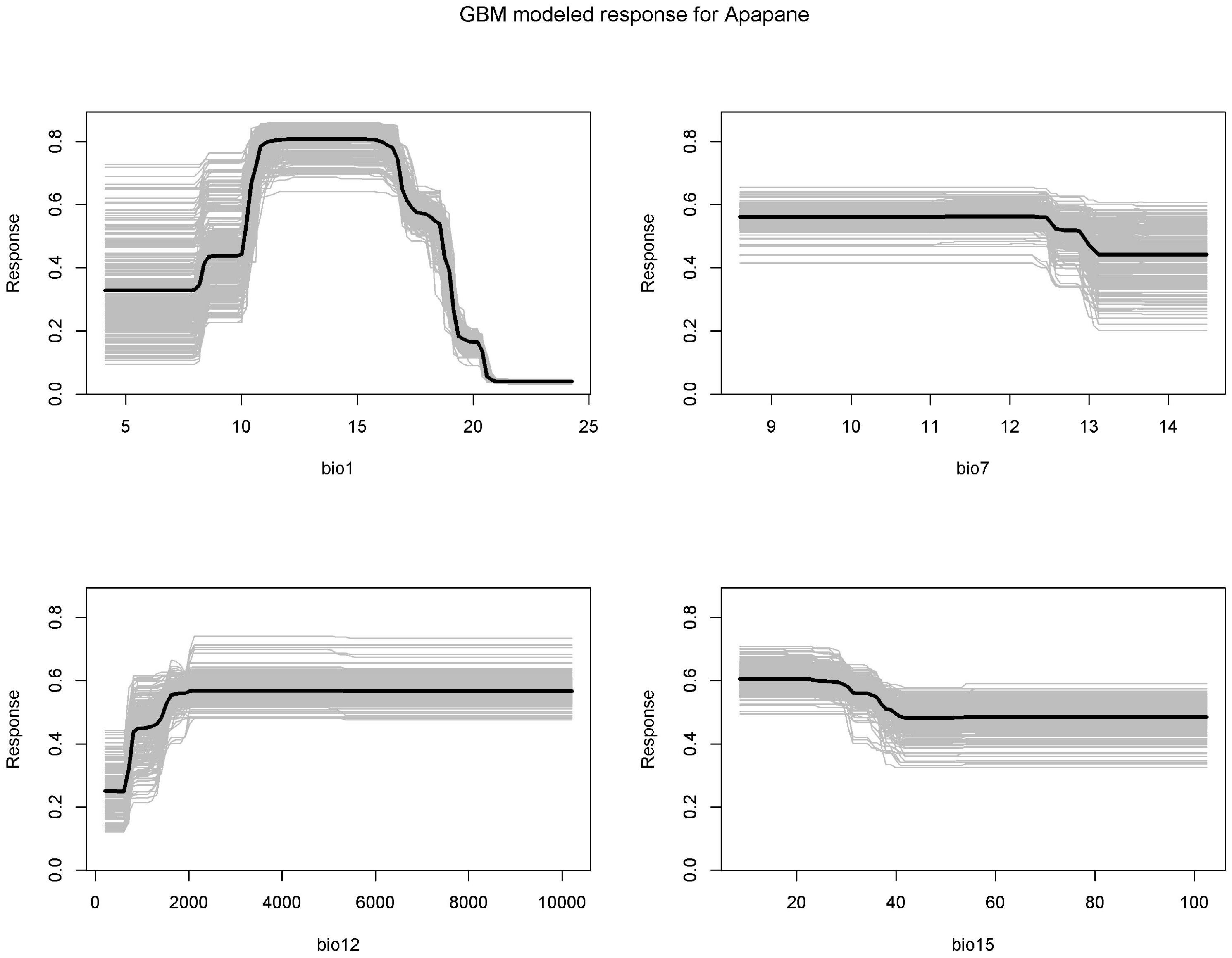

S3: SPECIES RESPONSE CURVES
REDUCED MODEL RELIABILITY SPECIES

Apapane response curves MAXENT all vars

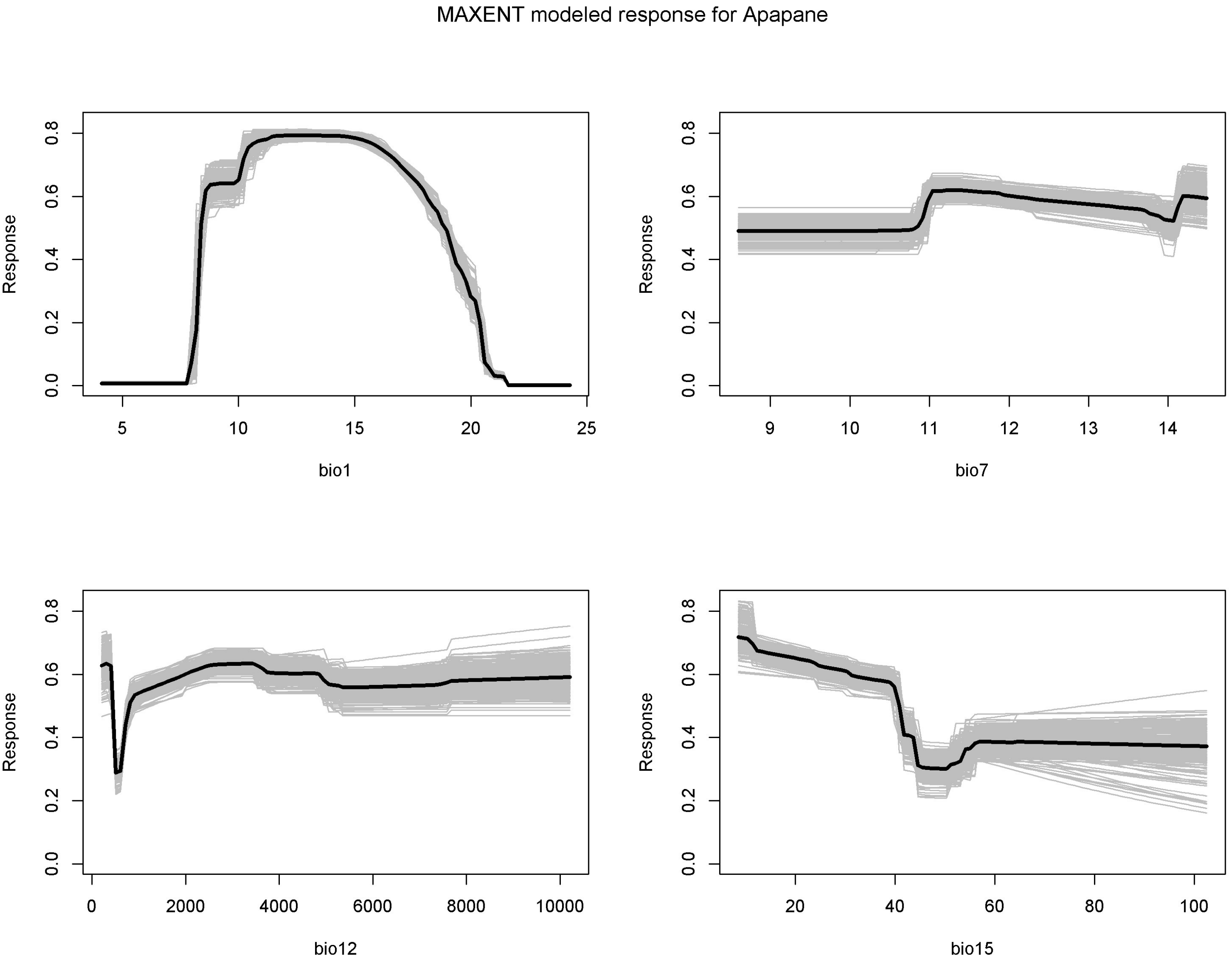

S3: SPECIES RESPONSE CURVES
REDUCED MODEL RELIABILITY SPECIES

Hawaii.Amakihi response curves GBM all vars

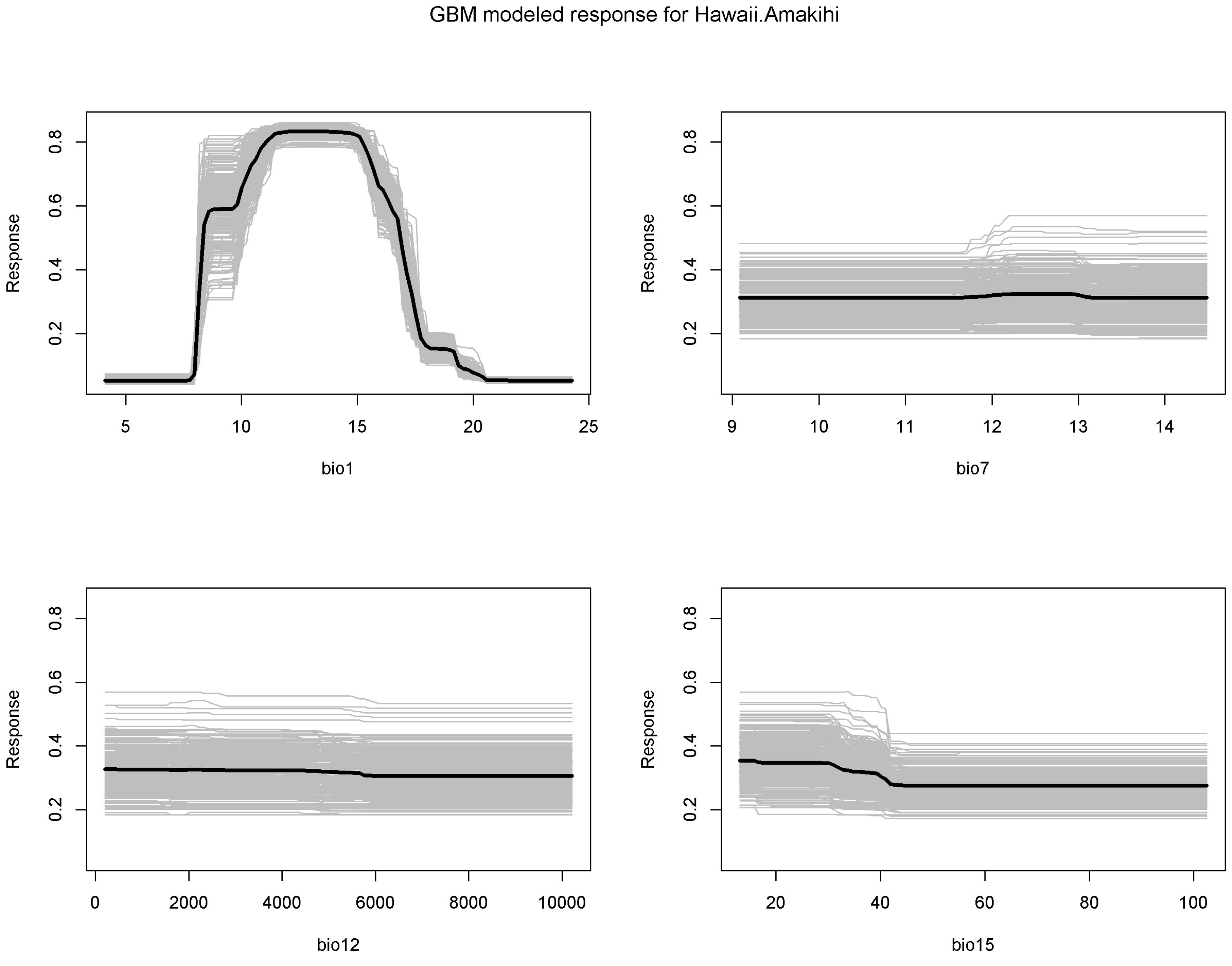

S3: SPECIES RESPONSE CURVES
REDUCED MODEL RELIABILITY SPECIES

Hawaii.Amakihi response curves MAXENT all vars

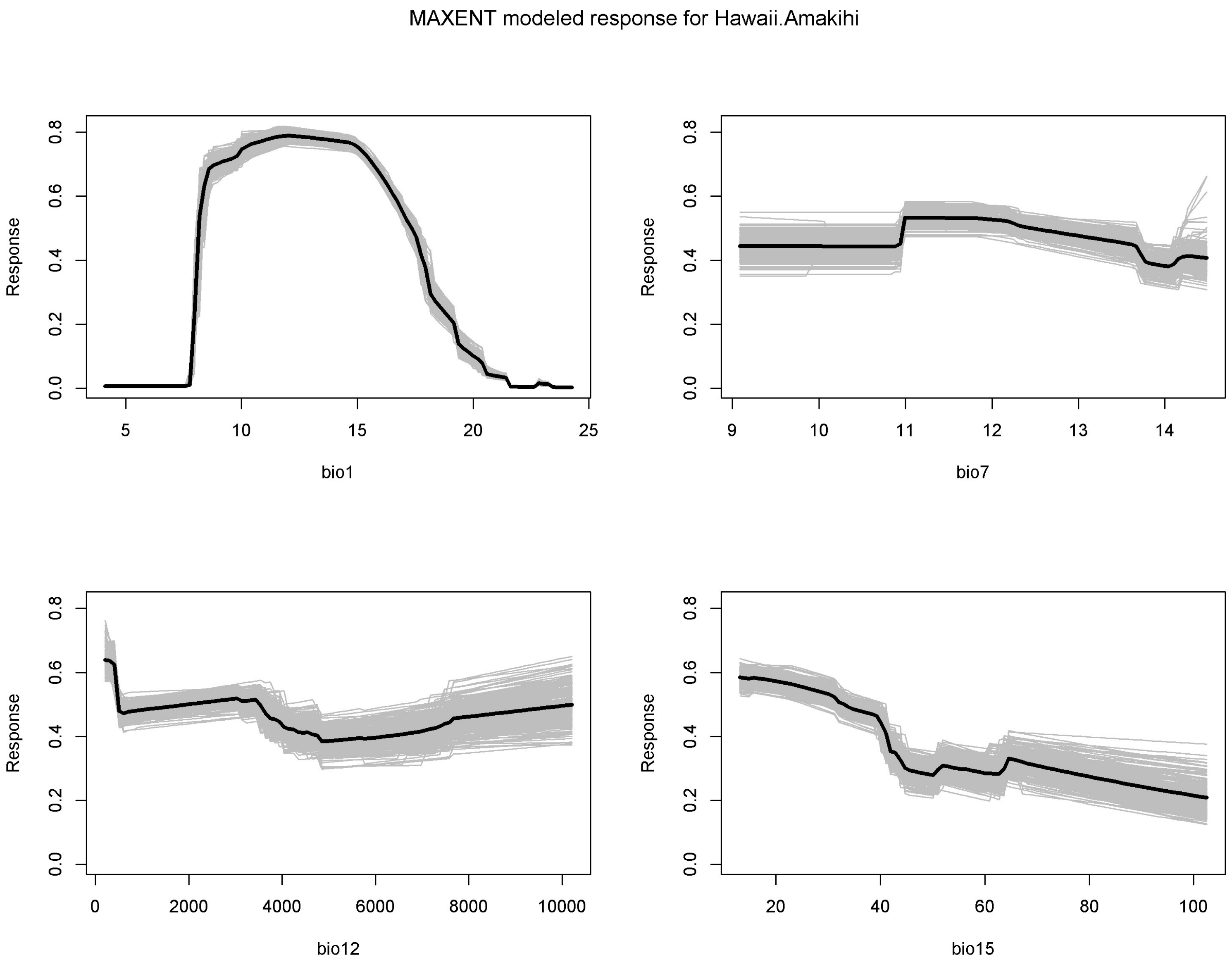

S3: SPECIES RESPONSE CURVES
REDUCED MODEL RELIABILITY SPECIES

Hawaii.Elepaio response curves GBM all vars

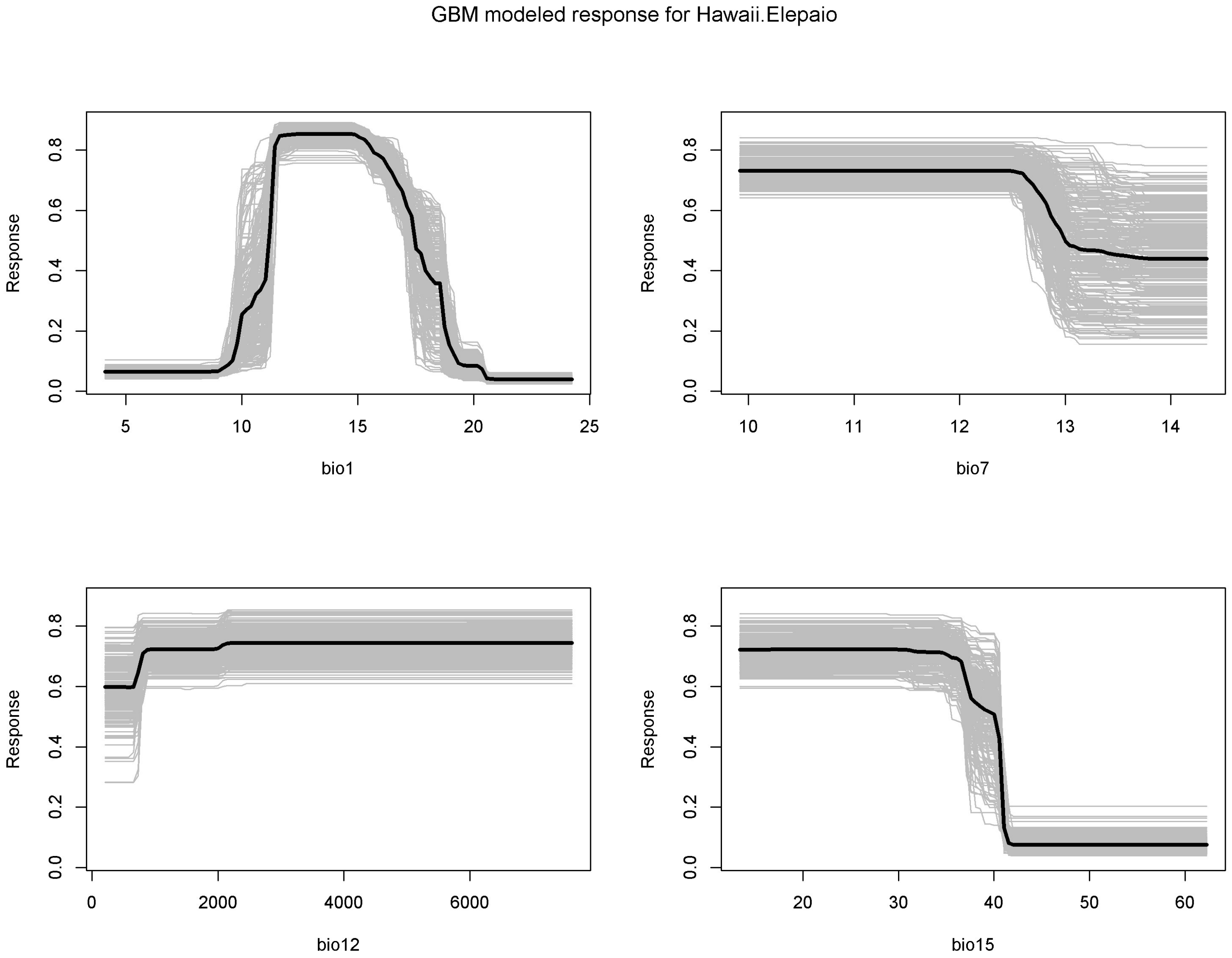

S3: SPECIES RESPONSE CURVES
REDUCED MODEL RELIABILITY SPECIES

Hawaii.Elepaio response curves MAXENT all vars

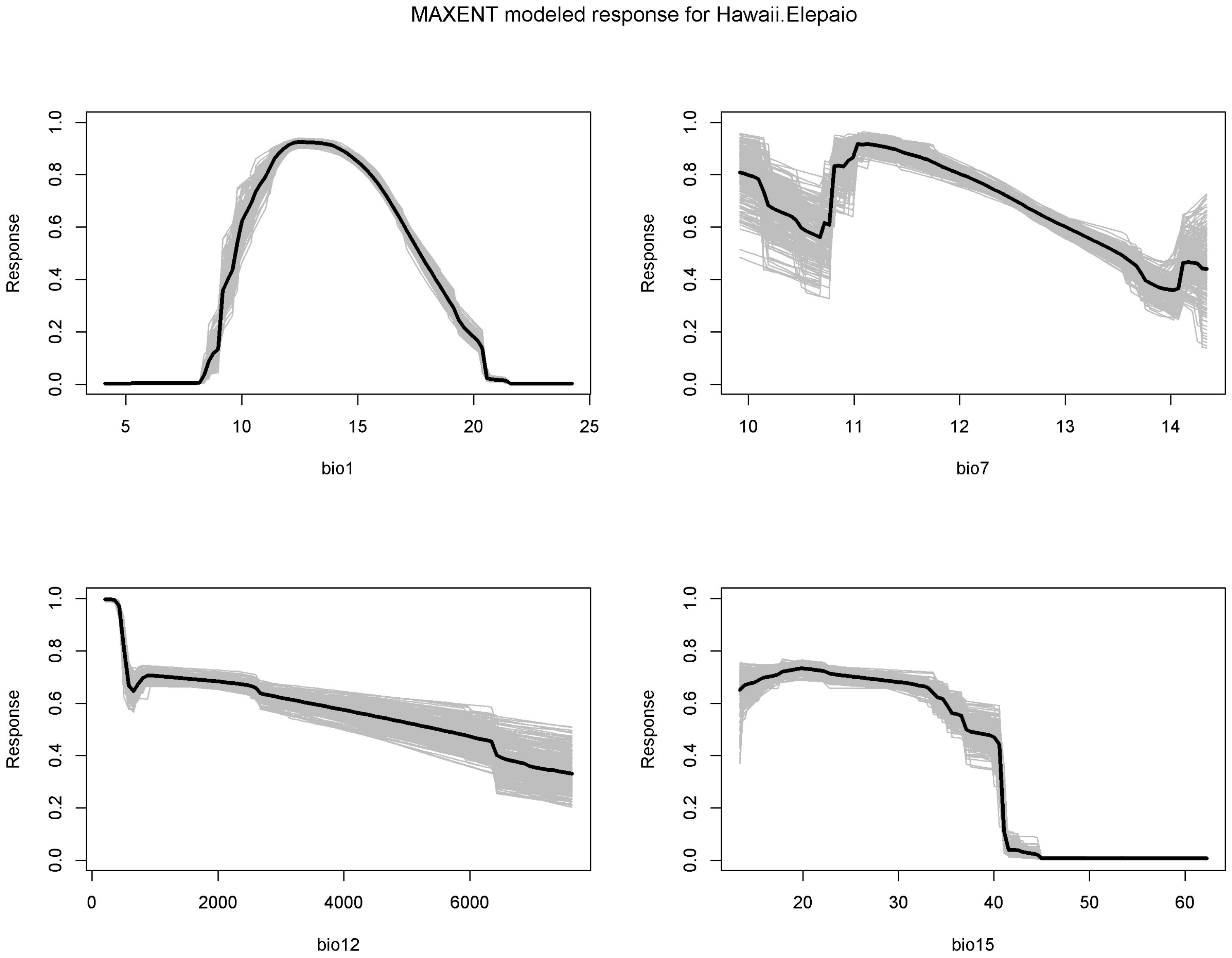

S3: SPECIES RESPONSE CURVES
REDUCED MODEL RELIABILITY SPECIES

Kauai.Amakihi response curves GBM all vars

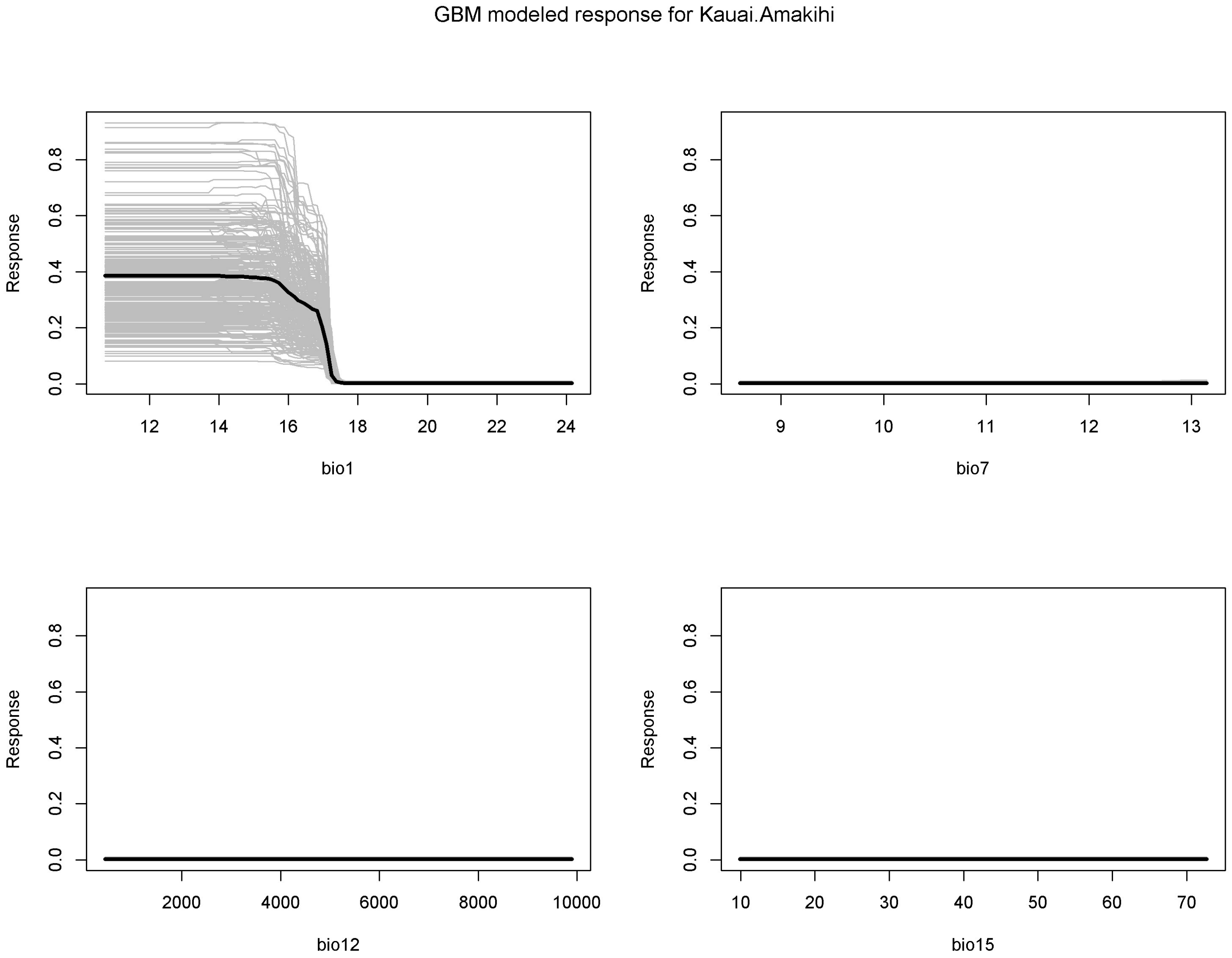

S3: SPECIES RESPONSE CURVES
REDUCED MODEL RELIABILITY SPECIES

Kauai.Amakihi response curves MAXENT all vars

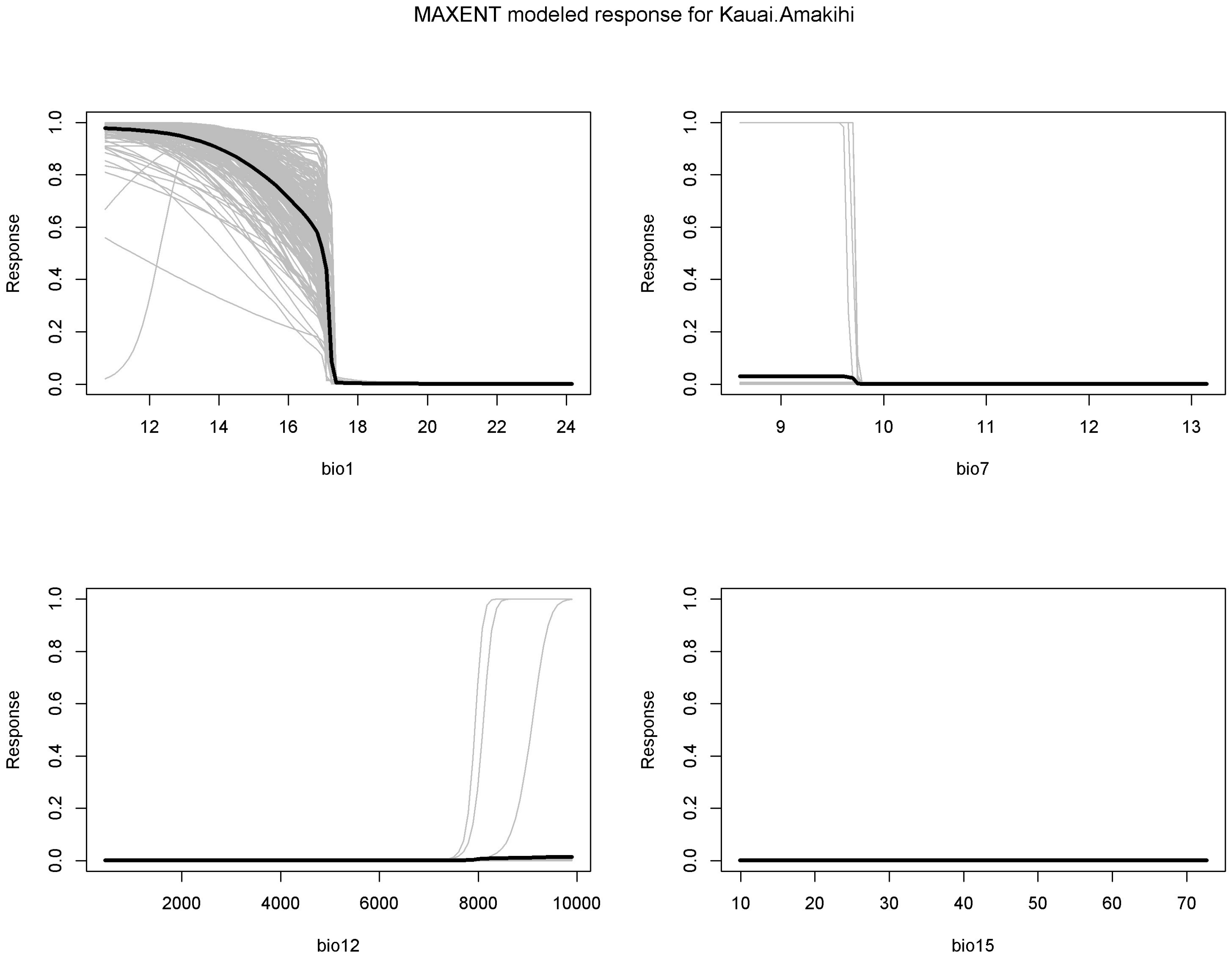

S3: SPECIES RESPONSE CURVES
REDUCED MODEL RELIABILITY SPECIES

Kauai.Elepaio response curves GBM all vars

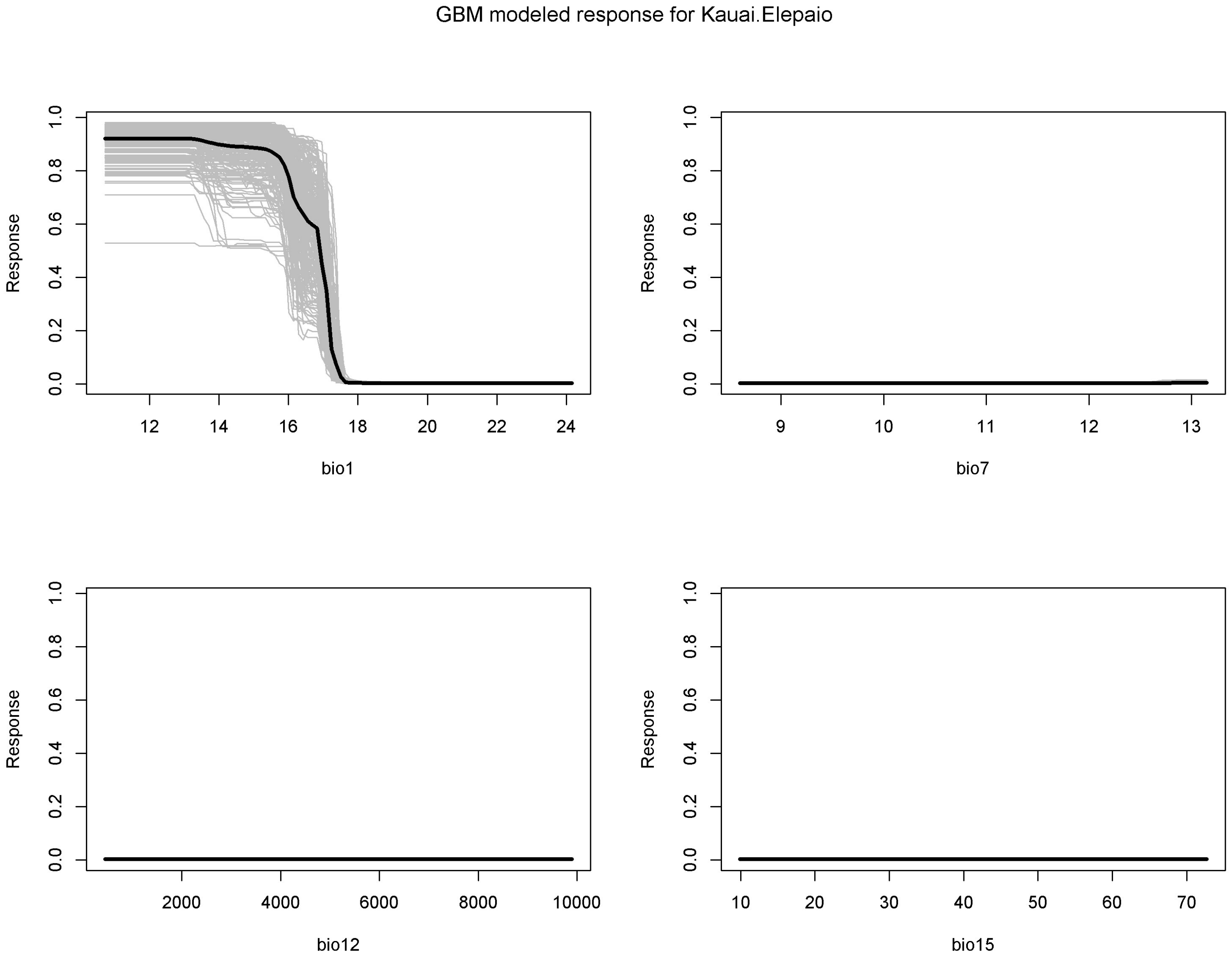

S3: SPECIES RESPONSE CURVES
REDUCED MODEL RELIABILITY SPECIES

Kauai.Elepaio response curves MAXENT all vars

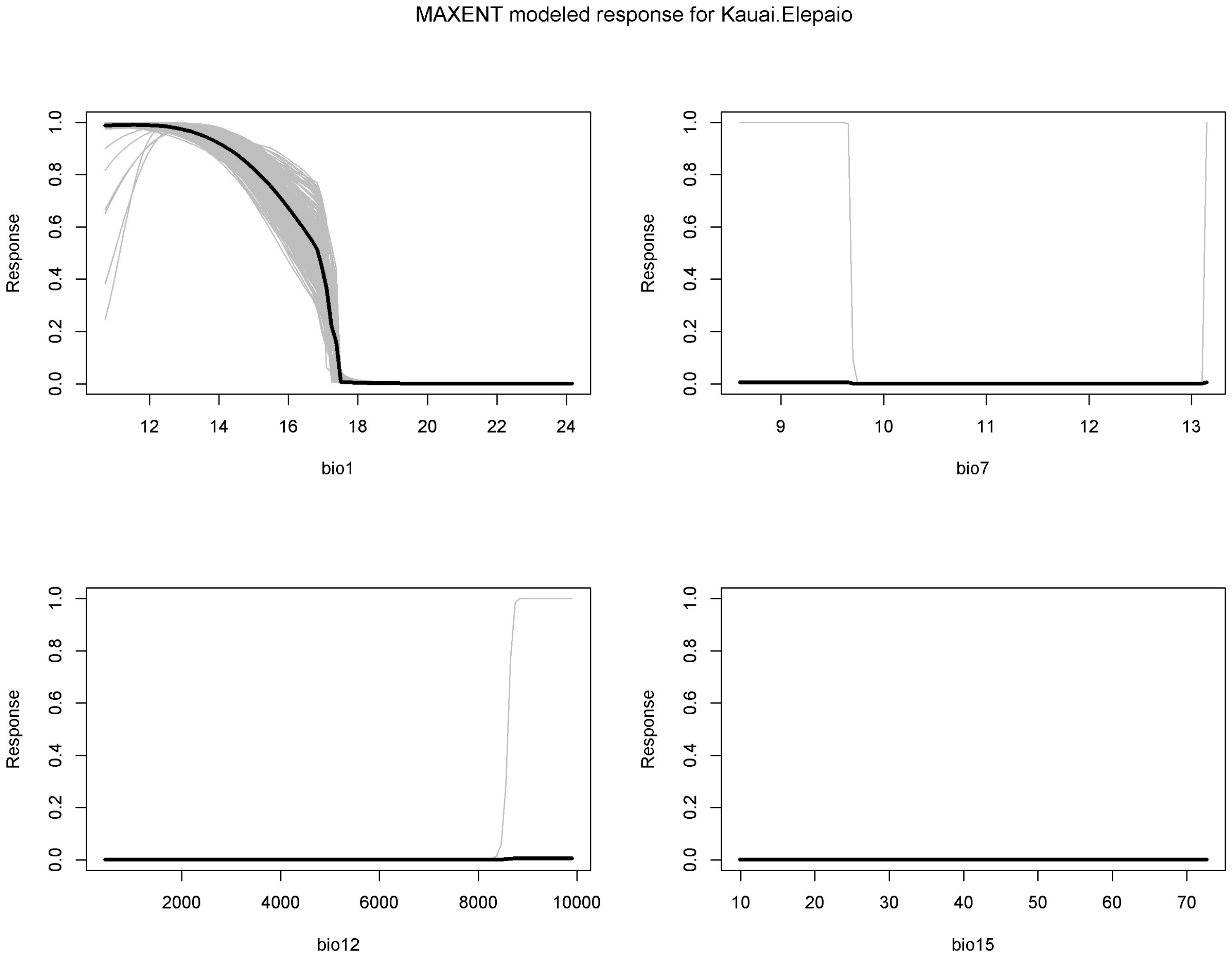

S3: SPECIES RESPONSE CURVES
REDUCED MODEL RELIABILITY SPECIES

Oahu.Amakihi response curves GBM all vars

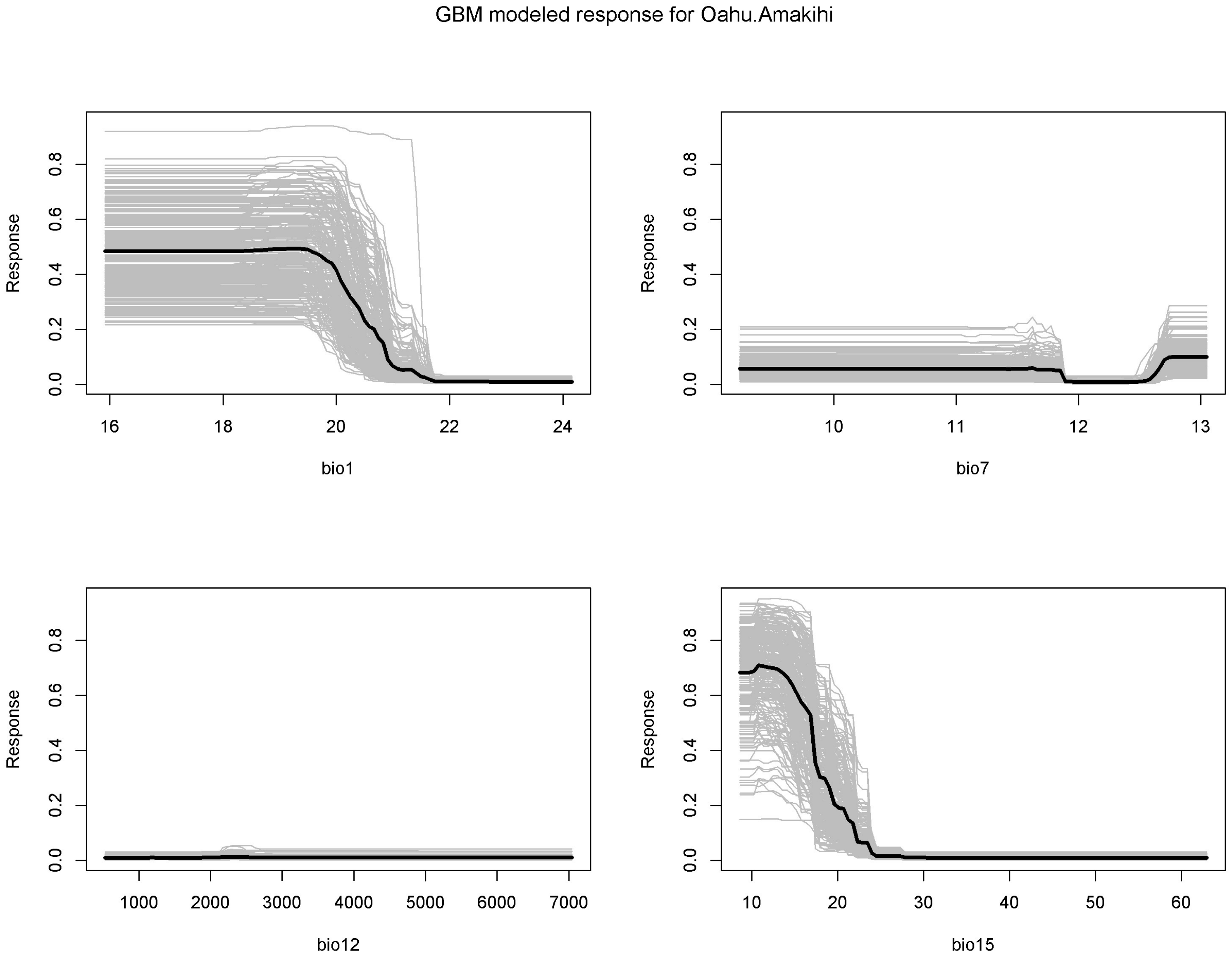

S3: SPECIES RESPONSE CURVES
REDUCED MODEL RELIABILITY SPECIES

Oahu.Amakihi response curves MAXENT all vars

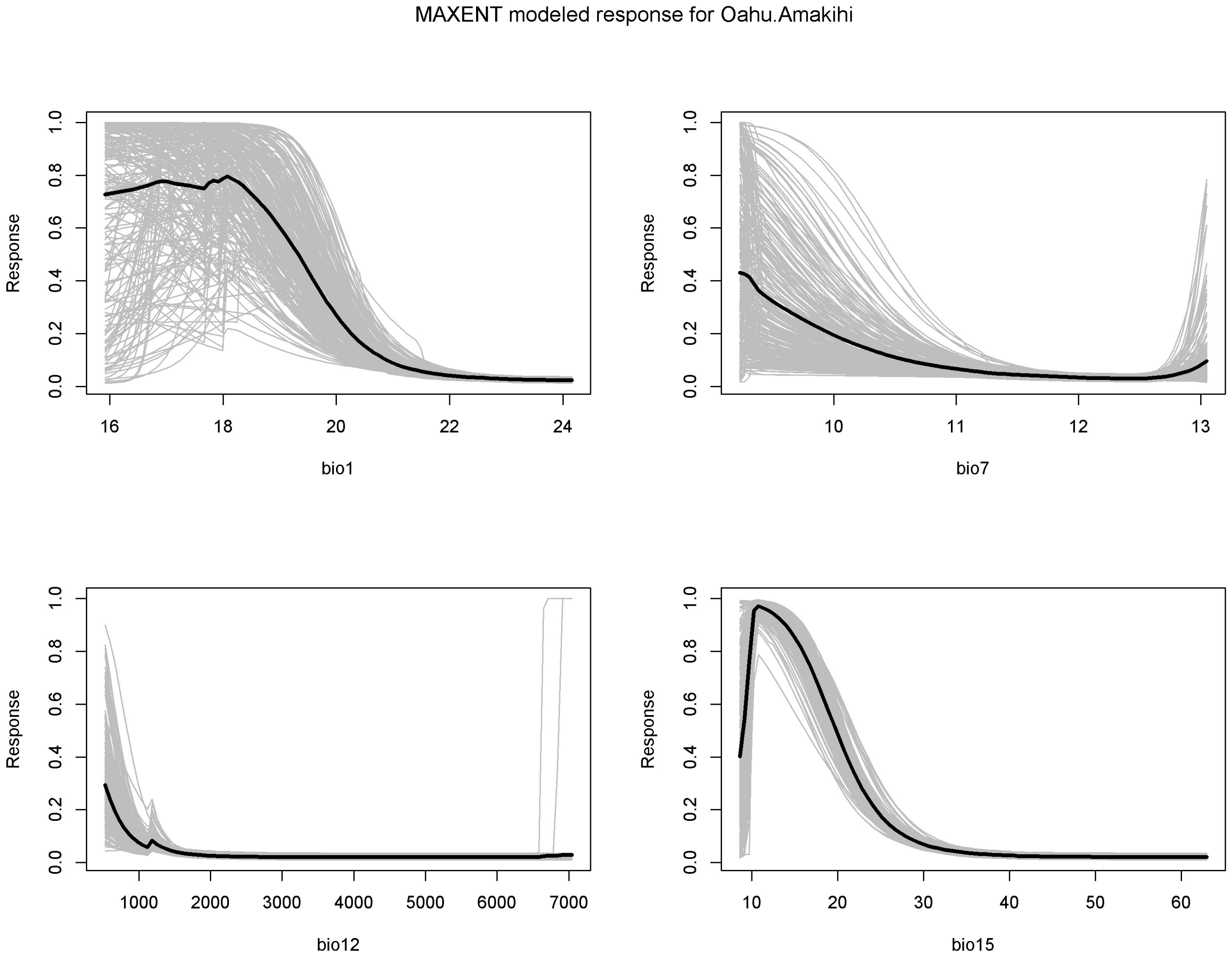

S3: SPECIES RESPONSE CURVES
REDUCED MODEL RELIABILITY SPECIES

Oahu.Elepaio response curves GBM all vars

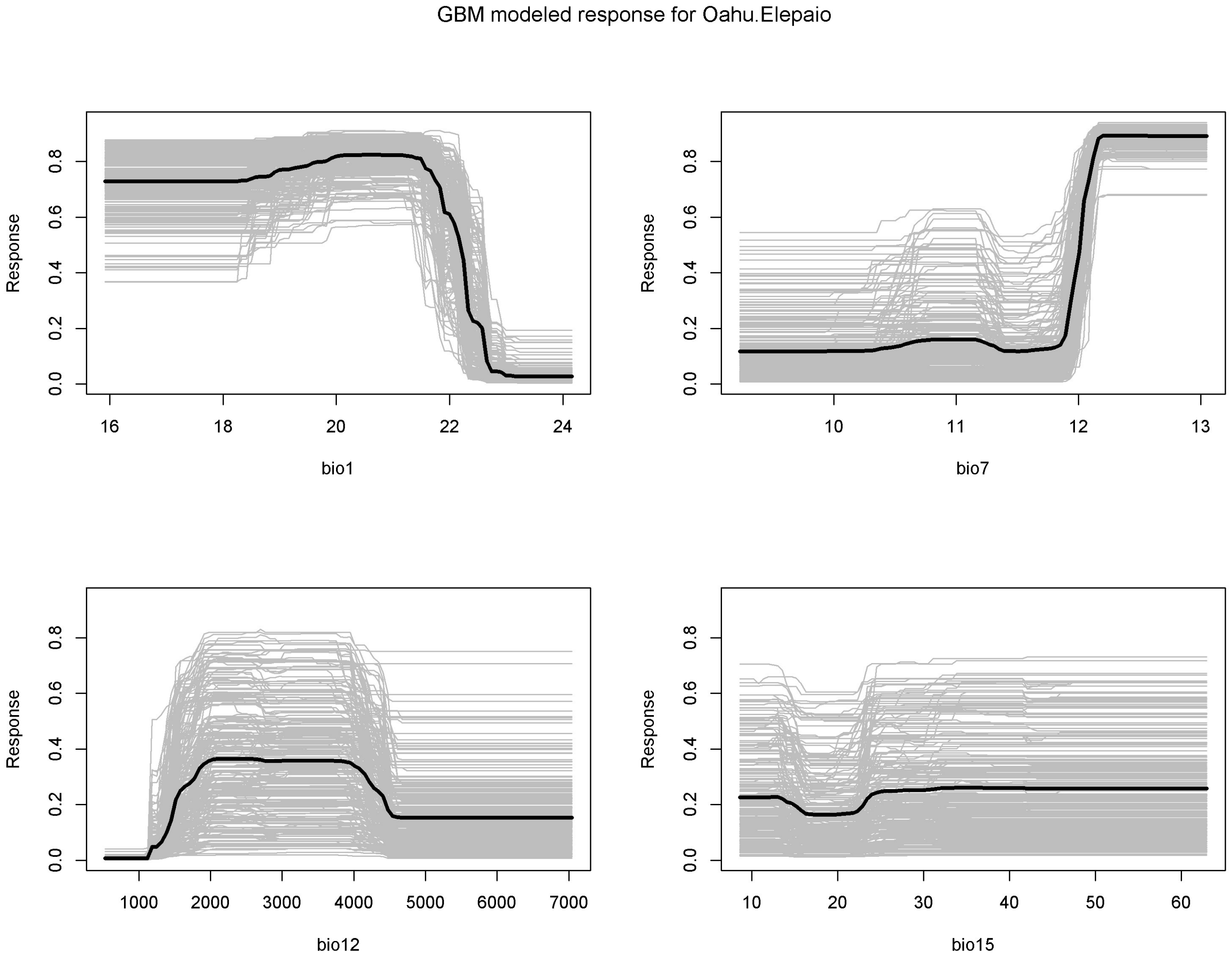

S3: SPECIES RESPONSE CURVES
REDUCED MODEL RELIABILITY SPECIES

Oahu.Elepaio response curves MAXENT all vars

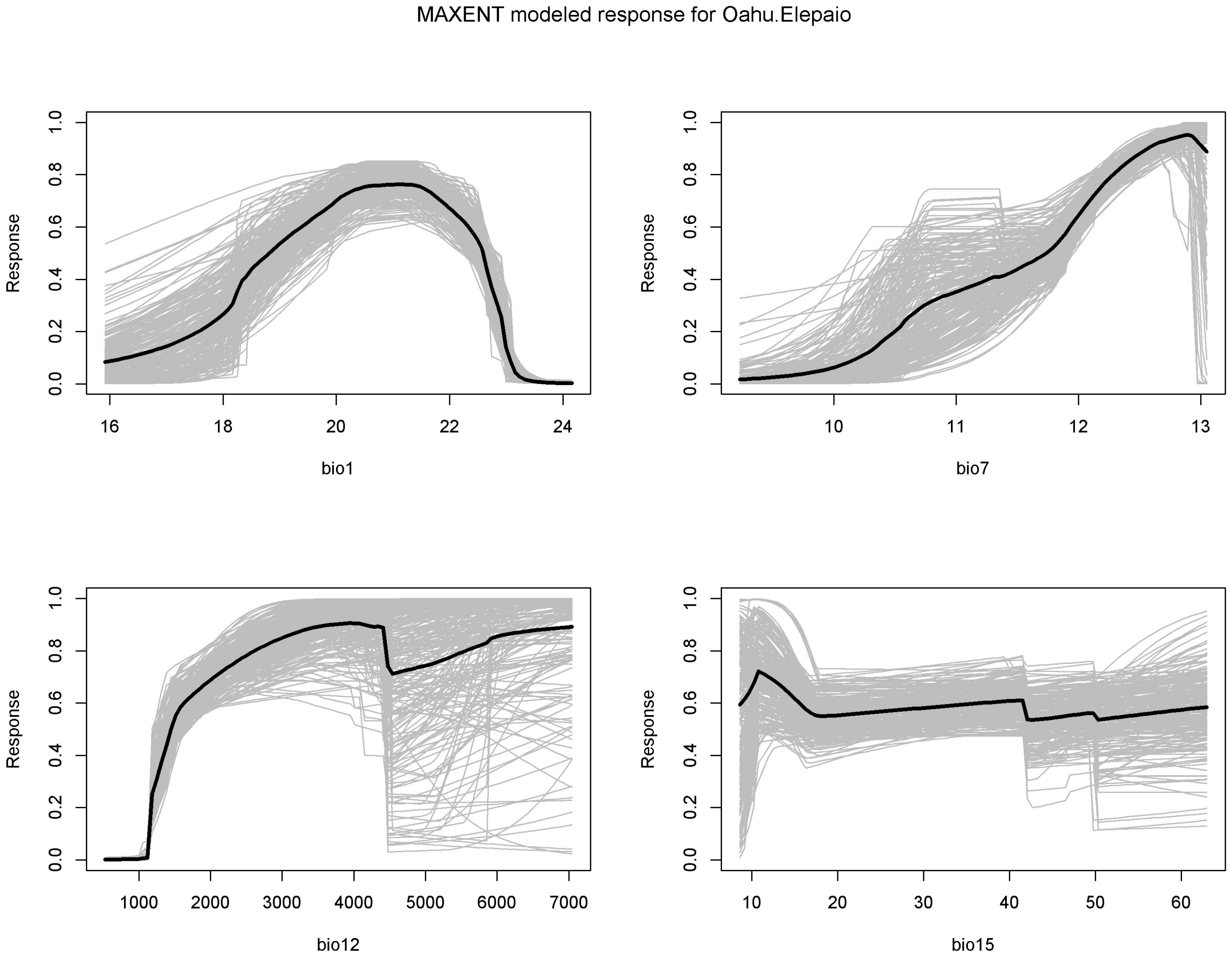

S3: SPECIES RESPONSE CURVES
REDUCED MODEL RELIABILITY SPECIES

Omao response curves GBM all vars

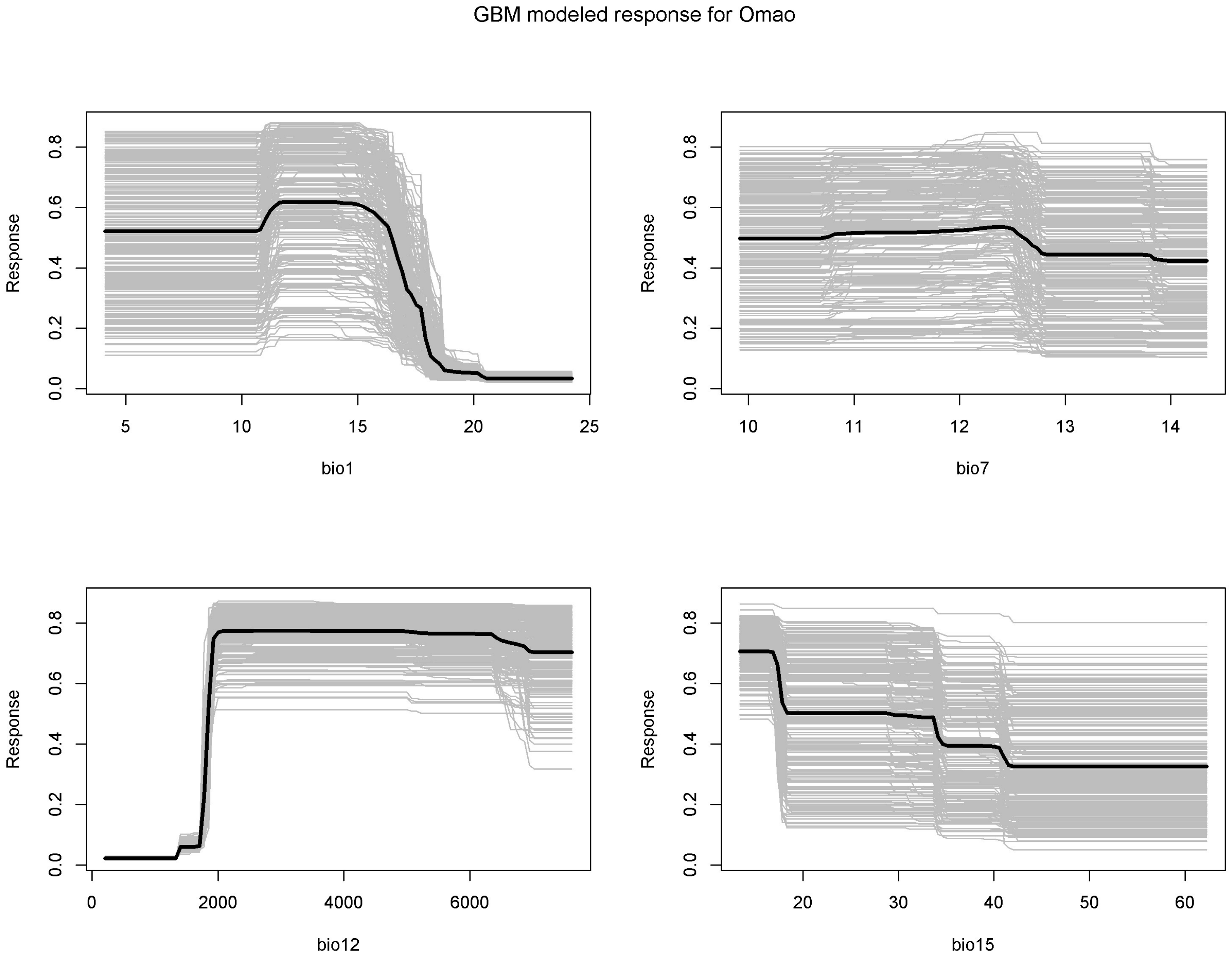

S3: SPECIES RESPONSE CURVES
REDUCED MODEL RELIABILITY SPECIES

Omao response curves MAXENT all vars

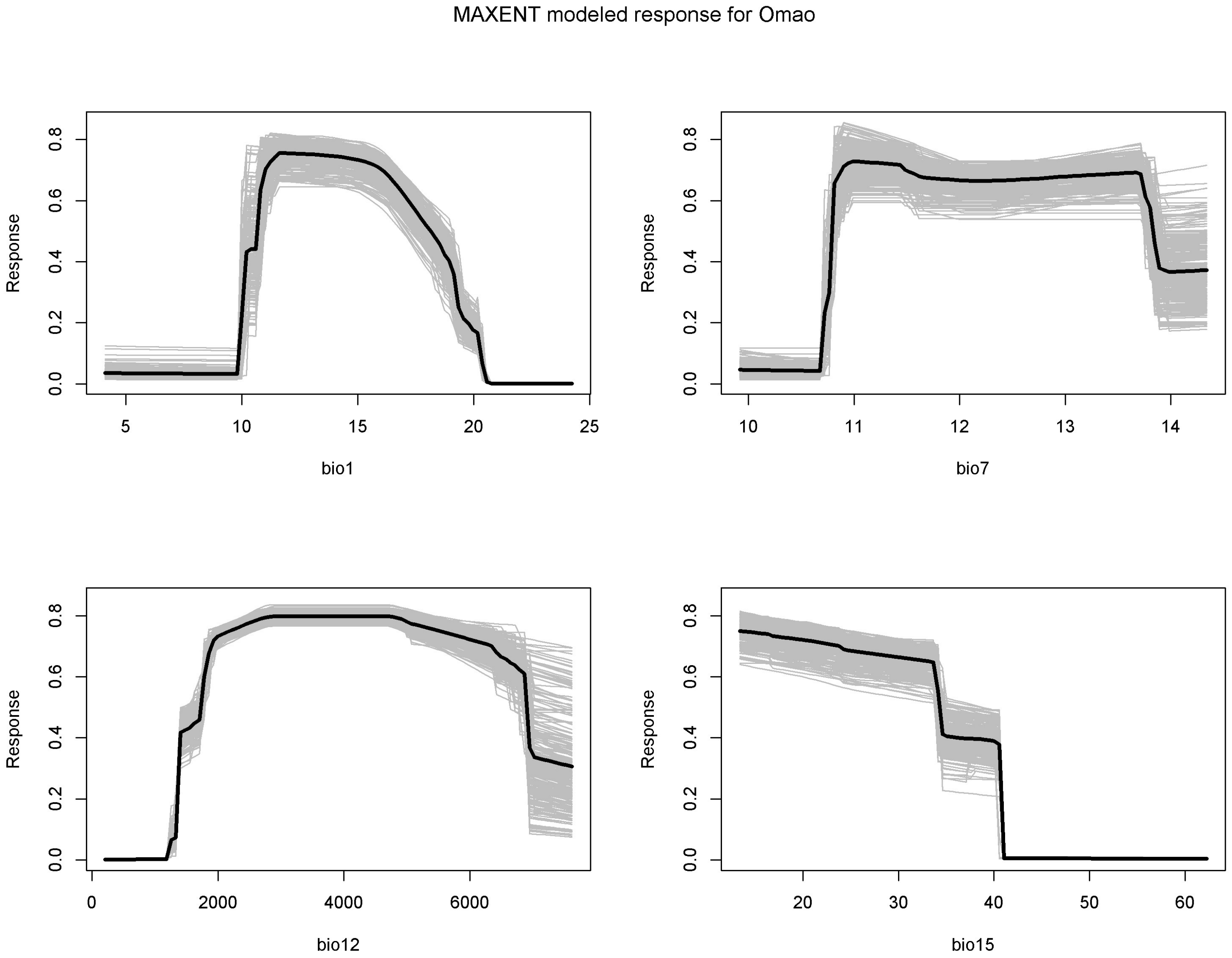

S3: SPECIES RESPONSE CURVES
REDUCED MODEL RELIABILITY SPECIES

Palila response curves GBM all vars

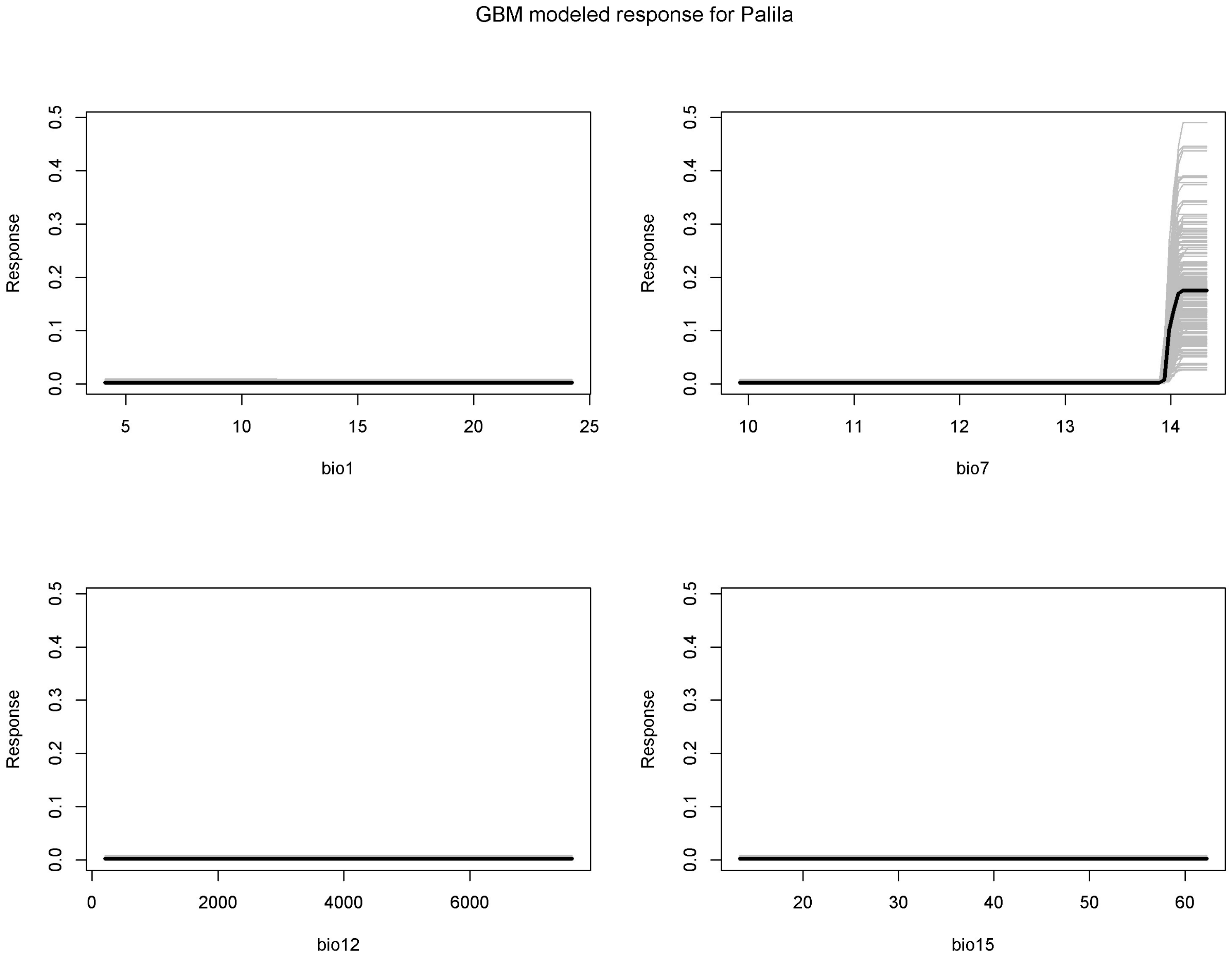

S3: SPECIES RESPONSE CURVES
REDUCED MODEL RELIABILITY SPECIES

Palila response curves MAXENT all vars

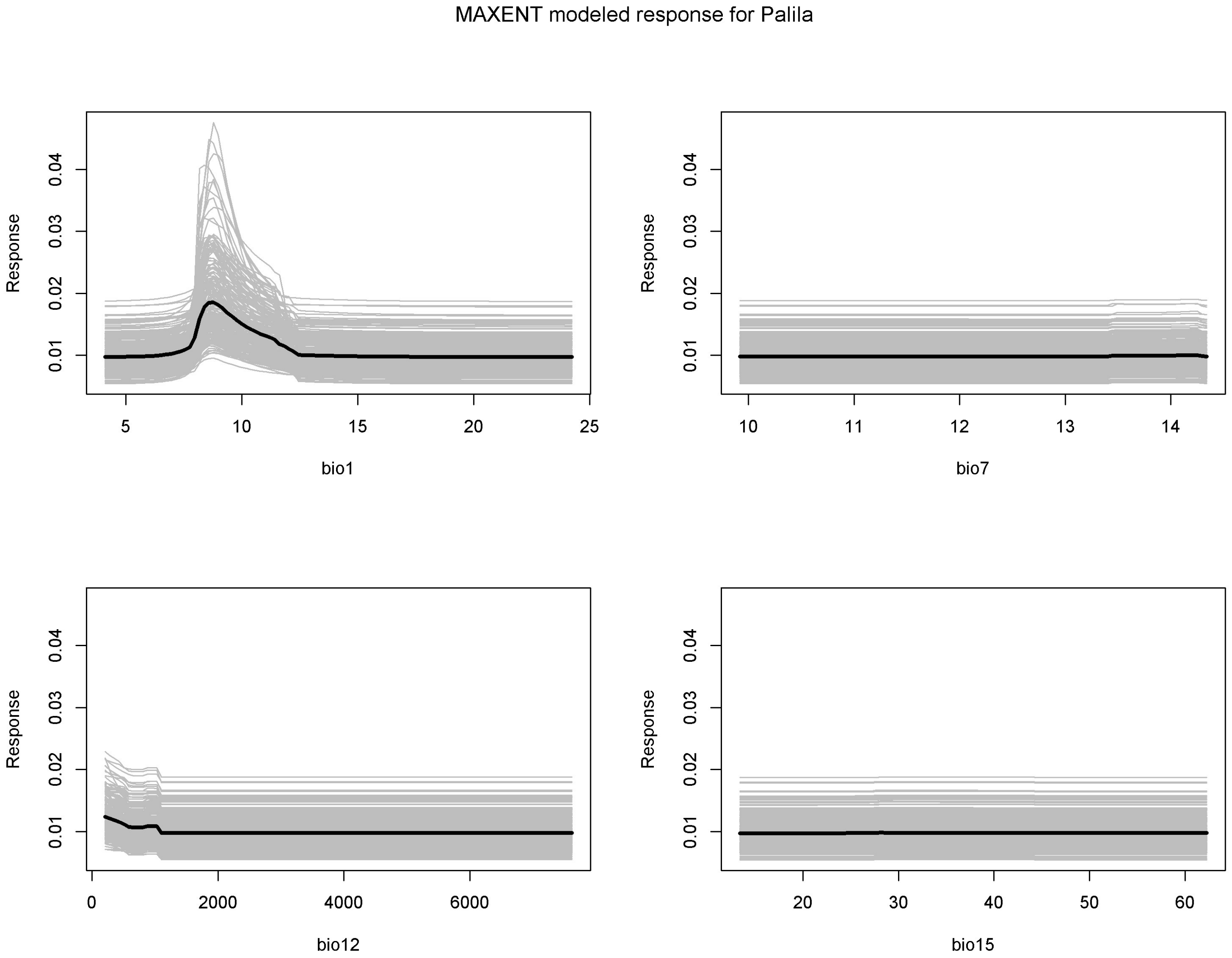

S3: VARIABLE IMPORTANCE GRAPHS
HIGH MODEL RELIABILITY SPECIES

Akekee GBM variable importance violin plot

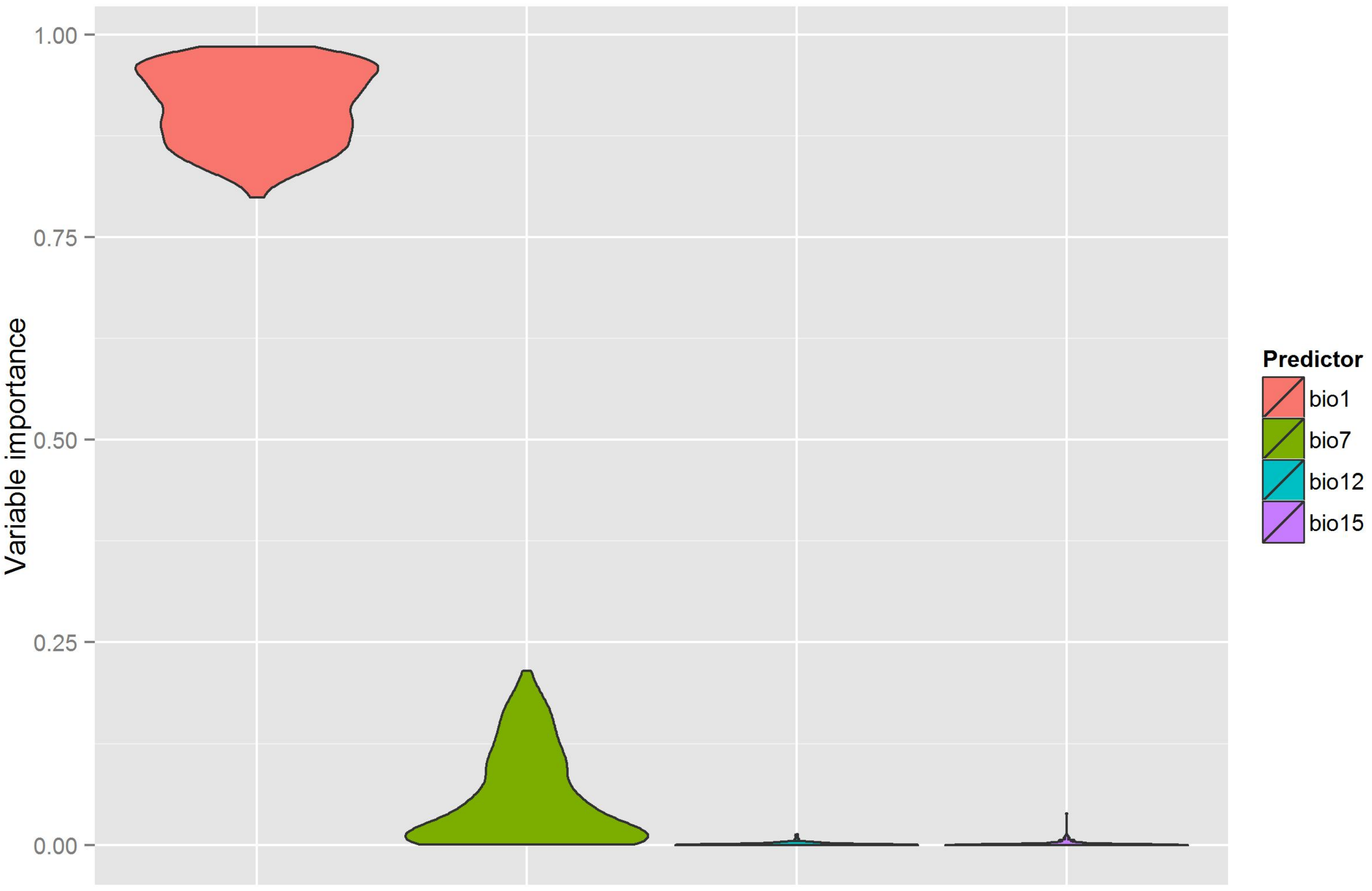

S3: VARIABLE IMPORTANCE GRAPHS
HIGH MODEL RELIABILITY SPECIES

Akekee MAXENT variable importance violin plot

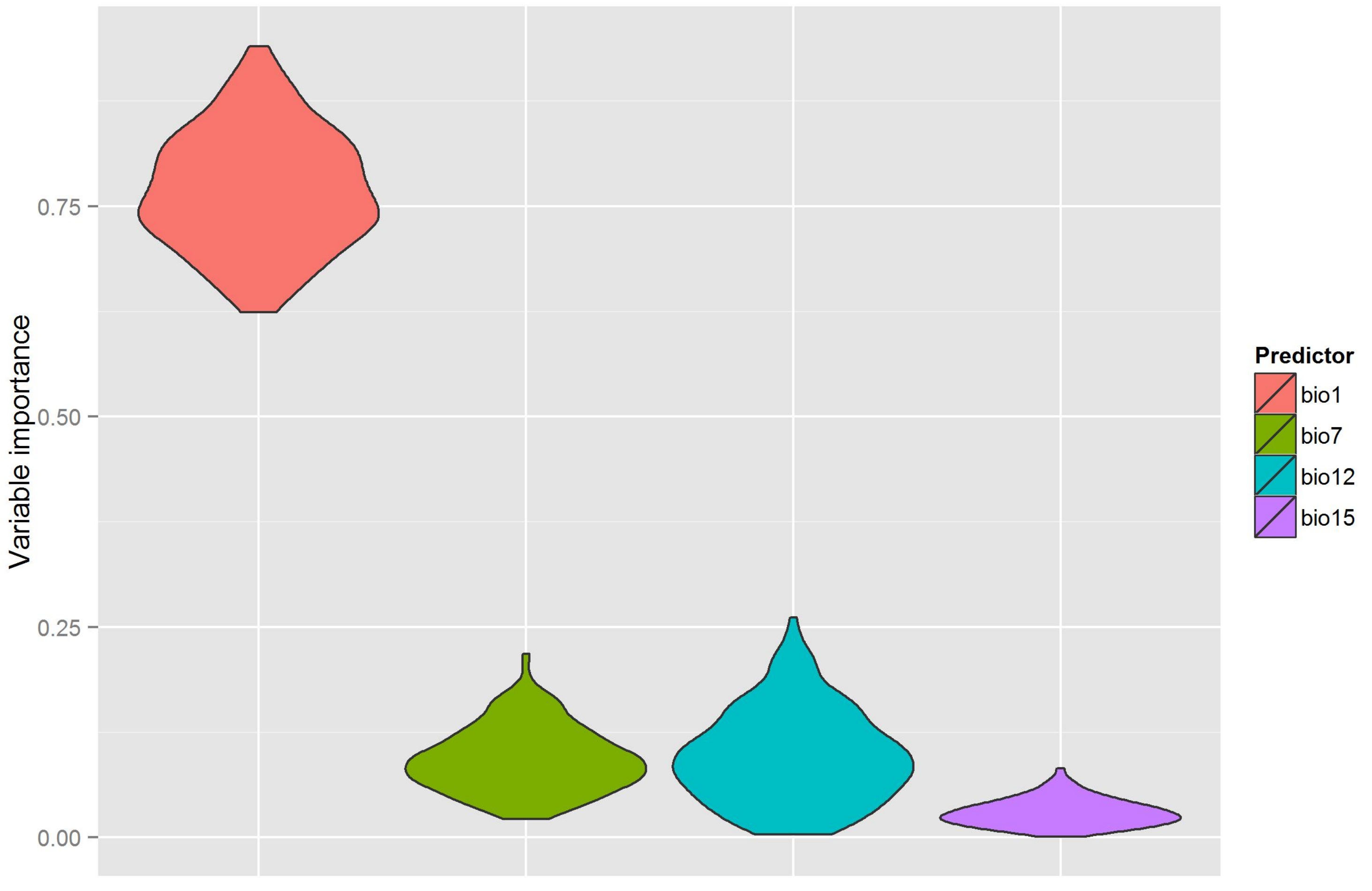

S3: VARIABLE IMPORTANCE GRAPHS
HIGH MODEL RELIABILITY SPECIES

Akiapolauu GBM variable importance violin plot

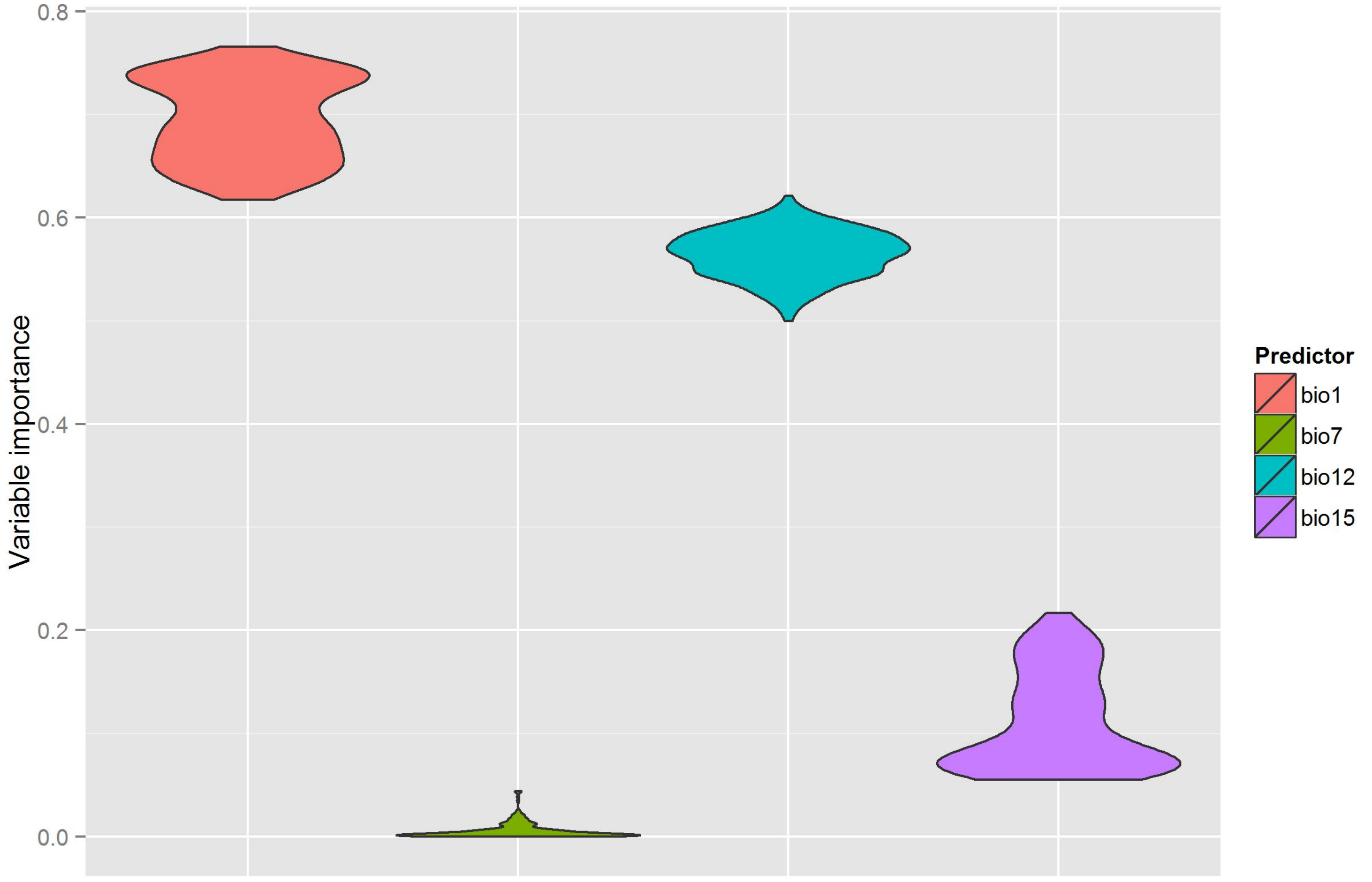

S3: VARIABLE IMPORTANCE GRAPHS
HIGH MODEL RELIABILITY SPECIES

Akiapolauu MAXENT variable importance violin plot

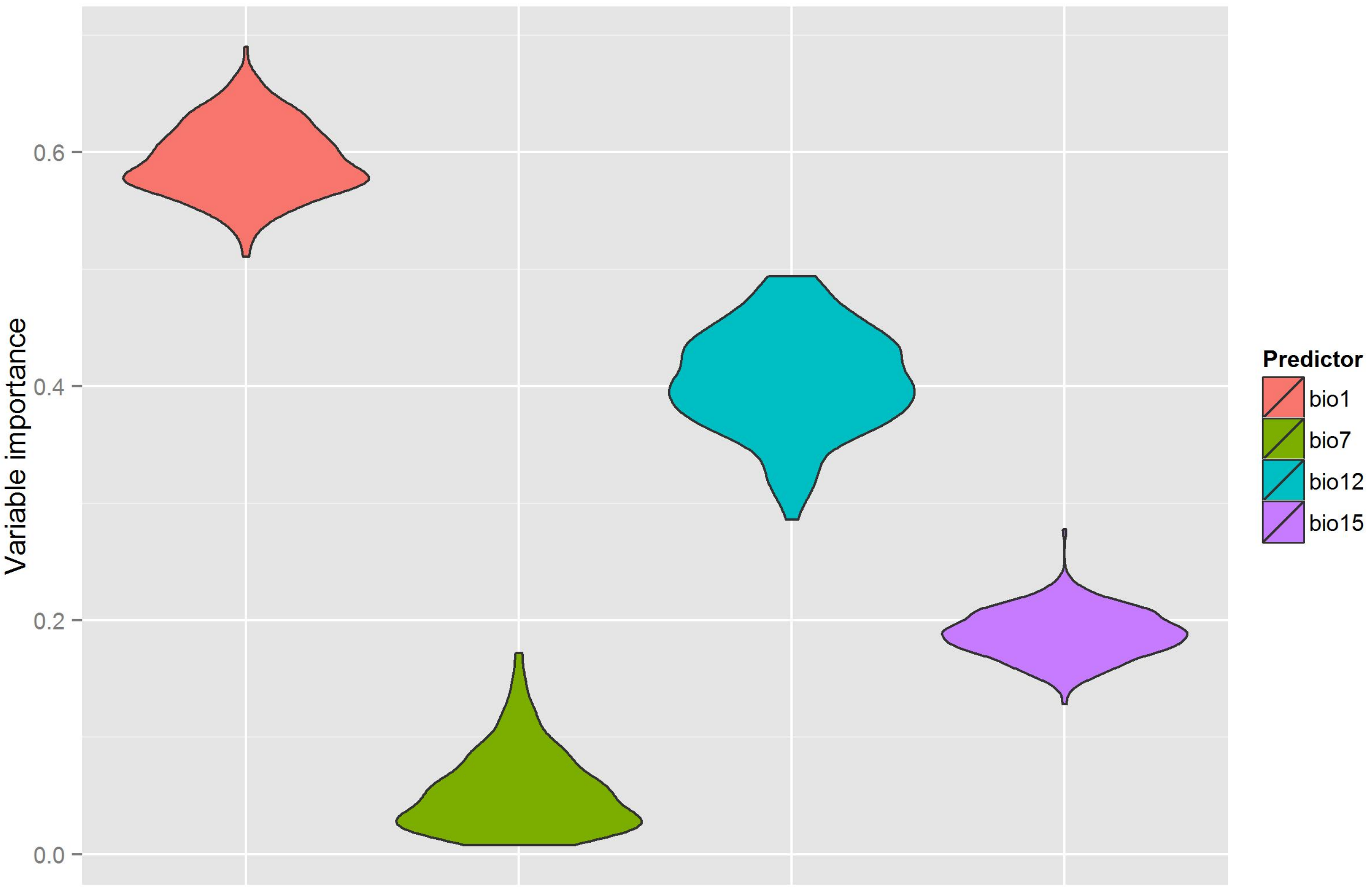

S3: VARIABLE IMPORTANCE GRAPHS
HIGH MODEL RELIABILITY SPECIES

Akikiki GBM variable importance violin plot

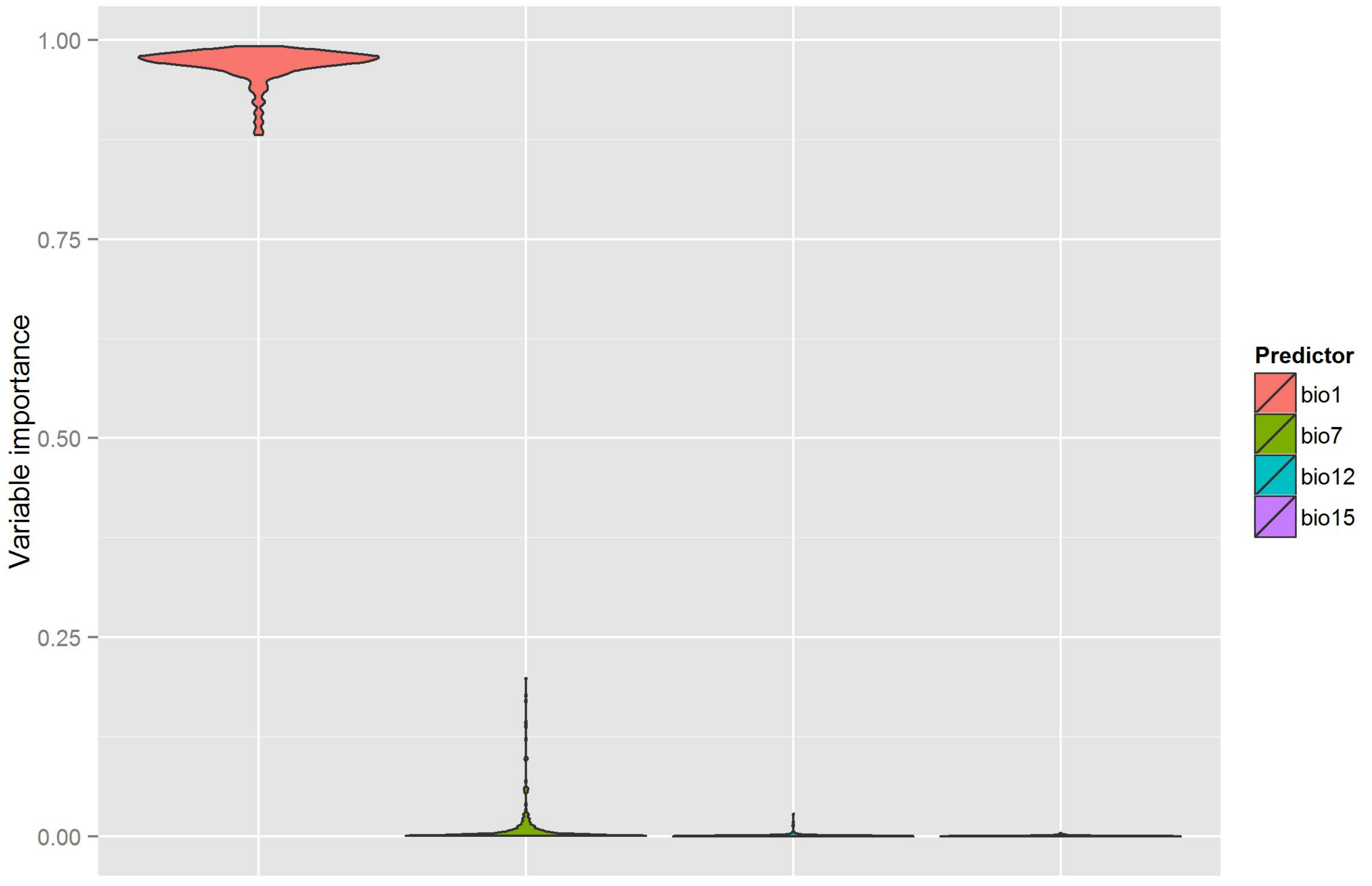

S3: VARIABLE IMPORTANCE GRAPHS
HIGH MODEL RELIABILITY SPECIES

Akikiki MAXENT variable importance violin plot

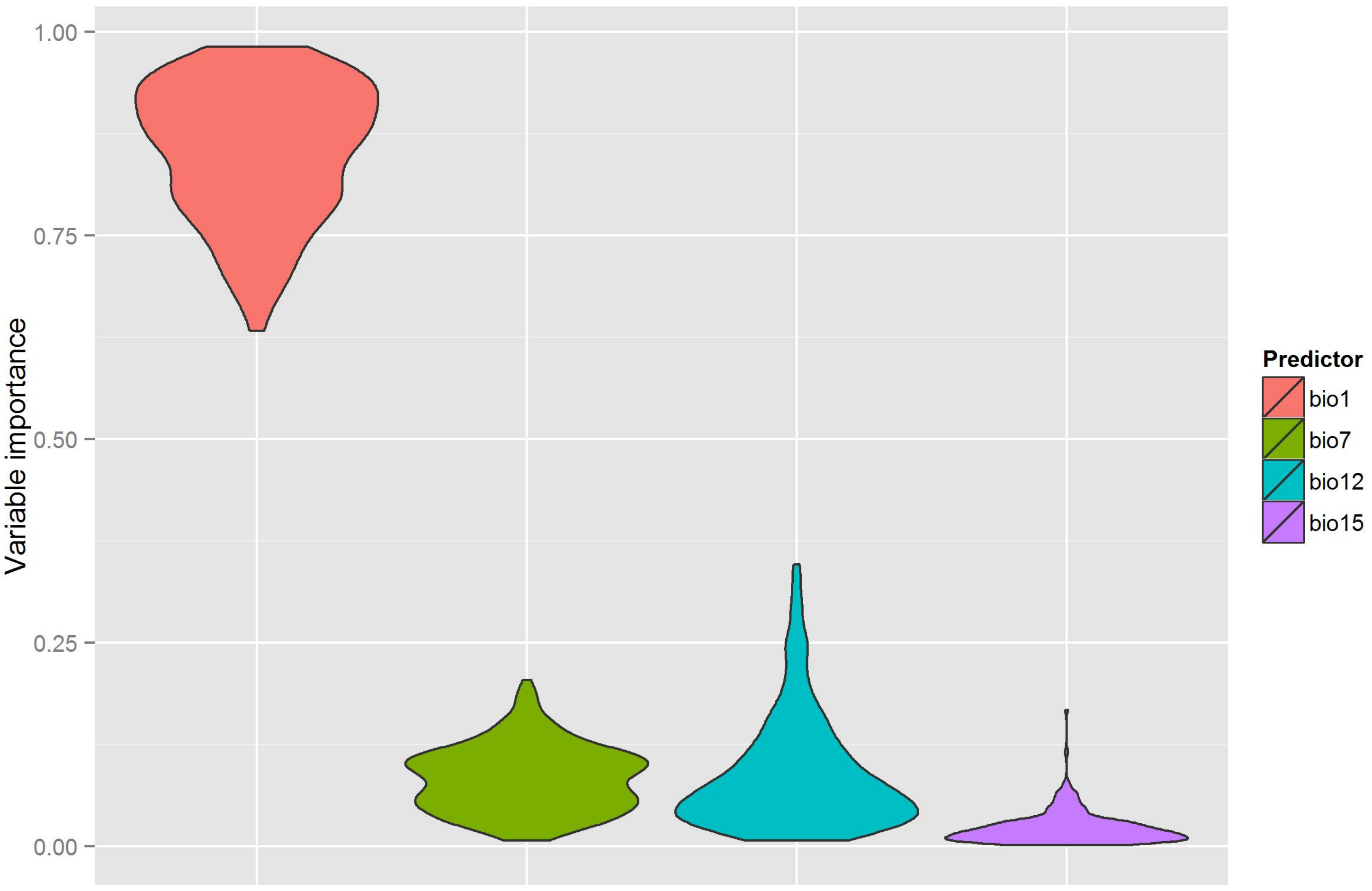

S3: VARIABLE IMPORTANCE GRAPHS
HIGH MODEL RELIABILITY SPECIES

Akohekohe GBM variable importance violin plot

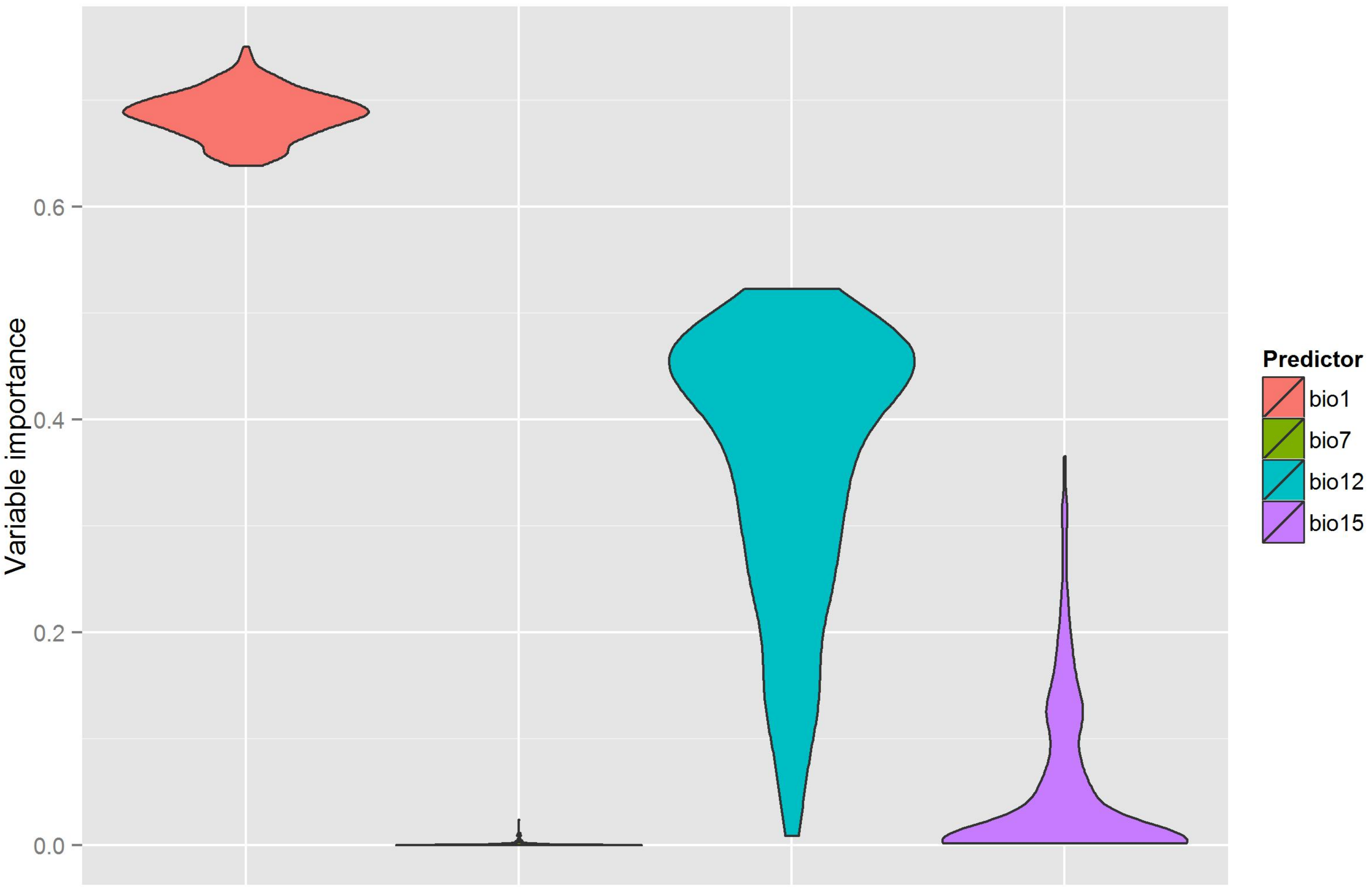

S3: VARIABLE IMPORTANCE GRAPHS
HIGH MODEL RELIABILITY SPECIES

Akohekohe MAXENT variable importance violin plot

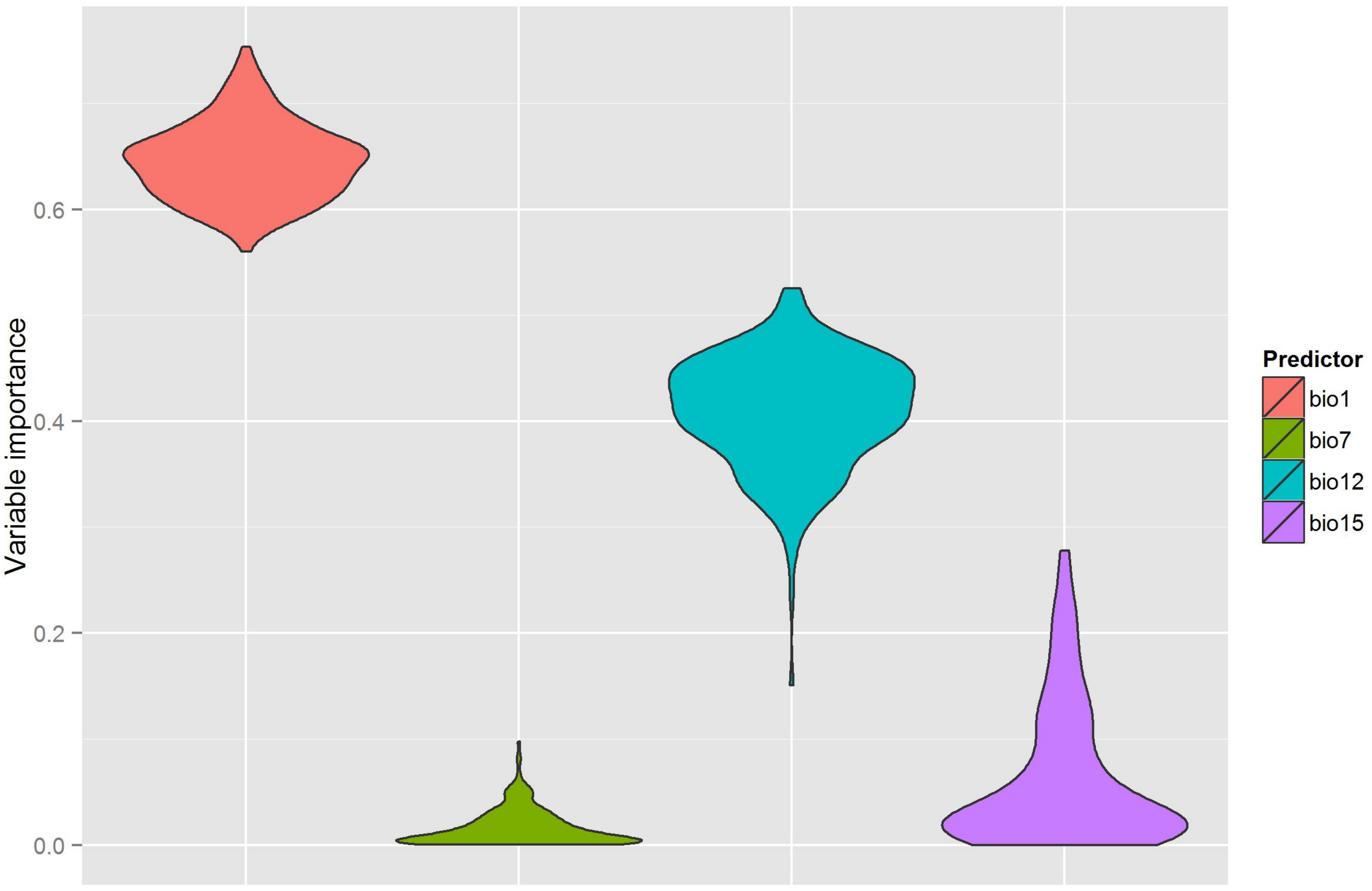

S3: VARIABLE IMPORTANCE GRAPHS
HIGH MODEL RELIABILITY SPECIES

Hawaii Akepa GBM variable importance violin plot

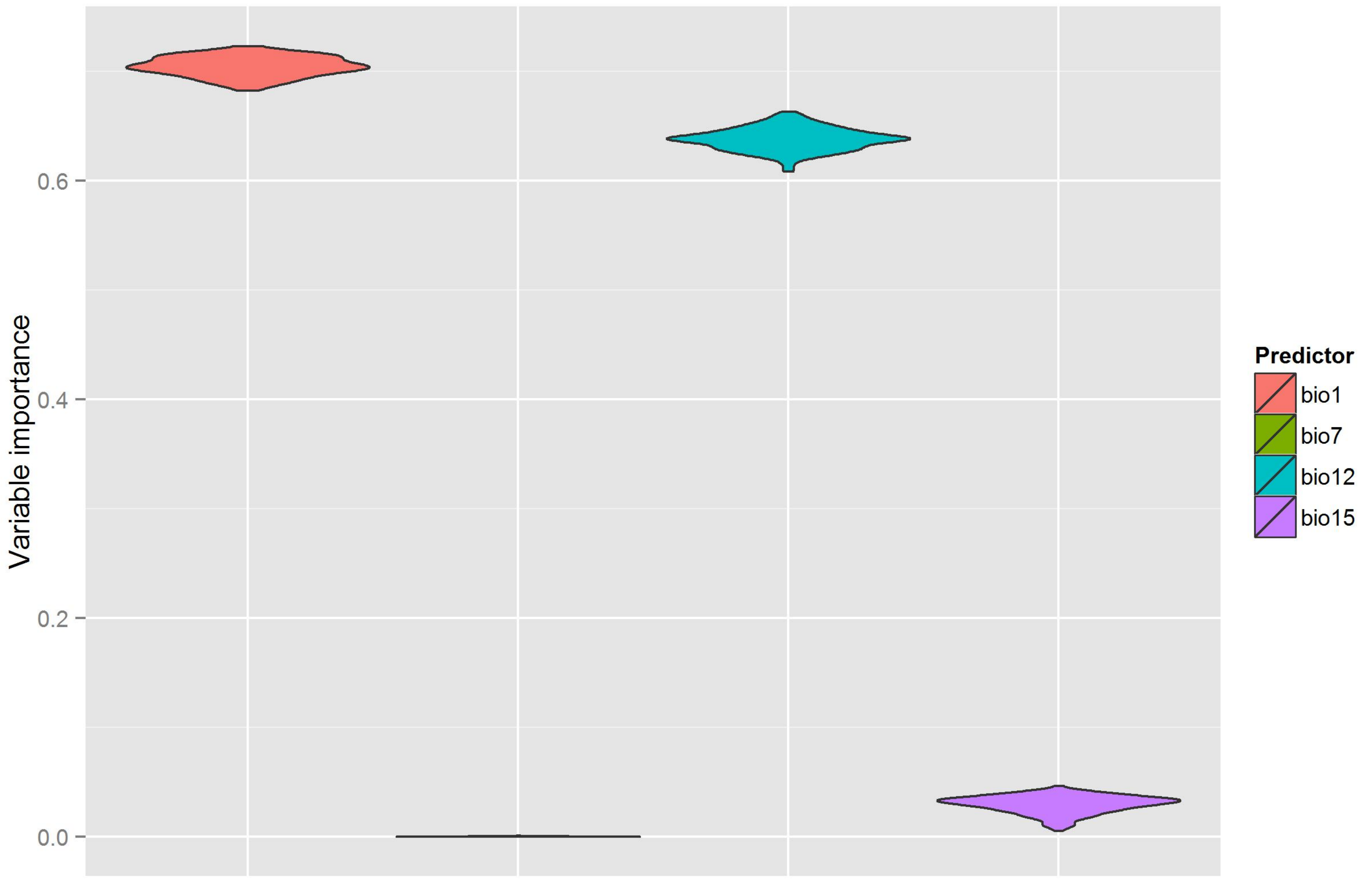

S3: VARIABLE IMPORTANCE GRAPHS
HIGH MODEL RELIABILITY SPECIES

Hawaii Akepa MAXENT variable importance violin plot

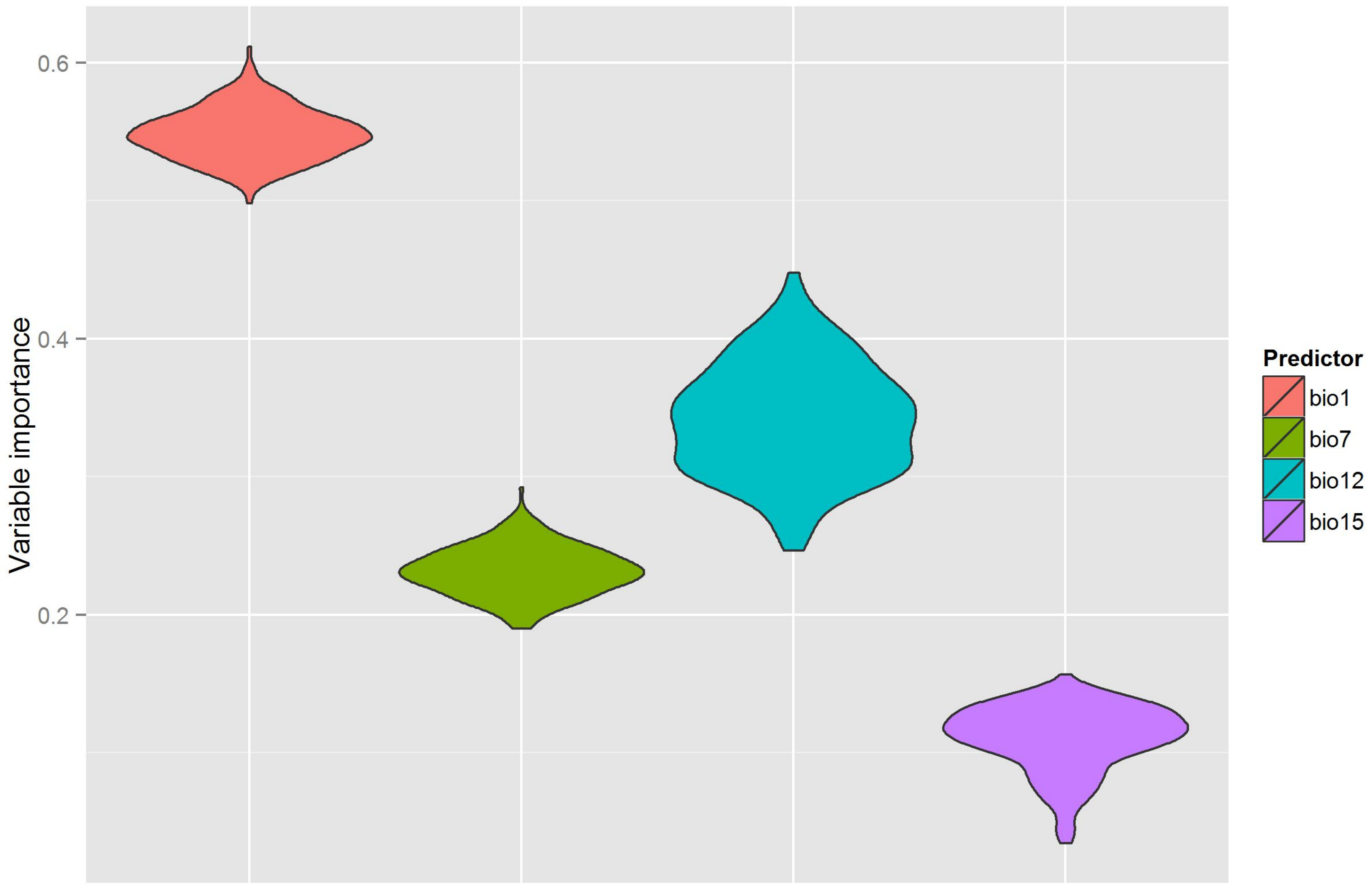

S3: VARIABLE IMPORTANCE GRAPHS
HIGH MODEL RELIABILITY SPECIES

Hawaii Creeper GBM variable importance violin plot

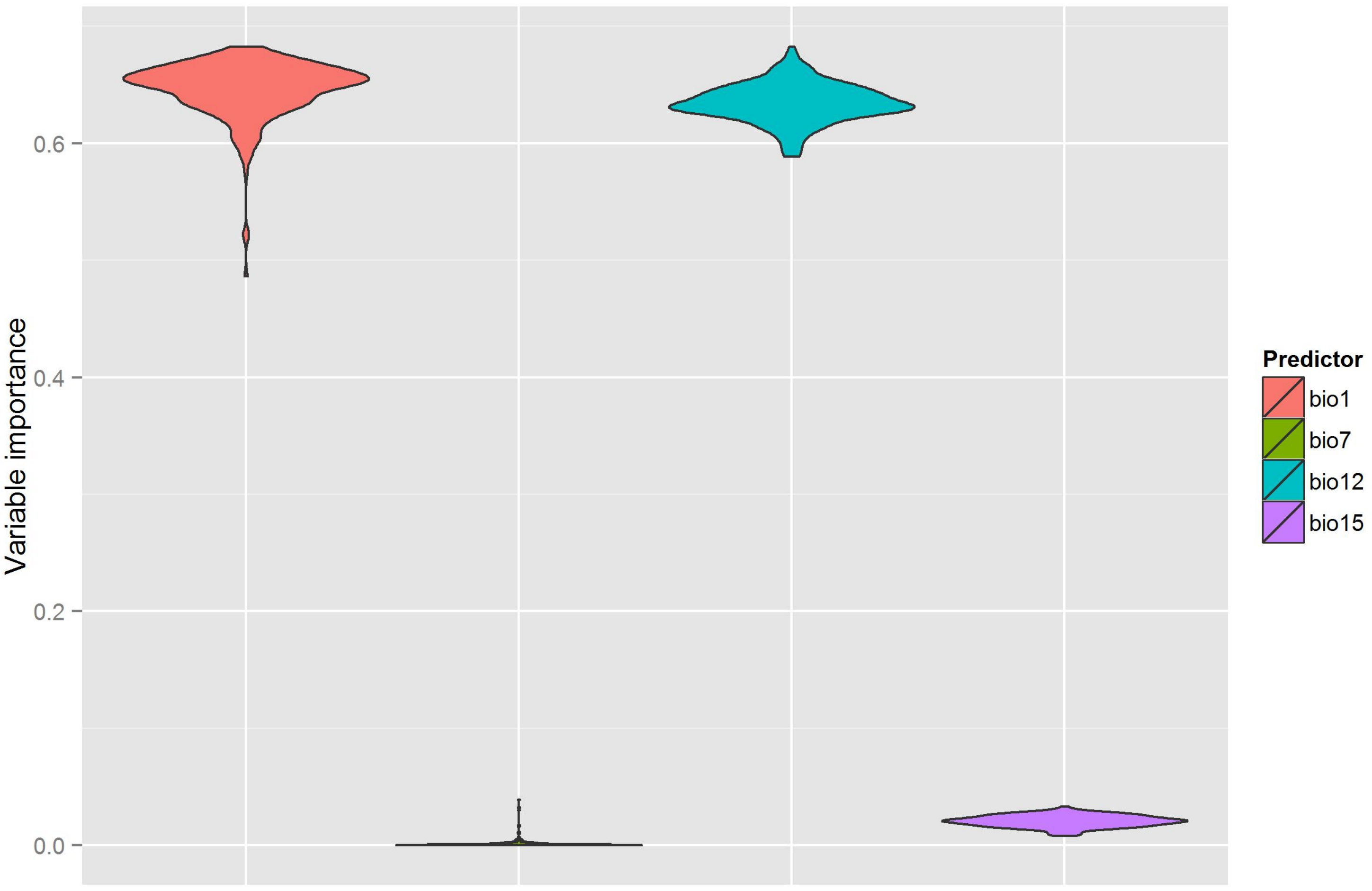

S3: VARIABLE IMPORTANCE GRAPHS
HIGH MODEL RELIABILITY SPECIES

Hawaii Creeper MAXENT variable importance violin plot

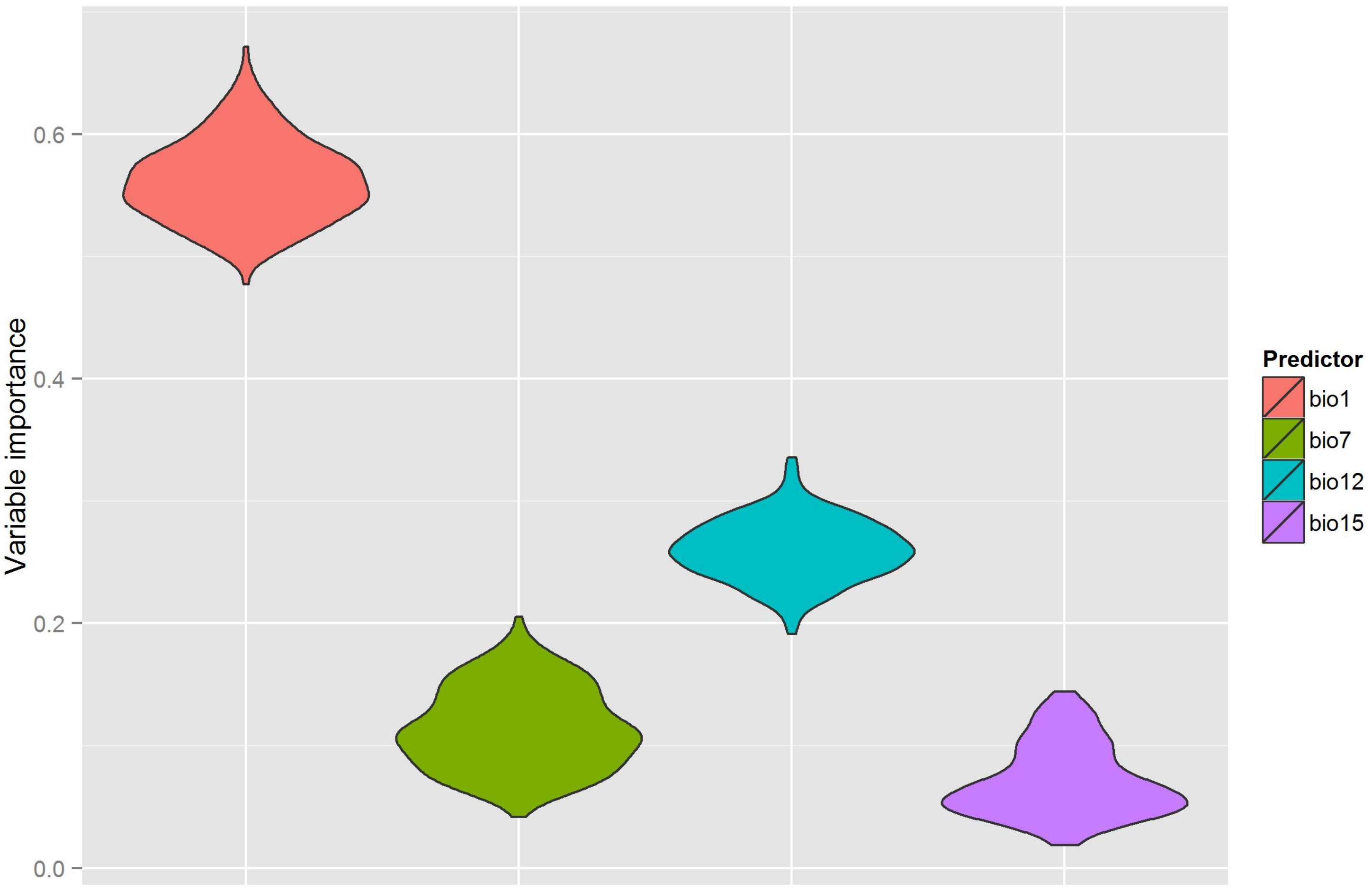

S3: VARIABLE IMPORTANCE GRAPHS
HIGH MODEL RELIABILITY SPECIES

Iiwi GBM variable importance violin plot

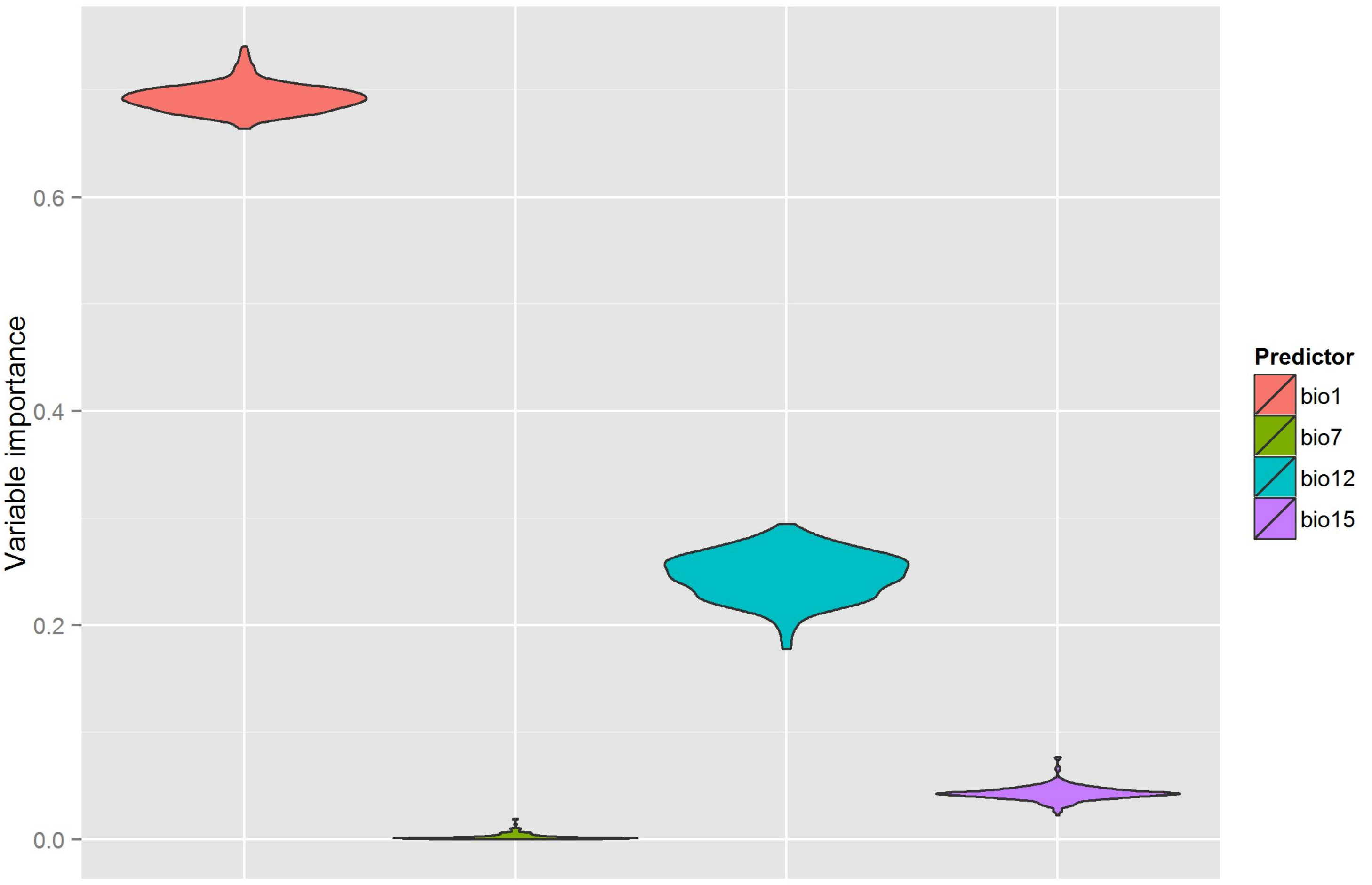

S3: VARIABLE IMPORTANCE GRAPHS
HIGH MODEL RELIABILITY SPECIES

Iiwi MAXENT variable importance violin plot

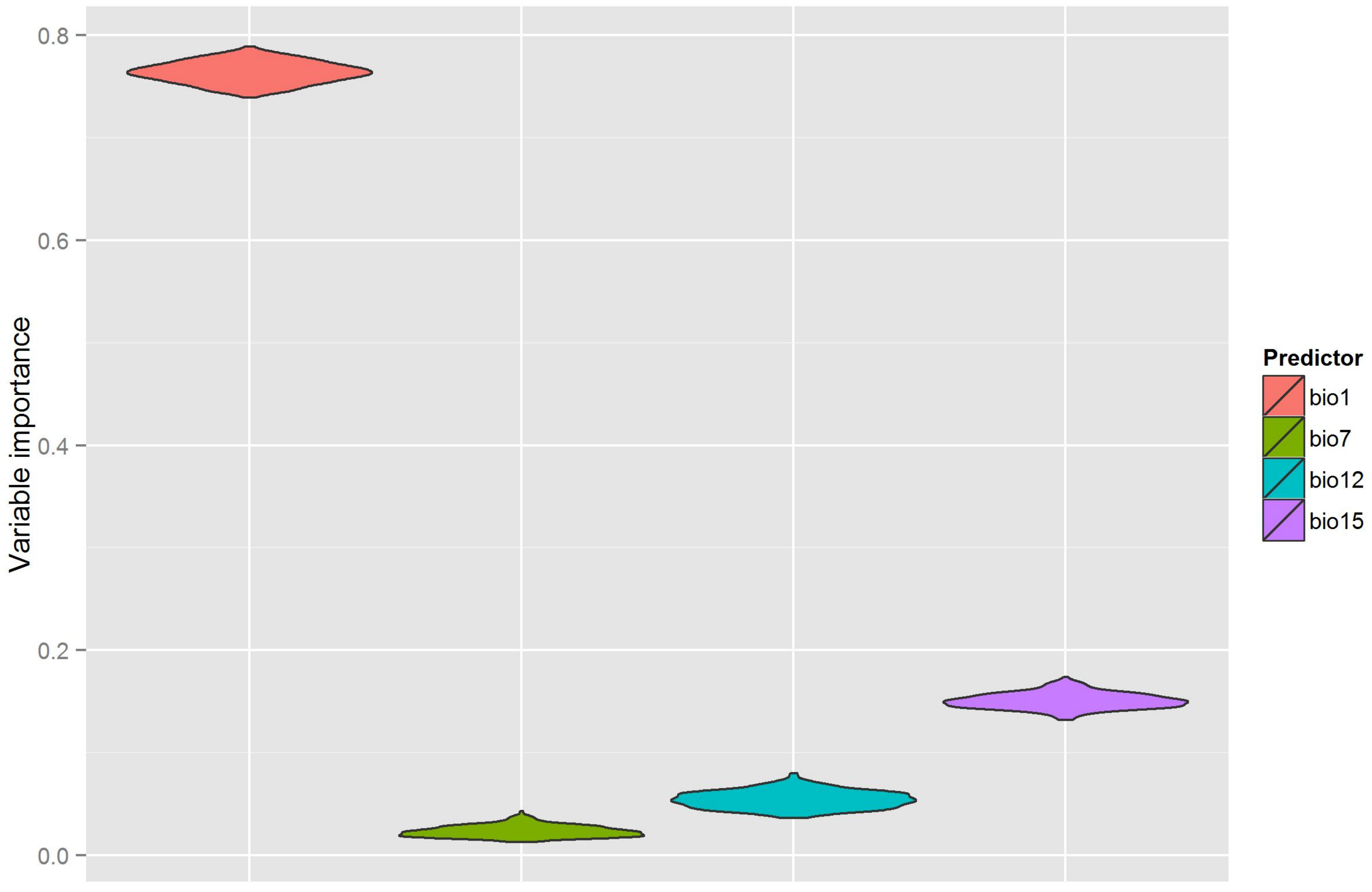

S3: VARIABLE IMPORTANCE GRAPHS
HIGH MODEL RELIABILITY SPECIES

Maui Alauahio GBM variable importance violin plot

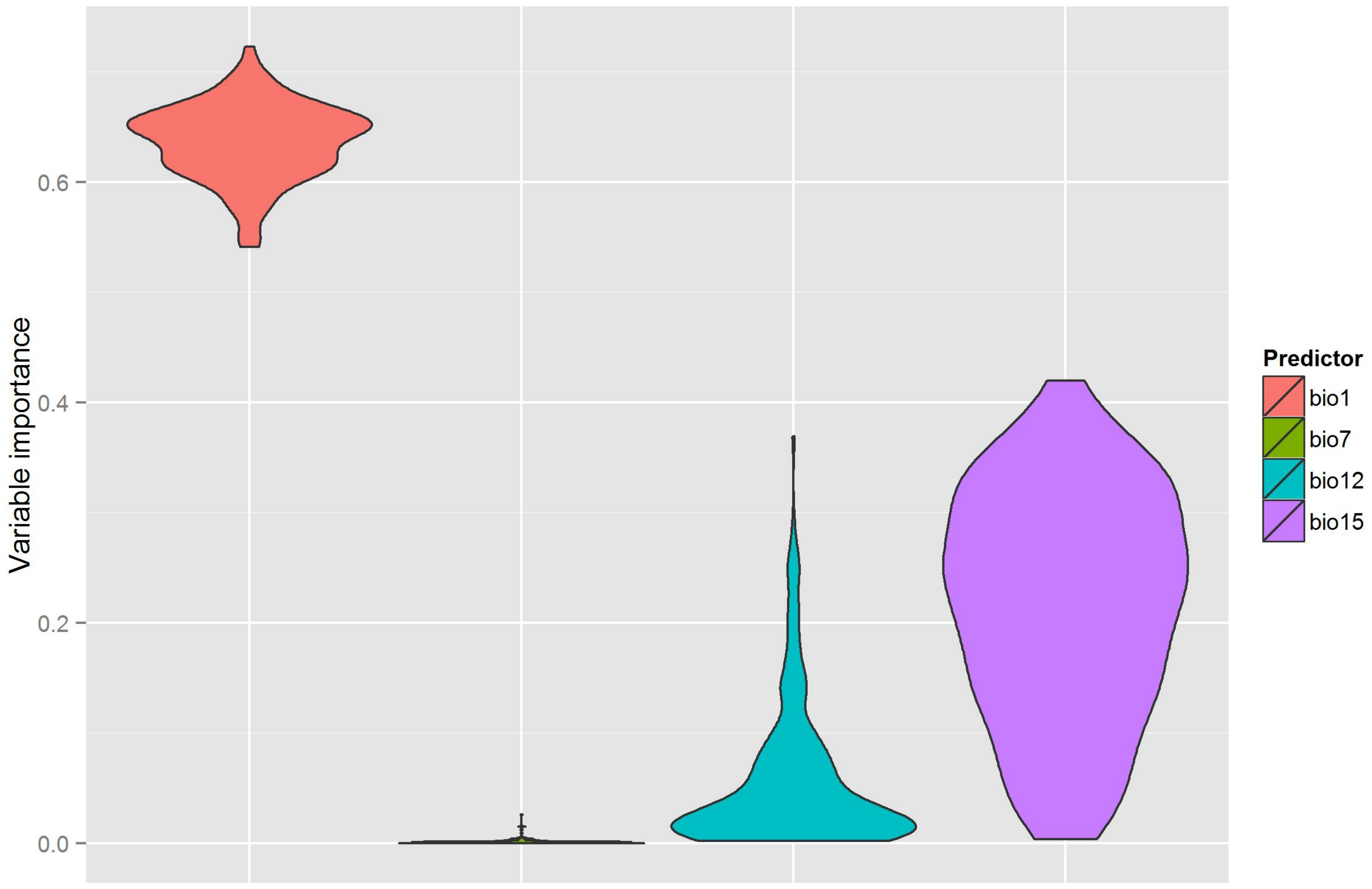

S3: VARIABLE IMPORTANCE GRAPHS
HIGH MODEL RELIABILITY SPECIES

Maui Alauahio MAXENT variable importance violin plot

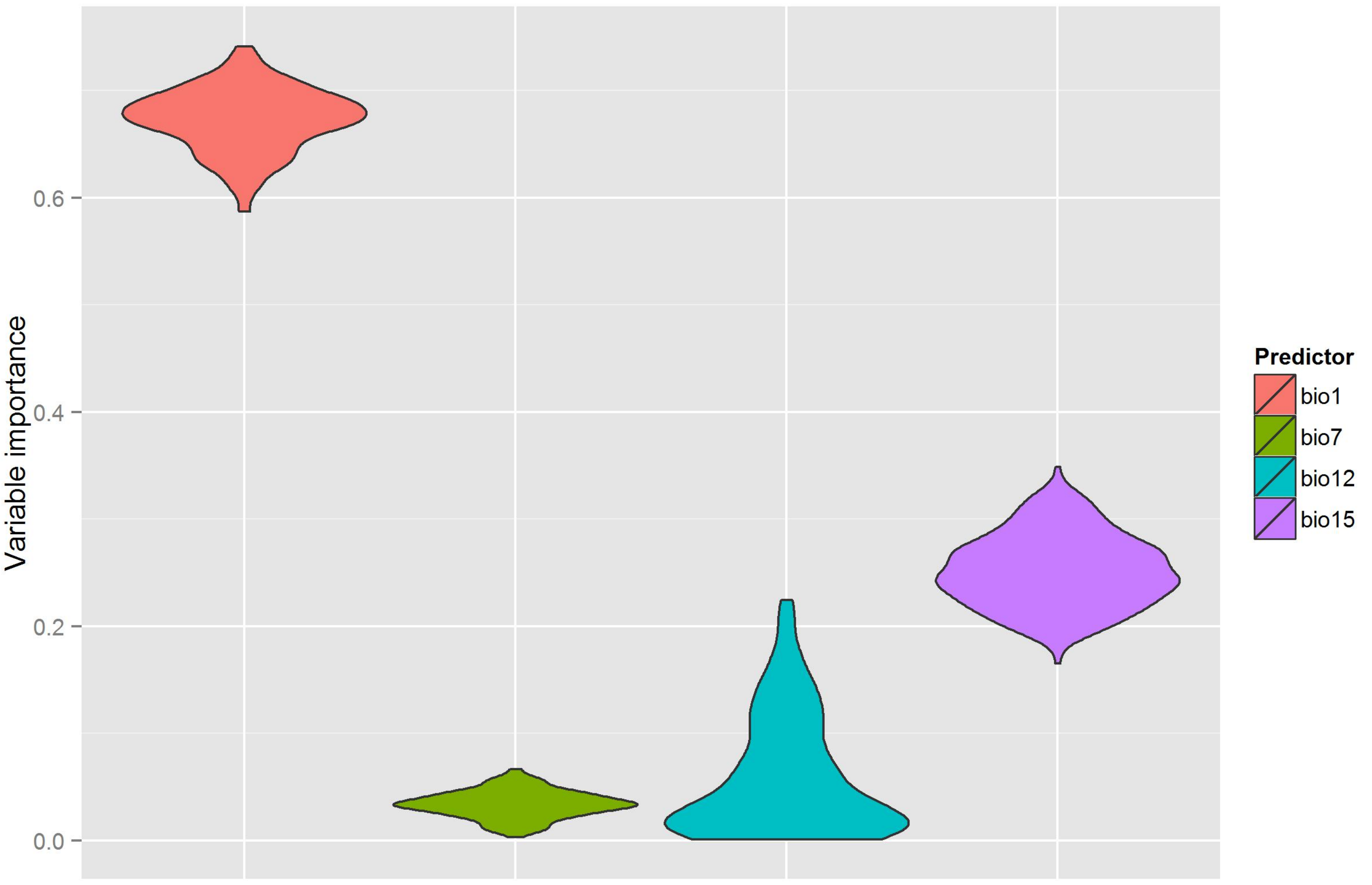

S3: VARIABLE IMPORTANCE GRAPHS
HIGH MODEL RELIABILITY SPECIES

Maui Parrotbill GBM variable importance violin plot

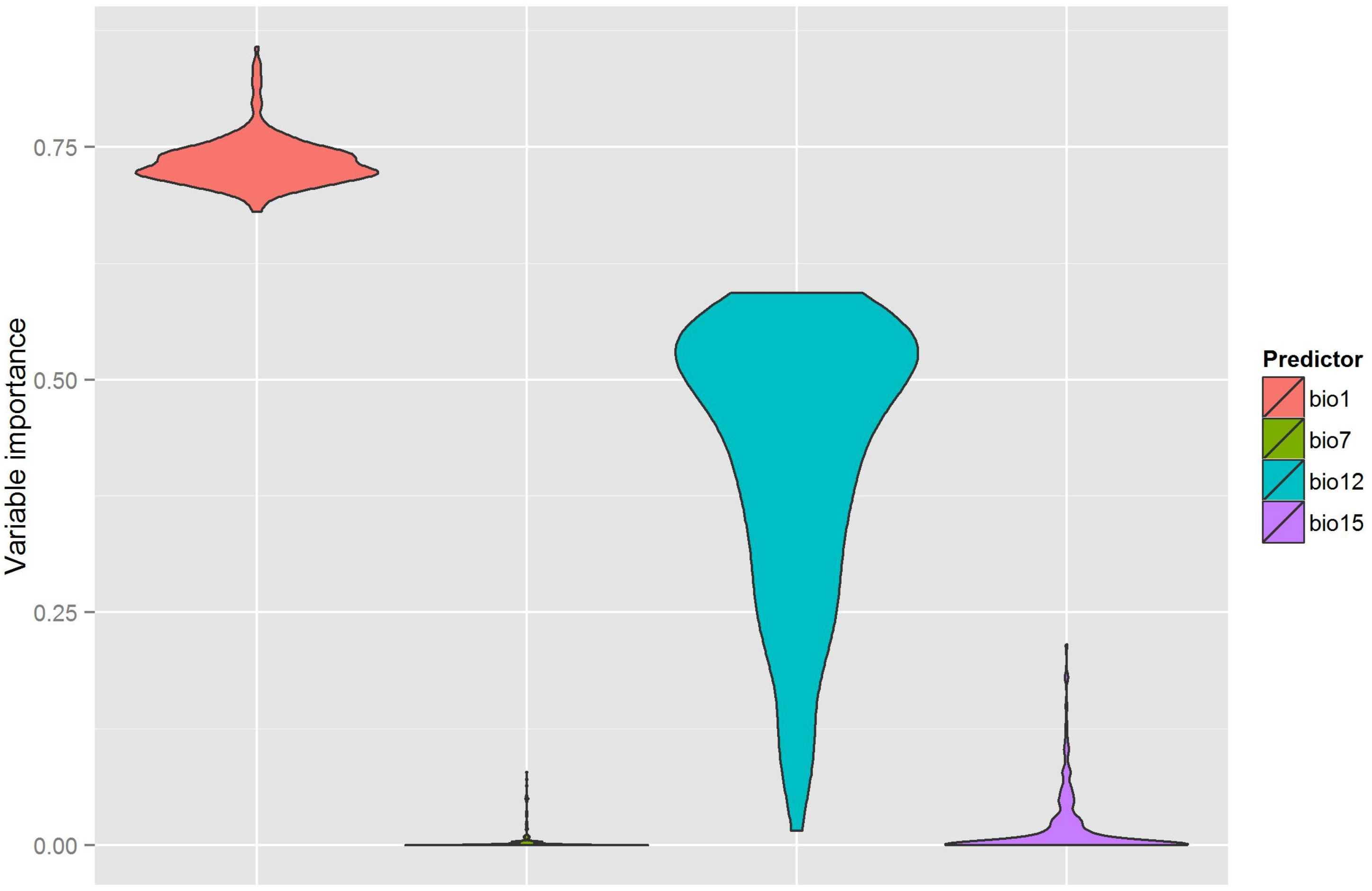

S3: VARIABLE IMPORTANCE GRAPHS
HIGH MODEL RELIABILITY SPECIES

Maui Parrotbill MAXENT variable importance violin plot

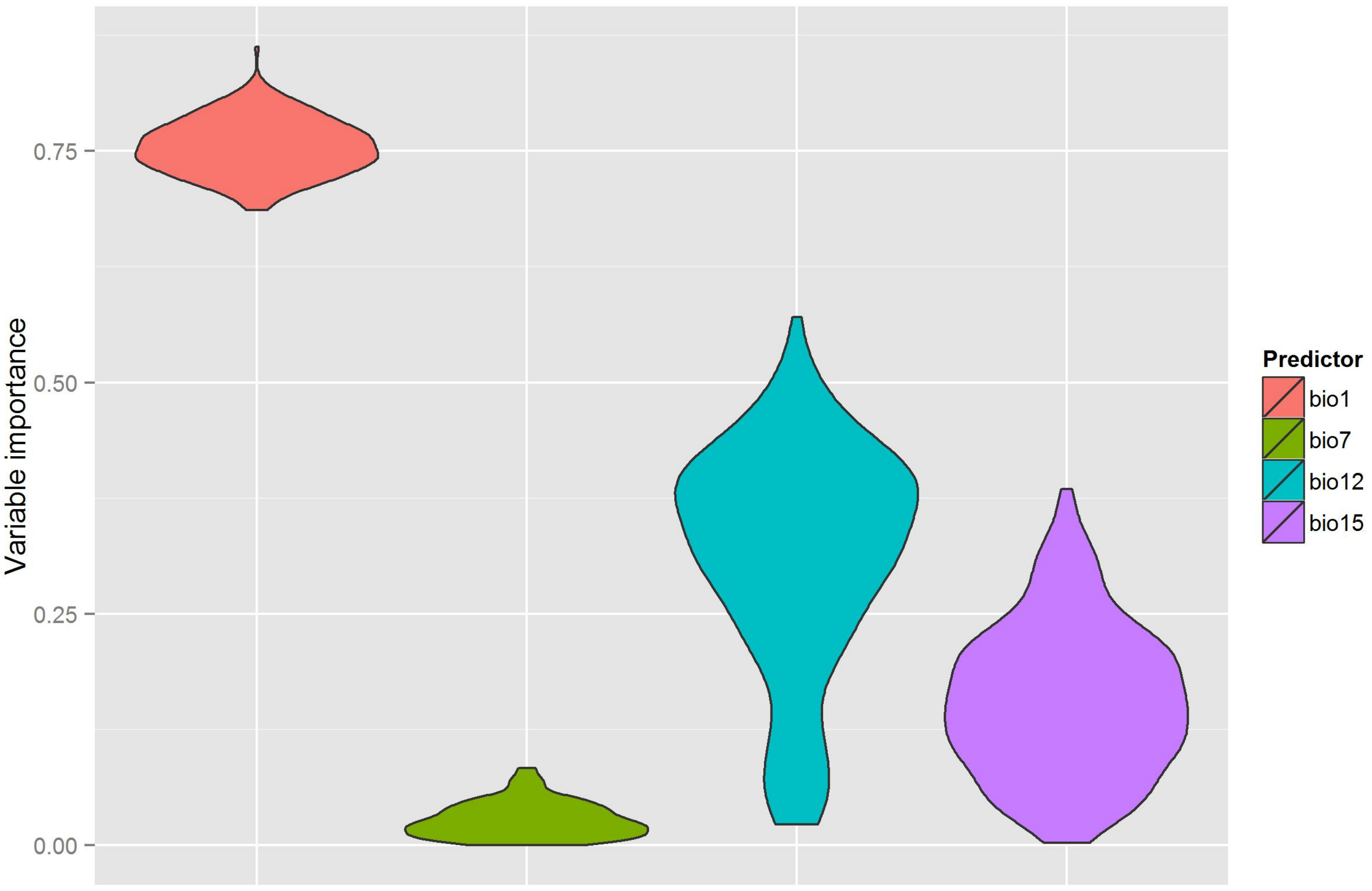

S3: VARIABLE IMPORTANCE GRAPHS
HIGH MODEL RELIABILITY SPECIES

Puaiohi GBM variable importance violin plot

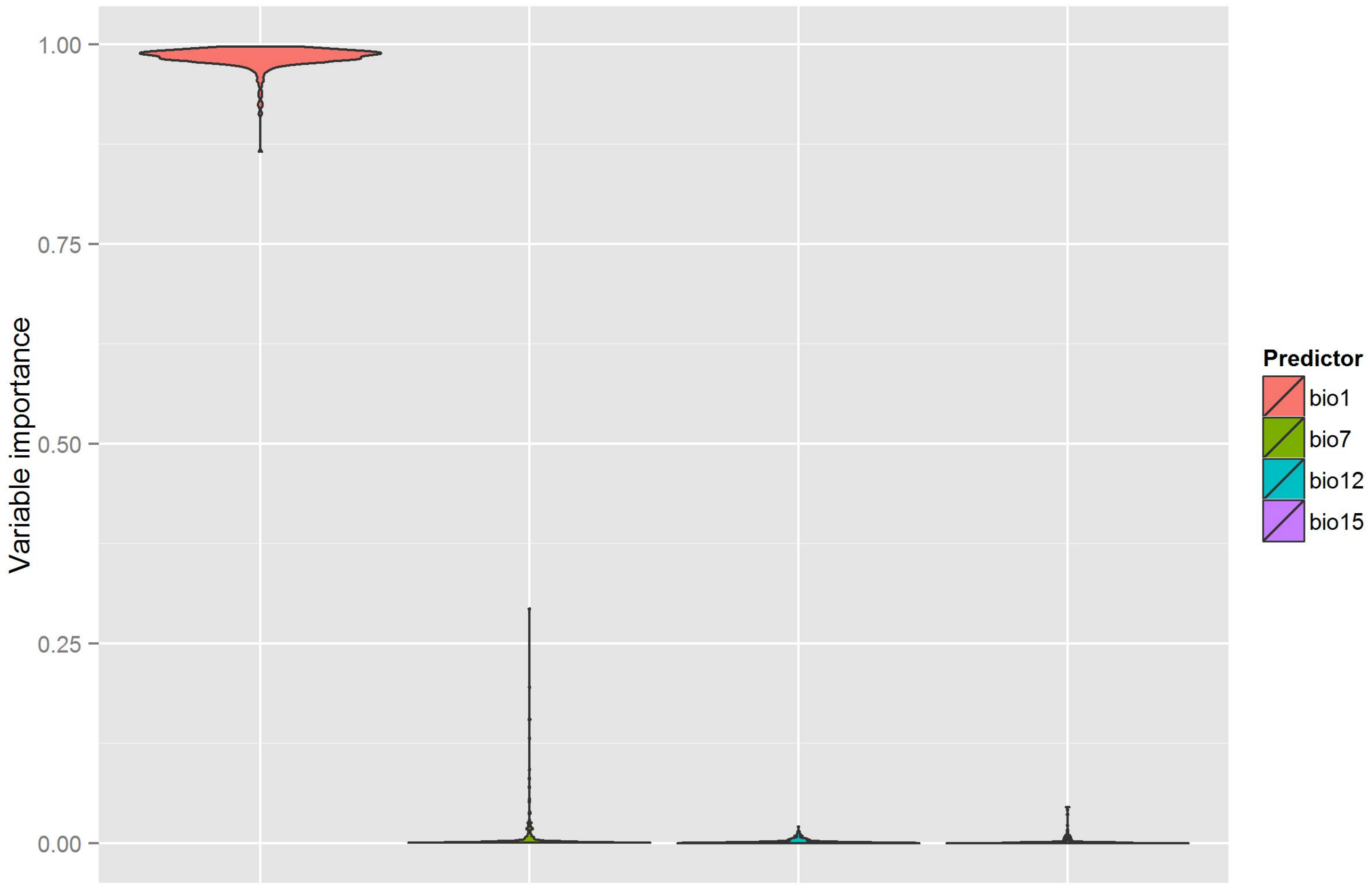

S3: VARIABLE IMPORTANCE GRAPHS
HIGH MODEL RELIABILITY SPECIES

Puaiohi MAXENT variable importance violin plot

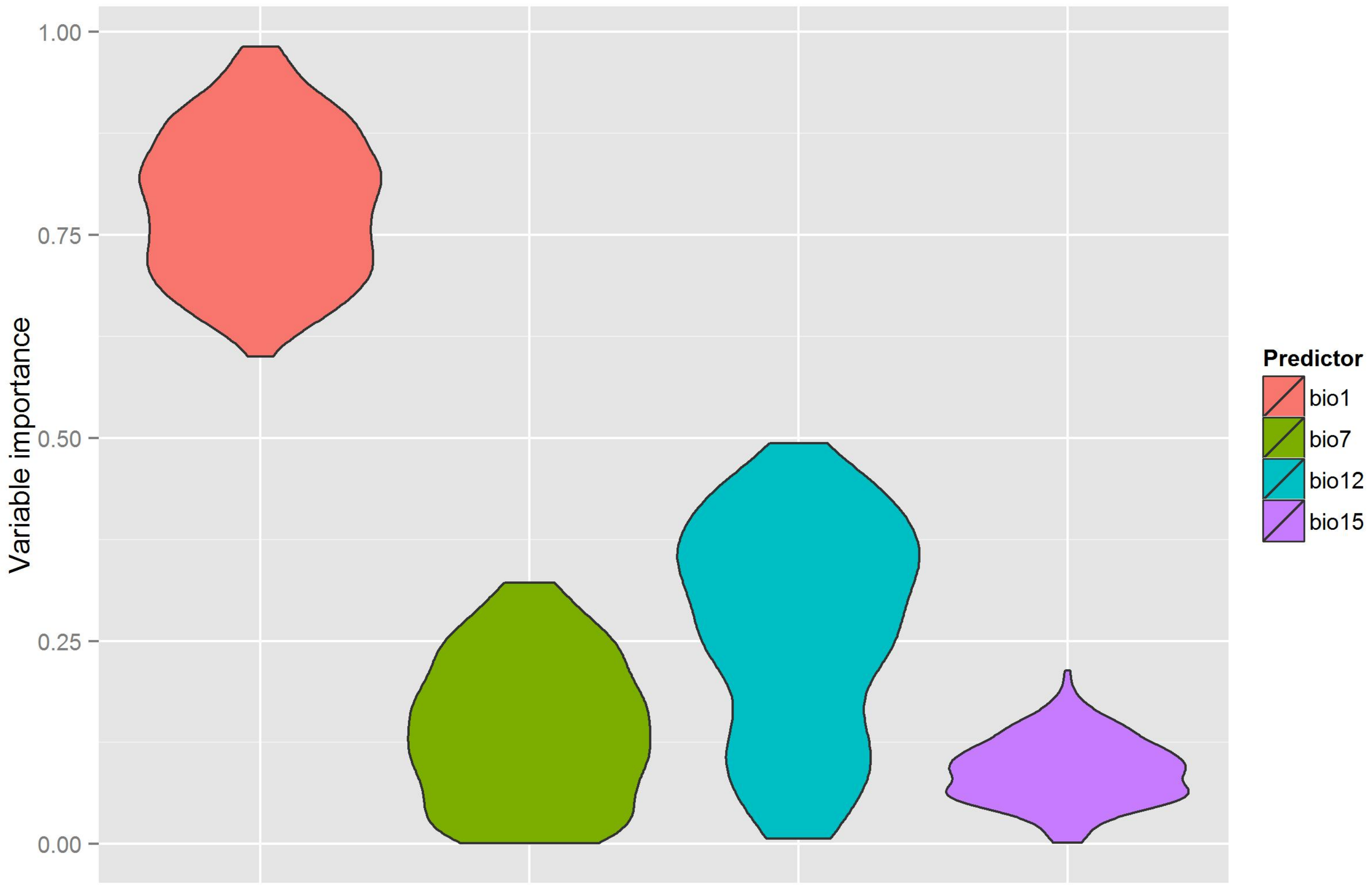

S3: VARIABLE IMPORTANCE GRAPHS
REDUCED MODEL RELIABILITY SPECIES

Anianiau GBM variable importance violin plot

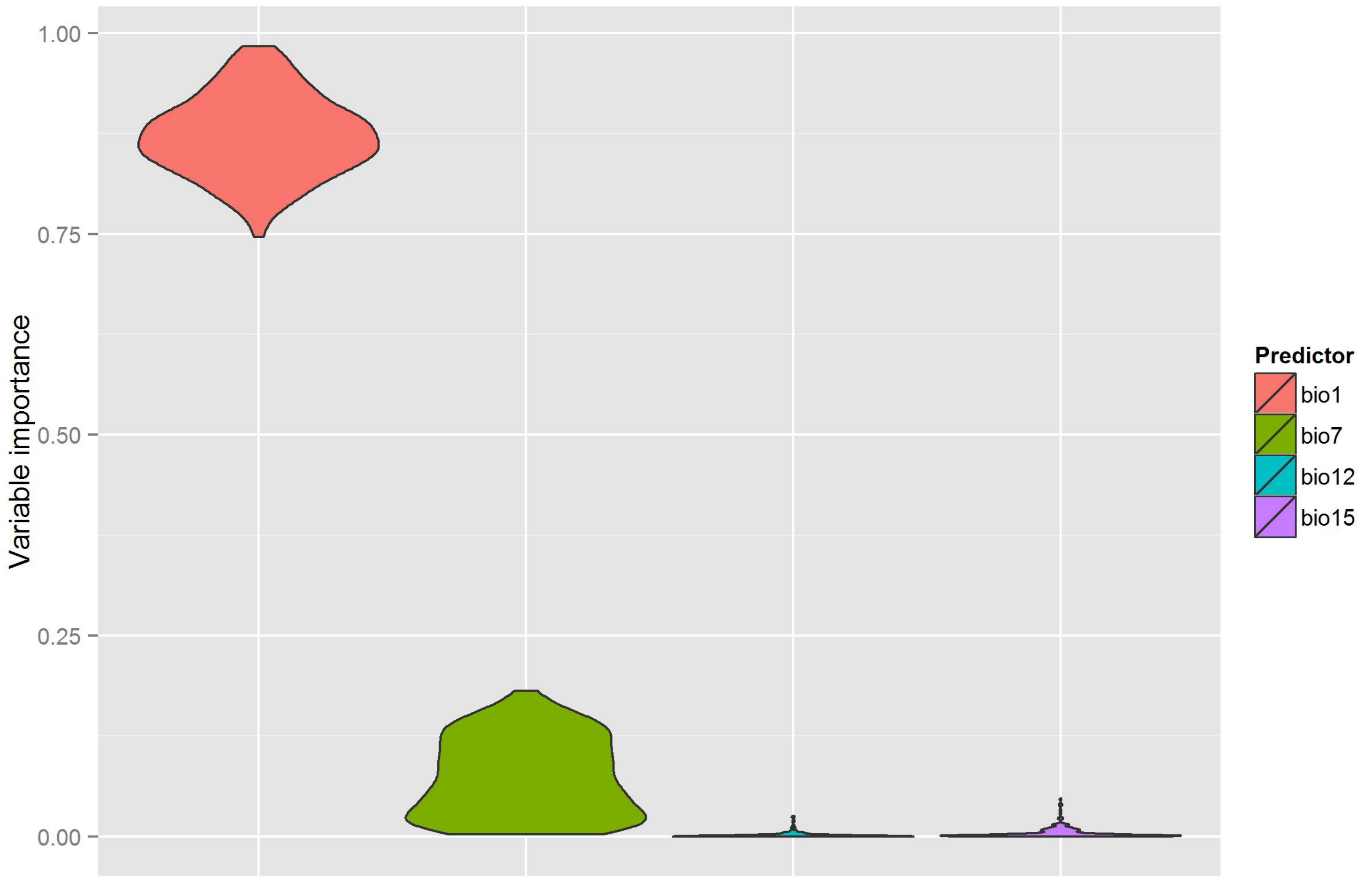

S3: VARIABLE IMPORTANCE GRAPHS
REDUCED MODEL RELIABILITY SPECIES

Anianiau MAXENT variable importance violin plot

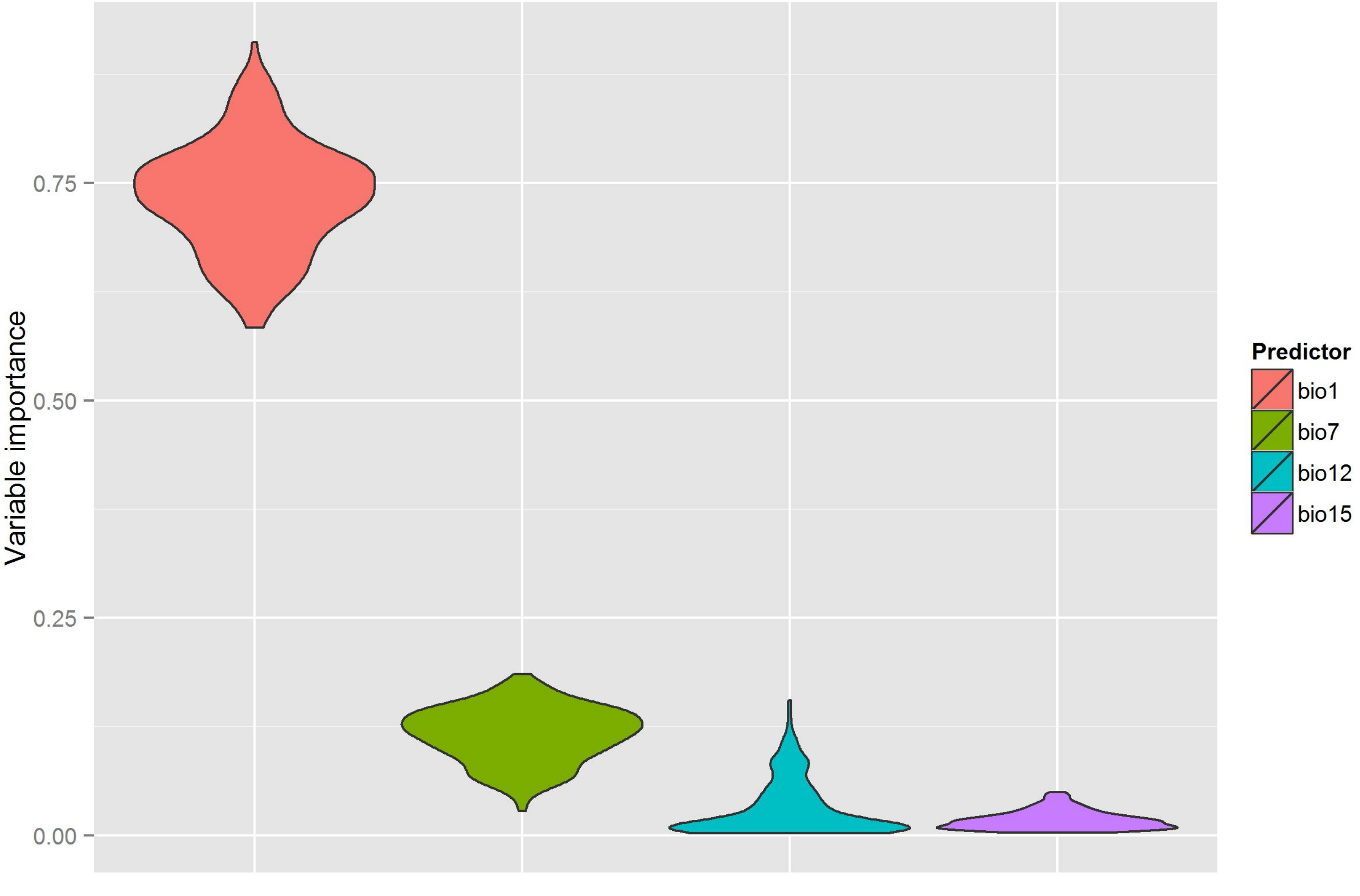

S3: VARIABLE IMPORTANCE GRAPHS
REDUCED MODEL RELIABILITY SPECIES

Apapane GBM variable importance violin plot

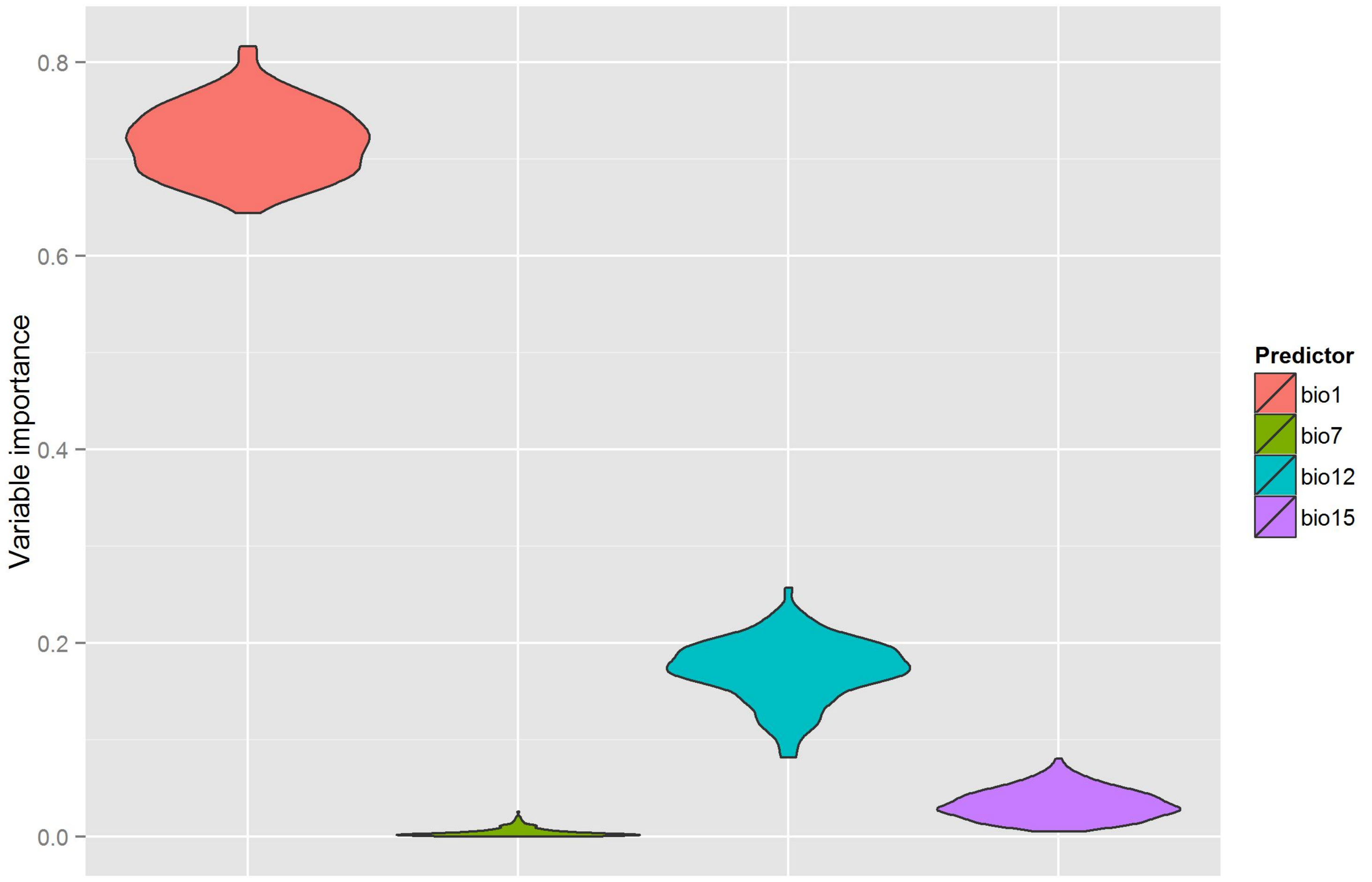

S3: VARIABLE IMPORTANCE GRAPHS
REDUCED MODEL RELIABILITY SPECIES

Apapane MAXENT variable importance violin plot

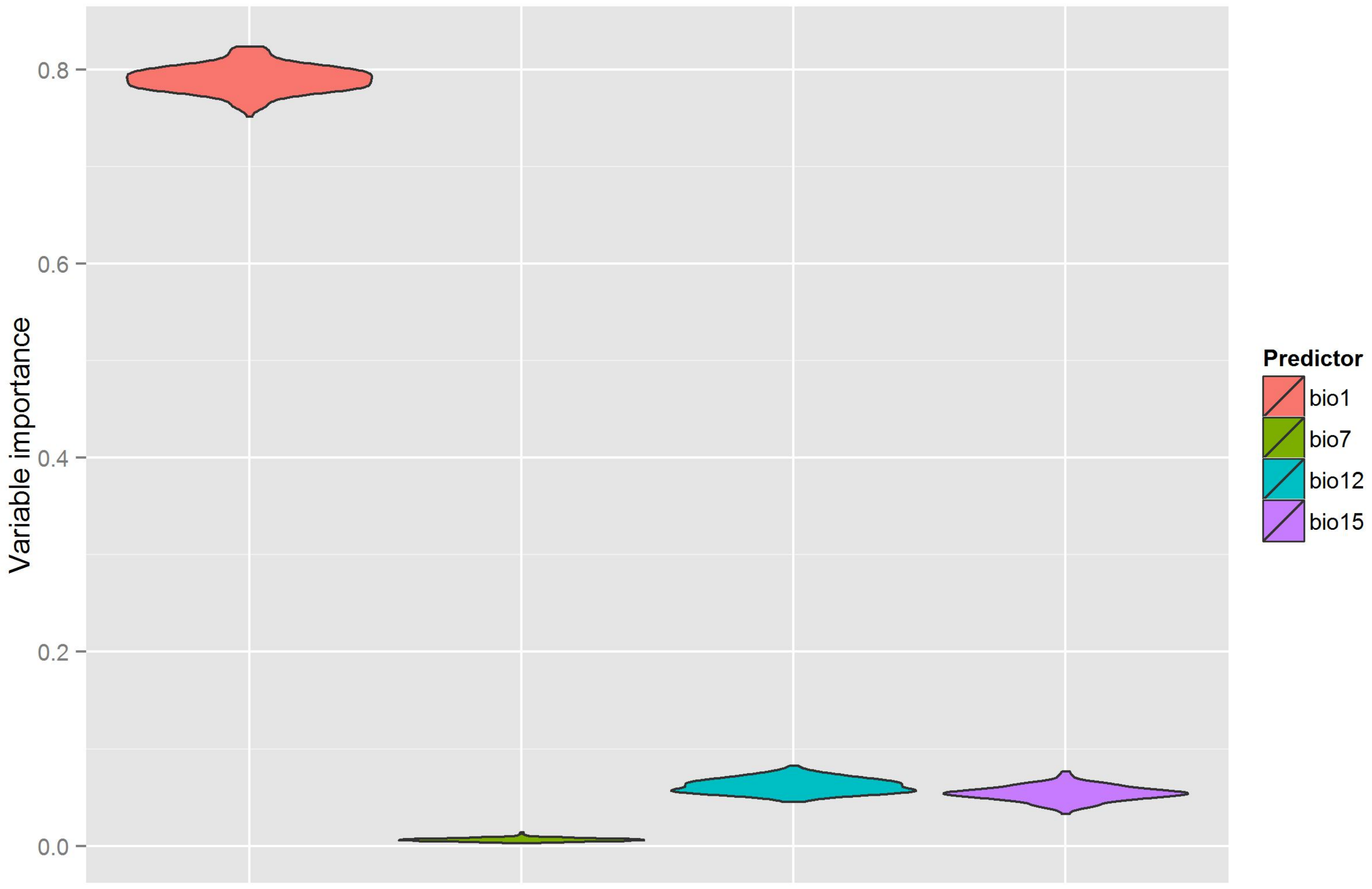

S3: VARIABLE IMPORTANCE GRAPHS
REDUCED MODEL RELIABILITY SPECIES

Hawaii Amakihi GBM variable importance violin plot

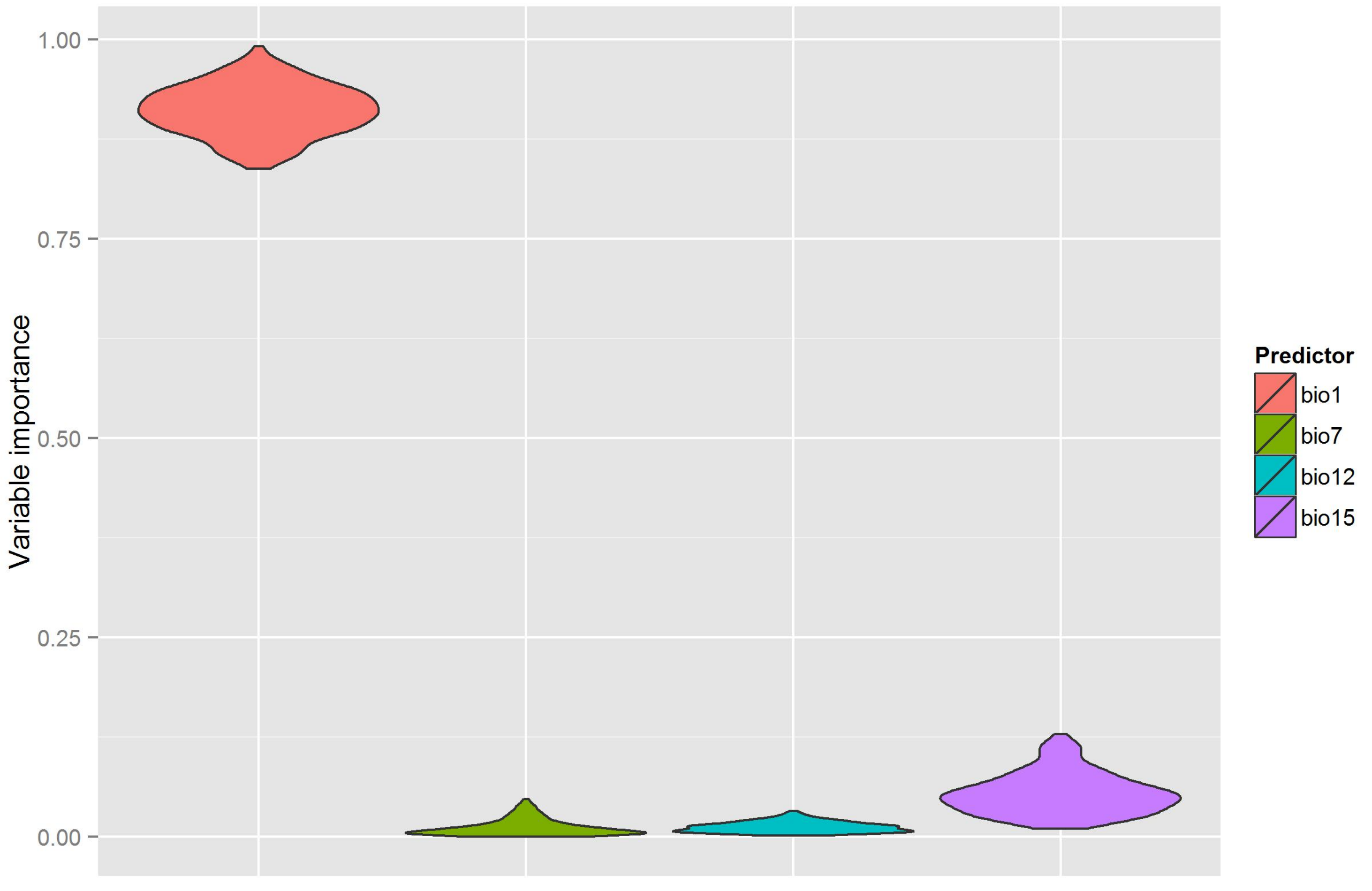

S3: VARIABLE IMPORTANCE GRAPHS
REDUCED MODEL RELIABILITY SPECIES

Hawaii Amakihi MAXENT variable importance violin plot

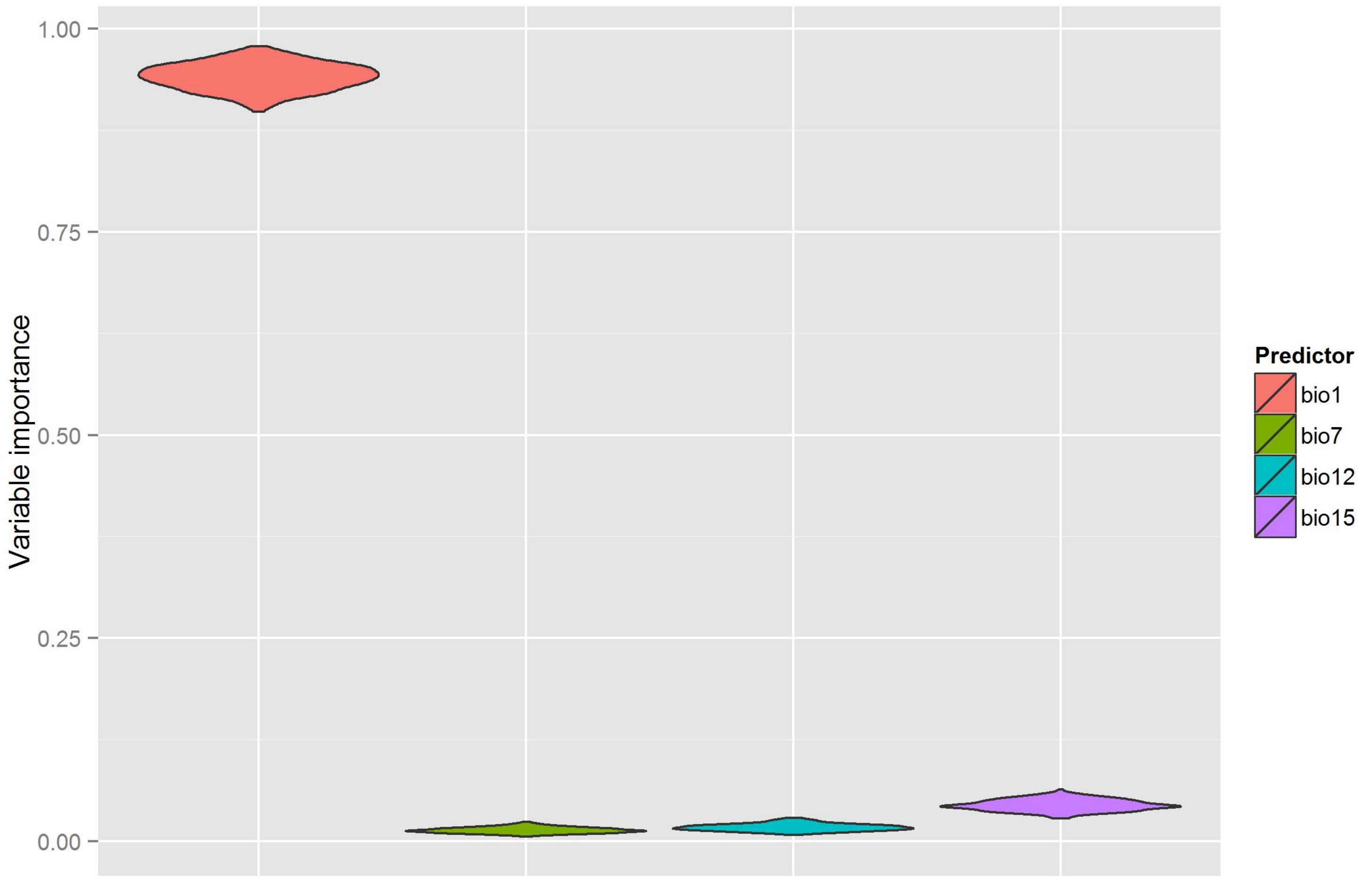

S3: VARIABLE IMPORTANCE GRAPHS
REDUCED MODEL RELIABILITY SPECIES

Hawaii Elepaio GBM variable importance violin plot

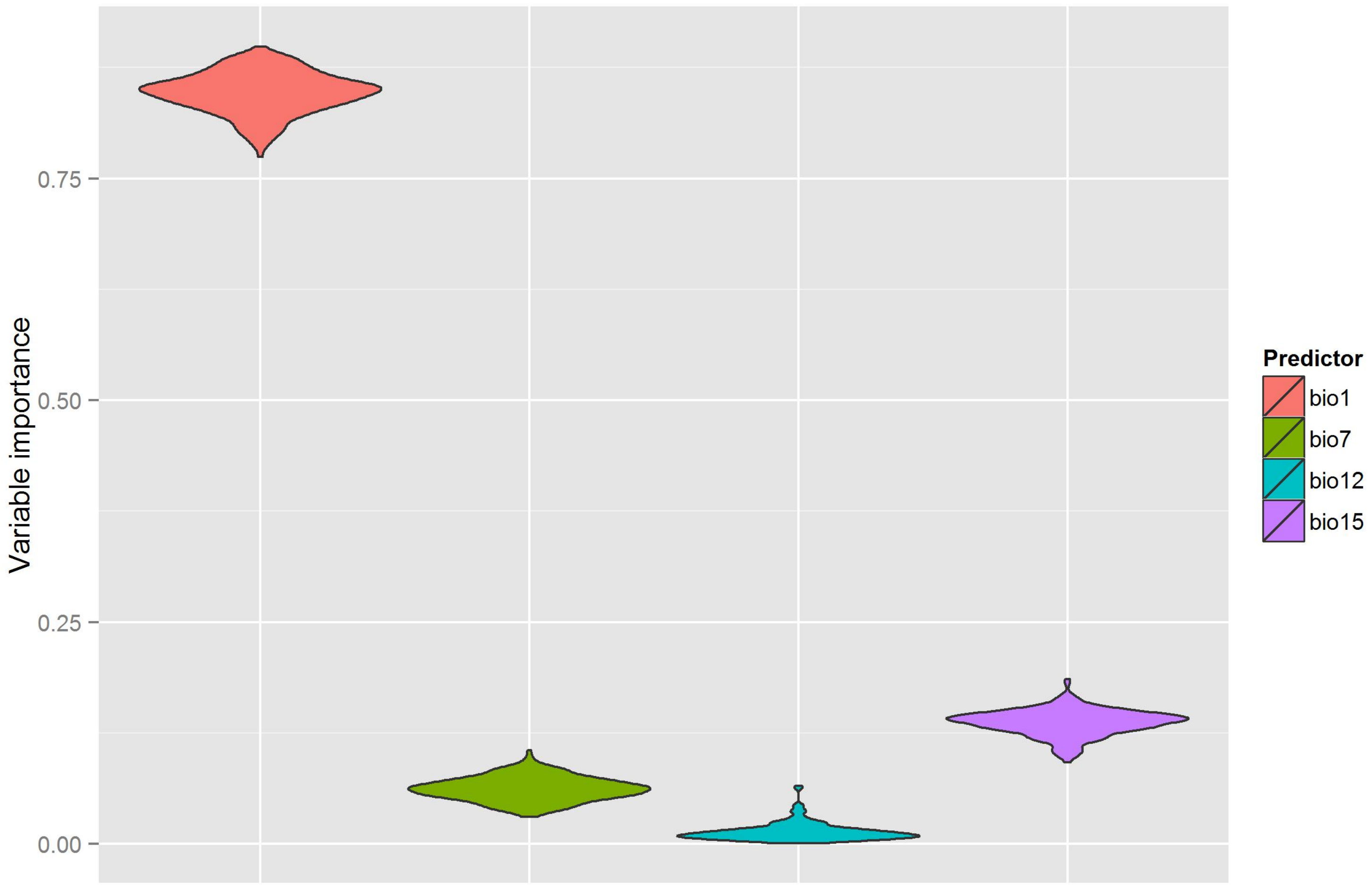

S3: VARIABLE IMPORTANCE GRAPHS
REDUCED MODEL RELIABILITY SPECIES

Hawaii Elepaio MAXENT variable importance violin plot

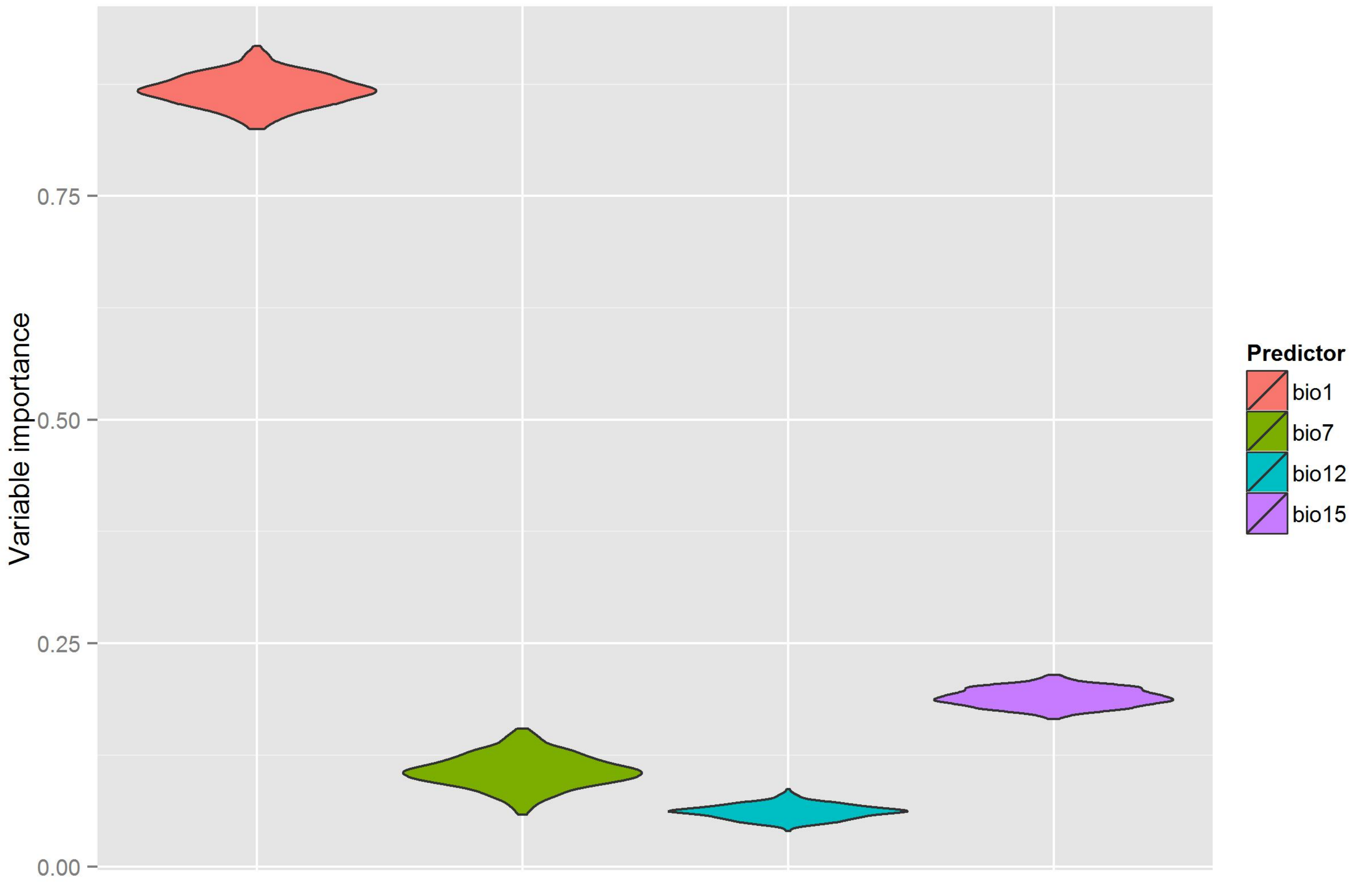

S3: VARIABLE IMPORTANCE GRAPHS
REDUCED MODEL RELIABILITY SPECIES

Kauai Amakihi GBM variable importance violin plot

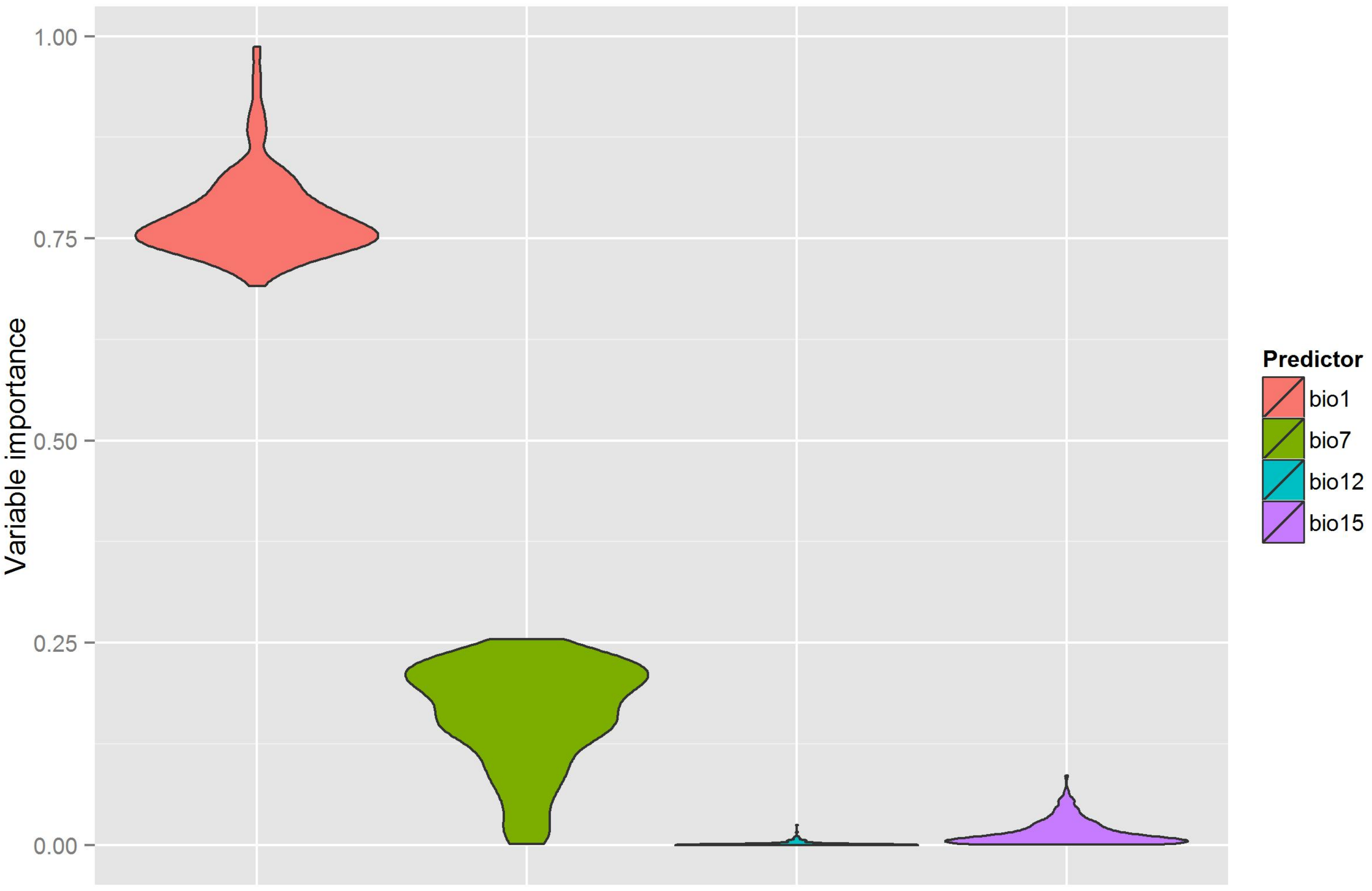

S3: VARIABLE IMPORTANCE GRAPHS
REDUCED MODEL RELIABILITY SPECIES

Kauai Amakihi MAXENT variable importance violin plot

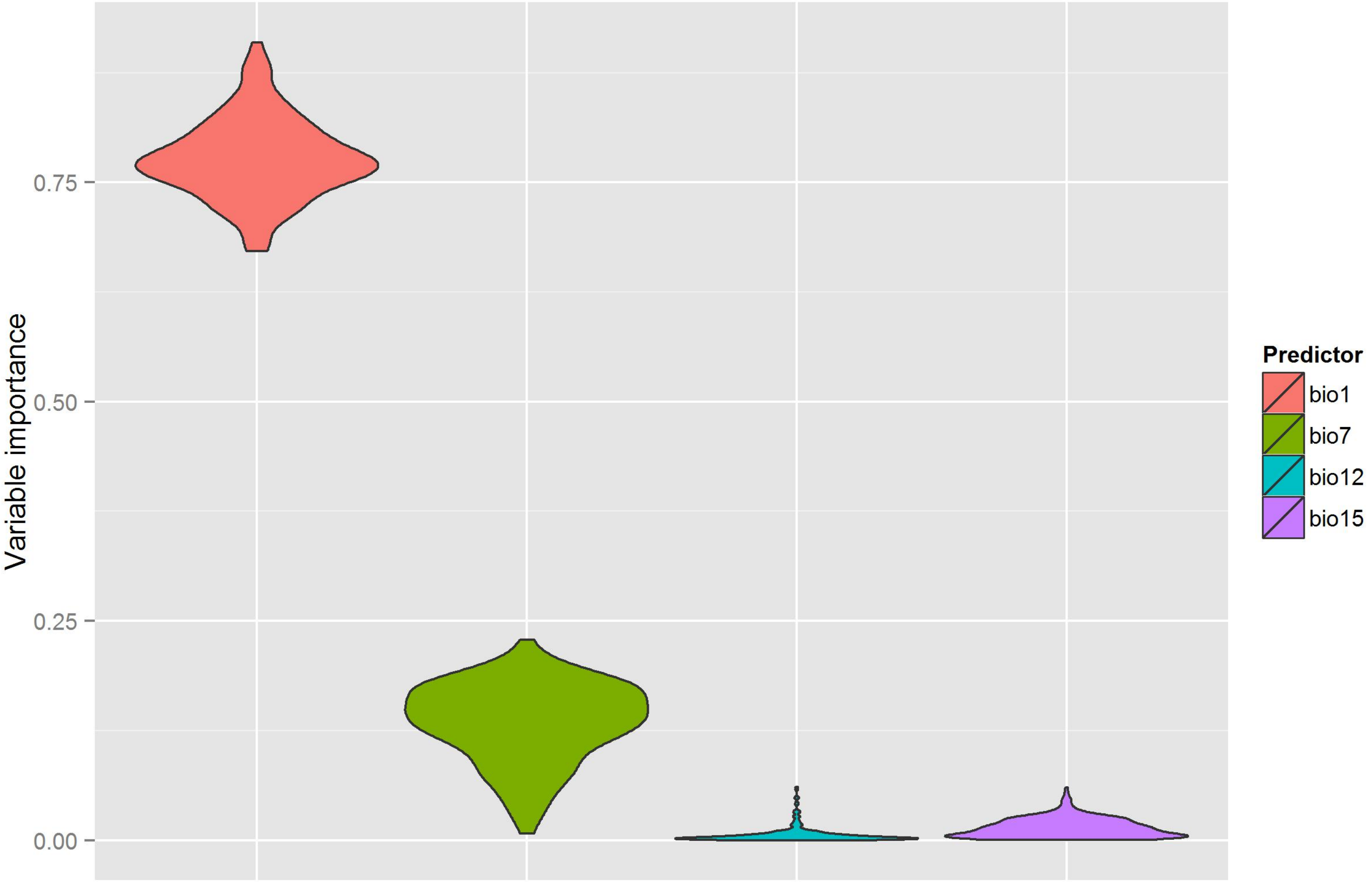

S3: VARIABLE IMPORTANCE GRAPHS
REDUCED MODEL RELIABILITY SPECIES

Kauai Elepaio GBM variable importance violin plot

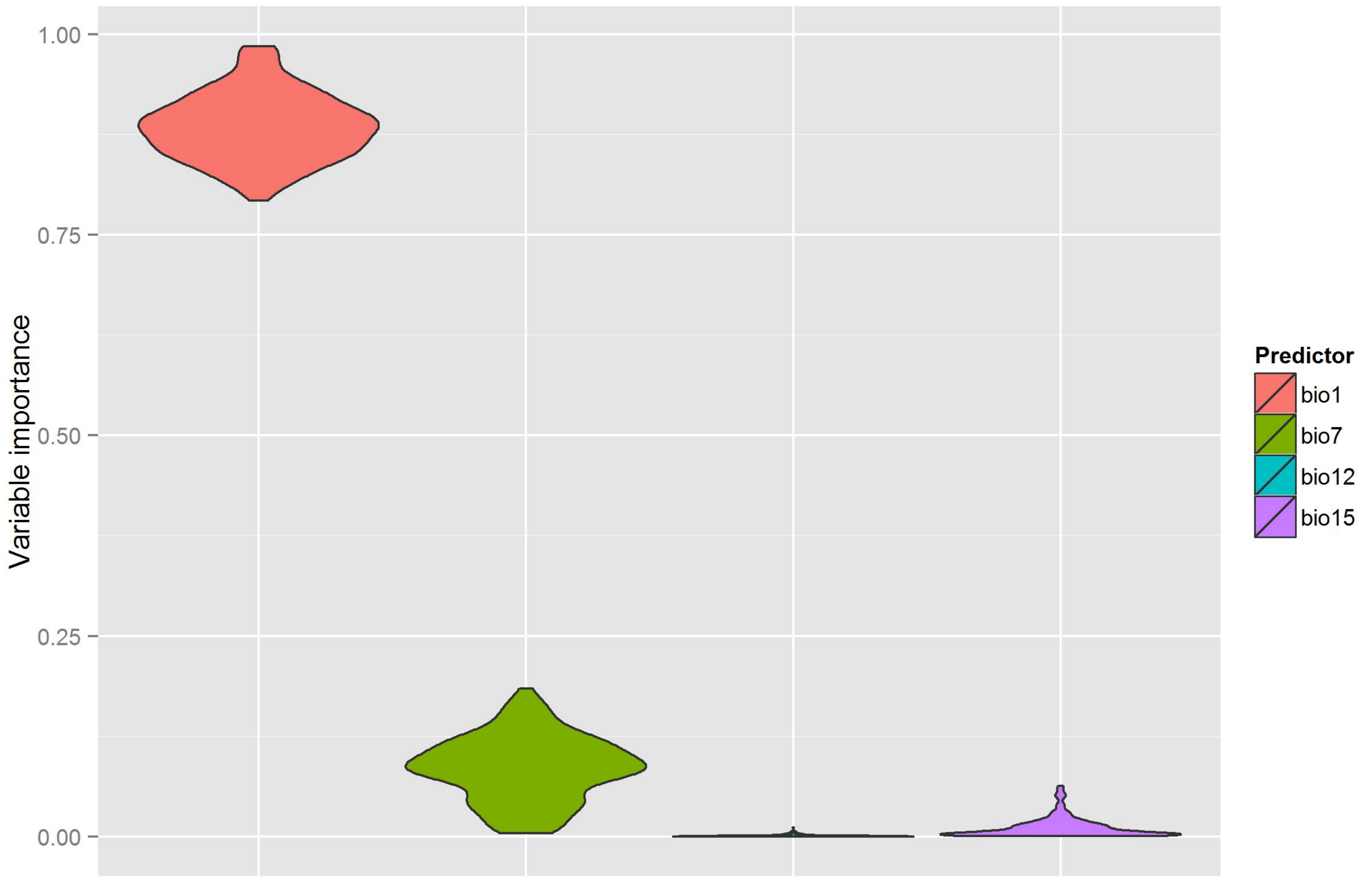

S3: VARIABLE IMPORTANCE GRAPHS
REDUCED MODEL RELIABILITY SPECIES

Kauai Elepaio MAXENT variable importance violin plot

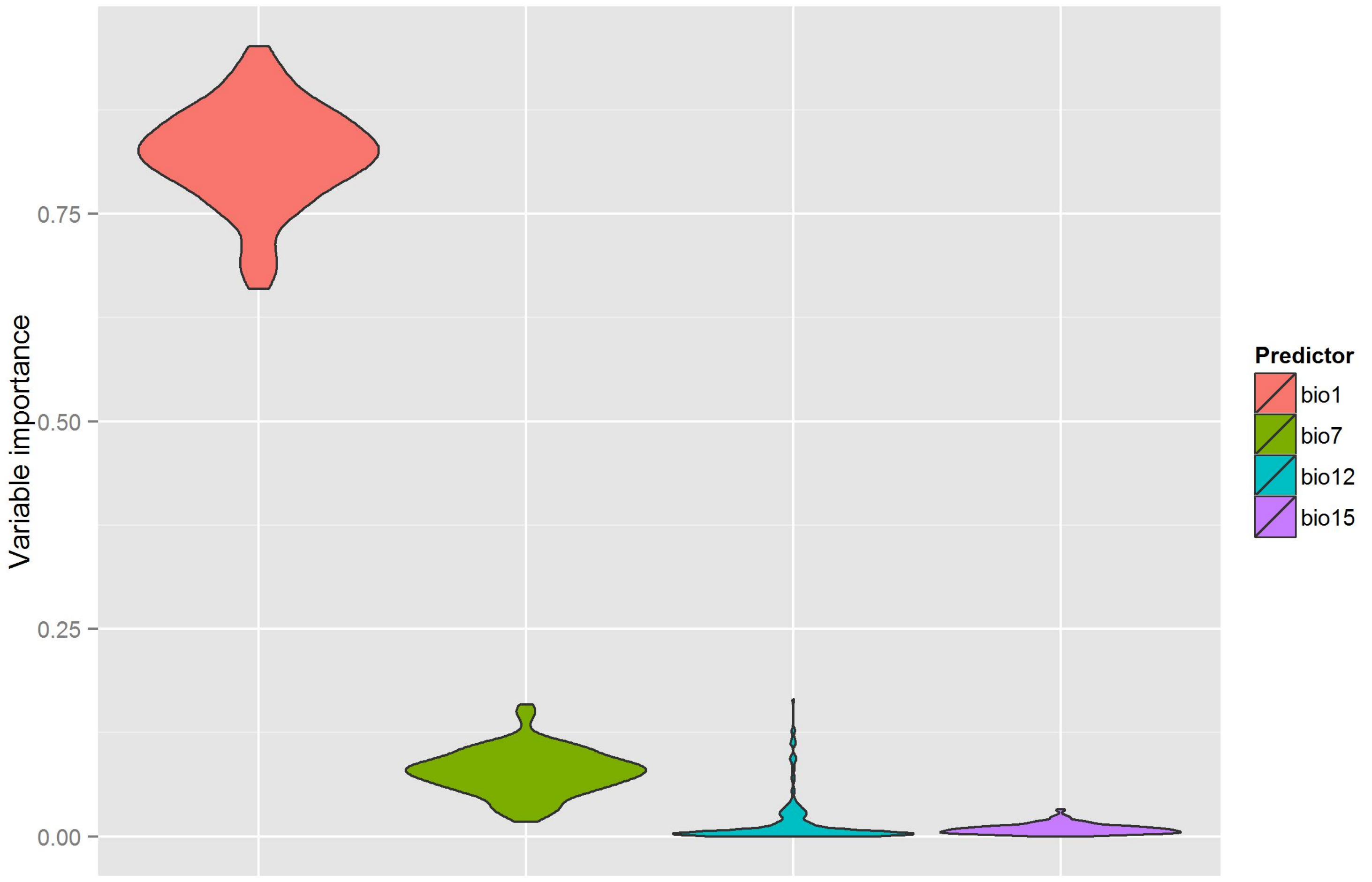

S3: VARIABLE IMPORTANCE GRAPHS
REDUCED MODEL RELIABILITY SPECIES

Oahu Amakihi GBM variable importance violin plot

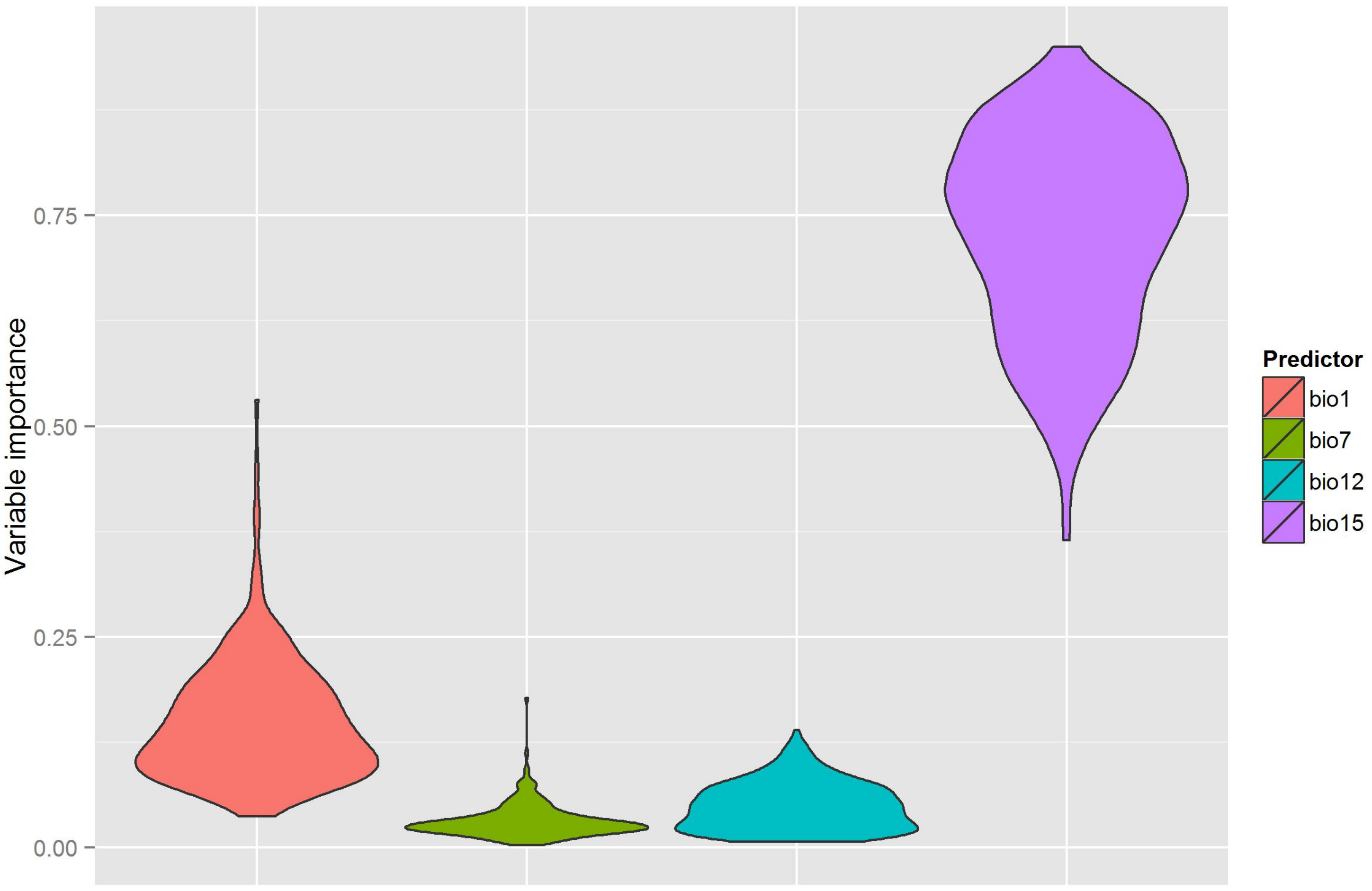

S3: VARIABLE IMPORTANCE GRAPHS
REDUCED MODEL RELIABILITY SPECIES

Oahu Amakihi MAXENT variable importance violin plot

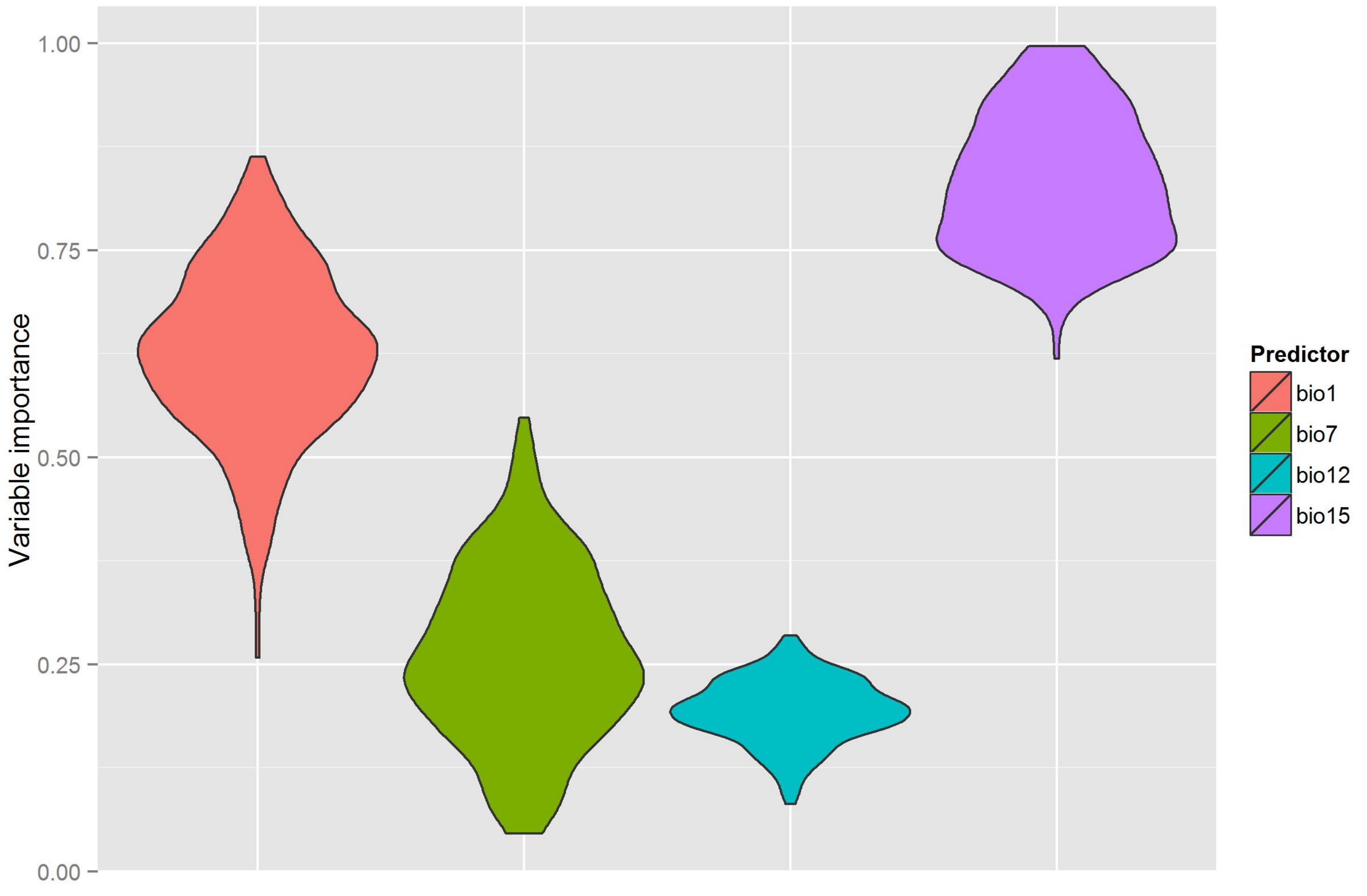

S3: VARIABLE IMPORTANCE GRAPHS
REDUCED MODEL RELIABILITY SPECIES

Oahu Elepaio GBM variable importance violin plot

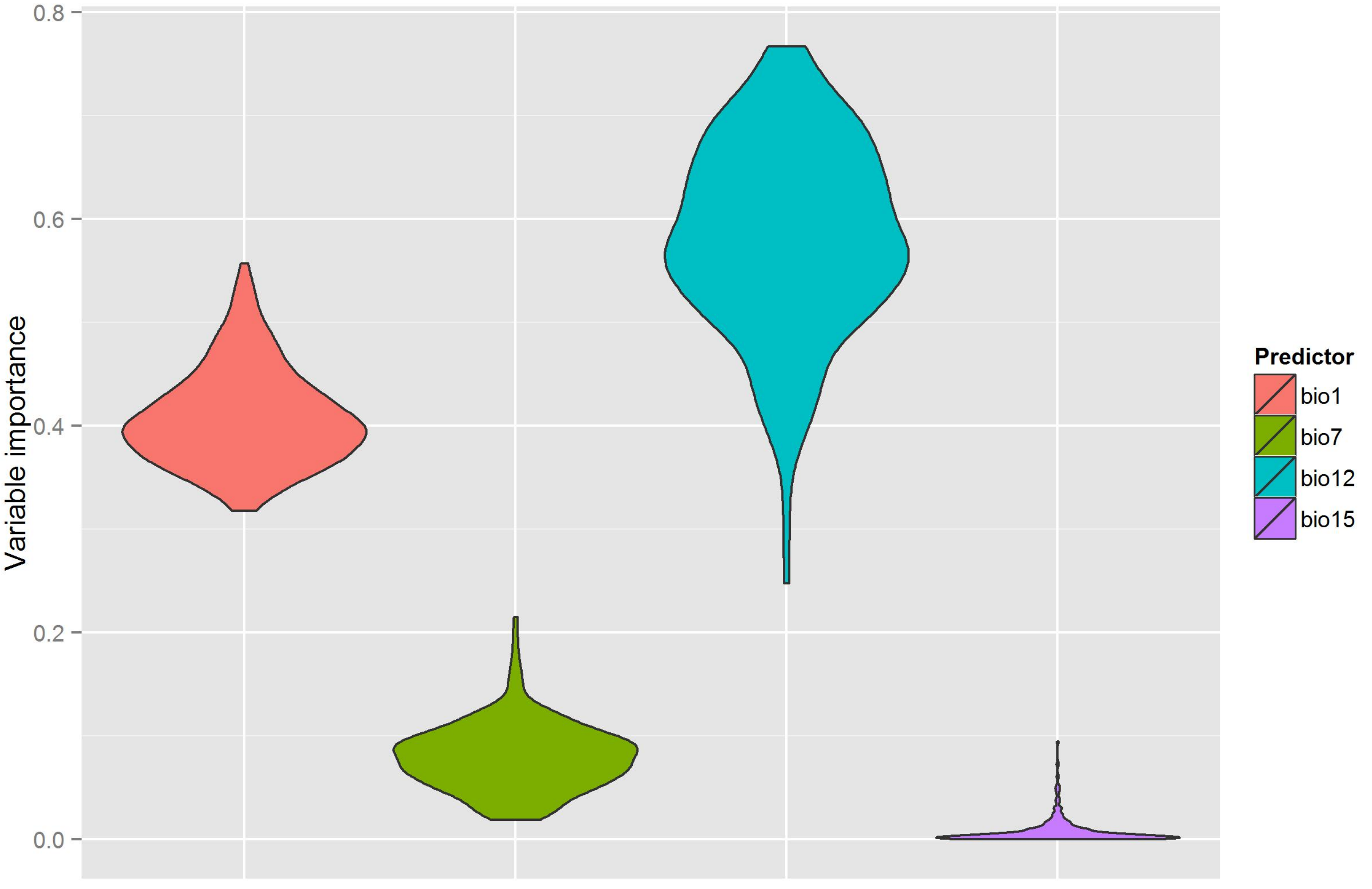

S3: VARIABLE IMPORTANCE GRAPHS
REDUCED MODEL RELIABILITY SPECIES

Oahu Elepaio MAXENT variable importance violin plot

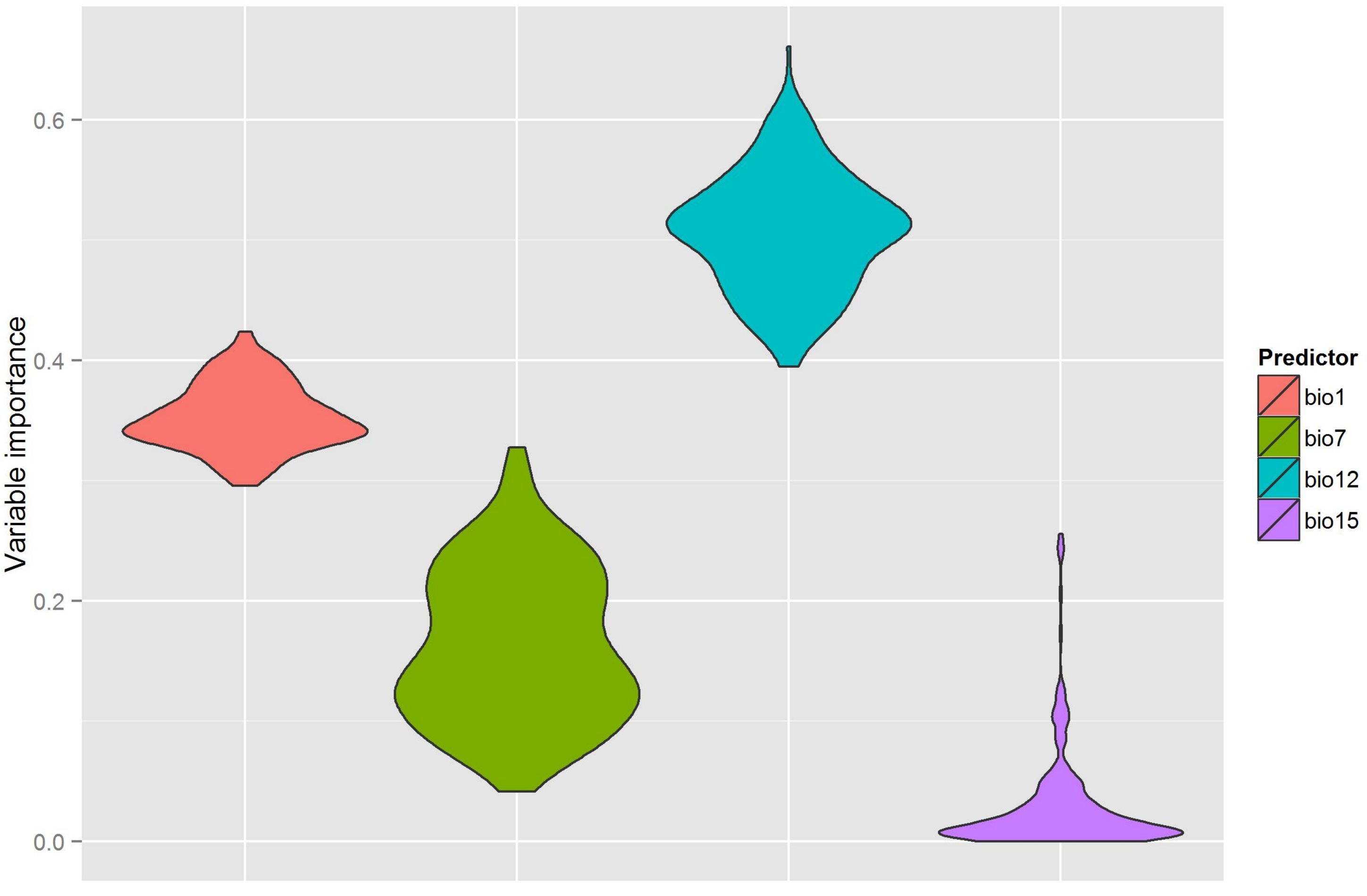

S3: VARIABLE IMPORTANCE GRAPHS
REDUCED MODEL RELIABILITY SPECIES

Omao GBM variable importance violin plot

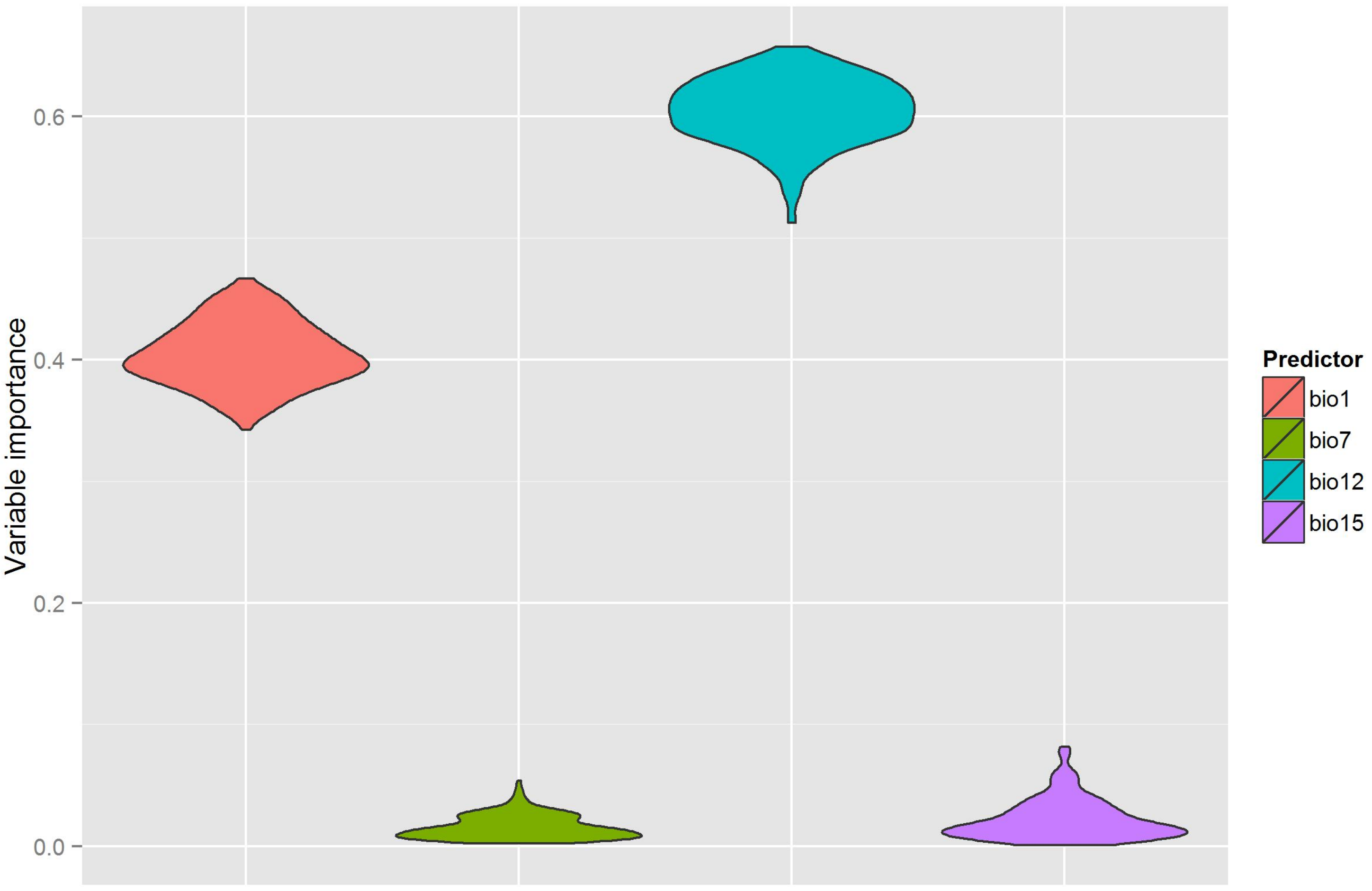

S3: VARIABLE IMPORTANCE GRAPHS
REDUCED MODEL RELIABILITY SPECIES

Omao MAXENT variable importance violin plot

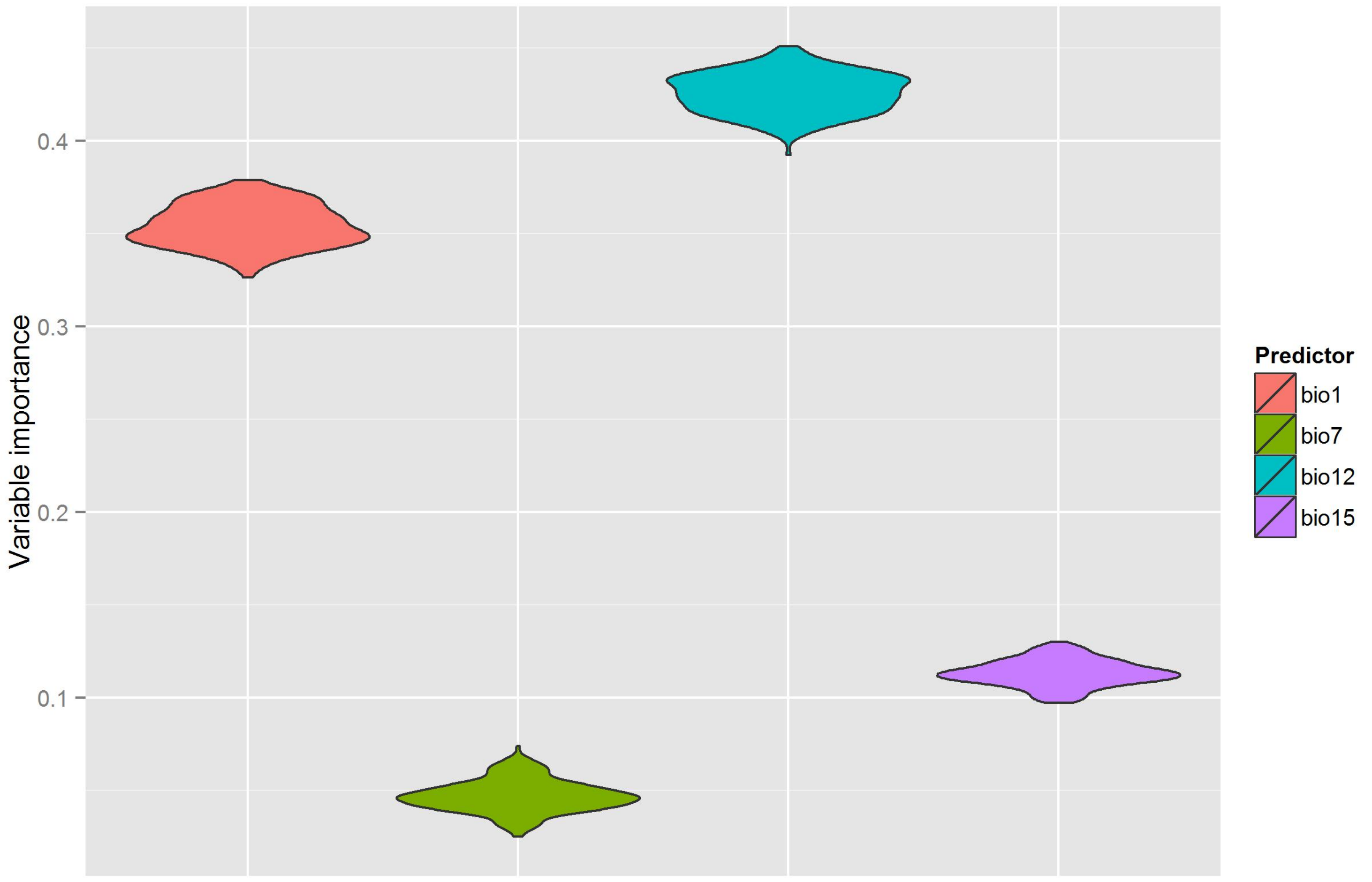

S3: VARIABLE IMPORTANCE GRAPHS
REDUCED MODEL RELIABILITY SPECIES

Palila GBM variable importance violin plot

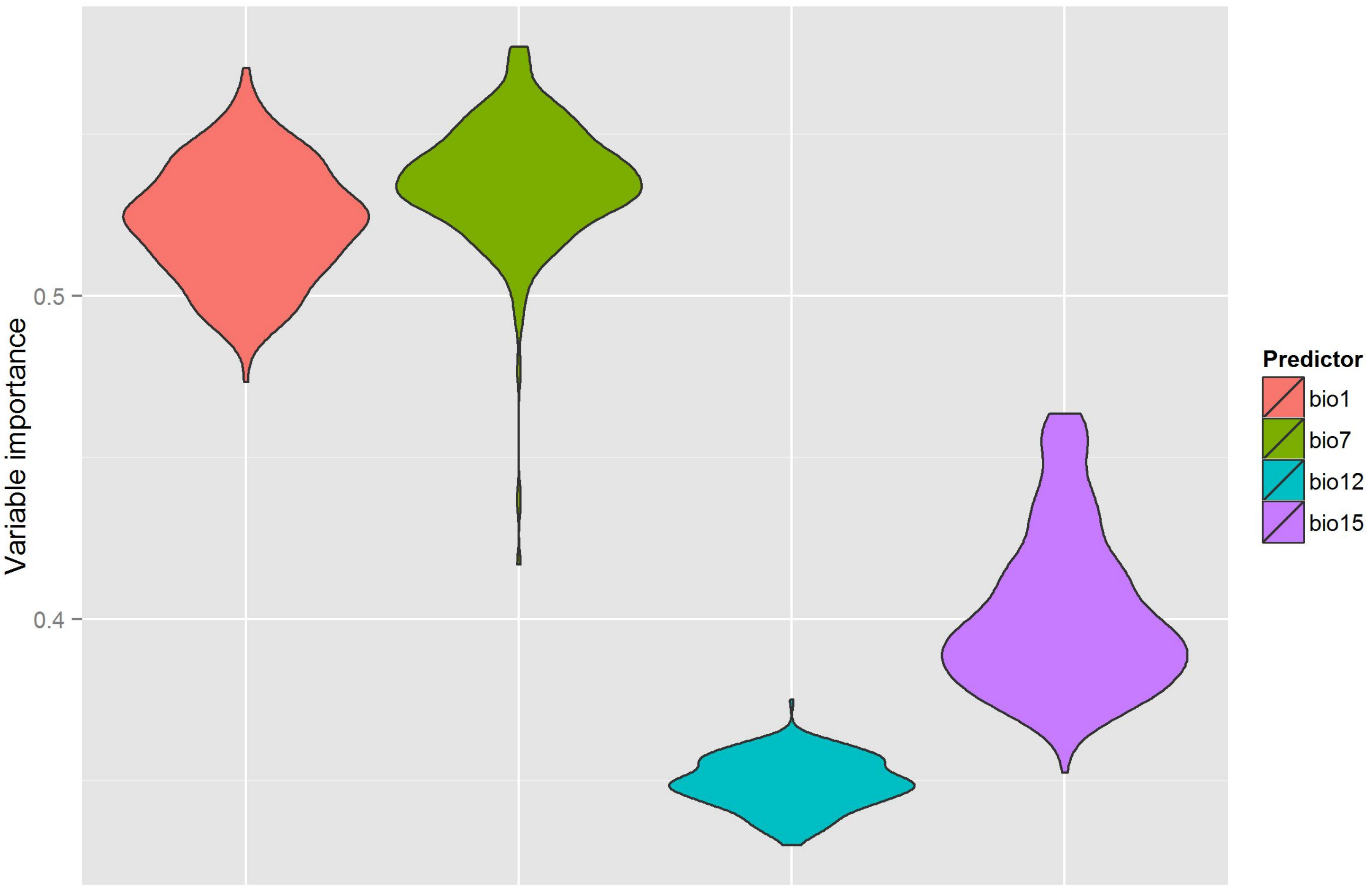

S3: VARIABLE IMPORTANCE GRAPHS
REDUCED MODEL RELIABILITY SPECIES

Palila MAXENT variable importance violin plot

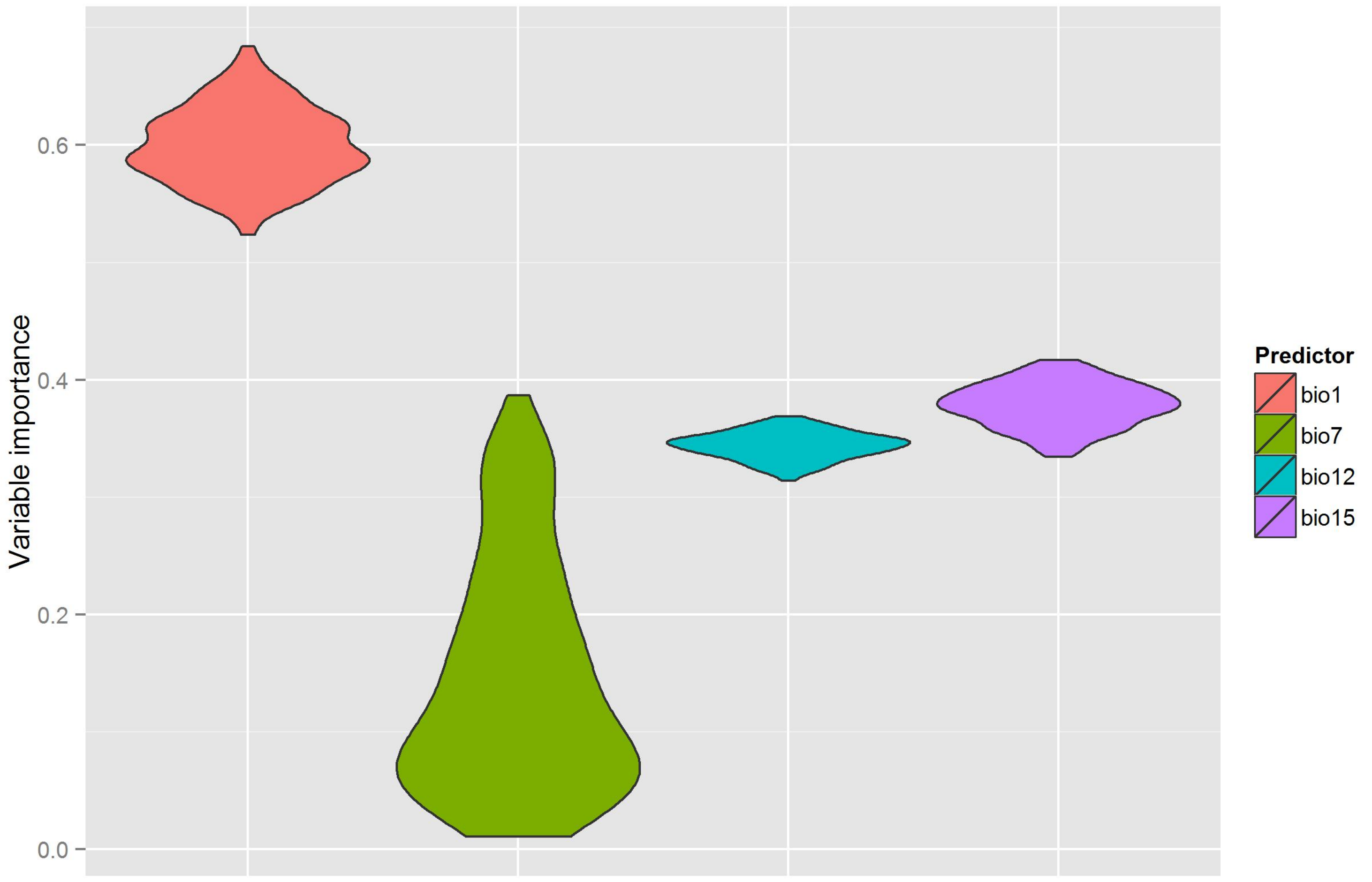

Supplement: S3 File — (PDF) [file pone.0140389.s003.pdf]
