# Supplementary material for: Large-Scale Range Collapse of Hawaiian Forest Birds under Climate Change and the Need 21st Century Conservation Options
Source: PLoS One. 2015 Oct 28;10(10):e0140389. doi: 10.1371/journal.pone.0140389 (PMC4625087; doi:10.1371/journal.pone.0140389)

Appendix S4. Agreement between expert-derived and modeled current climate-based ranges for all Hawaiian forest bird species.

The table and graph below quantify the amount of agreement between the modeled and expert curent ranges.

Maps show the agreement between our modeled current range without subtraction of areas beyond primary habitat for each extant Hawaiian forest bird species. Green cells indicate modelled current range. Polygons bounded by a red line indicate the extent of expert defined range.

Species	Agreement	Model reliability
\`Akekee	0.97	High
\`Akiapōlā\`au	0.97	High
\`Akikiki	0.99	High
\`Ākohekohe	0.99	High
Hawai\`i \`ākepa	0.97	High
Hawai\`i creeper	0.96	High
\`I\`iwi	0.92	High
Maui \`alauahio	0.96	High
Maui parrotbill	0.97	High
Puaiohi	0.98	High
\`Anianiau	0.98	Reduced
\`Apapane	0.86	Reduced
Hawai\`i \`amakihi	0.80	Reduced
Hawai\`i \`elepaio	0.84	Reduced
Kauai \`amakihi	0.88	Reduced
Kauai \`elepaio	0.88	Reduced
O\`ahu \`amakihi	0.91	Reduced
O\`ahu \`elepaio	0.82	Reduced
\`Oma\`o	0.86	Reduced
Palila	0.95	Reduced

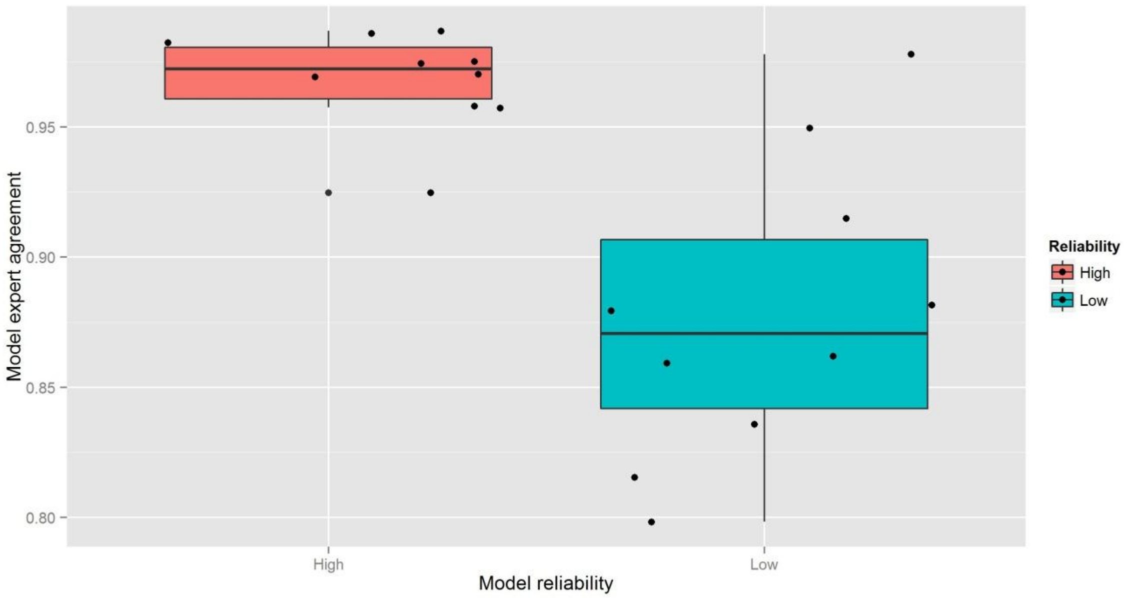

Model expert agreement
0.95
0.90
0.85
0.80
High
Low
Model reliability
Reliability
High
Low

S4: Agreement between expert-derived ranges and current climate-based ranges
HIGH MODEL RELIABILITY SPECIES

Akekee Expert vs BIN baseline ROC

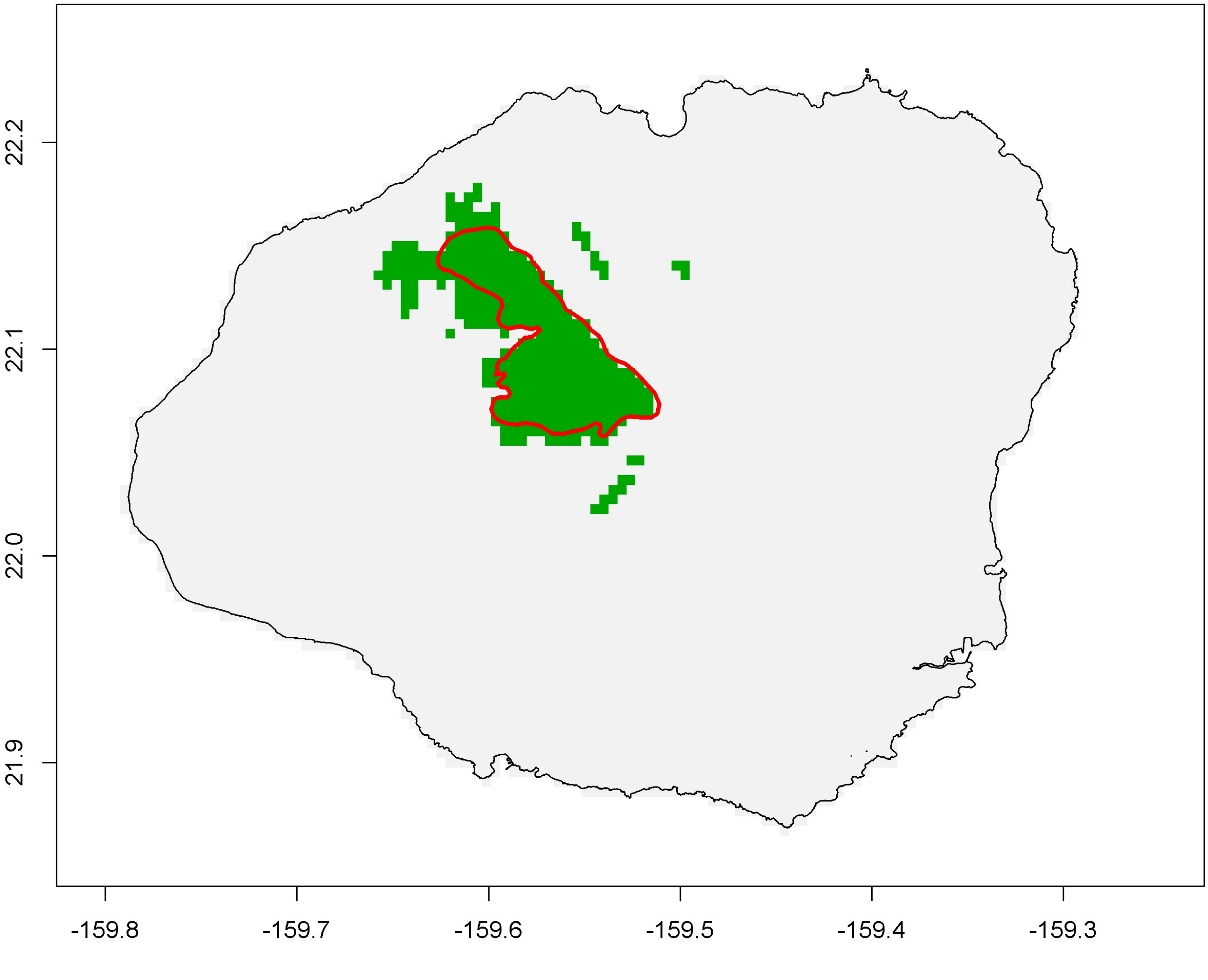

S4: Agreement between expert-derived ranges and current climate-based ranges
HIGH MODEL RELIABILITY SPECIES

Akiapolauu Expert vs BIN baseline ROC

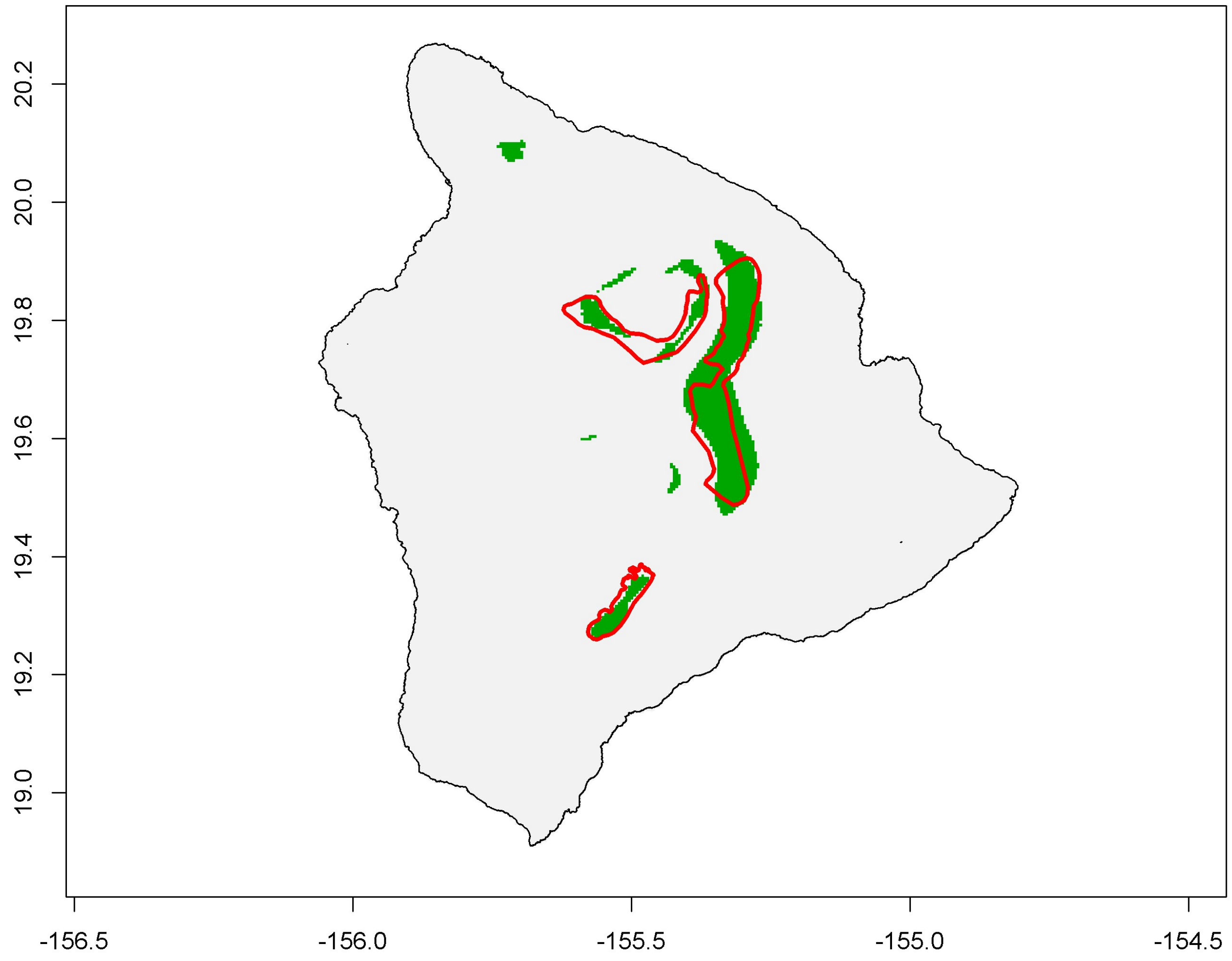

S4: Agreement between expert-derived ranges and current climate-based ranges
HIGH MODEL RELIABILITY SPECIES

Akikiki Expert vs BIN baseline ROC

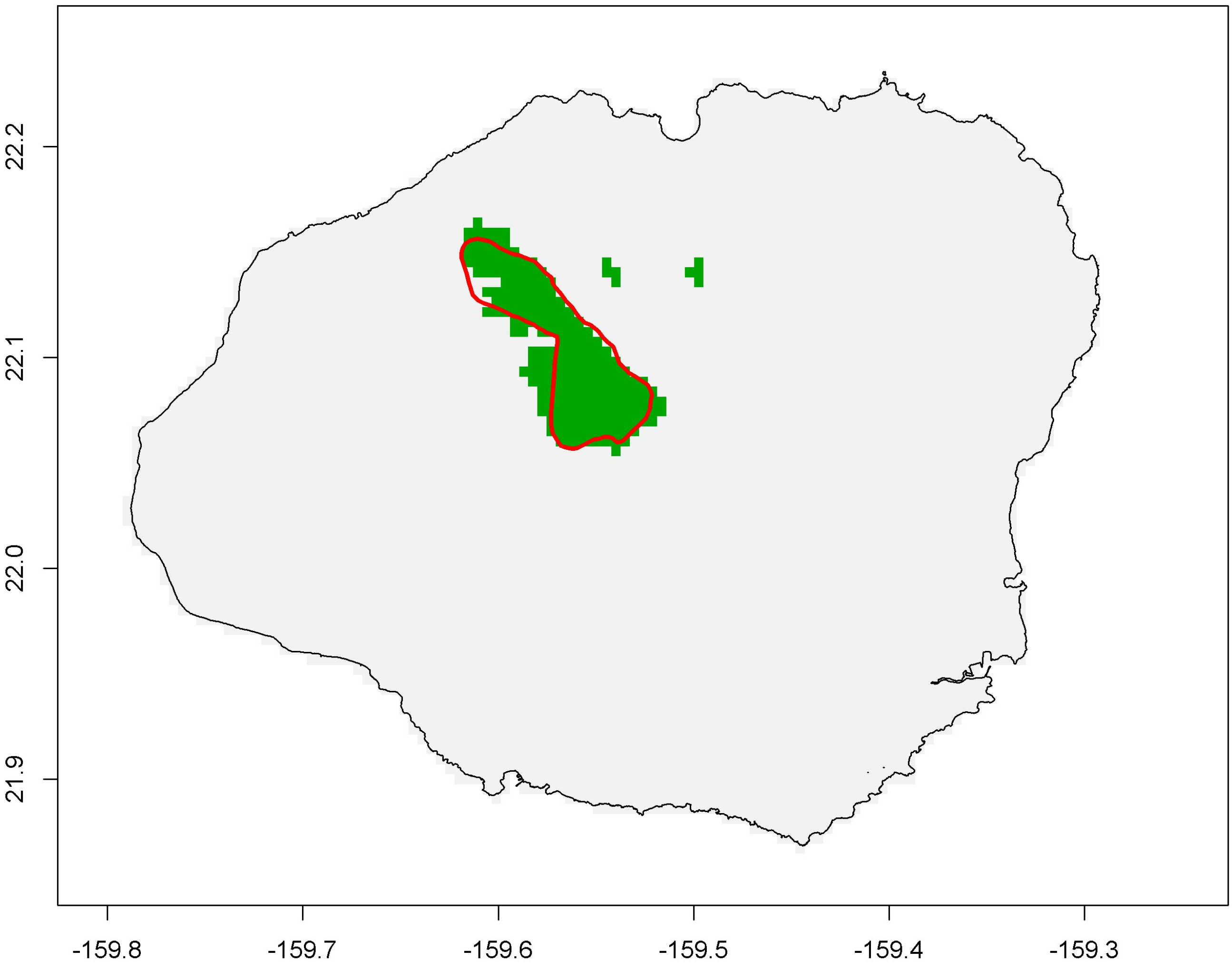

S4: Agreement between expert-derived ranges and current climate-based ranges
HIGH MODEL RELIABILITY SPECIES

Akohekohe Expert vs BIN baseline ROC

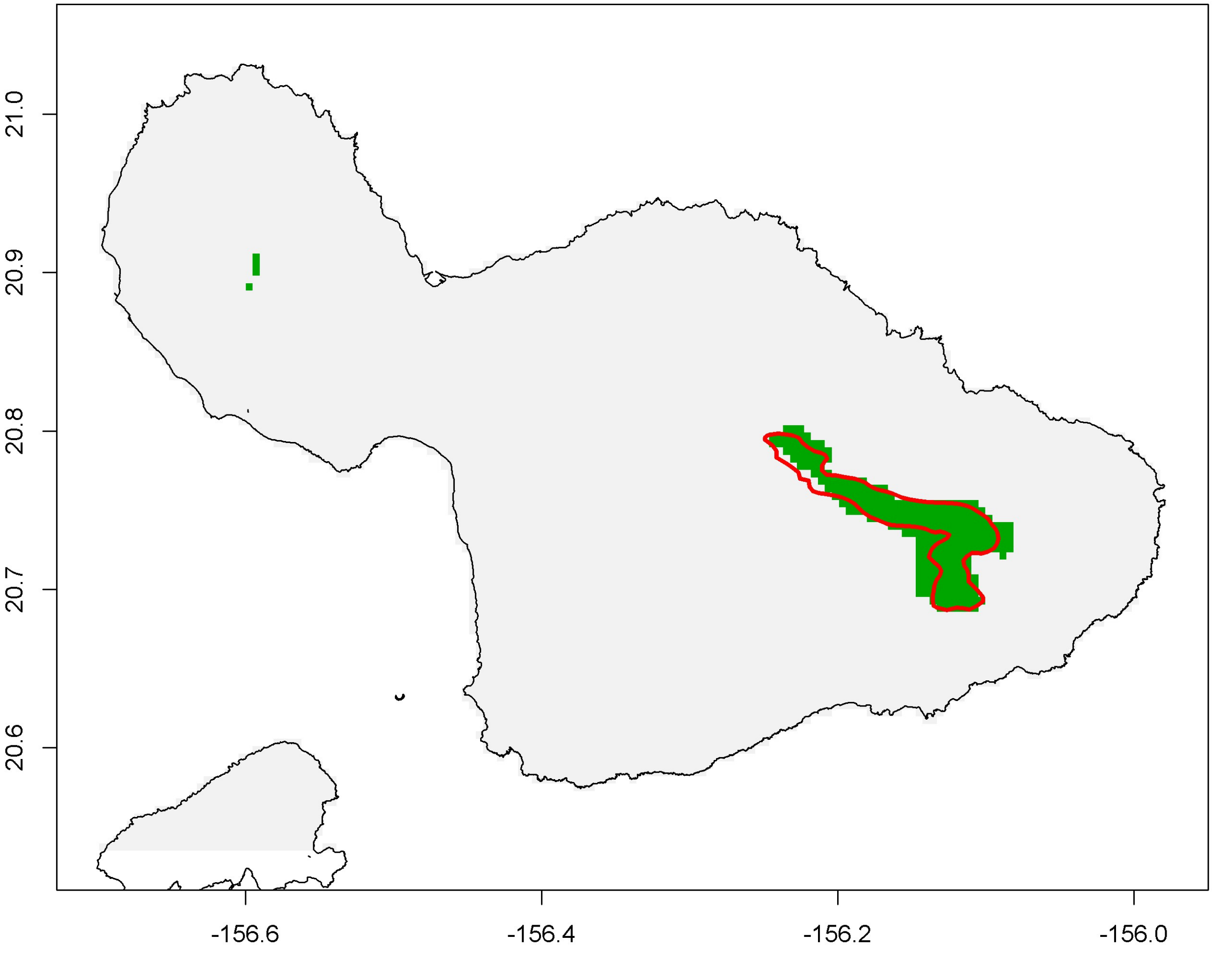

S4: Agreement between expert-derived ranges and current climate-based ranges
HIGH MODEL RELIABILITY SPECIES

Hawaii Akepa Expert vs BIN baseline ROC

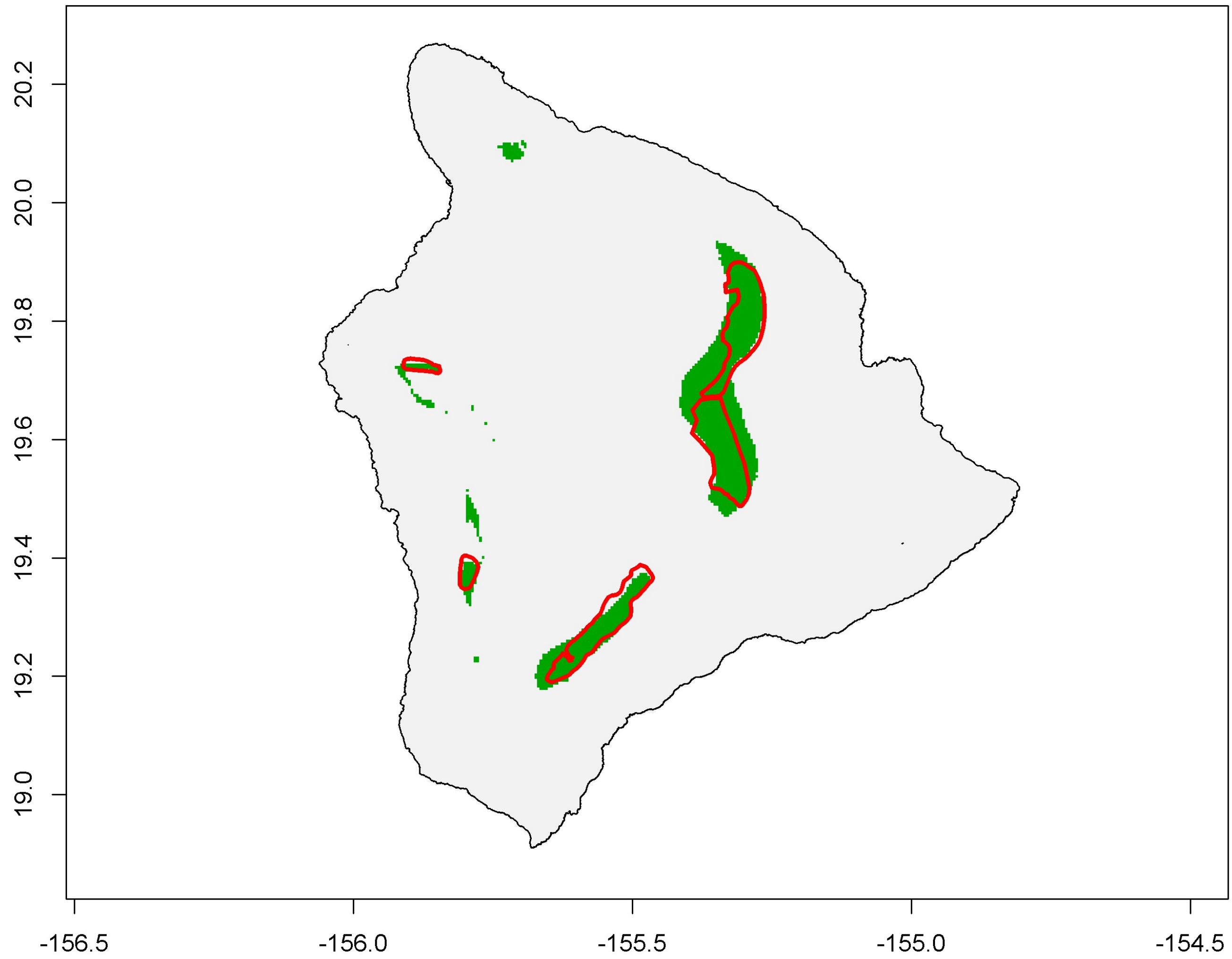

S4: Agreement between expert-derived ranges and current climate-based ranges
HIGH MODEL RELIABILITY SPECIES

Hawaii Creeper Expert vs BIN baseline ROC

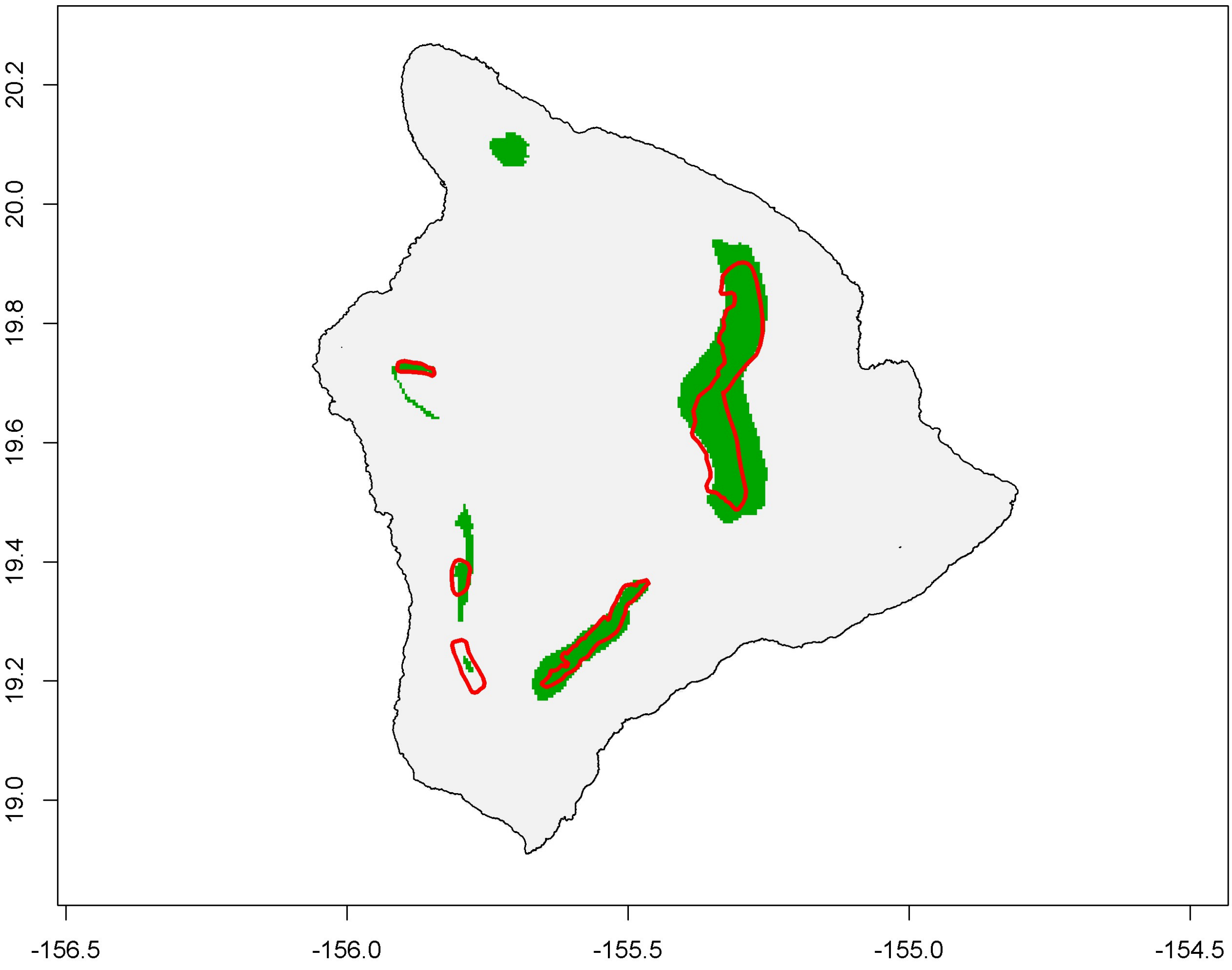

S4: Agreement between expert-derived ranges and current climate-based ranges
HIGH MODEL RELIABILITY SPECIES

Iiwi Expert vs BIN baseline ROC

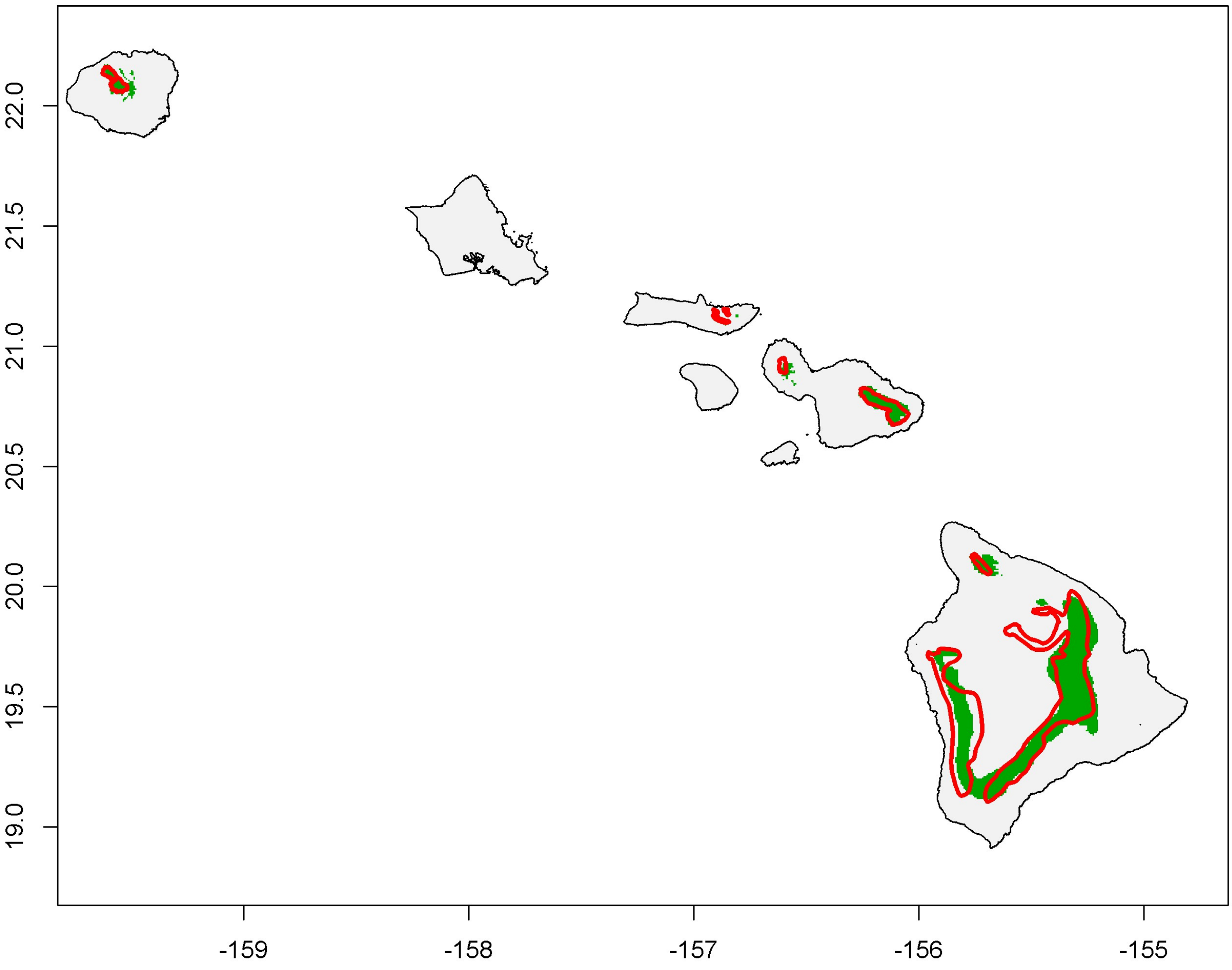

S4: Agreement between expert-derived ranges and current climate-based ranges
HIGH MODEL RELIABILITY SPECIES

Maui Alauahio Expert vs BIN baseline ROC

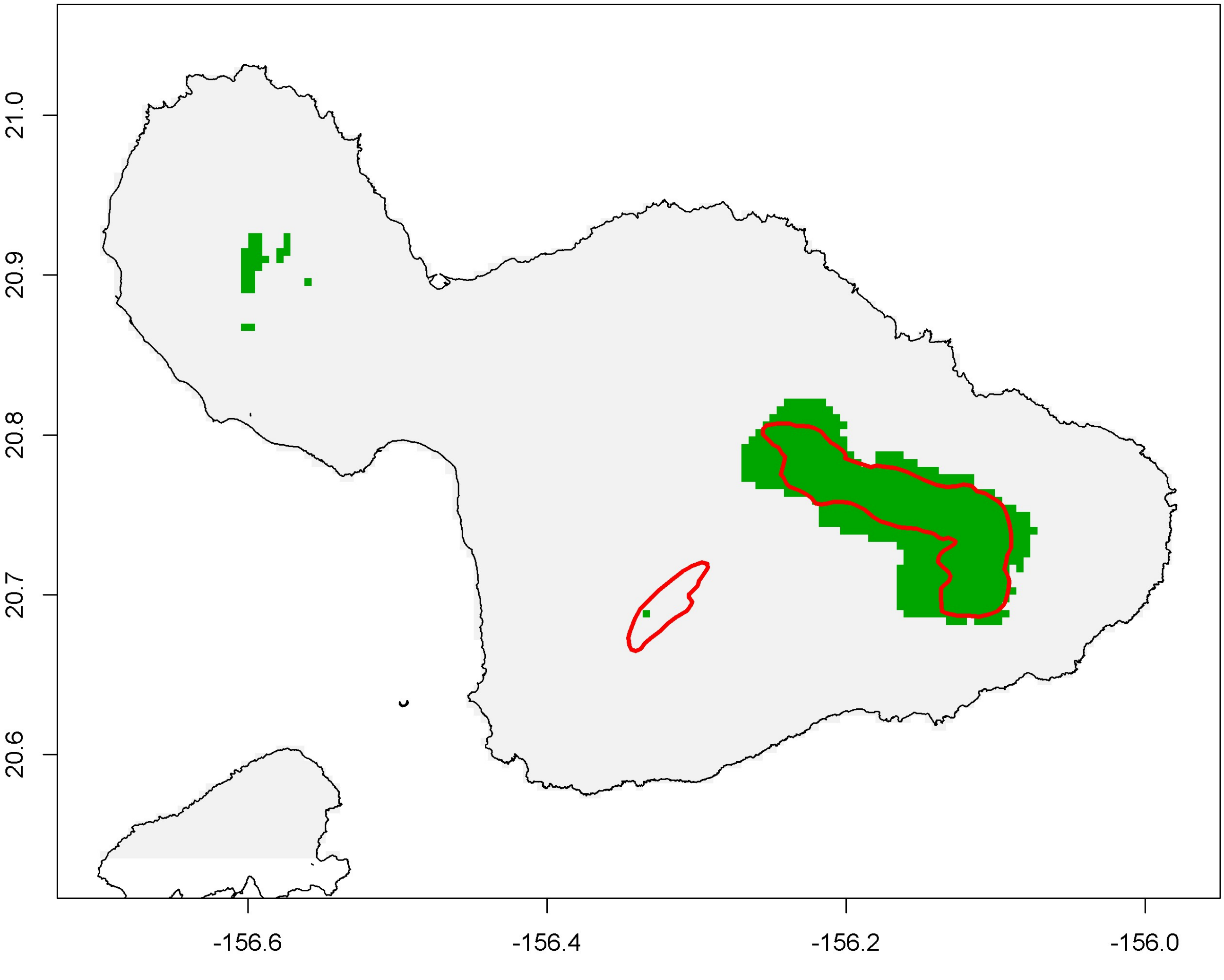

S4: Agreement between expert-derived ranges and current climate-based ranges
HIGH MODEL RELIABILITY SPECIES

Maui Parrotbill Expert vs BIN baseline ROC

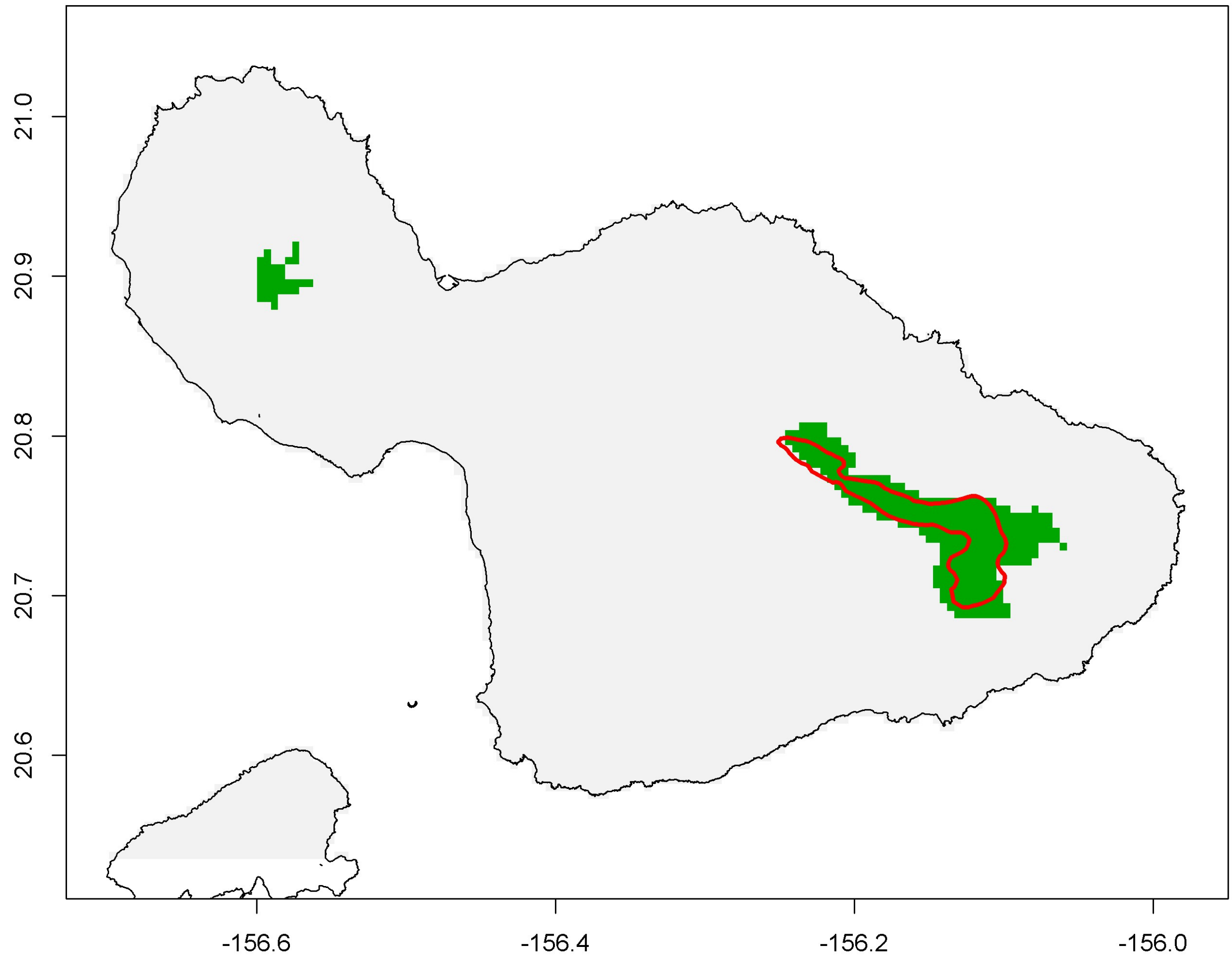

S4: Agreement between expert-derived ranges and current climate-based ranges
HIGH MODEL RELIABILITY SPECIES

Puaiohi Expert vs BIN baseline ROC

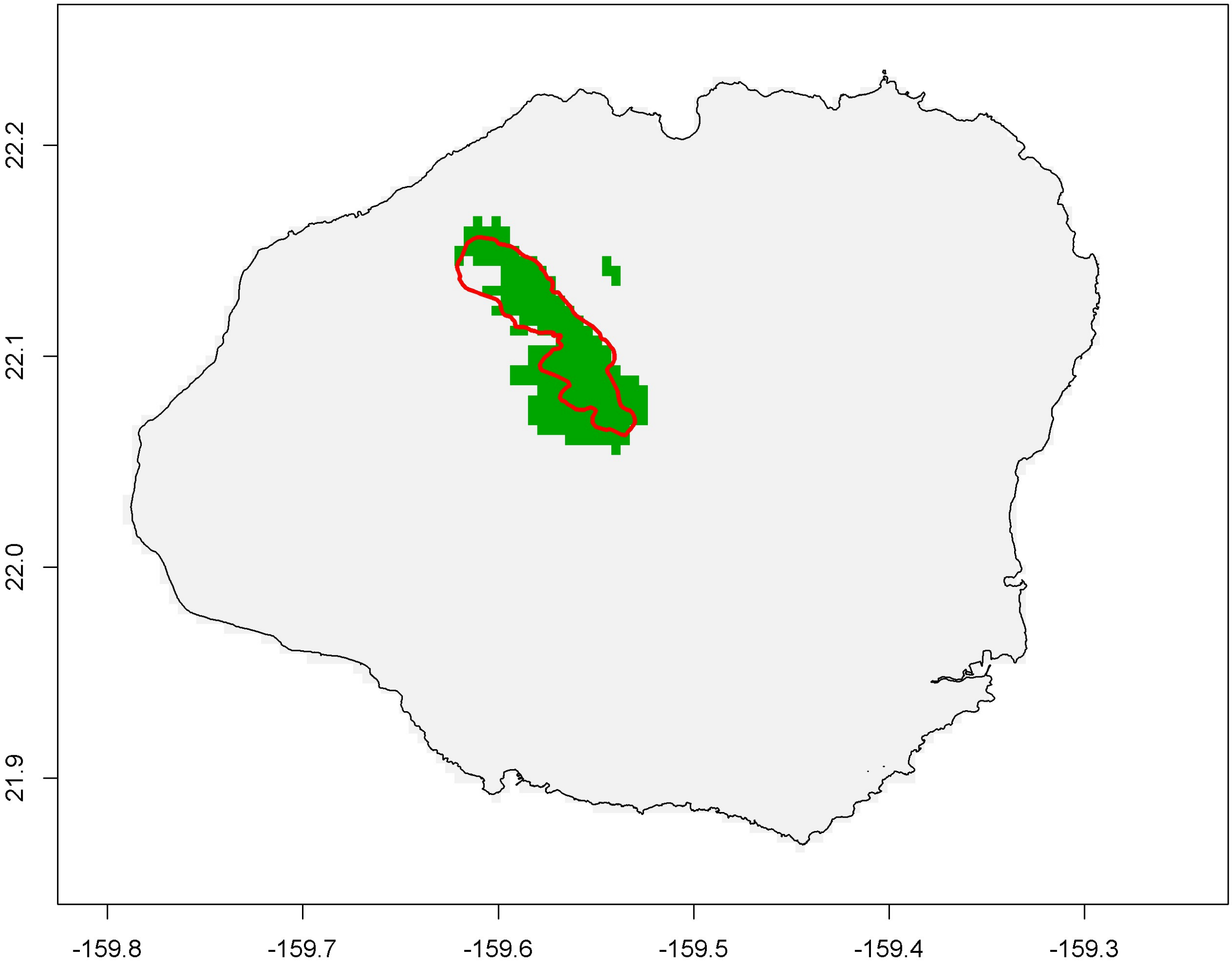

S4: Agreement between expert-derived ranges and current climate-based ranges
REDUCED MODEL RELIABILITY SPECIES

Anianiau Expert vs BIN baseline ROC

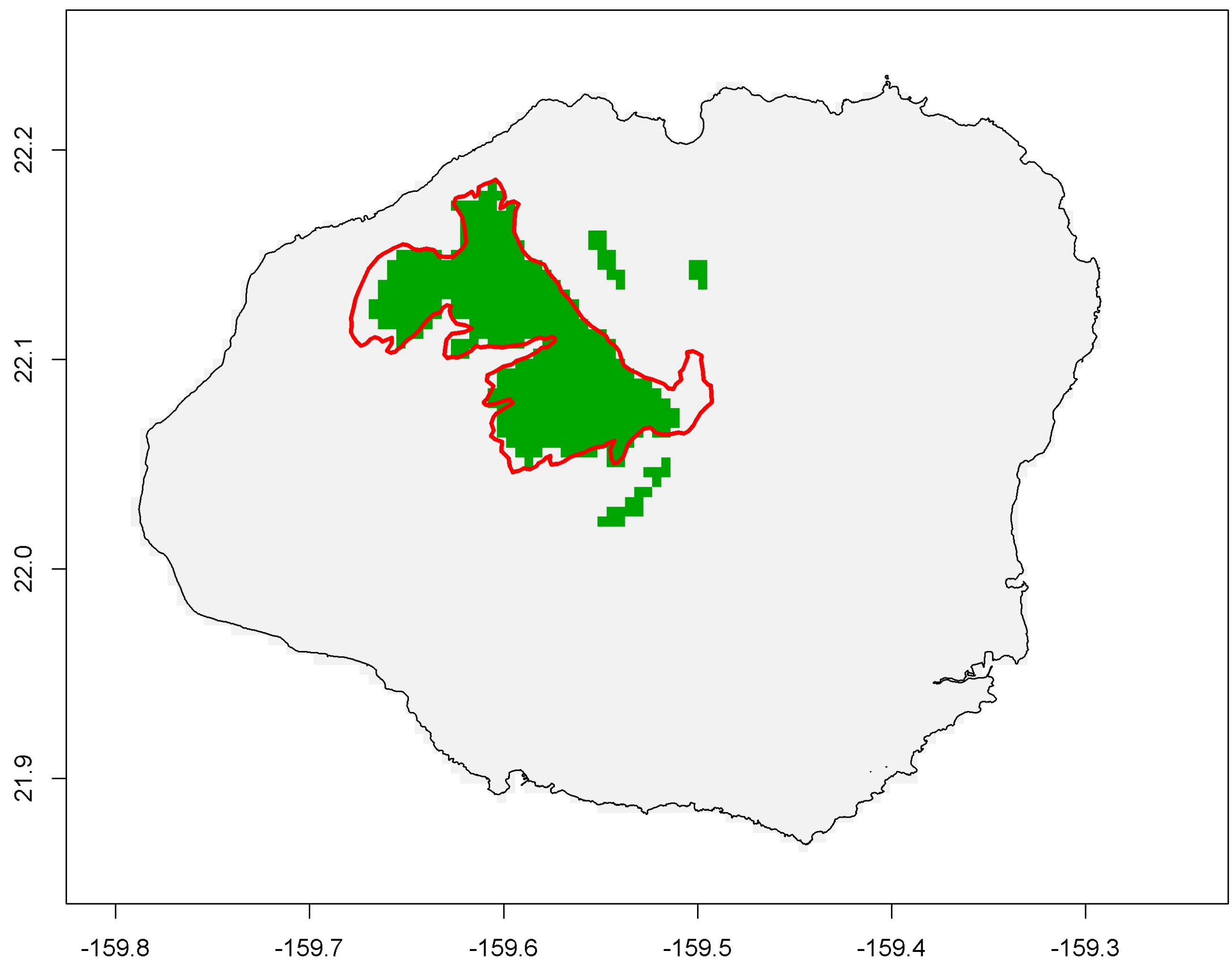

S4: Agreement between expert-derived ranges and current climate-based ranges
REDUCED MODEL RELIABILITY SPECIES

Apapane Expert vs BIN baseline ROC

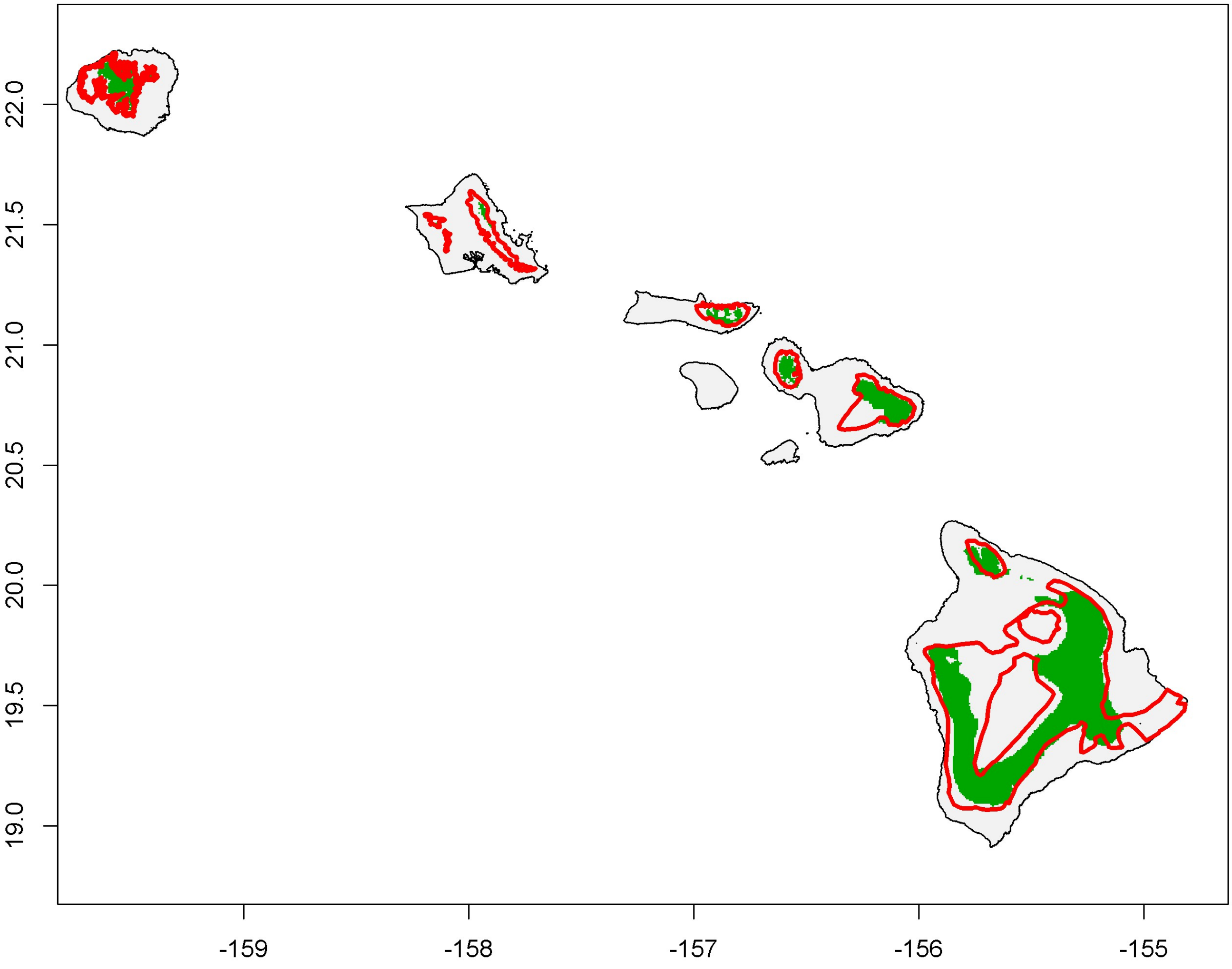

S4: Agreement between expert-derived ranges and current climate-based ranges
REDUCED MODEL RELIABILITY SPECIES

Hawaii Amakihi Expert vs BIN baseline ROC

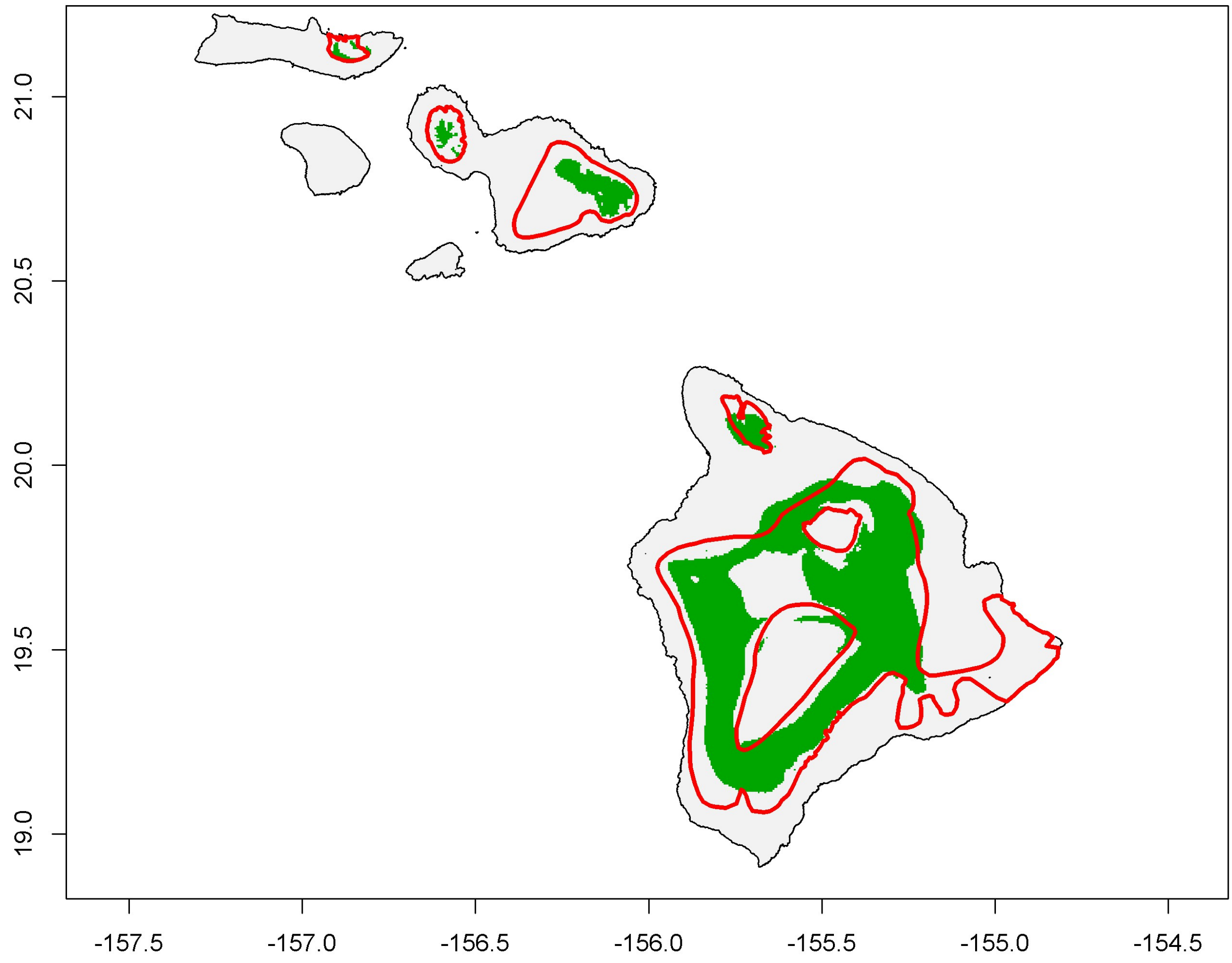

S4: Agreement between expert-derived ranges and current climate-based ranges
REDUCED MODEL RELIABILITY SPECIES

Hawaii Elepaio Expert vs BIN baseline ROC

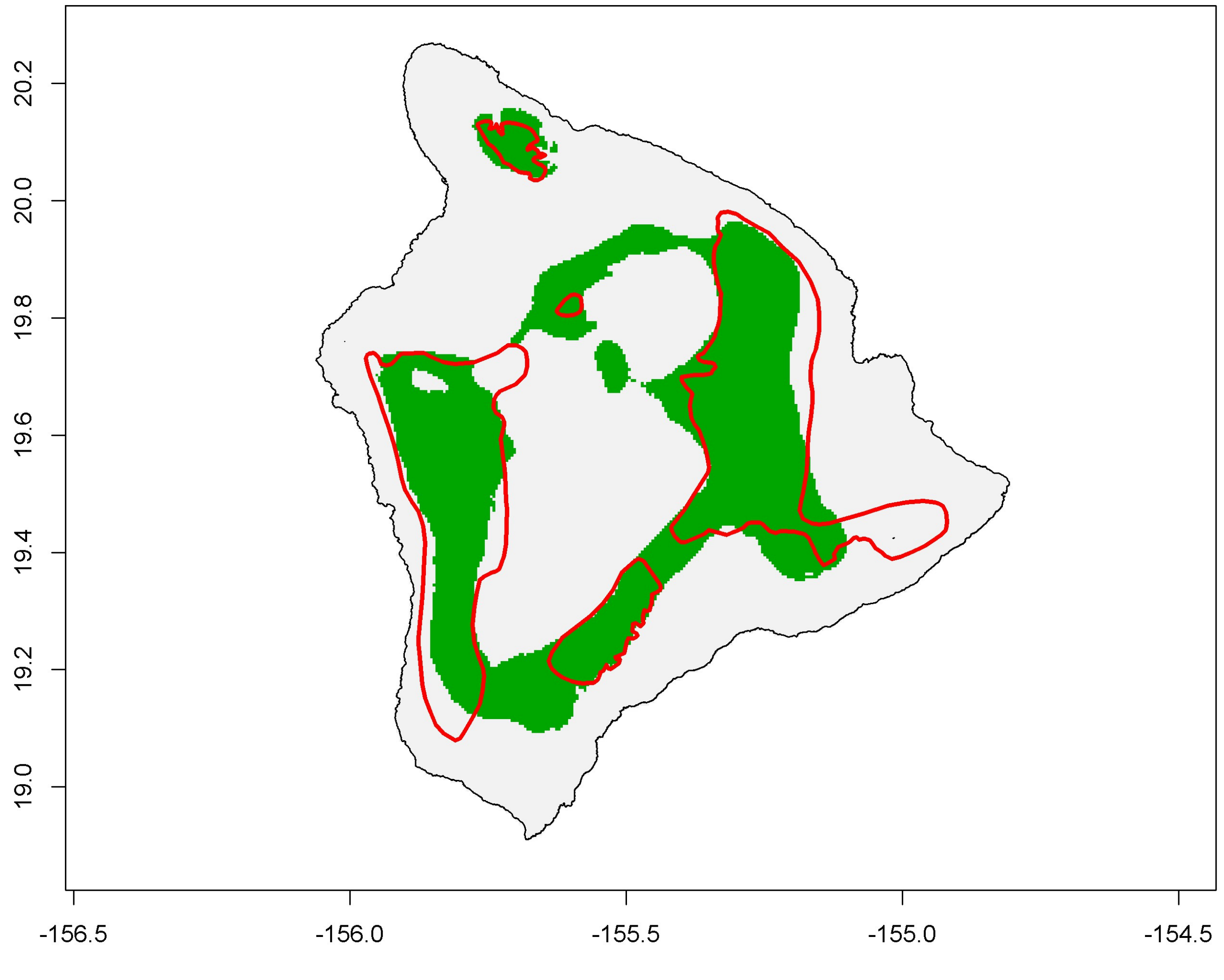

S4: Agreement between expert-derived ranges and current climate-based ranges
REDUCED MODEL RELIABILITY SPECIES

Kauai Amakihi Expert vs BIN baseline ROC

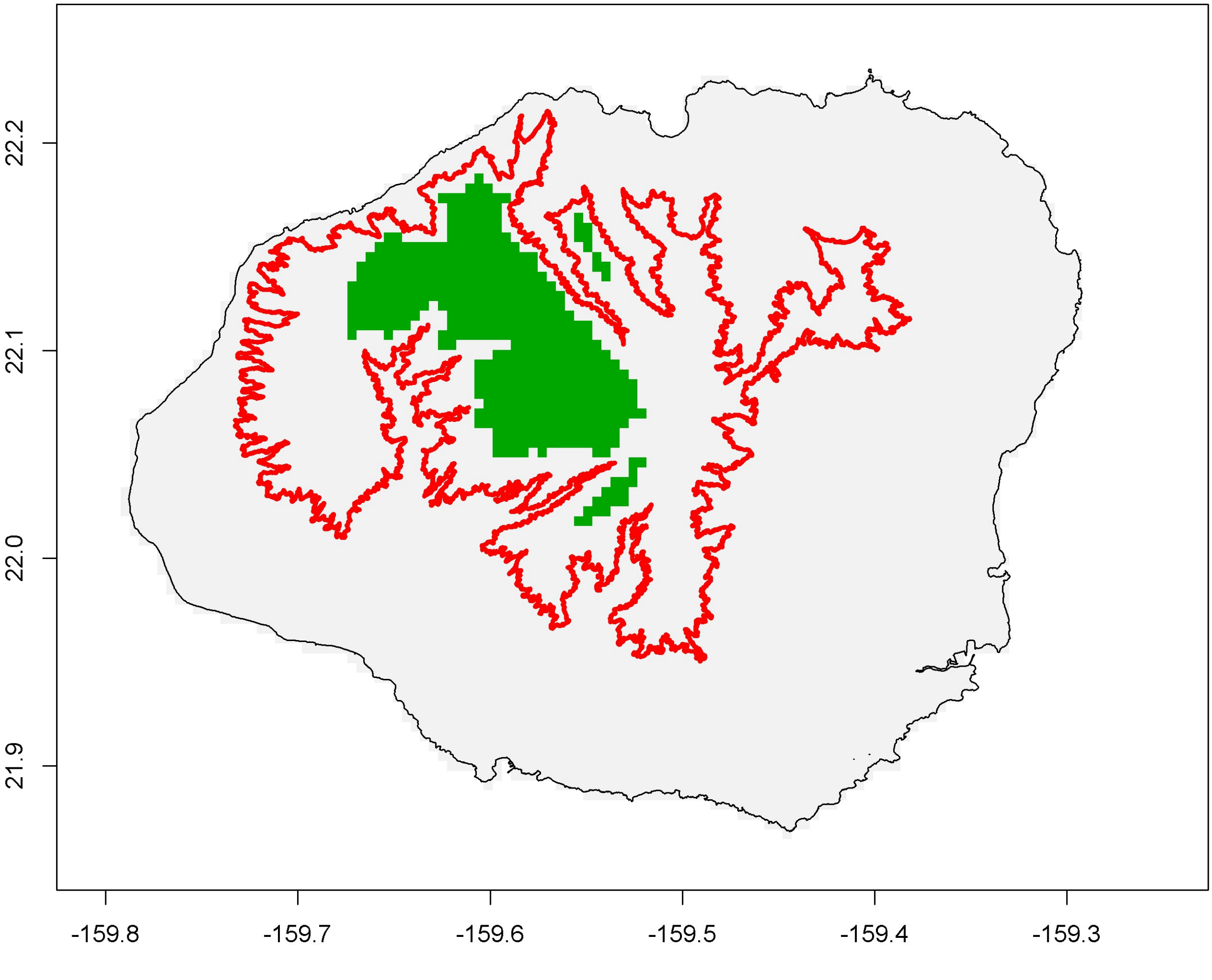

S4: Agreement between expert-derived ranges and current climate-based ranges
REDUCED MODEL RELIABILITY SPECIES

Kauai Elepaio Expert vs BIN baseline ROC

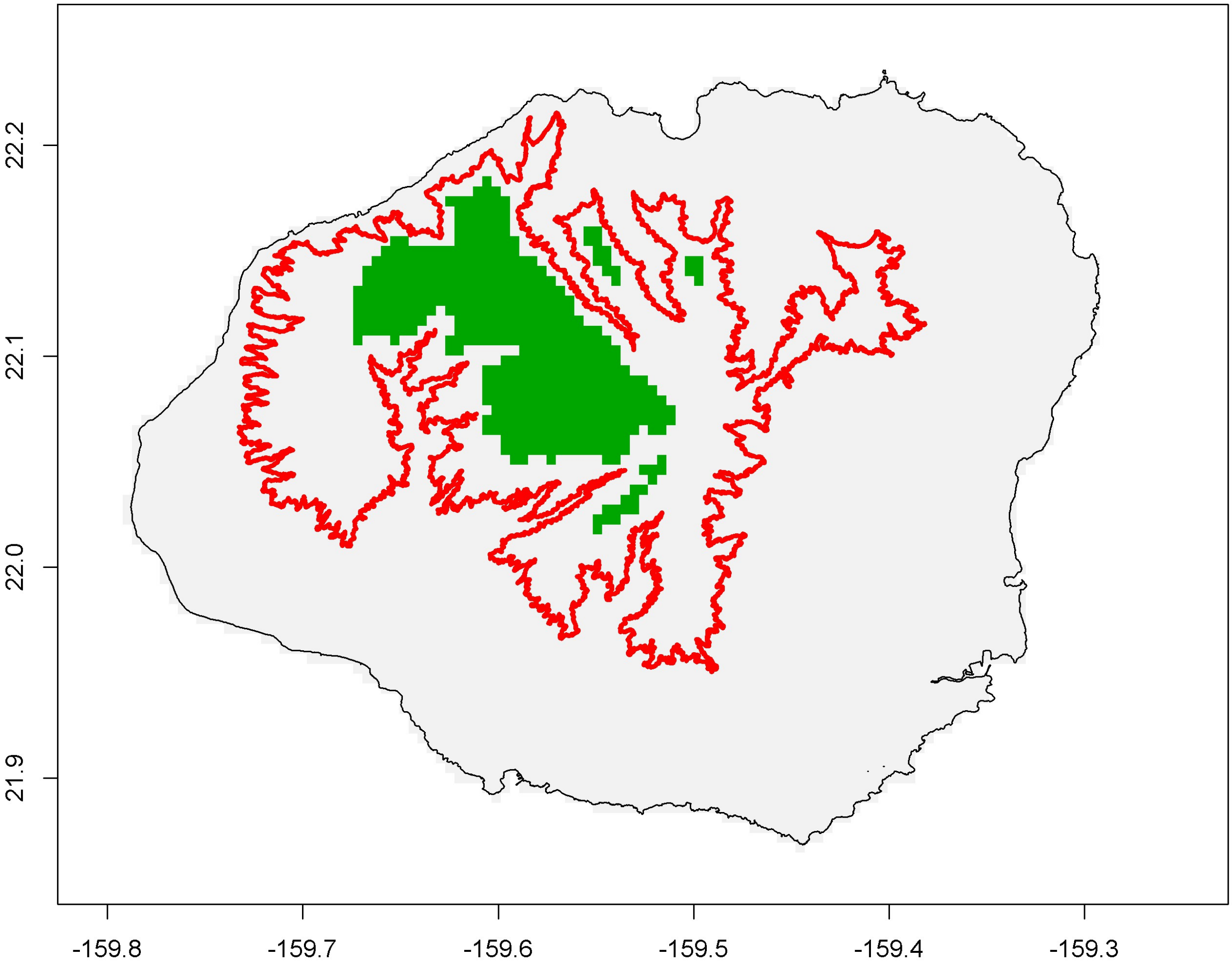

S4: Agreement between expert-derived ranges and current climate-based ranges
REDUCED MODEL RELIABILITY SPECIES

Oahu Amakihi Expert vs BIN baseline ROC

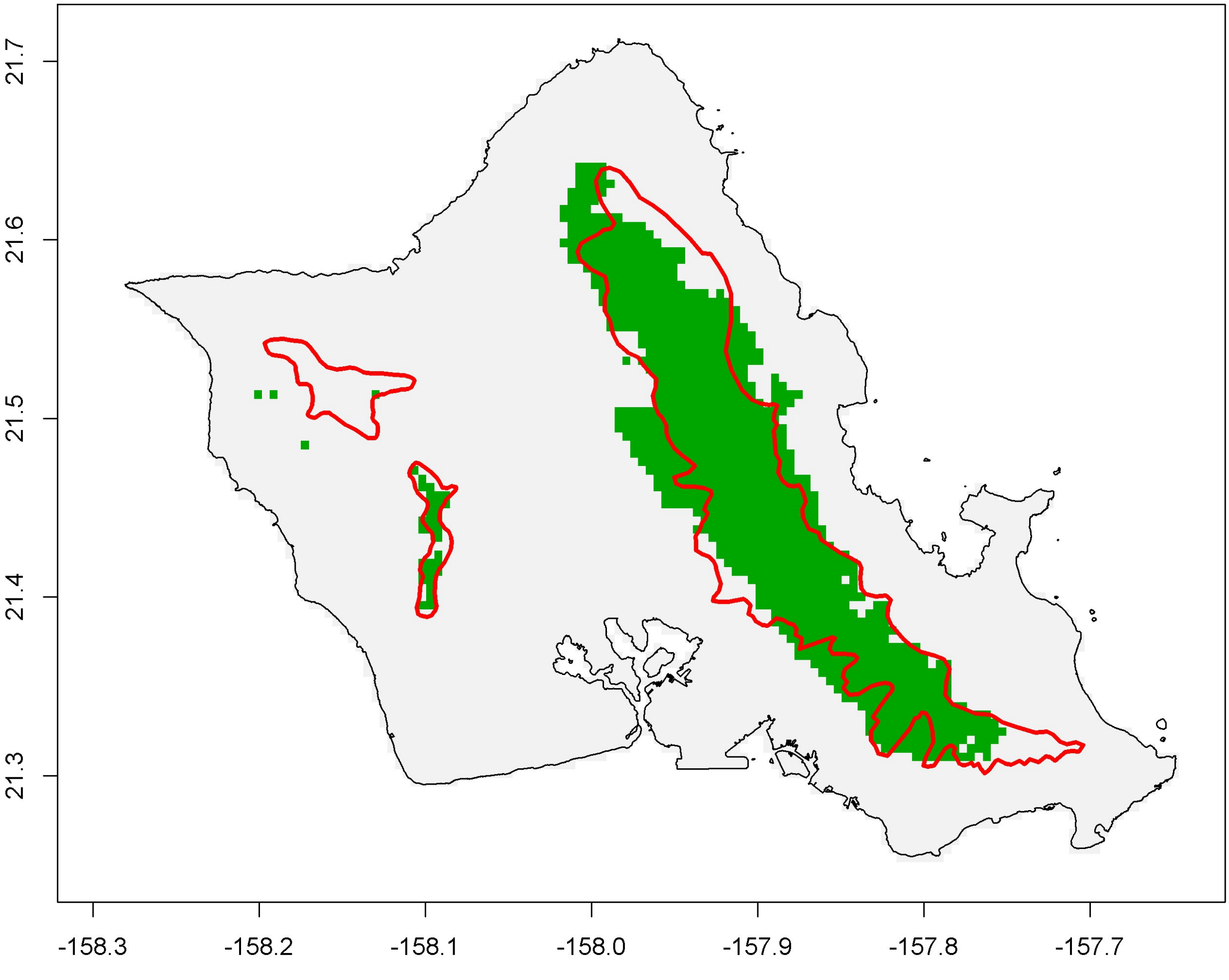

S4: Agreement between expert-derived ranges and current climate-based ranges
REDUCED MODEL RELIABILITY SPECIES

Oahu Elepaio Expert vs BIN baseline ROC

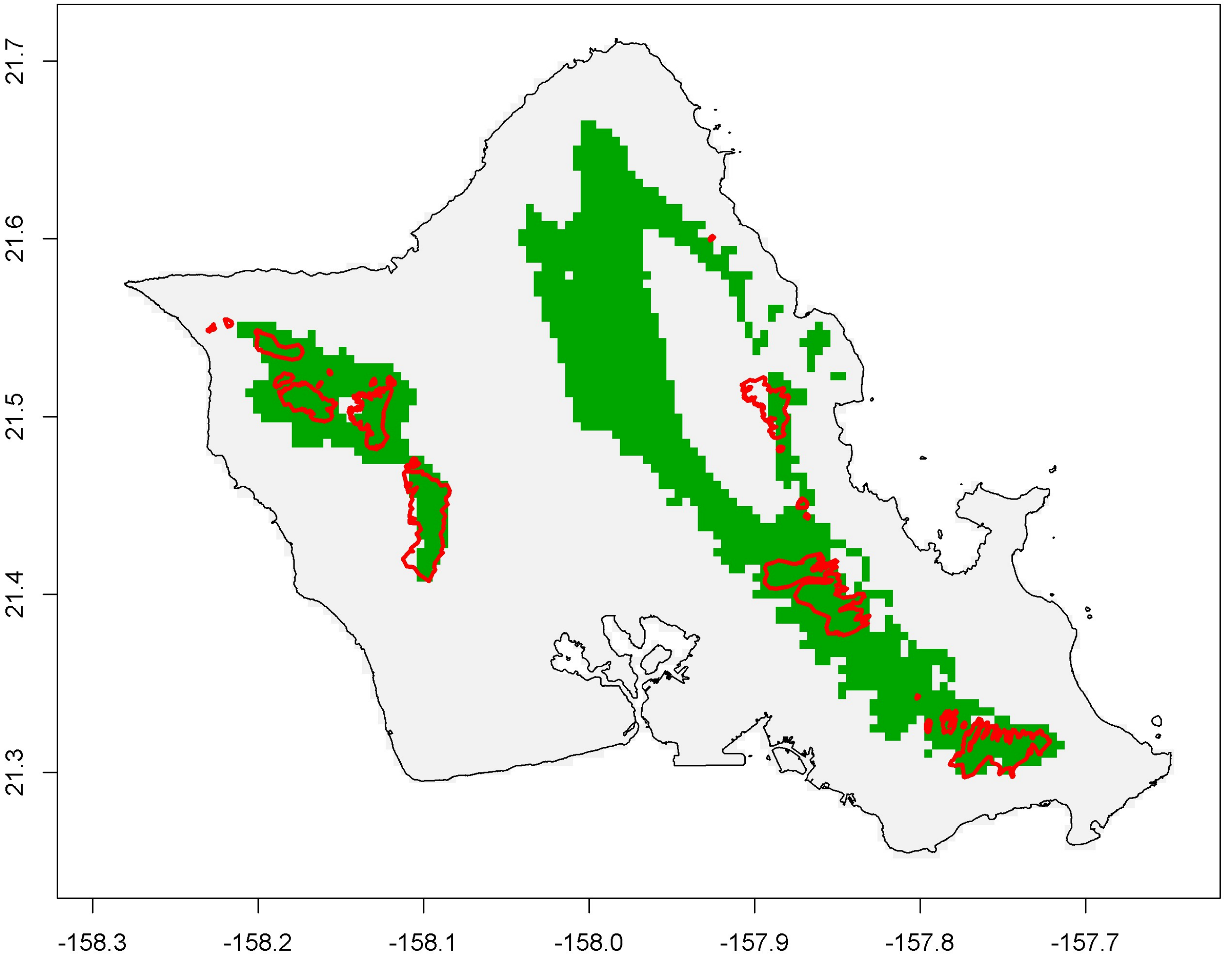

S4: Agreement between expert-derived ranges and current climate-based ranges
REDUCED MODEL RELIABILITY SPECIES

Omao Expert vs BIN baseline ROC

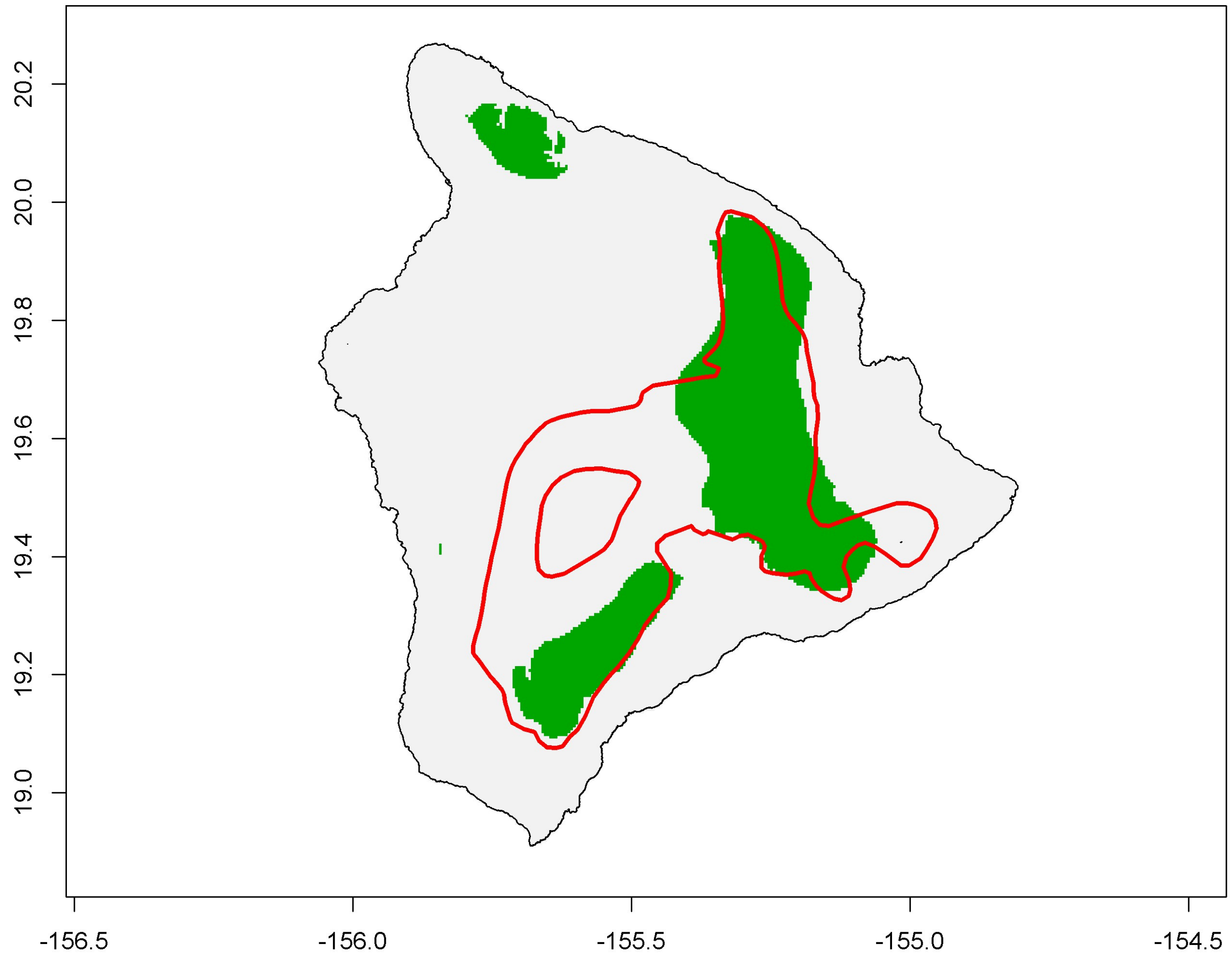

S4: Agreement between expert-derived ranges and current climate-based ranges
REDUCED MODEL RELIABILITY SPECIES

Palila Expert vs BIN baseline ROC

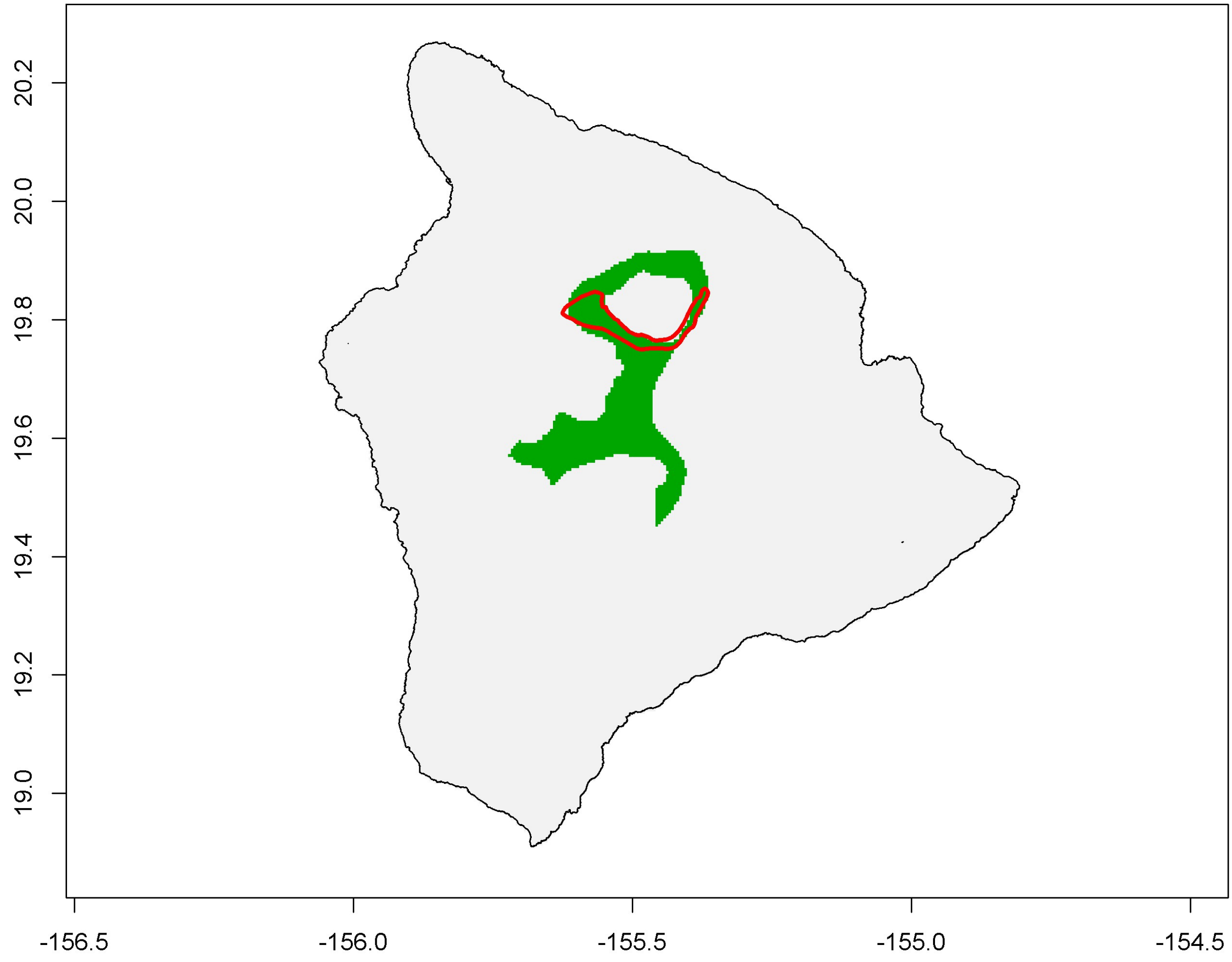

Supplement: S4 File — (PDF) [file pone.0140389.s004.pdf]
