# Supplementary material for: Large-Scale Range Collapse of Hawaiian Forest Birds under Climate Change and the Need 21st Century Conservation Options
Source: PLoS One. 2015 Oct 28;10(10):e0140389. doi: 10.1371/journal.pone.0140389 (PMC4625087; doi:10.1371/journal.pone.0140389)

Appendix S5. Table of forest bird primary habitat associations, figures of proportional presence of species along climate gradients, and map of 2100 analogous climate areas.

Analogue climate map shows areas (in green) where 2100 climate is projected to remain within the 99.9% quantile range of current bioclimatic values (for bio 1, 7, 12, 15). This is based on a simple rectilinear climate envelope approach (refs below). The map shows the majority of non-analogue areas are in coastal areas that by 2100 are projected to be hotter than anywhere currently in the archipelago. Native forest birds are largely absent from these low elevation areas today.

Busby, J.R. 1991. BIOCLIM - a bioclimate analysis and prediction system. Plant Protection Quarterly 6(1): 8–9.

Nix, H.A. 1986. A biogeographic analysis of Australian Elapid snakes. p. 4–15. In Longmore, R. (ed.), Atlas of Australian Elapid snakes. Bureau of Flora and Fauna, Canberra.

Price, J.P., J.D. Jacobi, S.M. Gon, D. Matsuwaki, L. Mehrhoff, W. Wagner, M. Lucas, and B. Rowe. 2012. Mapping plant species ranges in the Hawaiian Islands-Developing a methodology and associated GIS layers. United States Geological Survey.

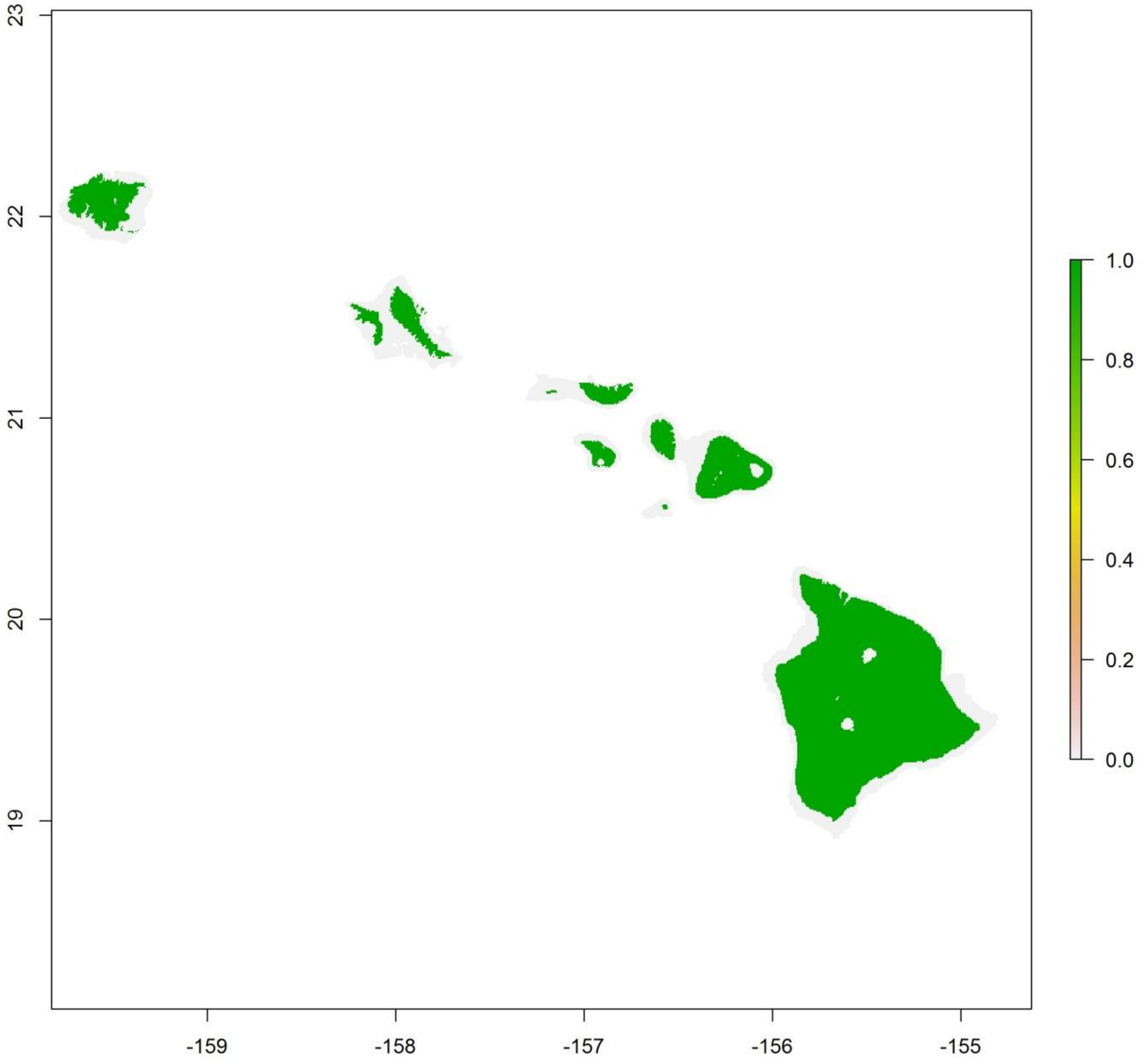

Table listing the primary habitat considered for each species, as described in manuscript. Percent distribution of known species locations across broad land cover classes. Values in bold denote cover classes that make up to 90% of known species sightings. Since we exclude 'other' cover, highlighted values represent cover types considered the primary habitat for the species.

**Land cover**	**Wet forest**	**Mesic forest**	**Dry shrubland**	**Other**	**Dry forest**	**Perennial grassland**	**Wet/mesic forest**	**Evergreen shrubland**	**Dry grassland**	**Mesic shrubland**	**Deciduous shrubland**	**Mesic grassland**
**Akekee**	**93**	4	0	1	0	0	2	0	0	0	0	0
**Anianiau**	**91**	3	0	2	0	0	4	0	0	0	0	0
**Akikiki**	**100**	0	0	0	0	0	0	0	0	0	0	0
**Puaiohi**	**100**	0	0	0	0	0	0	0	0	0	0	0
**Hawaii_Akepa**	**61**	**33**	4	1	0	0	**0**	0	0	0	0	1
**Hawaii_Creeper**	**65**	**31**	2	1	0	0	**0**	0	0	0	0	1
**Kauai_Amakihi**	**79**	**9**	0	3	0	0	**9**	0	0	0	0	0
**Kauai_Elepaio**	**82**	**8**	0	3	0	0	**8**	0	0	0	0	0
**Omao**	**67**	**23**	4	4	0	1	**1**	0	0	0	0	0
**Maui_Parrotbill**	**63**	**23**	0	**14**	0	0	**0**	0	0	0	0	0
**Oahu_Amakihi**	**48**	**6**	0	**13**	1	0	**28**	4	0	0	1	0
**Akohekohe**	**57**	**22**	0	**20**	0	0	**0**	0	0	0	0	0
**Maui_Alauahio**	**71**	**13**	0	**14**	1	1	**1**	0	0	0	0	0
**Akiapolauu**	**52**	**32**	**7**	1	3	4	**0**	0	0	0	0	0
**Apapane**	**53**	**23**	**8**	5	3	3	**2**	0	0	1	0	0
**Iiwi**	**60**	**26**	**6**	3	2	2	**1**	0	0	0	0	0
**Hawaii_Elepaio**	**51**	**29**	**5**	3	**7**	3	**1**	0	0	0	0	0
**Hawaii_Amakihi**	**35**	**28**	**14**	**6**	**9**	5	**1**	0	0	0	0	0
**Oahu_Elepaio**	**16**	**17**	0	3	1	1	**55**	**7**	0	0	1	0
**Palila**	0	0	**45**	8	**34**	**12**	0	0	0	0	0	0
**All combined**	54	25	8	4	4	3	2	0	0	0	0	0

All proportional presence figures below follow the exact format as figure 1 in the manuscript.

File S5. Proportional presence of species along climate gradients

To determine if location data encompassed the full range of each species modeled, using presence/absence data from all locations for each species, we compared overall species prevalence (proportion of total points a species was sought in which species was actually observed) with prevalence at the low and high ends of our climate predictors (using <2.5% and >97.5% quantile values). In this simplified analyses we classified species as being comprehensively sampled if they had prevalence at the limits of surveyed climate < 50% of the overall species prevalence, with prevalence weighted by climate predictor importance. This was done as we naturally expected the prevalence at the limits of surveyed climate predictors to be substantially smaller than overall prevalence.

As a comparative example, the figure below shows proportional presence of species representing adequate (a- Akikiki) and poor (b- Kaua'i `Amakihi) sampling along temperature gradients. In the case of Kauai `Amakihi, the high proportion of presence locations with respect to total bird survey locations at higher temperatures indicate the species can likely inhabit locations hotter than those represented across all available bird surveys. Similar figures for all other species and predictors are included below.

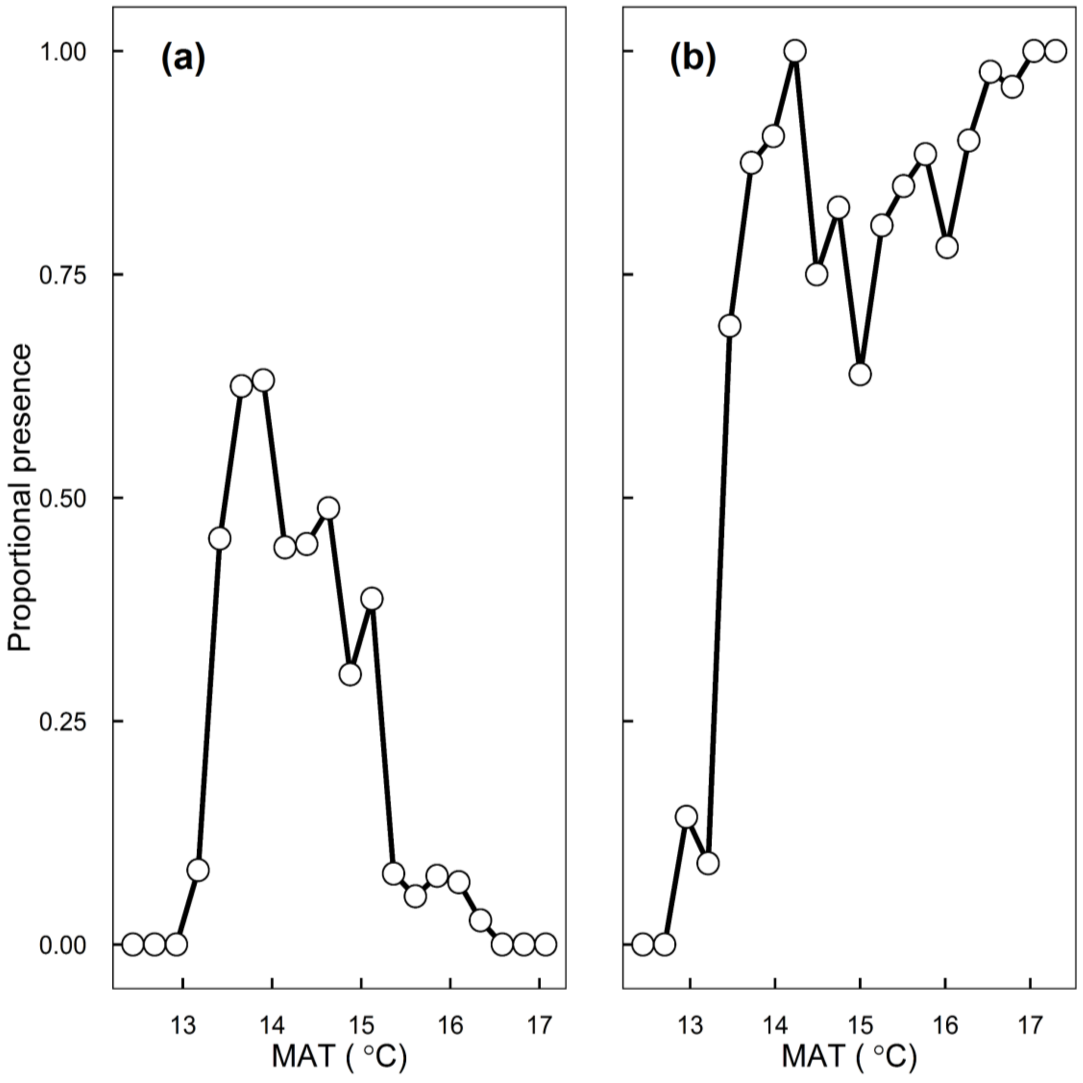

S5
Proportional presence of species along climate gradients

Proportional presence Akekee bio1

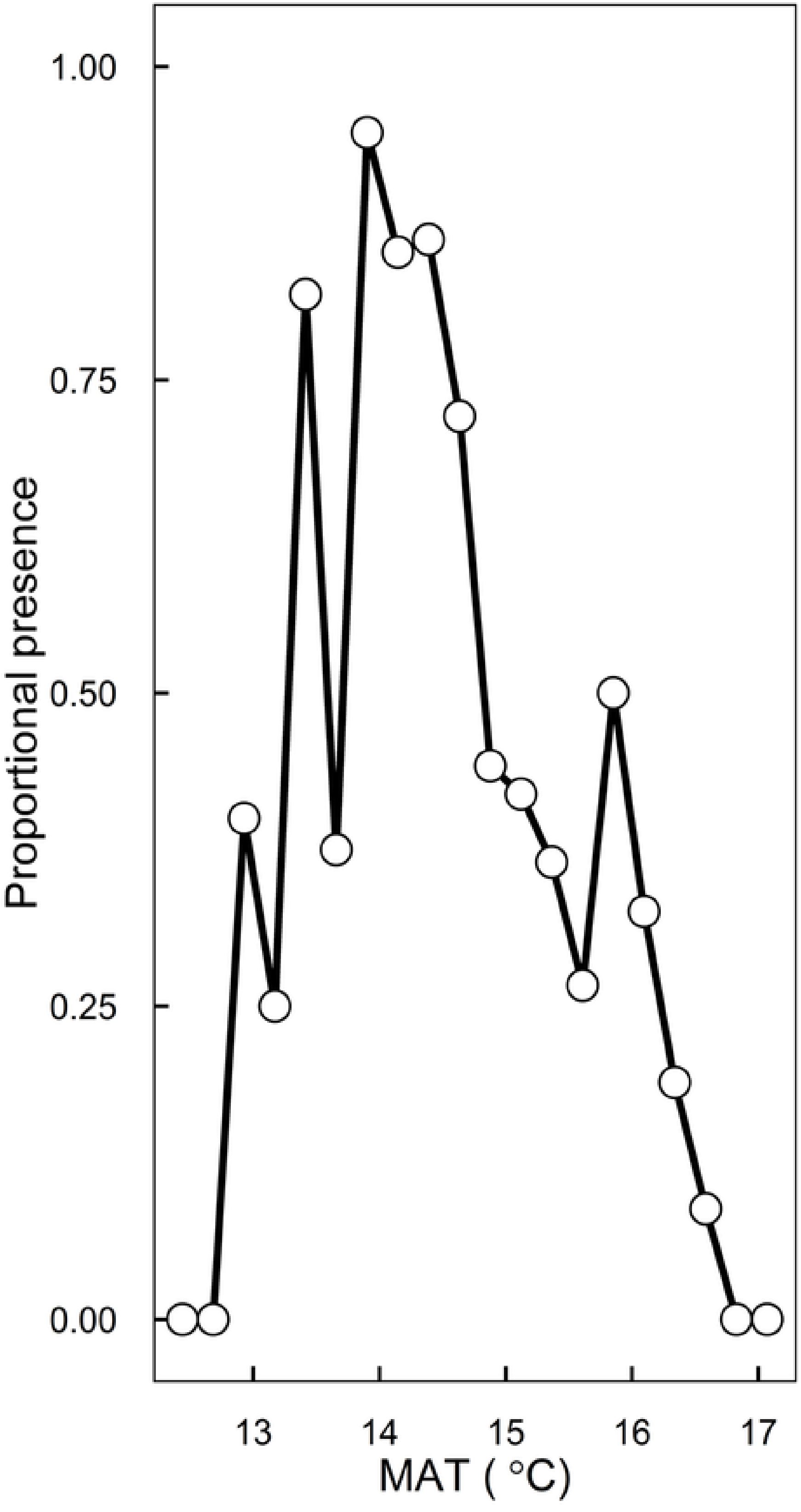

S5
Proportional presence of species along climate gradients

Proportional presence Akekee bio12

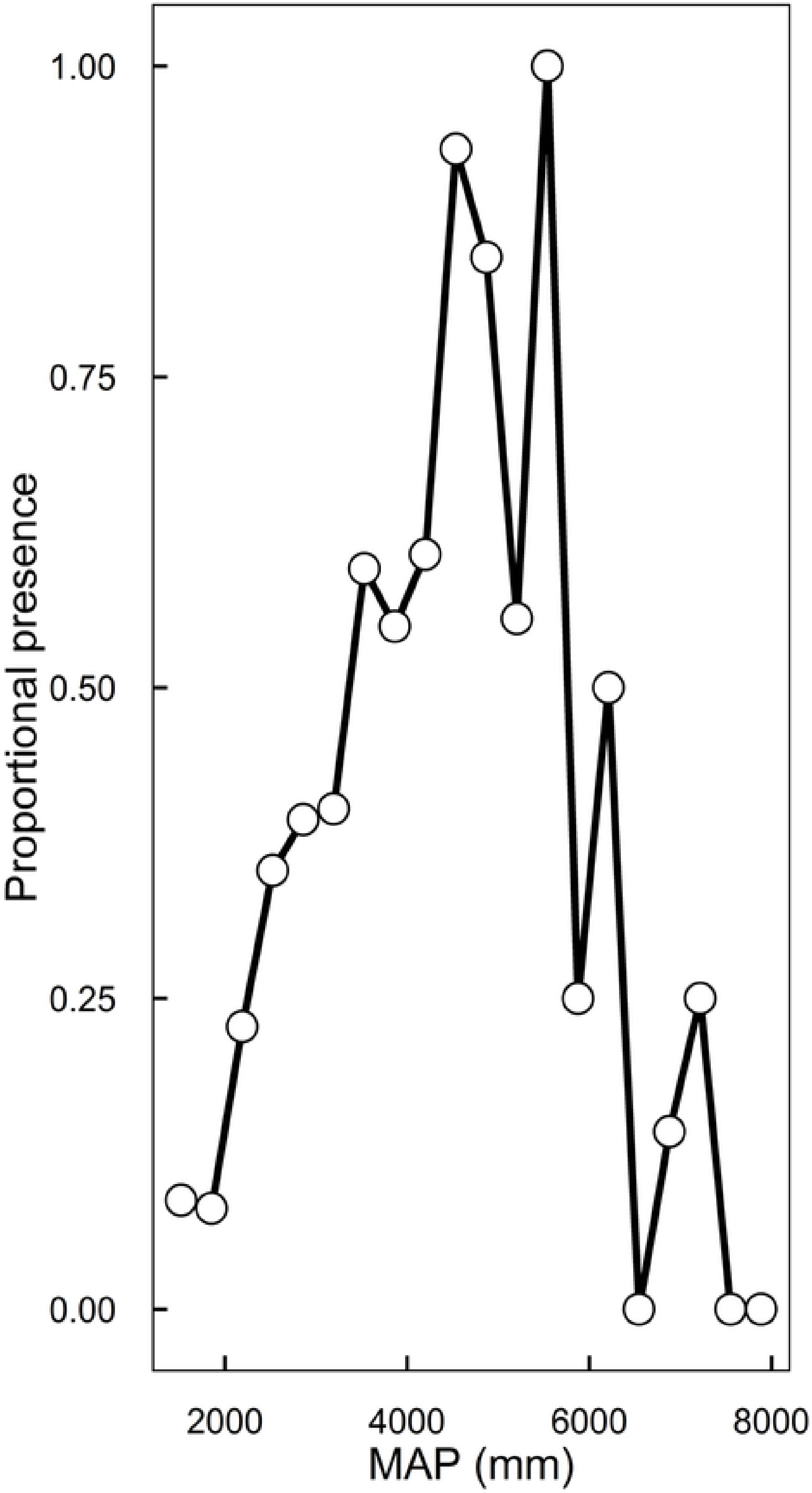

S5
Proportional presence of species along climate gradients

Proportional presence Akekee bio15

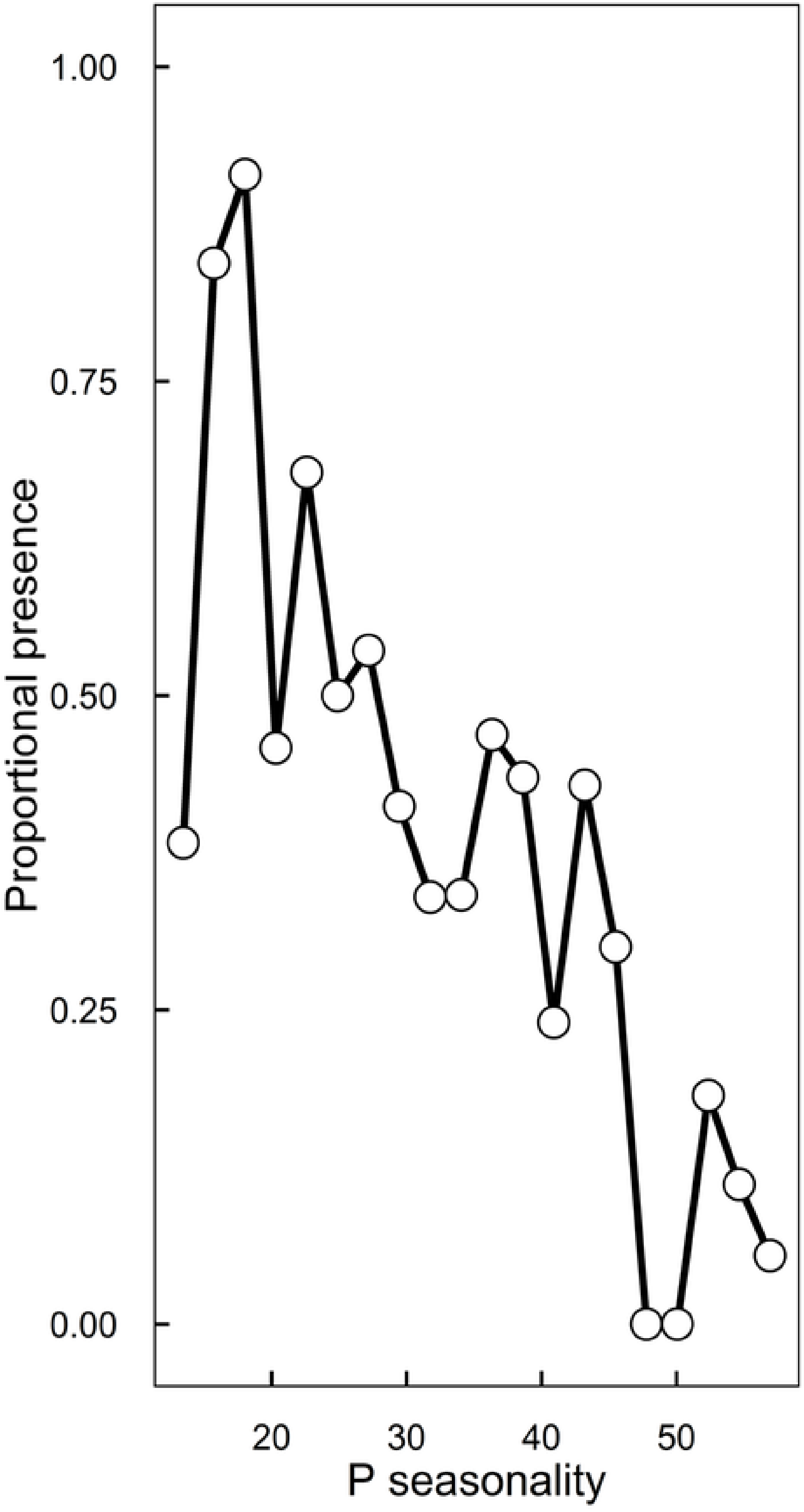

S5
Proportional presence of species along climate gradients

Proportional presence Akekee bio7

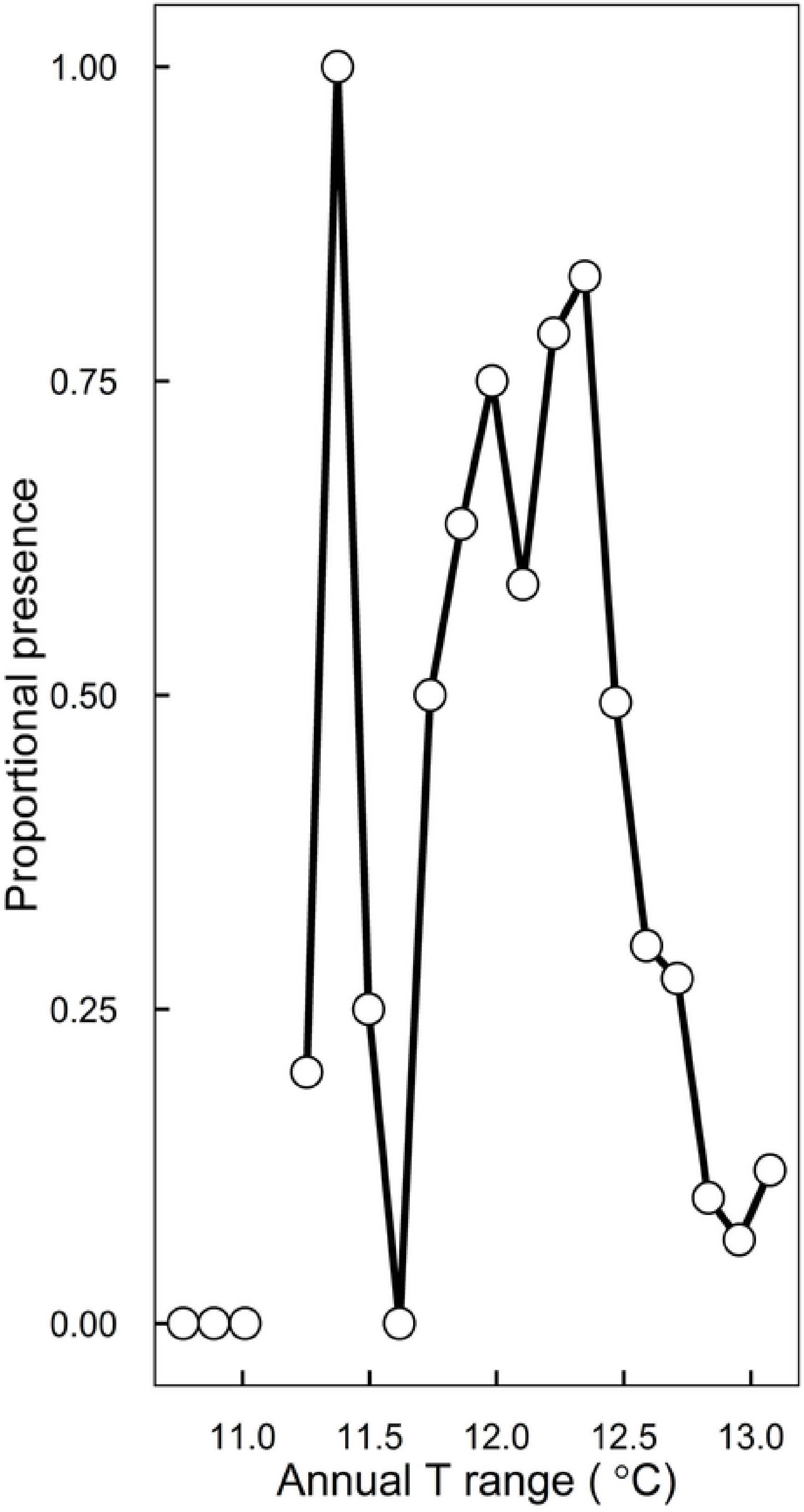

S5
Proportional presence of species along climate gradients

Proportional presence Akiapolauu bio1

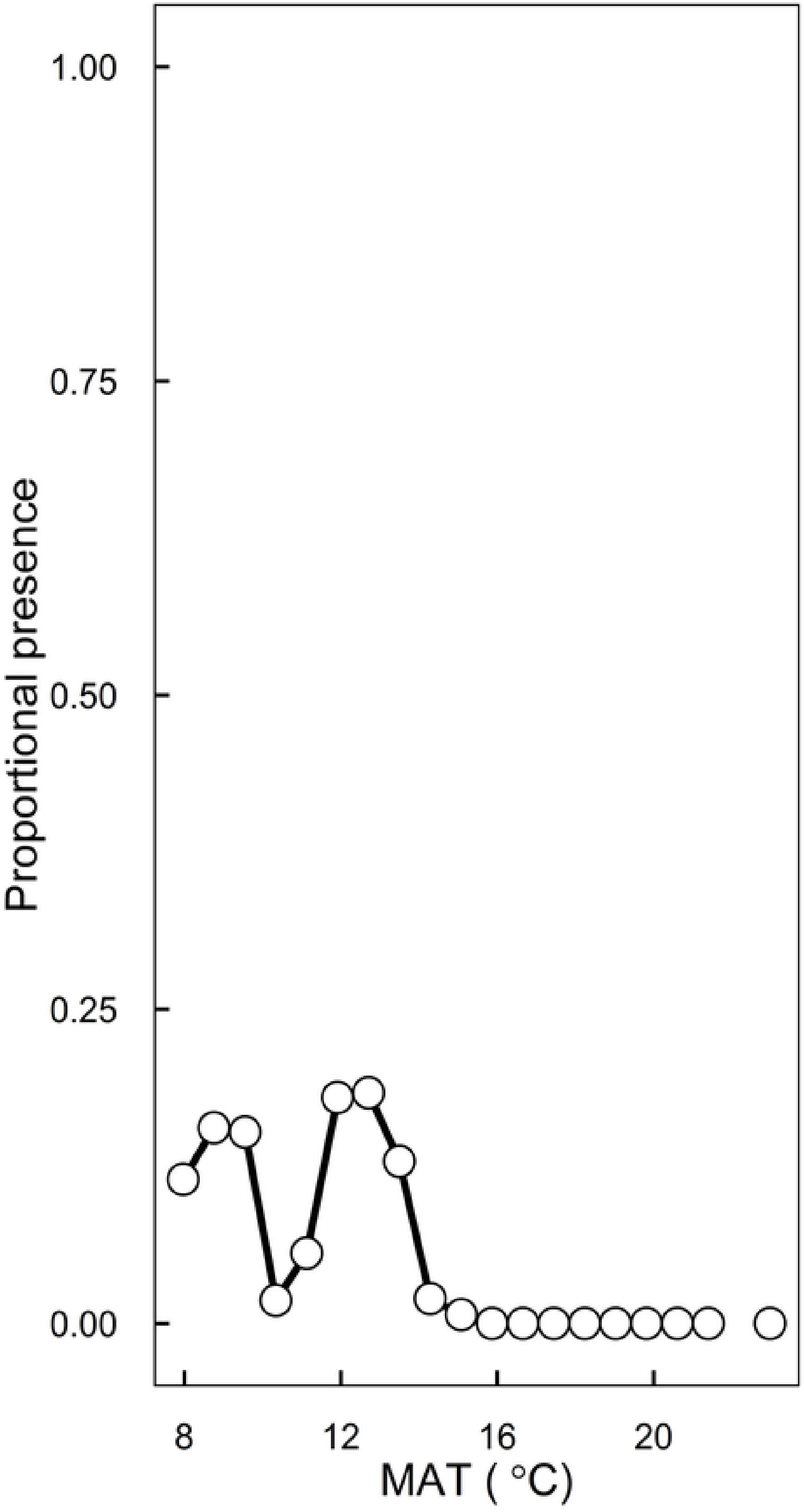

S5
Proportional presence of species along climate gradients

Proportional presence Akiapolauu bio12

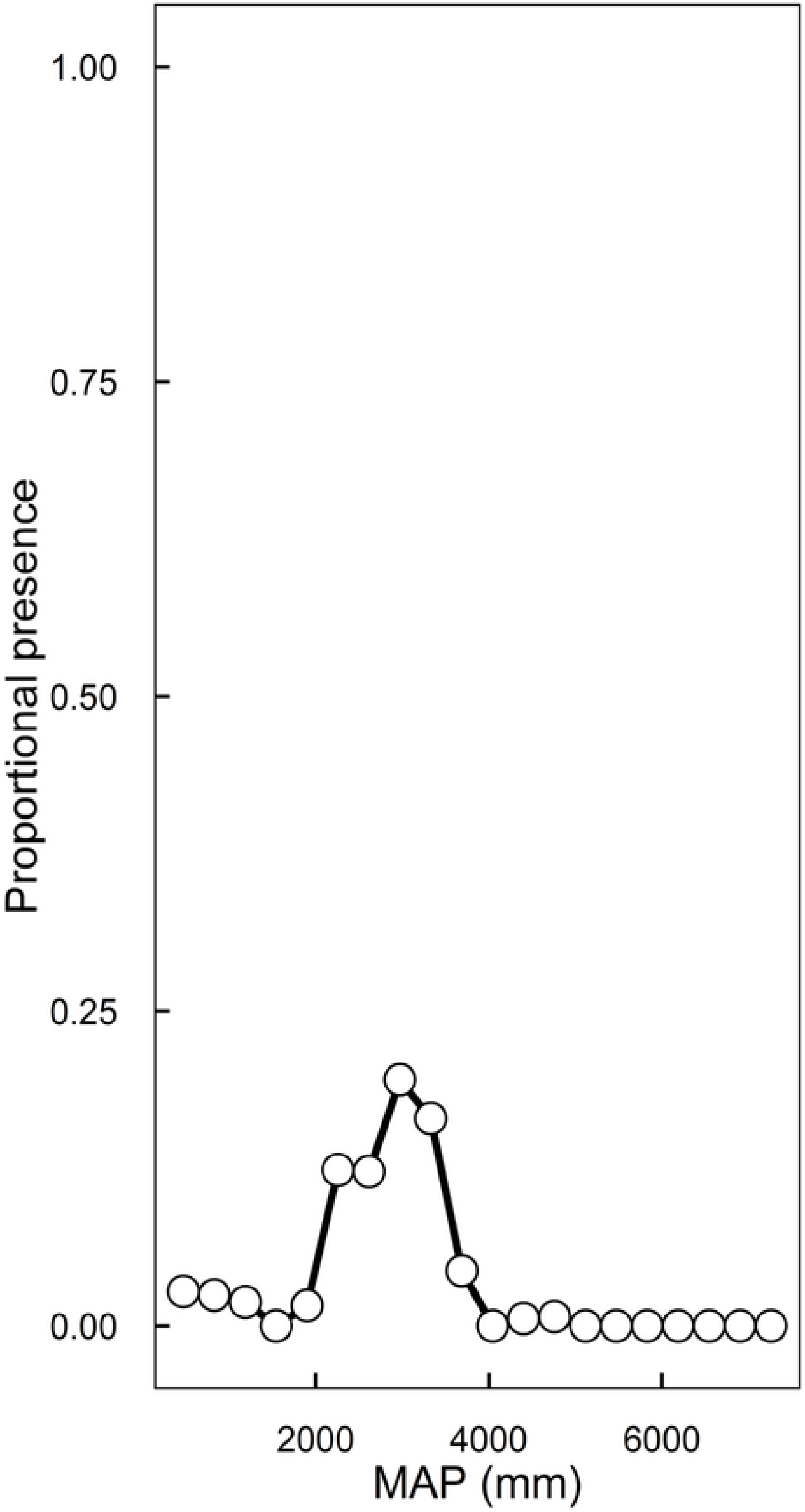

S5
Proportional presence of species along climate gradients

Proportional presence Akiapolauu bio15

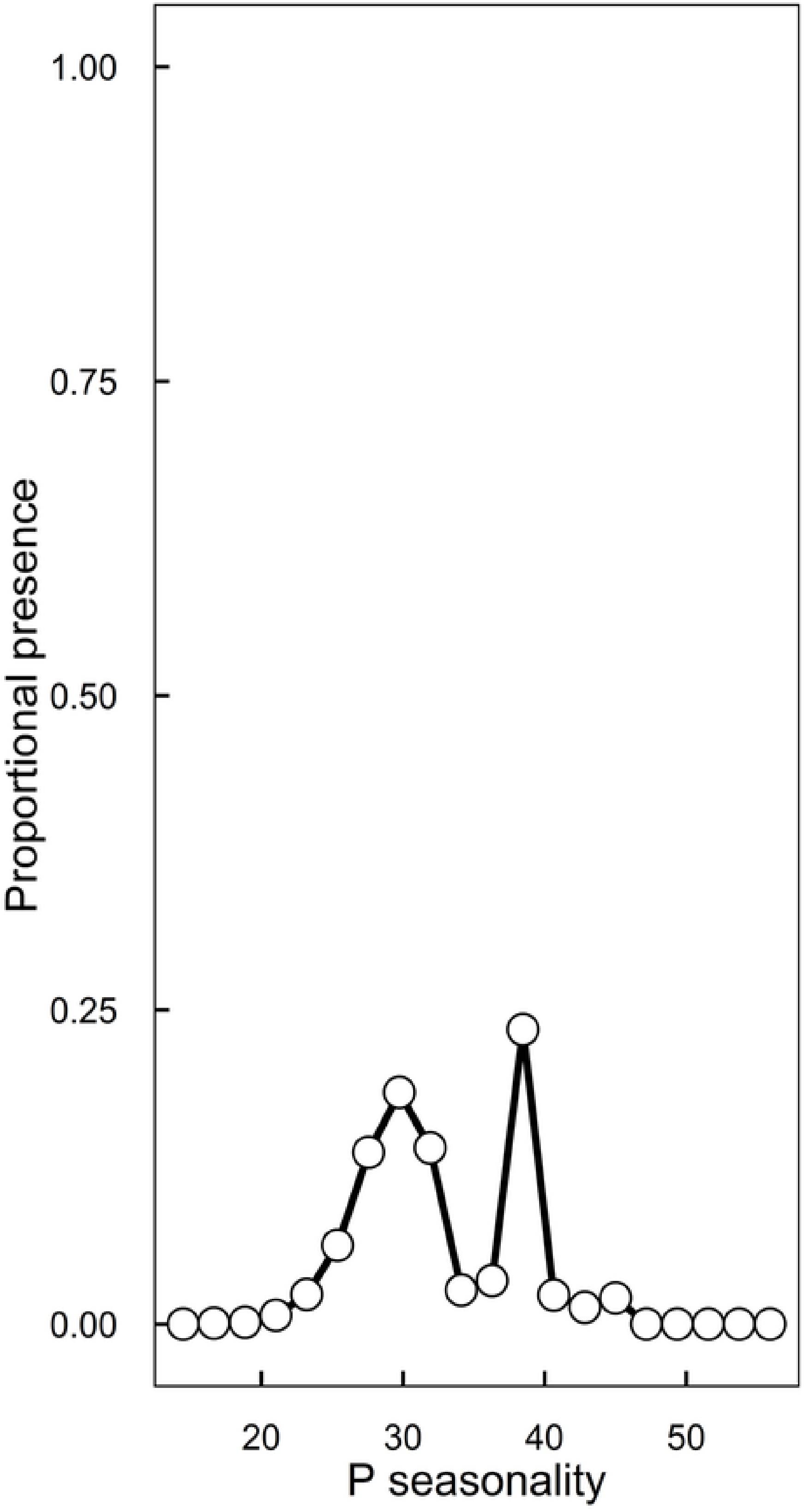

S5
Proportional presence of species along climate gradients

Proportional presence Akiapolauu bio7

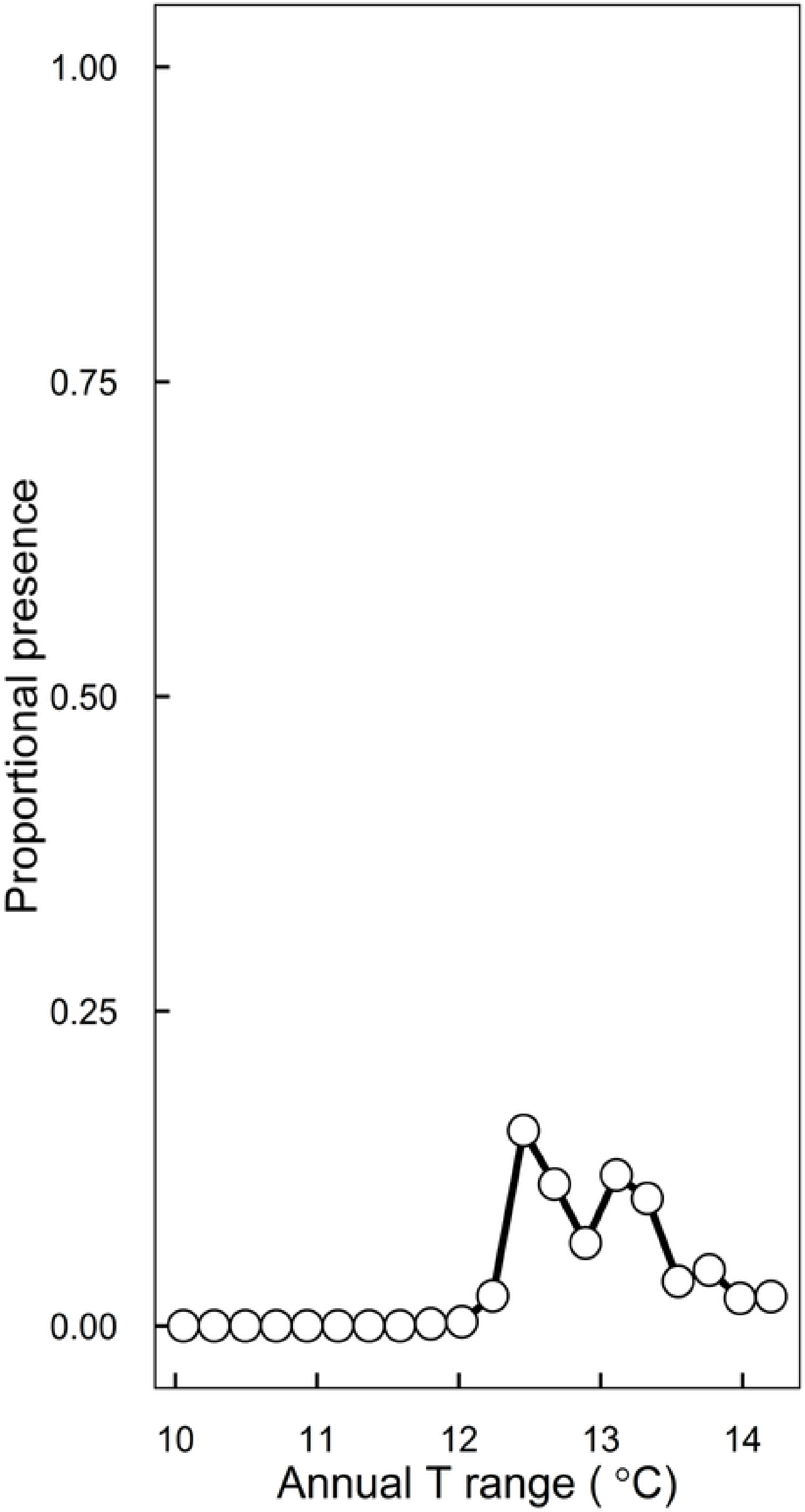

S5
Proportional presence of species along climate gradients

Proportional presence Akikiki bio1

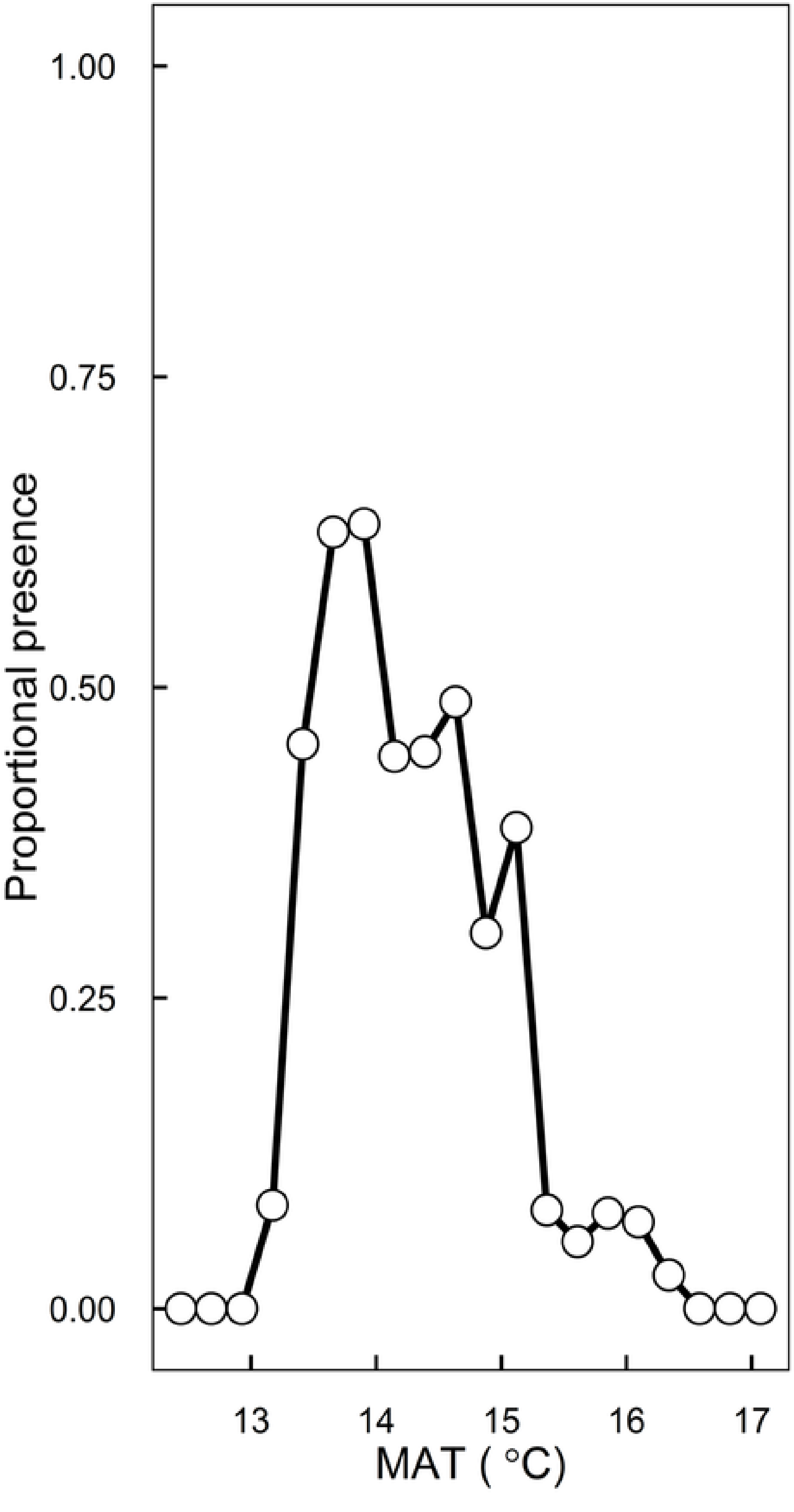

S5
Proportional presence of species along climate gradients

Proportional presence Akikiki bio12

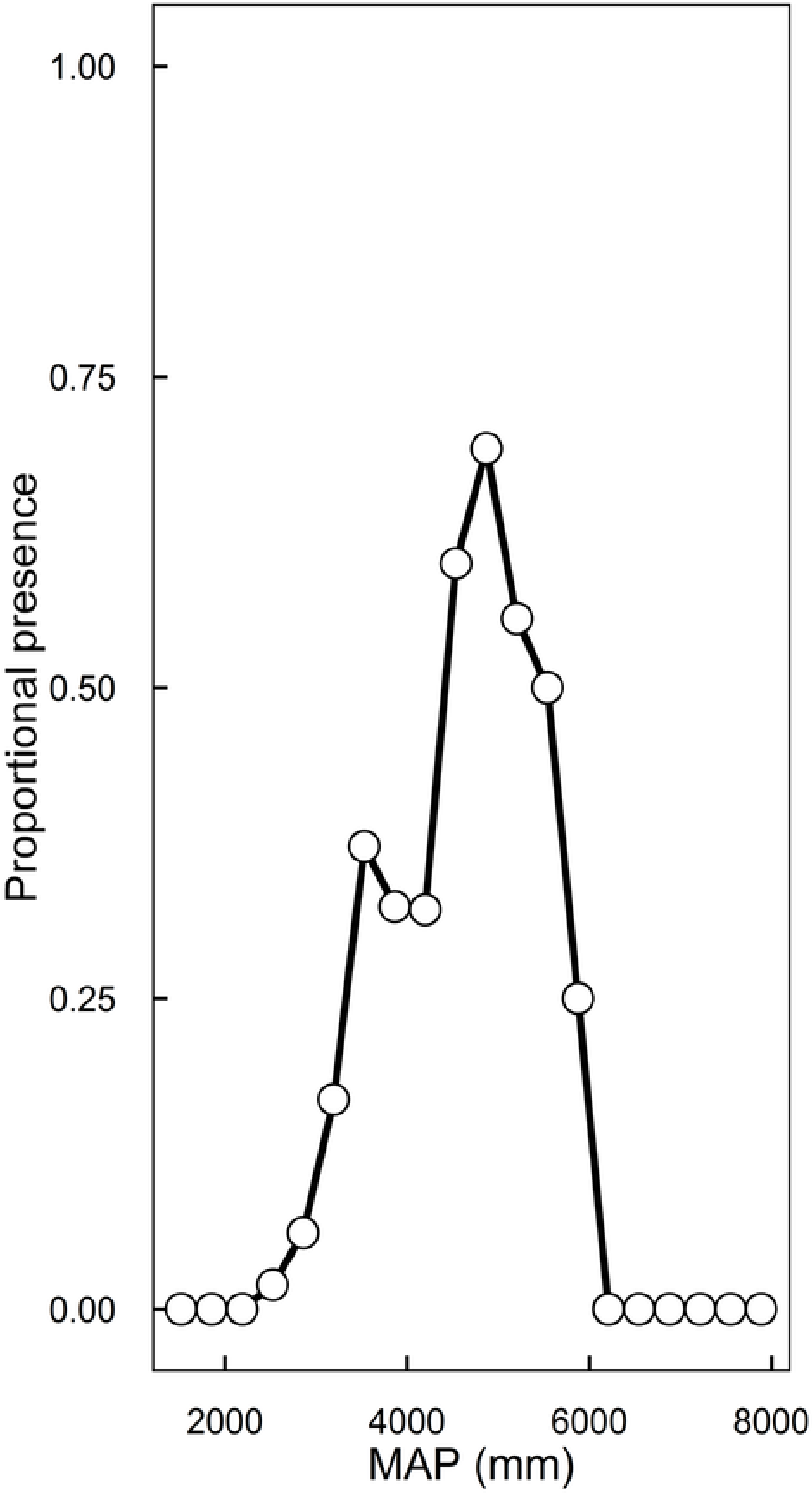

S5
Proportional presence of species along climate gradients

Proportional presence Akikiki bio15

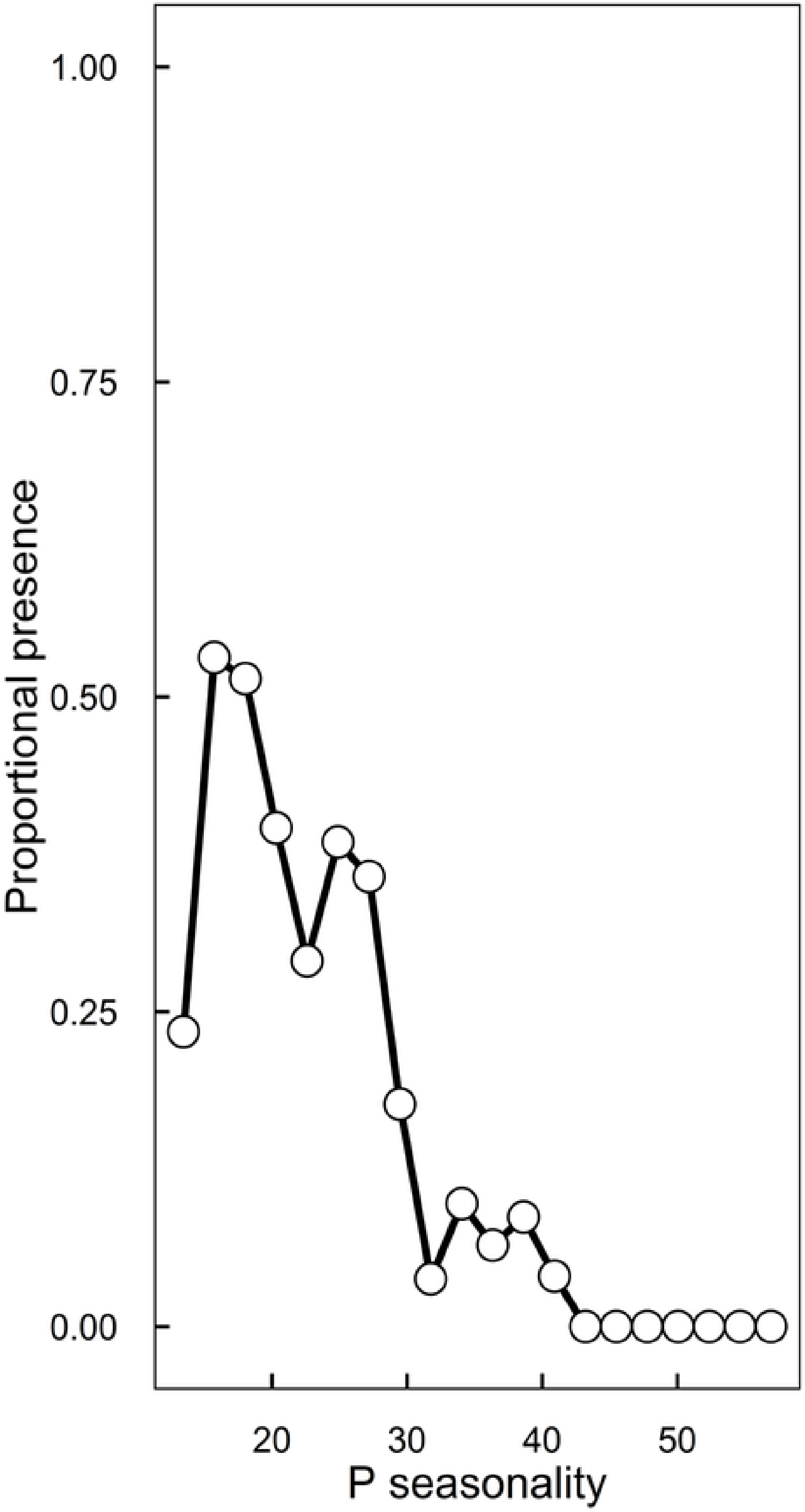

S5
Proportional presence of species along climate gradients

Proportional presence Akikiki bio7

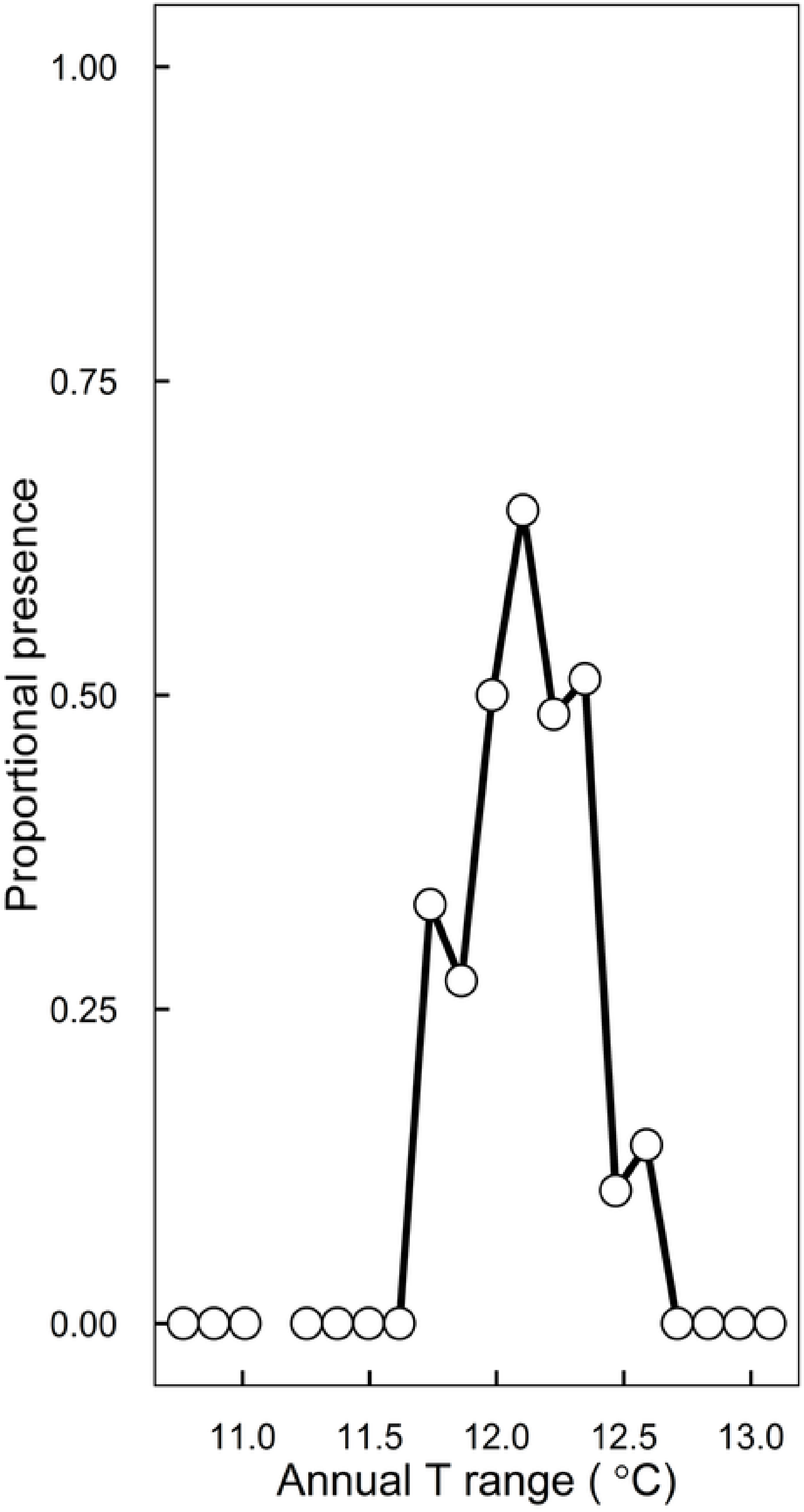

S5
Proportional presence of species along climate gradients

Proportional presence Akohekohe bio1

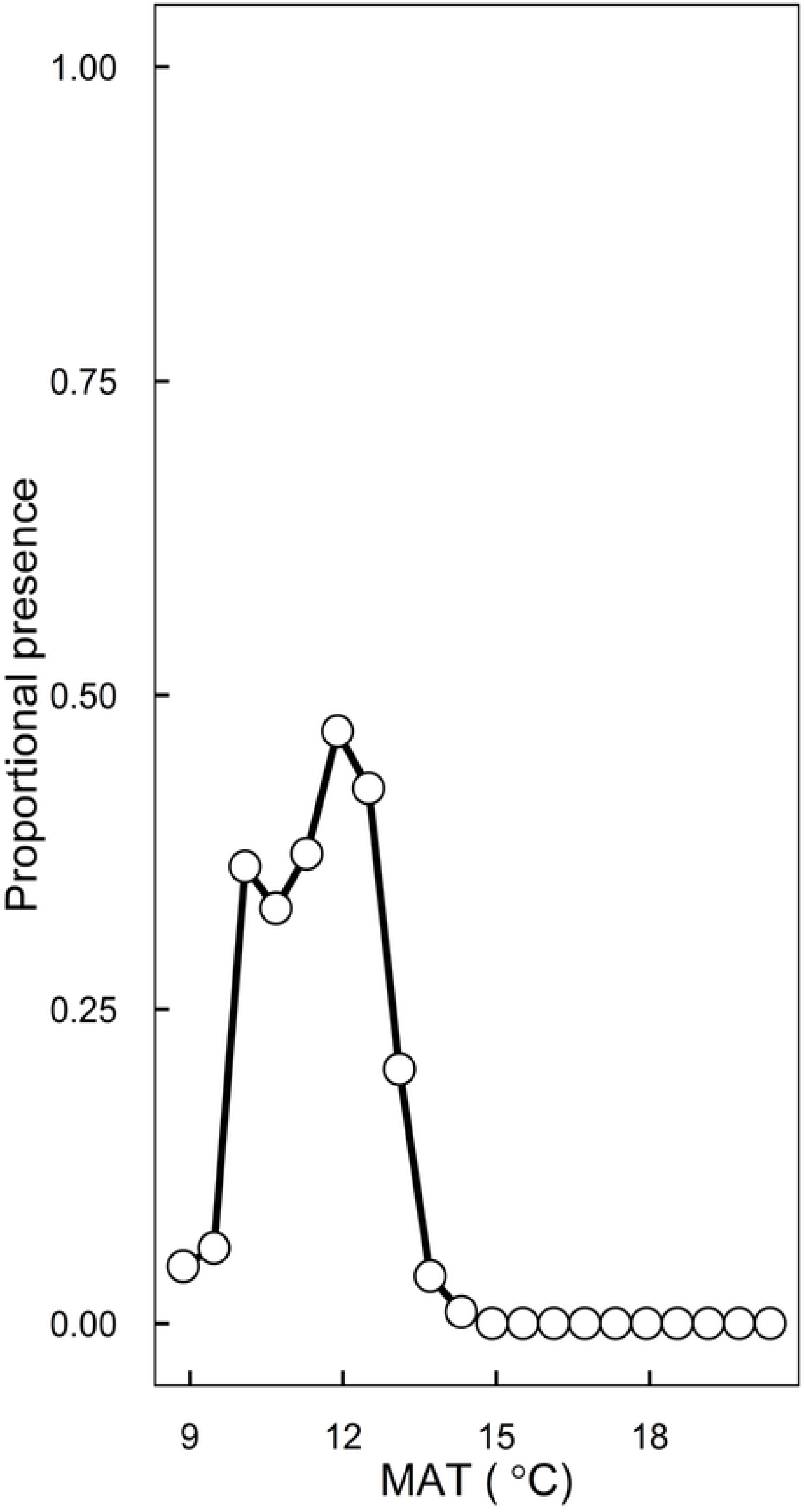

S5
Proportional presence of species along climate gradients

Proportional presence Akohekohe bio12

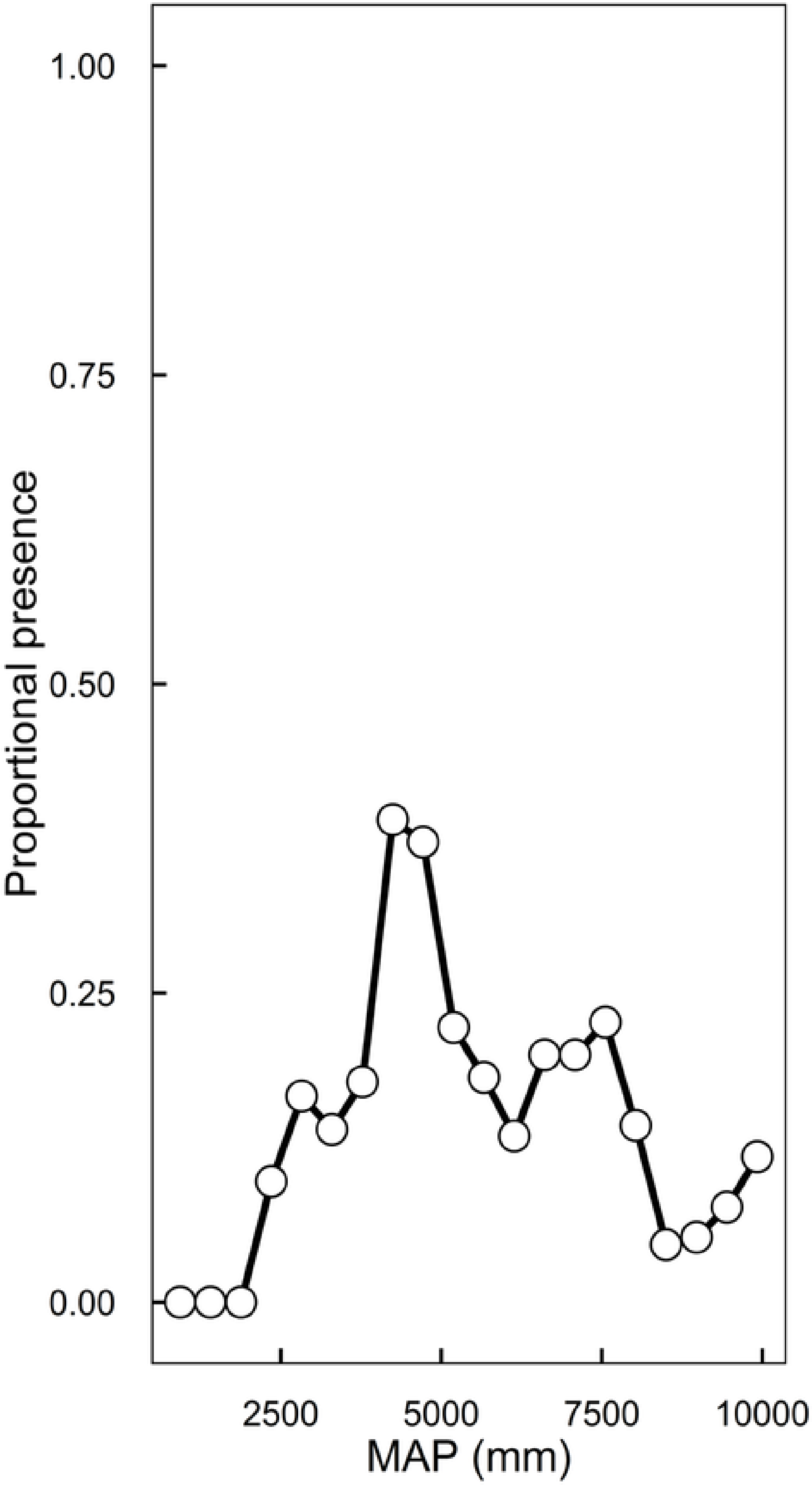

S5
Proportional presence of species along climate gradients

Proportional presence Akohekohe bio15

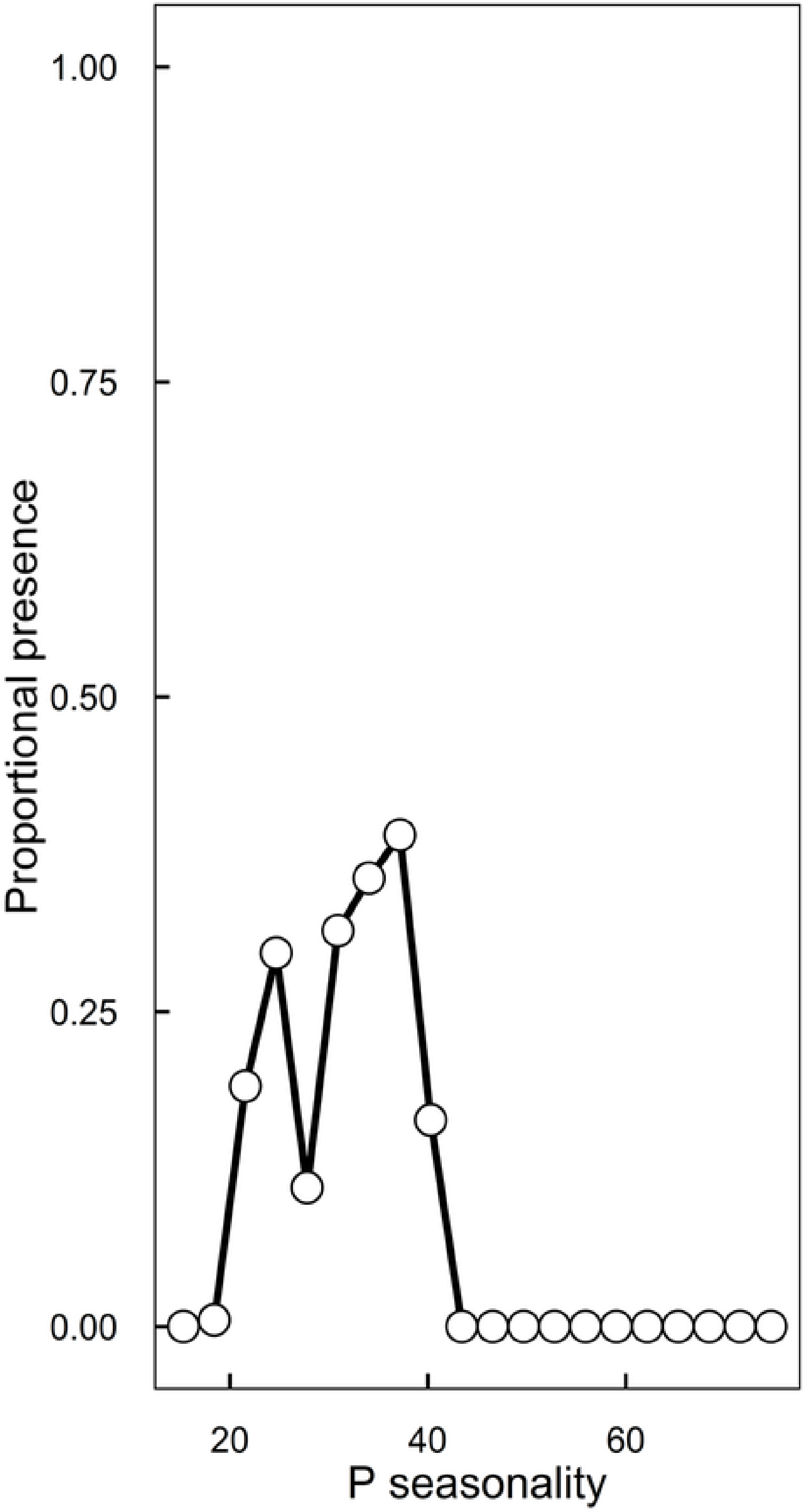

S5
Proportional presence of species along climate gradients

Proportional presence Akohekohe bio7

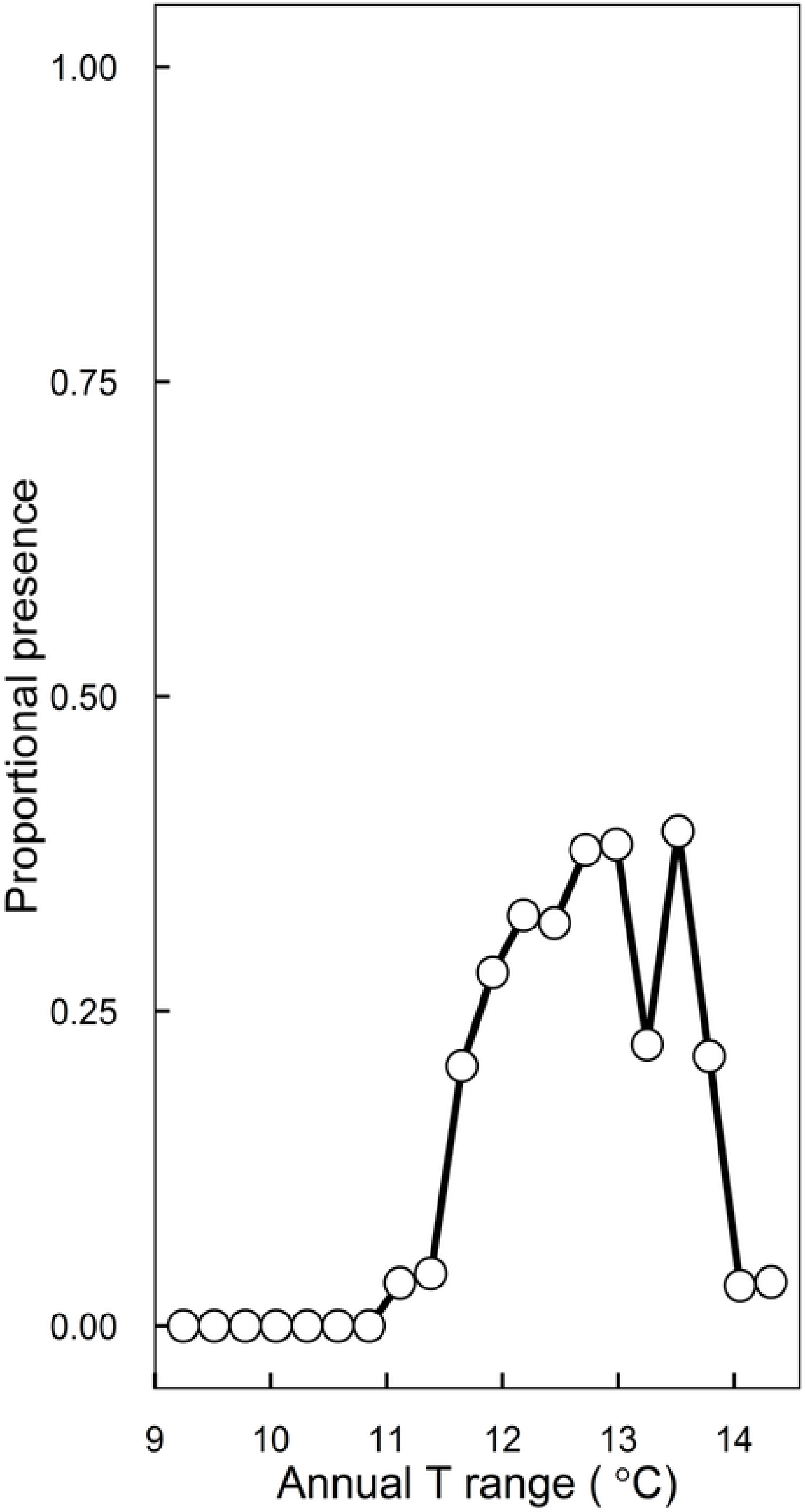

S5
Proportional presence of species along climate gradients

Proportional presence Anianiau bio1

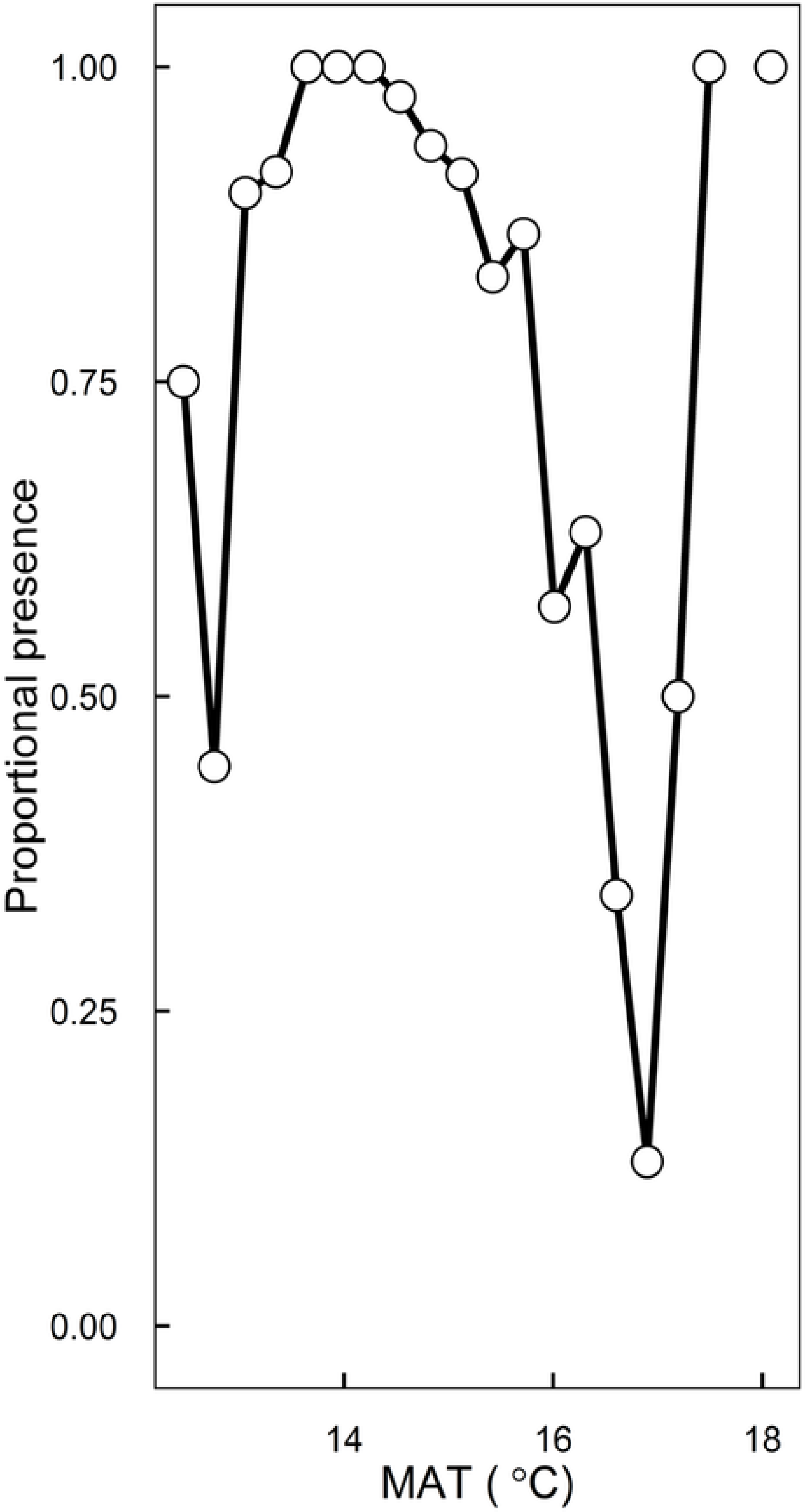

S5
Proportional presence of species along climate gradients

Proportional presence Anianiau bio12

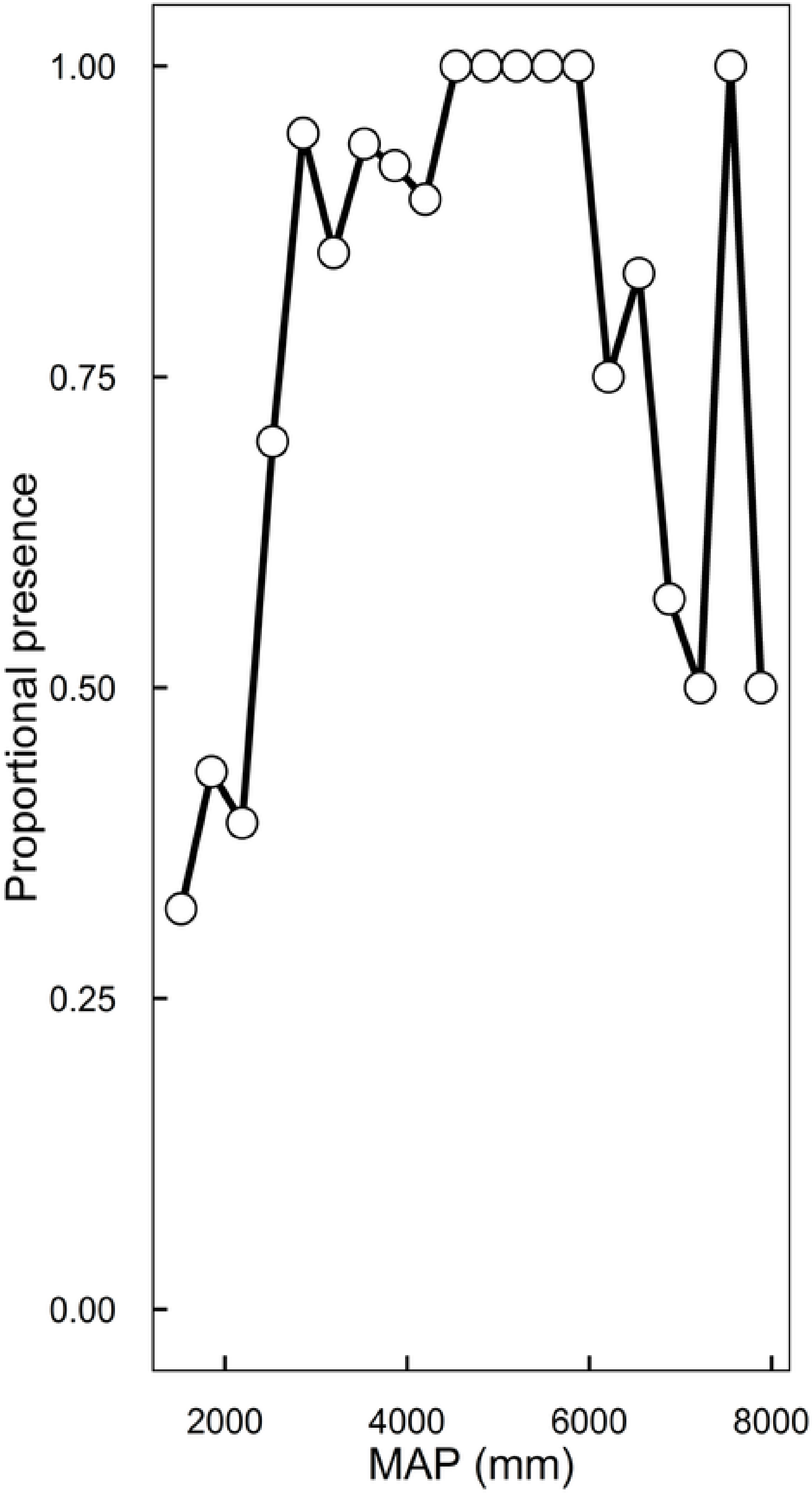

S5
Proportional presence of species along climate gradients

Proportional presence Anianiau bio15

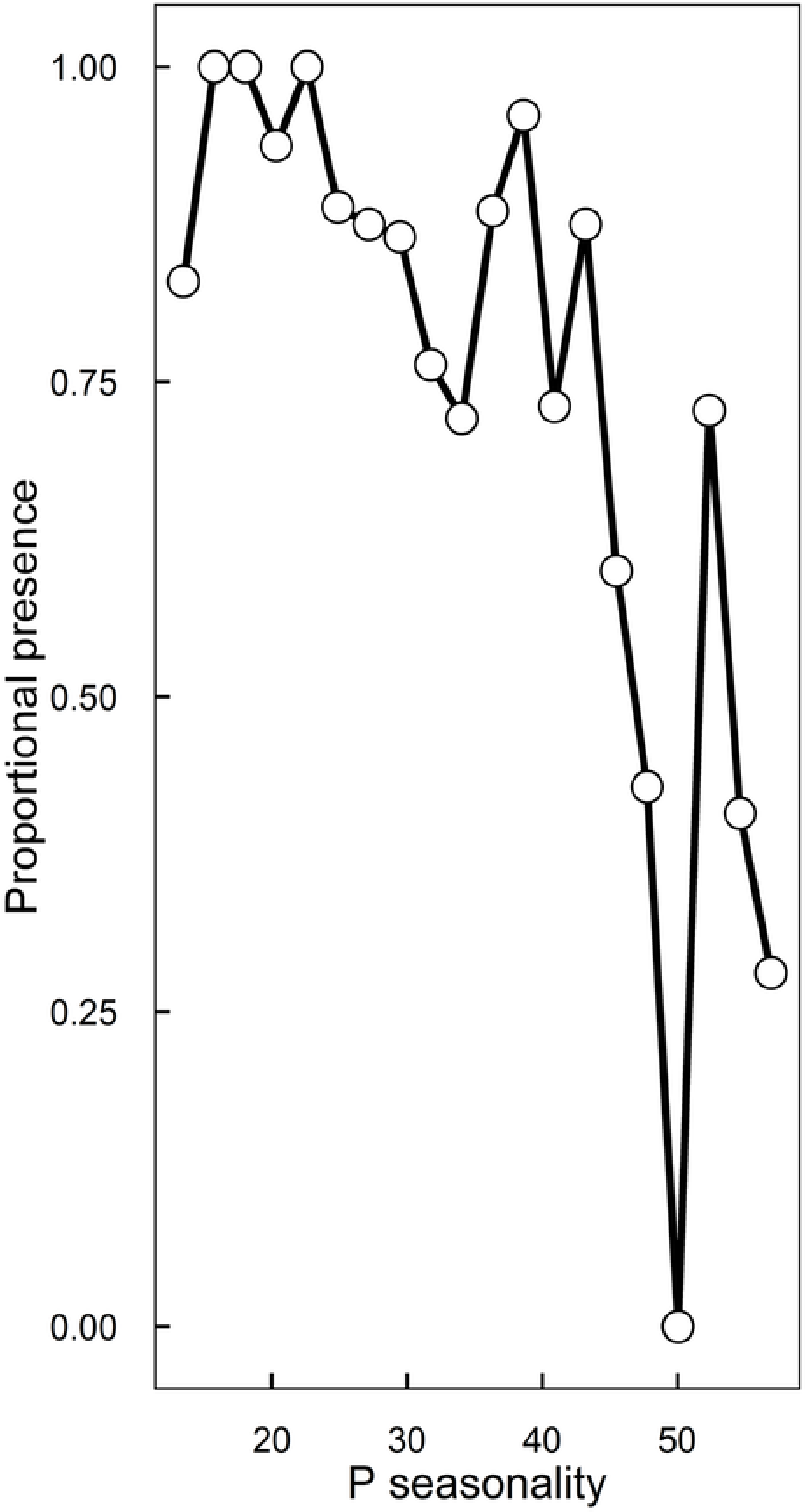

S5
Proportional presence of species along climate gradients

Proportional presence Anianiau bio7

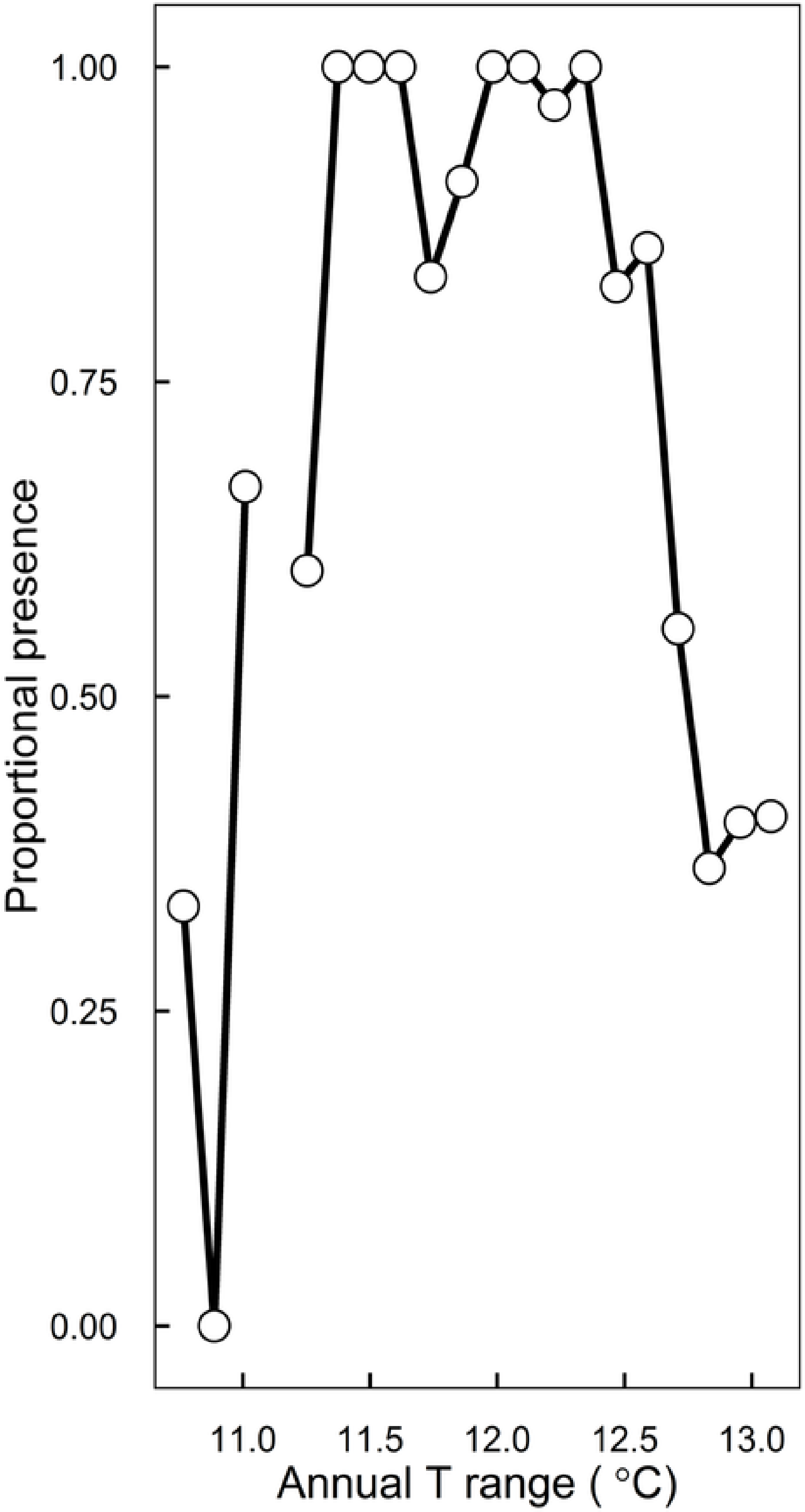

S5
Proportional presence of species along climate gradients

Proportional presence Apapane bio1

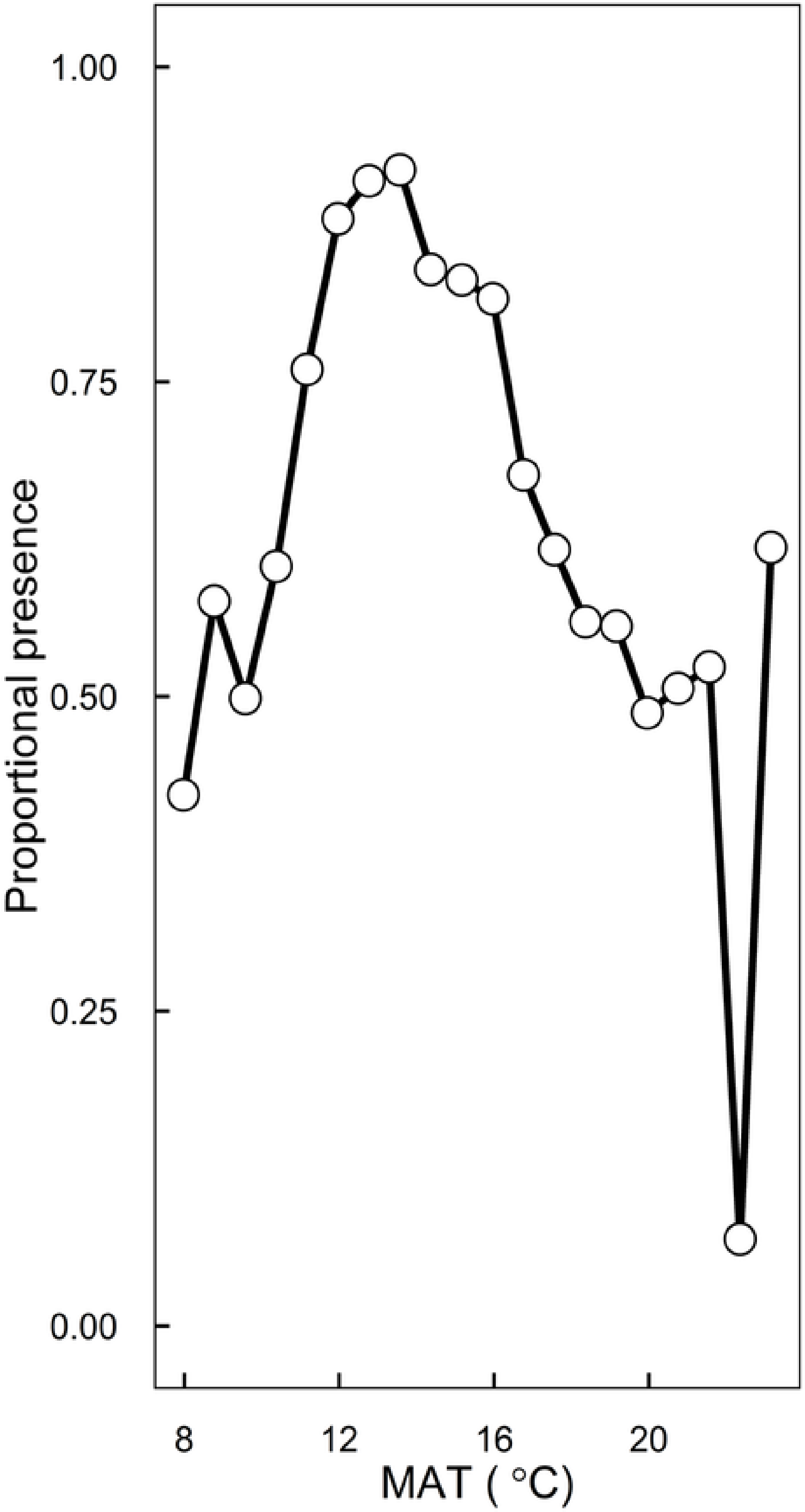

S5
Proportional presence of species along climate gradients

Proportional presence Apapane bio12

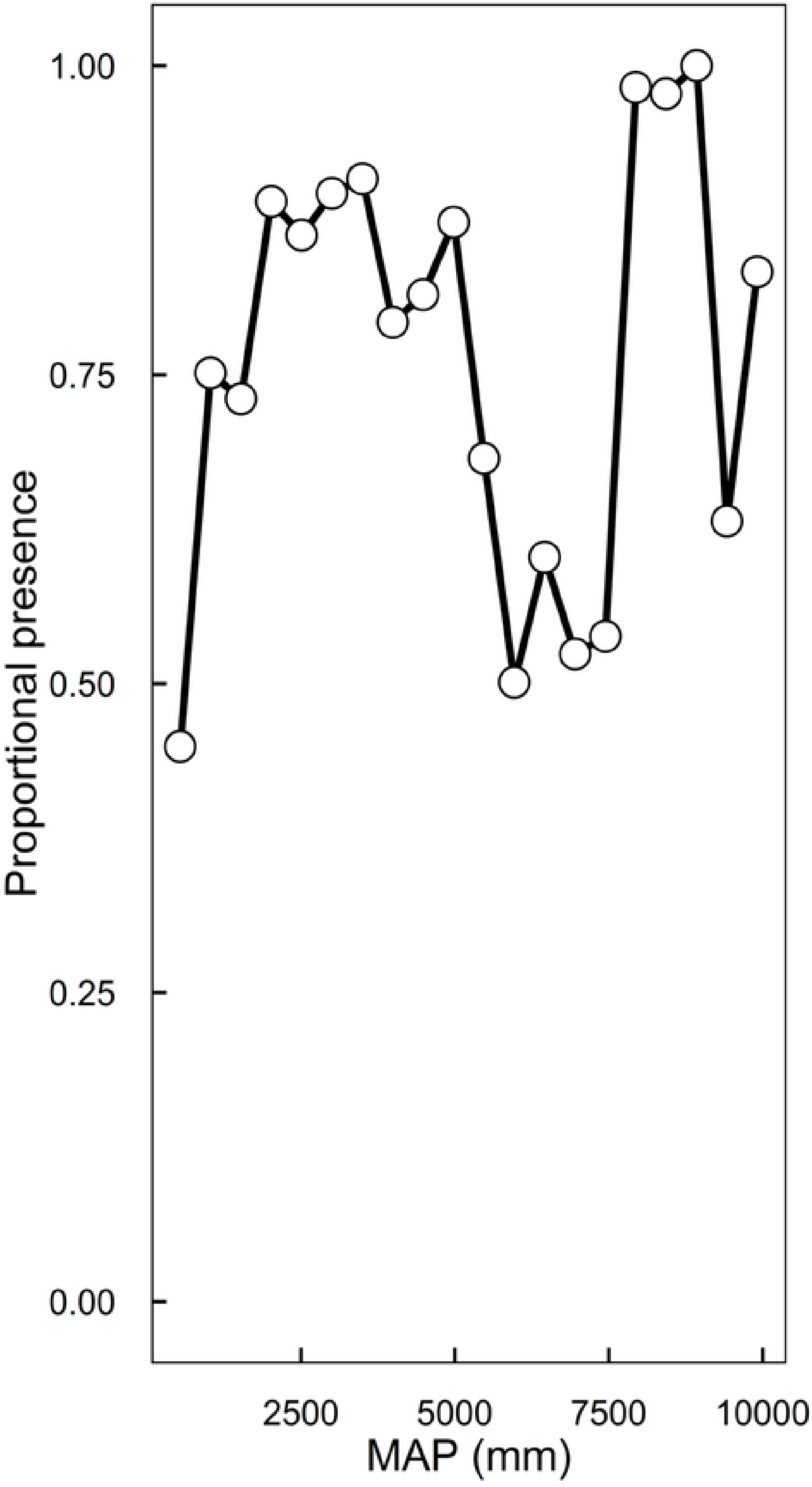

S5
Proportional presence of species along climate gradients

Proportional presence Apapane bio15

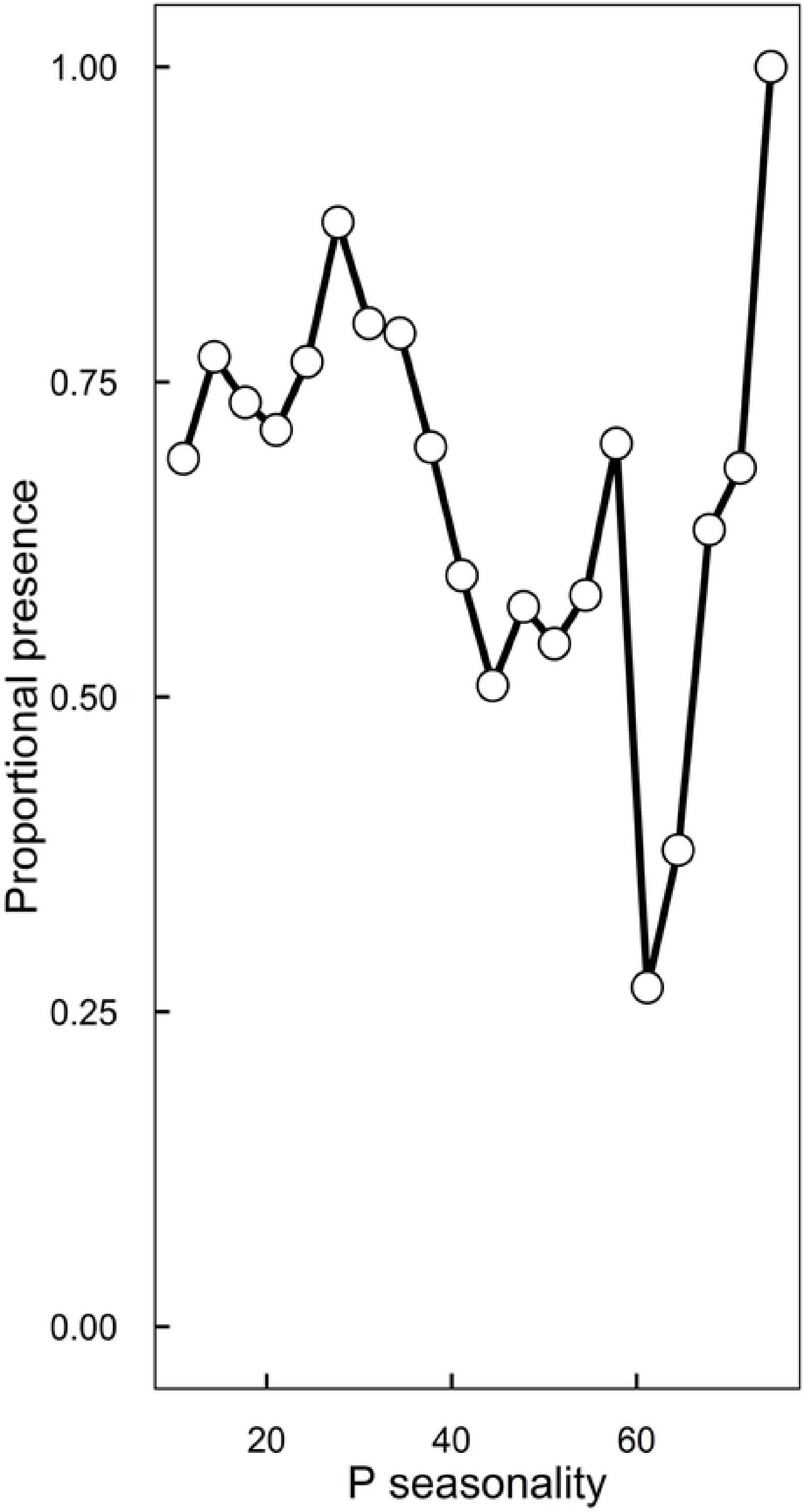

S5

Proportional presence of species along climate gradients

Proportional presence Apapane bio7

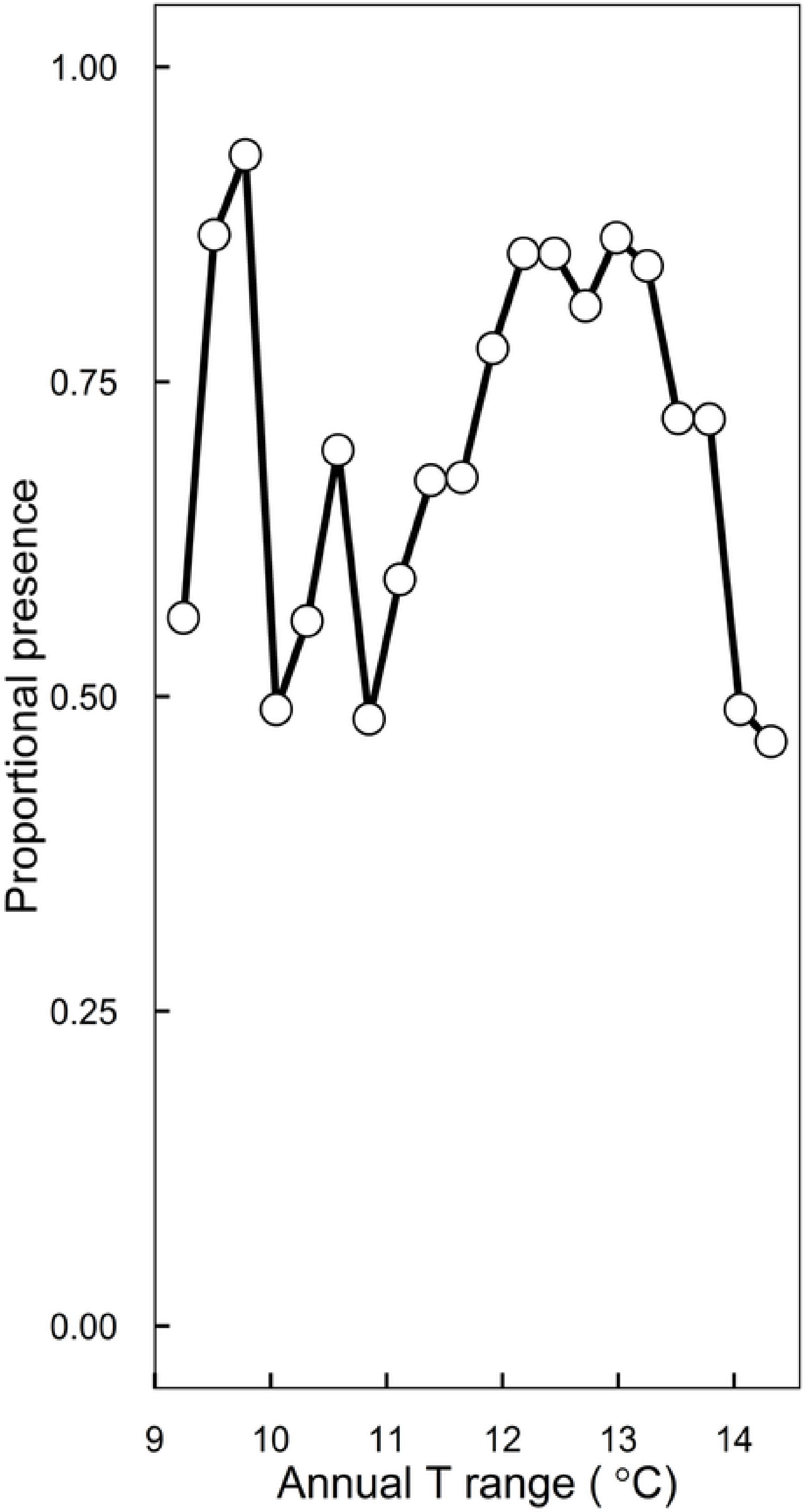

S5
Proportional presence of species along climate gradients

Proportional presence Hawaii Akepa bio1

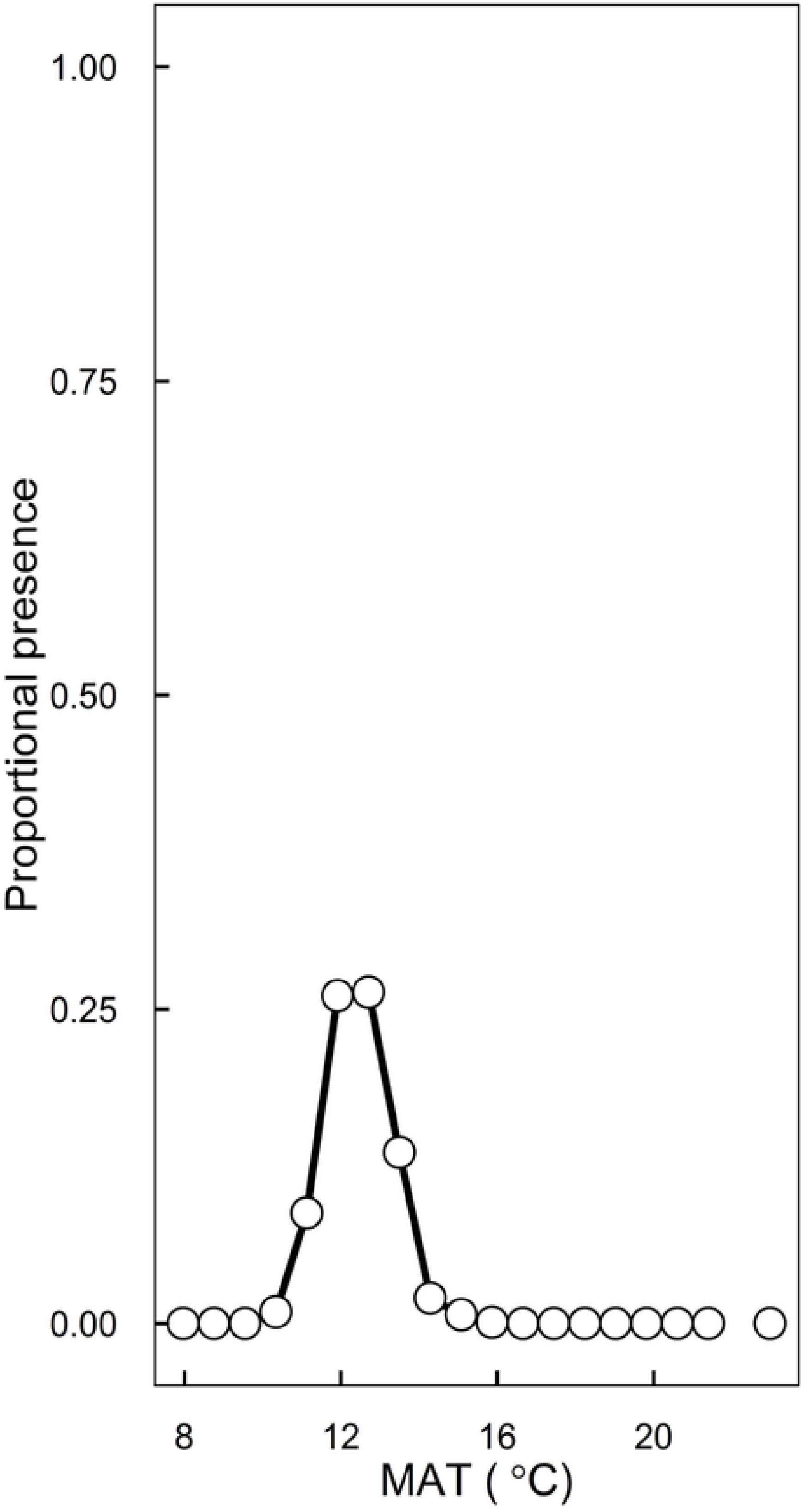

S5
Proportional presence of species along climate gradients

Proportional presence Hawaii Akepa bio12

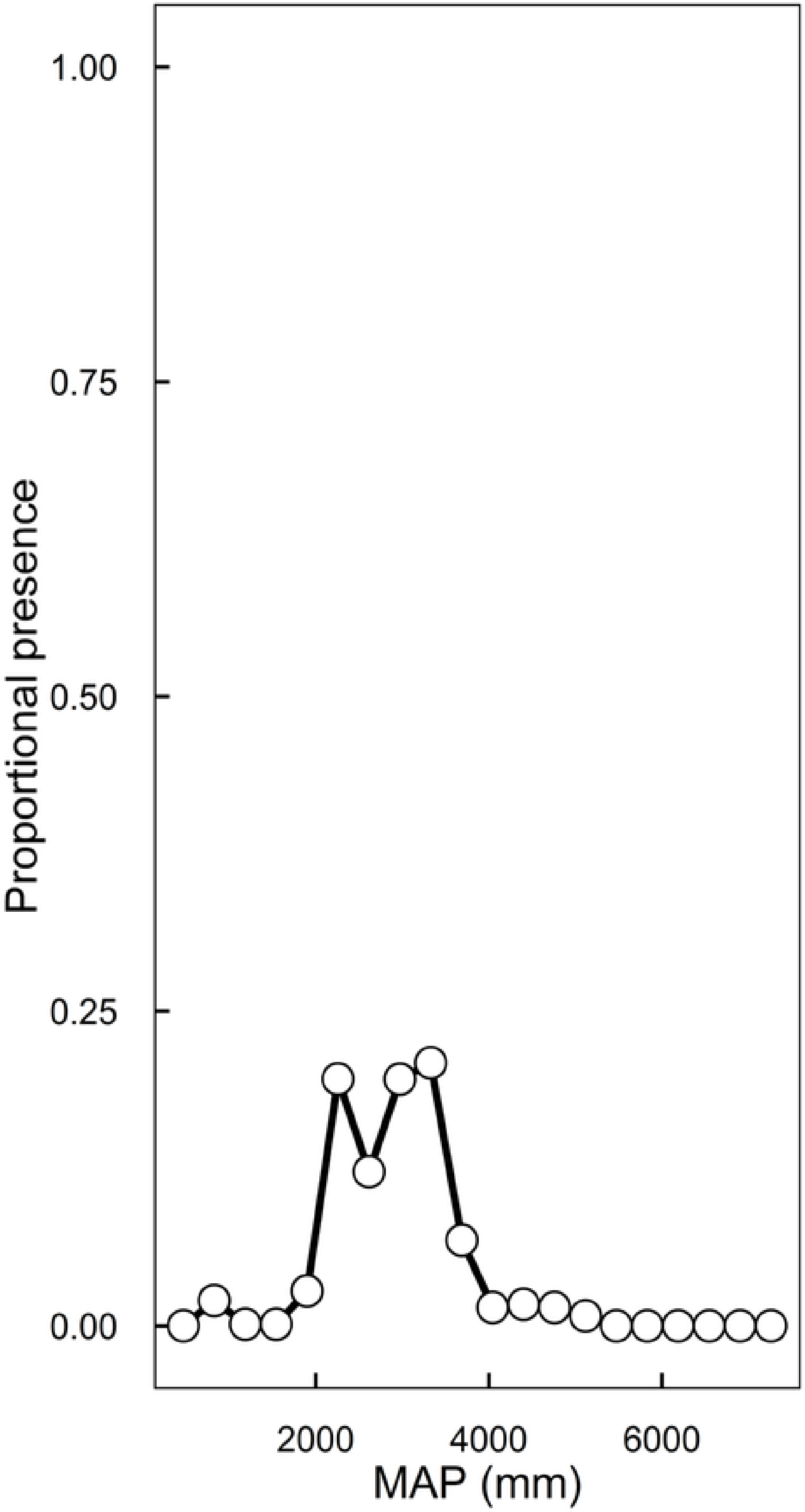

S5
Proportional presence of species along climate gradients

Proportional presence Hawaii Akepa bio15

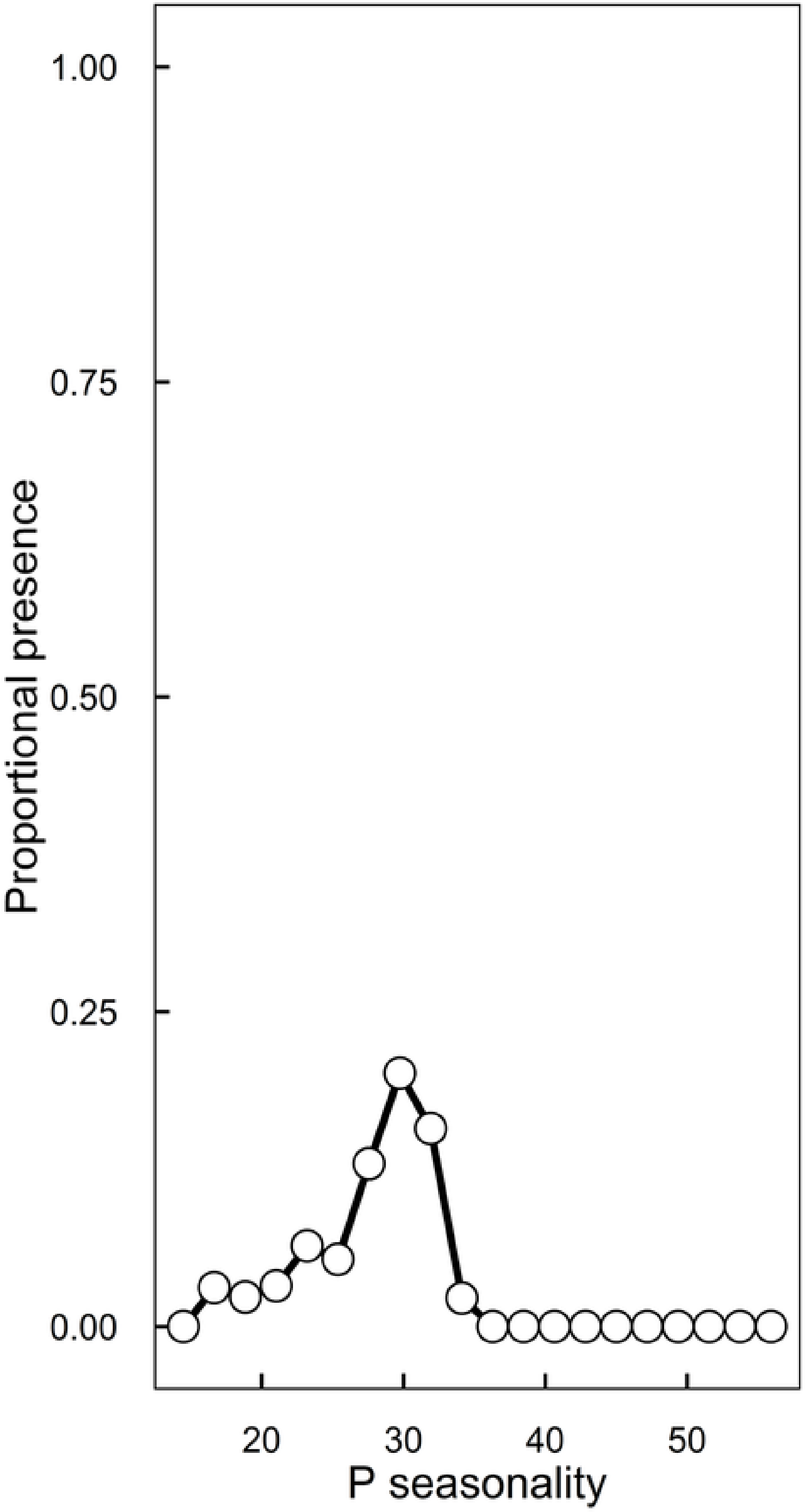

S5
Proportional presence of species along climate gradients

Proportional presence Hawaii Akepa bio7

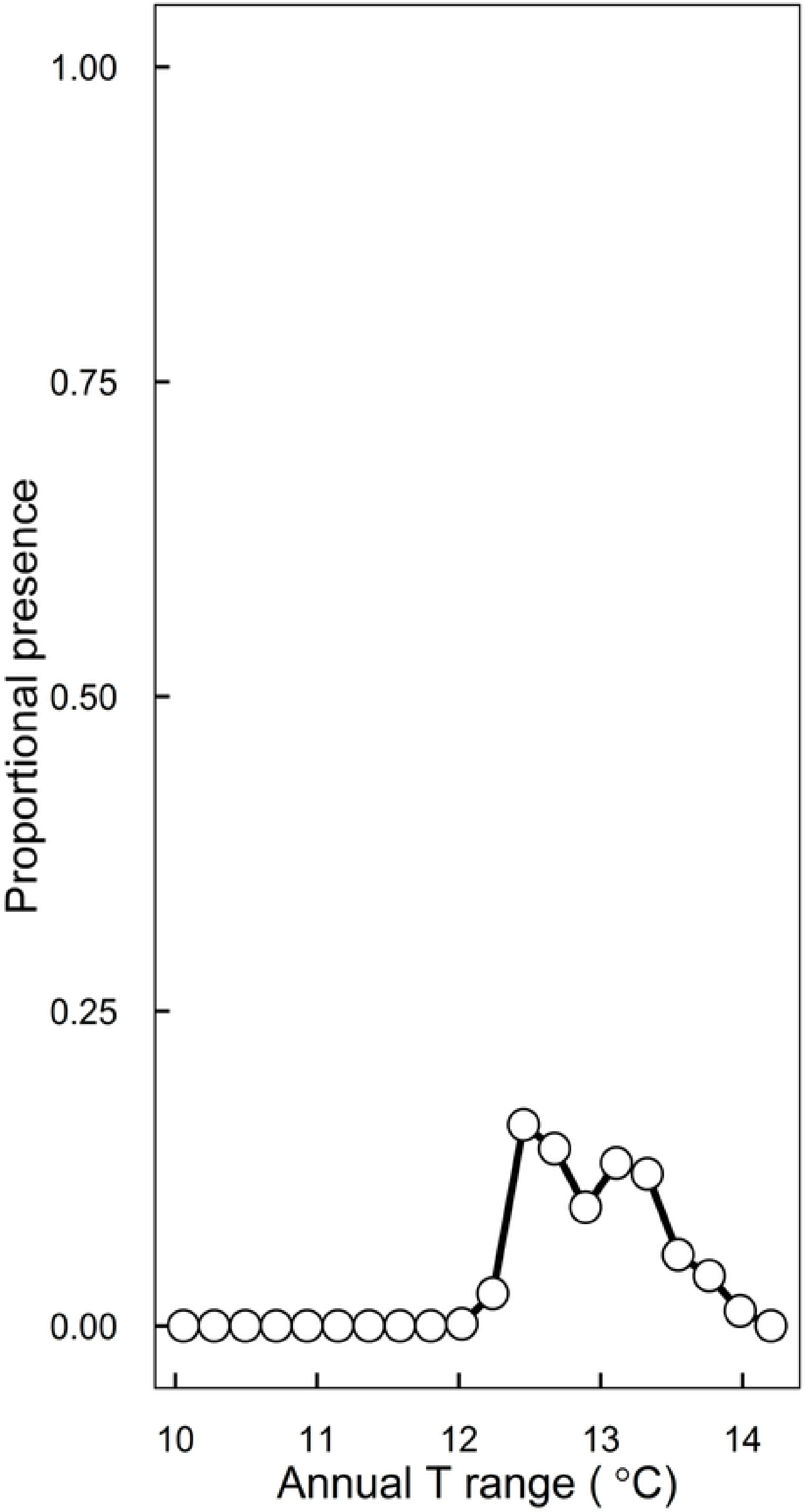

S5
Proportional presence of species along climate gradients

Proportional presence Hawaii Amakihi bio1

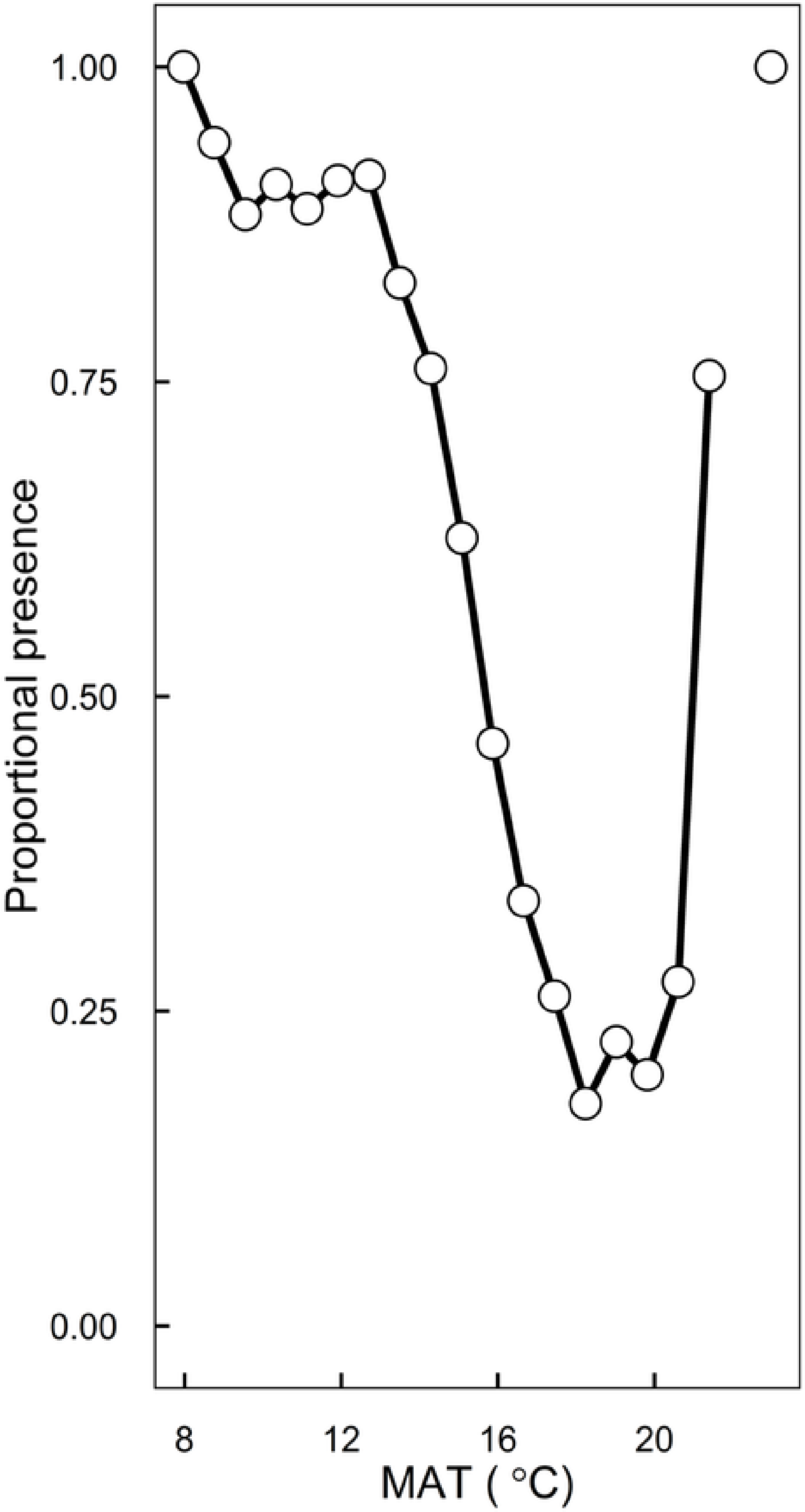

S5
Proportional presence of species along climate gradients

Proportional presence Hawaii Amakihi bio12

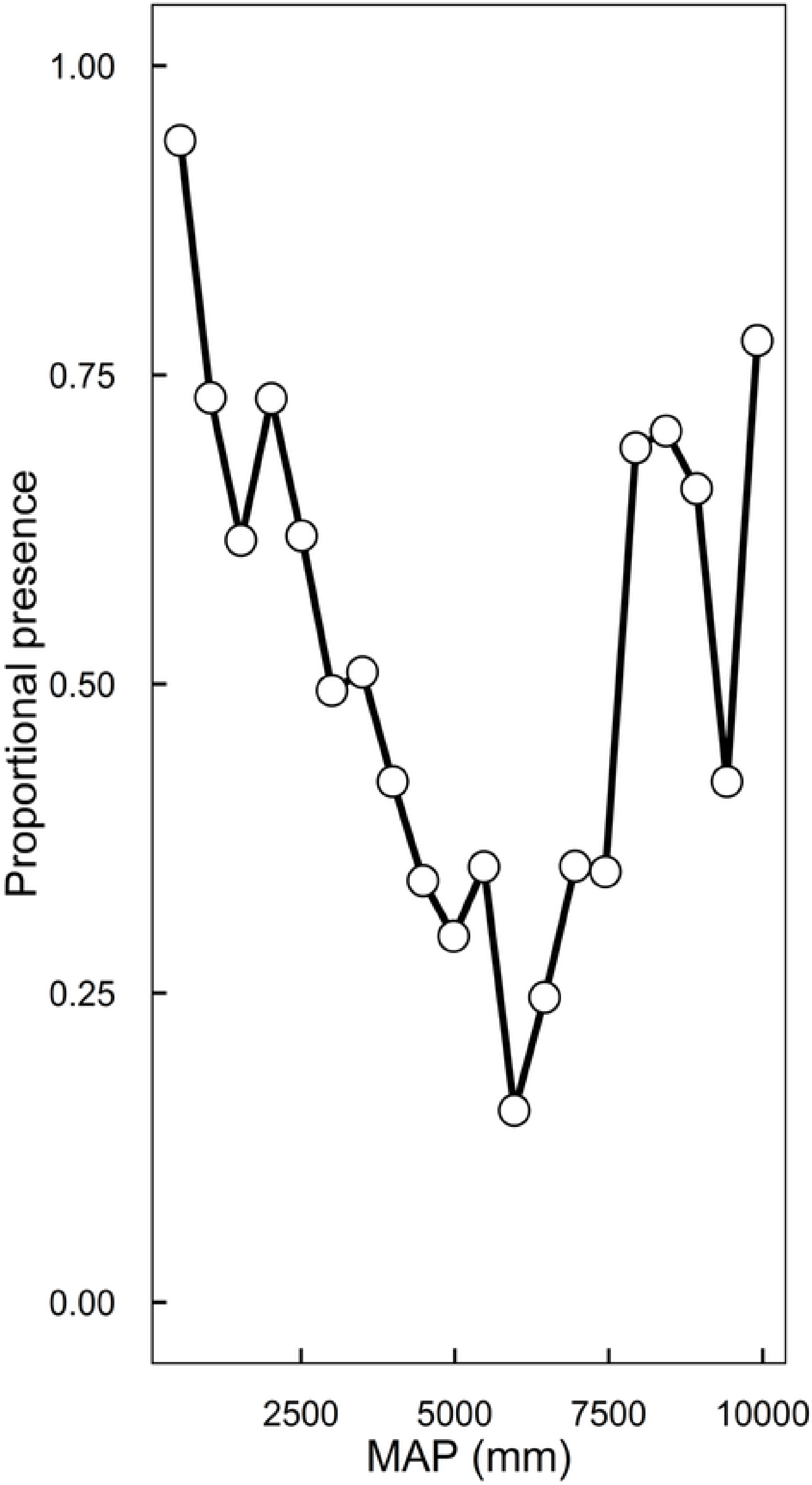

S5
Proportional presence of species along climate gradients

Proportional presence Hawaii Amakihi bio15

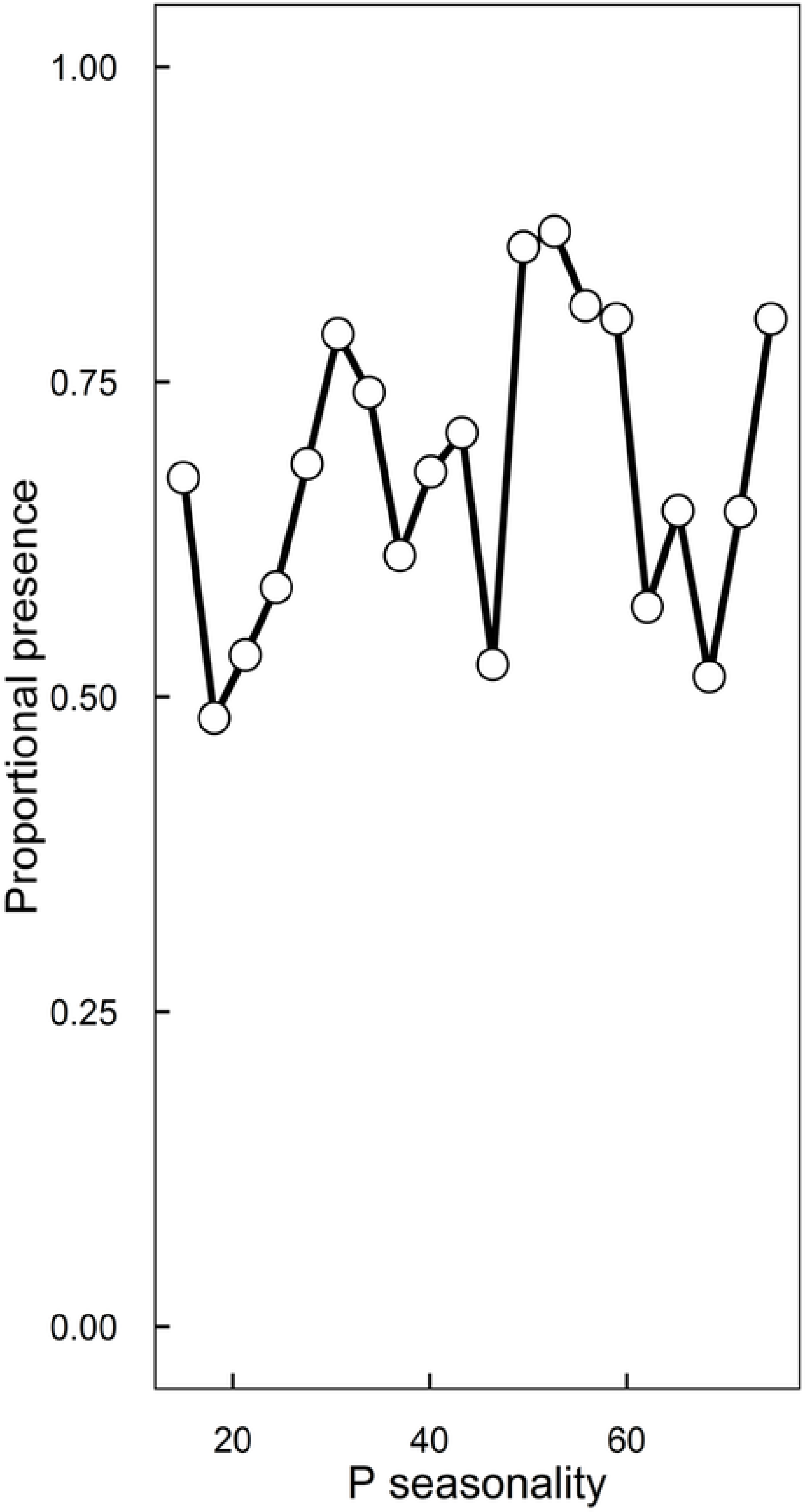

S5
Proportional presence of species along climate gradients

Proportional presence Hawaii Amakihi bio7

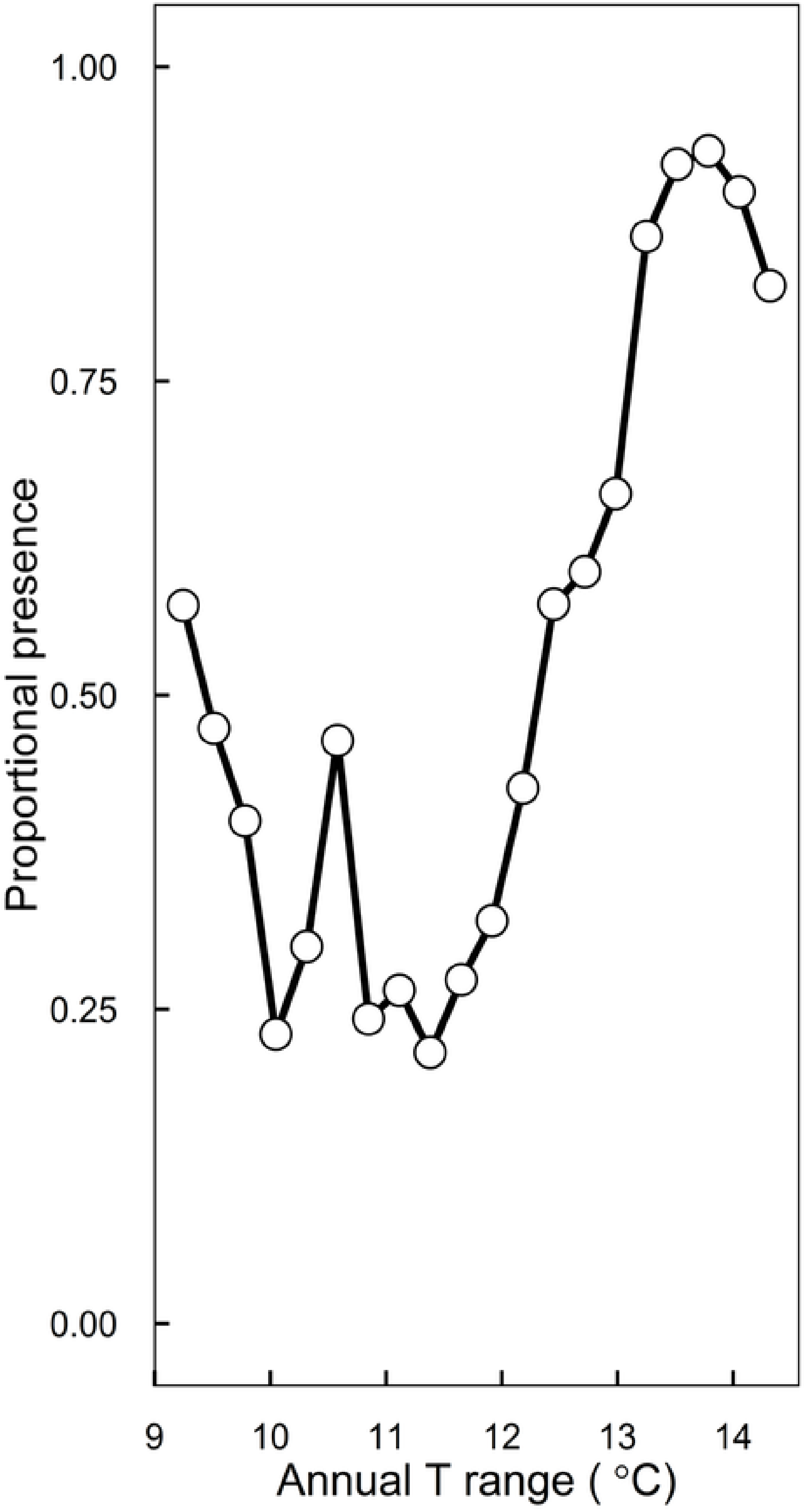

S5
Proportional presence of species along climate gradients

Proportional presence Hawaii Creeper bio1

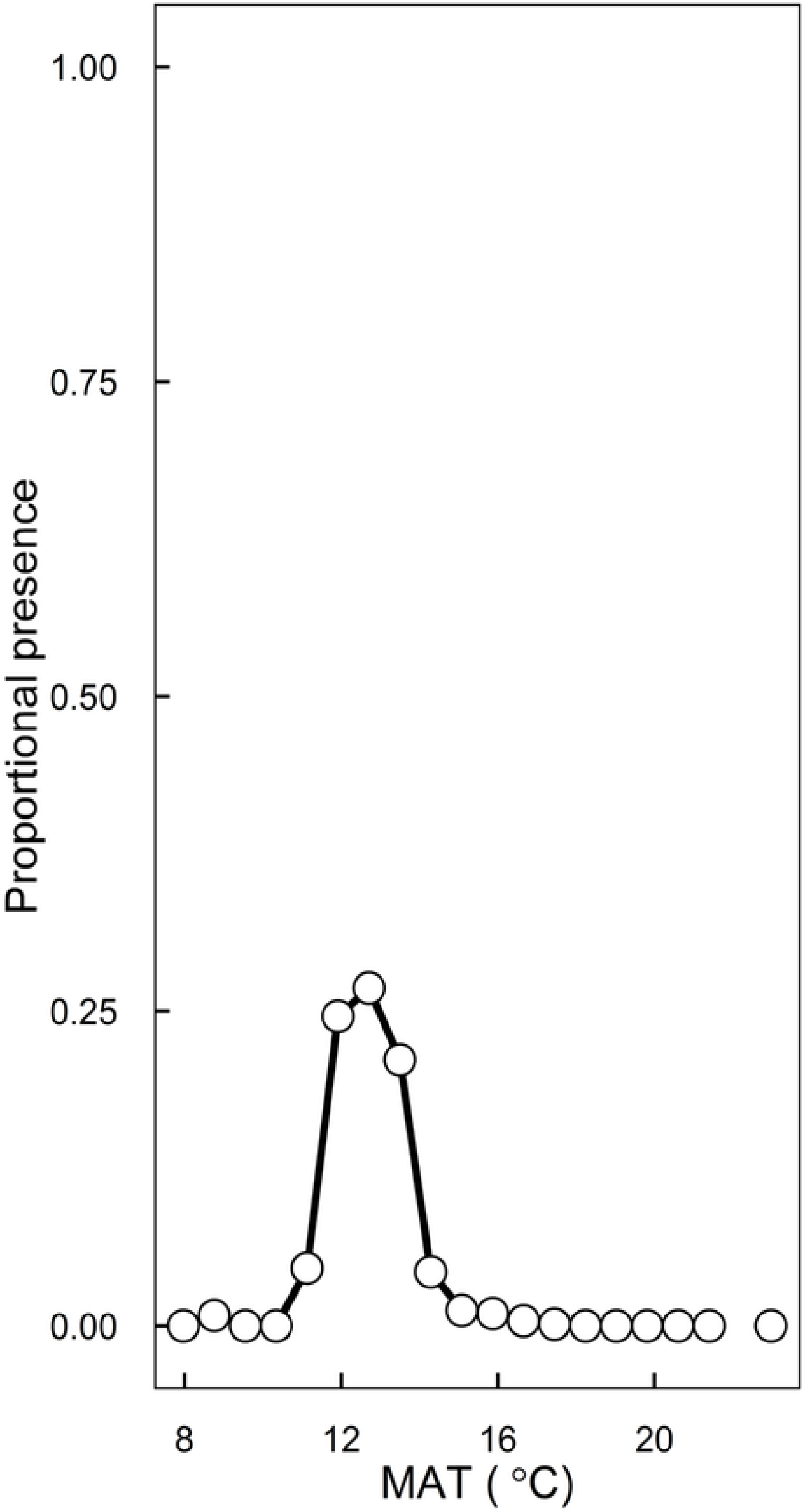

S5
Proportional presence of species along climate gradients

Proportional presence Hawaii Creeper bio12

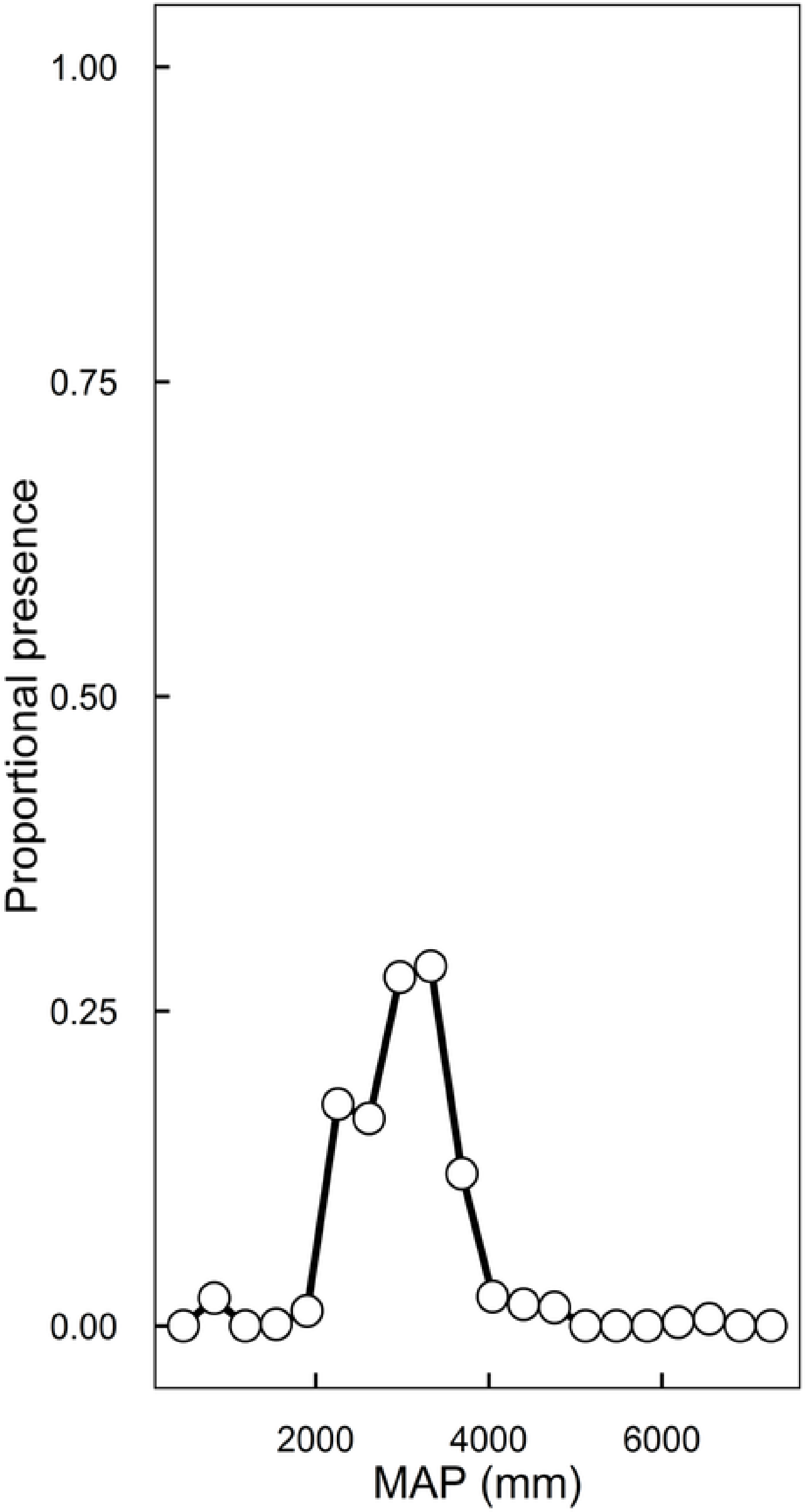

S5
Proportional presence of species along climate gradients

Proportional presence Hawaii Creeper bio15

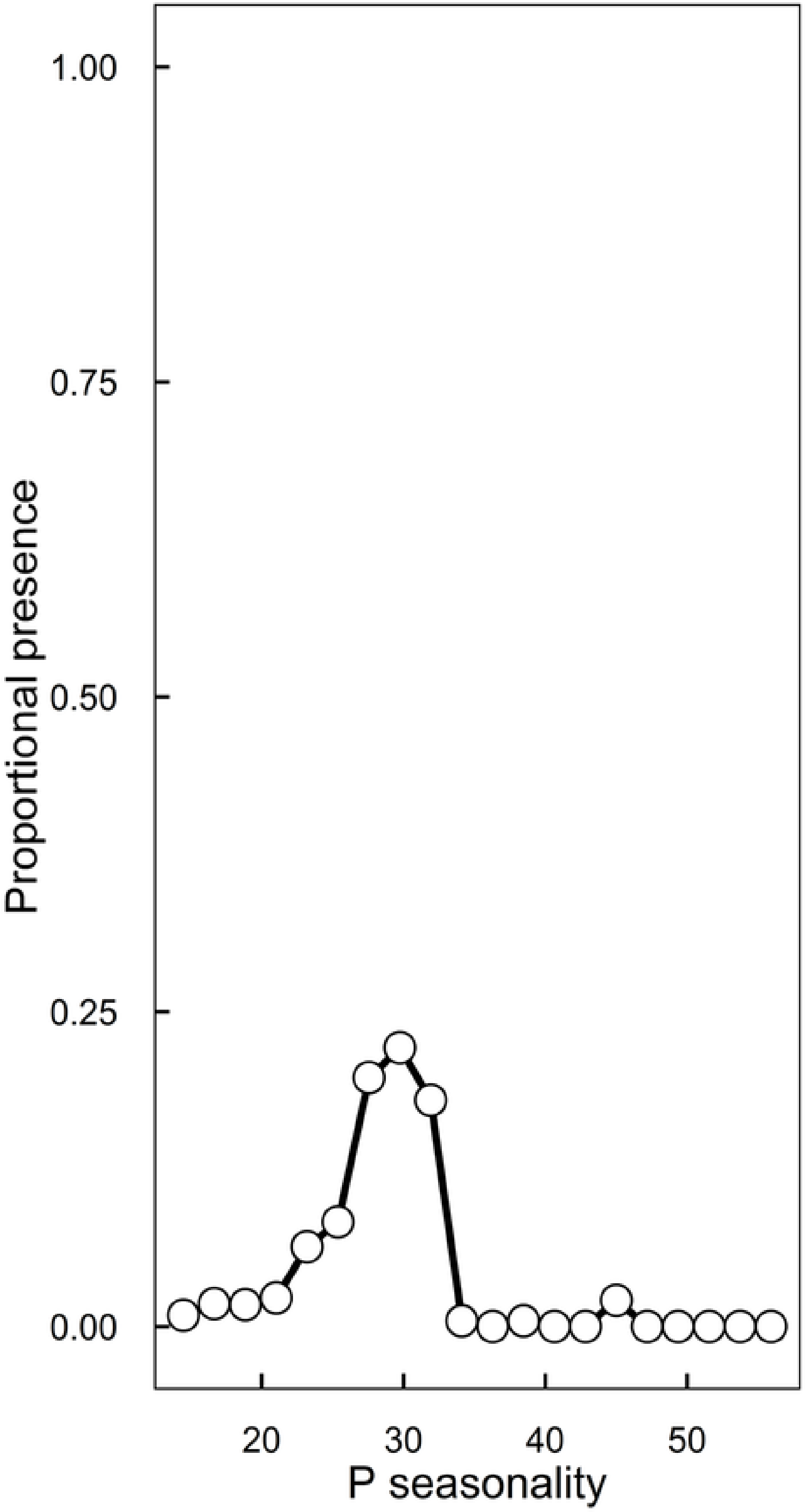

S5
Proportional presence of species along climate gradients

Proportional presence Hawaii Creeper bio7

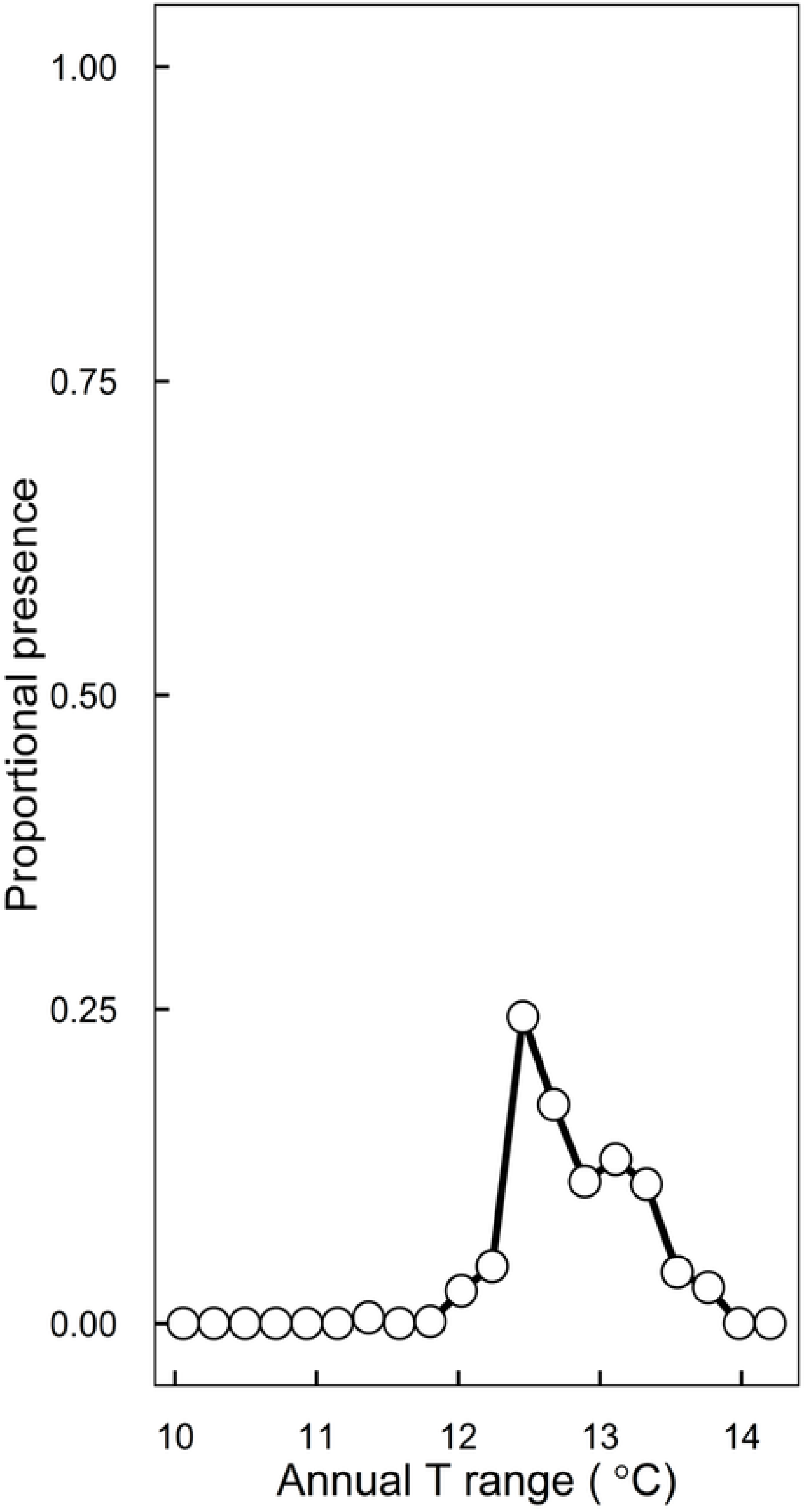

S5
Proportional presence of species along climate gradients

Proportional presence Hawaii Elepaio bio1

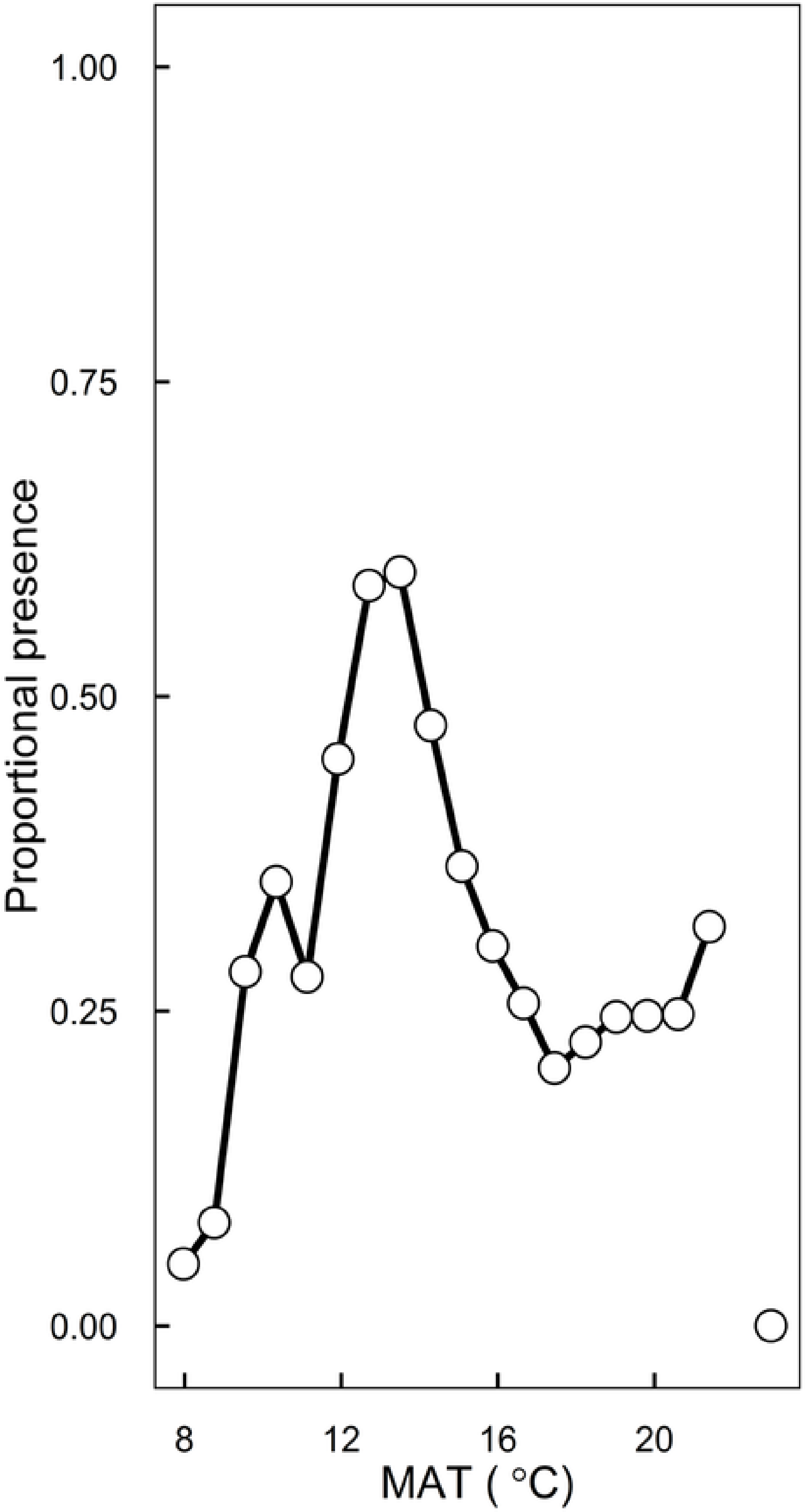

S5
Proportional presence of species along climate gradients

Proportional presence Hawaii Elepaio bio12

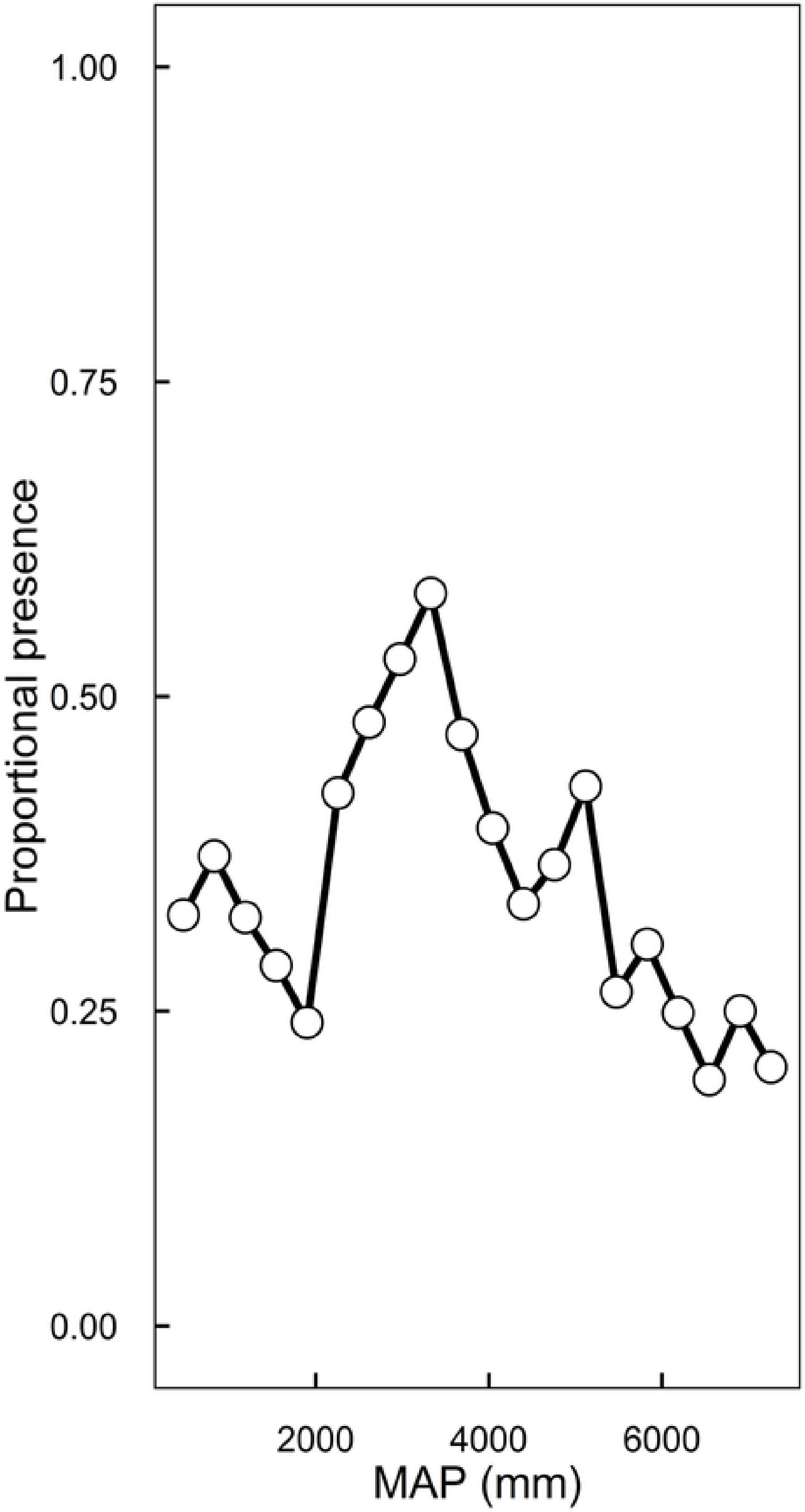

S5
Proportional presence of species along climate gradients

Proportional presence Hawaii Elepaio bio15

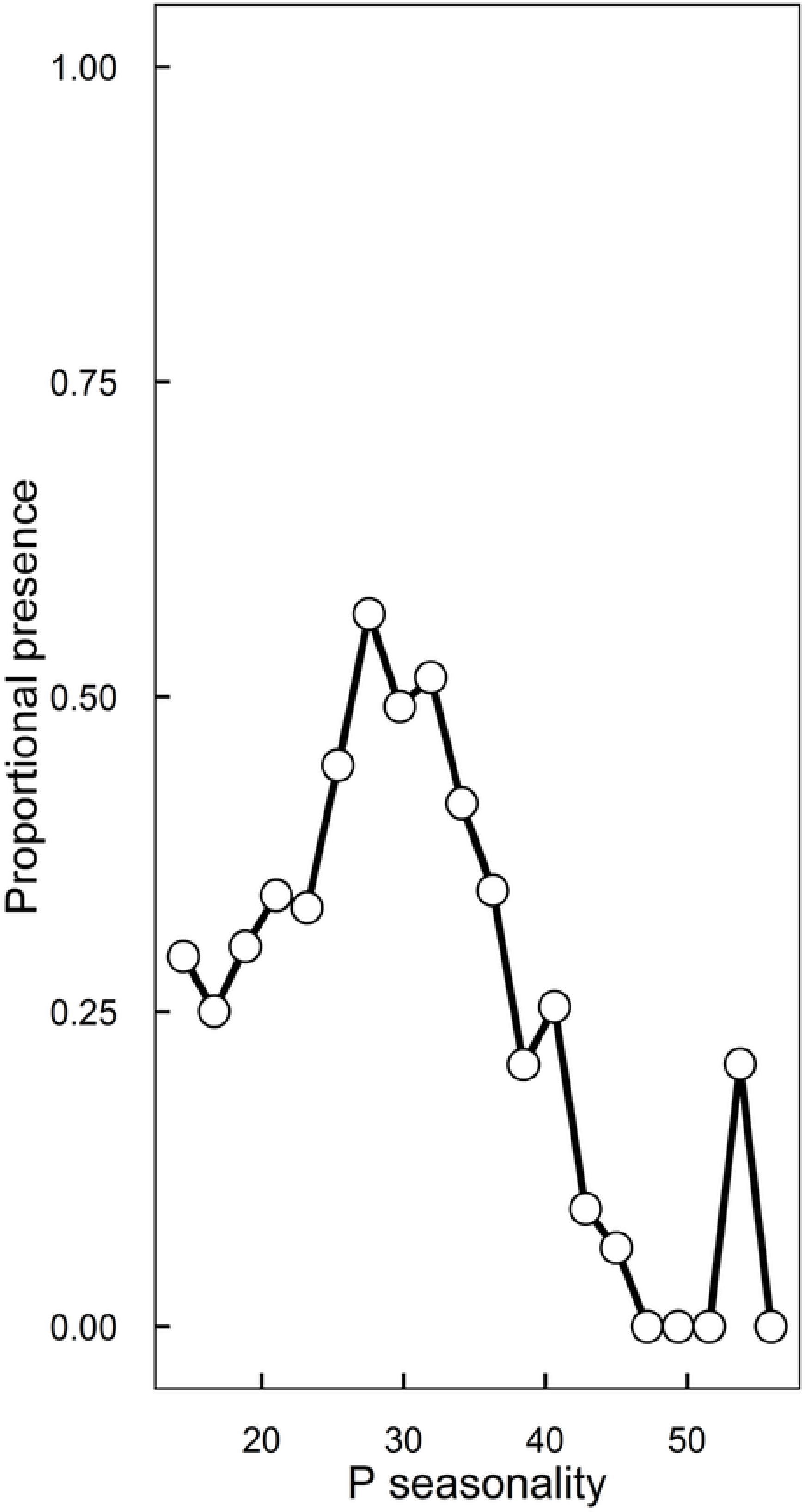

S5
Proportional presence of species along climate gradients

Proportional presence Hawaii Elepaio bio7

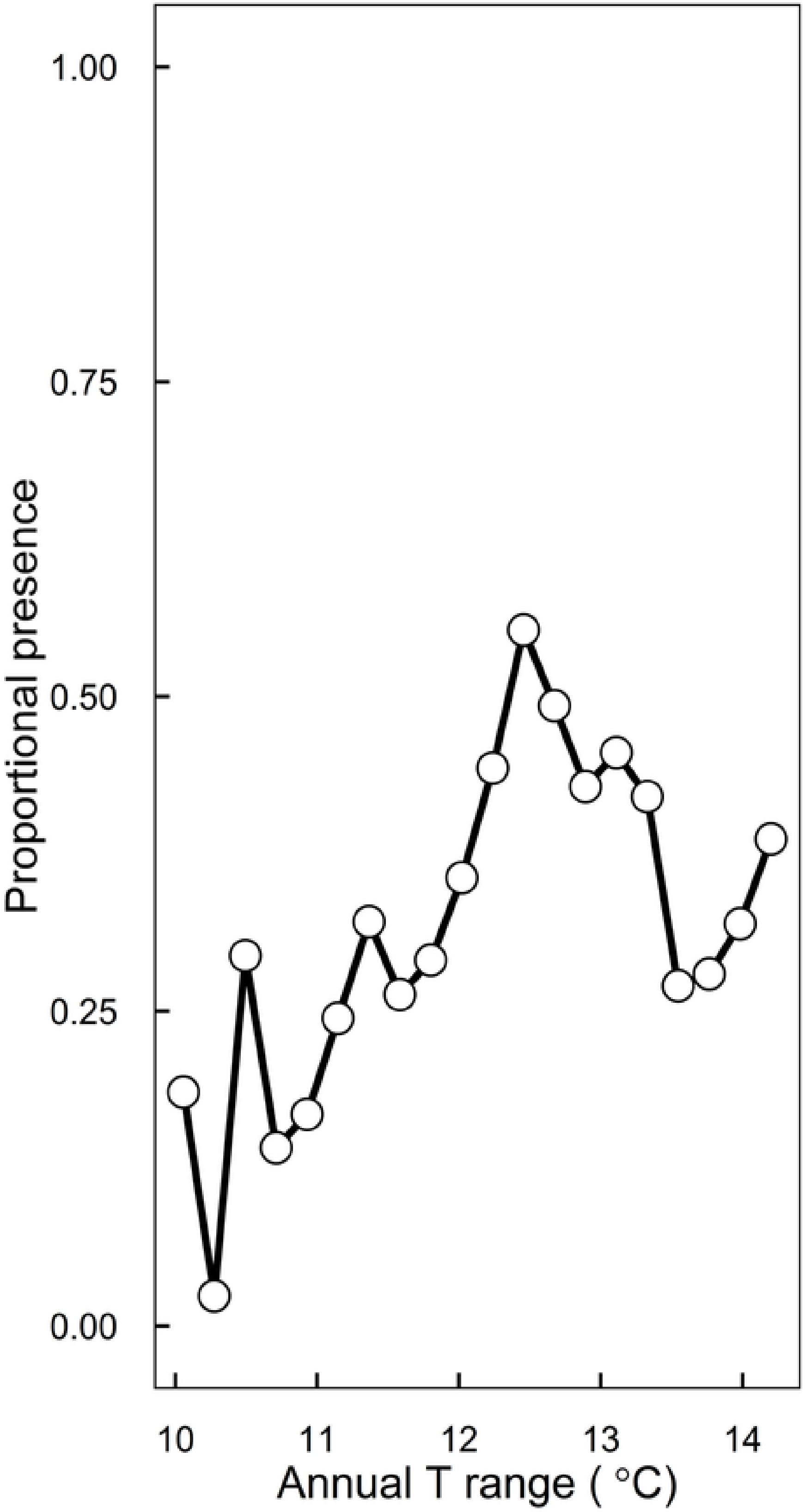

S5
Proportional presence of species along climate gradients

Proportional presence Iiwi bio1

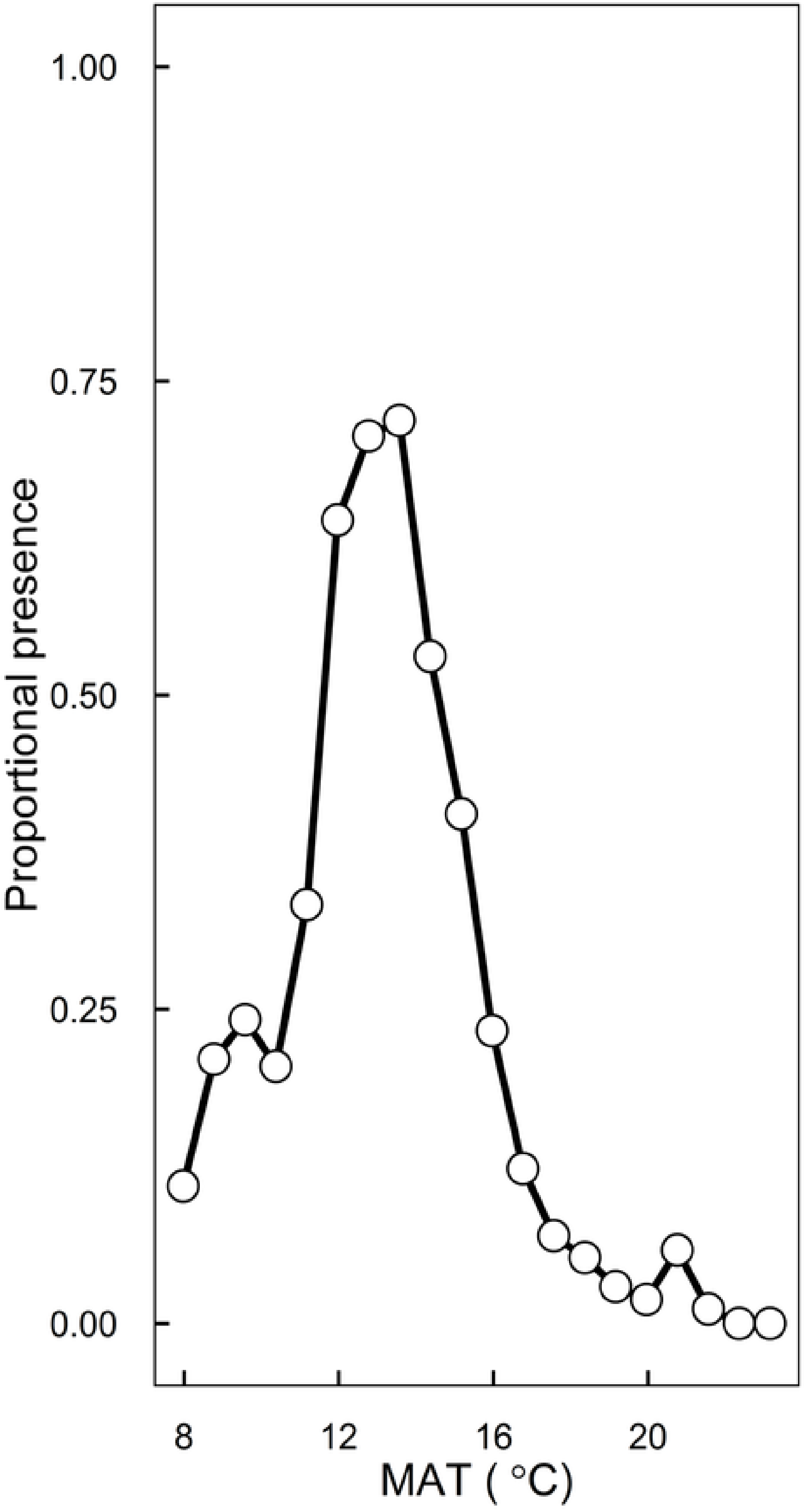

S5
Proportional presence of species along climate gradients

Proportional presence Iiwi bio12

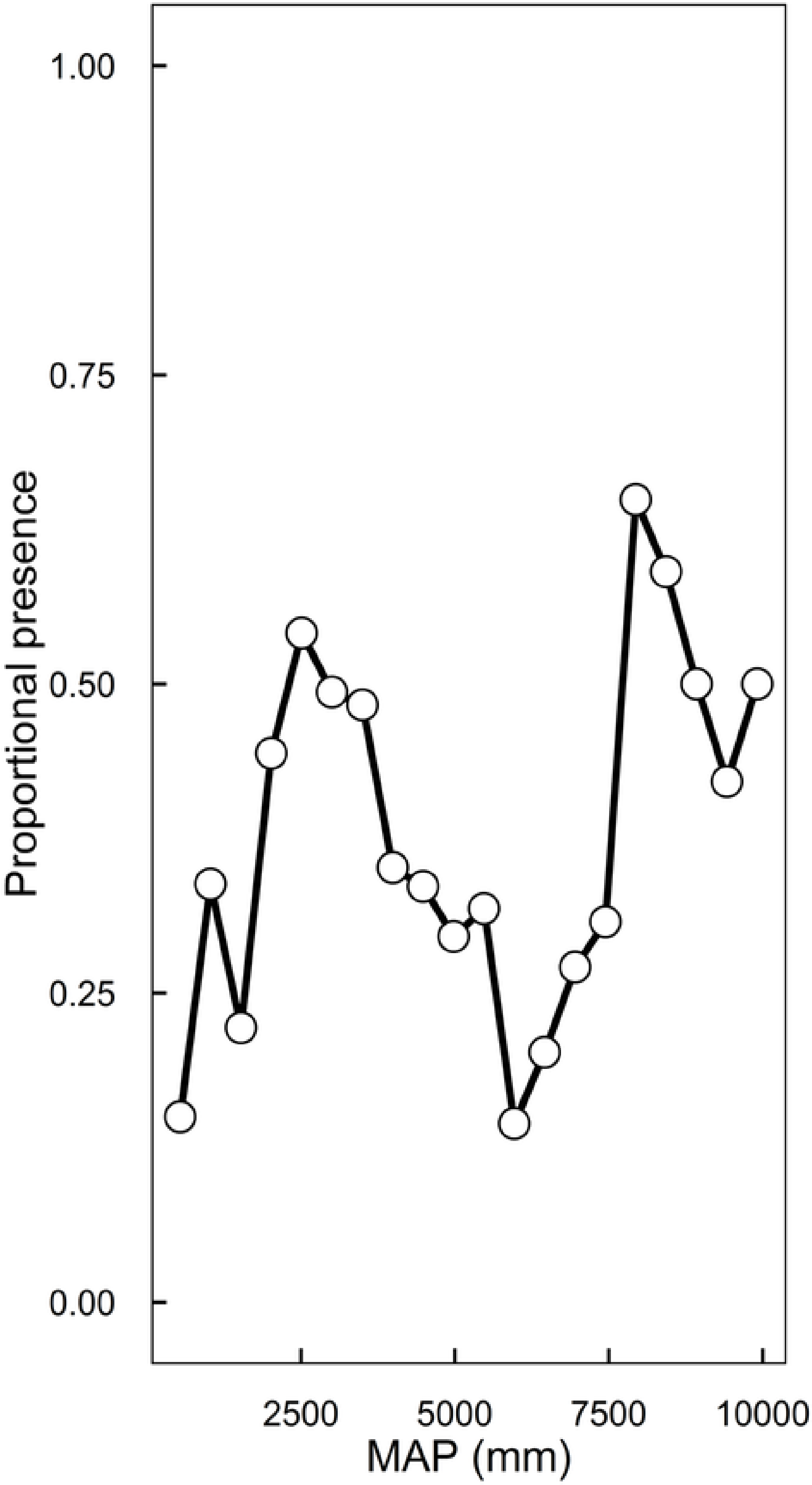

S5
Proportional presence of species along climate gradients

Proportional presence Iiwi bio15

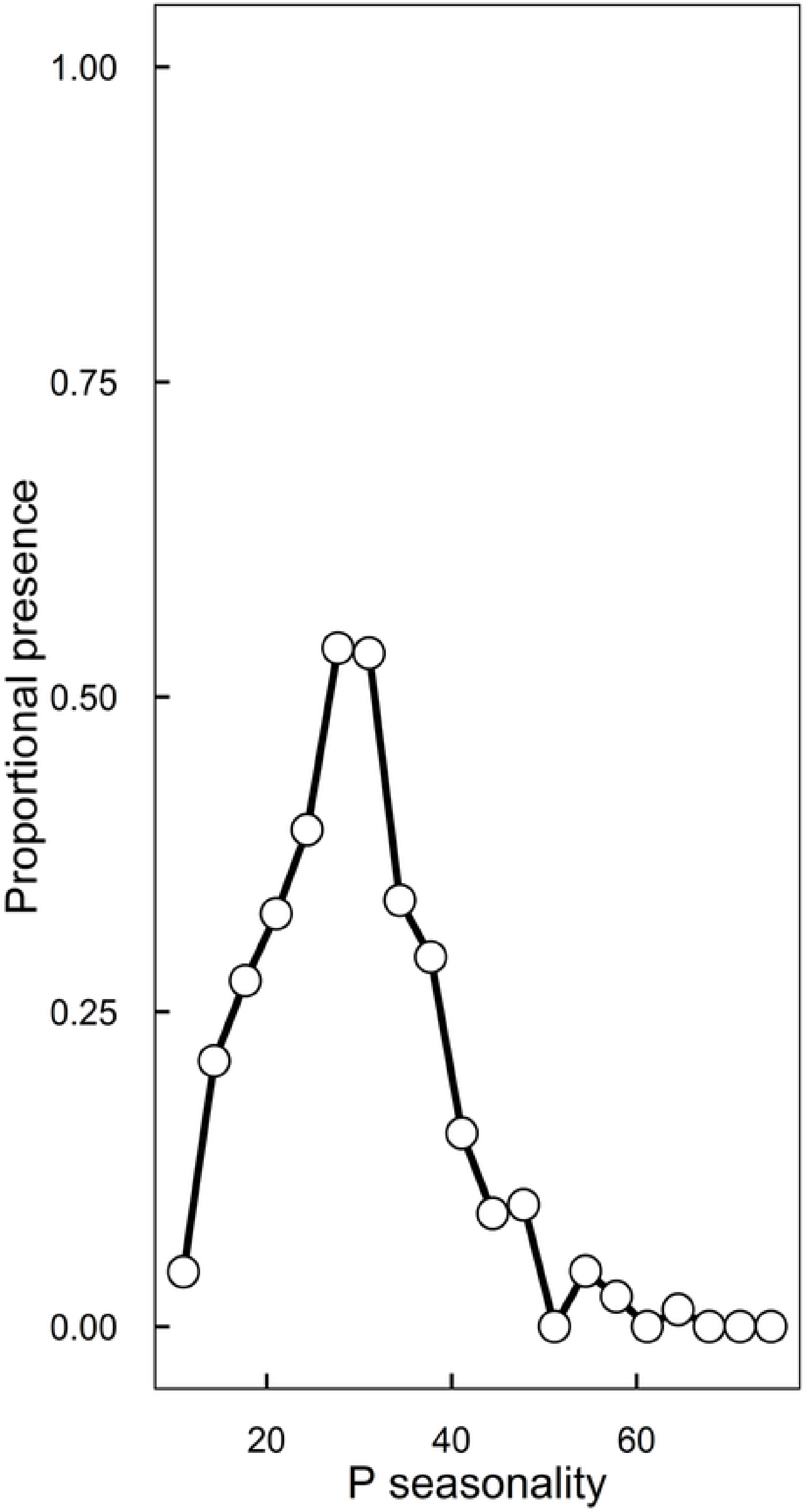

S5
Proportional presence of species along climate gradients

Proportional presence Iiwi bio7

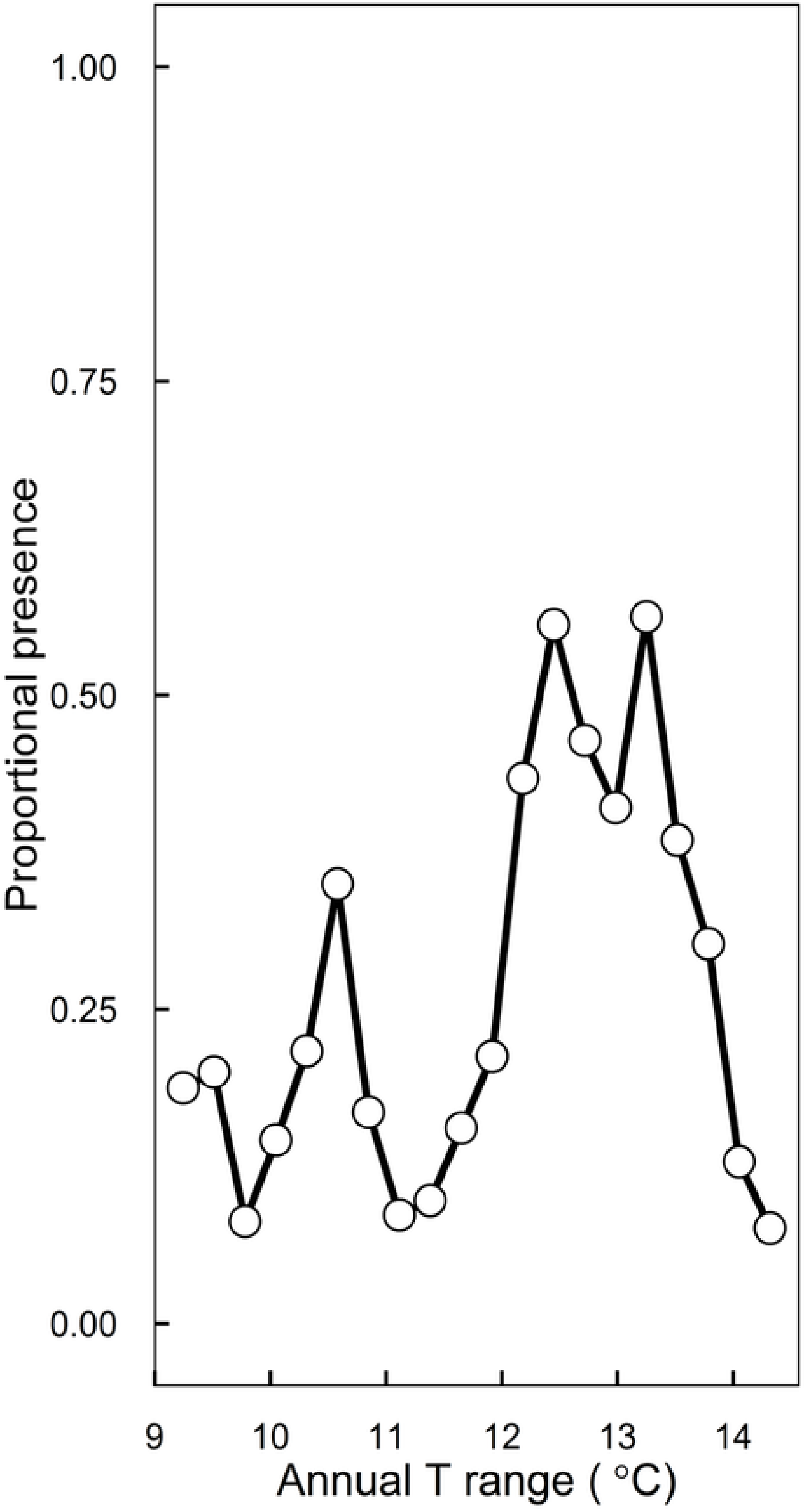

S5
Proportional presence of species along climate gradients

Proportional presence Kauai Amakihi bio1

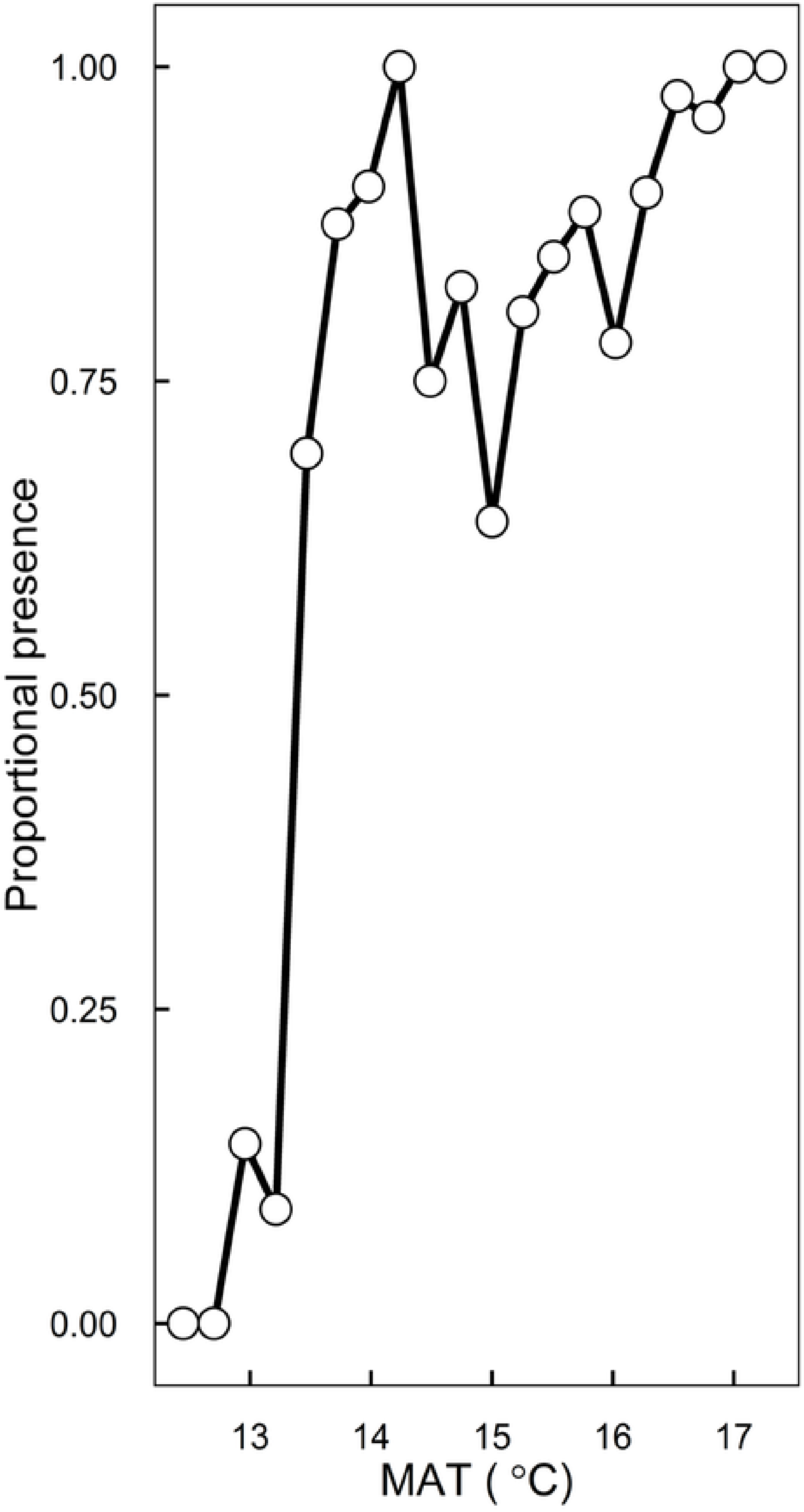

S5
Proportional presence of species along climate gradients

Proportional presence Kauai Amakihi bio12

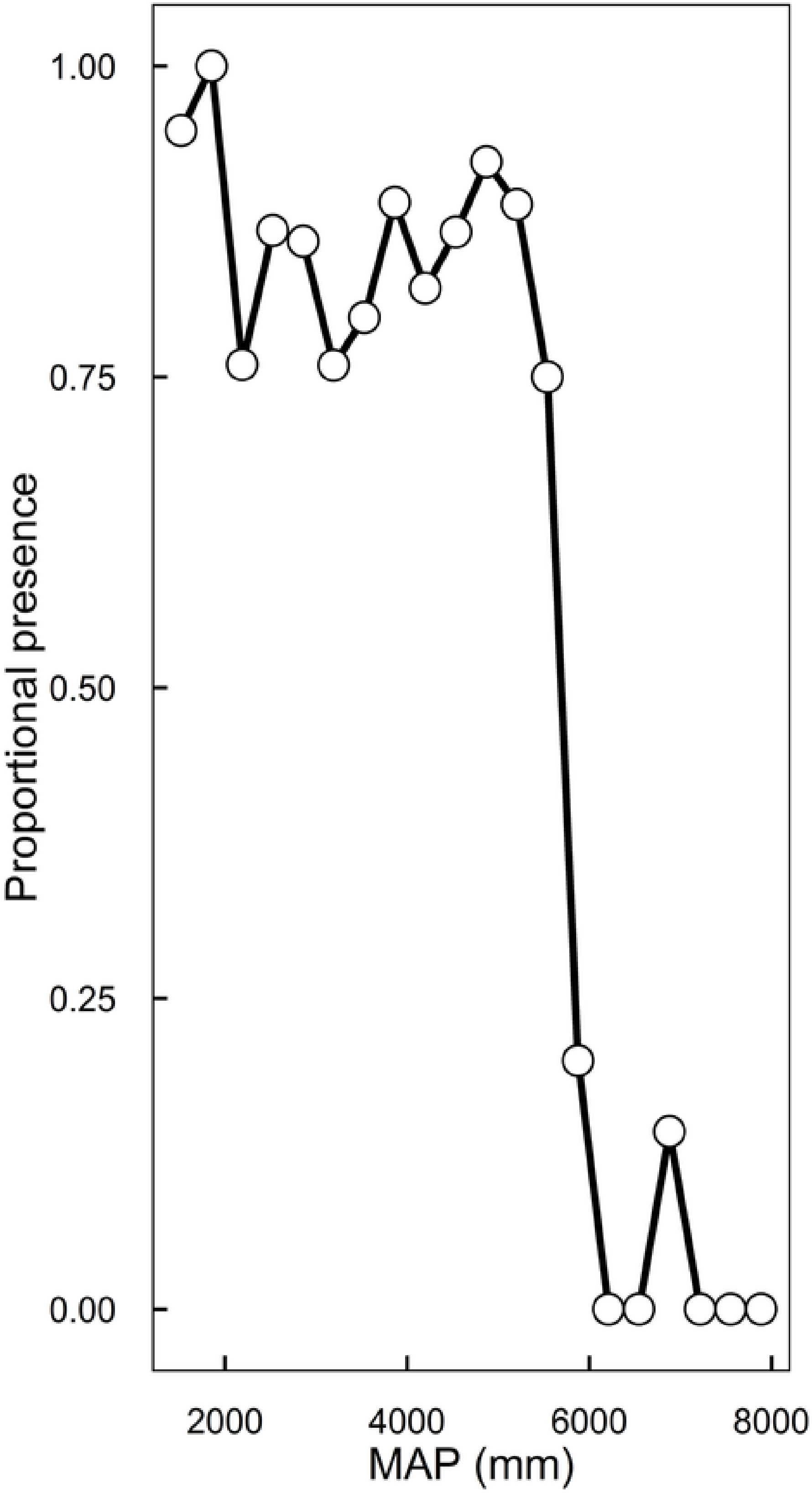

S5
Proportional presence of species along climate gradients

Proportional presence Kauai Amakihi bio15

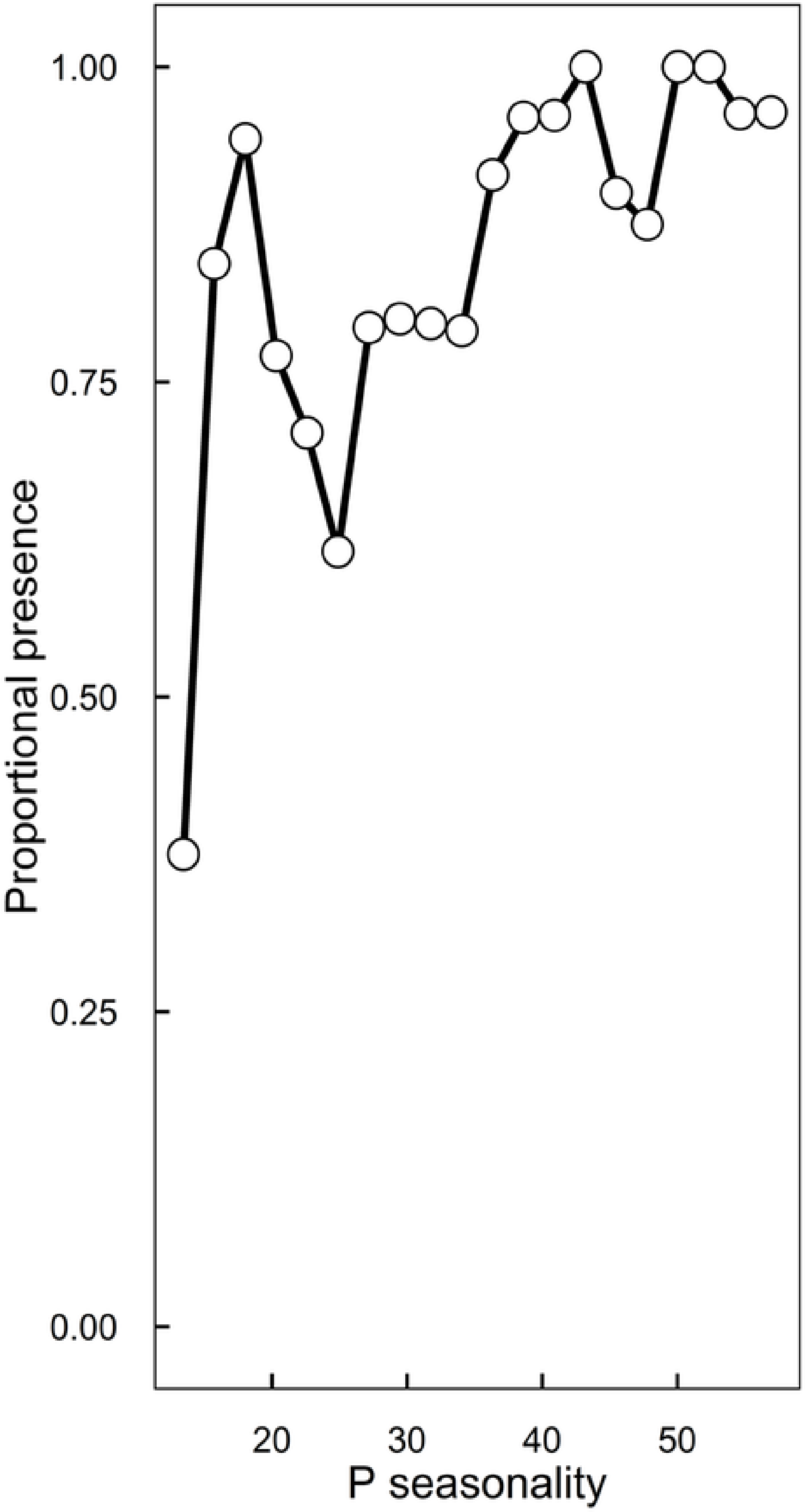

S5
Proportional presence of species along climate gradients

Proportional presence Kauai Amakihi bio7

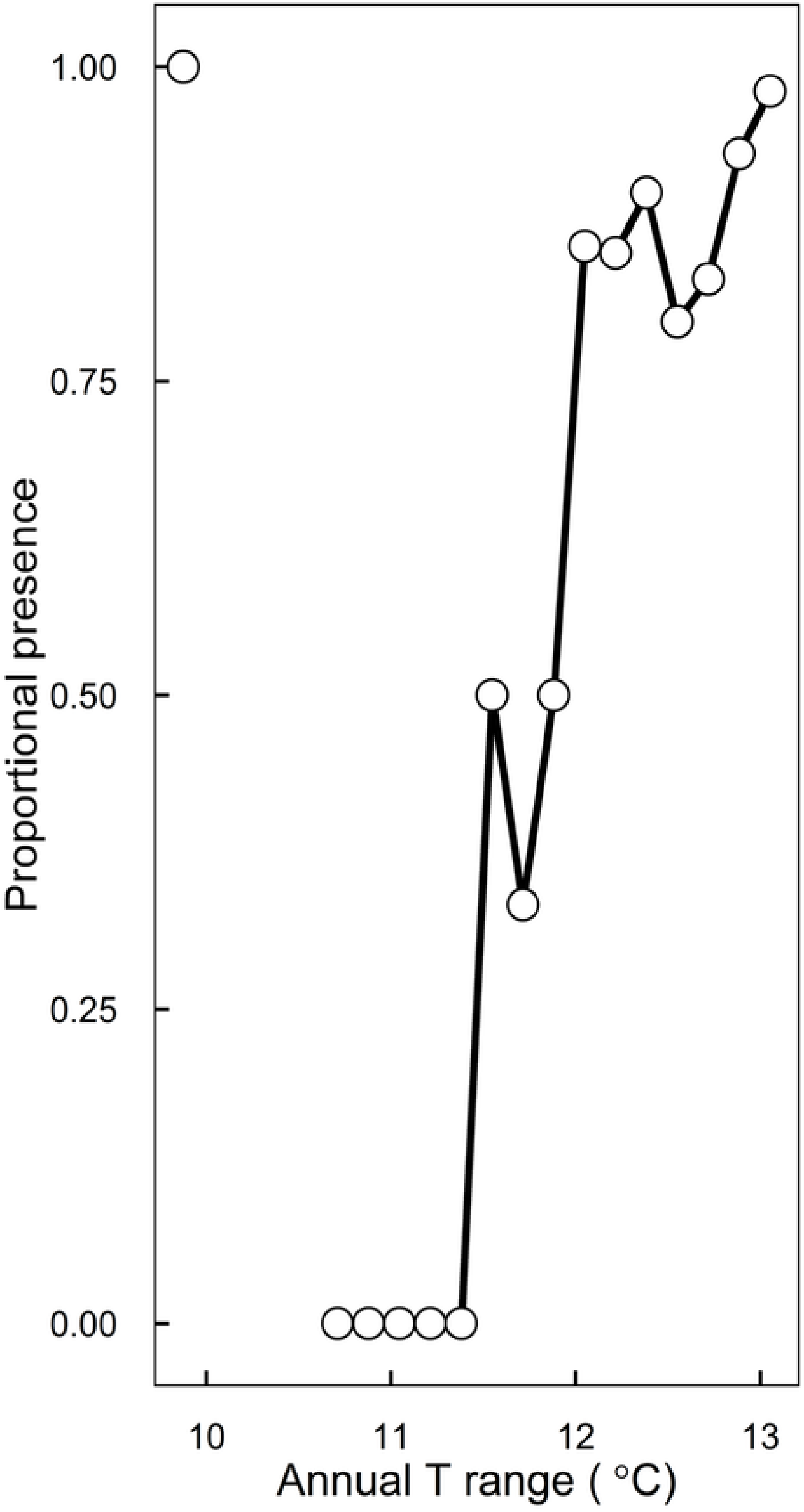

S5

Proportional presence of species along climate gradients

Proportional presence Kauai Elepaio bio1

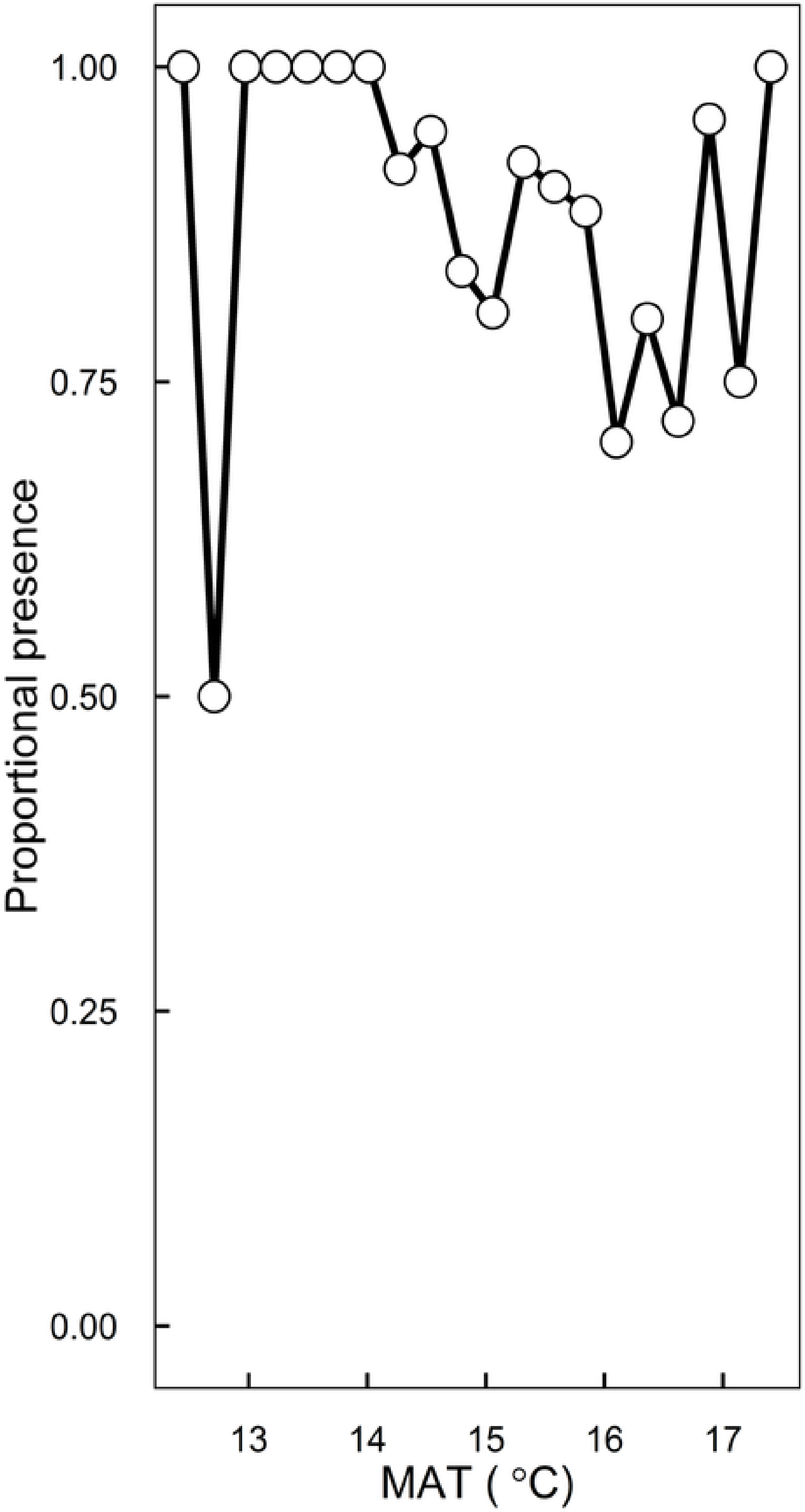

S5
Proportional presence of species along climate gradients

Proportional presence Kauai Elepaio bio12

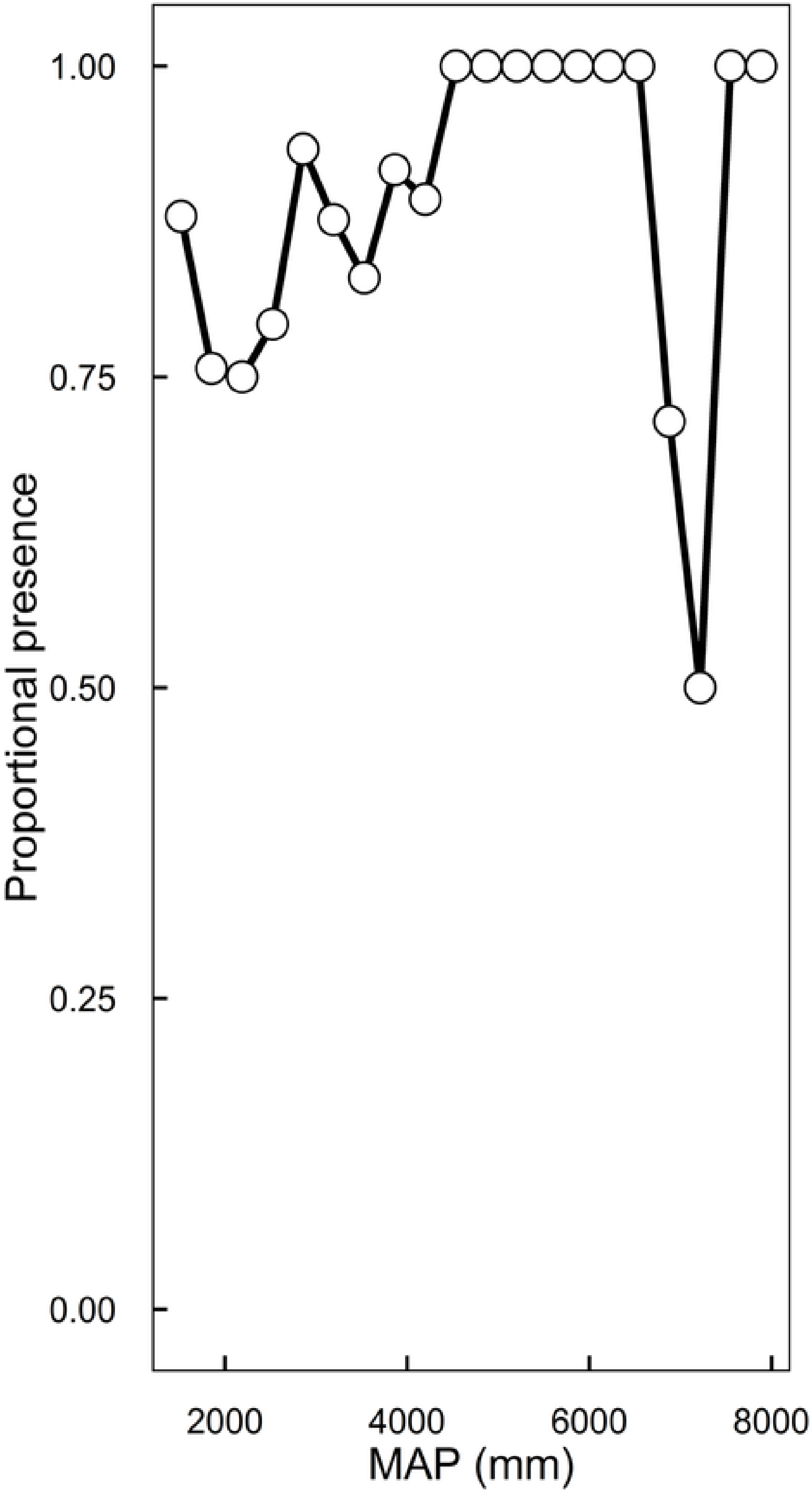

S5
Proportional presence of species along climate gradients

Proportional presence Kauai Elepaio bio15

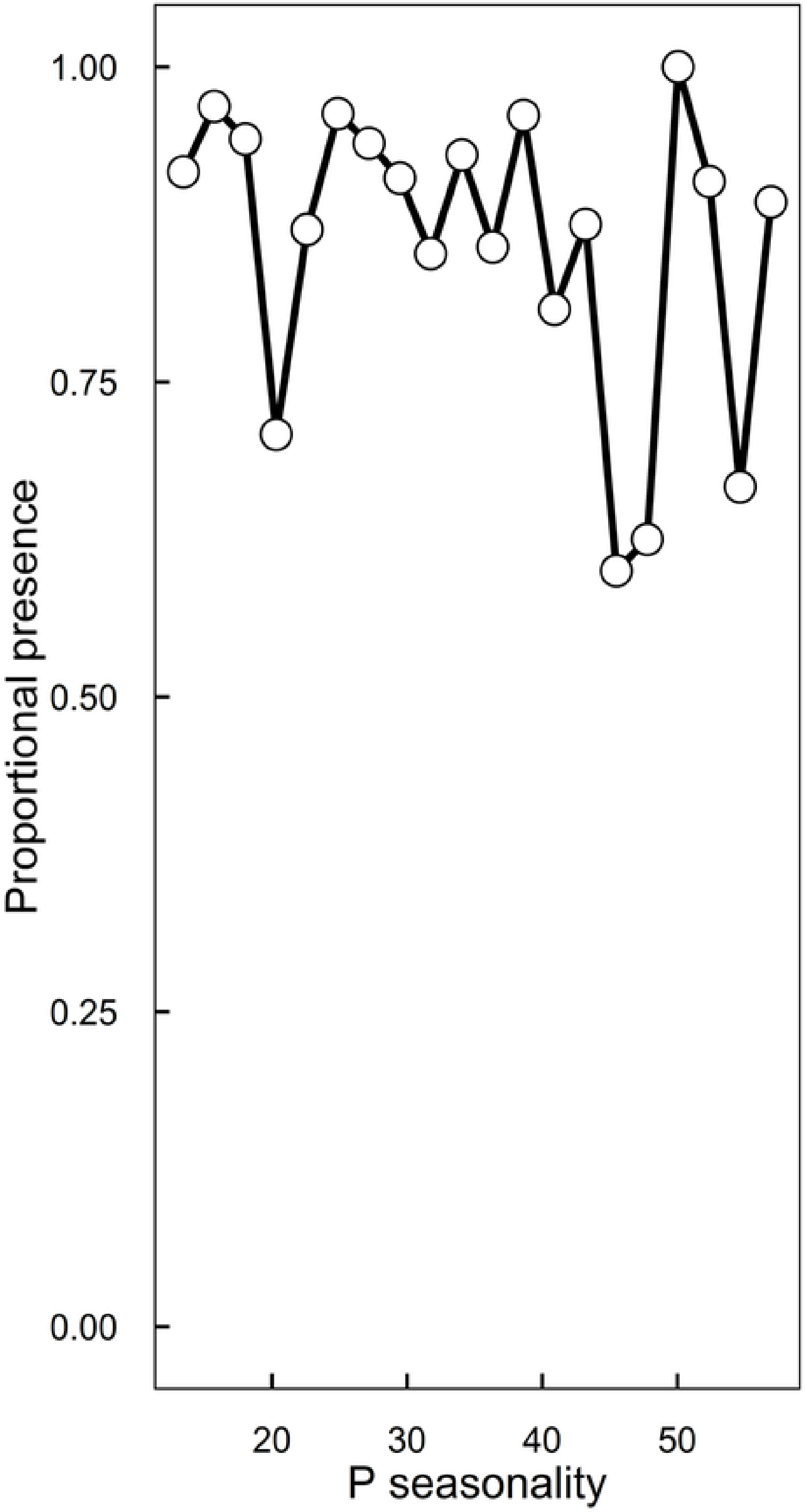

S5
Proportional presence of species along climate gradients

Proportional presence Kauai Elepaio bio7

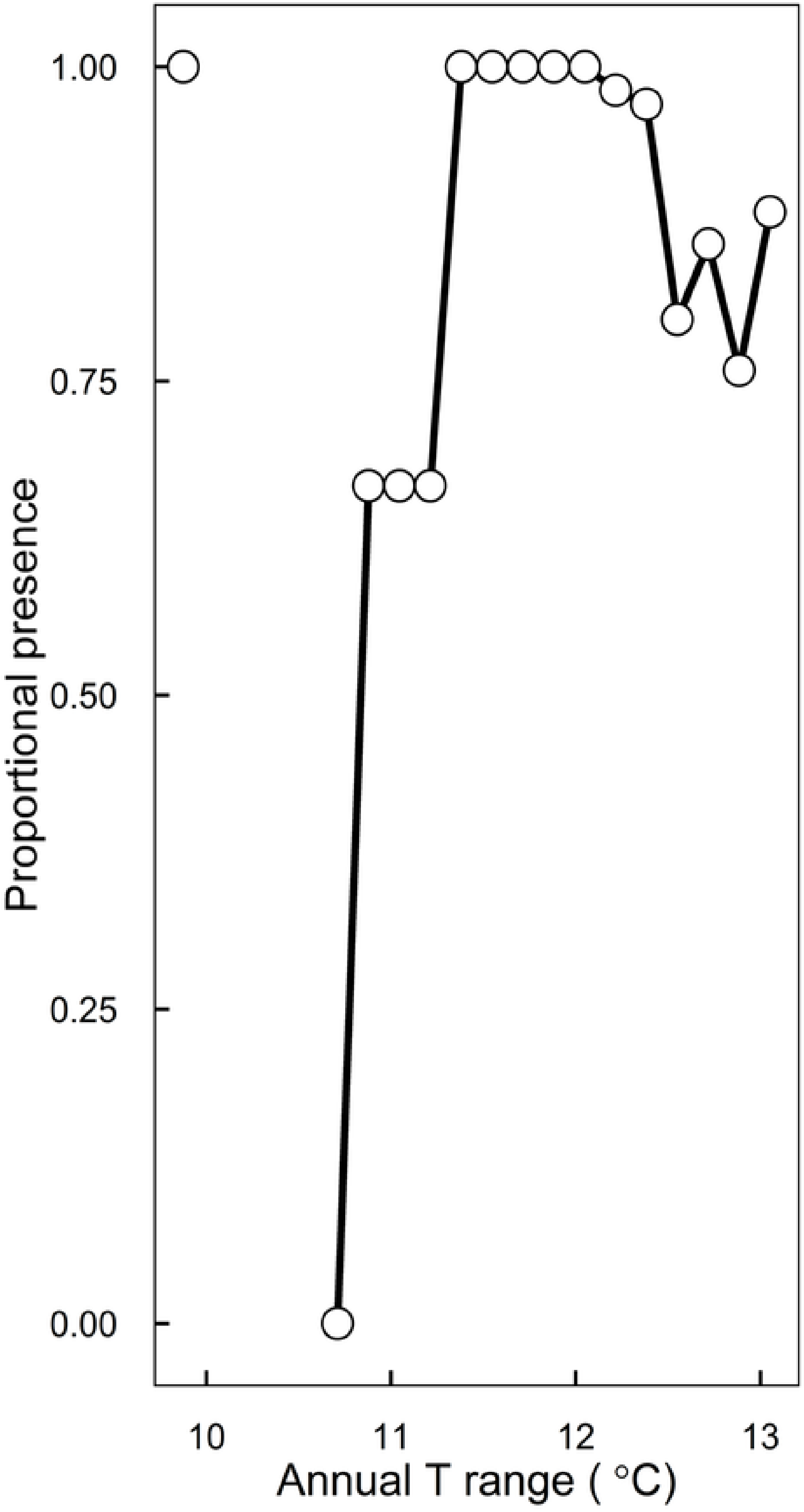

S5
Proportional presence of species along climate gradients

Proportional presence Maui Alauahio bio1

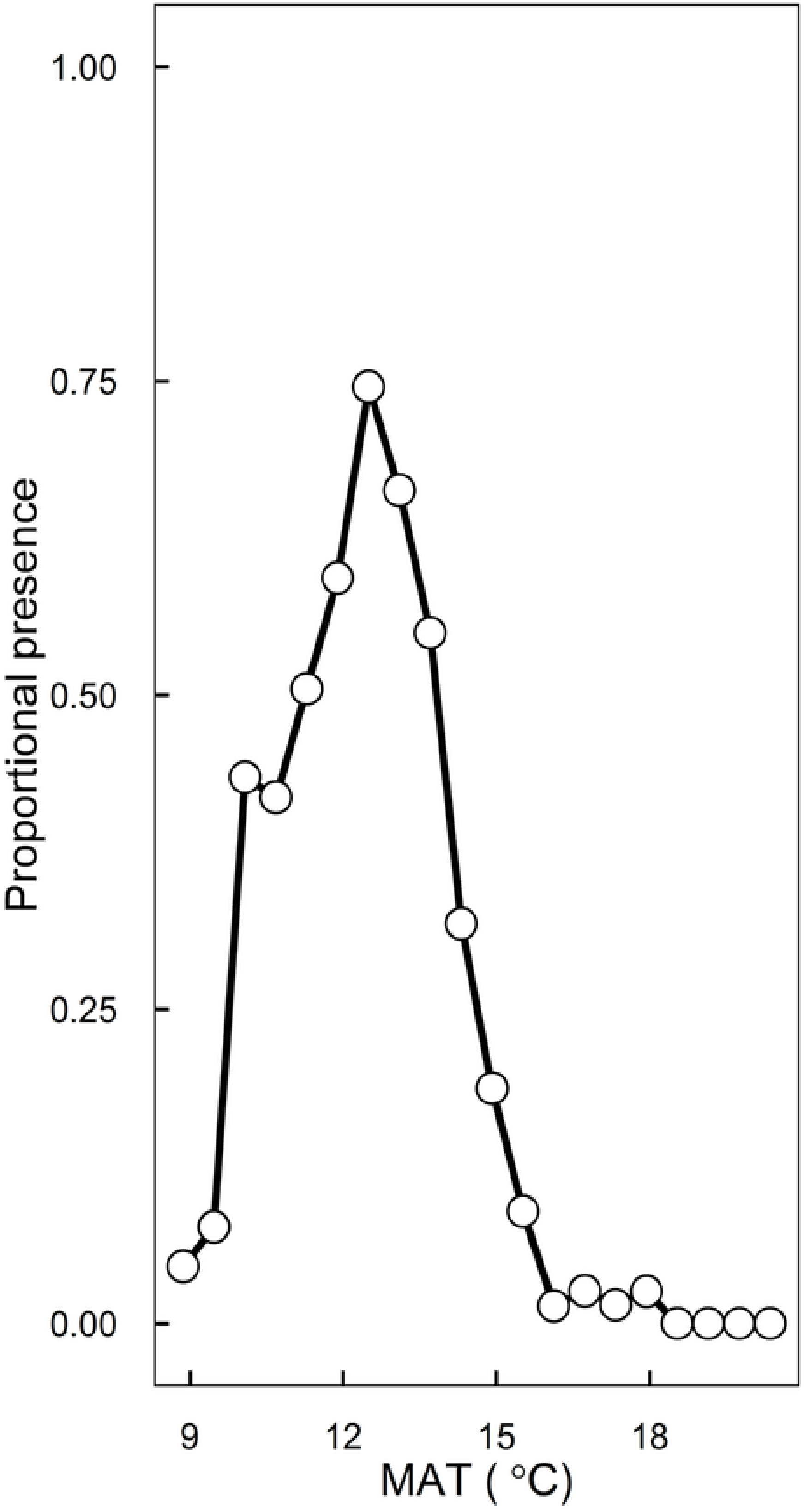

S5
Proportional presence of species along climate gradients

Proportional presence Maui Alauahio bio12

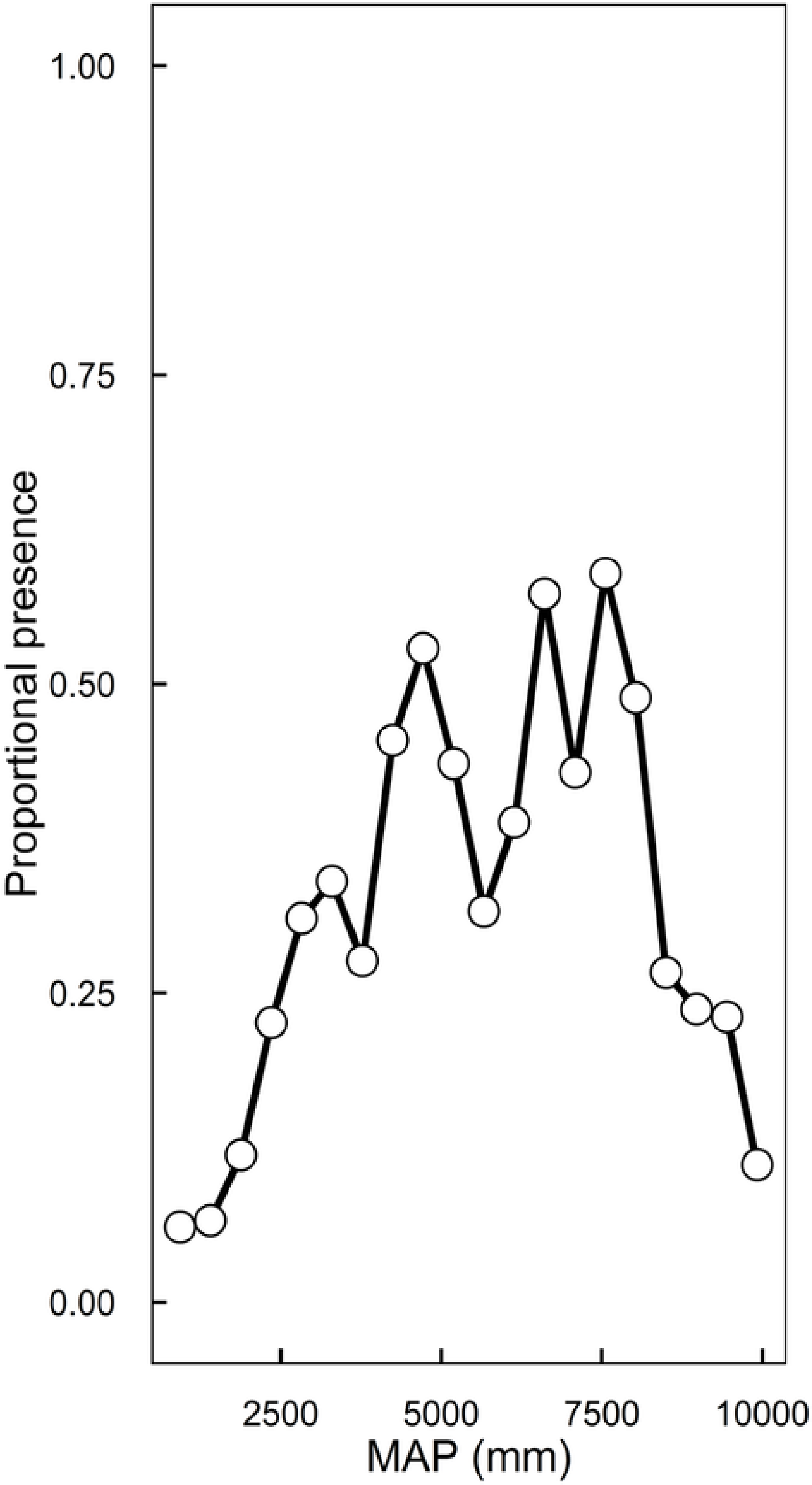

S5
Proportional presence of species along climate gradients

Proportional presence Maui Alauahio bio15

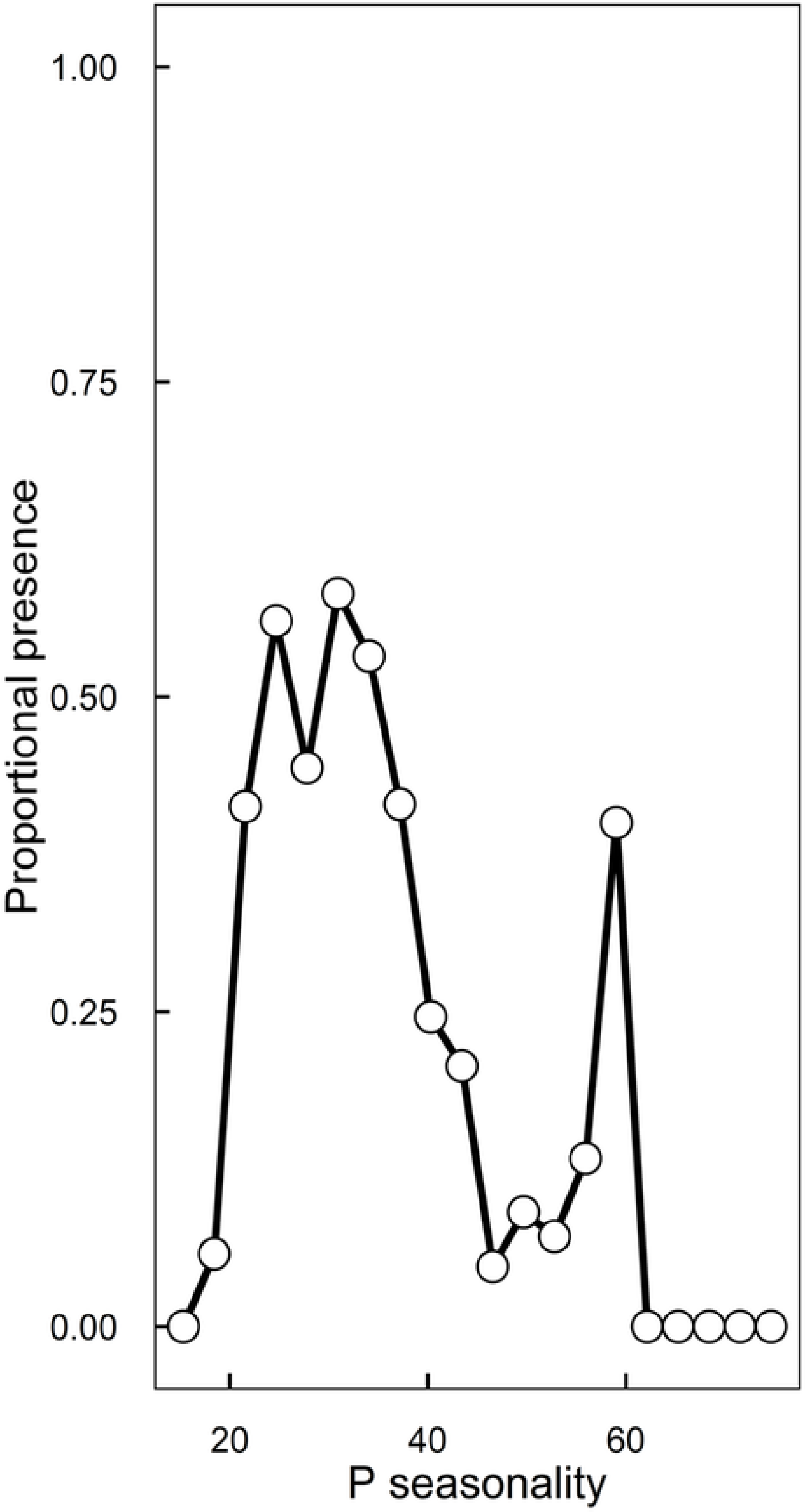

S5
Proportional presence of species along climate gradients

Proportional presence Maui Alauahio bio7

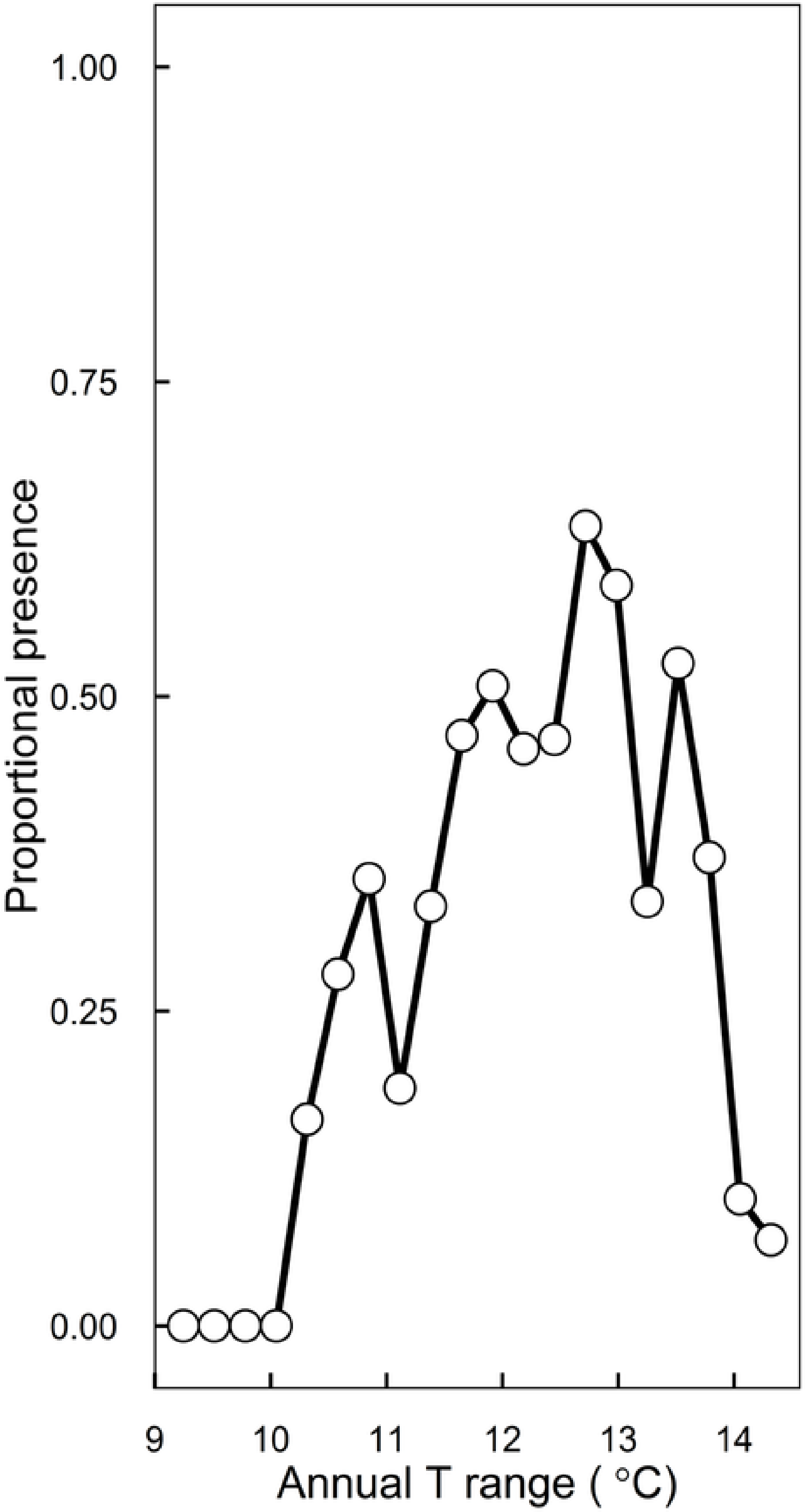

S5
Proportional presence of species along climate gradients

Proportional presence Maui Parrotbill bio1

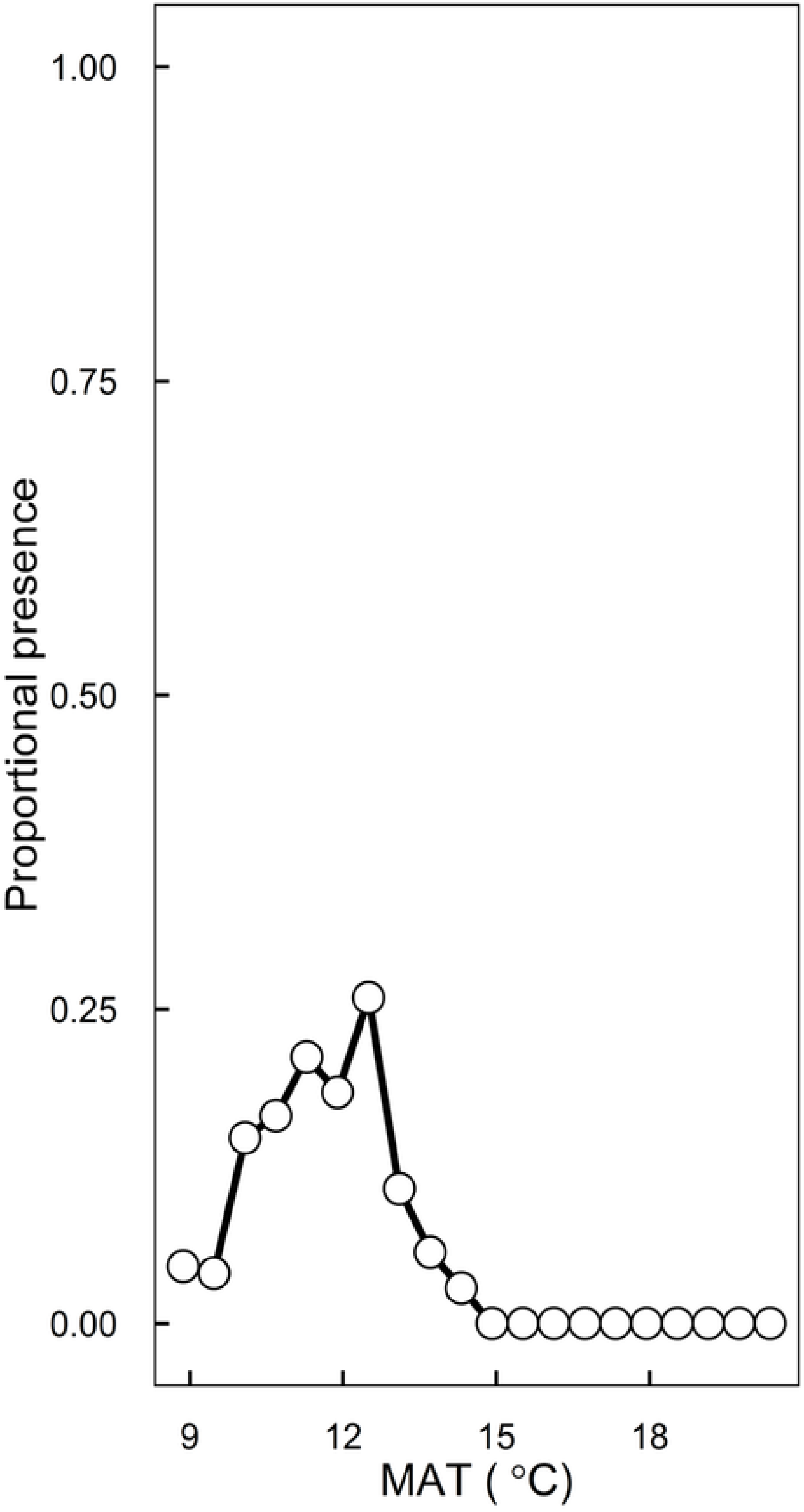

S5
Proportional presence of species along climate gradients

Proportional presence Maui Parrotbill bio12

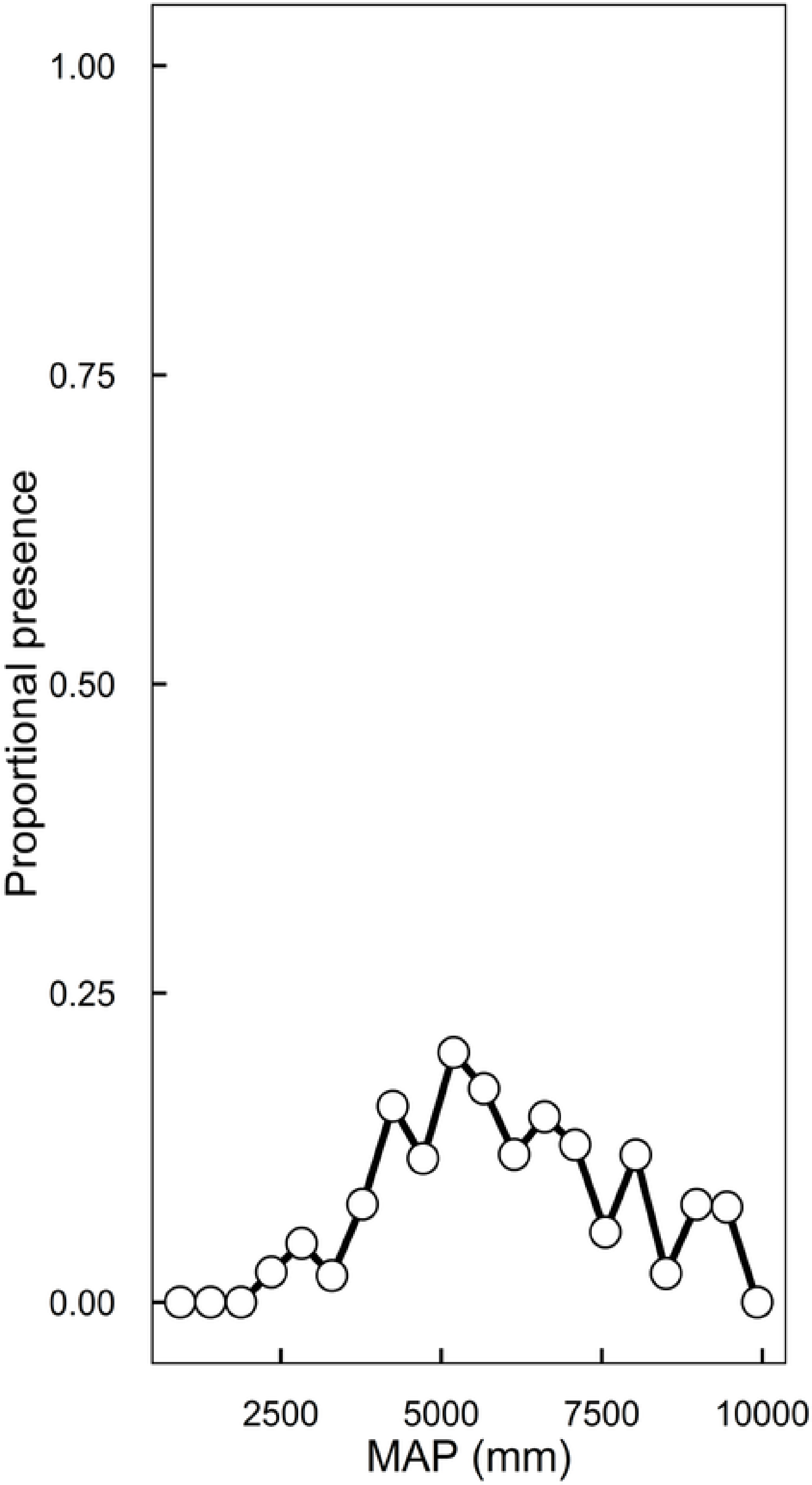

S5
Proportional presence of species along climate gradients

Proportional presence Maui Parrotbill bio15

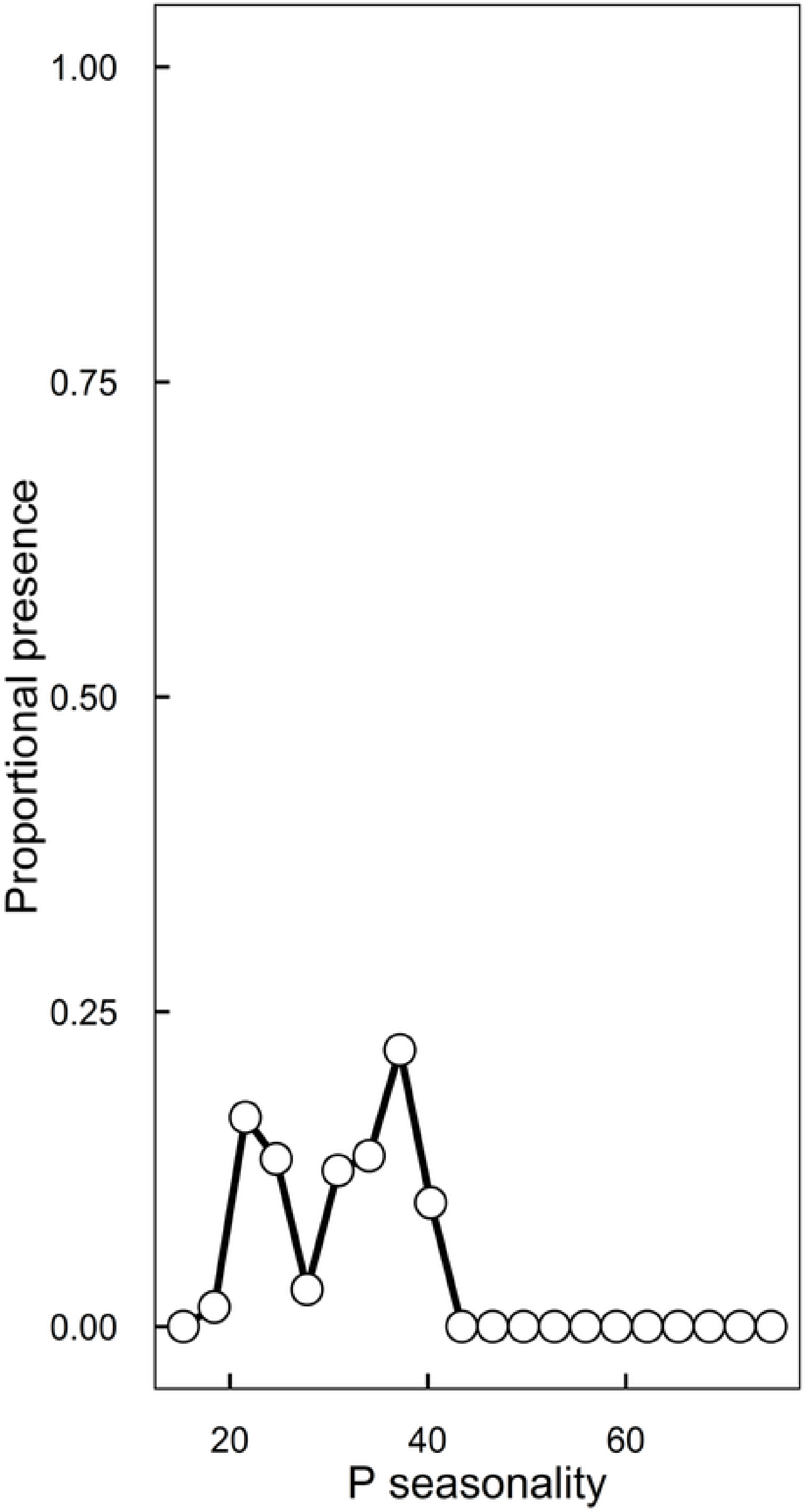

S5
Proportional presence of species along climate gradients

Proportional presence Maui Parrotbill bio7

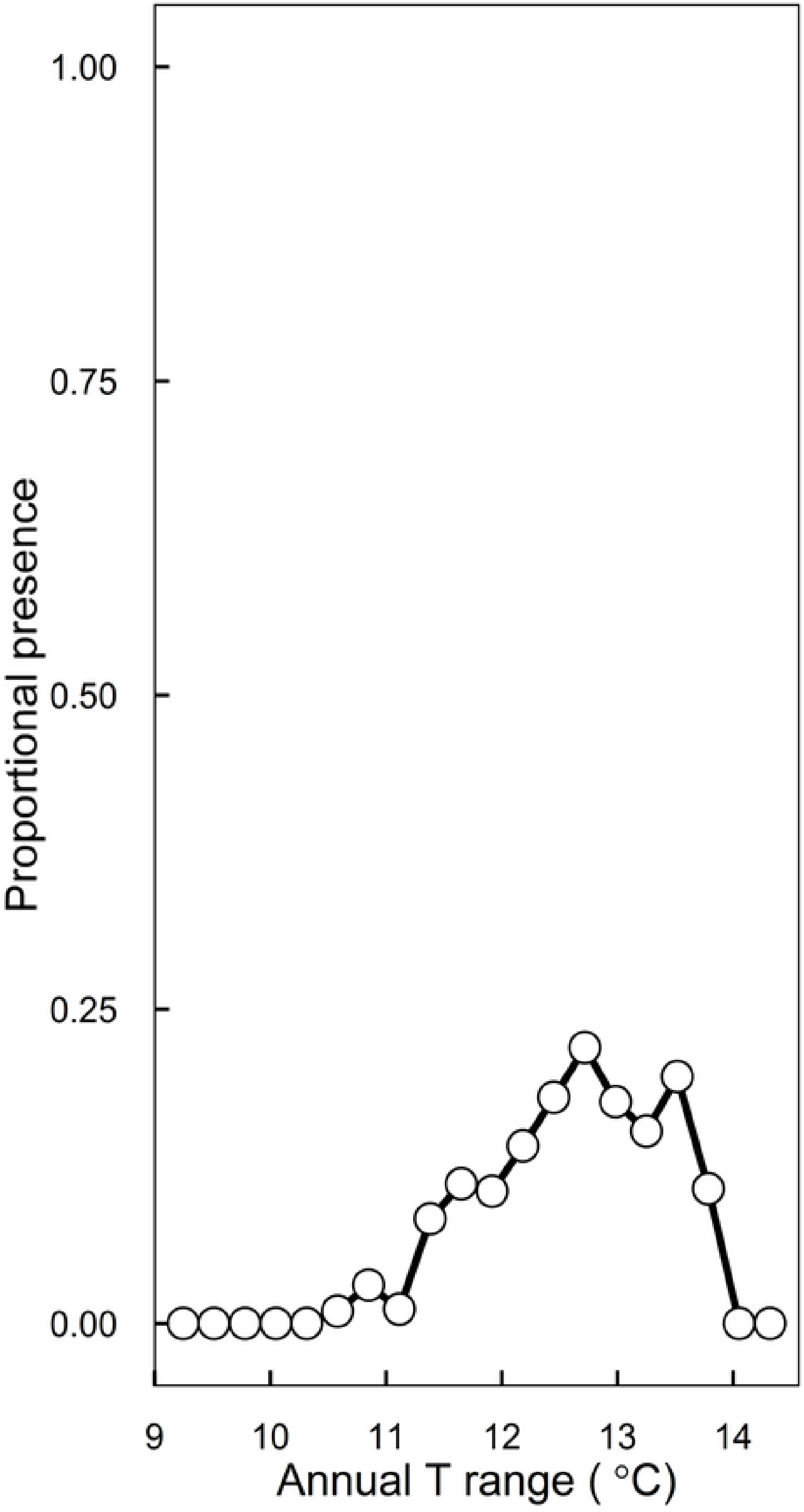

S5
Proportional presence of species along climate gradients

Proportional presence Oahu Amakihi bio1

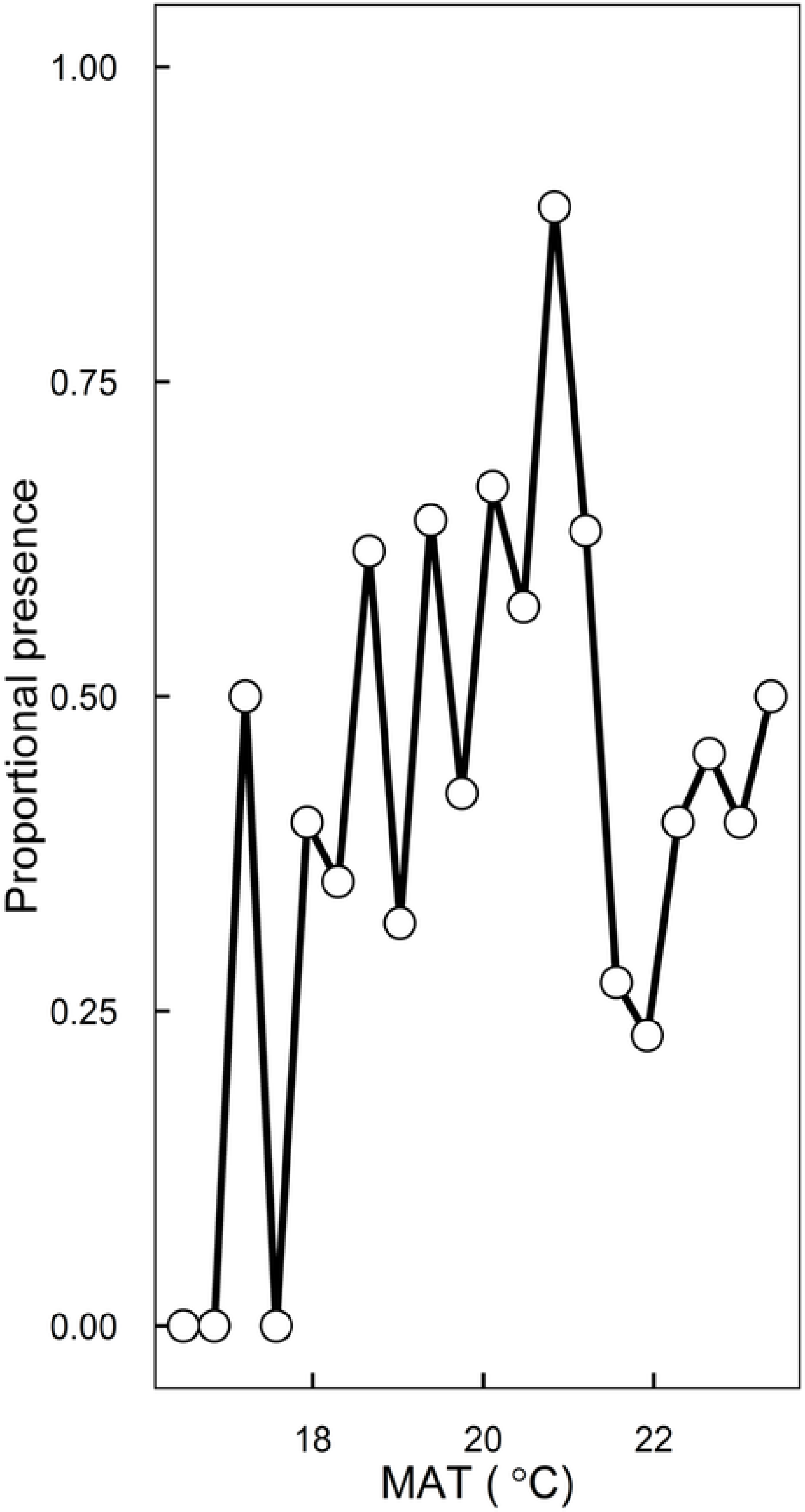

S5

Proportional presence of species along climate gradients

Proportional presence Oahu Amakihi bio12

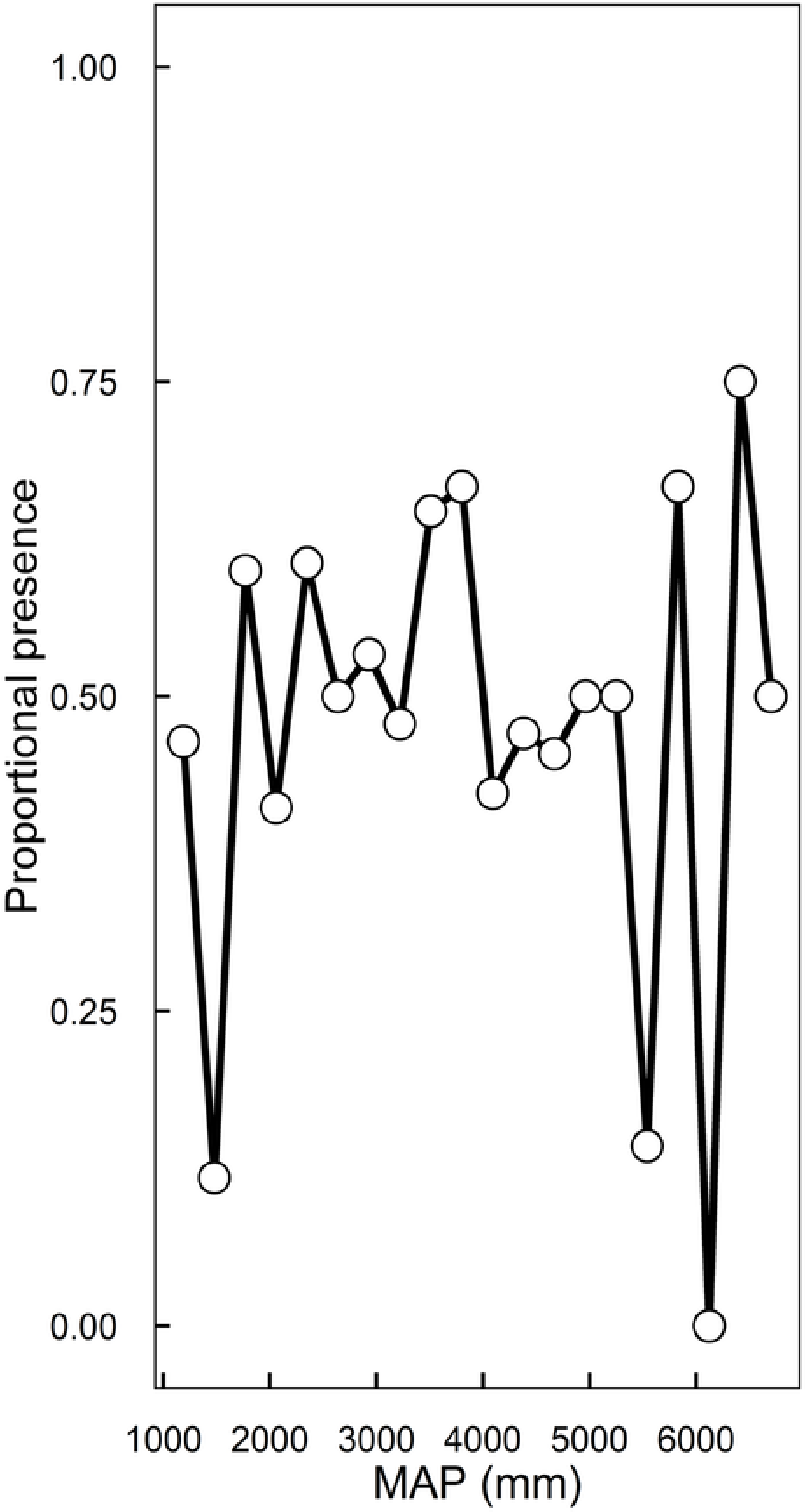

S5
Proportional presence of species along climate gradients

Proportional presence Oahu Amakihi bio15

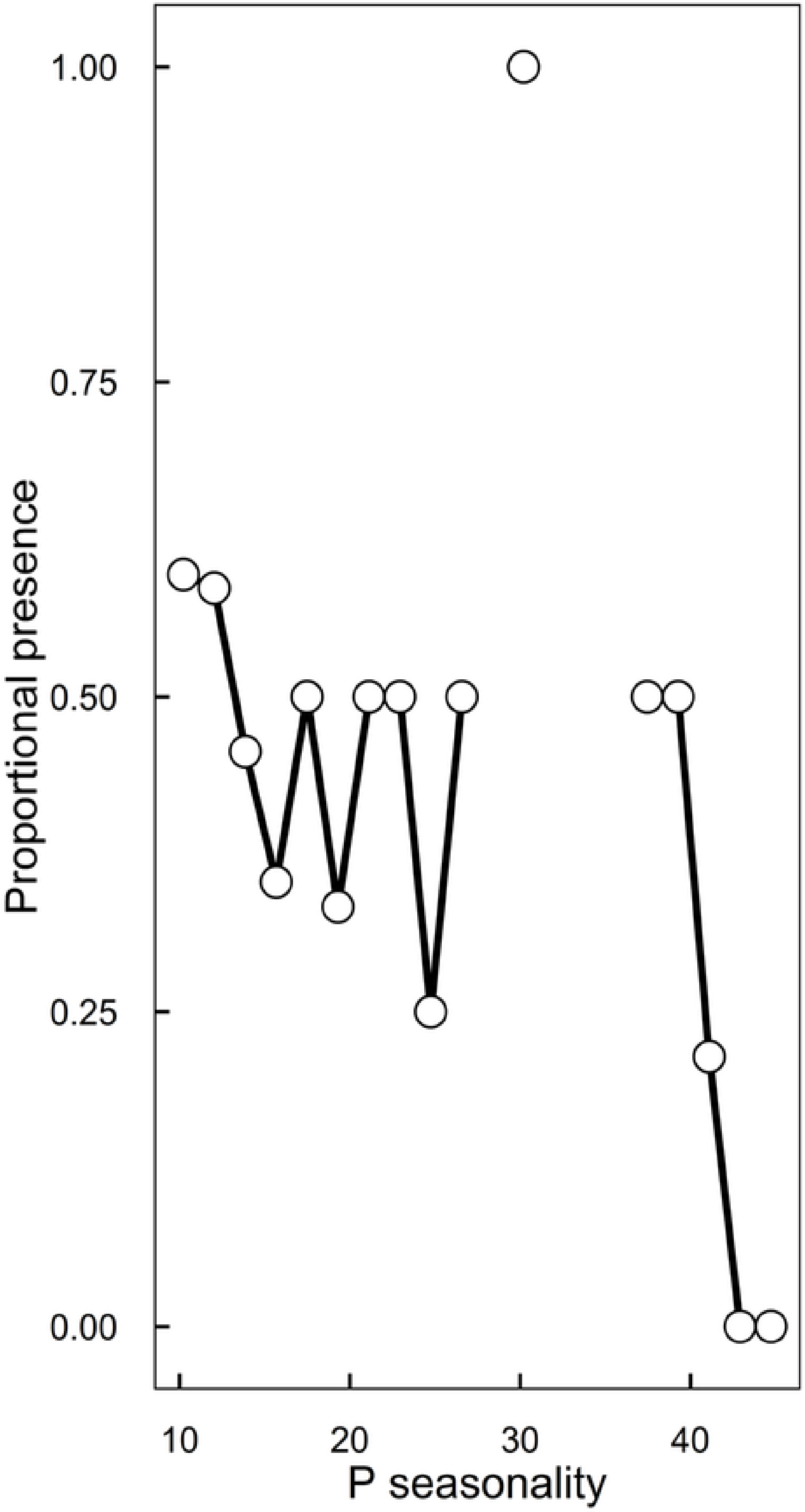

S5
Proportional presence of species along climate gradients

Proportional presence Oahu Amakihi bio7

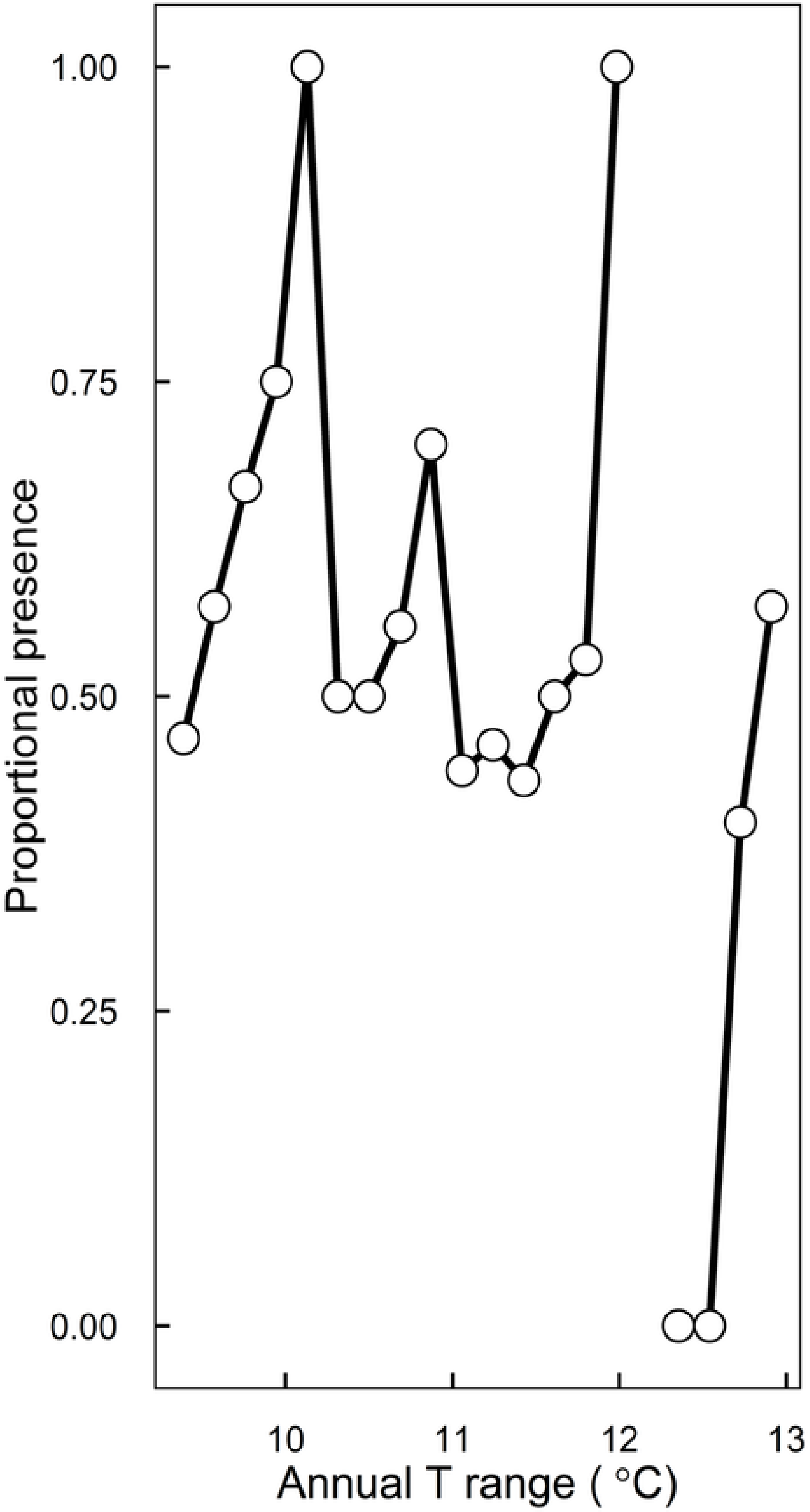

S5
Proportional presence of species along climate gradients

Proportional presence Oahu Elepaio bio1

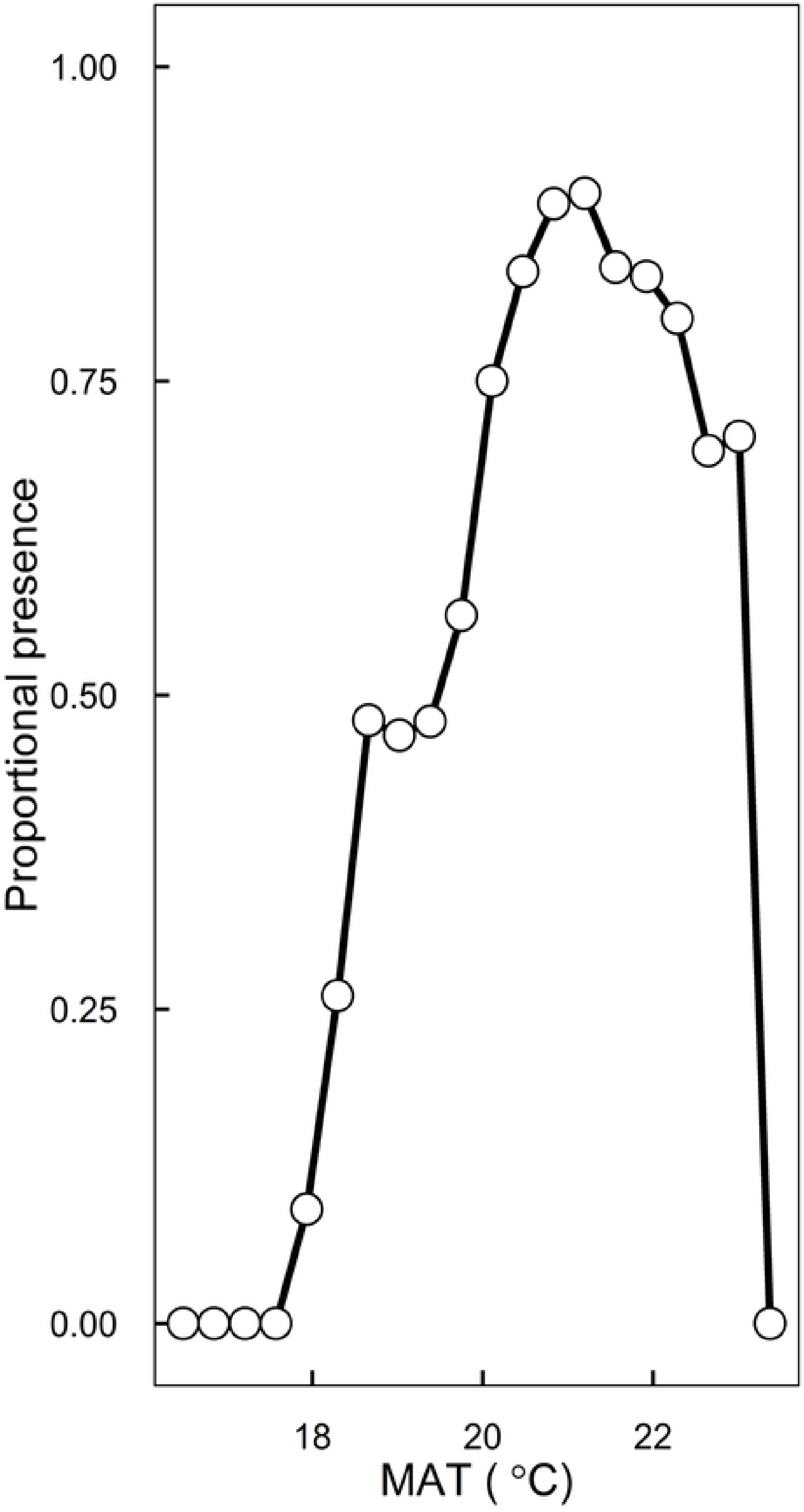

S5
Proportional presence of species along climate gradients

Proportional presence Oahu Elepaio bio12

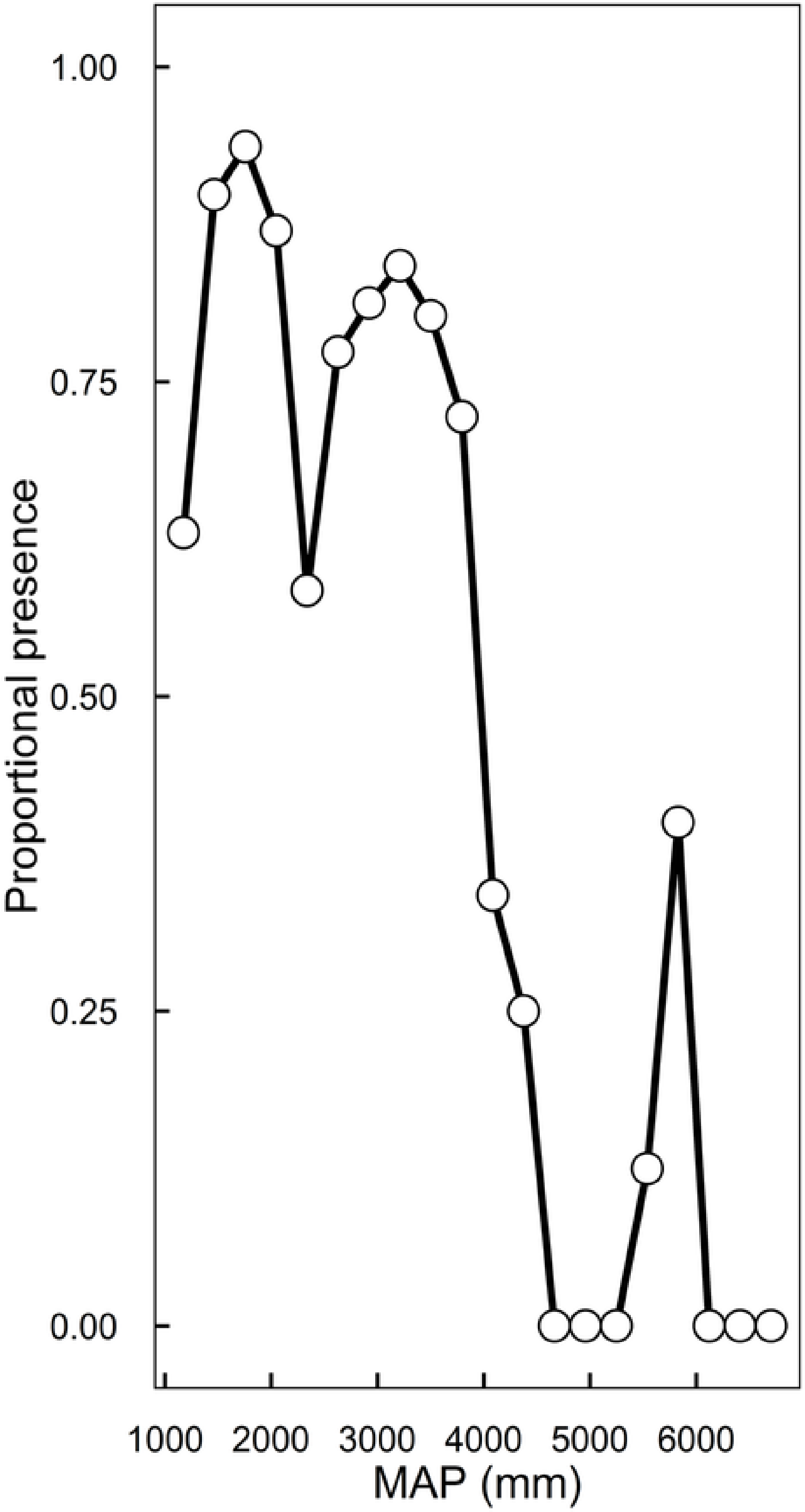

S5
Proportional presence of species along climate gradients

Proportional presence Oahu Elepaio bio15

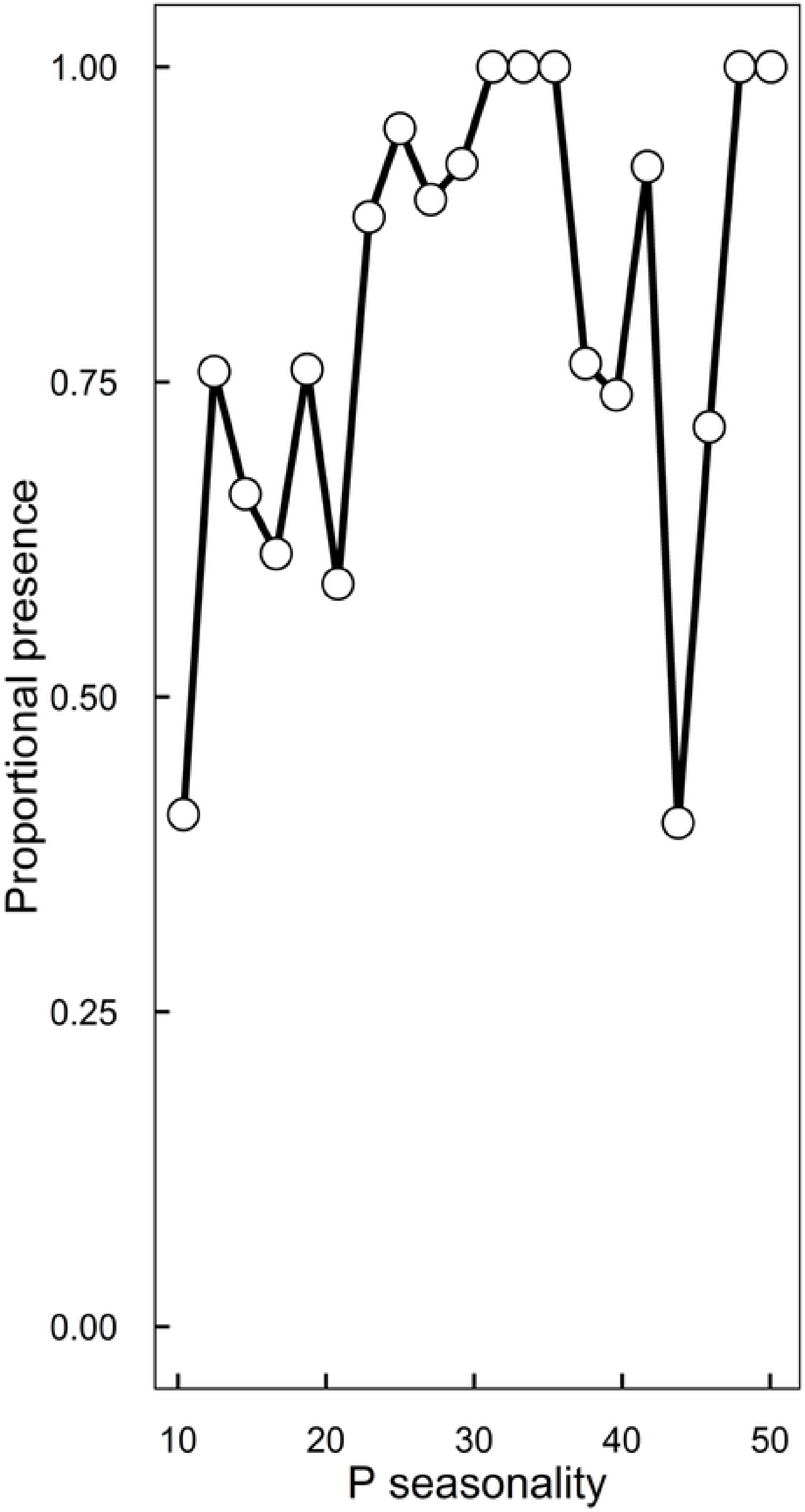

S5
Proportional presence of species along climate gradients

Proportional presence Oahu Elepaio bio7

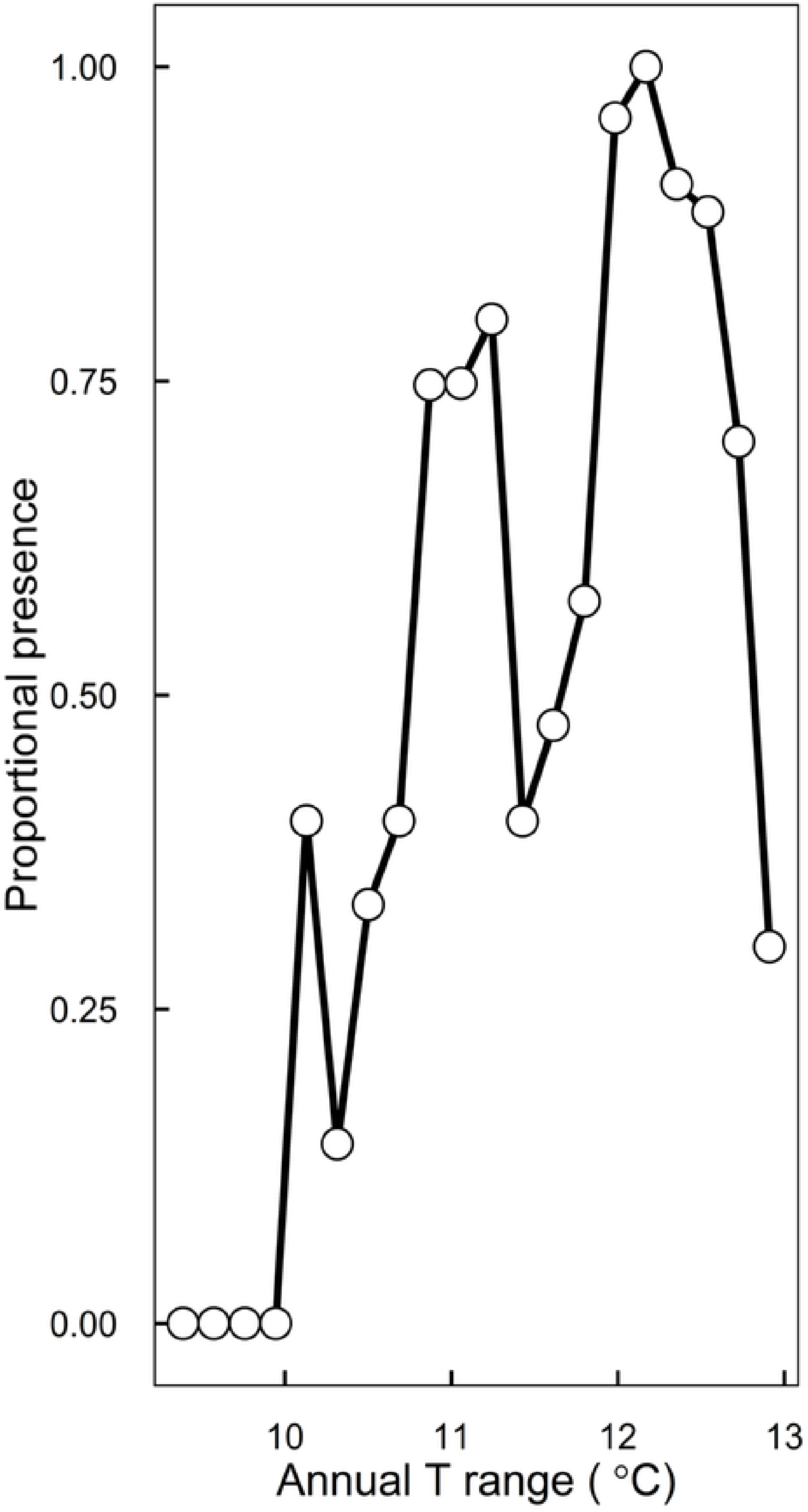

S5
Proportional presence of species along climate gradients

Proportional presence Omao bio1

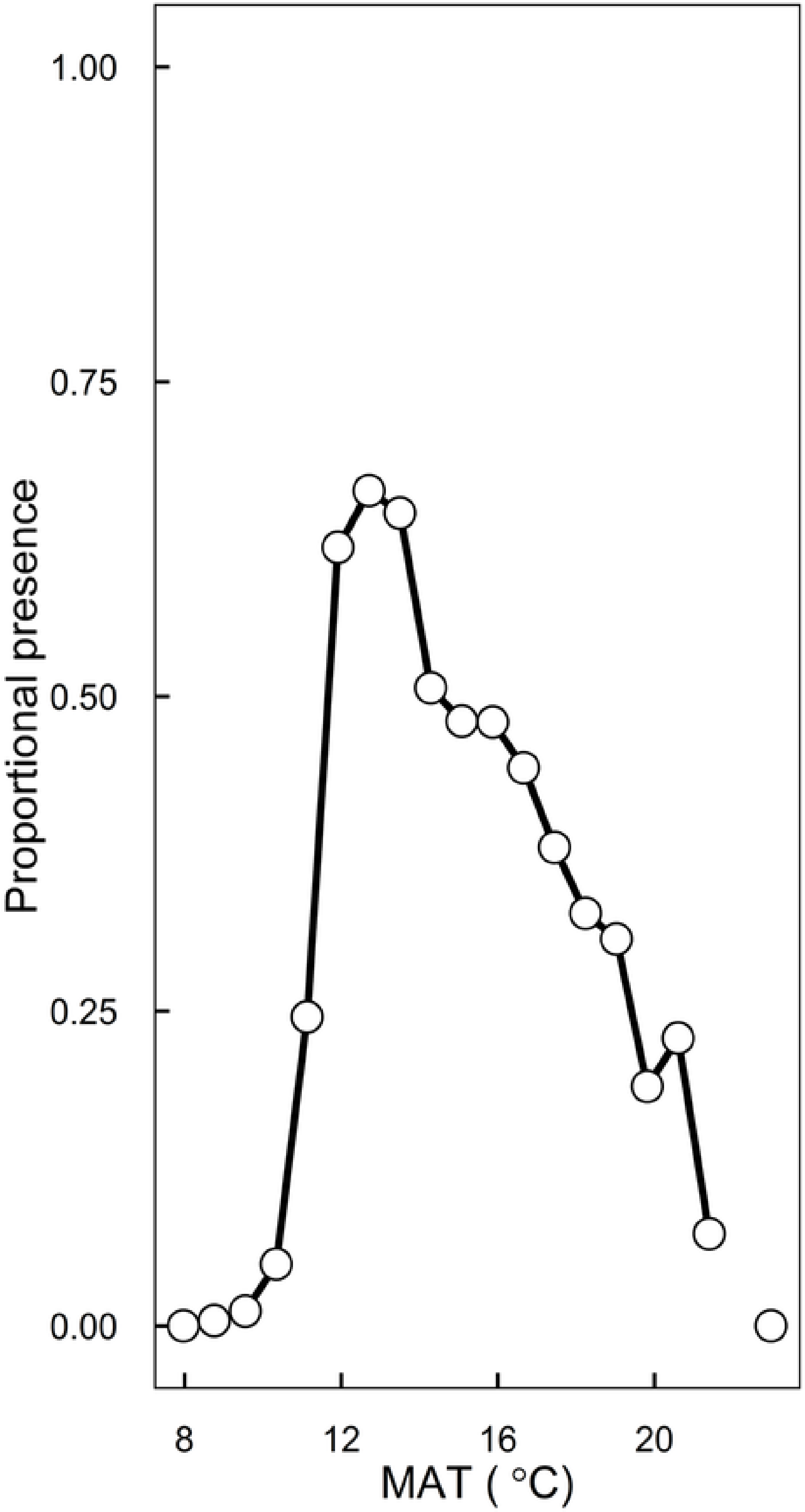

S5
Proportional presence of species along climate gradients

Proportional presence Omao bio12

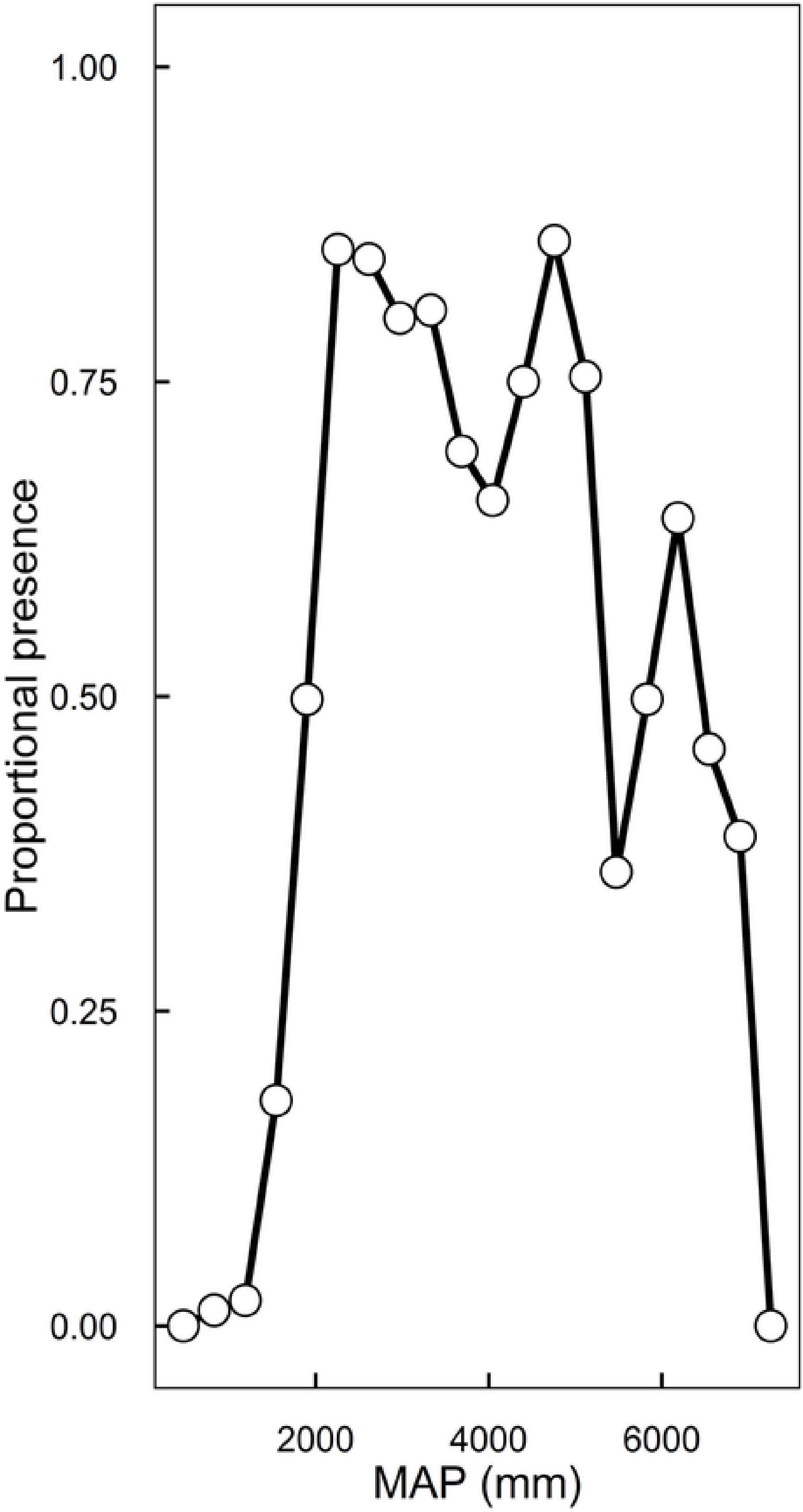

S5
Proportional presence of species along climate gradients

Proportional presence Omao bio15

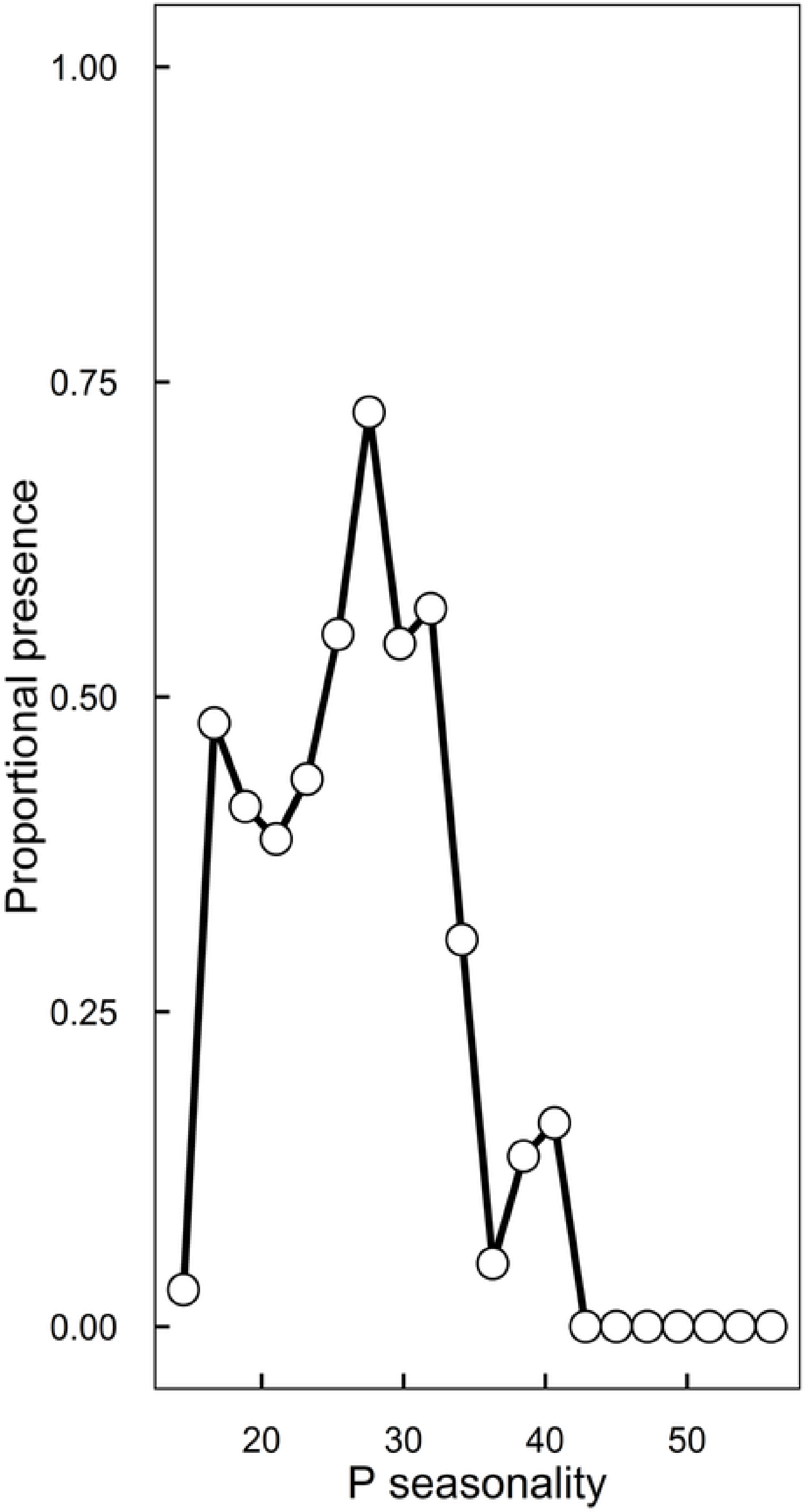

S5
Proportional presence of species along climate gradients

Proportional presence Omao bio7

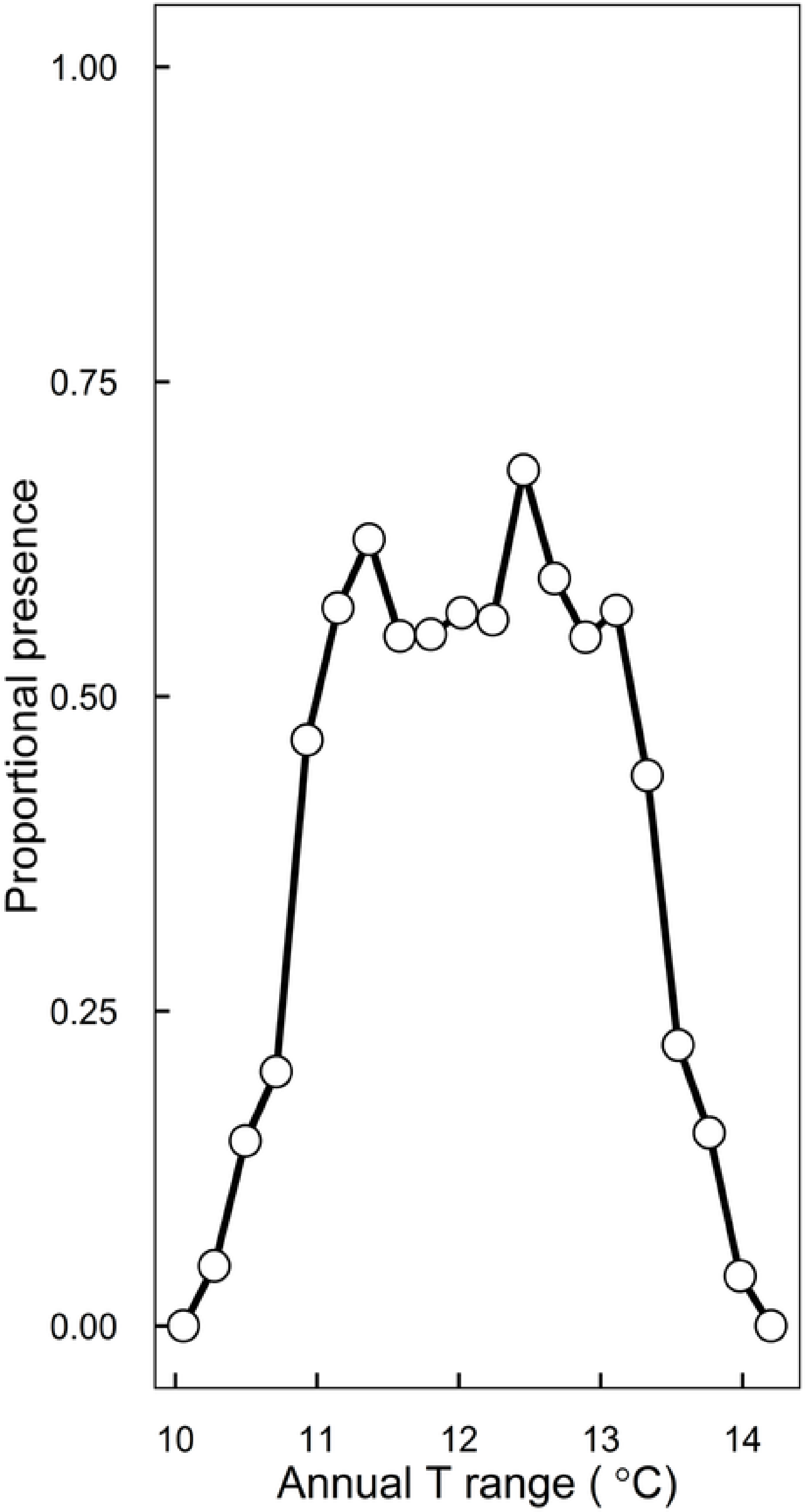

S5
Proportional presence of species along climate gradients

Proportional presence Palila bio1

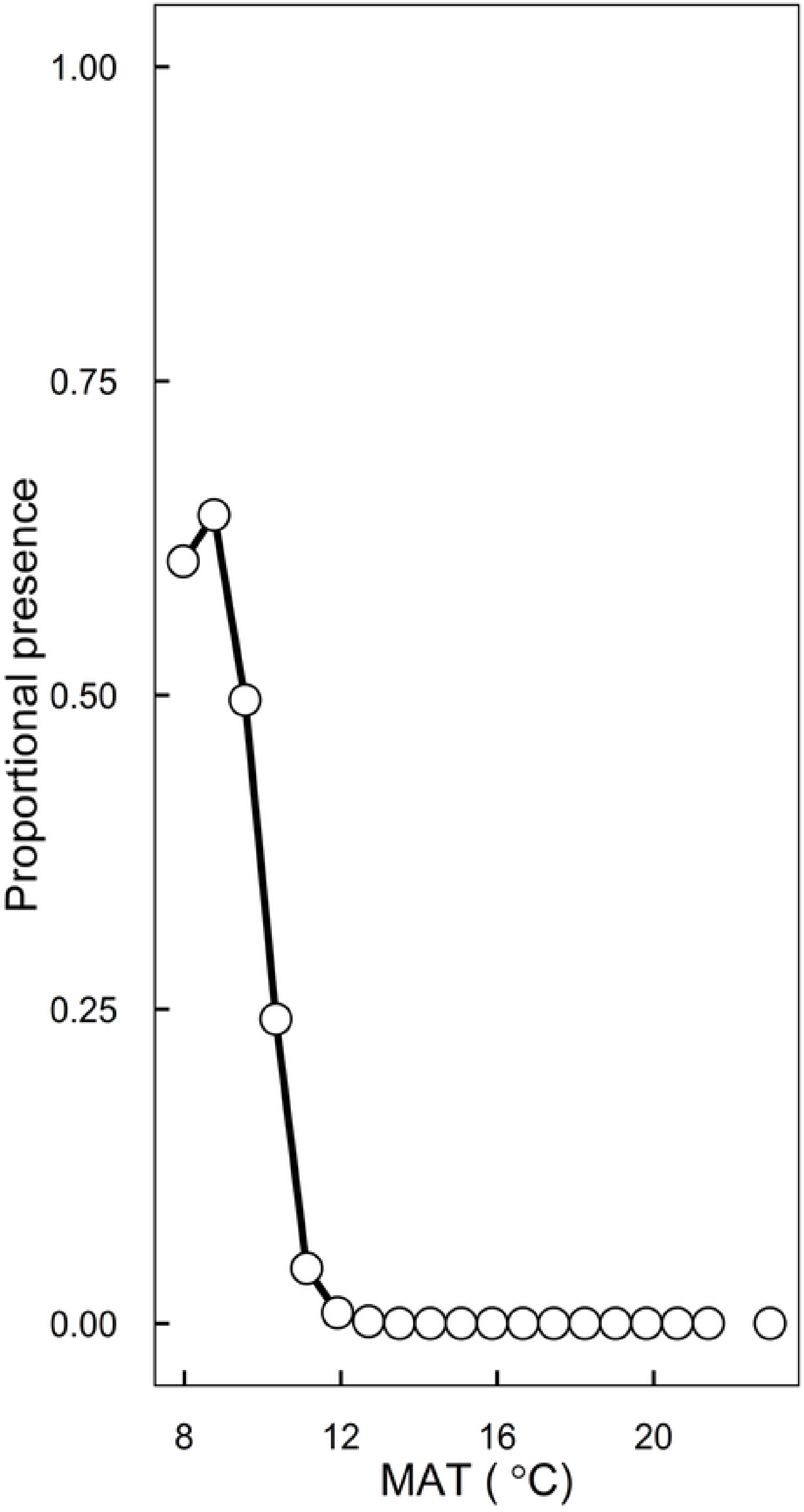

S5
Proportional presence of species along climate gradients

Proportional presence Palila bio12

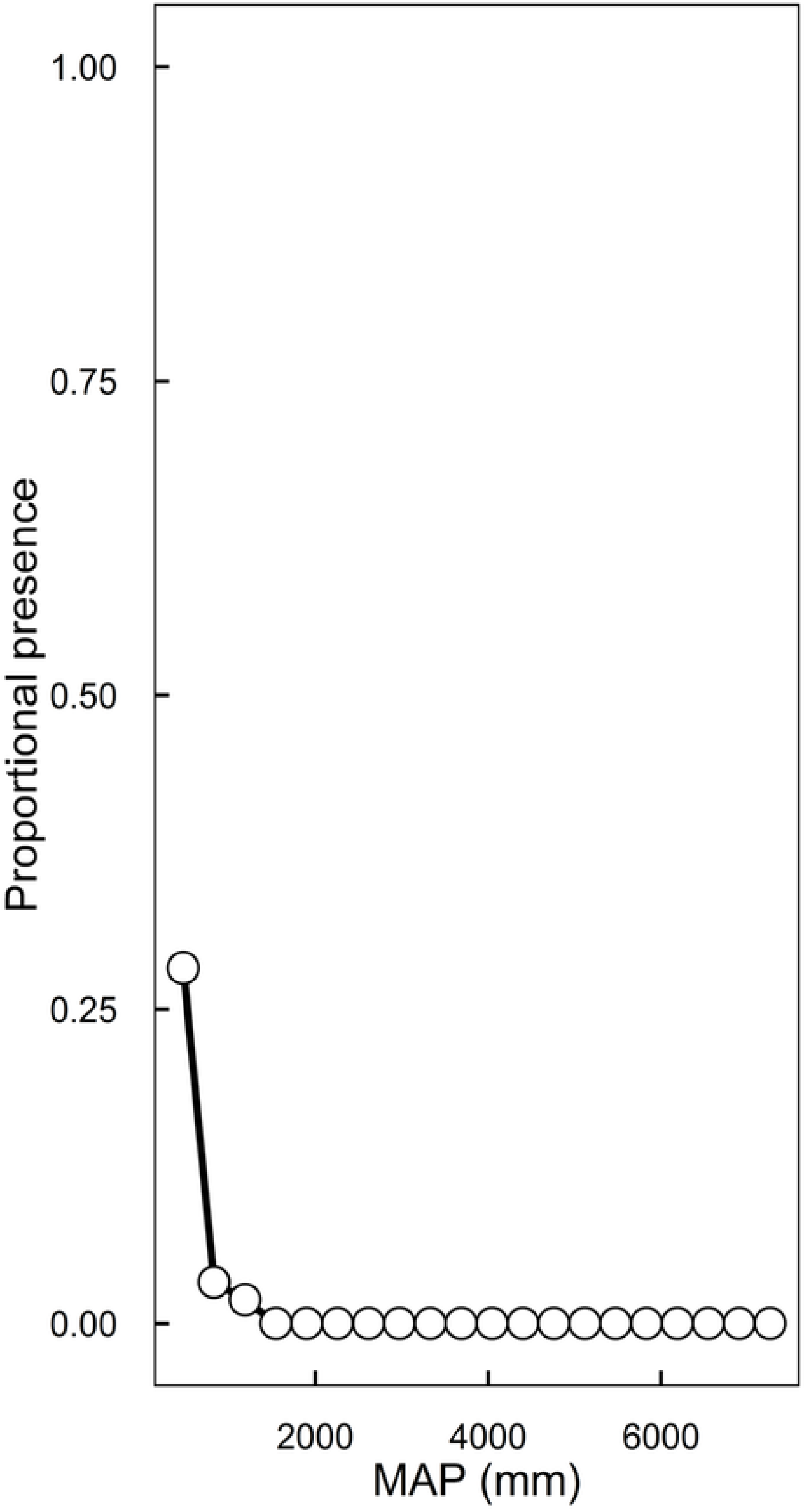

S5
Proportional presence of species along climate gradients

Proportional presence Palila bio15

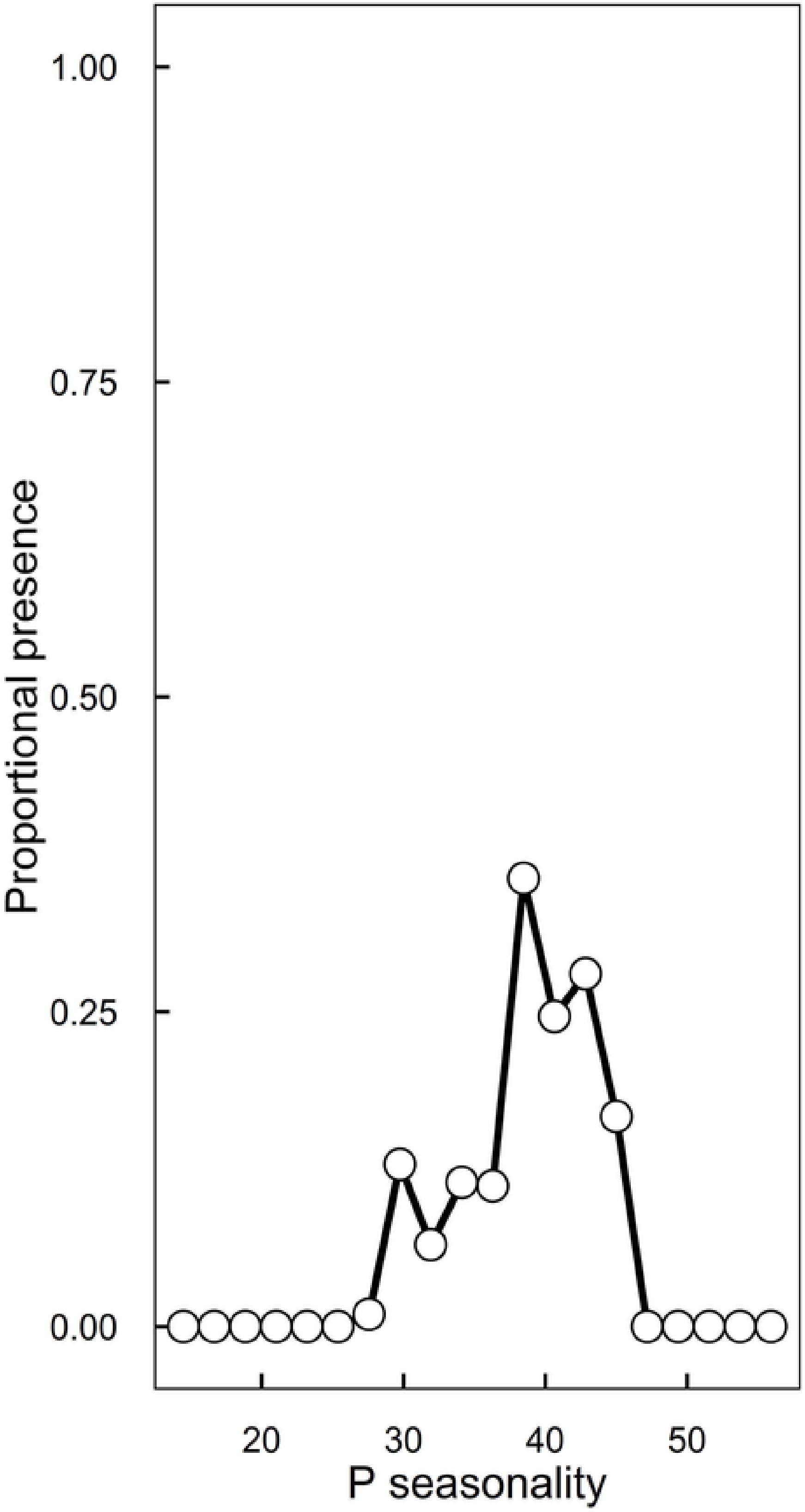

S5
Proportional presence of species along climate gradients

Proportional presence Palila bio7

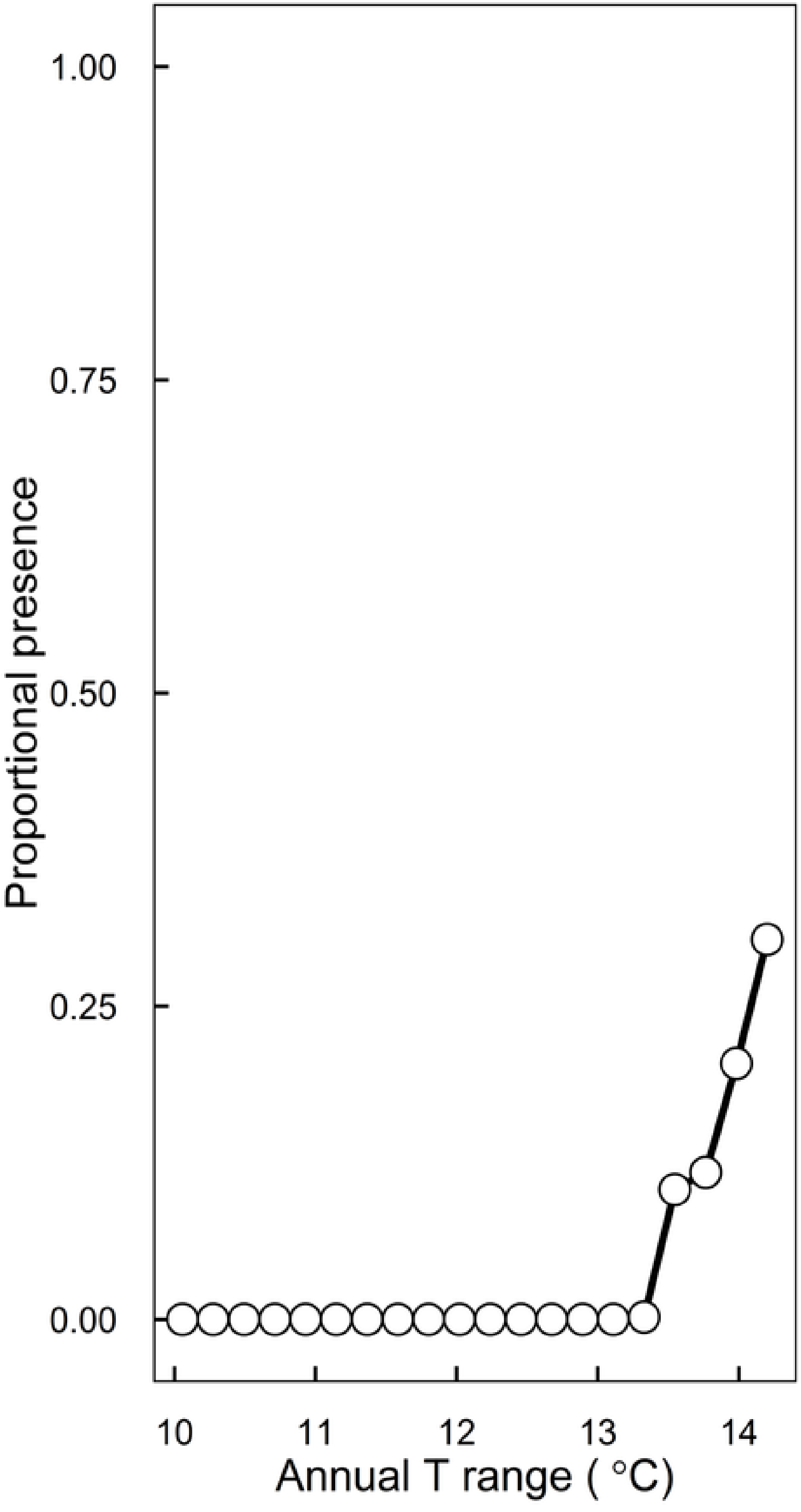

S5
Proportional presence of species along climate gradients

Proportional presence Puaiohi bio1

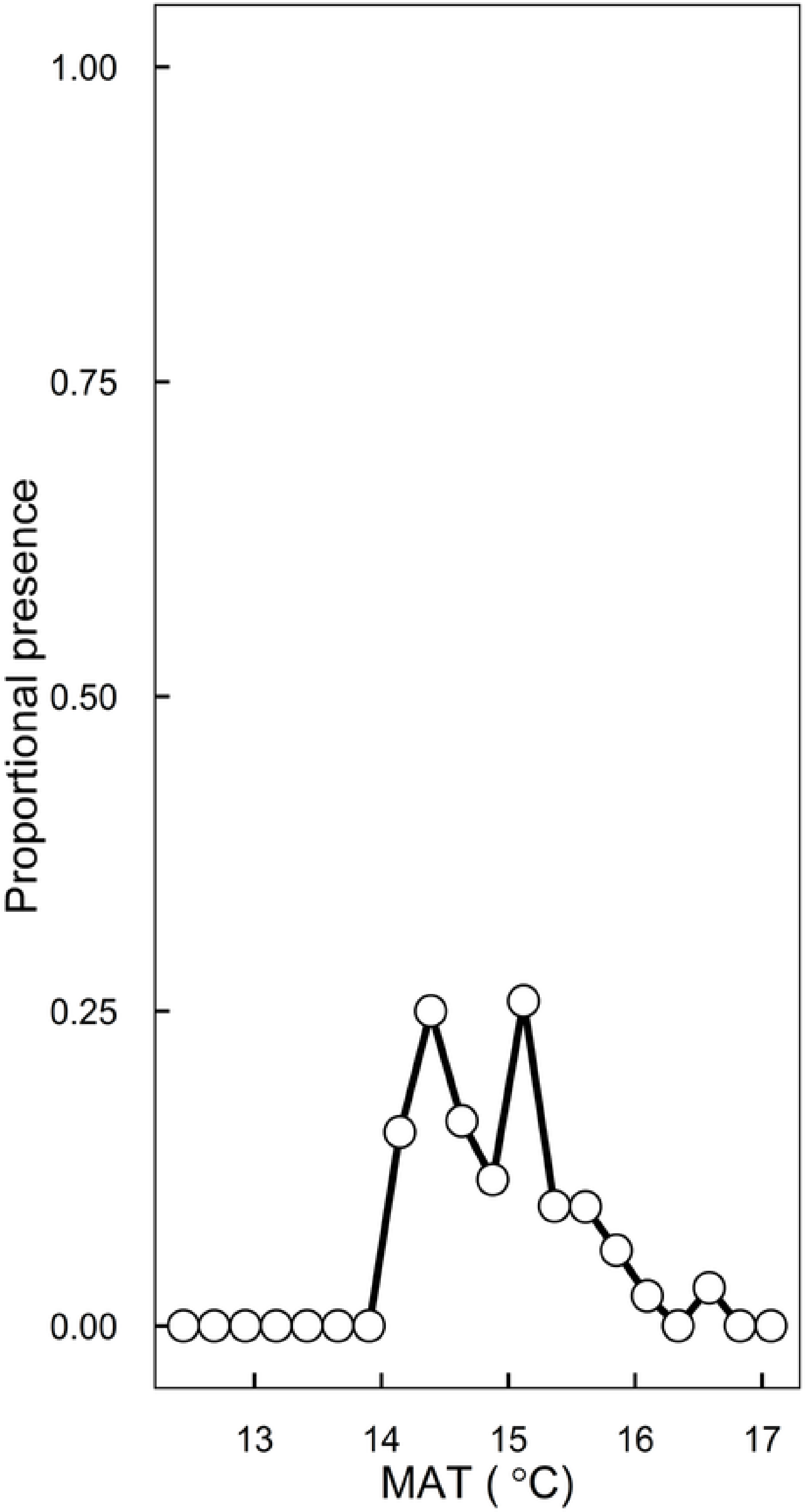

S5
Proportional presence of species along climate gradients

Proportional presence Puaiohi bio12

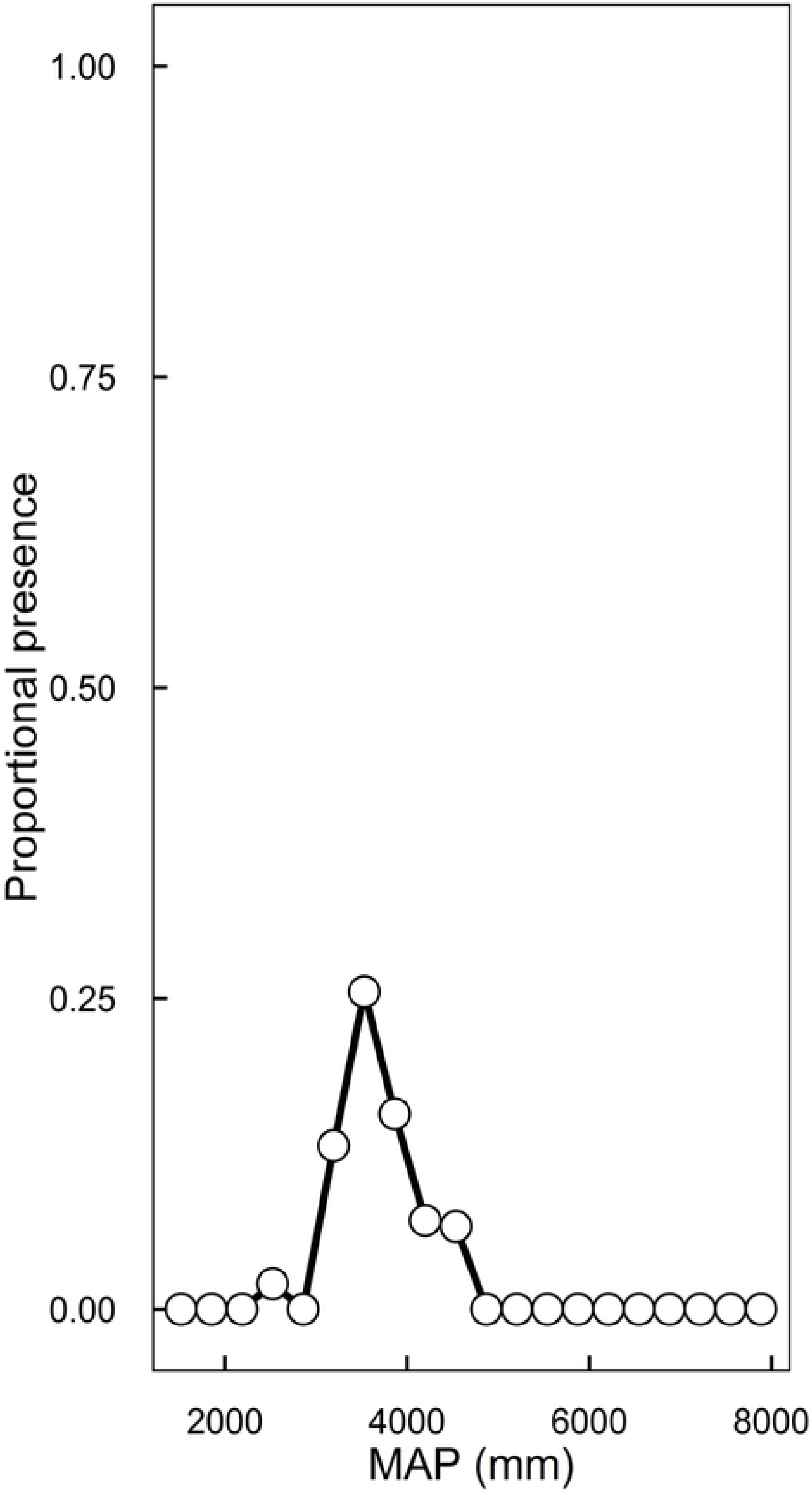

S5

Proportional presence of species along climate gradients

Proportional presence Puaiohi bio15

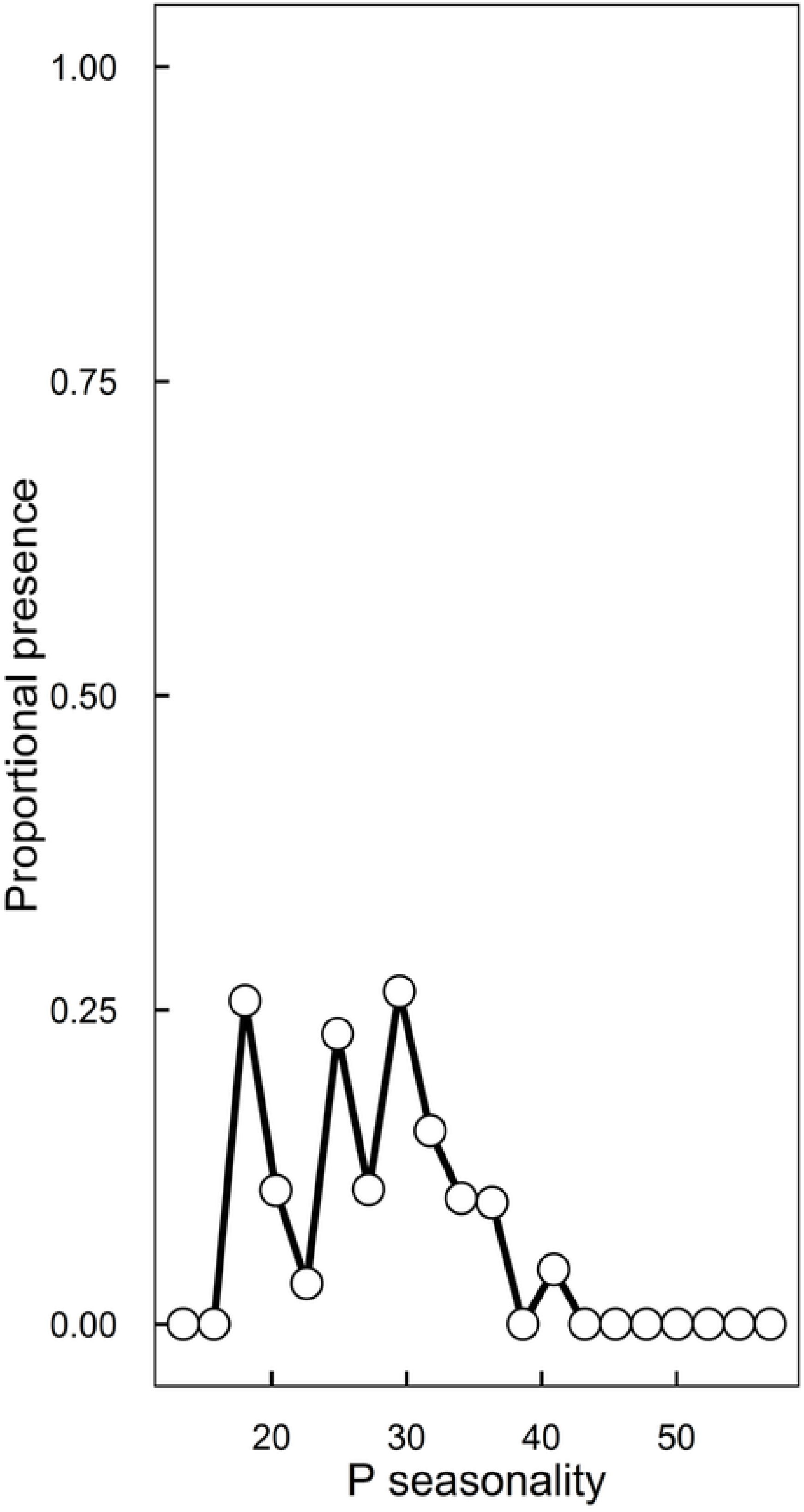

S5
Proportional presence of species along climate gradients

Proportional presence Puaiohi bio7

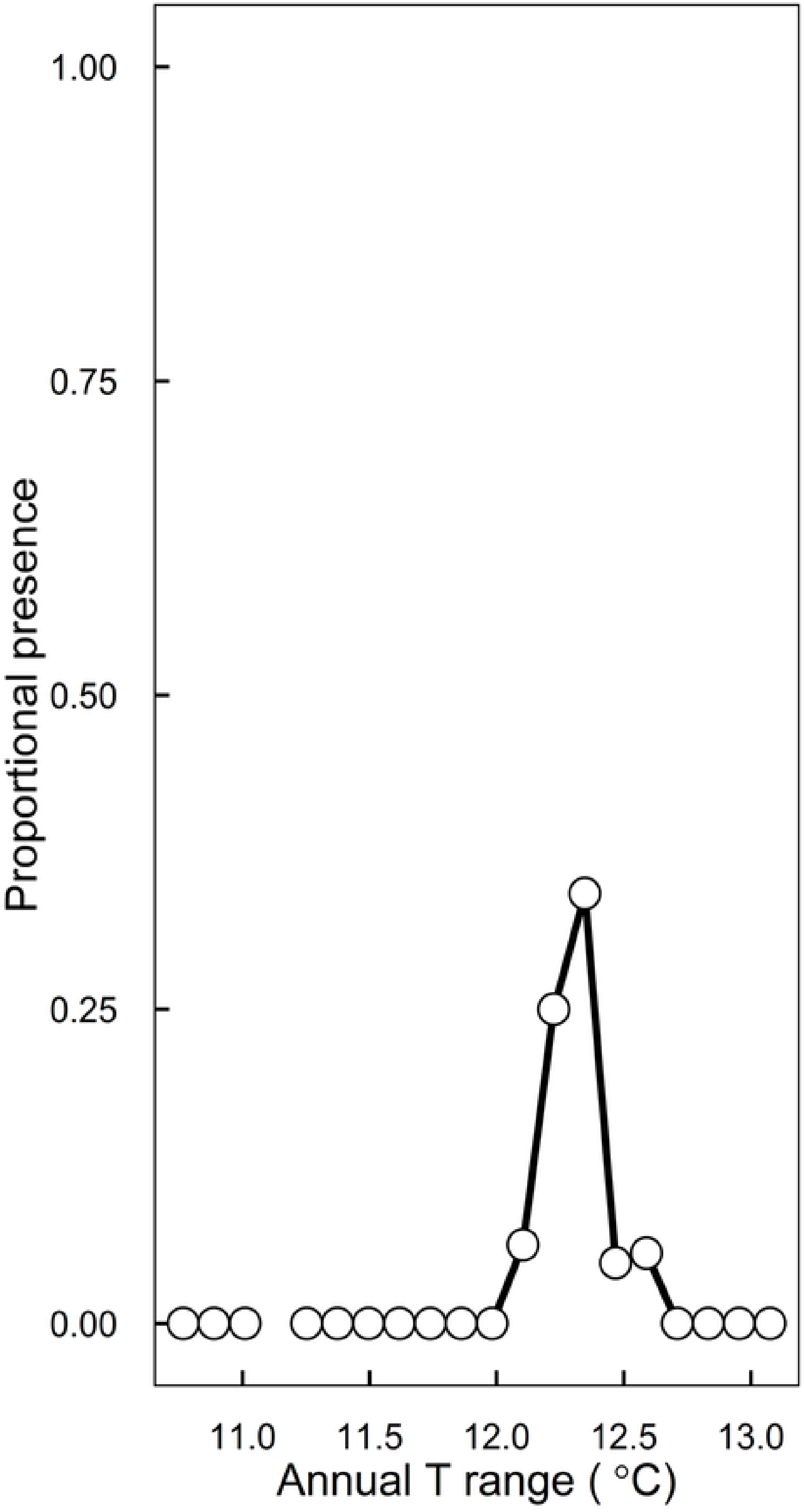

Supplement: S5 File — (PDF) [file pone.0140389.s005.pdf]
