# Supplementary material for: Large-Scale Range Collapse of Hawaiian Forest Birds under Climate Change and the Need 21st Century Conservation Options
Source: PLoS One. 2015 Oct 28;10(10):e0140389. doi: 10.1371/journal.pone.0140389 (PMC4625087; doi:10.1371/journal.pone.0140389)

Appendix S6. Maps of climate-based species range shifts within primary habitat.

Maps for each individual species expected range shifts as shown and described in figure 2 of the manuscript.

S6: Maps of climate-based species range shifts within primary habitat
HIGH MODEL RELIABILITY SPECIES

Akekee

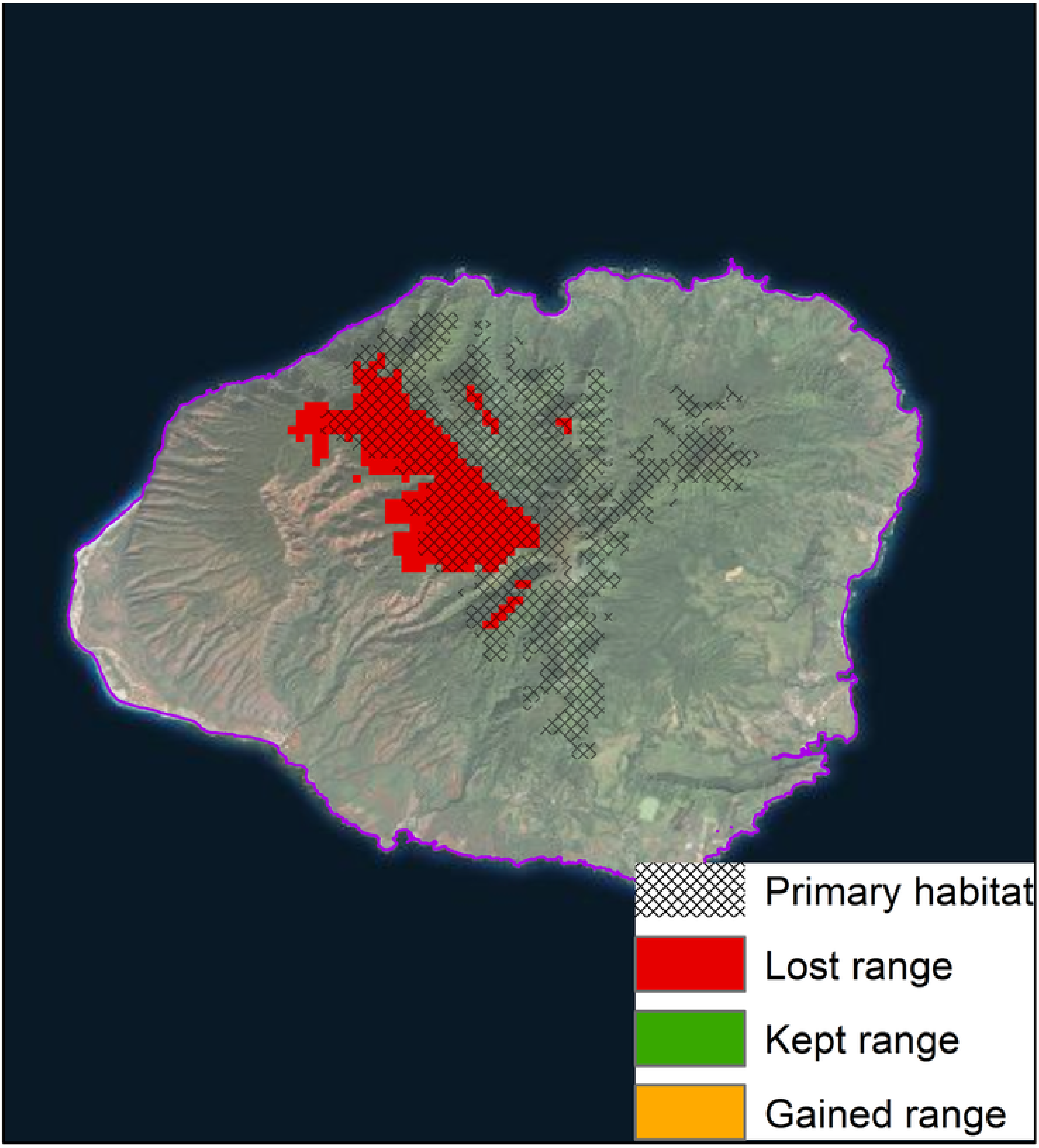

S6: Maps of climate-based species range shifts within primary habitat
HIGH MODEL RELIABILITY SPECIES

Akiapolauu

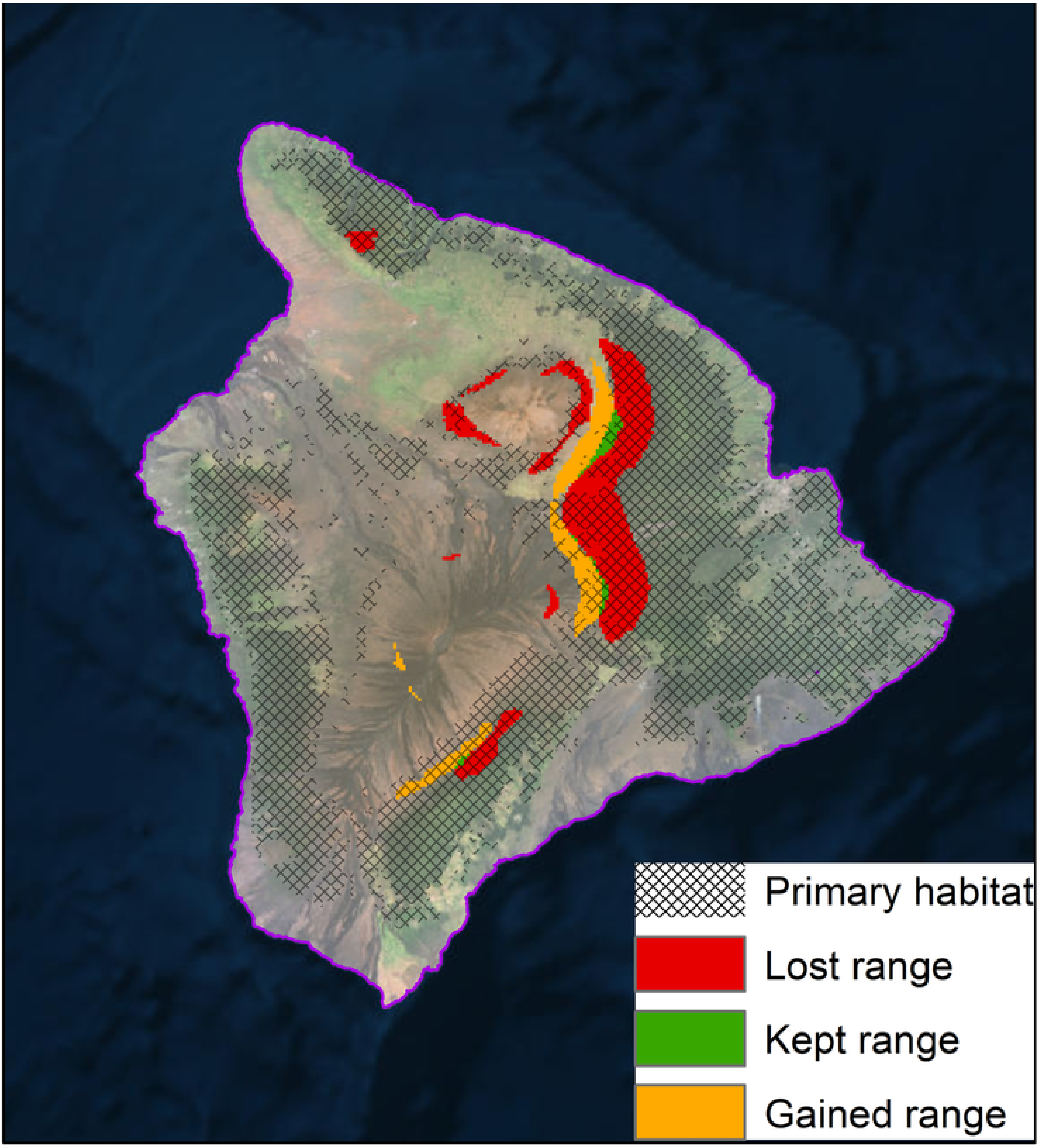

S6: Maps of climate-based species range shifts within primary habitat
HIGH MODEL RELIABILITY SPECIES

Akikiki

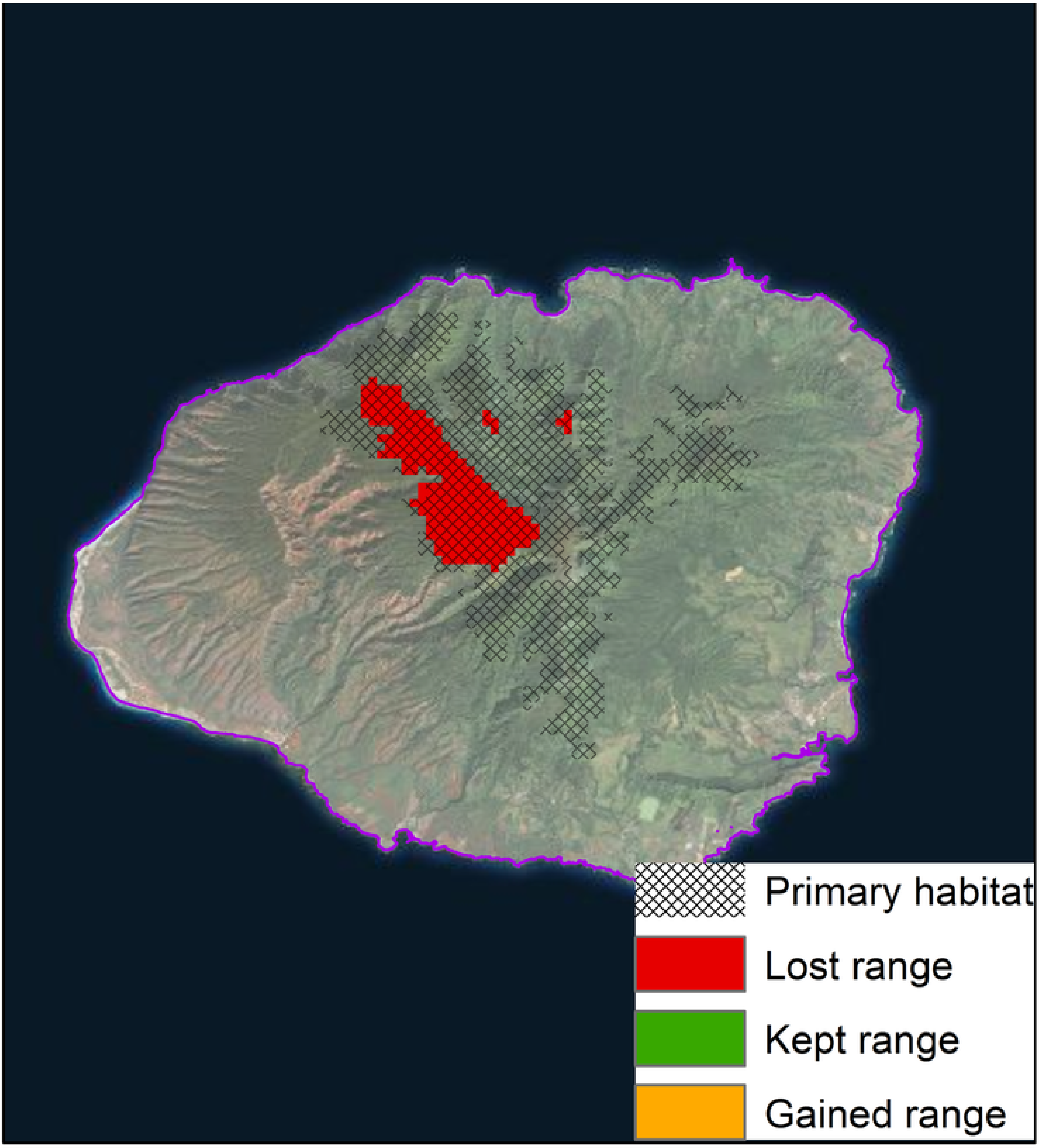

S6: Maps of climate-based species range shifts within primary habitat
HIGH MODEL RELIABILITY SPECIES

Akohekohe

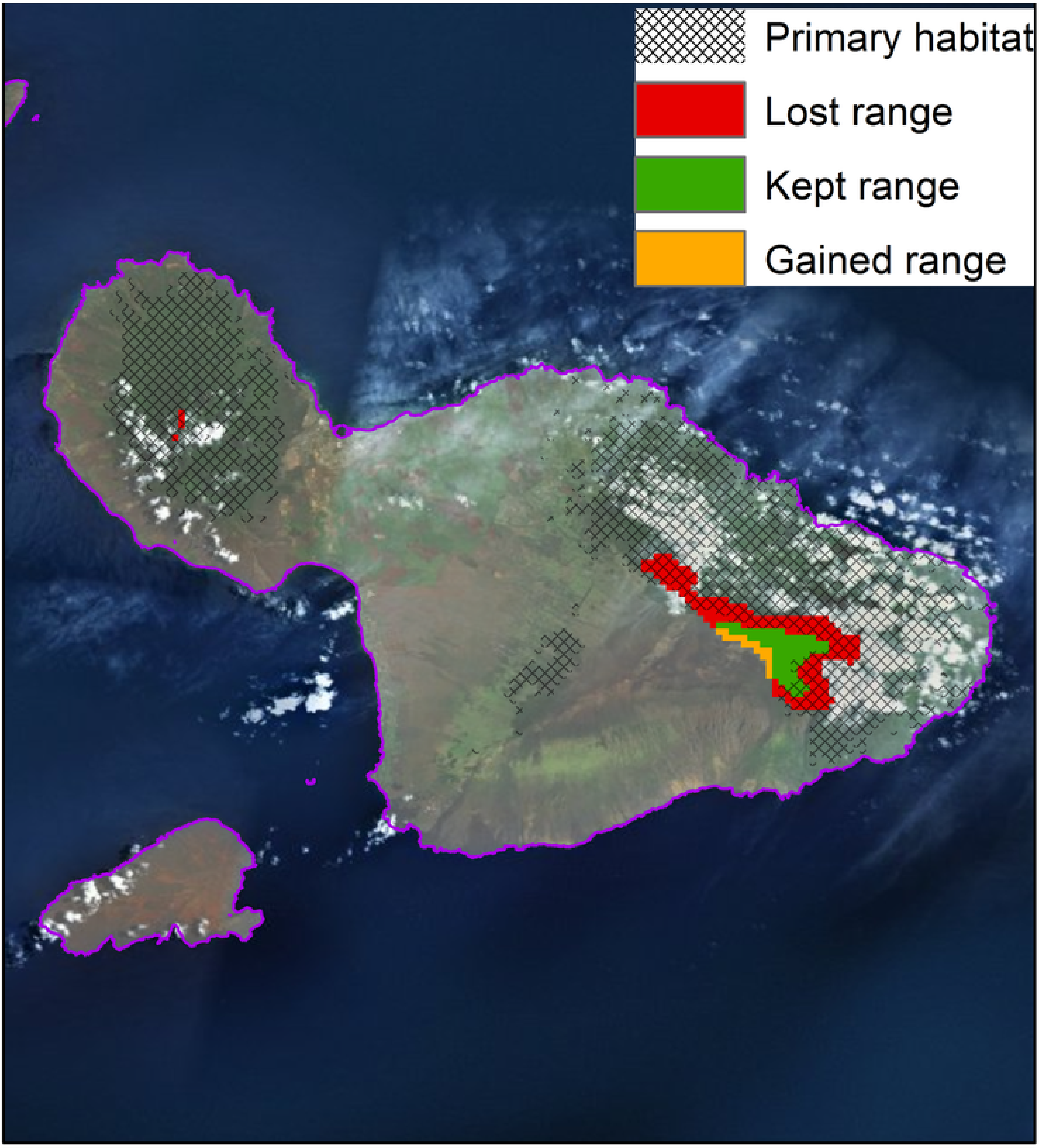

S6: Maps of climate-based species range shifts within primary habitat
HIGH MODEL RELIABILITY SPECIES

Hawaii Akepa

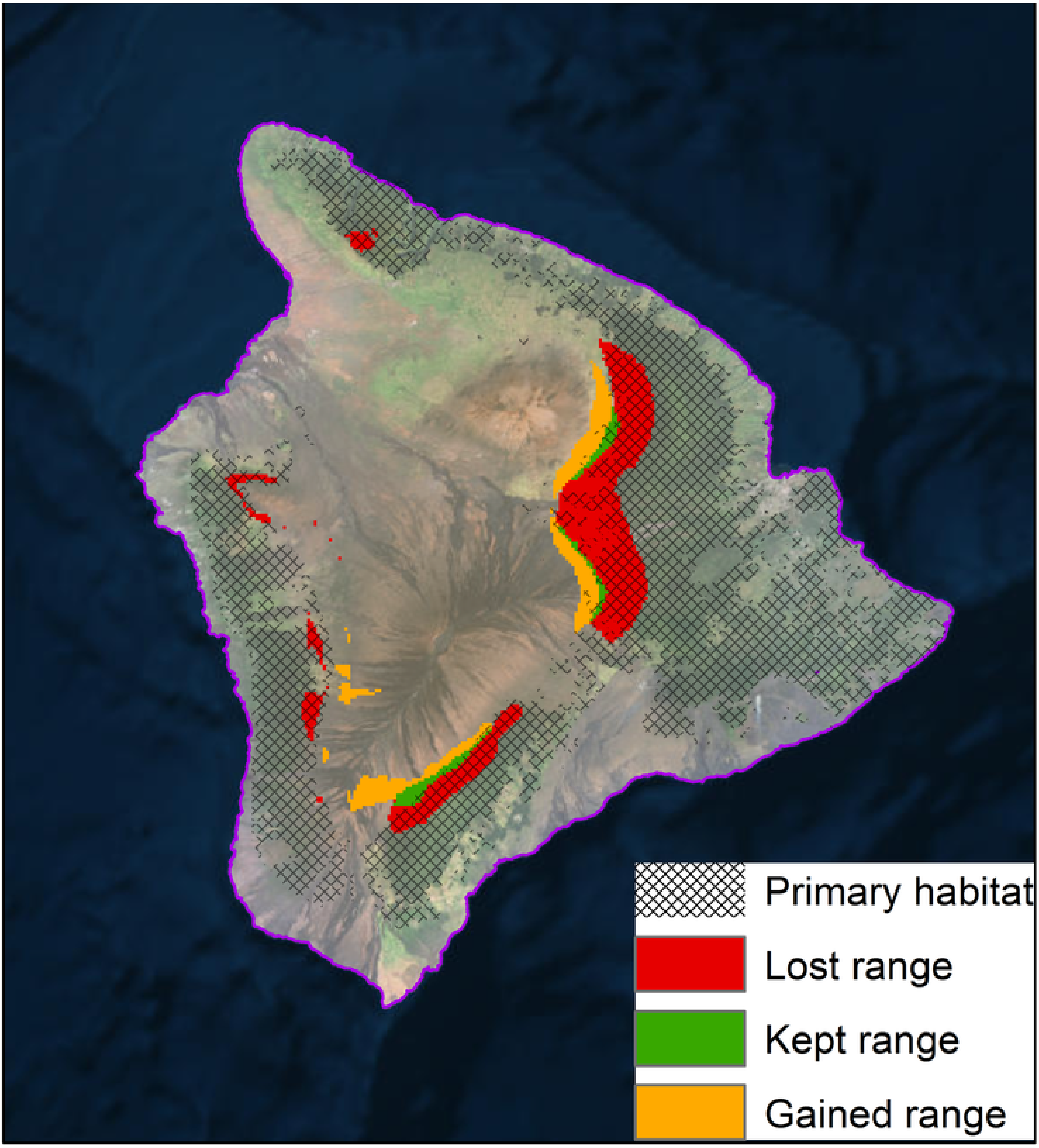

S6: Maps of climate-based species range shifts within primary habitat
HIGH MODEL RELIABILITY SPECIES

Hawaii Creeper

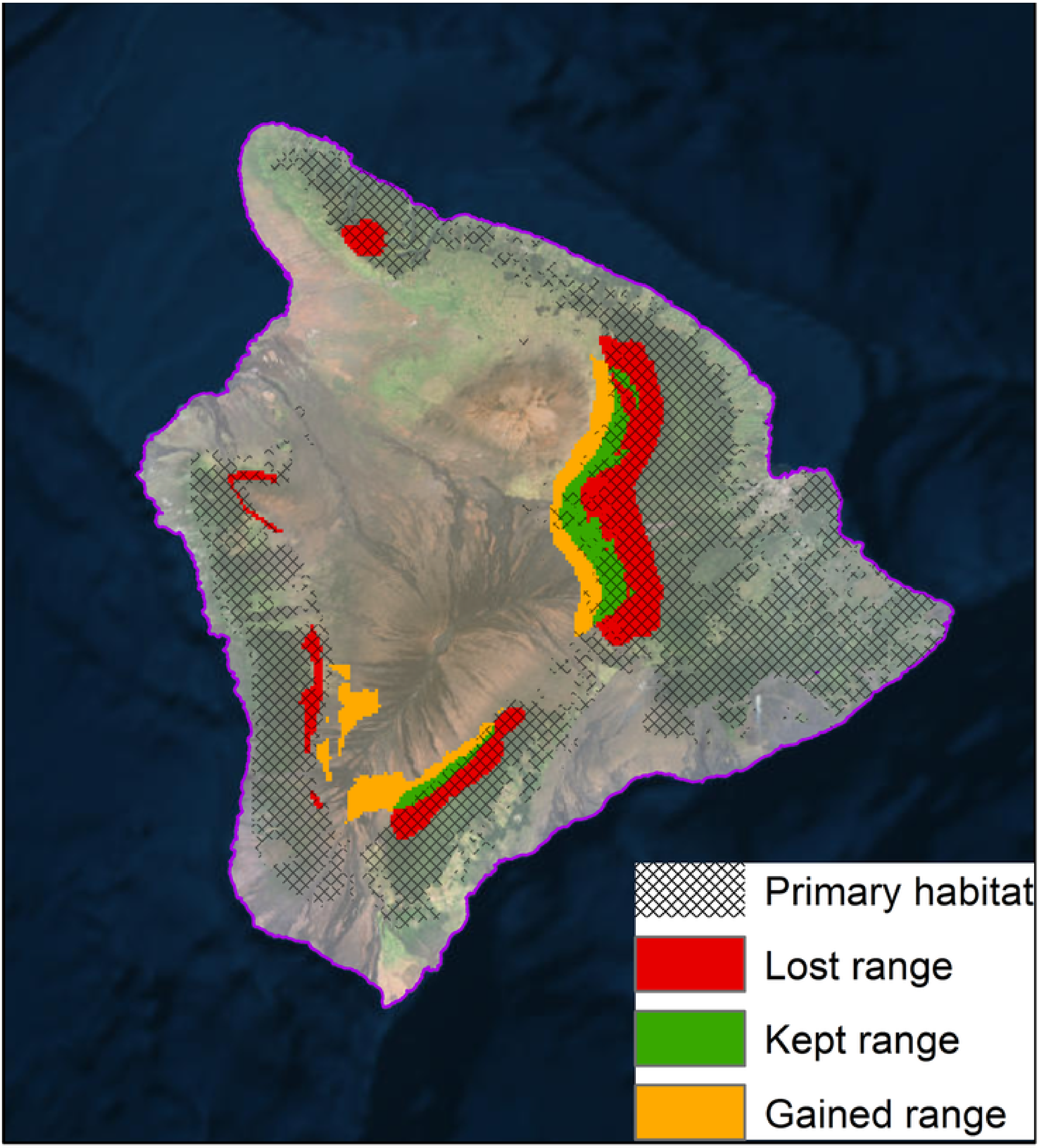

S6: Maps of climate-based species range shifts within primary habitat
HIGH MODEL RELIABILITY SPECIES

Iiwi

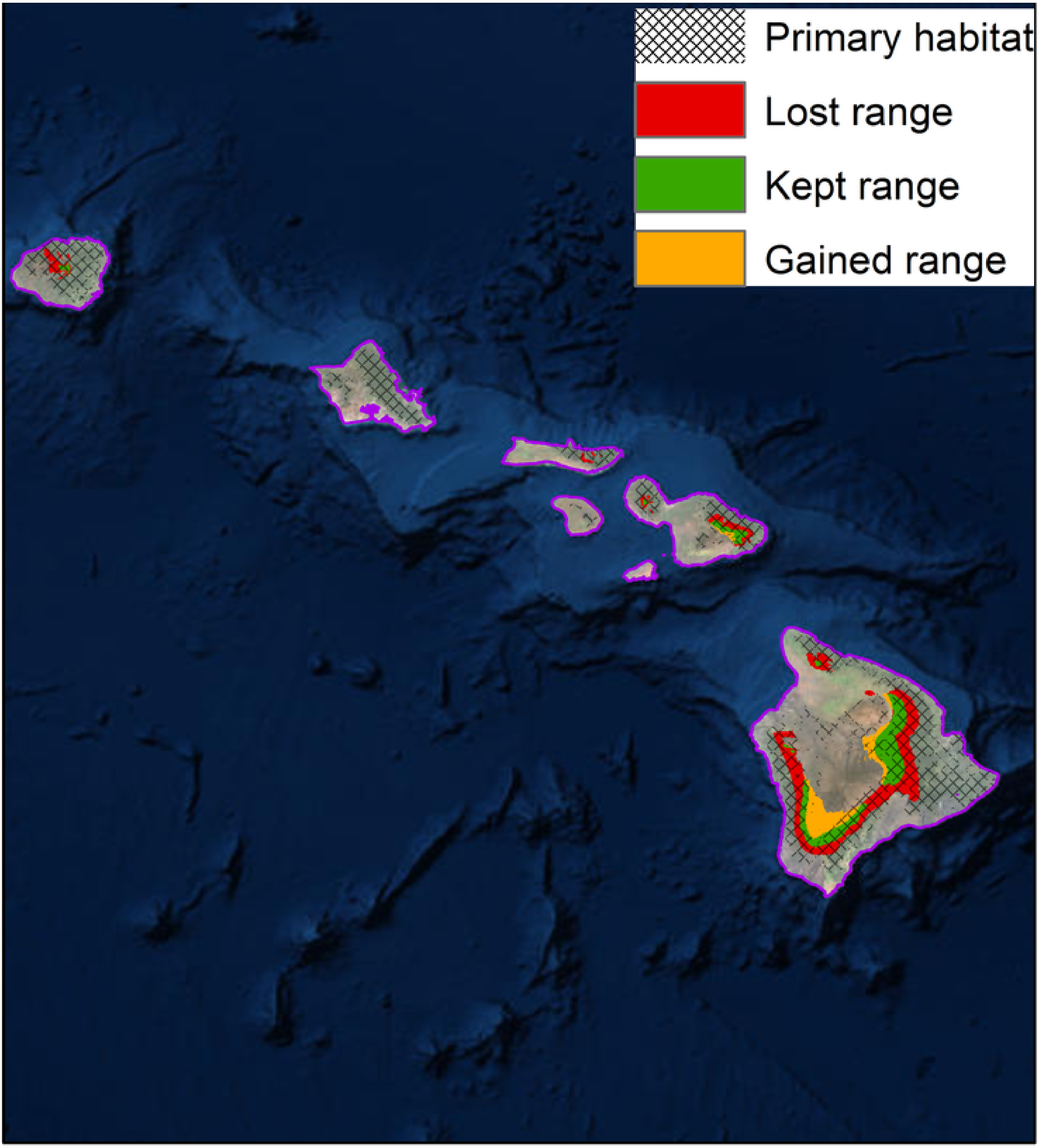

S6: Maps of climate-based species range shifts within primary habitat
HIGH MODEL RELIABILITY SPECIES

Maui Alauahio

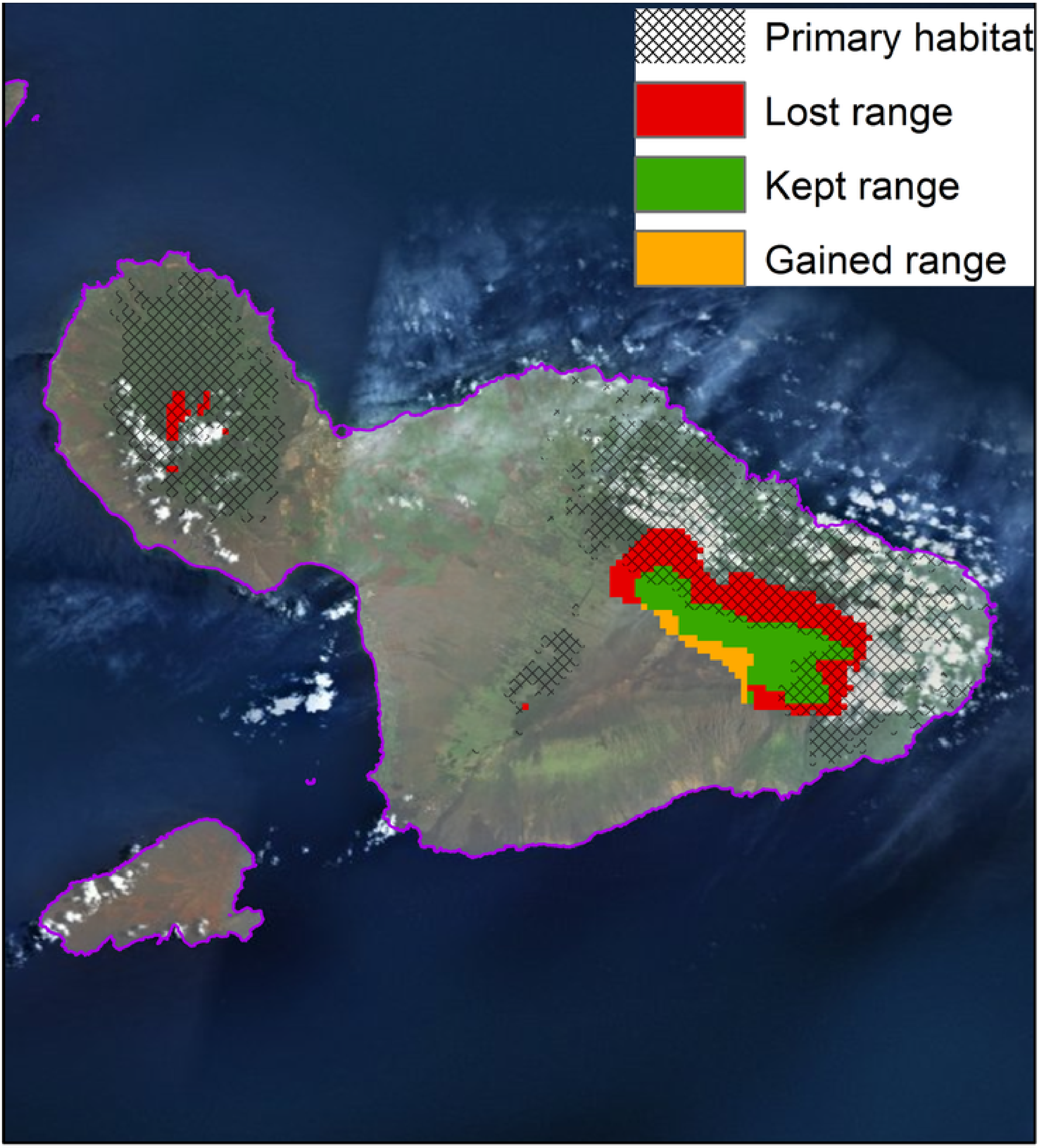

S6: Maps of climate-based species range shifts within primary habitat
HIGH MODEL RELIABILITY SPECIES

Maui Parrotbill

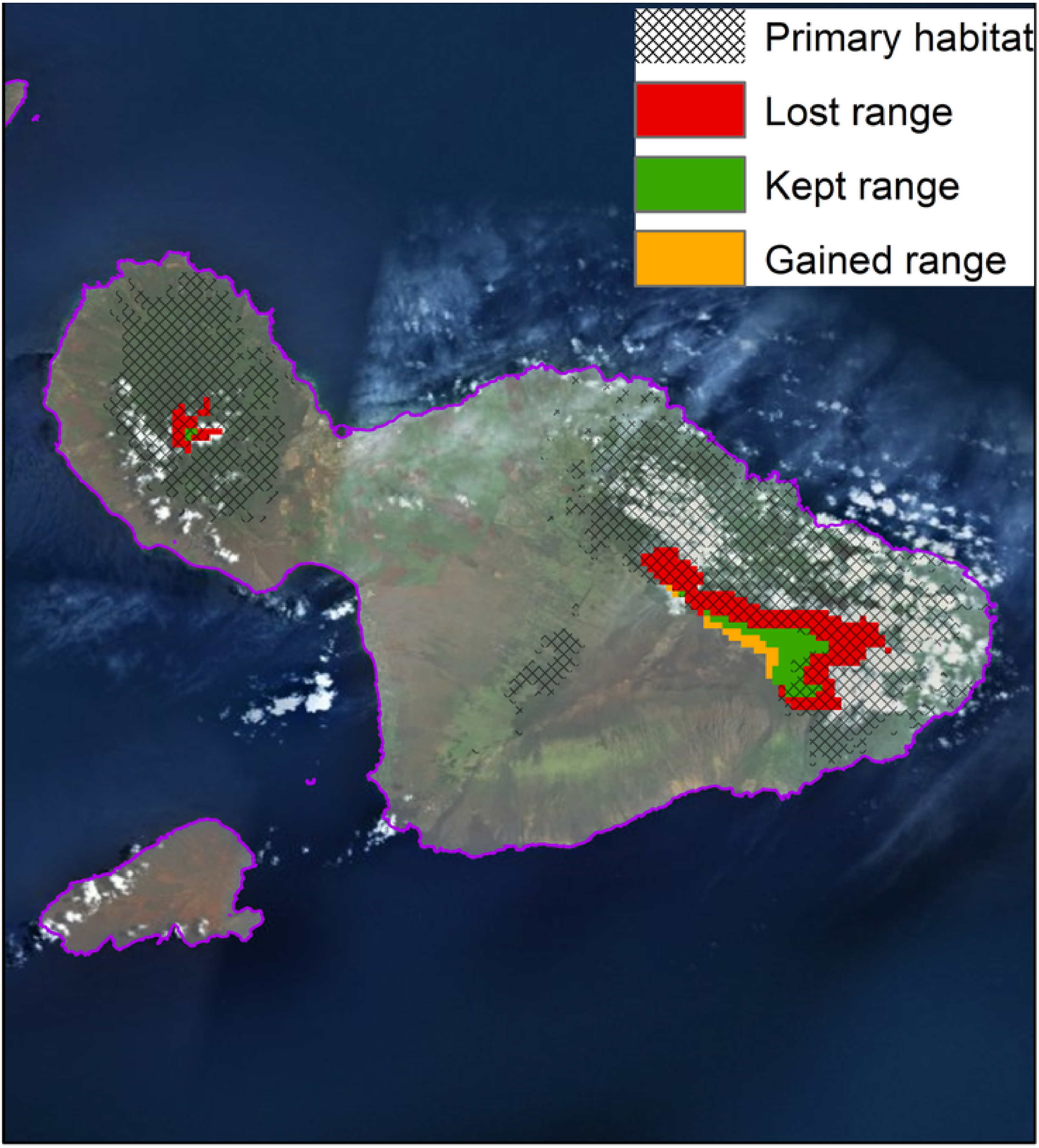

S6: Maps of climate-based species range shifts within primary habitat
HIGH MODEL RELIABILITY SPECIES

Puaiohi

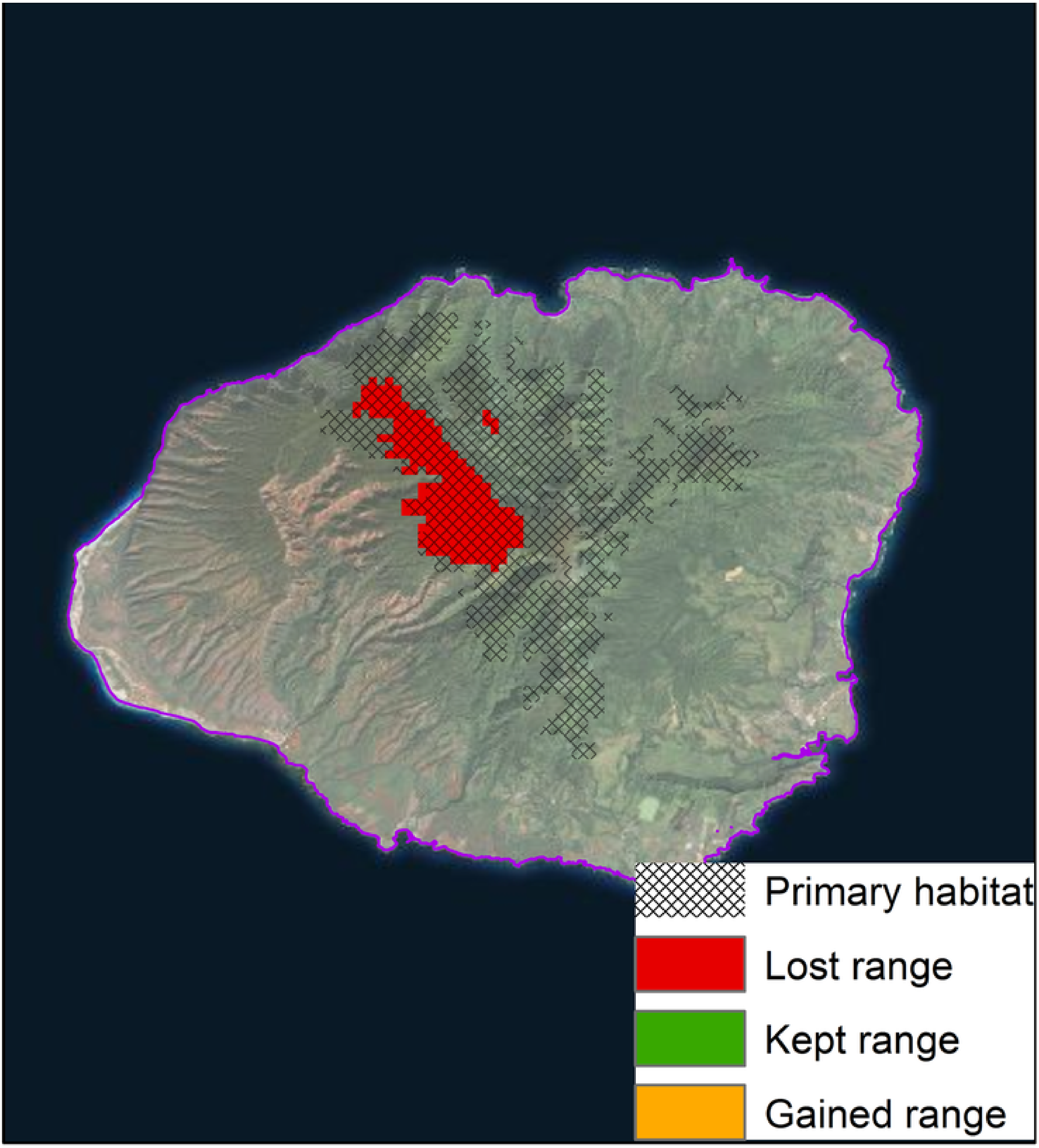

S7: Maps of climate-based species range shifts within primary habitat
REDUCED MODEL RELIABILITY SPECIES

Anianiau

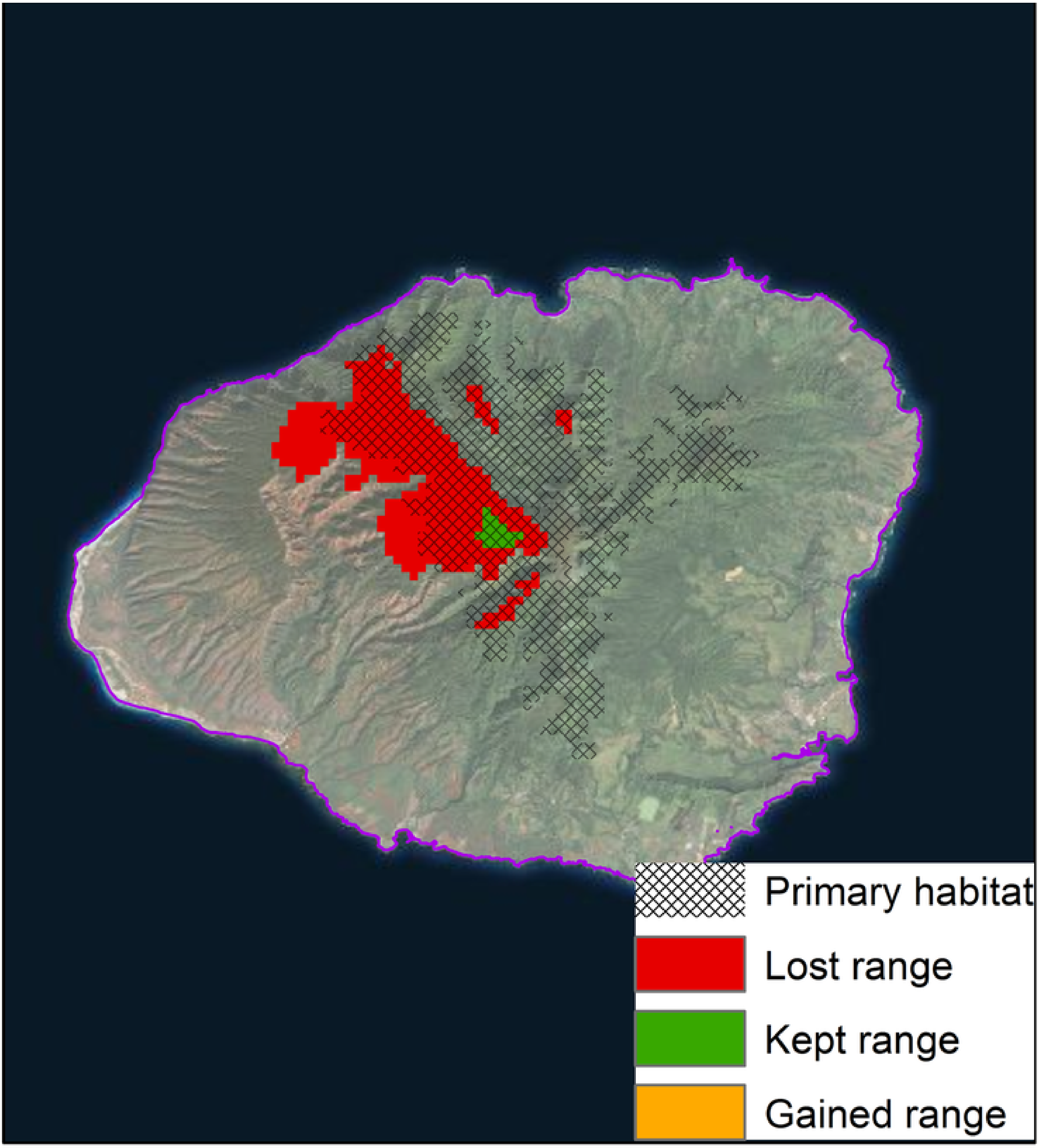

S7: Maps of climate-based species range shifts within primary habitat
REDUCED MODEL RELIABILITY SPECIES

Apapane

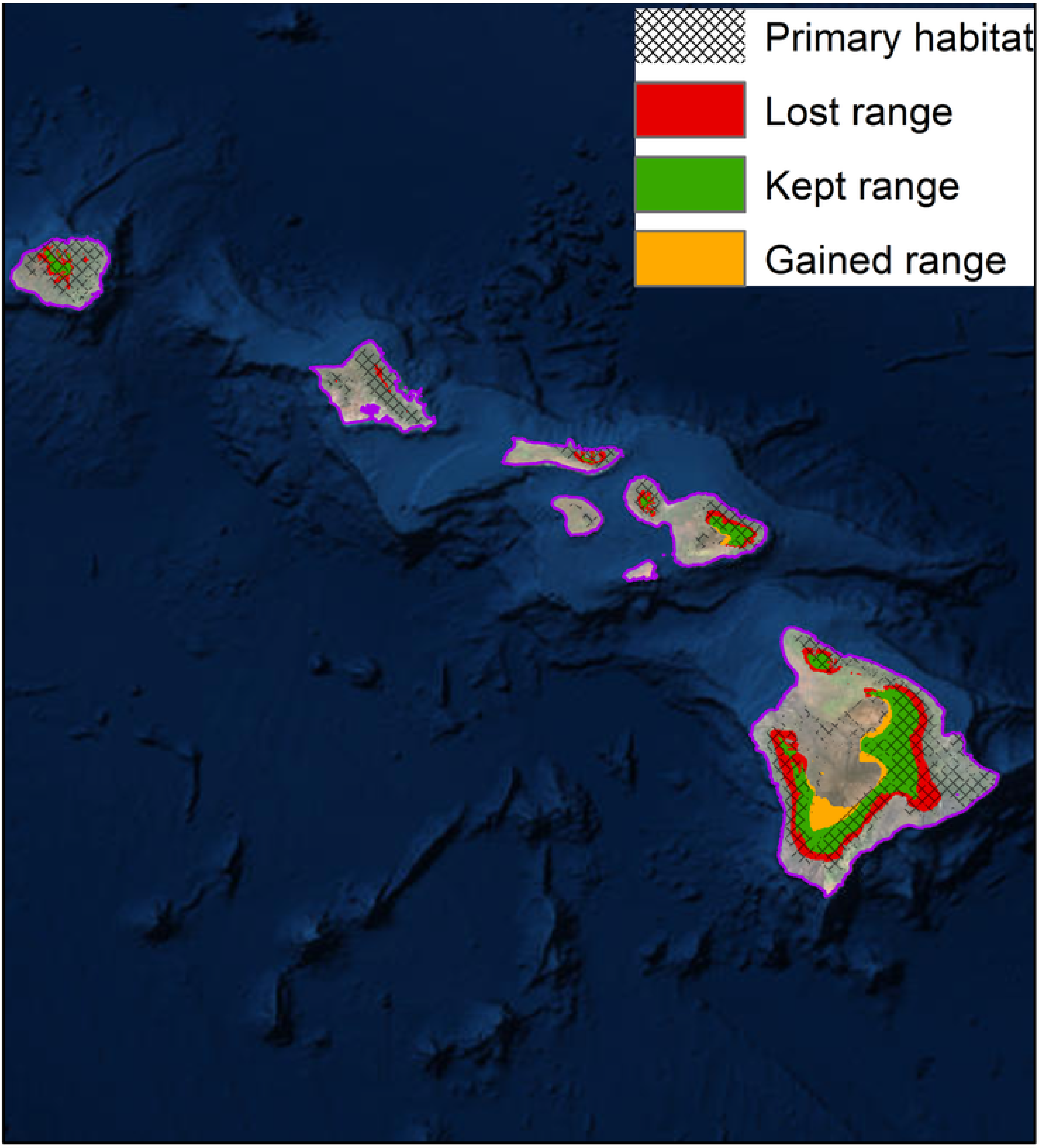

S7: Maps of climate-based species range shifts within primary habitat
REDUCED MODEL RELIABILITY SPECIES

Hawaii Amakihi

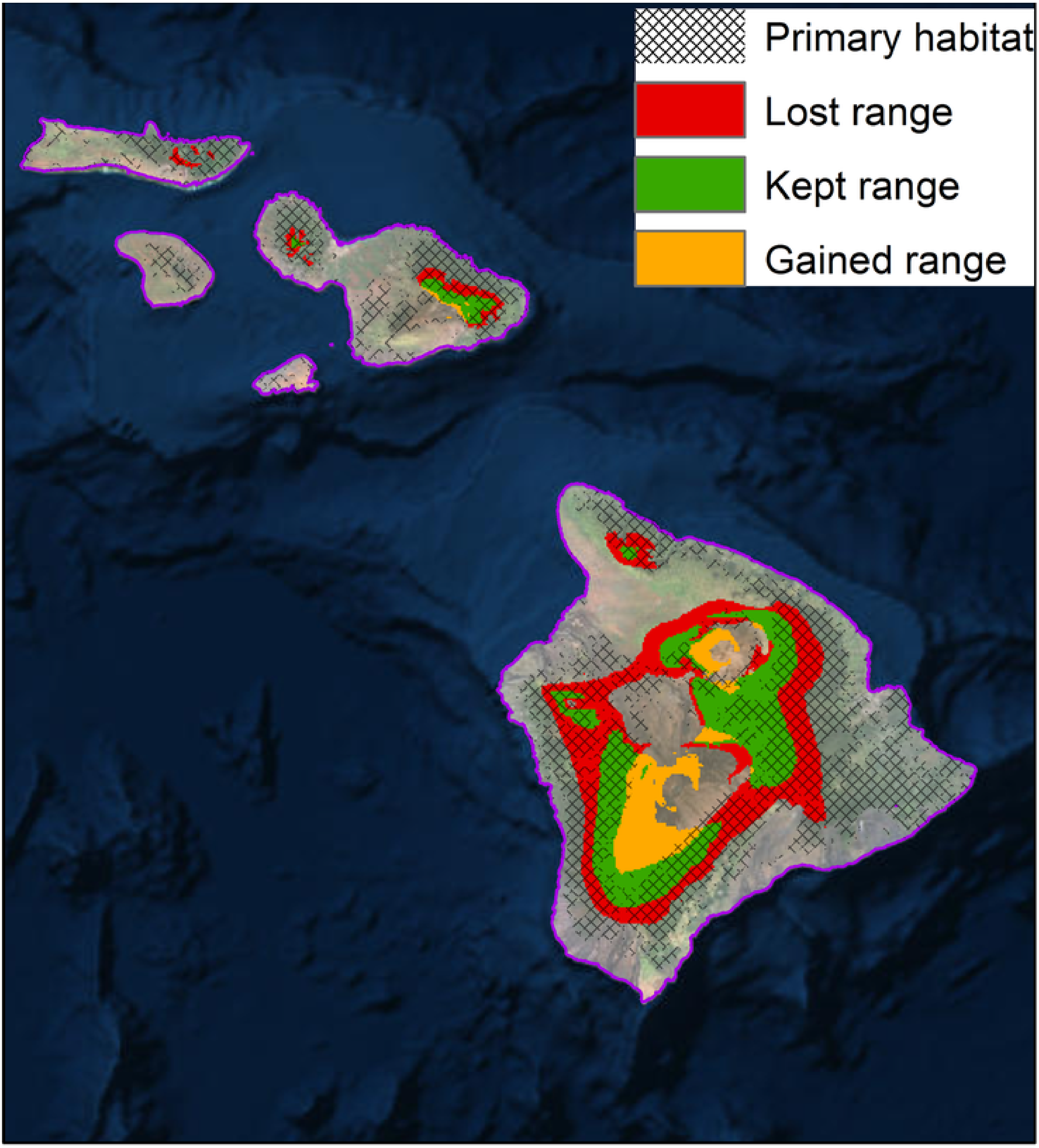

S7: Maps of climate-based species range shifts within primary habitat
REDUCED MODEL RELIABILITY SPECIES

Hawaii Elepaio

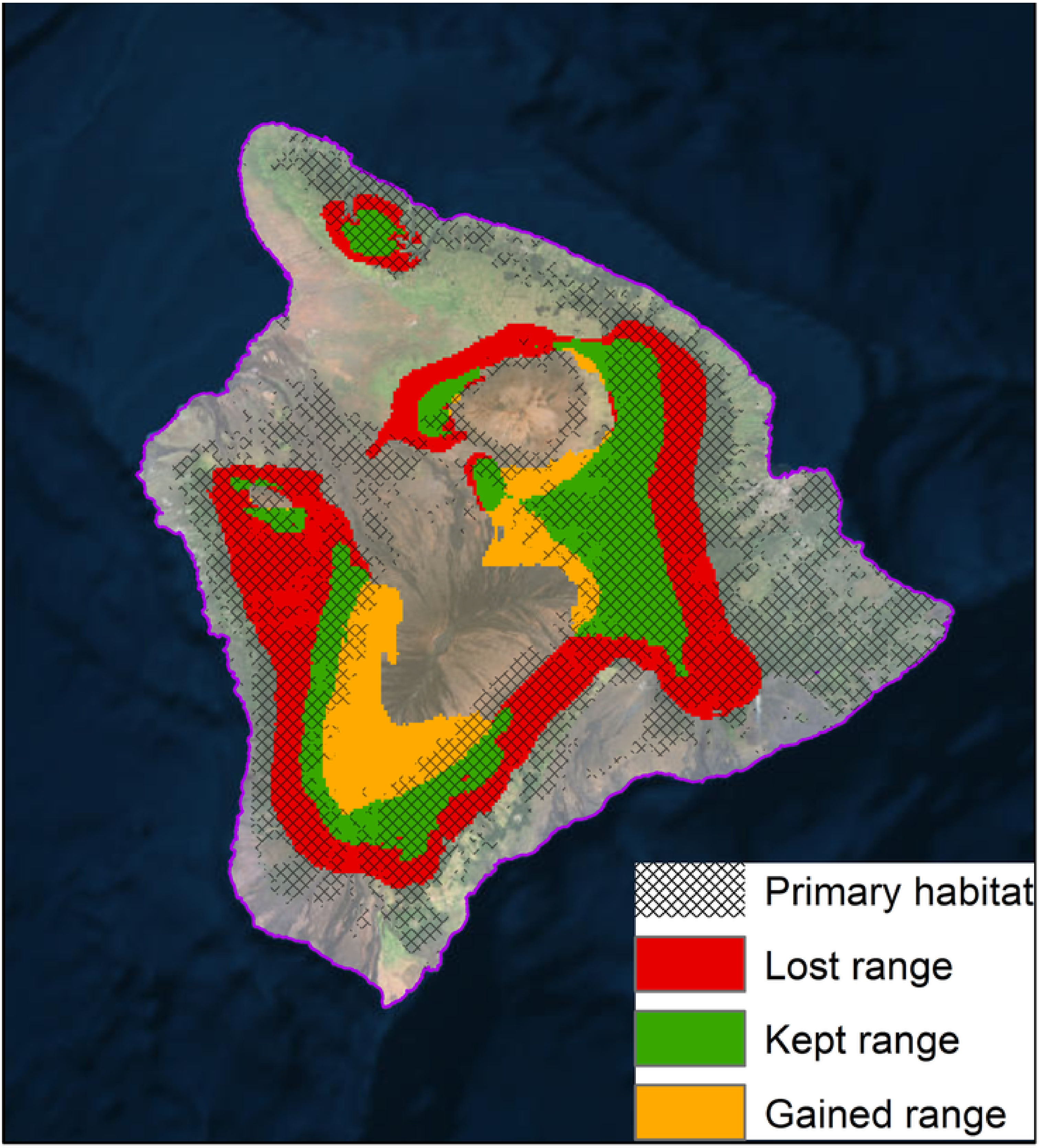

S7: Maps of climate-based species range shifts within primary habitat
REDUCED MODEL RELIABILITY SPECIES

Kauai Amakihi

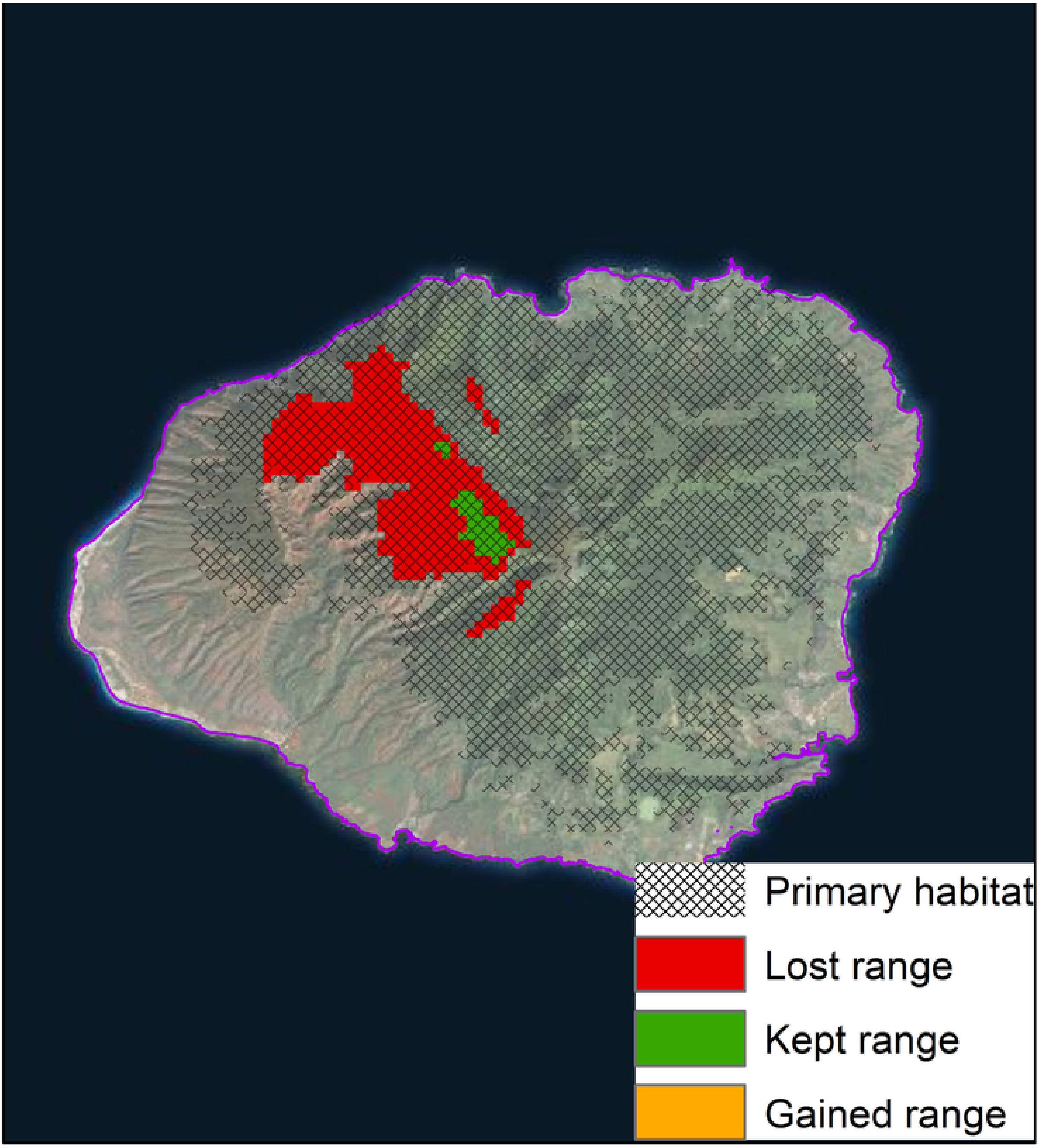

S7: Maps of climate-based species range shifts within primary habitat
REDUCED MODEL RELIABILITY SPECIES

Kauai Elepaio

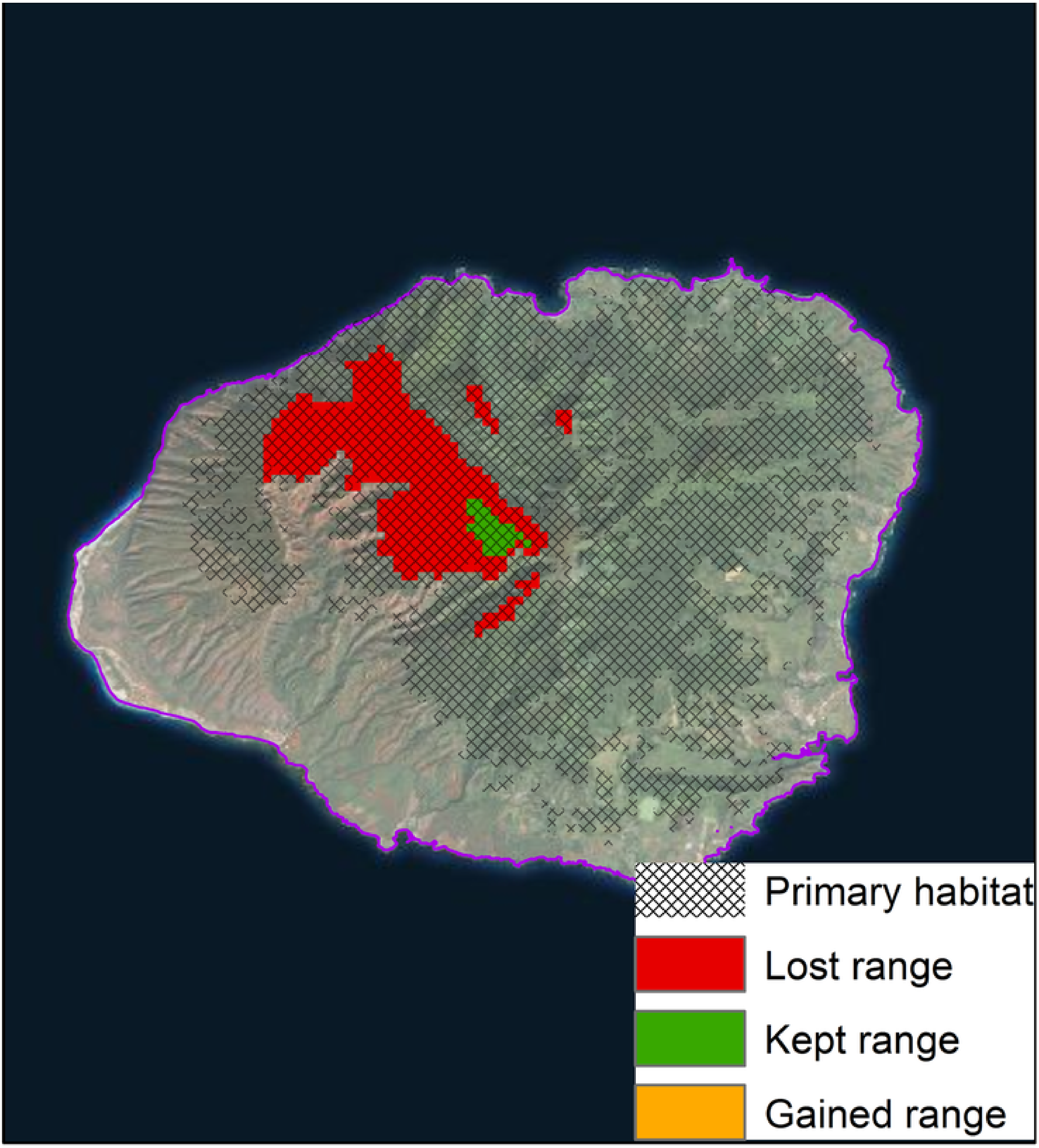

S7: Maps of climate-based species range shifts within primary habitat
REDUCED MODEL RELIABILITY SPECIES

Oahu Amakihi

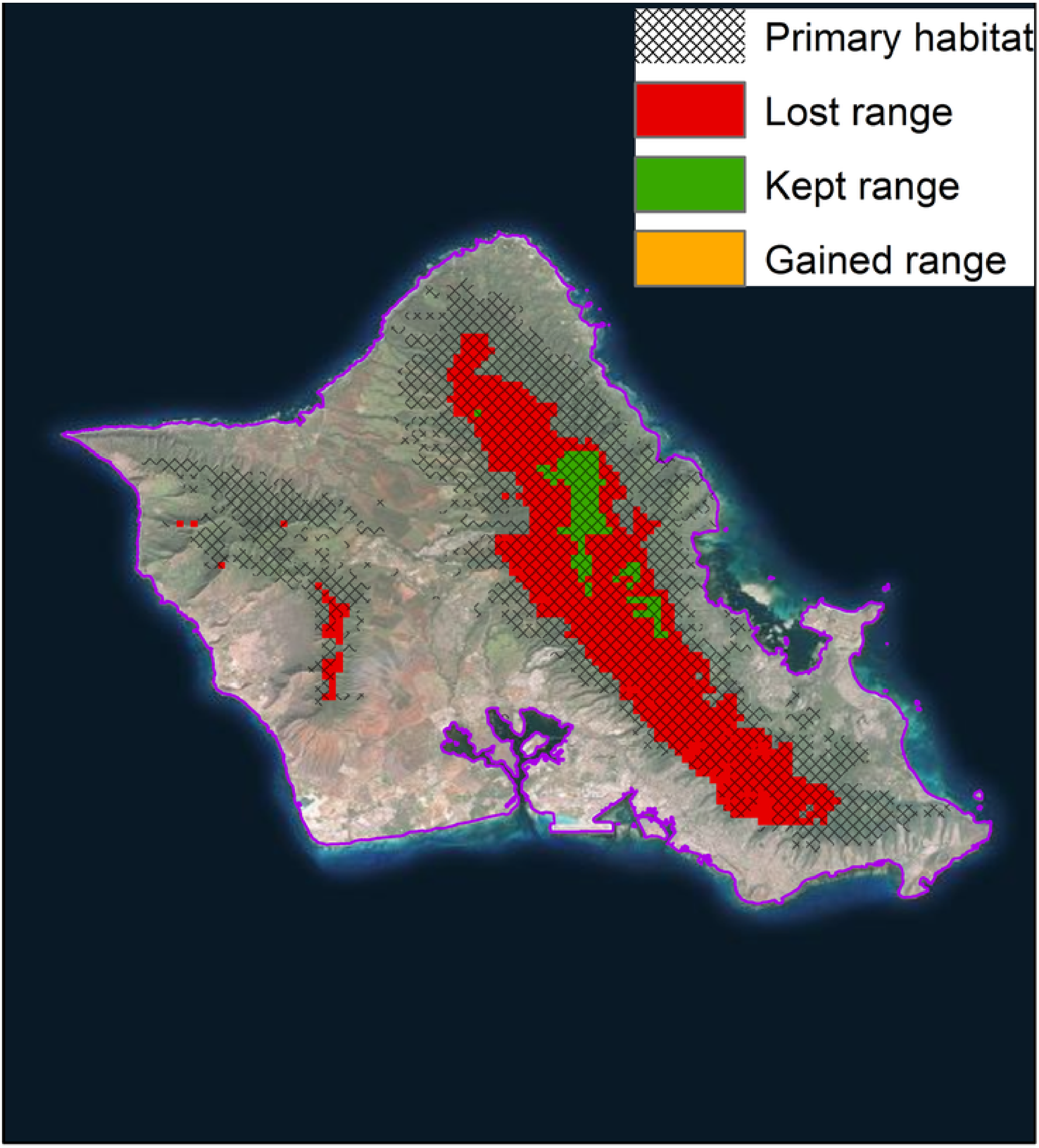

S7: Maps of climate-based species range shifts within primary habitat
REDUCED MODEL RELIABILITY SPECIES

Oahu Elepaio

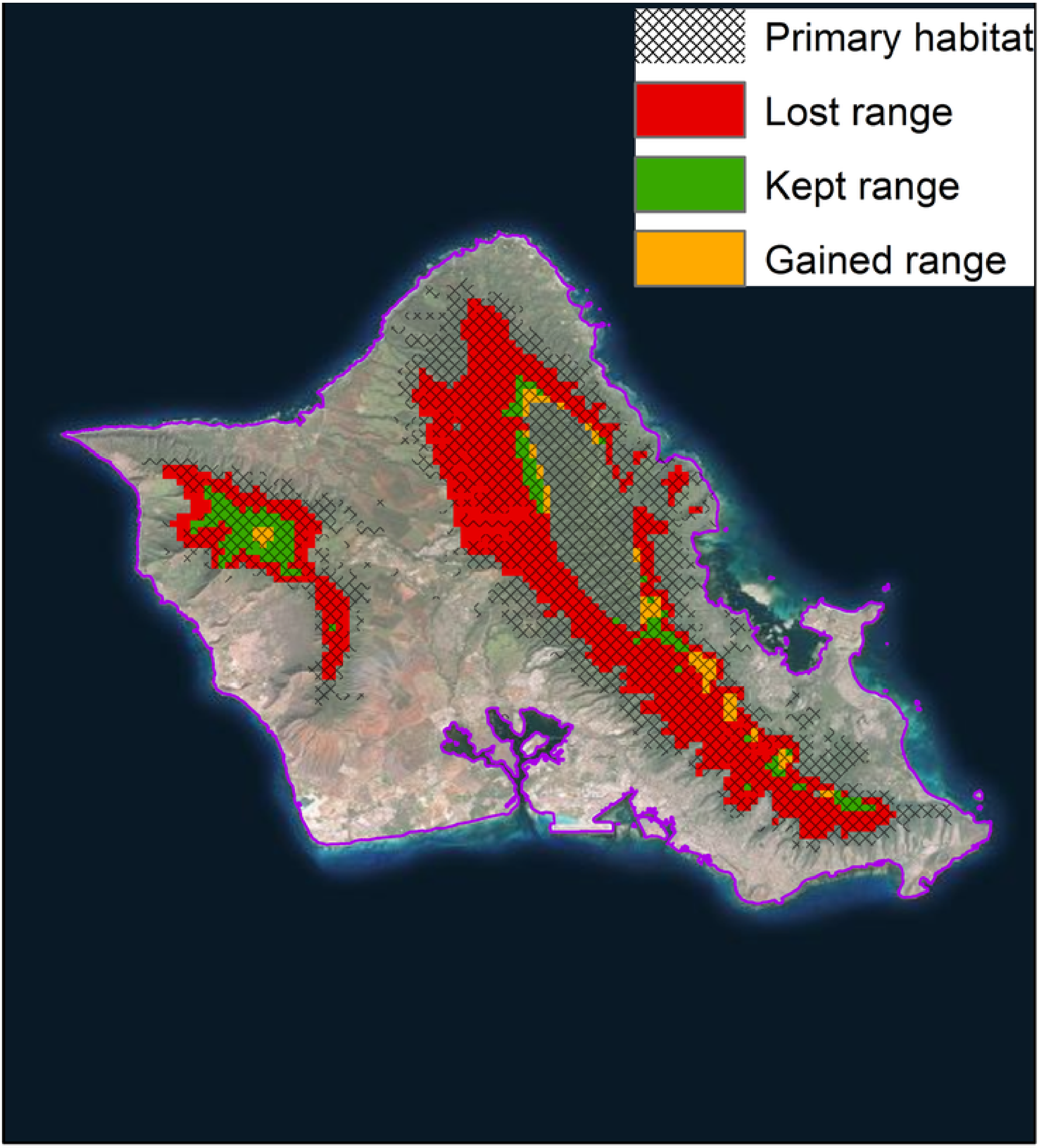

S7: Maps of climate-based species range shifts within primary habitat
REDUCED MODEL RELIABILITY SPECIES

Omao

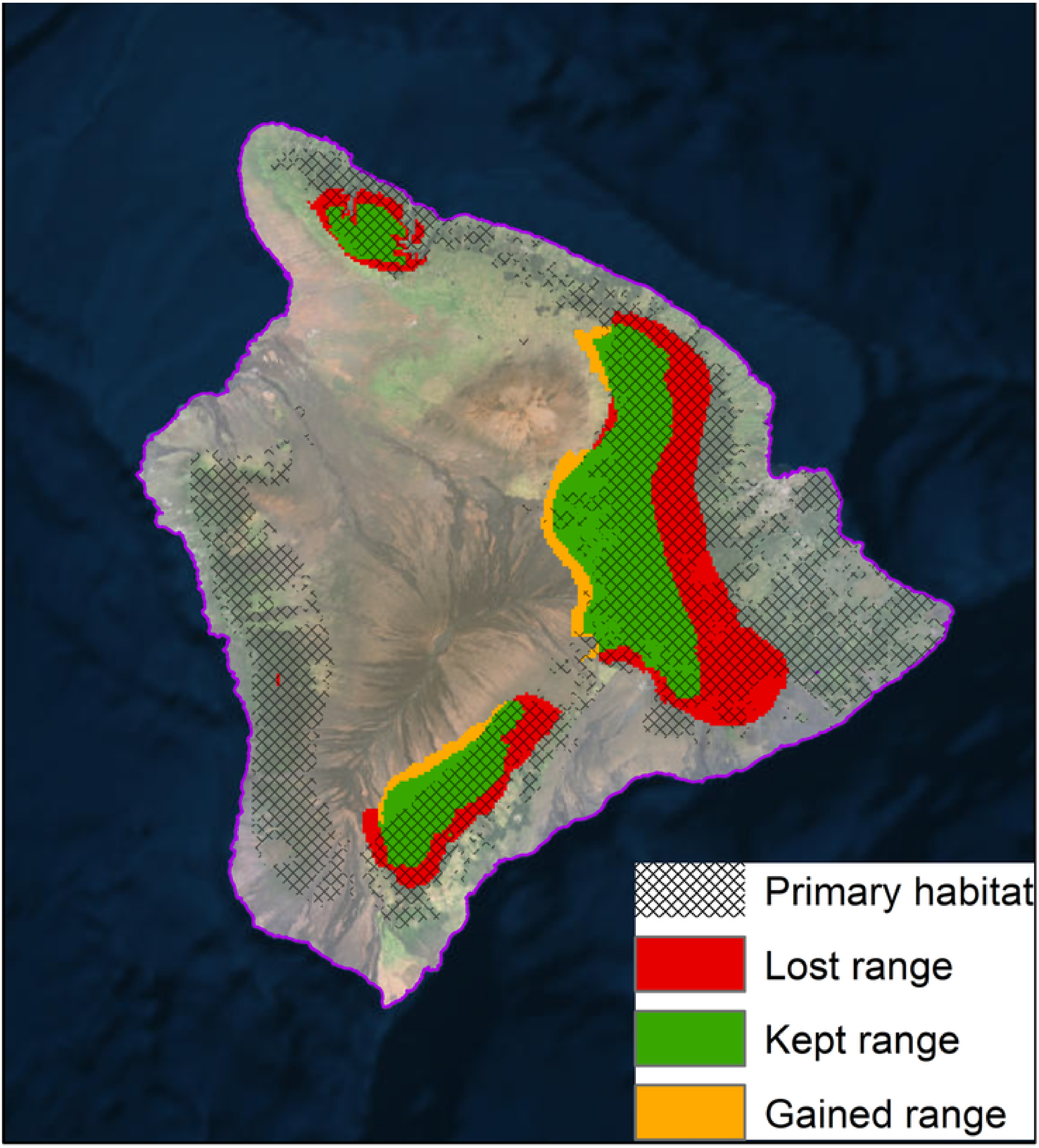

S7: Maps of climate-based species range shifts within primary habitat
REDUCED MODEL RELIABILITY SPECIES

Palila

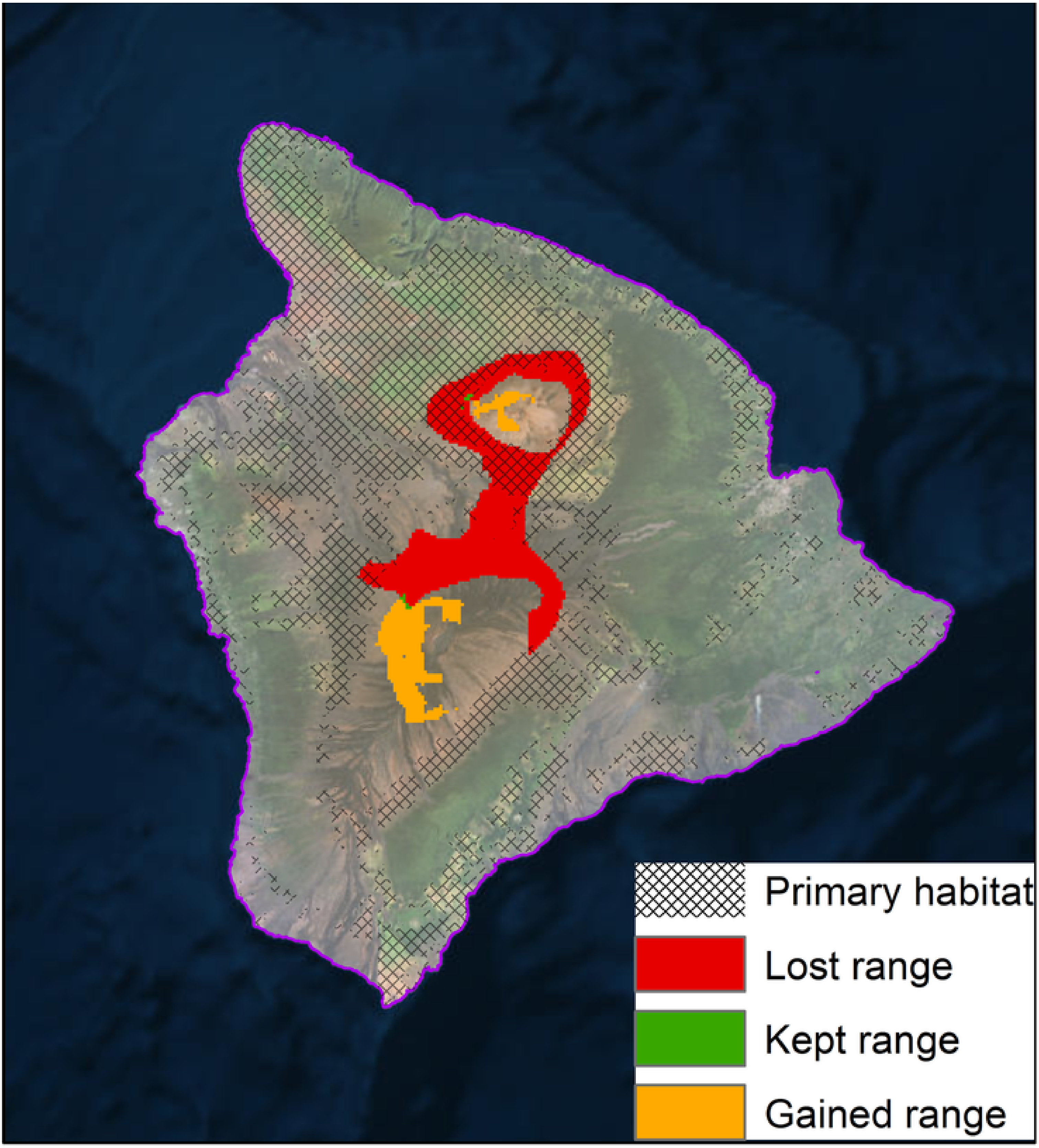

Supplement: S6 File — (PDF) [file pone.0140389.s006.pdf]
