# Supplementary material for: Large-Scale Range Collapse of Hawaiian Forest Birds under Climate Change and the Need 21st Century Conservation Options
Source: PLoS One. 2015 Oct 28;10(10):e0140389. doi: 10.1371/journal.pone.0140389 (PMC4625087; doi:10.1371/journal.pone.0140389)

Appendix S7. Combined species maps showing changes in forest bird diversity.

This appendix has two sets of maps showing the overlap of all forest bird species across the landscape.

The first set includes all 20 modeled species (as the manuscript figures 3-6) while the second set has similar maps but only considering the 10 high model reliability species. Besides copies of figures 3-6, both sets includes maps showing number of species range gain, loss, kept across the landscape.

MAPS INCLUDING ONLY HIGH MODEL RELIABILITY SPECIES

Current (left) and future (right) forest bird richness across Hawai`i based on climate-based modeled range and current availability of primary habitat (Figure 2)

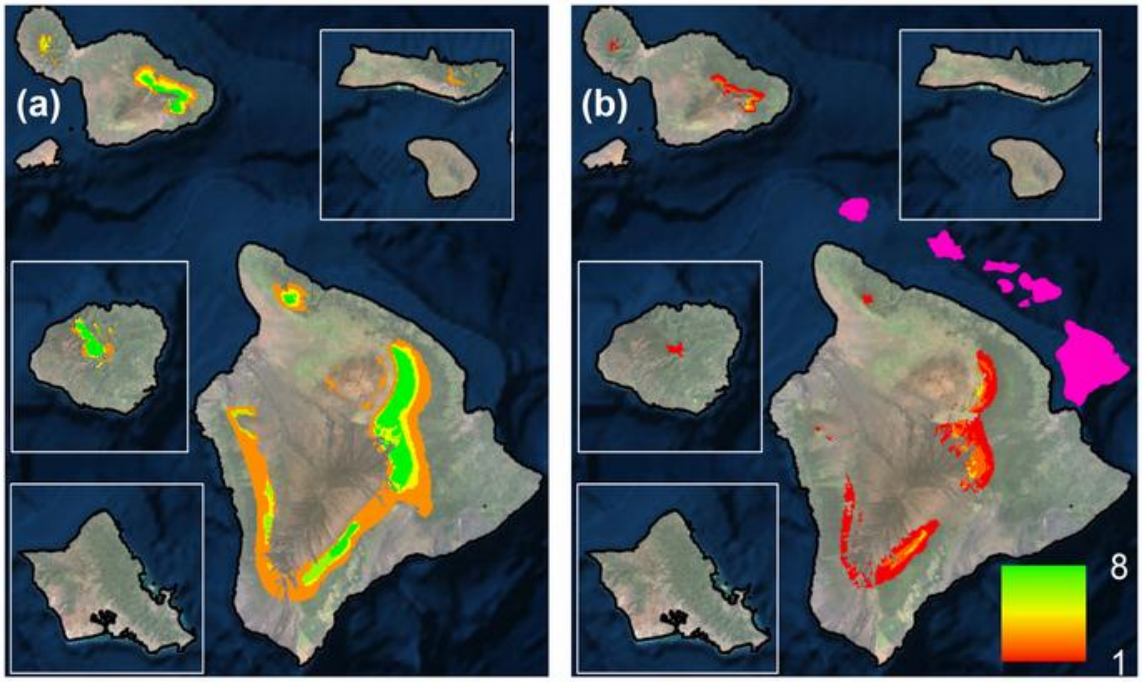

MAPS INCLUDING ONLY HIGH MODEL RELIABILITY SPECIES

Projected number of forest bird species gaining range across Hawai`i with (right) and without (left) consideration of current availability of primary habitat

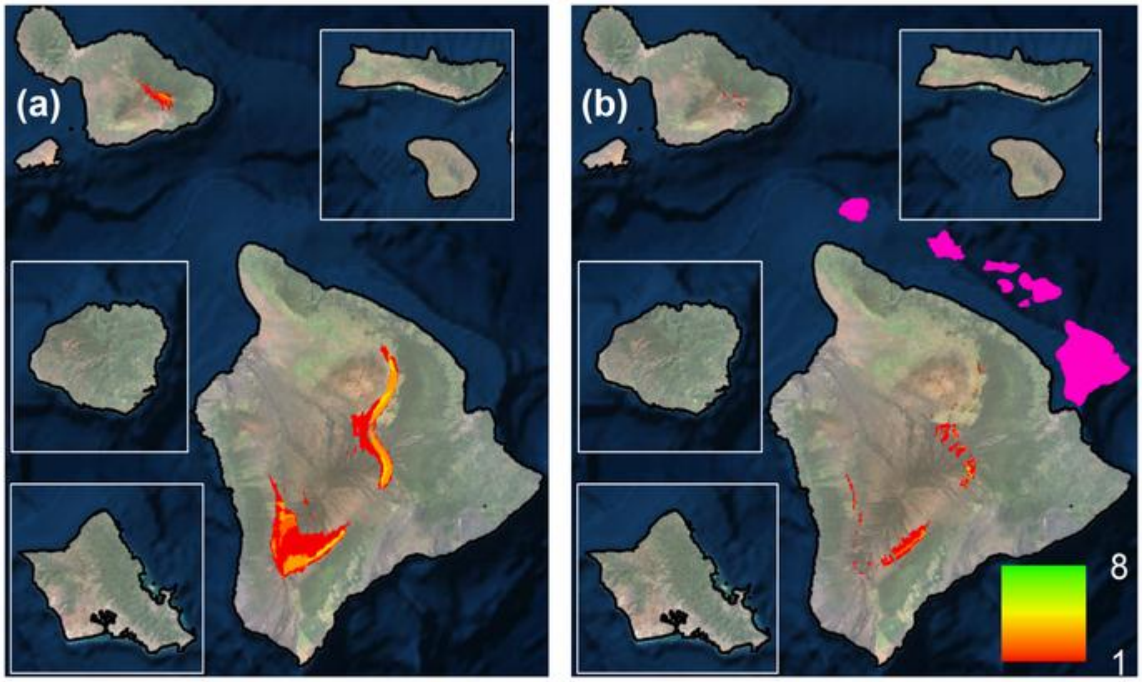

MAPS INCLUDING ONLY HIGH MODEL RELIABILITY SPECIES

Potential forest bird habitat protection priority areas based on the greatest number of species projected to maintain their range between now and end of century. Current protected areas are delineated in green (National parks, State parks, Natural area reserves, wildlife refuges, sea bird sanctuaries, Nature Conservancy lands and other major private conservation areas) (Figure 4)

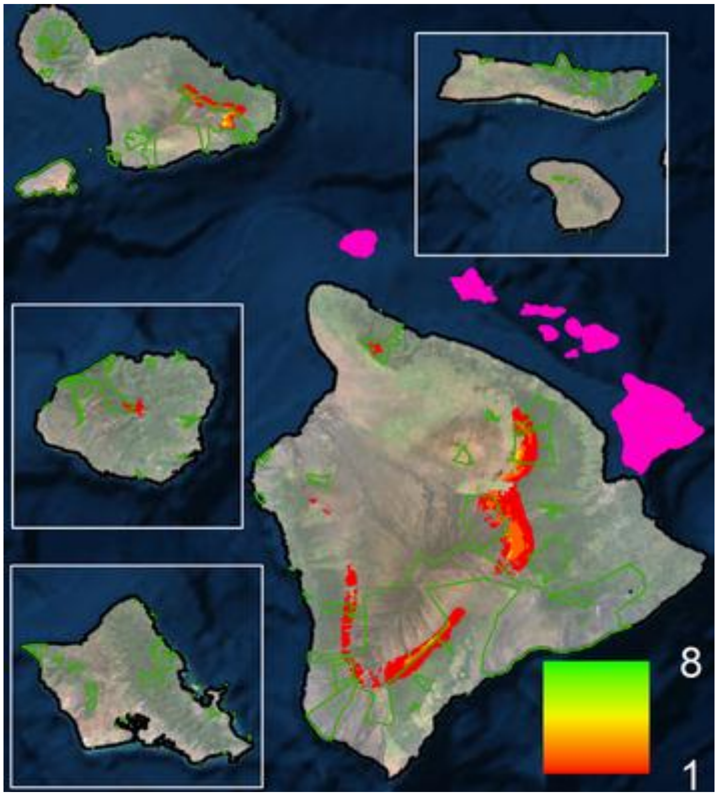

MAPS INCLUDING ONLY HIGH MODEL RELIABILITY SPECIES

Potential forest bird habitat restoration priority areas based on currently converted forest bird habitat in locations remaining climatically suitable for species between now and end of century (Figure 5)

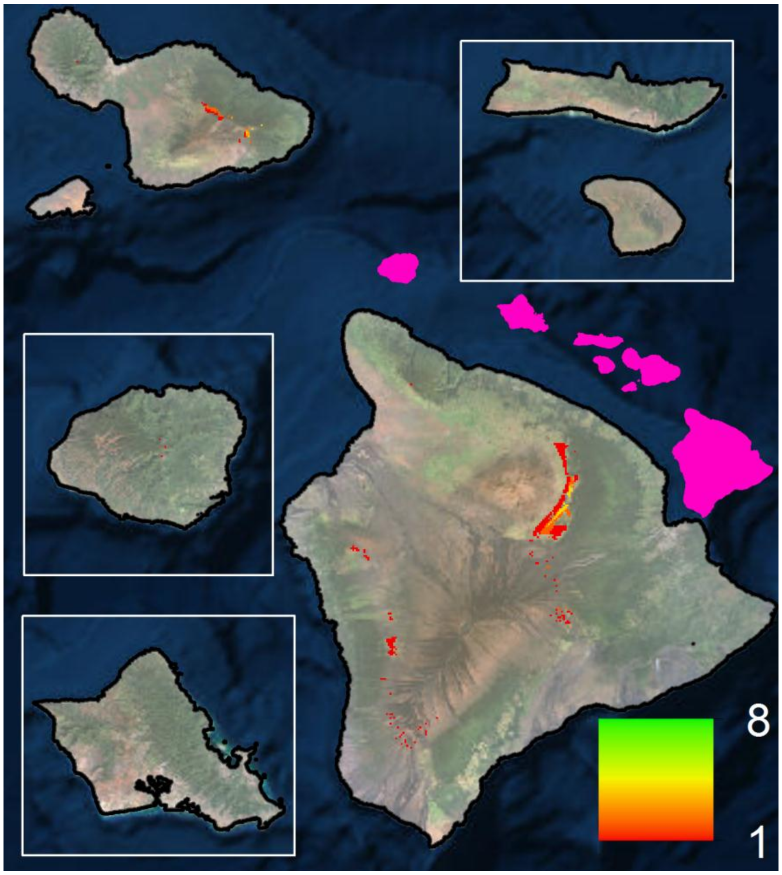

MAPS INCLUDING ONLY HIGH MODEL RELIABILITY SPECIES

Number of native forest bird species gaining climate-based range between baseline and future scenarios within primary habitat for each species

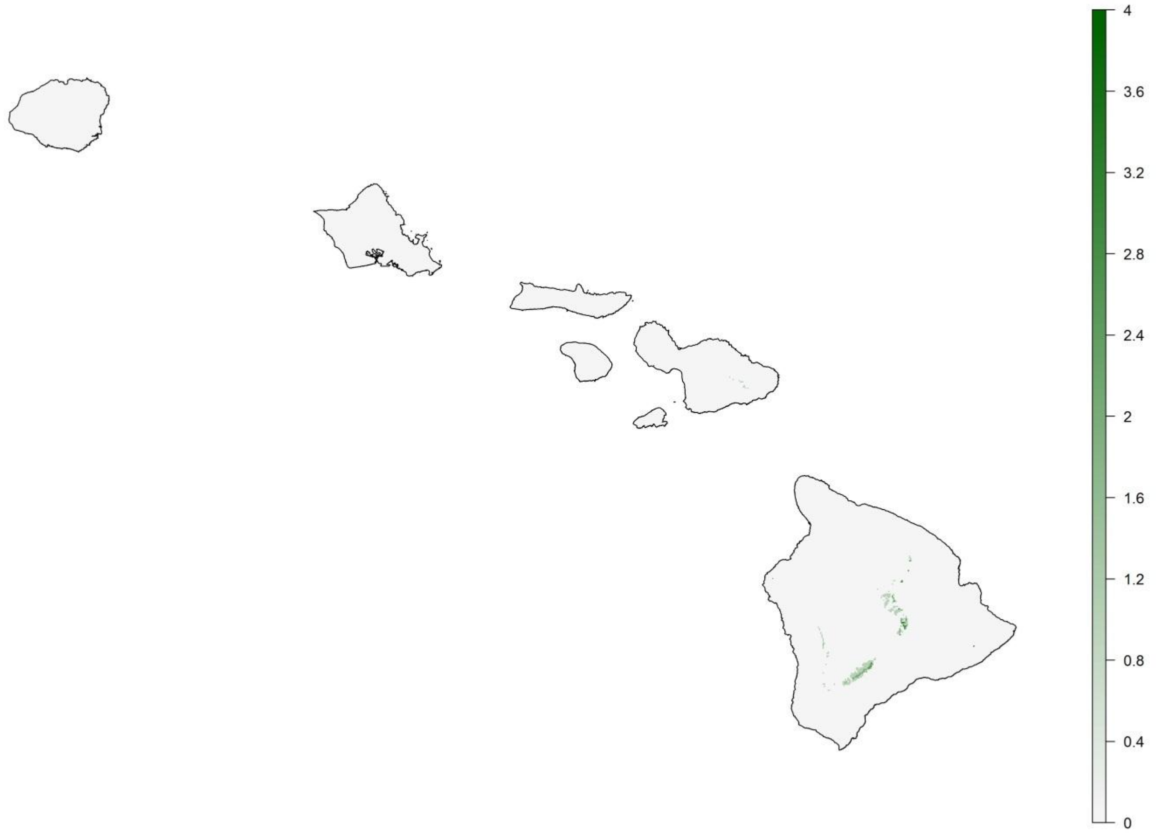

MAPS INCLUDING ONLY HIGH MODEL RELIABILITY SPECIES

Number of native forest bird species keeping climate-based range between baseline and future scenarios within primary habitat for each species

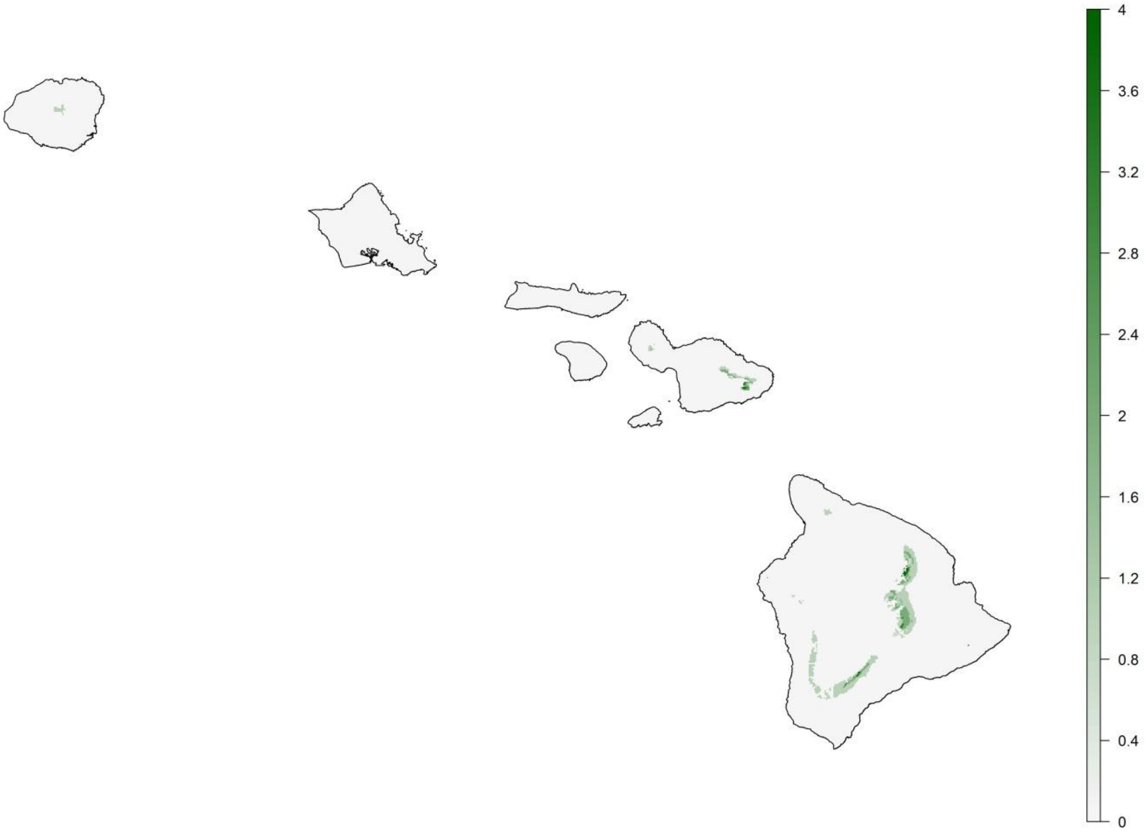

MAPS INCLUDING ONLY HIGH MODEL RELIABILITY SPECIES

Number of native forest bird species losing climate-based range between baseline and future scenarios within primary habitat for each species

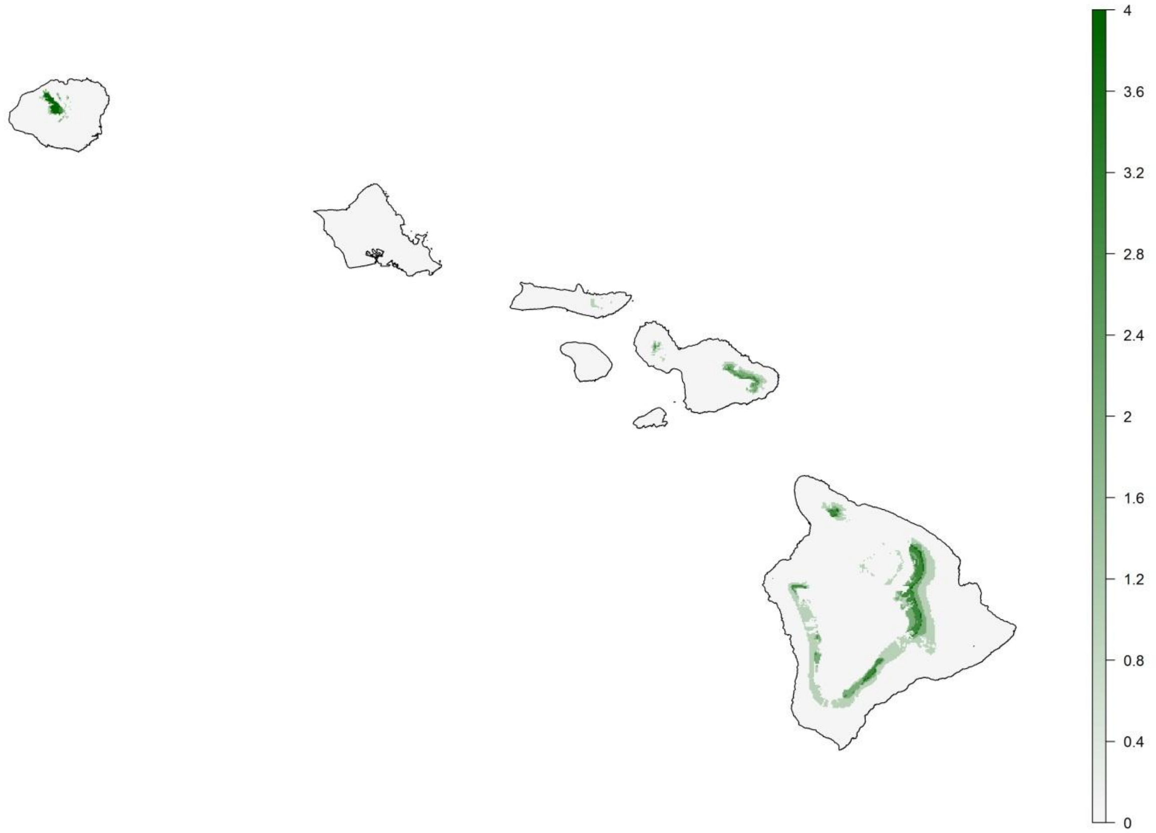

MAPS INCLUDING ALL EXTANT SPECIES

Current (left) and future (right) forest bird richness across Hawai`i based on climate-based modeled range and current availability of primary habitat (Figure 2)

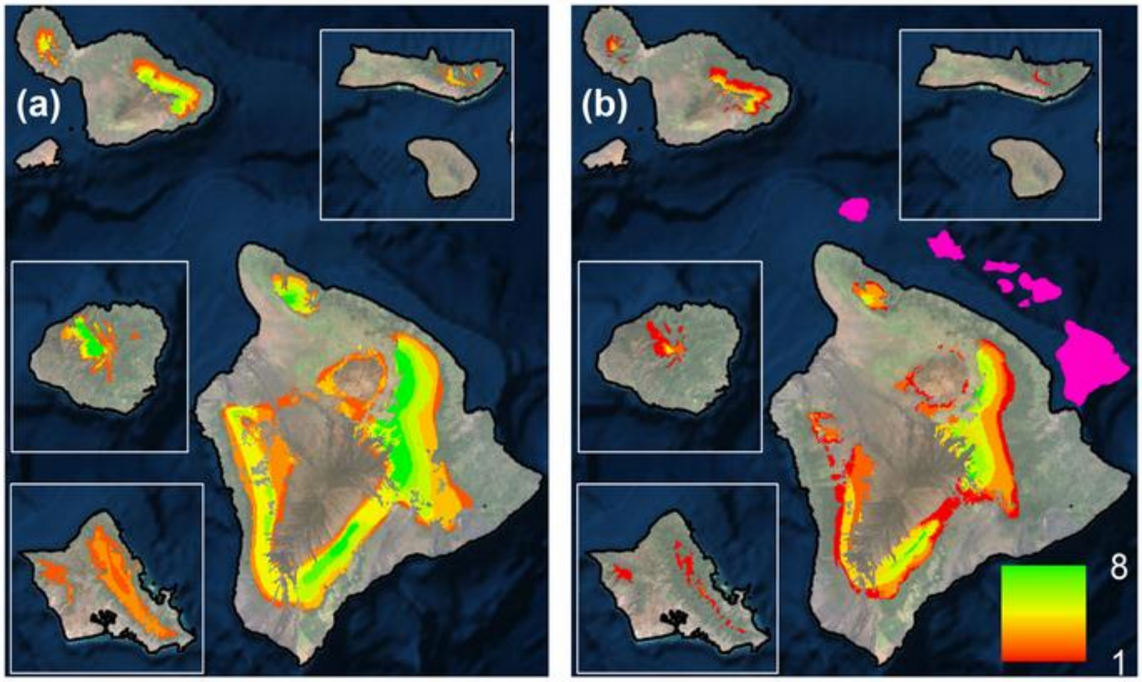

MAPS INCLUDING ALL EXTANT SPECIES

Projected number of forest bird species gaining range across Hawai`i with (right) and without (left) consideration of current availability of primary habitat

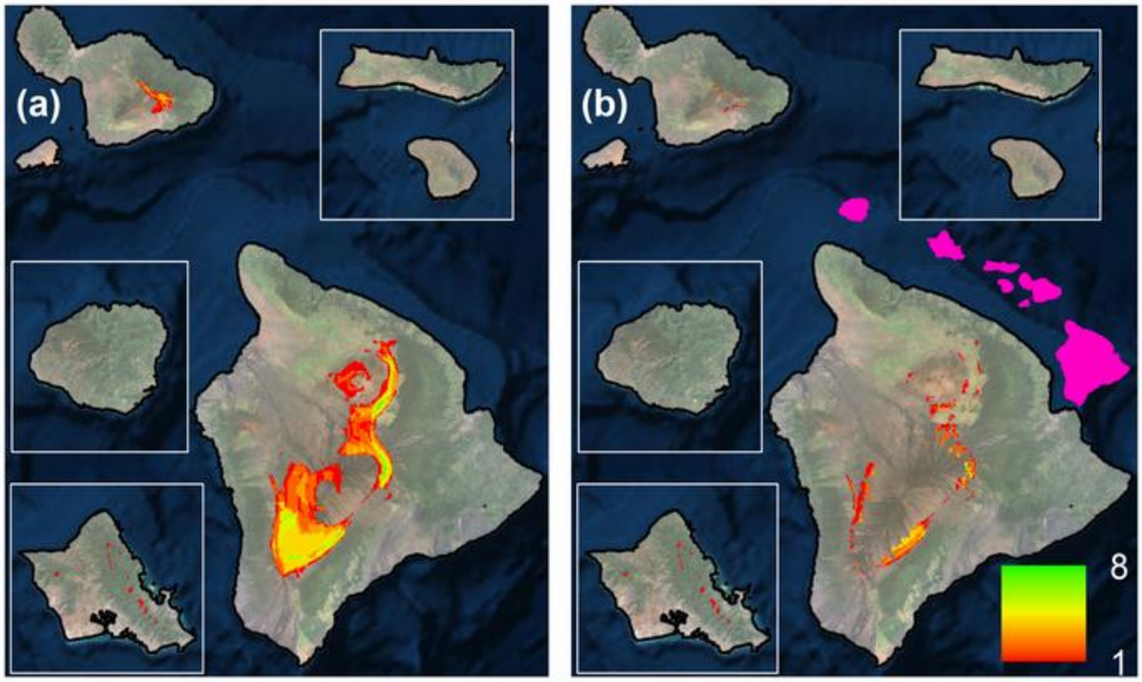

MAPS INCLUDING ALL EXTANT SPECIES

Potential forest bird habitat protection priority areas based on the greatest number of species projected to maintain their range between now and end of century. Current protected areas are delineated in green (National parks, State parks, Natural area reserves, wildlife refuges, sea bird sanctuaries, Nature Conservancy lands and other major private conservation areas) (Figure 4)

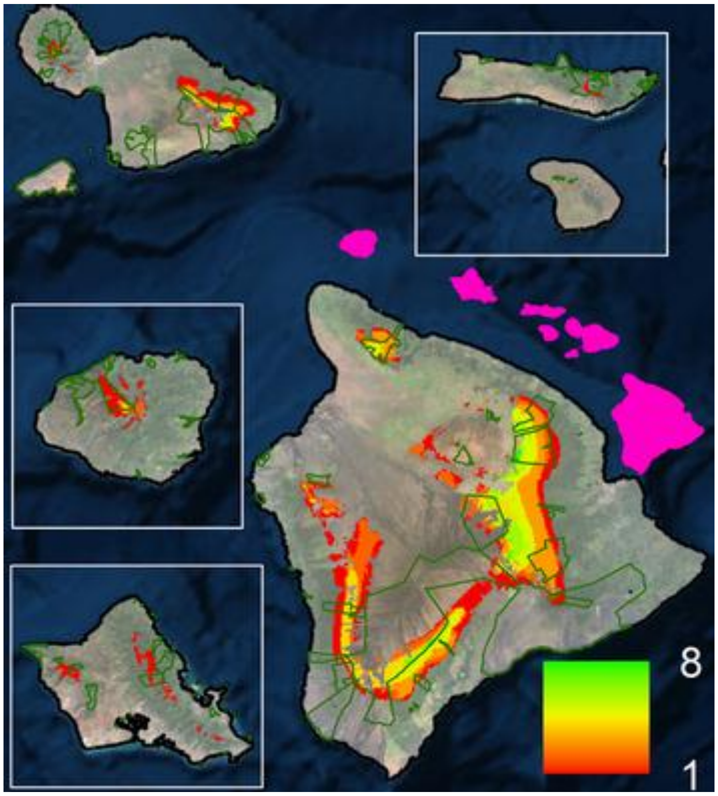

MAPS INCLUDING ALL EXTANT SPECIES

Potential forest bird habitat restoration priority areas based on currently converted forest bird habitat in locations remaining climatically suitable for species between now and end of century (Figure 5)

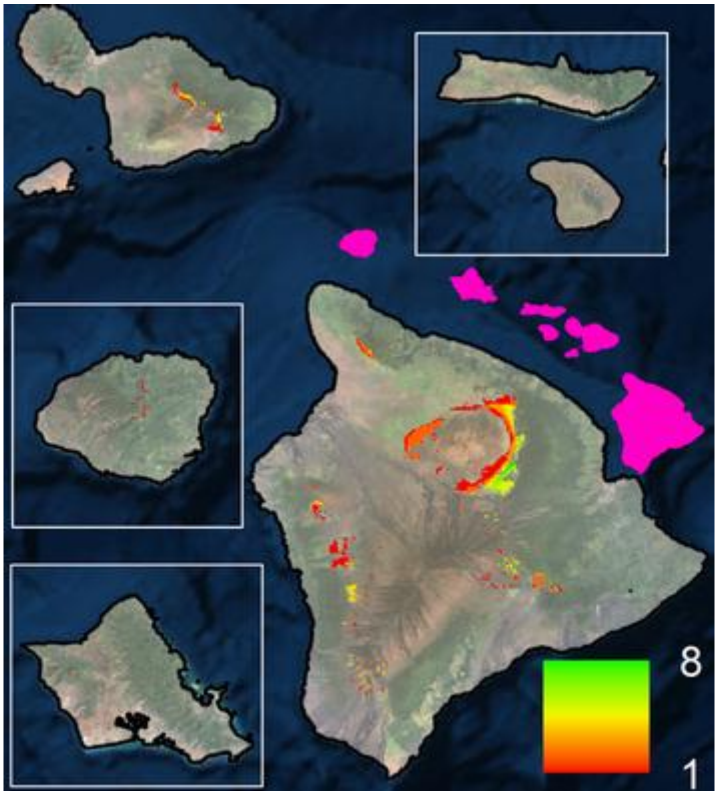

MAPS INCLUDING ALL EXTANT SPECIES

Number of native forest bird species gaining climate-based range between baseline and future scenarios within primary habitat for each species

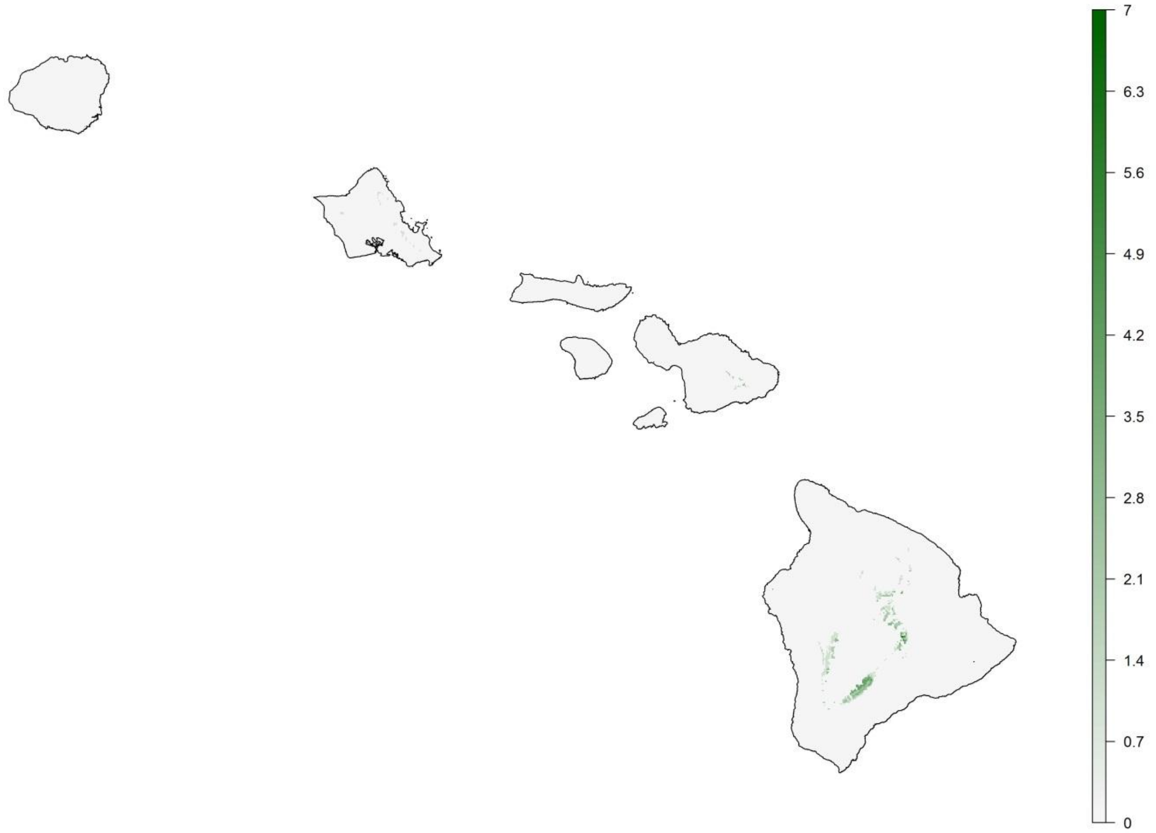

MAPS INCLUDING ALL EXTANT SPECIES

Number of native forest bird species keeping climate-based range between baseline and future scenarios within primary habitat for each species

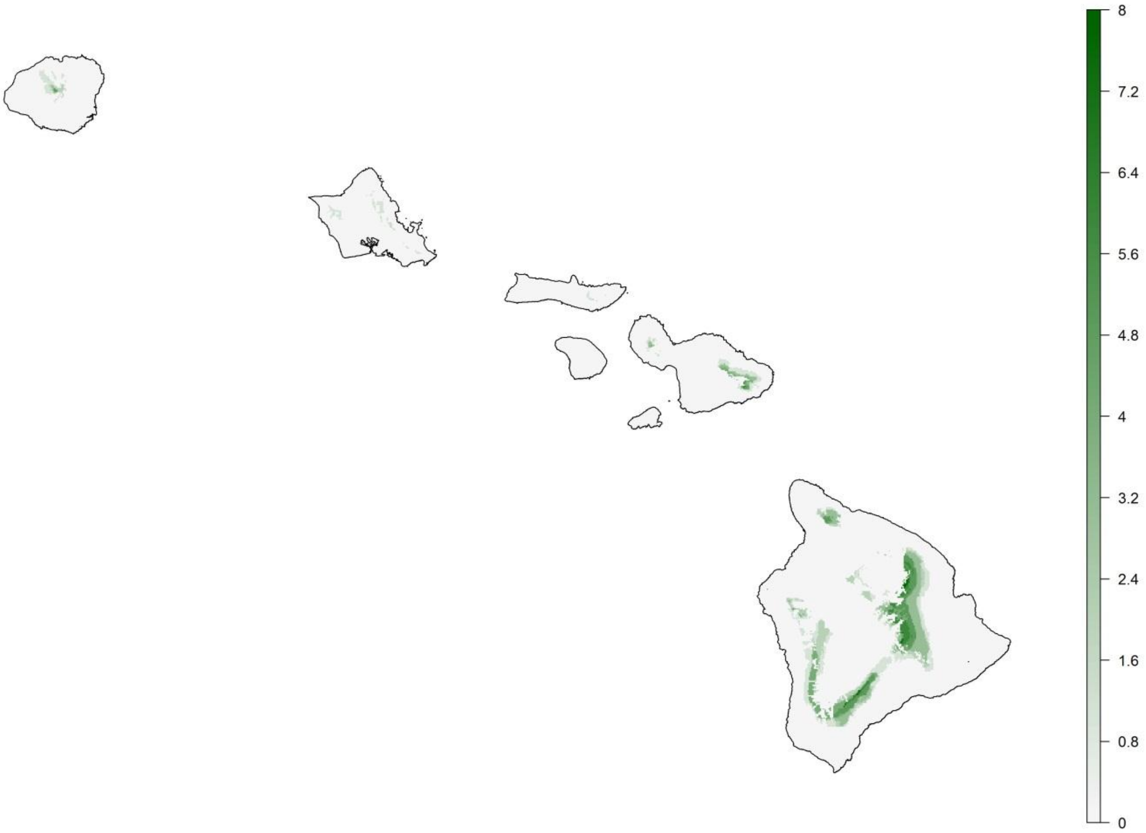

MAPS INCLUDING ALL EXTANT SPECIES

Number of native forest bird species losing climate-based range between baseline and future scenarios within primary habitat for each species

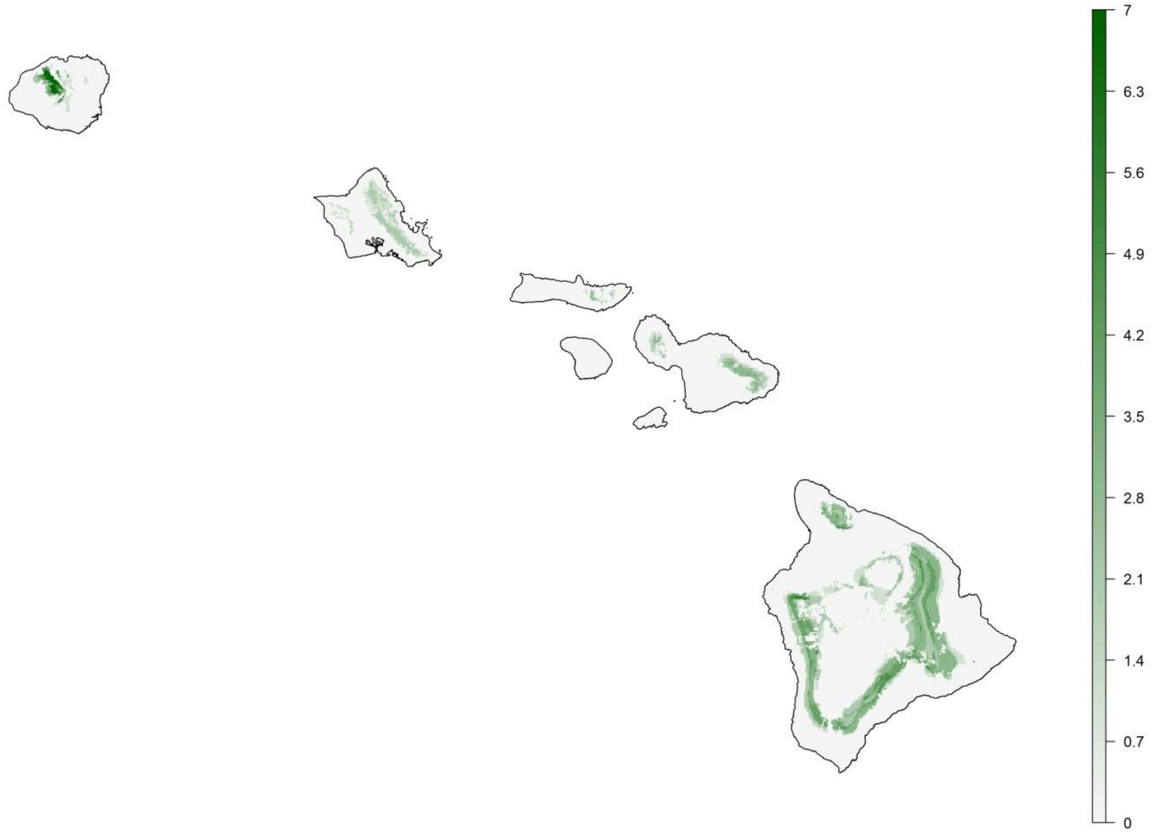

Supplement: S7 File — (PDF) [file pone.0140389.s007.pdf]
