# Supplementary material for: Large-Scale Range Collapse of Hawaiian Forest Birds under Climate Change and the Need 21st Century Conservation Options
Source: PLoS One. 2015 Oct 28;10(10):e0140389. doi: 10.1371/journal.pone.0140389 (PMC4625087; doi:10.1371/journal.pone.0140389)
Supplement: S8 File — (PDF) [file pone.0140389.s008.pdf]

File S8. Projected changes between 1990-2010 and 2080-2100 in range for all Hawaiian forest bird species limited to current available primary habitat. All range estimates are in km^2

Species	Overall model reliability	Baseline range	Future range	% range change	Range lost	Range kept	Range gained
`Akekee	High	70	0	-100	70	0	0
`Akiapōlā`au	High	397	87	-78	381	16	71
`Akikiki	High	52	0	-100	52	0	0
`Ākohekohe	High	44	4	-92.1	41	4	0
Hawai`i `Ākepa	High	422	28	-93.4	404	19	10
Hawai`i Creeper	High	582	134	-76.9	468	114	21
`I`iwi	High	1852	743	-59.9	1214	638	105
Maui `Alauahio	High	102	26	-74.9	76	26	0
Maui Parrotbill	High	69	7	-89.9	62	7	0
Puaiohi	High	49	0	-100	49	0	0
`Anianiau	Reduced	77	4	-94.8	73	4	0
`Apapane	Reduced	2974	1708	-42.6	1346	1628	81
Hawai`i `Amakihi	Reduced	2341	1037	-55.7	1360	981	56
Hawai`i `Elepaio	Reduced	2395	1171	-51.1	1410	985	186
Kauai `Amakihi	Reduced	113	9	-92	104	9	0
Kauai `Elepaio	Reduced	116	7	-93.9	109	7	0

O`ahu `Amakihi	Reduced	257	27	-89.6	230	27	0
O`ahu `Elepaio	Reduced	307	56	-81.8	268	39	17
`Oma`o	Reduced	1541	868	-43.7	681	861	8
Palila	Reduced	215	0	-99.9	214	0	0
